# Supplementary material for: Trehalose-Induced Remodelling of the Human Microbiota Affects Clostridioides difficile Infection Outcome in an In Vitro Colonic Model: A Pilot Study
Source: Front Cell Infect Microbiol. 2021 Jul 2;11:670935. doi: 10.3389/fcimb.2021.670935 (PMC8284250; doi:10.3389/fcimb.2021.670935)
Supplement: Supplementary file 1 [file DataSheet_1.pdf]

Supplementary material

Trehalose-induced remodelling of the human microbiota affects *Clostridioides difficile* infection outcome in an *in vitro* colonic model: a pilot study

Supplementary Table 1. Comparison of bacterial families from the donors and the faecal slurry

Bacterial family	Abundance (%)					
	Slurry	Donor A	Donor B	Donor C	Donor D	Donor E
Methanobacteriaceae	0.042	0.041	0.035	0.111	0.003	0.000
Actinomycetaceae	0.006	0.000	0.001	0.017	0.000	0.007
Bifidobacteriaceae	6.525	5.504	5.701	4.932	0.024	15.795
Coriobacteriaceae	1.387	1.744	1.527	0.292	0.041	3.713
Bacteroidales	7.509	11.808	11.848	0.000	0.000	0.001
Barnesiellaceae	0.050	0.037	0.035	0.031	0.000	0.098
Odoribacteraceae	0.033	0.009	0.012	0.099	0.000	0.057
Paraprevotellaceae	0.955	1.531	1.283	0.000	0.000	0.000
Bacteroidaceae	12.905	7.875	8.530	12.540	20.431	13.921
Porphyromonadaceae	0.660	0.423	0.471	0.664	0.050	1.653
Prevotellaceae	0.693	1.105	0.953	0.000	0.000	0.000
Rikenellaceae	1.689	2.437	1.939	5.256	0.473	0.205
Muribaculaceae	0.006	0.000	0.000	0.144	0.000	0.000
Lactobacillaceae	1.510	0.003	0.004	0.003	9.777	0.000
Streptococcaceae	0.108	0.067	0.075	0.274	0.067	0.204
Turicibacteraceae	0.152	0.170	0.202	0.074	0.000	0.001
Clostridiales	10.576	13.343	14.081	18.764	6.648	11.586
Christensenellaceae	0.942	1.379	1.459	2.986	0.000	0.001
Clostridiaceae	3.998	4.984	4.872	4.056	0.338	0.912
Eubacteriaceae	0.012	0.000	0.000	0.086	0.000	0.000
Lachnospiraceae	13.547	10.156	10.459	1.789	20.529	18.324
Peptococcaceae	0.265	0.487	0.508	0.000	0.000	0.000
Peptostreptococcaceae	0.037	0.049	0.032	0.048	0.000	0.001
Ruminococcaceae	23.302	29.027	27.642	24.854	17.570	26.237
Veillonellaceae	2.158	2.847	2.822	0.888	2.216	1.655
Erysipelotrichaceae	1.422	1.251	1.392	0.331	1.850	2.492
Victivallaceae	0.443	1.002	1.344	0.000	0.000	0.001
Unknown Family - RF32	0.482	0.720	0.523	3.605	0.373	0.027
Alcaligenaceae	0.912	0.597	0.631	0.703	1.100	2.532
Desulfovibrionaceae	0.370	0.669	0.749	0.096	0.024	0.445
Enterobacteriaceae	4.729	0.039	0.068	0.309	16.911	0.041
Pasteurellaceae	0.017	0.061	0.065	0.115	0.001	0.032
Anaeroplasmataceae	0.161	0.396	0.520	0.000	0.000	0.000
Unknown Family - RF39	0.012	0.020	0.018	0.000	0.000	0.000
Unknown Family - ML615J-28	0.105	0.000	0.000	3.686	0.000	0.006
Verrucomicrobiaceae	2.283	0.220	0.200	13.247	1.574	0.051

Supplementary Table 2. Glucose and trehalose concentrations from model G (glucose supplemented), model T (trehalose supplemented), and model S (saline supplemented) (vessels 1-3 for each model). File name: Supplemental Table 2 sugar concentrations.

Supplementary Table 3. Day 38 KEGG pathway analysis from model G (glucose supplemented), model T (trehalose supplemented), and model S (saline supplemented) (vessel 3 for each model). Data shown are KEGG pathway assigned reads from four technical replicates for each model. File name: Supplementary Table 3 KEGG pathway.

Supplementary Table 4. Fold change ratios of significantly different metabolomic pathways and cell surface components between models G and S versus model T. The individual metabolomic pathways and cell surface components listed here were used to populate **Figure 6**.

Supplementary Figure 1. Recovery of bacterial populations from vessel 3 of model S, (S), model G, (G) and model T (T). The bacterial populations enumerated were, total obligate anaerobic bacteria (black lines), lactose-fermenting *Enterobacteriaceae* (red lines), *Clostridium* spp. (green lines), *Lactobacillus* spp. (blue lines), and *C. difficile* total counts (indigo lines). Results expressed as mean ± SD of three technical replicates. Horizontal blue arrow represents the period of clindamycin dosing to all gut models.

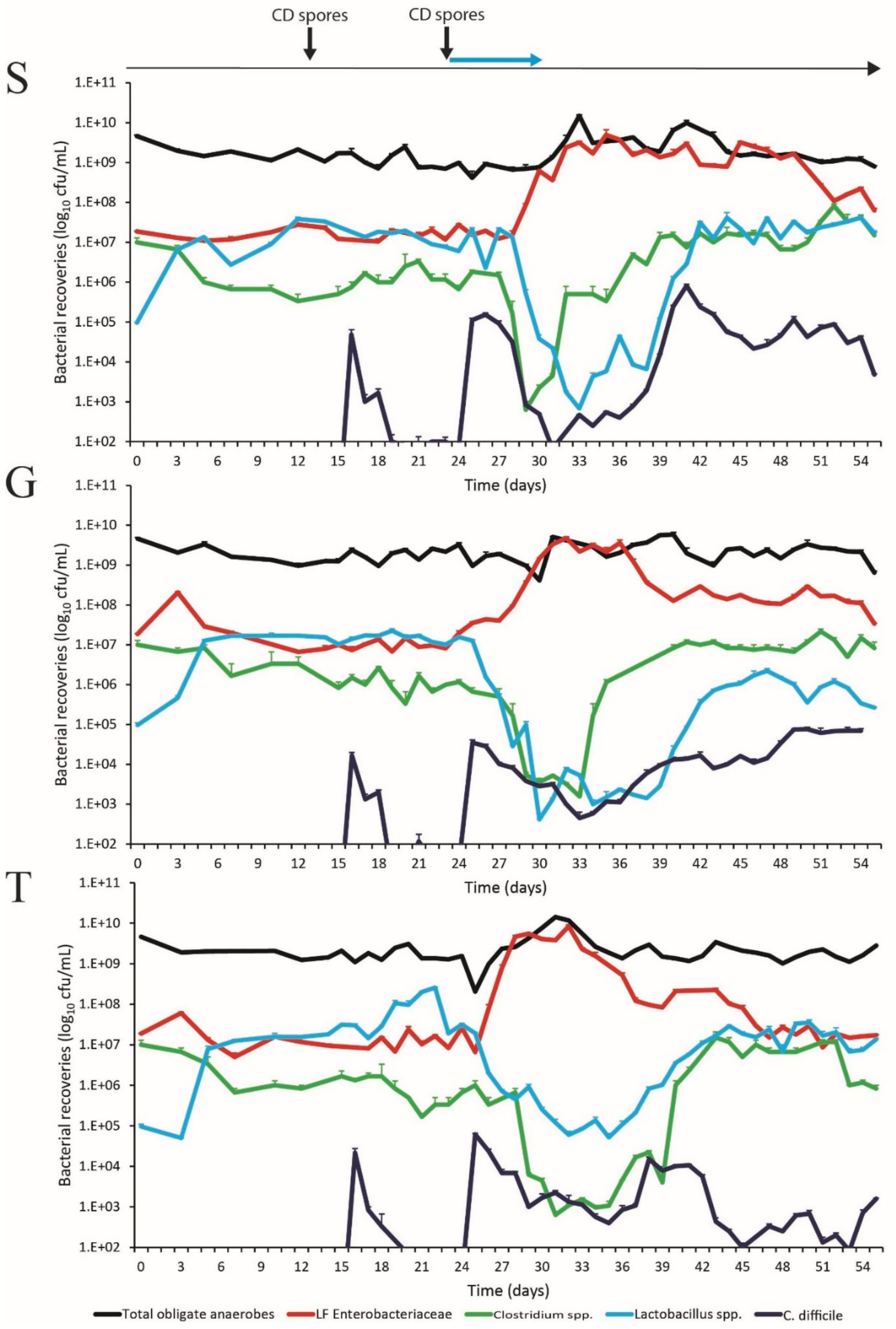

Supplementary Figure 2. Biosynthesis of amino acids (KEGG pathway Ko01230), with metabolic compounds produced from pathways that are most abundant in both model G and model S (CDI induction model) shown in red.

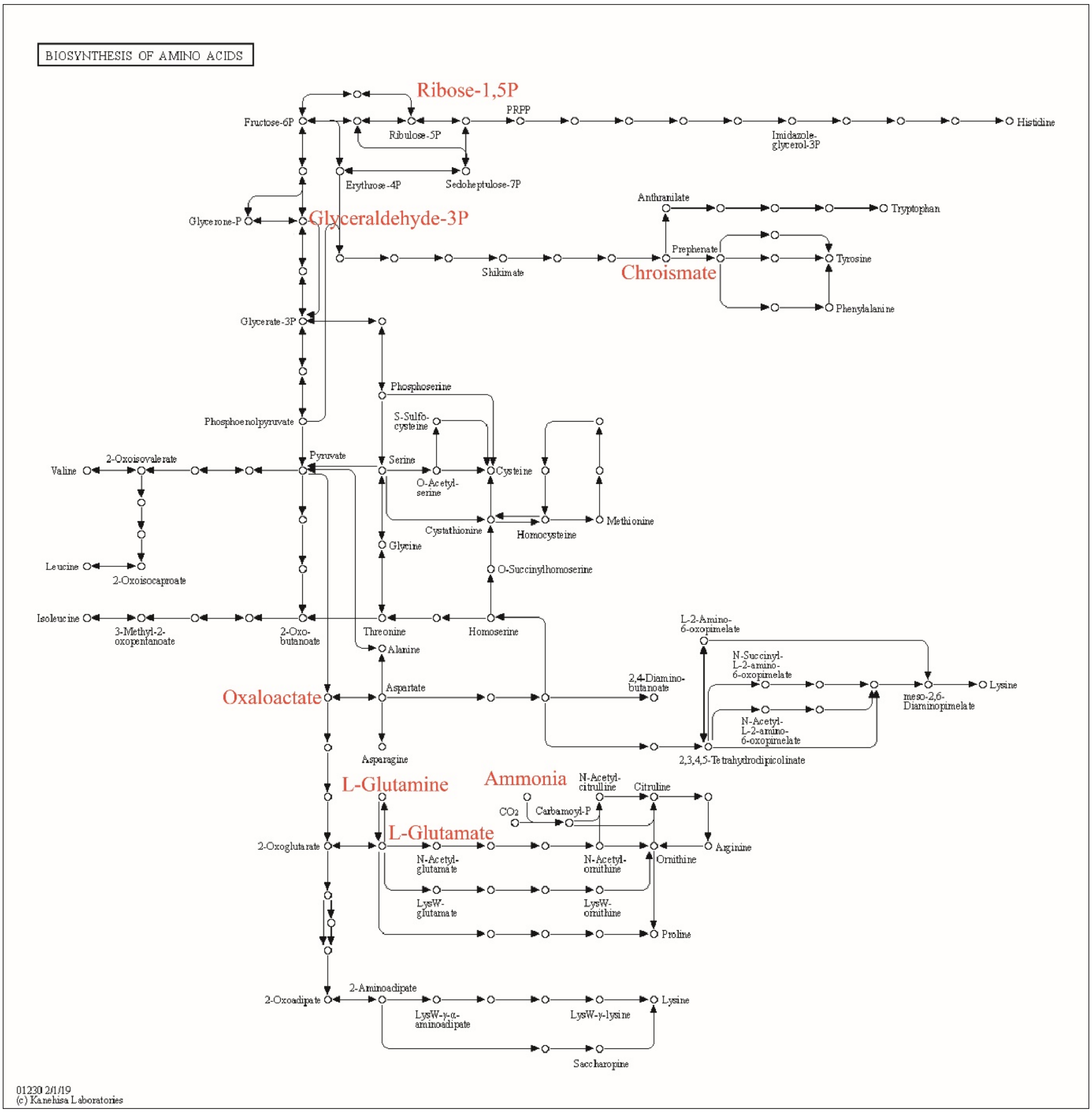

Supplementary Figure 3. Putative mechanisms of trehalose-induced protection against *C. difficile* infection.

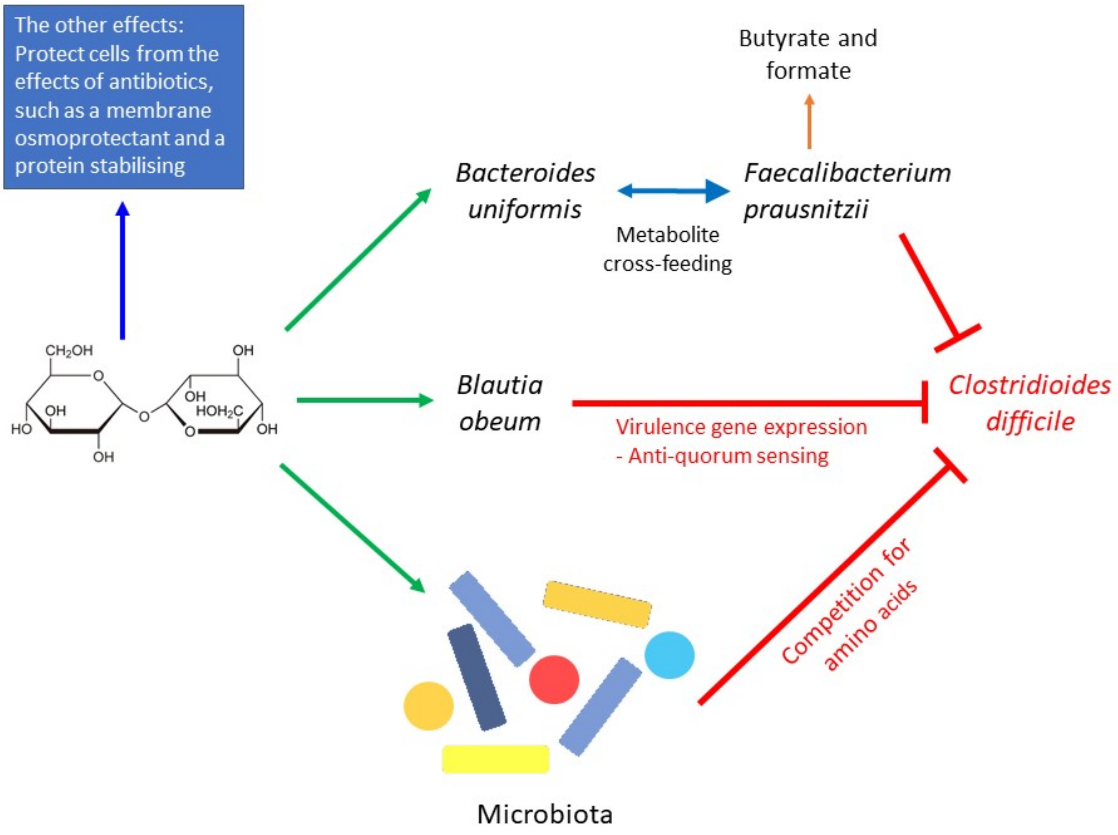

Supplementary methods

Enumeration of endogenous bacteria

Models were sampled for culture profiling of key intestinal microbiota populations using selective and non-selective agars described in **Supplementary Table 4**. Populations of total bacteria, *Clostridium* spp. lactose-fermenting Enterobacteriaceae, *Enterococcus* spp., *Bacteroides* spp., *Bifidobacterium* spp., *Lactobacillus* spp., *C. difficile* total viable cells and *C. difficile* spores. *C. difficile* spores were isolated by treating 0.5 mL of gut model fluid with 0.5 mL of 96% ethanol. The samples were incubated at room temperature for 1 h, serially diluted to 10–3 in peptone water, and 20 µL of each sample dilution was plated in triplicate onto supplemented Braziers CCEY agar (**Supplementary Table 3**). Plates were incubated anaerobically for 48 h and distinctive colonies were enumerated and identified based on colony morphology and Matrix-Assisted Laser Desorption Ionization-Time of Flight (MALDI-TOF) identification. Each bacterial population was measured in triplicate (three technical replicates of a single biological replicate) in vessels 2 and 3. The limit of detection for either total viable counts or spores were 1.2 or l.5, respectively, log_{10} cfu/mL.

Supplementary Table 4. Target populations and agar composition for bacterial enumeration.

Target populations	Agar	Supplements	Incubation (temp, environment)
Total anaerobes and total *Clostridium* spp.	Fastidious anaerobe agar	5% horse blood	37ºC, anerobic
Bifidobacterium spp.	42.5 g/L Columbia agar, and 5 g/L agar technical	0.5 g/L cysteine HCl, 5 g/L glucose	37ºC, anerobic
Bacteroides spp.	Bacteroides bile aesculin agar	5mg/L haemin, 10 µL/L vitamin K, 7.5 mg/L vancomycin, 1 mg/L penicillin, 75 mg/L kanamycin and 10 mg/L colistin	37ºC, anerobic
Lactobacillus spp.	52.2 g/L MRS broth and 20 g/L agar technical	0.5 g/L cysteine hydrocloride, 20 mg/L vancomycin	37ºC, anerobic
Total facultative anaerobes	Nutrient agar	N/A	37ºC, aerobic
Lactose fermenting Enterobacteriaceae	MaConkey's agar	N/A	37ºC, aerobic
Enterococcus spp.	*Kanamycin* aesculin *azide agar*	10 mg/L nalidixic acid, 10 mg/L aztreonam, and 20 mg/L kanamycin	37ºC, aerobic
Total spores (following alcohol shock for 1 hour)	Fastidious anaerobe agar	5% horse blood	37ºC, anerobic
C. difficile total viable cells	Braziers CCEY agar	D-cycloserine (250mg/L) cefoxitin (8mg/L), 5 mg/L lysozyme, and 20 mL/L lysed horse blood	37ºC, anerobic
C. difficile spores	Braziers CCEY agar	5 mg/L lysozyme, and 2% lysed horse blood	37ºC, anerobic

Supplementary Table 5. Ion chromatography conditions used to detect glucose and trehalose

Eluent Buffer

A: Water, B; 300 mM NaOH, C; 300 mM NaOH/1,500 mM sodium acetate

Time (min)	Flow (ml/min)	A%	B%	C%	
-10	1	86.8	13.2	0.0	
0	1	86.8	13.2	0.0	
15	1	86.8	13.2	0.0	Linear gradient
25	1	67.0	0.0	33.0	
30	1	67.0	0.0	33.0	
