# Supplementary material for: Trehalose-Induced Remodelling of the Human Microbiota Affects Clostridioides difficile Infection Outcome in an In Vitro Colonic Model: A Pilot Study
Source: Front Cell Infect Microbiol. 2021 Jul 2;11:670935. doi: 10.3389/fcimb.2021.670935 (PMC8284250; doi:10.3389/fcimb.2021.670935)
Supplement: Supplementary file 2 [file DataSheet_2.pdf]

Experimental period	Day	Trehalose concentration (mM)									Glucose concentration (mM)								
		Model G			Model T			Model S			Model G			Model T			Model S		
		V1	V2	V3	V1	V2	V3	V1	V2	V3	V1	V2	V3	V1	V2	V3	V1	V2	V3
CD spore reservoir	14	ND	ND	ND	5.2446	0.4353	ND	ND	0.0003	ND	6.98	0.0011	0.0021	0.0498	0.0253	0.0007	0.001	0.0007	0.0006
	15	ND	ND	ND	8.0957	ND	ND	ND	ND	ND	16.1177	0.0011	0.0017	0.0567	0.0003	0.0019	0.0017	0.0014	0.0066
	16	0.0128	ND	0.0004	4.1587	4.3267	ND	ND	ND	ND	0.1465	0.0067	0.0187	0.1018	ND	ND	ND	ND	ND
	17	0.0073	ND	0.0005	0.3459	0.0006	ND	ND	ND	0.0004	0.1272	0.0042	0.0029	0.0185	0.0018	0.0048	0.001	0.0009	0.0012
	18	ND	ND	ND	4.2475	ND	ND	ND	ND	ND	0.1478	0.0033	ND	0.0839	0.0018	0.0017	0.007	0.0021	0.0012
	19	ND	ND	ND	1.8968	ND	ND	ND	ND	ND	0.1343	0.0076	0.0014	0.1077	0.0012	0.0013	0.0009	0.0013	0.0009
	20	ND	ND	ND	0.3459	ND	ND	ND	ND	ND	0.0189	0.0108	0.0051	ND	0.0017	0.0024	0.0013	0.0014	0.001
	21	ND	ND	ND	ND	ND	ND	ND	ND	ND	0.0147	0.0034	0.0017	ND	0.001	0.0017	0.0015	0.0013	0.001
	22	ND	ND	ND	0.0502	ND	ND	ND	ND	ND	0.011	0.0015	0.0014	0.0025	0.0015	0.0011	0.0009	0.0014	0.0011
	23	ND	ND	0.0004	ND	ND	ND	ND	ND	ND	0.0009	ND	0.0235	0.0006	0.0012	0.0007	0.0015	0.001	0.0014
Clindamycin	24	ND	ND	ND	3.6687	ND	ND	ND	ND	ND	4.0395	ND	0.005	0.03	0.0015	0.0008	0.0014	0.0015	0.0013
	25	0.0583	ND	ND	11.7116	0.8146	ND	0.2221	ND	ND	28.252	0.3285	ND	0.4015	0.0163	0.0011	1.3918	0.0166	0.0016
	26	0.2536	0.007	ND	13.3425	6.6753	0.9993	0.4172	0.2168	0.021	27.8272	2.548	0.0147	1.6282	0.1154	0.0395	1.8408	0.5932	0.1239
	27	0.1788	ND	ND	8.2611	5.8113	2.5191	ND	ND	ND	20.1018	0.0954	0.0141	0.0552	0.1604	0.2399	0.0065	0.0027	0.0015
	28	0.0091	0.0127	ND	2.0596	ND	ND	ND	ND	ND	11.3207	0.0092	0.0051	0.059	0.0075	0.0024	0.0019	0.0013	0.001
	29	0.0048	ND	ND	2.8235	ND	ND	ND	ND	ND	8.9286	0.0046	0.008	0.1393	0.0042	0.0026	0.0013	ND	ND
	30	ND	ND	ND	ND	ND	ND	ND	ND	ND	ND	ND	ND	0.0016	0.0008	0.0009	0.0012	0.0047	0.0038
	31	ND	ND	ND	2.054	ND	ND	ND	ND	ND	6.7163	0.0032	0.001	0.1317	0.0016	0.0015	0.0049	ND	ND
Simulated CDI	32	ND	ND	ND	0.2552	ND	ND	ND	ND	ND	6.1944	0.0022	0.0007	0.1165	0.0049	0.0019	0.0051	ND	ND
	33	ND	ND	ND	ND	ND	ND	ND	ND	ND	0.0014	0.0021	0.0009	0.0046	0.002	0.0012	0.0078	0.0008	ND
	34	ND	ND	ND	1.1969	ND	ND	ND	ND	ND	7.0011	0.0021	0.001	0.2422	0.0042	0.0014	0.0053	0.0012	ND
	35	ND	ND	ND	ND	ND	ND	ND	ND	ND	0.0008	0.0137	0.0033	ND	0.0145	ND	0.0046	ND	ND
	36	ND	ND	ND	0.0112	ND	ND	ND	ND	ND	2.6103	0.0334	0.0168	0.0843	0.0652	0.0018	0.1078	0.001	ND
	37	ND	ND	ND	ND	ND	ND	ND	ND	ND	0.0009	0.0445	0.0116	0.0006	0.0129	0.0312	ND	0.001	ND
	38	ND	ND	ND	ND	ND	ND	ND	ND	ND	0.0017	0.0023	0.0013	0.001	0.0025	0.0009	0.0077	0.0018	0.0019
	39	ND	ND	ND	0.5673	ND	ND	ND	ND	ND	2.2232	ND	0.0009	0.6585	0.0007	0.0006	0.0089	0.0008	0.0009
	40	ND	ND	ND	2.1222	ND	ND	ND	ND	ND	6.8236	0.0019	0.0019	1.4805	ND	ND	0.0032	0.0011	0.0009
	41	ND	ND	ND	1.2728	ND	ND	ND	ND	ND	0.0031	0.0058	ND	0.0011	1.6883	0.0009	0.0081	0.0011	0.0018
	42	ND	ND	ND	2.2694	ND	ND	ND	ND	ND	0.0026	0.0058	0.0007	1.0114	0.0012	0.0019	0.0081	0.0009	0.0012
	43	ND	ND	ND	ND	ND	ND	ND	ND	ND	0.0065	0.0156	0.0022	0.0016	0.0011	0.0016	0.0035	0.0009	0.001
	44	ND	ND	ND	4.6967	ND	ND	ND	ND	ND	8.447	0.0145	0.0031	0.5768	0.0196	0.002	0.0044	0.001	0.0014
	45	ND	ND	ND	1.5605	ND	ND	ND	ND	ND	0.0102	0.0175	0.0087	0.337	0.0016	0.0022	0.0016	0.0013	0.0014
	46	ND	ND	ND	ND	ND	ND	ND	ND	ND	0.0046	0.0099	0.0027	0.0029	0.0126	0.0014	0.0013	0.0008	0.0007
	47	ND	ND	ND	ND	ND	ND				ND	ND	ND	ND	ND	ND			
	48	ND	ND	ND	ND	ND	ND				0.0022	0.0015	0.0013	0.0041	0.0008	0.0015			
	49	ND	ND	ND	ND	ND	ND				0.0016	0.0013	0.0009	0.003	0.0011	0.001			
	50	ND	ND	ND	ND	ND	ND				0.0027	0.0018	0.0016	0.0077	0.0009	0.0016			
