# Supplementary material for: Trehalose-Induced Remodelling of the Human Microbiota Affects Clostridioides difficile Infection Outcome in an In Vitro Colonic Model: A Pilot Study
Source: Front Cell Infect Microbiol. 2021 Jul 2;11:670935. doi: 10.3389/fcimb.2021.670935 (PMC8284250; doi:10.3389/fcimb.2021.670935)
Supplement: Supplementary file 3 [file DataSheet_3.pdf]

	Saline model				Trehalose model				Glucose model			
#Datasets	SA D38	SB D38	SC D38	SD D38	TA D38	TB D38	TC D38	TD D38	GA D38	GB D38	GC D38	GD D38
KEGG	9253574	9230269	9114938	9229998	8411070	8468258	8365147	8417468	8612545	8645575	8550853	8644083
Metabolism	3324452	3323200	3279165	3319148	3197007	3210979	3186753	3196959	3255493	3256410	3228229	3257346
Carbohydrate metabolism	1110504	1115680	1106843	1118697	1179116	1186477	1181704	1176146	1229890	1238874	1228443	1236169
ko00010 Glycolysis / Gluconeogenesis	173787	174835	172940	174648	172844	175917	173790	173529	184557	186962	184294	185290
K00001 alcohol dehydrogenase [EC:1.1.1.1]	1456	1456	1473	1402	1185	1259	1208	1141	1839	1925	1870	1902
K00002 alcohol dehydrogenase (NADP+) [EC:1.1.1.2]	174	169	149	148	43	37	52	65	306	308	336	335
K00016 L-lactate dehydrogenase [EC:1.1.1.27]	1548	1452	1512	1681	610	687	665	615	1685	1888	1825	1772
K00114 alcohol dehydrogenase (cytochrome c) [EC:1.1.2.8]	2545	2673	2492	2486	1293	1237	1225	1497	867	813	764	751
K00121 S-(hydroxymethyl)glutathione dehydrogenase / alcohol dehydrogenase [EC:1.1.1.284 1.1.1.1]	2258	2350	2107	2222	1410	1419	1333	1335	1558	1506	1505	1497
K00128 aldehyde dehydrogenase (NAD+) [EC:1.2.1.3]	5102	5084	4807	4818	3106	2867	2785	3017	1916	1754	1686	1825
K00129 aldehyde dehydrogenase (NAD(P)+) [EC:1.2.1.5]	16	16	10	10	14	12	15	10	3	4	6	4
K00131 glyceraldehyde-3-phosphate dehydrogenase (NADP+) [EC:1.2.1.9]	2124	2160	1918	2096	1063	1014	920	1156	658	722	700	712
K00134 glyceraldehyde 3-phosphate dehydrogenase [EC:1.2.1.12]	6507	6759	6631	6623	5652	5731	5693	5624	5233	5374	5245	5450
K00138 aldehyde dehydrogenase [EC:1.2.1.-]	4815	4669	4149	4632	2677	2513	2491	2640	2411	2391	2308	2483
K00149 aldehyde dehydrogenase family 9 member A1 [EC:1.2.1.47 1.2.1.3]	1	1	0	0	0	0	0	0	0	0	0	0
K00150 glyceraldehyde-3-phosphate dehydrogenase (NAD(P)) [EC:1.2.1.59]	0	2	2	1	0	3	2	3	4	12	6	13
K00161 pyruvate dehydrogenase E1 component alpha subunit [EC:1.2.4.1]	1703	1663	1418	1432	800	804	841	919	537	508	532	580
K00162 pyruvate dehydrogenase E1 component beta subunit [EC:1.2.4.1]	1648	1616	1373	1463	827	781	798	906	569	462	495	495
K00163 pyruvate dehydrogenase E1 component [EC:1.2.4.1]	3440	3384	3516	3516	2237	2394	2149	2183	2815	3102	2675	2964
K00169 pyruvate ferredoxin oxidoreductase alpha subunit [EC:1.2.7.1]	216	258	255	233	319	373	370	340	472	523	516	565
K00170 pyruvate ferredoxin oxidoreductase beta subunit [EC:1.2.7.1]	87	91	115	86	271	291	310	317	194	186	215	227
K00171 pyruvate ferredoxin oxidoreductase delta subunit [EC:1.2.7.1]	5	16	4	7	66	59	58	50	24	29	38	34
K00172 pyruvate ferredoxin oxidoreductase gamma subunit [EC:1.2.7.1]	92	139	139	122	156	171	181	166	270	280	293	294
K00174 2-oxoglutarate/2-oxoacid ferredoxin oxidoreductase subunit alpha [EC:1.2.7.3 1.2.7.11]	2742	2915	3097	2864	6669	6734	6879	6802	5579	5516	5922	5513
K00175 2-oxoglutarate/2-oxoacid ferredoxin oxidoreductase subunit beta [EC:1.2.7.3 1.2.7.11]	1687	1853	2015	1903	4182	4181	4270	4209	3675	3591	3674	3509
K00382 dihydrolipoamide dehydrogenase [EC:1.8.1.4]	9170	9150	8930	9118	9722	9692	9339	9543	8930	8583	8861	8866
K00627 pyruvate dehydrogenase E2 component (dihydrolipoamide acetyltransferase) [EC:2.3.1.12]	5358	5056	4803	4842	2778	2862	2887	3162	3520	3384	3306	3636
K00844 hexokinase [EC:2.7.1.1]	664	727	715	699	2558	2621	2670	2708	1967	1805	2044	1925
K00845 glucokinase [EC:2.7.1.2]	4977	5043	5103	5062	7634	7716	7883	7923	7174	7422	7334	7363
K00850 6-phosphofructokinase 1 [EC:2.7.1.11]	2702	2797	2789	2795	3416	3618	3575	3393	3769	3840	3773	3818
K00873 pyruvate kinase [EC:2.7.1.40]	7147	7129	7132	6836	6764	6674	6742	6952	7200	6971	7122	6903
K00886 polyphosphate glucokinase [EC:2.7.1.63]	2	2	2	3	2	0	0	0	8	3	13	9
K00895 diphosphate-dependent phosphofructokinase [EC:2.7.1.90]	937	1130	1188	1108	3502	3491	3555	3299	2447	2397	2481	2285
K00918 ADP-dependent phosphofructokinase/glucokinase [EC:2.7.1.146 2.7.1.147]	8	4	1	2	13	16	3	9	31	33	26	20
K00927 phosphoglycerate kinase [EC:2.7.2.3]	4518	4471	4418	4463	4657	4921	4595	4509	4638	4537	4454	4548
K01085 glucose-1-phosphatase [EC:3.1.3.10]	761	684	690	750	471	485	500	421	969	1082	956	998
K01086 fructose-1,6-bisphosphatase I / sedoheptulose-1,7-bisphosphatase [EC:3.1.3.11 3.1.3.37]	1	1	0	1	2	0	0	0	0	0	2	1
K01222 6-phospho-beta-glucosidase [EC:3.2.1.86]	2574	2700	2428	2620	978	1072	1015	963	4405	4885	4587	4583
K01223 6-phospho-beta-glucosidase [EC:3.2.1.86]	8415	8540	8261	8631	5678	6061	5781	5438	9521	10202	9329	10037
K01596 phosphoenolpyruvate carboxykinase (GTP) [EC:4.1.1.32]	36	22	30	21	43	23	42	31	45	20	34	33
K01610 phosphoenolpyruvate carboxykinase (ATP) [EC:4.1.1.49]	6524	6393	6680	6622	6704	6697	6816	6676	7149	7323	7398	7118
K01622 fructose 1,6-bisphosphate aldolase/phosphatase [EC:4.1.2.13 3.1.3.11]	2	0	0	0	1	4	0	0	2	13	3	7
K01623 fructose-bisphosphate aldolase, class I [EC:4.1.2.13]	167	167	157	154	32	55	55	37	349	303	338	327
K01624 fructose-bisphosphate aldolase, class II [EC:4.1.2.13]	6833	6723	6594	6717	5602	5734	5627	5650	6507	7002	6741	6911
K01689 enolase [EC:4.2.1.11]	4355	4359	4446	4327	5171	5542	5258	5229	4613	4407	4462	4639
K01785 aldose 1-epimerase [EC:5.1.3.3]	3982	4387	4311	4359	8224	8264	8357	8134	7455	7509	7425	7157
K01792 glucose-6-phosphate 1-epimerase [EC:5.1.3.15]	2168	2177	2047	2169	1293	1220	1206	1401	1221	1232	1200	1285
K01803 triosephosphate isomerase (TIM) [EC:5.3.1.1]	3258	3528	3483	3510	3358	3562	3401	3364	3850	3893	3906	3869
K01810 glucose-6-phosphate isomerase [EC:5.3.1.9]	5895	5831	6010	5964	5767	5779	5575	5595	5661	5601	5546	5744
K01834 2,3-bisphosphoglycerate-dependent phosphoglycerate mutase [EC:5.4.2.11]	1383	1365	1451	1352	2251	2339	2237	2282	2061	2089	2085	1930
K01835 phosphoglucomutase [EC:5.4.2.2]	3966	3823	3976	4107	4928	5010	5112	4791	5614	5460	5373	5604
K01895 acetyl-CoA synthetase [EC:6.2.1.1]	7843	7868	7754	7877	7723	7669	7743	7792	6178	6178	6273	6256
K01905 acetate---CoA ligase (ADP-forming) subunit alpha [EC:6.2.1.13]	13	20	12	8	0	0	0	0	0	3	0	0
K02446 fructose-1,6-bisphosphatase II [EC:3.1.3.11]	726	766	654	713	479	511	581	482	1109	1037	1015	1030
K02752 PTS system, beta-glucoside (arbutin/salicin/cellobiose)-specific IIB component [EC:2.7.1.-]	926	959	888	953	611	676	593	607	1247	1241	1232	1296
K02777 PTS system, sugar-specific IIA component [EC:2.7.1.-]	594	593	591	628	237	312	232	257	595	690	650	643
K02778 PTS system, glucose-specific IIB component [EC:2.7.1.199]	135	148	151	171	98	119	110	112	192	195	170	180
K02779 PTS system, glucose-specific IIC component	901	892	919	939	549	632	604	624	1247	1329	1351	1264
K02790 PTS system, maltose/glucose-specific IIB component [EC:2.7.1.199 2.7.1.208]	1360	1290	1245	1307	1098	1058	1100	1159	1403	1413	1351	1547
K02791 PTS system, maltose/glucose-specific IIC component	0	0	0	0	0	1	0	0	0	0	0	0
K03737 pyruvate-ferredoxin/flavodoxin oxidoreductase [EC:1.2.7.1 1.2.7.-]	7665	7761	7814	8173	8670	9001	8957	8852	11378	12075	11511	11442
K03841 fructose-1,6-bisphosphatase I [EC:3.1.3.11]	2218	2283	2214	2256	1383	1537	1396	1412	1620	1579	1627	1562
K04022 alcohol dehydrogenase	497	448	424	491	151	116	141	148	1108	973	1036	1071
K04041 fructose-1,6-bisphosphatase III [EC:3.1.3.11]	2186	2438	2585	2428	3966	4037	4107	3867	3809	3871	3716	3695
K04072 acetaldehyde dehydrogenase / alcohol dehydrogenase [EC:1.2.1.10 1.1.1.1]	2747	2670	2860	2783	1272	1265	1220	1306	3039	3274	3244	3201
K06859 glucose-6-phosphate isomerase, archaeal [EC:5.3.1.9]	8	10	14	10	8	9	12	4	39	56	39	53
K10705 glyceraldehyde-3-phosphate dehydrogenase, spermatogenic [EC:1.2.1.12]	1	1	0	0	0	0	0	0	0	0	0	0
K11532 fructose-1,6-bisphosphatase II / sedoheptulose-1,7-bisphosphatase [EC:3.1.3.11 3.1.3.37]	0	0	0	1	3	0	1	0	0	6	5	1
K11645 fructose-bisphosphate aldolase, class I [EC:4.1.2.13]	1631	1712	1832	1694	2982	3093	3088	2932	3033	3160	3008	3063
K13953 alcohol dehydrogenase, propanol-preferring [EC:1.1.1.1]	2060	2096	1985	1950	996	973	1021	1096	1236	1286	1235	1182
K13954 alcohol dehydrogenase [EC:1.1.1.1]	1899	1895	2188	2184	1270	1379	1287	1201	753	881	795	743
K14085 aldehyde dehydrogenase family 7 member A1 [EC:1.2.1.31 1.2.1.8 1.2.1.3]	0	0	0	0	0	1	0	0	0	0	0	0
K15633 2,3-bisphosphoglycerate-independent phosphoglycerate mutase [EC:5.4.2.12]	5655	5458	5484	5488	6065	6093	5980	5951	5807	5664	5764	5463
K15634 probable phosphoglycerate mutase [EC:5.4.2.12]	581	573	548	581	486	515	441	490	569	634	616	651
K15635 2,3-bisphosphoglycerate-independent phosphoglycerate mutase [EC:5.4.2.12]	1007	1018	1124	1052	2695	2757	2840	2675	2410	2236	2357	2276
K15778 phosphomannomutase / phosphoglucomutase [EC:5.4.2.8 5.4.2.2]	4256	4121	4159	4126	2399	2347	2312	2521	1575	1409	1453	1439
K16306 fructose-bisphosphate aldolase / 2-amino-3,7-dideoxy-D-threo-hept-6-ulosonate synthase [EC:4.1.2.13 2.2.1.10]	2	0	0	1	0	0	3	0	7	6	7	12
K16370 6-phosphofructokinase 2 [EC:2.7.1.11]	570	526	436	508	372	357	384	374	714	786	704	752
K20118 PTS system, glucose-specific IIC component	399	375	347	366	460	538	469	488	279	298	251	310
K20866 glucose-1-phosphatase [EC:3.1.3.10]	783	829	759	788	1713	1782	1660	1720	1473	1526	1432	1526
K21071 ATP-dependent phosphofructokinase / diphosphate-dependent phosphofructokinase [EC:2.7.1.11 2.7.1.90]	2786	2764	2797	2845	2883	2973	2874	2780	3195	3405	3320	3209
K22224 acetate---CoA ligase (ADP-forming) subunit beta [EC:6.2.1.13]	369	386	329	350	146	141	158	174	821	861	722	882
ko00020 Citrate cycle (TCA cycle)	120604	120221	119660	120947	124566	126571	125497	125566	128898	130185	129169	129342
K00024 malate dehydrogenase [EC:1.1.1.37]	1872	2021	2084	2002	4954	4766	4938	4822	4052	3976	3945	3824
K00030 isocitrate dehydrogenase (NAD+) [EC:1.1.1.41]	441	430	420	544	171	197	152	213	1001	1063	1071	1111
K00031 isocitrate dehydrogenase [EC:1.1.1.42]	5104	4863	4952	5034	4691	4819	4747	4729	4219	4124	4277	4186
K00116 malate dehydrogenase (quinone) [EC:1.1.5.4]	3904	3931	3863	4028	2189	2100	2060	2043	3112	3383	3160	3334
K00164 2-oxoglutarate dehydrogenase E1 component [EC:1.2.4.2]	2459	2319	2297	2442	1596	1686	1614	1642	2667	2841	2690	2800
K00176 2-oxoglutarate ferredoxin oxidoreductase subunit delta [EC:1.2.7.3]	264	316	255	299	517	557	459	401	544	478	556	539
K00177 2-oxoglutarate ferredoxin oxidoreductase subunit gamma [EC:1.2.7.3]	705	614	742	715	1048	1068	1133	1059	1123	1156	1161	1127
K00235 succinate dehydrogenase (ubiquinone) iron-sulfur subunit [EC:1.3.5.1]	0	0	0	0	0	1	0	0	1	0	0	0
K00239 succinate dehydrogenase / fumarate reductase, flavoprotein subunit [EC:1.3.5.1 1.3.5.4]	5317	5288	5481	5592	6988	7192	7109	7166	5851	5810	6036	5806
K00240 succinate dehydrogenase / fumarate reductase, iron-sulfur subunit [EC:1.3.5.1 1.3.5.4]	1948	2036	2067	2049	2675	2789	2897	2818	2257	2280	2269	2155
K00241 succinate dehydrogenase / fumarate reductase, cytochrome b subunit	1286	1222	1323	1293	2195	2131	2232	2159	1616	1653	1732	1656
K00242 succinate dehydrogenase / fumarate reductase, membrane anchor subunit	748	751	689	714	439	476	448	477	516	487	423	450
K00244 fumarate reductase flavoprotein subunit [EC:1.3.5.4]	4725	4730	4595	4828	4230	4751	4466	4583	6176	6587	6494	6696
K00245 fumarate reductase iron-sulfur subunit [EC:1.3.5.4]	501	530	527	547	429	434	487	422	699	758	745	784
K00246 fumarate reductase subunit C	287	314	312	316	274	286	235	261	400	435	418	445
K00247 fumarate reductase subunit D	234	245	224	226	146	158	131	158	365	341	340	356
K00658 2-oxoglutarate dehydrogenase E2 component (dihydrolipoamide succinyltransferase) [EC:2.3.1.61]	2943	3053	3004	3038	3621	3672	4004	4135	3136	3004	3021	3045
K01647 citrate synthase [EC:2.3.3.1]	4779	4725	4806	4753	4915	5021	4772	4786	5561	5716	5546	5551
K01676 fumarate hydratase, class I [EC:4.2.1.2]	5087	4968	4925	5045	6133	6182	6121	5997	5773	5797	5742	5683
K01677 fumarate hydratase subunit alpha [EC:4.2.1.2]	709	653	674	627	207	247	265	196	796	938	859	813
K01678 fumarate hydratase subunit beta [EC:4.2.1.2]	344	363	355	326	82	105	101	93	490	491	488	471
K01679 fumarate hydratase, class II [EC:4.2.1.2]	5167	5090	4879	4879	3118	3214	2912	3048	2480	2401	2358	2434
K01681 aconitate hydratase [EC:4.2.1.3]	9263	9138	8926	9323	8552	8488	8559	8683	11053	11240	11064	11185
K01682 aconitate hydratase 2 / 2-methylisocitrate dehydratase [EC:4.2.1.3 4.2.1.99]	6721	6802	6535	6544	4264	4417	4153	4069	5429	5283	5132	5471
K01899 succinyl-CoA synthetase alpha subunit [EC:6.2.1.4 6.2.1.5]	0	0	0	0	0	0	0	0	2	0	0	0
K01900 succinyl-CoA synthetase beta subunit [EC:6.2.1.4 6.2.1.5]	0	0	0	1	0	2	0	0	0	1	0	0
K01902 succinyl-CoA synthetase alpha subunit [EC:6.2.1.5]	2076	2121	2090	2143	2536	2402	2647	2449	2211	2212	2202	2257
K01903 succinyl-CoA synthetase beta subunit [EC:6.2.1.5]	3039	3001	3200	3071	3574	3533	3402	3266	3089	2990	3077	2994
K01958 pyruvate carboxylase [EC:6.4.1.1]	1059	899	885	920	548	578	622	649	1191	1363	1166	1431
K01959 pyruvate carboxylase subunit A [EC:6.4.1.1]	1857	1848	1833	1852	1040	951	958	1083	624	636	567	627
K01960 pyruvate carboxylase subunit B [EC:6.4.1.1]	5119	5279	5293	5058	8746	9129	8849	8842	6079	6007	6104	5734
K05942 citrate (Re)-synthase [EC:2.3.3.3]	162	161	153	160	40	45	57	37	270	307	244	308
K18118 succinyl-CoA:acetate CoA-transferase [EC:2.8.3.18]	2111	2193	2082	2176	1204	1111	1070	1124	958	845	812	793
ko00030 Pentose phosphate pathway	112743	112570	111715	113078	99656	101420	100478	99284	111300	112739	110524	112052
K00032 phosphogluconate 2-dehydrogenase [EC:1.1.1.43]	1364	1367	1230	1302	742	709	659	718	385	295	329	377
K00033 6-phosphogluconate dehydrogenase [EC:1.1.1.44 1.1.1.343]	2399	2455	2533	2601	3729	3701	3782	3586	3841	4250	3990	4053

K00034 glucose 1-dehydrogenase [EC:1.1.1.47]	53	53	57	64	19	16	11	12	56	61	77	63
K00036 glucose-6-phosphate 1-dehydrogenase [EC:1.1.1.49 1.1.1.363]	6901	6838	6884	6843	6039	6066	6001	6174	5414	5131	5143	5096
K00090 glyoxylate/hydroxypyruvate/2-ketogluconate reductase [EC:1.1.1.79 1.1.1.81 1.1.1.215]	1733	1799	1552	1736	945	1062	973	1020	1230	1236	1198	1246
K00117 quinoprotein glucose dehydrogenase [EC:1.1.5.2]	5019	4864	4718	4740	3115	3347	3146	3189	3047	2972	2886	3127
K00615 transketolase [EC:2.2.1.1]	13517	13414	13461	13442	9604	9691	9778	9303	14442	14290	14105	14499
K00616 transaldolase [EC:2.2.1.2]	2686	2810	2779	2906	2831	2905	3012	2917	3855	3909	3806	3842
K00851 gluconokinase [EC:2.7.1.12]	1170	1269	1332	1296	801	740	808	797	965	957	924	948
K00852 ribokinase [EC:2.7.1.15]	4636	4221	4473	4477	3765	3703	3574	3590	4678	4839	4681	4857
K00874 2-dehydro-3-deoxygluconokinase [EC:2.7.1.45]	3277	3126	3415	3411	3608	3636	3623	3626	4248	4381	4480	4464
K00948 ribose-phosphate pyrophosphokinase [EC:2.7.6.1]	4083	4114	4036	4141	3554	3612	3629	3693	3819	3769	3811	3656
K01053 gluconolactonase [EC:3.1.1.17]	465	424	429	401	251	335	295	320	137	141	138	131
K01057 6-phosphogluconolactonase [EC:3.1.1.31]	1528	1496	1478	1452	1993	1981	1980	2071	1380	1230	1305	1215
K01619 deoxyribose-phosphate aldolase [EC:4.1.2.4]	2692	2736	2571	2624	2905	2980	3027	2905	3748	3807	3818	3705
K01621 xylulose-5-phosphate/fructose-6-phosphate phosphoketolase [EC:4.1.2.9 4.1.2.22]	154	128	146	120	267	277	259	236	34	79	72	63
K01625 2-dehydro-3-deoxyphosphogluconate aldolase / (4S)-4-hydroxy-2-oxoglutarate aldolase [EC:4.1.2.14 4.1.3.42]	3200	3039	2905	2986	2555	2647	2684	2680	2467	2661	2564	2611
K01690 phosphogluconate dehydratase [EC:4.2.1.12]	1462	1511	1427	1471	908	931	985	990	1487	1581	1470	1530
K01783 ribulose-phosphate 3-epimerase [EC:5.1.3.1]	2481	2589	2754	2803	2789	2882	2859	2620	2624	2620	2596	2576
K01807 ribose 5-phosphate isomerase A [EC:5.3.1.6]	1841	1888	1900	1896	2265	2129	2181	2110	2164	2173	2267	2216
K01808 ribose 5-phosphate isomerase B [EC:5.3.1.6]	1050	1039	1018	987	1233	1295	1262	1241	1392	1475	1373	1455
K01839 phosphopentomutase [EC:5.4.2.7]	1312	1341	1452	1472	666	636	671	617	1827	2075	1955	2113
K03738 aldehyde:ferredoxin oxidoreductase [EC:1.2.7.5]	212	255	244	232	154	150	151	119	574	691	564	608
K05308 gluconate/galactonate dehydratase [EC:4.2.1.140]	14	12	23	17	4	5	3	4	33	25	30	28
K05774 ribose 1,5-bisphosphokinase [EC:2.7.4.23]	1071	1081	986	960	533	515	468	569	685	740	723	796
K06151 gluconate 2-dehydrogenase alpha chain [EC:1.1.99.3]	2832	2866	2492	2813	1274	1403	1317	1487	1621	1595	1532	1532
K06152 gluconate 2-dehydrogenase gamma chain [EC:1.1.99.3]	1222	1216	1129	1190	596	559	491	570	890	923	859	917
K07404 6-phosphogluconolactonase [EC:3.1.1.31]	1754	1878	1811	1850	624	756	633	563	1879	2100	1948	1970
K08093 3-hexulose-6-phosphate synthase [EC:4.1.2.43]	3	4	3	2	2	1	0	2	13	16	13	12
K08094 6-phospho-3-hexuloisomerase [EC:5.3.1.27]	29	35	19	25	11	5	9	1	70	64	70	68
K11441 dehydrogluconokinase [EC:2.7.1.13]	1184	1196	1076	1135	532	575	550	631	344	304	298	361
K11529 glycerate 2-kinase [EC:2.7.1.165]	2766	2585	2489	2539	1699	1718	1667	1694	908	854	858	882
K13812 bifunctional enzyme Fae/Hps [EC:4.2.1.147 4.1.2.43]	2	0	0	0	0	0	2	2	6	4	2	8
K13831 3-hexulose-6-phosphate synthase / 6-phospho-3-hexuloisomerase [EC:4.1.2.43 5.3.1.27]	1	1	1	0	0	0	1	0	9	1	3	3
K17463 2-dehydro-3-deoxy-phosphogluconate aldolase [EC:4.1.2.14]	0	0	2	0	0	0	3	0	0	0	2	7
K17464 PTS system, D-glucosaminate-specific IIA component [EC:2.7.1.203]	28	32	26	21	8	12	4	2	45	74	49	51
K17465 PTS system, D-glucosaminate-specific IIB component [EC:2.7.1.203]	20	31	30	34	1	14	9	14	64	77	70	68
K17466 PTS system, D-glucosaminate-specific IIC component	76	63	53	59	18	12	5	18	110	84	88	90
K17467 PTS system, D-glucosaminate-specific IID component	56	71	67	64	8	10	9	16	98	106	112	111
K17468 D-glucosaminate-6-phosphate ammonia-lyase [EC:4.3.1.29]	0	0	2	0	2	0	2	0	1	0	1	2
K18022 glyceraldehyde dehydrogenase small subunit [EC:1.2.99.8]	0	0	0	0	1	0	0	0	0	0	0	0
K19813 glucose dehydrogenase [EC:1.1.5.9]	2	2	0	1	0	0	0	0	0	0	0	0
K22229 2-ketogluconate reductase [EC:1.1.1.215]	4	2	3	3	3	1	4	3	0	0	0	0
K22345 glucosaminate ammonia-lyase [EC:4.3.1.9]	1426	1463	1385	1436	801	818	771	872	571	463	437	450
ko00040 Pentose and glucuronate interconversions	67575	67509	68186	69040	87252	88003	88499	86556	94585	95082	95426	95212
K00007 D-arabinitol 4-dehydrogenase [EC:1.1.1.11]	720	748	707	732	525	557	506	561	863	963	910	905
K00008 L-iditol 2-dehydrogenase [EC:1.1.1.14]	1003	1060	1121	1055	1601	1652	1652	1568	2118	2078	2171	2130
K00011 aldehyde reductase [EC:1.1.1.21]	0	1	1	1	2	0	0	0	1	0	0	1
K00012 UDPglucose 6-dehydrogenase [EC:1.1.1.22]	4860	4927	4712	4734	4380	4256	4495	4275	4567	4348	4457	4574
K00039 ribitol 2-dehydrogenase [EC:1.1.1.56]	299	269	291	290	90	85	81	68	626	583	502	623
K00040 fructuronate reductase [EC:1.1.1.57]	2486	2472	2378	2464	1347	1297	1290	1280	3215	3389	3118	3429
K00041 tagaturonate reductase [EC:1.1.1.58]	2083	2071	2201	2086	3393	3504	3370	3429	3525	3390	3570	3383
K00065 2-dehydro-3-deoxy-D-gluconate 5-dehydrogenase [EC:1.1.1.127]	1115	1035	1014	1182	628	690	676	657	1474	1531	1418	1577
K00078 dihydrodiol dehydrogenase / D-xylose 1-dehydrogenase (NADP) [EC:1.3.1.20 1.1.1.179]	3	5	2	3	0	0	0	0	6	4	8	7
K00848 rhamnulokinase [EC:2.7.1.5]	3459	3558	3642	3705	6682	6534	6926	6491	6511	6449	6858	6456
K00853 L-ribulokinase [EC:2.7.1.16]	1299	1247	1142	1256	837	875	798	826	1743	1891	1807	1757
K00854 xylulokinase [EC:2.7.1.17]	4970	4712	4793	4953	5452	5612	5553	5484	6650	6832	6708	6951
K00875 D-ribulokinase [EC:2.7.1.47]	580	677	551	582	190	194	150	159	1142	1331	1145	1285
K00880 L-xylulokinase [EC:2.7.1.53]	823	804	818	881	776	776	759	779	1397	1476	1339	1455
K00963 UTP--glucose-1-phosphate uridylyltransferase [EC:2.7.7.9]	1849	1761	1751	1800	1380	1480	1322	1405	1673	1922	1817	1890
K01051 pectinesterase [EC:3.1.1.11]	3926	4011	4473	4185	11896	11951	11827	11802	9605	8792	9556	9076
K01184 polygalacturonase [EC:3.2.1.15]	289	304	279	275	409	449	442	414	0	0	0	1
K01195 beta-glucuronidase [EC:3.2.1.31]	2965	2979	3153	3092	7622	7568	7800	7477	7097	6746	7115	6902
K01629 rhamnulose-1-phosphate aldolase [EC:4.1.2.19]	1607	1667	1649	1640	2389	2398	2490	2204	2563	2676	2734	2633
K01685 altronate hydrolase [EC:4.2.1.7]	2431	2566	2555	2492	3502	3676	3728	3688	3753	3776	3898	4004
K01686 mannonate dehydratase [EC:4.2.1.8]	3849	3979	4004	3948	4450	4710	4738	4386	5266	5541	5429	5326
K01728 pectate lyase [EC:4.2.2.2]	3	0	0	1	0	0	5	0	2	0	2	4
K01730 oligogalacturonide lyase [EC:4.2.2.6]	631	689	615	699	365	326	336	314	1045	1122	1034	1116
K01804 L-arabinose isomerase [EC:5.3.1.4]	2549	2511	2697	2657	3997	3737	4147	3917	4192	4436	4335	4125
K01805 xylose isomerase [EC:5.3.1.5]	2294	2252	2320	2376	3355	3399	3445	3341	3967	4059	4083	4057
K01812 glucuronate isomerase [EC:5.3.1.12]	2614	2561	2772	2718	3536	3561	3659	3543	3609	3502	3594	3642
K01815 4-deoxy-L-threo-5-hexosulose-uronate ketol-isomerase [EC:5.3.1.17]	1960	1995	2170	2032	4663	4455	4441	4525	3514	3740	3703	3442
K01820 L-rhamnose isomerase / sugar isomerase [EC:5.3.1.14 5.3.1.-]	0	2	2	2	3	2	0	0	0	5	3	1
K03077 L-ribulose-5-phosphate 4-epimerase [EC:5.1.3.4]	2082	2170	2151	2159	2252	2448	2505	2392	2848	2789	2768	2669
K03078 3-dehydro-L-gulonate-6-phosphate decarboxylase [EC:4.1.1.85]	667	600	631	634	551	568	551	578	697	633	632	718
K03079 L-ribulose-5-phosphate 3-epimerase [EC:5.1.3.22]	1003	944	889	996	678	845	773	719	973	1027	1059	1008
K03331 L-xylulose reductase [EC:1.1.1.10]	0	0	0	1	0	0	0	0	0	0	0	1
K05351 D-xylulose reductase [EC:1.1.1.9]	4	1	2	5	6	1	3	3	2	10	5	6
K05352 ribitol-5-phosphate 2-dehydrogenase (NADP+) [EC:1.1.1.405]	1	5	0	2	0	1	2	7	4	11	6	9
K08092 3-dehydro-L-gulonate 2-dehydrogenase [EC:1.1.1.130]	292	283	295	327	451	465	435	407	114	162	122	132
K08322 L-gulonate 5-dehydrogenase [EC:1.1.1.380]	691	720	614	708	379	374	414	420	1084	1083	1004	1101
K08323 mannonate dehydratase [EC:4.2.1.8]	762	763	769	788	754	820	784	726	675	660	663	673
K09988 D-lyxose ketol-isomerase [EC:5.3.1.15]	514	517	477	597	469	522	523	499	618	720	608	718
K13877 2,5-dioxopentanoate dehydrogenase [EC:1.2.1.26]	4060	3708	3521	3761	2044	1876	1757	2170	1255	1019	1003	1098
K14274 xylonolactonase [EC:3.1.1.-]	565	558	598	604	372	363	342	419	739	781	786	817
K16849 altronate dehydratase small subunit [EC:4.2.1.7]	73	66	68	71	35	43	55	38	119	114	106	115
K16850 altronate dehydratase large subunit [EC:4.2.1.7]	649	677	635	681	321	321	308	286	985	1029	909	1014
K17743 D-xylose reductase [EC:1.1.1.307]	0	0	1	1	3	1	3	0	0	1	2	0
K17818 D-arabinitol dehydrogenase (NADP+) [EC:1.1.1.287]	2	1	2	3	2	2	3	0	4	2	3	5
K18106 D-galacturonate reductase [EC:1.1.1.-]	0	0	0	0	0	0	0	0	0	0	0	1
K19588 2,5-dioxopentanoate dehydrogenase [EC:1.2.1.26]	1	2	1	1	7	3	2	3	4	0	2	1
K21030 D-ribitol-5-phosphate cytidylyltransferase [EC:2.7.7.40]	4	2	2	1	0	0	1	0	1	12	3	18
K21031 D-ribitol-5-phosphate cytidylyltransferase [EC:2.7.7.40]	0	0	0	0	0	0	0	0	0	0	0	1
K21619 tagaturonate epimerase [EC:5.1.2.7]	39	40	38	26	2	0	0	0	97	85	76	82
K21681 ribitol-5-phosphate 2-dehydrogenase (NADP+) / D-ribitol-5-phosphate cytidylyltransferase [EC:1.1.1.405 2.7.7.40]	152	160	196	144	246	292	236	272	24	22	24	36
K22185 D-xylose 1-dehydrogenase [EC:1.1.1.175]	153	173	166	163	230	227	210	213	0	3	0	0
K22186 xylonate dehydratase [EC:4.2.1.82]	3	0	1	0	2	0	0	1	0	2	0	1
K22187 alpha-ketoglutaric semialdehyde dehydrogenase [EC:1.2.1.-]	3	0	3	1	5	0	2	2	2	4	2	7
K22396 xylonate dehydratase [EC:4.2.1.82]	873	919	772	881	1390	1450	1302	1371	2	6	11	9
K22397 2-dehydro-3-deoxy-D-pentonate aldolase [EC:4.1.2.28]	785	768	823	849	636	630	628	668	404	415	402	381
K22933 galacturan 1,4-alpha-galacturonidase [EC:3.2.1.67]	809	758	769	788	89	65	81	62	679	723	751	712
K22935 D-ribulokinase [EC:2.7.1.47]	55	48	35	47	5	1	0	3	85	118	111	98
K22994 pectate disaccharide-lyase [EC:4.2.2.9]	11	5	11	9	21	22	32	19	116	141	122	118
ko00051 Fructose and mannose metabolism	99507	100904	100066	100728	112467	113728	113153	110641	116005	117468	116532	116869
K00009 mannitol-1-phosphate 5-dehydrogenase [EC:1.1.1.17]	798	742	791	706	575	597	613	514	990	1087	996	1123
K00045 mannitol 2-dehydrogenase [EC:1.1.1.67]	2522	2627	2248	2329	3277	3234	3157	3215	2298	2077	2273	2148
K00064 D-threo-aldose 1-dehydrogenase [EC:1.1.1.122]	0	0	0	1	0	0	3	2	3	8	6	4
K00066 GDP-mannose 6-dehydrogenase [EC:1.1.1.132]	1877	1974	1877	1895	1191	1266	1246	1235	677	665	558	615
K00068 sorbitol-6-phosphate 2-dehydrogenase [EC:1.1.1.140]	736	823	820	770	547	688	608	582	753	750	794	795
K00847 fructokinase [EC:2.7.1.4]	5390	5227	5576	5413	7448	7276	7231	7228	6704	6628	7026	6951
K00863 triose/dihydroxyacetone kinase / FAD-AMP lyase (cyclizing) [EC:2.7.1.28 2.7.1.29 4.6.1.15]	1025	1087	1046	1046	693	734	712	666	1413	1313	1298	1436
K00879 L-fuculokinase [EC:2.7.1.51]	606	653	525	619	168	241	195	179	1253	1237	1196	1307
K00881 allose kinase [EC:2.7.1.55]	60	40	43	39	9	8	4	10	139	140	180	182
K00882 1-phosphofructokinase [EC:2.7.1.56]	1167	1077	1030	1092	545	532	565	511	1224	1285	1223	1436
K00900 6-phosphofructo-2-kinase [EC:2.7.1.105]	0	0	0	0	0	0	0	0	0	0	0	1
K00966 mannose-1-phosphate guanylyltransferase [EC:2.7.7.13]	14	19	16	21	0	0	0	0	24	38	28	42
K00971 mannose-1-phosphate guanylyltransferase [EC:2.7.7.13]	2438	2518	2612	2591	5333	5223	5491	5253	4634	4505	4610	4544
K01218 mannan endo-1,4-beta-mannosidase [EC:3.2.1.78]	489	505	496	509	185	210	185	232	326	345	360	355
K01628 L-fuculose-phosphate aldolase [EC:4.1.2.17]	2122	2068	2136	2074	1043	1137	1077	1150	1606	1665	1552	1667
K01711 GDPmannose 4,6-dehydratase [EC:4.2.1.47]	2114	2221	2320	2252	4540	4418	4456	4333	3096	3220	3169	3159
K01729 poly(beta-D-mannuronate) lyase [EC:4.2.2.3]	515	510	490	467	414	430	416	453	107	79	87	91
K01795 mannuronan 5-epimerase [EC:5.1.3.37]	1317	1300	1245	1245	660	631	605	663	406	376	352	417
K01809 mannose-6-phosphate isomerase [EC:5.3.1.8]	1973	1891	1983	1891	2930	3000	2881	2686	2662	2548	2657	2641

K01813 L-rhamnose isomerase [EC:5.3.1.14]	2188	2321	2379	2329	3516	3678	3678	3581	3820	3984	3793	3794
K01818 L-fucose/D-arabinose isomerase [EC:5.3.1.25 5.3.1.3]	2097	2196	2258	2223	4023	3915	3965	3908	4625	4663	4665	4529
K01840 phosphomannomutase [EC:5.4.2.8]	1620	1746	1779	1789	3556	3505	3505	3544	3582	3481	3538	3321
K02377 GDP-L-fucose synthase [EC:1.1.1.271]	1022	1100	1029	1149	2584	2716	2653	2603	1655	1680	1508	1550
K02768 PTS system, fructose-specific IIA component [EC:2.7.1.202]	418	440	417	468	278	270	224	233	477	528	475	496
K02769 PTS system, fructose-specific IIB component [EC:2.7.1.202]	206	199	182	187	131	167	151	171	266	267	288	321
K02770 PTS system, fructose-specific IIC component	3676	3765	3503	3644	2009	1940	1789	1935	3411	3654	3318	3616
K02771 PTS system, fructose-specific IID component	0	0	0	0	0	0	0	0	0	0	0	1
K02781 PTS system, glucitol/sorbitol-specific IIA component [EC:2.7.1.198]	475	445	456	580	375	418	327	330	658	651	630	620
K02783 PTS system, glucitol/sorbitol-specific IIC component	956	938	905	941	736	721	712	640	1332	1359	1278	1367
K02793 PTS system, mannose-specific IIA component [EC:2.7.1.191]	188	157	147	152	31	26	61	17	105	127	138	114
K02794 PTS system, mannose-specific IIB component [EC:2.7.1.191]	967	997	871	958	417	470	400	418	1011	1114	1051	1071
K02795 PTS system, mannose-specific IIC component	1385	1343	1357	1420	488	505	471	487	1183	1299	1239	1259
K02796 PTS system, mannose-specific IID component	1417	1411	1444	1429	532	516	610	507	1261	1471	1362	1372
K02798 PTS system, mannitol-specific IIA component [EC:2.7.1.197]	65	59	51	64	24	23	27	16	102	111	93	90
K02800 PTS system, mannitol-specific IIC component	1552	1523	1422	1553	991	1062	993	1032	1889	2113	1955	2045
K02812 PTS system, sorbose-specific IIA component [EC:2.7.1.206]	87	79	93	74	66	70	73	58	25	36	31	26
K02813 PTS system, sorbose-specific IIB component [EC:2.7.1.206]	149	180	195	182	159	157	156	171	33	43	28	44
K02814 PTS system, sorbose-specific IIC component	190	195	245	203	251	265	268	269	45	67	62	67
K02815 PTS system, sorbose-specific IID component	267	287	269	286	292	296	294	292	45	66	45	54
K03332 fructan beta-fructosidase [EC:3.2.1.80]	1635	1913	1849	1781	5225	5214	5283	4912	4049	3933	4033	3799
K05305 fucokinase [EC:2.7.1.52]	104	100	121	106	37	69	92	49	204	203	182	229
K07026 mannosyl-3-phosphoglycerate phosphatase [EC:3.1.3.70]	160	158	163	173	178	299	257	251	26	46	19	34
K07046 L-fuconolactonase [EC:3.1.1.-]	9	2	4	7	0	2	0	1	0	0	2	0
K08261 D-sorbitol dehydrogenase (acceptor) [EC:1.1.99.21]	1	0	1	0	3	0	0	0	0	0	0	0
K12660 2-dehydro-3-deoxy-L-rhamnonate aldolase [EC:4.1.2.53]	530	518	454	518	335	313	322	342	638	605	622	668
K12661 L-rhamnonate dehydratase [EC:4.2.1.90]	699	741	709	746	482	581	485	465	1029	1064	1002	1100
K15856 GDP-4-dehydro-6-deoxy-D-mannose reductase [EC:1.1.1.281]	1859	1744	1660	1739	975	916	867	891	626	524	603	669
K16011 mannose-1-phosphate guanylyltransferase / mannose-6-phosphate isomerase [EC:2.7.7.13 5.3.1.8]	6543	6460	6085	6326	3518	3457	3448	3537	2144	2039	1943	1989
K16881 mannose-1-phosphate guanylyltransferase / phosphomannomutase [EC:2.7.7.13 5.4.2.8]	11	18	11	9	2	0	2	0	10	2	3	4
K17195 D-allulose-6-phosphate 3-epimerase [EC:5.1.3.-]	338	340	326	366	76	78	105	70	600	638	562	641
K17742 sorbose reductase [EC:1.1.1.289]	1	0	0	0	0	0	0	0	1	0	0	0
K18333 L-fucose dehydrogenase	20	9	11	13	3	2	0	4	13	11	5	7
K18334 L-fuconate dehydratase [EC:4.2.1.68]	3	2	3	2	1	0	7	6	3	10	15	4
K18335 2-keto-3-deoxy-L-fuconate dehydrogenase [EC:1.1.1.-]	3	5	0	1	0	5	3	0	2	1	1	3
K18337 L-rhamnose 1-dehydrogenase [EC:1.1.1.378 1.1.1.377 1.1.1.173]	2	7	1	1	0	0	0	0	0	1	0	0
K19290 mannuronan synthase [EC:2.4.1.33]	2230	2184	2287	2302	1302	1216	1162	1247	729	695	708	761
K19291 mannuronan synthase [EC:2.4.1.33]	1684	1554	1525	1517	929	827	777	888	507	556	458	511
K19355 mannan endo-1,4-beta-mannosidase [EC:3.2.1.78]	80	69	69	53	200	180	188	194	13	37	45	37
K19956 L-sorbose 1-phosphate reductase [EC:1.1.1.-]	434	405	465	418	456	447	443	435	202	202	190	197
K21883 2-dehydro-3-deoxy-L-rhamnonate dehydrogenase (NAD+) [EC:1.1.1.401]	1	2	3	5	0	0	0	0	1	4	2	1
ko00052 Galactose metabolism	98623	101417	103752	102272	155223	156456	157136	153939	154648	155519	155464	154988
K00035 D-galactose 1-dehydrogenase [EC:1.1.1.48]	1	0	0	0	1	2	0	2	2	0	0	2
K00094 galactitol-1-phosphate 5-dehydrogenase [EC:1.1.1.251]	5	3	3	2	10	13	10	6	47	55	38	43
K00849 galactokinase [EC:2.7.1.6]	2393	2603	2469	2460	3289	3311	3448	3314	3443	3599	3314	3607
K00883 2-dehydro-3-deoxygalactonokinase [EC:2.7.1.58]	995	924	945	984	527	566	582	540	1590	1511	1424	1533
K00917 tagatose 6-phosphate kinase [EC:2.7.1.144]	119	130	154	136	60	57	45	53	212	271	218	255
K00965 UDPglucose--hexose-1-phosphate uridylyltransferase [EC:2.7.7.12]	1321	1413	1373	1488	1030	1083	983	977	1529	1441	1494	1511
K01182 oligo-1,6-glucosidase [EC:3.2.1.10]	1373	1325	1385	1366	323	374	351	332	1526	1619	1547	1621
K01187 alpha-glucosidase [EC:3.2.1.20]	9858	10342	11056	10380	22900	23140	23139	22711	19666	19818	20303	19484
K01190 beta-galactosidase [EC:3.2.1.23]	22473	23862	24933	23509	54266	54176	54438	53231	40247	38418	39959	38794
K01193 beta-fructofuranosidase [EC:3.2.1.26]	4207	4334	4229	4392	2703	2867	2703	2603	5125	5236	5272	5441
K01220 6-phospho-beta-galactosidase [EC:3.2.1.85]	82	76	79	98	30	66	34	56	127	151	148	138
K01229 lactase-phlorizin hydrolase [EC:3.2.1.108 3.2.1.62]	1	0	0	0	0	0	0	0	0	0	0	1
K01631 2-dehydro-3-deoxyphosphogalactonate aldolase [EC:4.1.2.21]	593	623	600	749	336	346	340	374	1104	1112	1031	1021
K01635 tagatose 1,6-diphosphate aldolase [EC:4.1.2.40]	154	158	174	170	46	77	55	66	176	239	218	219
K01684 galactonate dehydratase [EC:4.2.1.6]	1720	1614	1721	1715	567	596	626	642	1664	1806	1670	1831
K01784 UDP-glucose 4-epimerase [EC:5.1.3.2]	4262	4362	4301	4297	4811	4755	4699	4702	5126	5274	5242	5493
K01819 galactose-6-phosphate isomerase [EC:5.3.1.26]	108	107	87	109	35	54	50	42	135	165	143	119
K01854 UDP-galactopyranose mutase [EC:5.4.99.9]	1271	1340	1336	1376	1278	1176	1317	1308	2606	2722	2528	2666
K02079 N-acetylgalactosamine-6-phosphate deacetylase [EC:3.5.1.25]	13	2	2	5	14	19	18	11	67	91	55	62
K02080 galactosamine-6-phosphate isomerase [EC:5.3.1.-]	185	143	150	173	256	282	252	227	72	67	71	58
K02744 PTS system, N-acetylgalactosamine-specific IIA component [EC:2.7.1.-]	326	308	394	383	197	217	199	206	167	205	184	219
K02745 PTS system, N-acetylgalactosamine-specific IIB component [EC:2.7.1.-]	41	55	48	61	13	15	16	9	90	53	78	60
K02746 PTS system, N-acetylgalactosamine-specific IIC component	83	91	90	84	34	34	24	29	135	167	136	108
K02747 PTS system, N-acetylgalactosamine-specific IID component	121	105	119	152	21	35	27	36	115	206	155	189
K02773 PTS system, galactitol-specific IIA component [EC:2.7.1.200]	67	68	86	57	27	30	29	26	95	132	122	148
K02774 PTS system, galactitol-specific IIB component [EC:2.7.1.200]	69	46	40	49	24	16	11	7	101	120	77	95
K02775 PTS system, galactitol-specific IIC component	1338	1472	1557	1487	3198	3233	3307	3170	3232	3324	3385	3450
K02786 PTS system, lactose-specific IIA component [EC:2.7.1.207]	24	27	23	21	8	20	6	18	39	48	25	28
K02788 PTS system, lactose-specific IIC component	119	104	138	96	73	79	69	45	138	179	169	139
K06617 raffinose synthase [EC:2.4.1.82]	0	0	1	0	0	2	0	0	0	0	0	0
K07406 alpha-galactosidase [EC:3.2.1.22]	2047	2029	1906	1946	490	530	489	431	3502	3637	3365	3697
K07407 alpha-galactosidase [EC:3.2.1.22]	8234	8579	9005	8696	14632	14741	14912	14439	15668	15810	15673	15470
K08302 tagatose 1,6-diphosphate aldolase GatY/KbaY [EC:4.1.2.40]	213	228	215	240	341	377	342	304	193	197	225	209
K10984 PTS system, galactosamine-specific IIB component [EC:2.7.1.-]	108	102	95	116	219	204	153	141	18	21	15	14
K10985 PTS system, galactosamine-specific IIC component	149	164	159	188	261	273	260	258	30	39	46	42
K10986 PTS system, galactosamine-specific IID component	210	160	162	192	269	301	268	279	64	57	49	47
K12111 evolved beta-galactosidase subunit alpha [EC:3.2.1.23]	582	521	562	576	131	102	102	102	772	834	781	836
K12112 evolved beta-galactosidase subunit beta	3	2	0	0	2	8	2	6	30	32	19	30
K12308 beta-galactosidase [EC:3.2.1.23]	7522	7550	7621	7746	8363	8310	8731	8624	12368	12998	12754	12824
K12309 beta-galactosidase [EC:3.2.1.23]	4	5	1	4	9	5	7	9	4	10	4	5
K16371 D-tagatose-1,6-bisphosphate aldolase subunit GatZ/KbaZ	272	291	266	251	551	519	524	488	133	147	150	157
K17716 UDP-glucose 4-epimerase [EC:5.1.3.2]	189	185	206	196	74	50	73	75	109	115	98	122
K21620 galactitol 2-dehydrogenase [EC:1.1.1.16]	2	0	0	2	0	0	0	5	2	2	2	1
K21621 tagatose kinase [EC:2.7.1.101]	0	2	0	0	4	2	3	2	2	0	2	7
K21622 D-tagatose 6-phosphate 4-epimerase [EC:5.1.3.40]	0	0	0	1	0	2	0	0	7	8	2	6
ko00053 Ascorbate and aldarate metabolism	31565	31134	30516	31214	27069	27246	27067	26966	31810	31724	31617	31764
K01630 2-dehydro-3-deoxyglucarate aldolase [EC:4.1.2.20]	736	719	667	770	367	336	352	361	824	912	887	885
K01706 glucarate dehydratase [EC:4.2.1.40]	1226	1190	1186	1252	1009	1030	1081	1072	1205	1300	1197	1233
K01708 galactarate dehydratase [EC:4.2.1.42]	874	873	852	928	708	701	656	681	1135	1122	1122	1141
K02821 PTS system, ascorbate-specific IIA component [EC:2.7.1.194]	1082	1013	1010	1057	648	578	543	500	1559	1706	1630	1771
K02822 PTS system, ascorbate-specific IIB component [EC:2.7.1.194]	841	776	849	845	373	353	375	424	1206	1330	1279	1242
K03475 PTS system, ascorbate-specific IIC component	3851	3813	3771	4014	2213	2255	2258	2055	5352	5462	5478	5488
K03476 L-ascorbate 6-phosphate lactonase [EC:3.1.1.-]	1146	1098	1060	1093	576	686	626	581	1334	1401	1441	1423
K13874 L-arabinonolactonase [EC:3.1.1.15]	273	364	297	312	80	79	61	64	648	720	642	652
K13875 L-arabonate dehydrase [EC:4.2.1.25]	18	20	16	14	3	1	2	3	39	30	26	27
K13876 2-keto-3-deoxy-L-arabinonate dehydratase [EC:4.2.1.43]	2	0	1	1	0	0	0	0	1	0	0	0
K18981 uronate dehydrogenase [EC:1.1.1.203]	0	2	0	0	0	0	0	2	2	2	0	1
K18983 D-galactarolactone cycloisomerase [EC:5.5.1.27]	43	54	40	52	4	4	8	6	112	95	96	131
K20023 L-talarate/galactarate dehydratase [EC:4.2.1.156 4.2.1.42]	267	261	234	257	337	353	321	300	69	77	83	84
K23003 L-galactose dehydrogenase [EC:1.1.1.-]	525	626	650	539	1906	1942	2073	2001	1449	1418	1577	1337
K23004 L-galactono-1,5-lactonase [EC:3.1.1.-]	579	635	763	574	2148	2193	2113	2164	1469	1420	1558	1325
K23005 L-galactonate 5-dehydrogenase [EC:1.1.1.414]	520	524	630	584	1904	1934	1901	1779	1429	1309	1307	1326
K23007 L-galactonate 5-dehydrogenase [EC:1.1.1.414]	226	219	236	209	297	361	337	311	66	63	82	83
ko00500 Starch and sucrose metabolism	170892	171950	172710	172783	219449	219930	220950	217325	233873	236153	235102	235064
K00688 glycogen phosphorylase [EC:2.4.1.1]	6185	6421	6444	6237	8504	8623	8431	8153	8608	8888	8959	8717
K00690 sucrose phosphorylase [EC:2.4.1.7]	318	290	349	318	70	58	62	42	474	518	449	504
K00691 maltose phosphorylase [EC:2.4.1.8]	177	178	171	213	51	47	50	34	128	225	142	214
K00693 glycogen synthase [EC:2.4.1.11]	0	0	0	0	2	0	0	0	0	0	0	0
K00694 cellulose synthase (UDP-forming) [EC:2.4.1.12]	3540	3546	3353	3629	2892	3025	2801	2835	4078	4291	3986	4262
K00695 sucrose synthase [EC:2.4.1.13]	0	0	0	0	0	0	0	0	0	0	2	0
K00697 trehalose 6-phosphate synthase [EC:2.4.1.15 2.4.1.347]	845	780	787	782	480	596	596	623	1152	1207	1084	1102
K00700 1,4-alpha-glucan branching enzyme [EC:2.4.1.18]	6462	6110	6258	6152	6080	6244	6255	6228	6869	7003	6728	6764
K00702 cellobiose phosphorylase [EC:2.4.1.20]	156	127	174	122	116	95	91	80	260	265	275	255
K00703 starch synthase [EC:2.4.1.21]	4177	3965	3914	4062	3609	3683	3581	3668	3659	3757	3570	3649
K00705 4-alpha-glucanotransferase [EC:2.4.1.25]	6389	6350	6405	6342	8163	7974	8232	8221	7412	7256	7207	7418
K00975 glucose-1-phosphate adenylyltransferase [EC:2.7.7.27]	2543	2459	2519	2552	1142	1250	1124	1157	2789	2953	2792	2914
K00978 glucose-1-phosphate cytidylyltransferase [EC:2.7.7.33]	781	832	884	837	1424	1425	1572	1430	1212	1160	1222	1103
K01087 trehalose 6-phosphate phosphatase [EC:3.1.3.12]	442	436	403	429	315	293	257	286	538	525	506	589
K01176 alpha-amylase [EC:3.2.1.1]	2391	2467	2547	2628	2064	2245	2070	2042	3137	3223	3102	3241

K01178 glucoamylase [EC:3.2.1.3]	0	0	1	0	0	0	0	0	0	0	0	0
K01179 endoglucanase [EC:3.2.1.4]	1698	2008	2107	1985	6744	6610	6838	6589	5575	5269	5430	5180
K01188 beta-glucosidase [EC:3.2.1.21]	31	48	33	35	9	3	3	4	26	29	19	28
K01194 alpha,alpha-trehalase [EC:3.2.1.28]	3907	4151	3820	3903	2458	2568	2478	2616	3352	3528	3326	3468
K01200 pullulanase [EC:3.2.1.41]	2006	2118	2078	2080	2306	2459	2337	2359	3962	4010	3907	3955
K01208 cyclomaltodextrinase / maltogenic alpha-amylase / neopullulanase [EC:3.2.1.54 3.2.1.133 3.2.1.135]	361	346	396	350	71	92	70	77	554	635	570	608
K01210 glucan 1,3-beta-glucosidase [EC:3.2.1.58]	6	6	5	3	1	2	3	2	4	3	3	7
K01212 levanase [EC:3.2.1.65]	0	0	0	0	0	0	1	0	0	1	0	0
K01214 isoamylase [EC:3.2.1.68]	4004	3849	3641	3699	1975	2083	1914	2073	1735	1791	1654	1817
K01226 trehalose-6-phosphate hydrolase [EC:3.2.1.93]	1652	1610	1632	1648	947	967	877	879	2013	2040	1944	2059
K01232 maltose-6'-phosphate glucosidase [EC:3.2.1.122]	2048	2031	1797	2031	1528	1483	1565	1524	2044	2179	2078	2183
K01236 maltooligosyltrehalose trehalohydrolase [EC:3.2.1.141]	2885	2732	2464	2676	1644	1663	1595	1732	744	659	656	745
K01513 ectonucleotide pyrophosphatase/phosphodiesterase family member 1/3 [EC:3.1.4.1 3.6.1.9]	0	0	0	0	0	0	0	0	0	0	1	0
K01838 beta-phosphoglucomutase [EC:5.4.2.6]	782	846	733	784	154	181	196	184	580	608	597	629
K02438 glycogen debranching enzyme [EC:3.2.1.196]	1336	1305	1140	1208	943	956	986	939	1660	1805	1765	1798
K02749 PTS system, alpha-glucoside-specific IIB component [EC:2.7.1.208 2.7.1.-]	2357	2285	2216	2415	1167	1151	1115	1170	3660	4004	3822	4095
K02759 PTS system, cellobiose-specific IIA component [EC:2.7.1.196 2.7.1.205]	1007	1010	927	988	583	640	633	576	1404	1376	1376	1398
K02760 PTS system, cellobiose-specific IIB component [EC:2.7.1.196 2.7.1.205]	1217	1210	1151	1232	751	773	803	742	1416	1525	1441	1475
K02761 PTS system, cellobiose-specific IIC component	6568	6660	6655	6953	4127	4212	4058	3900	8486	8871	8450	8760
K02810 PTS system, sucrose-specific IIC component	2680	2451	2447	2534	1331	1372	1352	1234	2901	2866	2848	3027
K02818 PTS system, trehalose-specific IIB component [EC:2.7.1.201]	1718	1473	1572	1570	828	767	844	833	1852	1918	1822	1887
K05341 amylosucrase [EC:2.4.1.4]	328	322	378	343	44	45	61	42	357	342	371	318
K05342 alpha,alpha-trehalose phosphorylase [EC:2.4.1.64]	826	744	848	842	199	237	216	232	622	683	700	673
K05343 maltose alpha-D-glucosyltransferase / alpha-amylase [EC:5.4.99.16 3.2.1.1]	3055	3110	2805	2903	1416	1289	1298	1444	1916	2019	1916	1962
K05349 beta-glucosidase [EC:3.2.1.21]	26943	28311	29760	28470	68652	67441	69998	67356	59832	59055	60751	58564
K05350 beta-glucosidase [EC:3.2.1.21]	1972	1871	1862	1961	683	711	675	688	2175	2281	2252	2281
K05988 dextranase [EC:3.2.1.11]	1048	1062	1135	1068	3734	3636	3611	3786	3030	2873	3026	2833
K06044 (1->4)-alpha-D-glucan 1-alpha-D-glucosylmutase [EC:5.4.99.15]	4090	4131	3544	3668	2454	2351	2211	2619	1105	1019	893	1005
K06896 maltose 6'-phosphate phosphatase [EC:3.1.3.90]	70	79	81	87	14	16	17	23	73	67	77	57
K07405 alpha-amylase [EC:3.2.1.1]	815	892	860	804	2768	2632	2844	2642	1973	1834	1955	1873
K16055 trehalose 6-phosphate synthase/phosphatase [EC:2.4.1.15 3.1.3.12]	287	273	277	259	343	312	363	313	66	79	52	75
K16147 starch synthase (maltosyl-transferring) [EC:2.4.99.16]	2802	2615	2712	2708	1399	1405	1264	1435	842	785	780	849
K16148 alpha-maltose-1-phosphate synthase [EC:2.4.1.342]	6	1	2	1	0	4	3	0	2	24	7	20
K16149 1,4-alpha-glucan branching enzyme [EC:2.4.1.18]	1	0	0	0	0	0	0	0	0	0	0	0
K16150 glycogen synthase [EC:2.4.1.11]	0	0	0	0	0	0	0	1	0	0	0	0
K16153 glycogen phosphorylase/synthase [EC:2.4.1.1 2.4.1.11]	44	53	33	48	35	46	41	36	30	80	47	53
K20108 PTS system, maltose-specific IIC component	6	15	10	8	5	2	3	3	1	3	6	3
K20811 inulosucrase [EC:2.4.1.9]	0	0	0	0	1	0	3	1	0	2	2	1
K21574 glucan 1,4-alpha-glucosidase [EC:3.2.1.3]	2542	2701	2877	2705	9107	9067	9020	8791	6904	6443	6855	6309
K22451 4-alpha-glucanotransferase [EC:2.4.1.25]	2	3	4	6	77	73	94	43	13	10	8	12
ko00520 Amino sugar and nucleotide sugar metabolism	169556	172176	173086	173341	208423	209681	210430	207887	198209	199143	198548	199329
K00075 UDP-N-acetylmuramate dehydrogenase [EC:1.3.1.98]	2926	2947	3085	2872	3297	3293	3261	3156	3185	3292	3266	3238
K00523 CDP-4-dehydro-6-deoxyglucose reductase, E3 [EC:1.17.1.1]	1709	1616	1575	1618	918	951	974	920	534	497	546	496
K00698 chitin synthase [EC:2.4.1.16]	0	0	0	0	0	0	7	0	0	1	0	1
K00790 UDP-N-acetylglucosamine 1-carboxyvinyltransferase [EC:2.5.1.7]	4554	4585	4568	4572	4698	4801	4764	4706	5044	5245	5026	5183
K00820 glutamine---fructose-6-phosphate transaminase (isomerizing) [EC:2.6.1.16]	7697	8048	7856	7956	7582	7462	7512	7471	6392	6814	6788	6830
K00884 N-acetylglucosamine kinase [EC:2.7.1.59]	483	484	519	542	375	381	354	369	739	658	756	771
K00885 N-acylmannosamine kinase [EC:2.7.1.60]	191	220	185	202	270	358	278	317	64	64	55	50
K00983 N-acylneuraminate cytidylyltransferase [EC:2.7.7.43]	10	18	16	13	6	10	12	3	63	65	46	79
K00992 N-acetyl-alpha-D-muramate 1-phosphate uridylyltransferase [EC:2.7.7.99]	1339	1249	1230	1295	721	691	745	780	413	410	429	394
K01183 chitinase [EC:3.2.1.14]	693	751	706	781	349	363	372	266	1116	1231	1108	1242
K01198 xylan 1,4-beta-xylosidase [EC:3.2.1.37]	2949	2927	3148	3183	4585	4524	4509	4465	4836	4724	4819	5043
K01207 beta-N-acetylhexosaminidase [EC:3.2.1.52]	3873	3574	3672	3646	1612	1688	1524	1610	2495	2586	2467	2596
K01209 alpha-L-arabinofuranosidase [EC:3.2.1.55]	5134	5239	5499	5377	9252	9419	9774	9159	9916	10085	9932	9737
K01443 N-acetylglucosamine-6-phosphate deacetylase [EC:3.5.1.25]	5714	5811	5921	5787	8813	9010	9128	8941	7356	7456	7653	7274
K01452 chitin deacetylase [EC:3.5.1.41]	1	1	2	2	0	3	0	0	1	0	1	0
K01639 N-acetylneuraminate lyase [EC:4.1.3.3]	1266	1367	1353	1351	2186	2157	2082	2110	1842	1946	2011	1995
K01654 N-acetylneuraminate synthase [EC:2.5.1.56]	81	79	104	112	33	27	13	18	69	93	93	87
K01709 CDP-glucose 4,6-dehydratase [EC:4.2.1.45]	892	903	934	926	1446	1528	1459	1458	1188	1176	1313	1096
K01787 N-acylglucosamine 2-epimerase [EC:5.1.3.8]	1056	1133	1271	1118	3503	3522	3513	3443	2641	2483	2686	2408
K01788 N-acylglucosamine-6-phosphate 2-epimerase [EC:5.1.3.9]	890	827	836	870	444	426	382	430	1173	1453	1247	1316
K01791 UDP-N-acetylglucosamine 2-epimerase (non-hydrolysing) [EC:5.1.3.14]	2210	2334	2350	2282	2948	3012	2970	2871	3013	3133	3061	3076
K01836 phosphoacetylglucosamine mutase [EC:5.4.2.3]	0	0	0	0	0	0	0	0	0	0	2	0
K02472 UDP-N-acetyl-D-mannosaminuronic acid dehydrogenase [EC:1.1.1.336]	1073	1115	1046	1080	705	793	713	714	1127	1238	1106	1263
K02473 UDP-N-acetylglucosamine 4-epimerase [EC:5.1.3.7]	65	75	86	87	28	45	20	49	139	182	135	155
K02474 UDP-N-acetyl-D-galactosamine dehydrogenase [EC:1.1.1.-]	121	152	114	120	51	49	53	58	168	227	149	192
K02564 glucosamine-6-phosphate deaminase [EC:3.5.99.6]	3836	3987	4068	4057	8456	8355	8630	8362	7035	6951	6819	6862
K02802 PTS system, N-acetylglucosamine-specific IIA component [EC:2.7.1.193]	0	2	0	0	0	0	0	0	0	1	0	0
K02803 PTS system, N-acetylglucosamine-specific IIB component [EC:2.7.1.193]	4402	4420	4027	4273	2292	2198	2162	2385	2835	2911	2768	2985
K03431 phosphoglucosamine mutase [EC:5.4.2.10]	4357	4643	4514	4594	2419	2499	2259	2366	3789	3992	3756	4056
K04042 bifunctional UDP-N-acetylglucosamine pyrophosphorylase / Glucosamine-1-phosphate N-acetyltransferase [EC:2.7.7.23 2.3.1.157]	3633	3675	3504	3826	2398	2339	2224	2193	2478	2600	2445	2548
K07102 N-acetylmuramate 1-kinase [EC:2.7.1.221]	1837	1852	1700	1876	1147	1061	1063	1155	597	670	627	629
K07106 N-acetylmuramic acid 6-phosphate etherase [EC:4.2.1.126]	1936	1889	2005	1945	2656	2685	2780	2736	2901	2921	2923	2903
K07806 UDP-4-amino-4-deoxy-L-arabinose-oxoglutarate aminotransferase [EC:2.6.1.87]	2602	2532	2364	2549	1401	1376	1402	1624	1672	1653	1566	1572
K08068 UDP-N-acetylglucosamine 2-epimerase (hydrolysing) [EC:3.2.1.183]	0	0	0	0	0	0	0	0	0	2	0	0
K08678 UDP-glucuronate decarboxylase [EC:4.1.1.35]	524	623	646	574	1740	1900	1787	1817	1261	1197	1217	1287
K08679 UDP-glucuronate 4-epimerase [EC:5.1.3.6]	703	653	615	699	420	457	451	424	755	766	696	799
K10011 UDP-4-amino-4-deoxy-L-arabinose formyltransferase / UDP-glucuronic acid dehydrogenase (UDP-4-keto-hexauronic acid decarboxylating) [EC:2.1.2.1	3065	3156	2686	2974	1666	1872	1710	1840	2274	2258	2252	2307
K10012 undecaprenyl-phosphate 4-deoxy-4-formamido-L-arabinose transferase [EC:2.4.2.53]	1848	1900	1714	1763	1129	1069	1079	1080	1451	1494	1295	1530
K11192 PTS system, N-acetylmuramic acid-specific IIC component	578	496	401	500	168	155	135	156	1044	1076	955	1034
K12373 hexosaminidase [EC:3.2.1.52]	12233	13626	14836	13716	42749	42813	43874	42787	31383	30037	31681	30023
K12448 UDP-arabinose 4-epimerase [EC:5.1.3.5]	0	0	2	0	0	0	0	0	0	4	1	0	2
K12452 CDP-4-dehydro-6-deoxyglucose reductase, E1 [EC:1.17.1.1]	955	974	1132	1109	1970	1963	2147	2043	1681	1617	1592	1694
K12454 CDP-paratose 2-epimerase [EC:5.1.3.10]	77	48	81	62	14	5	10	14	2	0	0	0
K13010 perosamine synthetase [EC:2.6.1.102]	0	3	3	5	1	2	4	3	4	5	6	4
K13014 undecaprenyl phosphate-alpha-L-ara4FN deformylase [EC:3.5.1.-]	1766	1864	1676	1751	987	1013	944	1143	1224	1254	1140	1219
K13015 UDP-N-acetyl-D-glucosamine dehydrogenase [EC:1.1.1.136]	1928	1892	2074	1907	1066	1204	1053	1100	644	618	575	630
K13016 UDP-N-acetyl-2-amino-2-deoxyglucuronate dehydrogenase [EC:1.1.1.335]	1411	1353	1464	1495	812	850	777	772	438	463	449	400
K13017 UDP-2-acetamido-2-deoxy-ribo-hexuluronate aminotransferase [EC:2.6.1.98]	1491	1555	1699	1597	866	913	847	958	502	479	465	444
K13018 UDP-2-acetamido-3-amino-2,3-dideoxy-glucuronate N-acetyltransferase [EC:2.3.1.201]	884	880	910	956	511	536	443	416	309	284	253	251
K13019 UDP-GlcNAc3NAcA epimerase [EC:5.1.3.23]	1488	1506	1662	1586	873	871	898	880	476	429	581	443
K13020 UDP-N-acetyl-2-amino-2-deoxyglucuronate dehydrogenase [EC:1.1.1.335]	1	0	0	1	0	0	0	0	0	0	0	0
K13381 bifunctional chitinase/lysozyme [EC:3.2.1.14 3.2.1.17]	6	5	9	8	8	5	12	5	142	179	128	156
K15855 exo-1,4-beta-D-glucosaminidase [EC:3.2.1.165]	0	0	0	0	0	2	0	0	0	0	0	0
K15894 UDP-N-acetylglucosamine 4,6-dehydratase [EC:4.2.1.115]	1	0	0	1	0	0	0	0	1	3	0	3
K15895 UDP-4-amino-4,6-dideoxy-L-N-acetyl-beta-L-altrosamine transaminase [EC:2.6.1.92]	0	0	0	0	0	0	0	0	1	1	0	0
K15898 pseudaminic acid synthase [EC:2.5.1.97]	76	66	53	76	12	9	12	16	116	128	107	136
K15899 pseudaminic acid cytidylyltransferase [EC:2.7.7.81]	13	15	15	15	7	4	5	3	27	21	23	24
K15913 UDP-N-acetylbacillosamine N-acetyltransferase [EC:2.3.1.203]	0	0	0	0	0	0	0	0	0	0	2	0
K15920 xylan 1,4-beta-xylosidase [EC:3.2.1.37]	2	1	0	1	0	0	0	0	1	0	1	0
K15921 arabinoxylan arabinofuranohydrolase [EC:3.2.1.55]	71	81	85	88	311	289	315	278	202	189	232	217
K18429 GDP/UDP-N,N'-diacetylbacillosamine 2-epimerase (hydrolysing) [EC:3.2.1.184]	89	87	95	84	24	35	29	26	25	29	20	38
K18430 N,N'-diacetyllegionaminate synthase [EC:2.5.1.101]	111	131	114	122	46	40	52	35	21	29	24	28
K18431 CMP-N,N'-diacetyllegionaminic acid synthase [EC:2.7.7.82]	57	42	76	44	13	19	40	18	21	24	19	22
K18675 N,N'-diacetylchitobiose phosphorylase [EC:2.4.1.280]	766	758	739	702	132	148	154	109	850	939	955	943
K18676 glucosamine kinase [EC:2.7.1.8]	11	18	15	13	0	0	5	2	8	13	13	7
K21749 N-acylneuraminate/3-deoxy-D-glycero-D-galacto-nononate cytidylyltransferase [EC:2.7.7.43 2.7.7.92]	0	0	0	0	0	0	0	0	0	2	0	1
K22292 N-acetyl-D-muramate 6-phosphate phosphatase [EC:3.1.3.105]	323	325	355	318	179	190	182	214	133	91	114	119
ko00620 Pyruvate metabolism	214233	214477	211102	214083	196205	198334	196154	197638	211431	213018	211213	213044
K00027 malate dehydrogenase (oxaloacetate-decarboxylating) [EC:1.1.1.38]	2930	2877	2871	2983	1923	2090	1951	1870	2048	2395	2230	2361
K00029 malate dehydrogenase (oxaloacetate-decarboxylating)(NADP+) [EC:1.1.1.40]	5209	5189	5154	5318	7027	7403	7149	7200	6273	6175	6137	6120
K00049 glyoxylate/hydroxypyruvate reductase [EC:1.1.1.79 1.1.1.81]	1	0	0	0	0	0	1	1	2	4	2	4
K00051 malate dehydrogenase (NADP+) [EC:1.1.1.82]	0	0	0	0	0	0	0	0	0	0	0	1
K00101 L-lactate dehydrogenase (cytochrome) [EC:1.1.2.3]	4494	4640	4165	4450	2519	2404	2267	2439	2929	3113	2871	3004
K00102 D-lactate dehydrogenase (cytochrome) [EC:1.1.2.4]	28	19	20	25	9	29	24	32	50	50	60	65
K00132 acetaldehyde dehydrogenase (acetylating) [EC:1.2.1.10]	521	502	477	475	101	101	99	77	281	355	272	316
K00156 pyruvate dehydrogenase (quinone) [EC:1.2.5.1]	6126	6183	6034	6165	6661	6490	6576	6476	5418	5462	5494	5374
K00158 pyruvate oxidase [EC:1.2.3.3]	5	6	3	5	17	11	12	6	5	10	8	5
K00467 lactate 2-monooxygenase [EC:1.13.12.4]	1	0	0	0	0	0	0	0	0	0	0	0
K00625 phosphate acetyltransferase [EC:2.3.1.8]	826	908	947	963	2238	2351	2492	2363	1822	1739	1902	1771
K00626 acetyl-CoA C-acetyltransferase [EC:2.3.1.9]	12594	12790	11954	12454	6888	6591	6385	6965	6821	6853	6638	6879

K00656 formate C-acetyltransferase [EC:2.3.1.54]	7945	7917	8001	8345	7622	7705	7755	7440	11462	11561	11251	11563
K00925 acetate kinase [EC:2.7.2.1]	3296	3363	3477	3447	3947	4034	3901	3969	4148	4181	4090	4204
K01003 oxaloacetate decarboxylase [EC:4.1.1.112]	335	364	345	411	187	173	160	228	106	139	139	130
K01006 pyruvate, orthophosphate dikinase [EC:2.7.9.1]	3379	3242	3490	3196	5671	5800	5897	5735	5078	5121	5307	4888
K01007 pyruvate, water dikinase [EC:2.7.9.2]	5017	4873	5022	4913	2926	3187	2887	3093	3465	3464	3380	3573
K01026 propionate CoA-transferase [EC:2.8.3.1]	722	833	699	788	190	225	214	205	1436	1406	1544	1542
K01067 acetyl-CoA hydrolase [EC:3.1.2.1]	0	0	0	0	1	0	0	0	0	0	0	0
K01069 hydroxyacylglutathione hydrolase [EC:3.1.2.6]	3441	3472	3483	3508	3006	3075	3125	3021	2876	2791	2947	2861
K01512 acylphosphatase [EC:3.6.1.7]	739	755	725	761	403	405	428	396	548	515	531	561
K01571 oxaloacetate decarboxylase (Na+ extruding) subunit alpha [EC:7.2.4.2]	1987	1940	2047	1978	651	667	644	615	3462	3549	3473	3659
K01573 oxaloacetate decarboxylase (Na+ extruding) subunit gamma	143	144	156	159	462	434	440	461	356	315	350	349
K01595 phosphoenolpyruvate carboxylase [EC:4.1.1.31]	5733	5478	5363	5511	3393	3386	3216	3462	3684	3584	3501	3668
K01638 malate synthase [EC:2.3.3.9]	4633	4764	4455	4608	2739	2892	2747	2841	2776	2824	2661	2731
K01649 2-isopropylmalate synthase [EC:2.3.3.13]	5799	5824	5830	5944	5937	5857	5723	5763	6806	6975	6960	7000
K01759 lactoylglutathione lyase [EC:4.4.1.5]	3219	3155	3203	3128	2364	2303	2311	2320	2190	2142	2280	2265
K01961 acetyl-CoA carboxylase, biotin carboxylase subunit [EC:6.4.1.2 6.3.4.14]	6545	6692	6582	6701	8752	8665	9021	9035	7342	7278	7398	7276
K01962 acetyl-CoA carboxylase carboxyl transferase subunit alpha [EC:6.4.1.2 2.1.3.15]	1838	1852	1945	1848	1172	1258	1174	1308	1327	1443	1394	1391
K01963 acetyl-CoA carboxylase carboxyl transferase subunit beta [EC:6.4.1.2 2.1.3.15]	3022	2941	3063	2958	1607	1574	1475	1488	2072	2130	1947	2156
K02160 acetyl-CoA carboxylase biotin carboxyl carrier protein	666	678	660	627	438	423	505	462	592	635	563	626
K02594 homocitrate synthase NifV [EC:2.3.3.14]	274	274	209	220	317	353	317	319	12	14	13	13
K03777 D-lactate dehydrogenase (quinone) [EC:1.1.5.12]	1052	1006	934	1000	631	727	666	664	1369	1570	1424	1429
K03778 D-lactate dehydrogenase [EC:1.1.1.28]	2892	3138	2939	2981	3557	3360	3398	3298	3266	3112	3226	3184
K04020 phosphotransacetylase	373	387	341	420	113	142	130	131	823	823	843	868
K04021 aldehyde dehydrogenase	567	554	452	567	122	158	131	162	1096	1108	1107	1202
K04073 acetaldehyde dehydrogenase [EC:1.2.1.10]	315	280	266	314	71	85	72	75	569	558	533	568
K05523 D-lactate dehydratase / protein deglycase [EC:4.2.1.130 3.5.1.124]	1199	1261	1149	1187	683	667	583	658	468	396	394	381
K07248 lactaldehyde dehydrogenase / glycolaldehyde dehydrogenase [EC:1.2.1.22 1.2.1.21]	962	946	970	902	715	838	795	787	1141	999	1092	1161
K10977 methanogen homocitrate synthase [EC:2.3.3.14 2.3.3.-]	0	2	0	0	0	2	2	3	5	7	3	20
K11262 acetyl-CoA carboxylase / biotin carboxylase 1 [EC:6.4.1.2 6.3.4.14 2.1.3.15]	0	1	0	0	0	0	0	0	0	0	0	0
K11263 acetyl-CoA/propionyl-CoA carboxylase, biotin carboxylase, biotin carboxyl carrier protein [EC:6.4.1.2 6.4.1.3 6.3.4.14]	4	0	7	5	8	5	5	11	14	19	19	18
K12972 glyoxylate/hydroxypyruvate reductase [EC:1.1.1.79 1.1.1.81]	1137	1005	1024	1038	669	730	672	733	1340	1421	1401	1441
K13788 phosphate acetyltransferase [EC:2.3.1.8]	3822	3778	3660	3827	2226	2252	2136	2331	2613	2530	2488	2628
K15024 putative phosphotransacetylase [EC:2.3.1.8]	102	134	114	122	32	30	30	23	161	144	144	175
K19266 lactaldehyde dehydrogenase [EC:1.2.1.22]	0	0	3	0	2	2	0	1	1	10	8	19
K20509 carboxybiotin decarboxylase [EC:7.2.4.1]	5192	5339	5507	5349	8011	8264	8525	7967	9880	9486	9603	9806
K22212 malolactic enzyme [EC:4.1.1.101]	9	5	19	13	40	28	14	16	22	33	32	24
K22373 lactate racemase [EC:5.1.2.1]	121	147	151	104	108	137	123	126	155	190	156	177
K23257 methylglyoxal/glyoxal reductase [EC:1.1.1.283 1.1.1.-]	351	360	359	412	62	67	87	69	289	293	351	318
ko00630 Glyoxylate and dicarboxylate metabolism	166949	165626	161838	164554	151801	150936	149939	152568	143537	142741	141973	143116
K00015 glyoxylate reductase [EC:1.1.1.26]	157	160	147	137	30	48	32	35	128	146	125	117
K00018 glycerate dehydrogenase [EC:1.1.1.29]	2829	2934	2829	2928	3384	3389	3173	3221	2443	2232	2343	2242
K00023 acetoacetyl-CoA reductase [EC:1.1.1.36]	4	2	1	5	0	0	0	1	5	0	0	0
K00042 2-hydroxy-3-oxopropionate reductase [EC:1.1.1.60]	1782	1805	1742	1906	1057	1064	1010	1079	1389	1437	1272	1425
K00048 lactaldehyde reductase [EC:1.1.1.77]	1622	1672	1676	1709	2771	2693	2959	2897	3218	3155	3161	3284
K00050 hydroxypyruvate reductase [EC:1.1.1.81]	19	14	16	19	26	30	29	29	5	9	3	3
K00104 glycolate oxidase [EC:1.1.3.15]	2825	2710	2707	2630	1540	1395	1317	1572	1182	1297	1172	1204
K00123 formate dehydrogenase major subunit [EC:1.17.1.9]	3153	3196	3192	3230	2283	2466	2353	2415	3640	3732	3600	3756
K00124 formate dehydrogenase iron-sulfur subunit	2179	2244	2012	2167	1251	1283	1233	1462	1365	1382	1275	1336
K00127 formate dehydrogenase subunit gamma	1306	1270	1185	1191	835	894	803	877	947	891	989	1022
K00281 glycine dehydrogenase [EC:1.4.4.2]	11909	11722	11036	11585	10117	10076	9784	10444	8371	8044	7883	8286
K00282 glycine dehydrogenase subunit 1 [EC:1.4.4.2]	261	244	285	300	150	171	153	151	244	291	207	261
K00283 glycine dehydrogenase subunit 2 [EC:1.4.4.2]	502	502	578	500	186	208	238	206	421	447	410	401
K00284 glutamate synthase (ferredoxin) [EC:1.4.7.1]	0	1	0	1	1	0	1	0	0	0	1	2
K00600 glycine hydroxymethyltransferase [EC:2.1.2.1]	7337	7324	7059	7016	5975	5980	5914	5982	5305	5019	4966	5089
K00605 aminomethyltransferase [EC:2.1.2.10]	5198	4861	4793	4843	4648	4471	4657	4688	3830	3582	3702	3787
K00830 alanine-glyoxylate transaminase / serine-glyoxylate transaminase / serine-pyruvate transaminase [EC:2.6.1.44 2.6.1.45 2.6.1.51]	1	9	3	7	0	4	0	2	7	14	3	7
K00865 glycerate 2-kinase [EC:2.7.1.165]	3393	3614	3388	3486	3549	3779	3714	3650	4913	4975	4969	4881
K01091 phosphoglycolate phosphatase [EC:3.1.3.18]	5374	5387	5416	5395	5363	5279	5455	5452	4357	4435	4476	4410
K01432 arylformamidase [EC:3.5.1.9]	0	2	1	1	0	4	2	1	0	2	2	1
K01433 formyltetrahydrofolate deformylase [EC:3.5.1.10]	3475	3469	3512	3572	3545	3496	3489	3359	2886	2707	2745	2634
K01455 formamidase [EC:3.5.1.49]	56	39	52	47	3	5	3	0	112	91	98	107
K01458 N-formylglutamate deformylase [EC:3.5.1.68]	424	424	448	427	371	395	409	354	86	102	87	71
K01569 oxalate decarboxylase [EC:4.1.1.2]	1	0	0	0	0	0	0	0	0	0	0	0
K01577 oxalyl-CoA decarboxylase [EC:4.1.1.8]	8	11	11	3	28	23	26	22	80	104	109	97
K01601 ribulose-bisphosphate carboxylase large chain [EC:4.1.1.39]	8	10	10	13	0	4	4	2	3	5	8	9
K01608 tartronate-semialdehyde synthase [EC:4.1.1.47]	175	199	195	201	117	120	120	119	288	306	312	265
K01637 isocitrate lyase [EC:4.1.3.1]	2456	2370	2381	2529	1549	1591	1423	1487	1664	1720	1641	1694
K01816 hydroxypyruvate isomerase [EC:5.3.1.22]	828	684	797	794	343	352	389	379	388	451	452	453
K01846 methylaspartate mutase sigma subunit [EC:5.4.99.1]	20	23	9	15	2	0	7	3	38	43	41	27
K01847 methylmalonyl-CoA mutase [EC:5.4.99.2]	2870	2877	3145	3015	8943	9073	9289	8726	6520	6283	6668	6373
K01848 methylmalonyl-CoA mutase, N-terminal domain [EC:5.4.99.2]	103	124	113	115	10	2	10	4	242	296	269	261
K01849 methylmalonyl-CoA mutase, C-terminal domain [EC:5.4.99.2]	19	37	23	26	0	0	2	2	48	51	41	50
K01915 glutamine synthetase [EC:6.3.1.2]	22068	21588	21624	21420	21354	20720	21133	21547	16407	16493	16608	16343
K01965 propionyl-CoA carboxylase alpha chain [EC:6.4.1.3]	2	2	0	1	2	4	0	1	0	5	5	7
K01966 propionyl-CoA carboxylase beta chain [EC:6.4.1.3 2.1.3.15]	1894	1885	2156	2030	6363	6046	6237	6211	4383	4390	4578	4345
K02437 glycine cleavage system H protein	1982	1966	1784	1905	1825	1775	1737	1836	1417	1442	1516	1506
K03418 N,N-dimethylformamidase [EC:3.5.1.56]	0	0	0	0	0	0	0	0	0	2	2	5
K03779 L(+)-tartrate dehydratase alpha subunit [EC:4.2.1.32]	609	563	623	564	195	206	191	208	886	1000	931	905
K03780 L(+)-tartrate dehydratase beta subunit [EC:4.2.1.32]	499	521	487	475	199	193	200	198	706	806	727	755
K03781 catalase [EC:1.11.1.6]	7175	7002	7010	7190	3948	3758	3777	3844	4126	4085	3953	4001
K04835 methylaspartate ammonia-lyase [EC:4.3.1.2]	52	44	40	40	3	4	0	2	105	95	109	103
K05606 methylmalonyl-CoA/ethylmalonyl-CoA epimerase [EC:5.1.99.1]	287	276	345	291	832	861	851	888	681	579	632	656
K07130 arylformamidase [EC:3.5.1.9]	1003	934	847	867	537	475	426	542	339	339	350	383
K07246 tartrate dehydrogenase/decarboxylase / D-malate dehydrogenase [EC:1.1.1.93 4.1.1.73 1.1.1.83]	467	470	431	461	154	167	178	166	821	840	801	868
K11472 glycolate oxidase FAD binding subunit	2049	1906	1633	1820	1001	976	865	1061	660	655	584	638
K11473 glycolate oxidase iron-sulfur subunit	1792	1803	1602	1702	949	900	898	1001	701	639	632	633
K11517 (S)-2-hydroxy-acid oxidase [EC:1.1.3.15]	0	0	2	0	0	0	0	0	0	0	0	0
K14067 malate-CoA ligase subunit beta [EC:6.2.1.9]	0	0	0	0	0	0	0	0	0	1	0	0
K15893 glycerate dehydrogenase [EC:1.1.1.29]	0	0	3	0	0	0	0	0	2	5	3	4
K15919 glyoxylate/hydroxypyruvate reductase [EC:1.1.1.79 1.1.1.81]	6	1	2	0	2	5	3	1	3	5	8	0
K17865 3-hydroxybutyryl-CoA dehydratase [EC:4.2.1.55]	14	16	10	9	0	2	0	2	121	81	95	81
K18123 4-hydroxy-2-oxoglutarate aldolase [EC:4.1.3.16]	4	1	2	2	0	0	1	0	2	8	3	3
K19268 methylaspartate mutase epsilon subunit [EC:5.4.99.1]	49	52	55	47	4	6	7	2	116	143	116	155
K19312 acetyl-CoA/propionyl-CoA carboxylase carboxyl transferase subunit [EC:6.4.1.2 6.4.1.3 2.1.3.15]	17	12	9	14	0	2	3	1	15	16	22	20
K22223 phosphoglycolate phosphatase [EC:3.1.3.18]	8	12	19	17	8	4	3	9	11	11	12	16
K22982 hydroxypyruvate reductase [EC:1.1.1.81]	1	0	1	1	0	1	0	0	8	5	2	8
ko00640 Propanoate metabolism	135970	136336	133365	136367	121875	120804	120364	121682	126428	126127	125023	127665
K00005 glycerol dehydrogenase [EC:1.1.1.6]	1910	1939	2001	2111	1263	1249	1224	1189	2681	2744	2660	2733
K00086 1,3-propanediol dehydrogenase [EC:1.1.1.202]	518	506	473	532	141	150	138	109	1006	984	967	964
K00140 malonate-semialdehyde dehydrogenase (acetylating) / methylmalonate-semialdehyde dehydrogenase [EC:1.2.1.18 1.2.1.27]	6337	6070	6133	6269	3295	3105	3090	3356	3449	3543	3356	3453
K00166 2-oxoisovalerate dehydrogenase E1 component alpha subunit [EC:1.2.4.4]	1634	1549	1476	1553	856	722	674	865	489	529	491	461
K00167 2-oxoisovalerate dehydrogenase E1 component beta subunit [EC:1.2.4.4]	1569	1482	1369	1523	754	670	681	873	509	493	421	478
K00232 acyl-CoA oxidase [EC:1.3.3.6]	0	0	0	0	0	0	0	0	0	0	3	0
K00248 butyryl-CoA dehydrogenase [EC:1.3.8.1]	2181	2107	1995	2105	1256	1107	1142	1159	1076	1071	960	1072
K00822 beta-alanine--pyruvate transaminase [EC:2.6.1.18]	4661	4602	4448	4407	2607	2372	2306	2470	1425	1440	1256	1422
K00823 4-aminobutyrate aminotransferase [EC:2.6.1.19]	796	809	810	773	558	572	560	509	903	1031	887	1109
K00932 propionate kinase [EC:2.7.2.15]	495	410	364	515	149	130	143	151	929	1046	970	1026
K01034 acetate CoA/acetoacetate CoA-transferase alpha subunit [EC:2.8.3.8 2.8.3.9]	5	4	7	3	6	4	8	10	27	49	39	50
K01035 acetate CoA/acetoacetate CoA-transferase beta subunit [EC:2.8.3.8 2.8.3.9]	25	28	26	17	1	7	6	10	47	58	56	71
K01574 acetoacetate decarboxylase [EC:4.1.1.4]	5	0	1	3	0	2	1	1	6	5	6	1
K01578 malonyl-CoA decarboxylase [EC:4.1.1.9]	1	0	0	0	0	2	0	0	0	0	0	0
K01659 2-methylcitrate synthase [EC:2.3.3.5]	1830	1831	1579	1644	922	925	899	1037	615	553	641	634
K01692 enoyl-CoA hydratase [EC:4.2.1.17]	2564	2527	2395	2447	1388	1298	1221	1333	1259	1171	1202	1224
K01699 propanediol dehydratase large subunit [EC:4.2.1.28]	1066	1114	1062	1073	641	776	709	662	1396	1484	1426	1608
K01720 2-methylcitrate dehydratase [EC:4.2.1.79]	2277	2286	2125	2145	1242	1197	1176	1242	784	708	696	790
K01734 methylglyoxal synthase [EC:4.2.3.3]	1150	1399	1222	1243	1539	1518	1570	1388	1740	1838	1854	1746
K01782 3-hydroxyacyl-CoA dehydrogenase / enoyl-CoA hydratase / 3-hydroxybutyryl-CoA epimerase [EC:1.1.1.35 4.2.1.17 5.1.2.3]	4154	4016	3994	4206	2376	2402	2240	2417	2705	2627	2473	2733
K01825 3-hydroxyacyl-CoA dehydrogenase / enoyl-CoA hydratase / 3-hydroxybutyryl-CoA epimerase / enoyl-CoA isomerase [EC:1.1.1.35 4.2.1.17 5.1.2.3 5.3.3	4786	4649	4709	4546	2924	2929	2807	2978	2916	2903	2954	3089
K01908 propionyl-CoA synthetase [EC:6.2.1.17]	2515	2413	2227	2350	1265	1273	1247	1381	933	940	832	871
K03416 methylmalonyl-CoA carboxyltransferase 5S subunit [EC:2.1.3.1]	0	1	1	1	1	0	0	0	1	0	0	0
K03417 methylisocitrate lyase [EC:4.1.3.30]	1310	1195	1095	1202	658	641	591	669	458	404	413	404

K05605 3-hydroxyisobutyryl-CoA hydrolase [EC:3.1.2.4]	0	2	0	0	1	1	0	0	0	0	0	0
K07250 4-aminobutyrate aminotransferase / (S)-3-amino-2-methylpropionate transaminase / 5-aminovalerate transaminase [EC:2.6.1.19 2.6.1.22 2.6.1.48]	734	766	717	757	193	186	210	197	1689	1785	1611	1652
K07511 enoyl-CoA hydratase [EC:4.2.1.17]	0	0	2	1	0	0	0	0	1	0	0	0
K08325 NADP-dependent alcohol dehydrogenase [EC:1.1.-.-]	1535	1559	1482	1559	2777	2916	2841	2880	2702	2590	2641	2548
K09699 2-oxoisovalerate dehydrogenase E2 component (dihydrolipoyl transacylase) [EC:2.3.1.168]	1217	1181	1058	1257	643	558	501	681	395	376	383	397
K09788 2-methylaconitate isomerase [EC:5.3.3.-]	1552	1548	1442	1392	775	686	824	896	492	507	444	429
K11264 methylmalonyl-CoA decarboxylase [EC:4.1.1.-]	0	0	0	0	11	8	3	3	2	3	0	0
K11381 2-oxoisovalerate dehydrogenase E1 component [EC:1.2.4.4]	1194	1302	1407	1331	4001	4056	4138	4040	2733	2770	2799	2600
K13919 propanediol dehydratase medium subunit [EC:4.2.1.28]	405	439	389	432	313	271	310	247	557	489	603	598
K13920 propanediol dehydratase small subunit [EC:4.2.1.28]	340	343	300	329	200	192	220	217	403	405	415	494
K13921 1-propanol dehydrogenase	728	733	727	778	435	485	432	457	958	906	970	955
K13922 propionaldehyde dehydrogenase [EC:1.2.1.87]	1002	1096	1023	1077	639	658	626	596	1389	1314	1349	1412
K13923 phosphate propanoyltransferase [EC:2.3.1.222]	321	407	274	364	239	305	236	267	447	483	438	482
K14729 multifunctional beta-oxidation protein [EC:4.2.1.- 1.1.1.-]	1	0	0	0	0	2	0	0	0	0	0	0
K17489 methylmalonyl-CoA carboxyltransferase 12S subunit [EC:2.1.3.1]	17	9	7	12	1	0	2	2	7	15	18	28
K18369 alcohol dehydrogenase [EC:1.1.1.-]	865	917	1009	960	1432	1404	1330	1328	1336	1315	1299	1395
K18471 methylglyoxal reductase [EC:1.1.1.-]	482	478	461	465	390	400	390	359	744	798	696	730
K19697 propionate kinase [EC:2.7.2.15]	716	720	688	802	430	432	449	467	928	971	890	1050
K19709 acetate CoA-transferase [EC:2.8.3.8]	85	78	77	59	118	133	115	135	205	172	191	216
K19745 acrylyl-CoA reductase (NADPH) [EC:1.3.1.-]	469	513	420	496	375	393	353	390	607	696	633	708
K20143 acrylyl-CoA reductase (NADH) [EC:1.3.1.95]	3	2	0	0	0	0	2	0	19	15	10	14
K20455 2-methylcitrate dehydratase (2-methyl-trans-aconitate forming) [EC:4.2.1.117]	2716	2810	2478	2598	1542	1344	1370	1497	836	783	709	748
K20626 lactoyl-CoA dehydratase subunit alpha [EC:4.2.1.54]	9	6	12	15	2	0	0	0	35	45	39	57
K20627 lactoyl-CoA dehydratase subunit beta [EC:4.2.1.54]	9	5	7	10	6	7	8	5	53	51	51	44
K22214 propionyl-CoA:succinyl-CoA transferase [EC:2.8.3.-]	0	0	0	0	14	9	6	23	9	4	3	1
K23259 isopropanol dehydrogenase (NADP+) [EC:1.1.1.80]	55	49	63	66	6	9	10	6	16	9	9	7
ko00650 Butanoate metabolism	135646	135907	133457	136759	114541	115903	114379	115720	128075	130074	127806	130251
K00004 (R,R)-butanediol dehydrogenase / meso-butanediol dehydrogenase / diacetyl reductase [EC:1.1.1.4 1.1.1.- 1.1.1.303]	3444	3483	3113	3297	1824	1759	1697	1874	1076	1003	1054	1109
K00019 3-hydroxybutyrate dehydrogenase [EC:1.1.1.30]	1498	1522	1433	1481	830	811	726	800	793	912	898	970
K00022 3-hydroxyacyl-CoA dehydrogenase [EC:1.1.1.35]	4	0	0	0	0	0	0	1	0	0	0	0
K00043 4-hydroxybutyrate dehydrogenase [EC:1.1.1.61]	0	1	0	0	0	0	0	0	0	1	1	0
K00074 3-hydroxybutyryl-CoA dehydrogenase [EC:1.1.1.157]	5128	5060	4541	4899	2552	2575	2367	2719	3579	3720	3337	3574
K00100 butanol dehydrogenase [EC:1.1.1.-]	1738	1689	1622	1580	834	913	861	940	597	586	619	681
K00135 succinate-semialdehyde dehydrogenase / glutarate-semialdehyde dehydrogenase [EC:1.2.1.16 1.2.1.79 1.2.1.20]	6810	6633	6518	6842	3726	3547	3634	3664	4741	4836	4859	5036
K00139 succinate-semialdehyde dehydrogenase [EC:1.2.1.24]	1	0	0	1	0	0	0	0	0	0	0	0
K00209 enoyl-[acyl-carrier protein] reductase / trans-2-enoyl-CoA reductase (NAD+) [EC:1.3.1.9 1.3.1.44]	1825	1887	1679	1778	986	959	898	1029	630	663	627	641
K00634 phosphate butyryltransferase [EC:2.3.1.19]	490	477	584	546	907	821	877	760	674	710	743	709
K00929 butyrate kinase [EC:2.7.2.7]	1473	1531	1662	1468	2622	2513	2600	2609	2351	2331	2333	2340
K01027 3-oxoacid CoA-transferase [EC:2.8.3.5]	3	5	5	3	9	11	8	7	6	10	10	6
K01028 3-oxoacid CoA-transferase subunit A [EC:2.8.3.5]	535	564	506	593	365	405	378	431	568	607	549	584
K01029 3-oxoacid CoA-transferase subunit B [EC:2.8.3.5]	562	526	450	542	313	320	347	333	470	568	497	534
K01039 glutaconate CoA-transferase, subunit A [EC:2.8.3.12]	639	643	562	667	357	371	367	379	216	174	199	208
K01040 glutaconate CoA-transferase, subunit B [EC:2.8.3.12]	1338	1295	1227	1187	814	755	702	720	438	431	358	417
K01575 acetolactate decarboxylase [EC:4.1.1.5]	426	473	451	504	286	330	364	277	640	634	594	666
K01580 glutamate decarboxylase [EC:4.1.1.15]	1039	1071	1083	1066	3318	3331	3323	3374	2576	2482	2624	2516
K01615 glutaconyl-CoA decarboxylase subunit alpha [EC:7.2.4.5]	25	35	36	30	67	79	76	89	26	37	40	36
K01640 hydroxymethylglutaryl-CoA lyase [EC:4.1.3.4]	1325	1249	1153	1244	662	657	638	652	447	425	397	373
K01641 hydroxymethylglutaryl-CoA synthase [EC:2.3.3.10]	56	93	87	56	19	21	27	20	70	113	81	89
K01652 acetolactate synthase I/II/III large subunit [EC:2.2.1.6]	16148	16395	15882	16536	11375	11742	11289	11370	13807	14064	13638	14040
K01653 acetolactate synthase I/III small subunit [EC:2.2.1.6]	2139	2147	2225	2167	2163	2234	2198	2149	2281	2397	2291	2373
K01715 enoyl-CoA hydratase [EC:4.2.1.17]	178	202	190	225	132	114	114	119	260	357	355	361
K01799 maleate isomerase [EC:5.2.1.1]	3	2	2	3	0	0	0	0	3	0	2	1
K01907 acetoacetyl-CoA synthetase [EC:6.2.1.16]	2504	2541	2409	2385	1287	1314	1136	1379	796	745	688	726
K03366 meso-butanediol dehydrogenase / (S,S)-butanediol dehydrogenase / diacetyl reductase [EC:1.1.1.- 1.1.1.76 1.1.1.304]	868	834	869	857	771	899	840	778	576	616	578	692
K03821 polyhydroxyalkanoate synthase subunit PhaC [EC:2.3.1.-]	5497	5392	5402	5410	2994	2931	2851	3101	1824	1632	1603	1702
K07516 3-hydroxyacyl-CoA dehydrogenase [EC:1.1.1.35]	11	3	5	1	16	13	24	20	1	4	2	7
K07518 hydroxybutyrate-dimer hydrolase [EC:3.1.1.22]	0	0	0	0	0	0	0	0	0	2	0	0
K08318 4-hydroxybutyrate dehydrogenase / sulfolactaldehyde 3-reductase [EC:1.1.1.61 1.1.1.373]	263	248	227	235	348	395	364	352	66	67	98	79
K08324 succinate-semialdehyde dehydrogenase [EC:1.2.1.16 1.2.1.24]	815	685	639	827	553	589	521	529	1000	1118	985	1134
K11258 acetolactate synthase II small subunit [EC:2.2.1.6]	179	151	146	175	122	133	119	115	220	198	173	230
K14268 5-aminovalerate/4-aminobutyrate aminotransferase [EC:2.6.1.48 2.6.1.19]	2343	2184	2110	2276	1241	1257	1264	1354	679	688	645	610
K14534 4-hydroxybutyryl-CoA dehydratase / vinylacetyl-CoA-Delta-isomerase [EC:4.2.1.120 5.3.3.3]	780	723	818	827	127	121	133	109	1441	1486	1568	1619
K16871 4-aminobutyrate---pyruvate transaminase [EC:2.6.1.96]	14	17	16	27	0	2	2	0	12	12	15	8
K18009 meso-butanediol dehydrogenase / (S,S)-butanediol dehydrogenase / diacetyl reductase [EC:1.1.1.- 1.1.1.76 1.1.1.304]	1	2	2	3	2	4	3	3	2	1	1	0
K18119 succinate-semialdehyde dehydrogenase [EC:1.2.1.76]	3	12	2	8	1	0	2	0	0	3	0	7
K18120 4-hydroxybutyrate dehydrogenase [EC:1.1.1.61]	81	53	56	56	19	20	15	16	17	23	38	34
K18122 4-hydroxybutyrate CoA-transferase [EC:2.8.3.-]	65	72	72	74	35	49	29	36	39	55	47	45
K23351 glutaconyl-CoA/methylmalonyl-CoA decarboxylase subunit gamma	417	444	456	432	963	903	899	908	721	843	788	670
K23352 glutaconyl-CoA/methylmalonyl-CoA decarboxylase subunit delta	198	251	239	213	49	49	43	53	175	249	223	201
ko00660 C5-Branched dibasic acid metabolism	37781	38036	37586	38769	35226	35582	34868	34361	36217	36500	35721	36542
K00052 3-isopropylmalate dehydrogenase [EC:1.1.1.85]	2570	2661	2578	2686	3458	3326	3436	3182	3374	3477	3304	3389
K01703 3-isopropylmalate/(R)-2-methylmalate dehydratase large subunit [EC:4.2.1.33 4.2.1.35]	4521	4354	4339	4552	4913	5049	4994	4747	4882	4859	4710	4855
K01704 3-isopropylmalate/(R)-2-methylmalate dehydratase small subunit [EC:4.2.1.33 4.2.1.35]	1899	1885	1875	1955	2206	2229	2088	2223	1894	2033	2112	1963
K09011 (R)-citramalate synthase [EC:2.3.1.182]	751	807	800	766	2452	2524	2365	2523	1770	1703	1705	1687
K10218 4-hydroxy-4-methyl-2-oxoglutarate aldolase [EC:4.1.3.17]	301	329	292	358	106	95	87	89	633	605	623	651
K18288 itaconate CoA-transferase [EC:2.8.3.-]	3	2	1	0	0	0	1	1	3	4	3	6
K18289 itaconate CoA-transferase [EC:2.8.3.- 2.8.3.22]	1841	1785	1804	1878	1022	952	875	974	537	481	526	574
K18291 itaconyl-CoA hydratase / mesaconyl-C4 CoA hydratase [EC:4.2.1.56 4.2.1.-]	1174	1149	1161	1190	619	628	637	631	392	346	323	373
K18292 (S)-citramalyl-CoA lyase [EC:4.1.3.25]	593	657	638	686	385	395	352	357	225	216	174	199
K22004 trans-aconitate decarbocylase [EC:4.1.1.-]	0	0	0	0	0	0	0	1	0	0	0	0
ko00562 Inositol phosphate metabolism	25821	26006	25548	26338	17182	17350	16758	17402	27334	28147	27205	28180
K00010 myo-inositol 2-dehydrogenase / D-chiro-inositol 1-dehydrogenase [EC:1.1.1.18 1.1.1.369]	1408	1350	1304	1378	282	287	299	281	2542	2713	2549	2702
K00889 1-phosphatidylinositol-4-phosphate 5-kinase [EC:2.7.1.68]	19	18	24	22	75	67	0	0	47	57	69	43
K01092 myo-inositol-1(or 4)-monophosphatase [EC:3.1.3.25]	2188	2392	2374	2397	2796	2862	2851	2860	3783	3738	3833	3876
K01093 4-phytase / acid phosphatase [EC:3.1.3.26 3.1.3.2]	432	537	445	505	153	125	115	109	1025	1067	985	1022
K01114 phospholipase C [EC:3.1.4.3]	6513	6572	6098	6453	3388	3540	3352	3634	2143	1954	1808	2070
K01771 1-phosphatidylinositol phosphodiesterase [EC:4.6.1.13]	2	0	2	1	1	5	11	17	19	11	12	7
K01858 myo-inositol-1-phosphate synthase [EC:5.5.1.4]	804	815	981	800	2615	2637	2548	2656	1886	1832	1970	1759
K03335 inosose dehydratase [EC:4.2.1.44]	1403	1322	1315	1416	355	330	291	352	2619	2923	2752	2996
K03336 3D-(3,5/4)-trihydroxycyclohexane-1,2-dione acylhydrolase (decyclizing) [EC:3.7.1.22]	1345	1287	1237	1319	292	276	260	258	2242	2401	2196	2405
K03337 5-deoxy-glucuronate isomerase [EC:5.3.1.30]	506	465	474	531	123	107	117	89	838	901	826	916
K03338 5-dehydro-2-deoxygluconokinase [EC:2.7.1.92]	1003	1010	1025	1075	268	279	257	240	1908	2041	1992	2085
K03339 6-phospho-5-dehydro-2-deoxy-D-gluconate aldolase [EC:4.1.2.29]	2	0	0	0	5	2	1	0	0	0	0	0
K06606 2-keto-myo-inositol isomerase [EC:5.3.99.11]	319	371	357	386	88	96	92	99	792	870	739	791
K13711 phosphatidylinositol 4-kinase type 2 [EC:2.7.1.67]	0	0	0	0	0	0	0	0	0	1	0	0
K16043 scyllo-inositol 2-dehydrogenase (NAD+) [EC:1.1.1.370]	1	1	3	1	0	0	0	0	12	7	10	12
K16044 scyllo-inositol 2-dehydrogenase (NADP+) [EC:1.1.1.371]	0	0	2	0	7	5	9	3	3	0	0	0
K19517 1D-myo-inositol 3-kinase [EC:2.7.1.64]	0	0	0	0	0	0	1	0	2	0	0	0
K20279 synaptojanin [EC:3.1.3.36]	0	0	0	0	0	0	0	0	0	0	2	0
K22230 scyllo-inositol 2-dehydrogenase (NADP+) [EC:1.1.1.-]	274	263	287	270	78	63	59	79	162	180	183	171
K22232 3-dehydro-scyllo-inosose hydrolase [EC:3.7.1.-]	4	3	2	2	3	2	4	5	9	10	12	1
K22233 5-keto-L-gluconate epimerase [EC:5.1.-.-]	3	2	2	3	0	0	0	0	3	5	5	2
Energy metabolism	609856	609815	605232	611512	560444	566647	558081	560060	560446	560068	556434	559554
ko00190 Oxidative phosphorylation	145690	146078	147707	148635	137554	140073	137205	137298	132458	132786	132346	132247
K00330 NADH-quinone oxidoreductase subunit A [EC:7.1.1.2]	1433	1445	1509	1515	1399	1425	1357	1446	1177	1089	1165	1146
K00331 NADH-quinone oxidoreductase subunit B [EC:7.1.1.2]	1446	1366	1571	1441	1930	2135	2012	1793	1664	1547	1612	1580
K00332 NADH-quinone oxidoreductase subunit C [EC:7.1.1.2]	3	0	2	1	0	0	0	3	0	5	4	5
K00333 NADH-quinone oxidoreductase subunit D [EC:7.1.1.2]	4	5	5	9	6	0	3	3	13	8	6	12
K00334 NADH-quinone oxidoreductase subunit E [EC:7.1.1.2]	1153	1088	1127	1214	624	648	609	596	889	1012	974	1041
K00335 NADH-quinone oxidoreductase subunit F [EC:7.1.1.2]	4128	4299	4308	4210	1920	1947	1939	2061	3183	3392	3218	3386
K00336 NADH-quinone oxidoreductase subunit G [EC:7.1.1.2]	6013	6018	5860	6234	3570	3483	3245	3546	4424	4570	4417	4514
K00337 NADH-quinone oxidoreductase subunit H [EC:7.1.1.2]	2556	2557	2583	2712	3415	3447	3372	3358	2974	2834	2864	2736
K00338 NADH-quinone oxidoreductase subunit I [EC:7.1.1.2]	1383	1360	1410	1480	1713	1763	1582	1600	1482	1406	1451	1448
K00339 NADH-quinone oxidoreductase subunit J [EC:7.1.1.2]	1086	1116	1091	1124	1593	1603	1651	1537	1471	1293	1388	1387
K00340 NADH-quinone oxidoreductase subunit K [EC:7.1.1.2]	695	695	677	775	930	936	940	1005	826	775	802	743
K00341 NADH-quinone oxidoreductase subunit L [EC:7.1.1.2]	4976	4931	5089	5096	6336	6697	6358	6523	5528	5094	5373	5320
K00342 NADH-quinone oxidoreductase subunit M [EC:7.1.1.2]	3813	4033	4058	3920	4784	4774	4475	4797	4009	4056	4065	3896
K00343 NADH-quinone oxidoreductase subunit N [EC:7.1.1.2]	3847	3828	3915	3895	4945	4961	4795	4726	4168	4039	4024	3935
K00356 NADH dehydrogenase [EC:1.6.99.3]	1	2	1	0	0	0	2	3	4	1	2	4
K00404 cytochrome c oxidase cbb3-type subunit I [EC:1.9.3.1]	7457	7450	7363	7317	4004	3861	3840	4044	2358	2173	2352	2334
K00405 cytochrome c oxidase cbb3-type subunit II	987	1037	987	1061	601	503	561	567	378	343	328	352

K00406 cytochrome c oxidase cbb3-type subunit III	1334	1269	1349	1174	697	688	604	624	429	413	352	375
K00407 cytochrome c oxidase cbb3-type subunit IV	513	481	484	504	268	283	262	264	164	156	165	146
K00410 ubiquinol-cytochrome c reductase cytochrome b/c1 subunit	0	0	0	0	0	1	0	0	0	0	0	0
K00411 ubiquinol-cytochrome c reductase iron-sulfur subunit [EC:7.1.1.8]	810	772	828	843	548	545	478	471	310	295	276	259
K00412 ubiquinol-cytochrome c reductase cytochrome b subunit	1861	1865	1926	1879	1034	1152	1052	1024	620	583	604	575
K00413 ubiquinol-cytochrome c reductase cytochrome c1 subunit	997	1054	1090	1087	591	600	575	557	331	378	288	312
K00424 cytochrome bd-I ubiquinol oxidase subunit X [EC:7.1.1.7]	81	50	56	68	53	50	63	43	85	105	98	86
K00425 cytochrome bd ubiquinol oxidase subunit I [EC:7.1.1.7]	5480	5553	5511	5510	5784	5813	5934	5746	7072	7205	7038	7319
K00426 cytochrome bd ubiquinol oxidase subunit II [EC:7.1.1.7]	2959	2867	2991	3017	3849	3798	3878	3585	4715	4781	4544	4698
K00937 polyphosphate kinase [EC:2.7.4.1]	6997	7138	7219	7038	6878	6880	7000	6953	6127	5929	6021	6240
K01507 inorganic pyrophosphatase [EC:3.6.1.1]	1496	1529	1406	1505	849	861	863	864	1326	1449	1351	1374
K01535 H+-transporting ATPase [EC:7.1.2.1]	1	5	0	1	0	0	0	0	0	0	0	0
K02107 V/A-type H+/Na+-transporting ATPase subunit G/H	21	20	16	22	5	8	3	1	21	30	24	23
K02108 F-type H+-transporting ATPase subunit a	3450	3448	3579	3489	3547	3695	3655	3531	3459	3383	3469	3219
K02109 F-type H+-transporting ATPase subunit b	1309	1405	1420	1390	1419	1309	1334	1293	1368	1222	1300	1215
K02110 F-type H+-transporting ATPase subunit c	912	941	961	977	765	765	746	710	810	801	820	785
K02111 F-type H+/Na+-transporting ATPase subunit alpha [EC:7.1.2.2 7.2.2.1]	6594	6558	6528	6717	5992	5937	6007	6005	6024	5856	6208	6156
K02112 F-type H+/Na+-transporting ATPase subunit beta [EC:7.1.2.2 7.2.2.1]	5890	5915	6324	6109	5721	5799	5765	5756	5414	5731	5557	5672
K02113 F-type H+-transporting ATPase subunit delta	2032	2047	2064	2153	2016	2074	2171	1973	1919	2025	1985	1931
K02114 F-type H+-transporting ATPase subunit epsilon	1484	1561	1664	1533	1374	1311	1162	1234	1277	1285	1287	1235
K02115 F-type H+-transporting ATPase subunit gamma	3407	3367	3468	3463	3059	3303	3183	3135	3288	3359	3264	3258
K02117 V/A-type H+/Na+-transporting ATPase subunit A [EC:7.1.2.2 7.2.2.1]	2616	2519	2787	2696	3612	3909	3931	3644	3812	3941	3968	3900
K02118 V/A-type H+/Na+-transporting ATPase subunit B	2186	2117	2181	2127	3166	3183	3224	3030	3171	3295	3139	3056
K02119 V/A-type H+/Na+-transporting ATPase subunit C	849	753	836	813	203	208	248	200	712	759	663	800
K02120 V/A-type H+/Na+-transporting ATPase subunit D	976	993	980	946	1307	1235	1259	1374	1341	1334	1450	1248
K02121 V/A-type H+/Na+-transporting ATPase subunit E	340	425	433	411	998	1052	1048	1038	733	780	854	810
K02122 V/A-type H+/Na+-transporting ATPase subunit F	315	315	287	303	85	81	95	76	321	272	298	284
K02123 V/A-type H+/Na+-transporting ATPase subunit I	2506	2549	2727	2650	4058	4216	4044	3975	3810	3912	3938	3944
K02124 V/A-type H+/Na+-transporting ATPase subunit K	599	600	591	594	1049	1056	1043	999	890	932	978	915
K02126 F-type H+-transporting ATPase subunit a	0	2	0	0	0	0	0	0	0	0	0	0
K02128 F-type H+-transporting ATPase subunit c	0	2	0	0	0	0	0	0	0	0	0	0
K02132 F-type H+-transporting ATPase subunit alpha	3	3	11	6	1	2	1	0	12	6	15	5
K02133 F-type H+-transporting ATPase subunit beta [EC:7.1.2.2]	2	1	0	1	3	3	3	1	0	1	3	2
K02145 V-type H+-transporting ATPase subunit A [EC:7.1.2.2]	0	0	0	0	0	1	0	0	0	0	0	0
K02147 V-type H+-transporting ATPase subunit B	0	0	0	1	0	0	0	0	0	0	0	0
K02256 cytochrome c oxidase subunit 1 [EC:1.9.3.1]	0	0	0	0	0	1	3	0	2	0	0	0
K02257 heme o synthase [EC:2.5.1.141]	2834	2634	2610	2714	1595	1628	1539	1675	1489	1512	1339	1572
K02258 cytochrome c oxidase assembly protein subunit 11	819	864	905	885	450	447	414	486	280	316	276	261
K02259 cytochrome c oxidase assembly protein subunit 15	1687	1642	1668	1596	903	885	761	802	541	555	555	480
K02262 cytochrome c oxidase subunit 3	0	0	0	0	1	0	0	0	0	0	0	0
K02274 cytochrome c oxidase subunit I [EC:1.9.3.1]	2751	2830	2831	2868	1674	1633	1516	1621	1028	994	907	909
K02275 cytochrome c oxidase subunit II [EC:1.9.3.1]	1468	1566	1355	1422	842	777	760	861	545	460	434	477
K02276 cytochrome c oxidase subunit III [EC:1.9.3.1]	1618	1508	1462	1537	806	874	765	859	529	459	504	503
K02277 cytochrome c oxidase subunit IV [EC:1.9.3.1]	1	0	0	0	0	1	0	0	0	0	0	0
K02297 cytochrome o ubiquinol oxidase subunit II [EC:7.1.1.3]	1168	1118	1171	1138	730	721	740	782	976	1086	992	1022
K02298 cytochrome o ubiquinol oxidase subunit I [EC:7.1.1.3]	4065	3975	4072	4192	2380	2492	2400	2439	2624	2635	2616	2544
K02299 cytochrome o ubiquinol oxidase subunit III	1231	1290	1277	1318	910	803	705	787	844	858	780	840
K02300 cytochrome o ubiquinol oxidase subunit IV	510	518	534	542	288	363	352	334	356	401	347	375
K02826 cytochrome aa3-600 menaquinol oxidase subunit II [EC:7.1.1.5]	0	0	0	0	0	0	0	2	0	0	0	0
K02827 cytochrome aa3-600 menaquinol oxidase subunit I [EC:7.1.1.5]	1	0	0	0	0	0	0	0	0	0	1	0
K02828 cytochrome aa3-600 menaquinol oxidase subunit III [EC:7.1.1.5]	1	0	0	1	0	0	0	1	0	0	0	0
K02829 cytochrome aa3-600 menaquinol oxidase subunit IV [EC:7.1.1.5]	0	0	0	1	0	0	0	1	0	0	0	0
K03878 NADH-ubiquinone oxidoreductase chain 1 [EC:7.1.1.2]	0	0	2	0	2	0	0	0	0	0	0	0
K03881 NADH-ubiquinone oxidoreductase chain 4 [EC:7.1.1.2]	0	0	0	0	0	0	0	0	0	0	1	0
K03883 NADH-ubiquinone oxidoreductase chain 5 [EC:7.1.1.2]	0	0	0	0	0	2	3	0	2	1	0	0
K03885 NADH dehydrogenase [EC:1.6.99.3]	5318	5498	5154	5339	5243	5237	5092	5163	4225	4045	4222	4154
K03888 menaquinol-cytochrome c reductase cytochrome b/c subunit	2	0	1	0	0	0	0	0	0	0	0	1
K03934 NADH dehydrogenase (ubiquinone) Fe-S protein 1 [EC:7.1.1.2 1.6.99.3]	0	0	0	0	0	0	0	0	0	0	0	3
K05576 NAD(P)H-quinone oxidoreductase subunit 4L [EC:7.1.1.2]	0	0	0	0	1	1	0	0	0	1	0	1
K05577 NAD(P)H-quinone oxidoreductase subunit 5 [EC:7.1.1.2]	0	1	0	0	0	0	0	0	0	0	0	0
K05587 bidirectional [NiFe] hydrogenase diaphorase subunit [EC:7.1.1.2]	0	0	0	1	1	0	0	0	0	0	1	1
K05588 bidirectional [NiFe] hydrogenase diaphorase subunit [EC:7.1.1.2]	1	2	1	1	0	0	0	0	0	0	0	0
K06019 pyrophosphatase PpaX [EC:3.6.1.1]	23	31	19	22	6	3	2	0	37	41	52	42
K13378 NADH-quinone oxidoreductase subunit C/D [EC:7.1.1.2]	4220	4253	4392	4509	5441	5591	5568	5468	4731	4770	4478	4604
K15862 cytochrome c oxidase cbb3-type subunit I/II [EC:1.9.3.1]	2	0	1	0	1	0	2	0	1	4	2	1
K15986 manganese-dependent inorganic pyrophosphatase [EC:3.6.1.1]	1275	1326	1365	1326	707	765	793	718	907	1137	995	1072
K22468 polyphosphate kinase [EC:2.7.4.1]	2648	2550	2396	2627	1493	1630	1413	1538	1347	1302	1383	1352
K22501 cytochrome bd-II ubiquinol oxidase subunit AppX [EC:7.1.1.7]	0	2	0	0	3	0	0	3	4	3	2	4
ko00195 Photosynthesis	25078	25242	26008	25831	23893	24193	24023	23637	23559	23662	23890	23471
ko00710 Carbon fixation in photosynthetic organisms	70353	70765	71165	70998	72378	73942	73301	71665	77711	78355	77439	77675
K00855 phosphoribulokinase [EC:2.7.1.19]	539	523	533	525	354	428	394	378	657	756	667	764
K11214 sedoheptulokinase [EC:2.7.1.14]	1	0	2	2	0	0	0	0	7	3	0	4
K14454 aspartate aminotransferase, cytoplasmic [EC:2.6.1.1]	0	1	0	0	0	0	0	0	0	0	0	0
K14455 aspartate aminotransferase, mitochondrial [EC:2.6.1.1]	0	0	0	0	1	0	2	0	0	1	0	0
ko00720 Carbon fixation pathways in prokaryotes	143254	143346	143547	144074	155127	157183	156797	156520	153867	154439	154047	153805
K00194 acetyl-CoA decarbonylase/synthase, CODH/ACS complex subunit delta [EC:2.1.1.245]	24	36	29	54	19	31	29	36	90	111	90	114
K00196 anaerobic carbon-monoxide dehydrogenase iron sulfur subunit	87	78	68	86	37	41	119	128	64	68	76	76
K00197 acetyl-CoA decarbonylase/synthase, CODH/ACS complex subunit gamma [EC:2.1.1.245]	76	49	43	42	59	42	35	53	127	177	130	151
K00198 anaerobic carbon-monoxide dehydrogenase catalytic subunit [EC:1.2.7.4]	317	304	321	328	101	93	91	80	609	649	581	565
K00297 methylenetetrahydrofolate reductase (NADPH) [EC:1.5.1.20]	3363	3383	3519	3382	3530	3539	3691	3747	3378	3132	3176	3155
K01491 methylenetetrahydrofolate dehydrogenase (NADP+) / methenyltetrahydrofolate cyclohydrolase [EC:1.5.1.5 3.5.4.9]	2495	2524	2501	2444	2967	2892	2862	2850	2795	2679	2741	2709
K01938 formate--tetrahydrofolate ligase [EC:6.3.4.3]	1952	1977	2051	1957	3553	3585	3732	3511	3430	3406	3202	3300
K05299 formate dehydrogenase (NADP+) alpha subunit [EC:1.17.1.10]	9	9	11	8	4	6	6	8	16	19	13	13
K14138 acetyl-CoA synthase [EC:2.3.1.169]	101	83	87	102	52	52	56	48	228	288	230	258
K14470 2-methylfumaryl-CoA isomerase [EC:5.4.1.3]	0	0	0	1	0	0	0	0	1	0	1	0
K15016 enoyl-CoA hydratase / 3-hydroxyacyl-CoA dehydrogenase [EC:4.2.1.17 1.1.1.35]	5	2	2	1	1	1	1	0	2	5	6	6
K15019 3-hydroxypropionyl-coenzyme A dehydratase [EC:4.2.1.116]	0	2	2	7	0	3	2	1	1	3	2	1
K15020 acryloyl-coenzyme A reductase [EC:1.3.1.84]	0	0	0	0	0	0	0	1	0	0	0	0
K15022 formate dehydrogenase (NADP+) beta subunit [EC:1.17.1.10]	76	72	79	79	119	91	95	96	106	130	127	129
K15023 5-methyltetrahydrofolate corrinoid/iron sulfur protein methyltransferase [EC:2.1.1.258]	43	29	44	35	35	36	29	28	90	91	102	87
K15039 3-hydroxypropionate dehydrogenase (NADP+) [EC:1.1.1.298]	4	2	2	0	0	0	0	0	0	0	1	0
K18209 fumarate reductase (CoM/CoB) subunit A [EC:1.3.4.1]	34	34	26	39	3	9	5	9	63	54	39	60
K18210 fumarate reductase (CoM/CoB) subunit B [EC:1.3.4.1]	0	3	2	0	3	2	3	0	7	9	3	23
K18556 NADH-dependent fumarate reductase subunit A [EC:1.3.1.6]	0	0	0	0	0	0	0	1	0	0	0	0
K18559 NADH-dependent fumarate reductase subunit D	0	0	0	0	0	0	0	2	0	0	0	0
ko00680 Methane metabolism	103508	103110	102439	103040	99764	102035	100002	99858	104726	104681	103420	104820
K00058 D-3-phosphoglycerate dehydrogenase / 2-oxoglutarate reductase [EC:1.1.1.95 1.1.1.399]	7254	6707	6997	6996	5036	5169	4978	4913	7449	7651	7393	7411
K00125 formate dehydrogenase (coenzyme F420) beta subunit [EC:1.17.98.3 1.8.98.6]	0	2	0	5	0	0	2	0	13	10	13	12
K00148 glutathione-independent formaldehyde dehydrogenase [EC:1.2.1.46]	2130	2040	2041	2117	1154	1053	1063	1161	693	627	630	710
K00200 formylmethanofuran dehydrogenase subunit A [EC:1.2.7.12]	2	1	3	1	0	0	2	0	8	17	13	16
K00201 formylmethanofuran dehydrogenase subunit B [EC:1.2.7.12]	0	4	4	1	0	4	2	0	4	15	2	12
K00202 formylmethanofuran dehydrogenase subunit C [EC:1.2.7.12]	2	2	3	0	0	0	2	2	4	4	2	10
K00203 formylmethanofuran dehydrogenase subunit D [EC:1.2.7.12]	0	0	0	0	0	0	0	0	2	2	3	1
K00204 4Fe-4S ferredoxin	0	2	0	0	0	0	2	1	2	0	3	9
K00205 4Fe-4S ferredoxin	0	1	2	0	2	6	1	0	7	19	11	15
K00317 dimethylamine/trimethylamine dehydrogenase [EC:1.5.8.1 1.5.8.2]	2	2	4	2	2	1	3	2	0	7	5	7
K00319 methylenetetrahydromethanopterin dehydrogenase [EC:1.5.98.1]	0	2	2	1	0	0	0	0	1	5	3	7
K00320 5,10-methylenetetrahydromethanopterin reductase [EC:1.5.98.2]	0	0	0	1	1	0	0	0	7	12	2	4
K00399 methyl-coenzyme M reductase alpha subunit [EC:2.8.4.1]	0	2	2	0	5	0	0	0	4	12	2	11
K00400 methyl coenzyme M reductase system, component A2	3	5	0	3	4	11	0	2	13	25	13	21
K00401 methyl-coenzyme M reductase beta subunit [EC:2.8.4.1]	0	2	0	1	0	2	2	2	5	11	9	9
K00402 methyl-coenzyme M reductase gamma subunit [EC:2.8.4.1]	0	0	0	0	0	0	3	0	5	4	1	4
K00440 coenzyme F420 hydrogenase subunit alpha [EC:1.12.98.1]	3	0	2	1	0	0	0	3	4	12	12	2
K00441 coenzyme F420 hydrogenase subunit beta [EC:1.12.98.1]	3	0	0	1	2	1	1	2	8	21	12	24
K00442 coenzyme F420 hydrogenase subunit delta	0	0	0	0	0	0	0	0	2	2	0	1
K00443 coenzyme F420 hydrogenase subunit gamma [EC:1.12.98.1]	0	0	2	3	0	1	1	2	0	4	8	7
K00577 tetrahydromethanopterin S-methyltransferase subunit A [EC:2.1.1.86]	0	0	0	0	0	0	0	3	4	6	1	17
K00578 tetrahydromethanopterin S-methyltransferase subunit B [EC:2.1.1.86]	0	0	1	1	0	0	0	0	2	5	1	3
K00579 tetrahydromethanopterin S-methyltransferase subunit C [EC:2.1.1.86]	2	0	1	0	2	0	0	0	3	3	4	6
K00580 tetrahydromethanopterin S-methyltransferase subunit D [EC:2.1.1.86]	3	0	0	0	0	0	1	1	4	6	2	8
K00581 tetrahydromethanopterin S-methyltransferase subunit E [EC:2.1.1.86]	0	3	0	0	0	0	0	2	5	3	2	6

K00582 tetrahydromethanopterin S-methyltransferase subunit F [EC:2.1.1.86]	0	0	1	0	0	0	0	1	0	0	0	1
K00583 tetrahydromethanopterin S-methyltransferase subunit G [EC:2.1.1.86]	0	1	0	0	1	1	0	0	0	0	0	0
K00584 tetrahydromethanopterin S-methyltransferase subunit H [EC:2.1.1.86]	0	0	0	3	0	2	0	2	12	16	5	22
K00672 formylmethanofuran--tetrahydromethanopterin N-formyltransferase [EC:2.3.1.101]	3	2	1	0	2	2	0	2	4	16	6	12
K00831 phosphoserine aminotransferase [EC:2.6.1.52]	2303	2293	2385	2285	3216	3259	3311	3247	3226	3008	3200	3103
K01070 S-formylglutathione hydrolase [EC:3.1.2.12]	1734	1674	1659	1699	992	1073	941	996	1185	1169	1119	1169
K01079 phosphoserine phosphatase [EC:3.1.3.3]	2516	2498	2413	2455	3399	3604	3451	3294	2869	2831	2918	2902
K01499 methenyltetrahydromethanopterin cyclohydrolase [EC:3.5.4.27]	8	7	8	17	0	0	3	2	10	13	5	12
K02203 phosphoserine / homoserine phosphotransferase [EC:3.1.3.3 2.7.1.39]	866	946	883	906	523	449	510	428	274	244	309	263
K03388 heterodisulfide reductase subunit A2 [EC:1.8.7.3 1.8.98.4 1.8.98.5 1.8.98.6]	104	119	124	150	53	38	39	35	244	265	193	247
K03389 heterodisulfide reductase subunit B2 [EC:1.8.7.3 1.8.98.4 1.8.98.5 1.8.98.6]	57	49	38	61	44	29	29	38	108	100	104	107
K03390 heterodisulfide reductase subunit C2 [EC:1.8.7.3 1.8.98.4 1.8.98.5 1.8.98.6]	34	19	21	31	18	21	17	21	56	70	61	61
K03421 methyl-coenzyme M reductase subunit C	0	0	0	0	2	0	0	2	2	4	8	6
K03422 methyl-coenzyme M reductase subunit D	0	0	0	0	0	0	0	2	0	8	0	3
K03532 trimethylamine-N-oxide reductase (cytochrome c), cytochrome c-type subunit TorC	14	26	26	31	38	46	30	35	103	82	101	84
K03533 TorA specific chaperone	4	9	2	3	8	6	5	10	47	36	51	28
K04480 methanol---5-hydroxybenzimidazolylcobamide Co-methyltransferase [EC:2.1.1.90]	10	7	25	19	24	32	18	14	47	50	46	73
K05884 L-2-hydroxycarboxylate dehydrogenase (NAD+) [EC:1.1.1.337]	36	47	30	28	6	2	2	1	87	90	88	106
K05979 2-phosphosulfolactate phosphatase [EC:3.1.3.71]	13	6	12	21	1	5	1	2	15	27	24	26
K06034 sulfopyruvate decarboxylase subunit alpha [EC:4.1.1.79]	0	0	0	0	0	0	2	0	4	2	5	9
K06914 tyramine---L-glutamate ligase [EC:6.3.4.24]	0	0	0	0	0	1	0	0	2	9	3	7
K07072 (4-(4-[2-(gamma-L-glutamylamino)ethyl]phenoxymethyl)furan-2-yl)methanamine synthase [EC:2.5.1.131]	2	0	0	0	0	5	0	2	3	5	2	6
K07144 5-(aminomethyl)-3-furanmethanol phosphate kinase [EC:2.7.4.31]	0	0	0	2	0	0	0	0	4	2	3	4
K07811 trimethylamine-N-oxide reductase (cytochrome c) [EC:1.7.2.3]	9	11	15	9	38	48	19	49	121	155	166	150
K07812 trimethylamine-N-oxide reductase (cytochrome c) [EC:1.7.2.3]	251	262	291	207	443	446	453	484	524	470	464	507
K07821 trimethylamine-N-oxide reductase (cytochrome c), cytochrome c-type subunit TorY	2	8	2	3	12	14	17	12	52	43	37	42
K08097 phosphosulfolactate synthase [EC:4.4.1.19]	0	0	0	0	0	0	0	0	2	6	2	4
K08264 heterodisulfide reductase subunit D [EC:1.8.98.1]	3	2	0	3	2	0	3	0	4	9	1	8
K08685 quinohemoprotein amine dehydrogenase [EC:1.4.9.1]	1	0	0	2	0	0	0	0	0	0	0	0
K09733 (5-formylfuran-3-yl)methyl phosphate synthase [EC:4.2.3.153]	2	3	0	0	0	0	2	0	0	9	3	4
K10978 methanogen homoisocitrate dehydrogenase [EC:1.1.1.87 1.1.1.-]	0	0	0	0	2	4	0	0	0	9	8	6
K11212 LPPG:FO 2-phospho-L-lactate transferase [EC:2.7.8.28]	0	1	5	0	2	4	2	0	8	4	4	1
K11260 4Fe-4S ferredoxin	0	0	1	0	0	0	2	0	0	1	1	1
K11261 formylmethanofuran dehydrogenase subunit E [EC:1.2.7.12]	127	123	125	149	54	45	47	52	211	259	234	247
K11780 7,8-didemethyl-8-hydroxy-5-deazariboflavin synthase [EC:4.3.1.32]	0	0	2	0	3	0	4	2	4	6	4	3
K11781 5-amino-6-(D-ribitylamino)uracil---L-tyrosine 4-hydroxyphenyl transferase [EC:2.5.1.147]	3	3	0	0	7	0	0	0	5	13	3	8
K12234 coenzyme F420-0:L-glutamate ligase / coenzyme F420-1:gamma-L-glutamate ligase [EC:6.3.2.31 6.3.2.34]	0	1	0	0	3	0	3	0	1	2	1	9
K13039 sulfopyruvate decarboxylase subunit beta [EC:4.1.1.79]	0	0	0	1	0	0	0	0	2	9	3	1
K13942 5,10-methenyltetrahydromethanopterin hydrogenase [EC:1.12.98.2]	2	2	0	0	3	0	0	2	0	6	3	11
K14080 [methyl-Co(III) methanol-specific corrinoid protein]:coenzyme M methyltransferase [EC:2.1.1.246]	8	20	11	9	11	10	8	4	16	24	21	31
K14081 methanol corrinoid protein	0	0	1	0	0	2	2	5	4	9	3	9
K14083 trimethylamine---corrinoid protein Co-methyltransferase [EC:2.1.1.250]	822	808	820	875	723	693	718	659	1915	2087	1829	2205
K14084 trimethylamine corrinoid protein	2	2	1	6	0	0	2	0	1	2	2	2
K14126 F420-non-reducing hydrogenase large subunit [EC:1.12.99.- 1.8.98.5]	3	6	0	3	0	4	5	4	4	2	0	28
K14127 F420-non-reducing hydrogenase iron-sulfur subunit [EC:1.12.99.- 1.8.98.5 1.8.98.6]	0	0	0	0	0	1	0	0	6	4	0	4
K14128 F420-non-reducing hydrogenase small subunit [EC:1.12.99.- 1.8.98.5]	0	0	1	0	0	0	2	1	2	8	3	20
K14941 2-phospho-L-lactate guanylyltransferase [EC:2.7.7.68]	2	0	1	1	0	0	0	0	2	1	3	7
K16176 methylamine---corrinoid protein Co-methyltransferase [EC:2.1.1.248]	10	12	12	8	3	5	2	2	5	4	9	4
K16178 dimethylamine---corrinoid protein Co-methyltransferase [EC:2.1.1.249]	0	0	0	0	0	0	0	0	0	0	0	1
K16179 dimethylamine corrinoid protein	0	0	0	1	0	0	1	0	0	0	0	1
K16792 methanogen homoaconitase large subunit [EC:4.2.1.114]	1	0	4	4	1	3	3	0	4	7	9	12
K16793 methanogen homoaconitase small subunit [EC:4.2.1.114]	1	0	0	0	0	0	1	0	1	2	1	5
K18933 tyrosine decarboxylase / aspartate 1-decarboxylase [EC:4.1.1.25 4.1.1.11]	0	0	0	3	2	1	2	2	3	0	8	5
K19793 (5-formylfuran-3-yl)methyl phosphate transaminase [EC:2.6.1.108]	0	0	1	0	0	1	0	0	0	0	0	0
K22081 methylamine---glutamate N-methyltransferase subunit A [EC:2.1.1.21]	0	0	0	0	0	0	0	2	0	0	0	0
K22083 methylamine---glutamate N-methyltransferase subunit C [EC:2.1.1.21]	1	0	0	0	0	0	0	3	0	0	0	0
K22084 methylglutamate dehydrogenase subunit A [EC:1.5.99.5]	0	0	0	1	0	1	0	0	0	1	0	0
K22305 phosphoserine phosphatase [EC:3.1.3.3]	4	2	3	3	3	1	2	3	6	7	10	11
K22480 heterodisulfide reductase subunit A1 [EC:1.8.7.3]	8	3	3	1	0	0	2	0	13	15	3	15
K22481 heterodisulfide reductase subunit B1 [EC:1.8.7.3]	2	0	0	1	0	1	0	2	6	7	3	10
K22482 heterodisulfide reductase subunit C1 [EC:1.8.7.3]	0	1	0	1	0	1	0	0	3	4	0	1
K22516 formate dehydrogenase (coenzyme F420) alpha subunit [EC:1.17.98.3 1.8.98.6]	36	26	20	31	14	8	19	9	22	35	29	26
ko00910 Nitrogen metabolism	120034	119552	117160	118852	102402	101623	100410	101796	98543	97125	97001	97654
K00260 glutamate dehydrogenase [EC:1.4.1.2]	59	72	65	44	29	27	25	23	92	109	114	93
K00261 glutamate dehydrogenase (NAD(P)+) [EC:1.4.1.3]	584	540	564	646	147	173	164	144	1125	1169	1191	1171
K00262 glutamate dehydrogenase (NADP+) [EC:1.4.1.4]	4671	4827	5022	4918	7000	7211	7056	7039	6549	6261	6492	6096
K00264 glutamate synthase (NADH) [EC:1.4.1.14]	8	14	19	11	11	10	19	9	22	11	21	14
K00265 glutamate synthase (NADPH) large chain [EC:1.4.1.13]	14239	14566	14524	14318	15161	14791	14306	14093	14526	14253	14248	14084
K00266 glutamate synthase (NADPH) small chain [EC:1.4.1.13]	7387	7375	7496	7262	9781	9848	10089	9830	9306	9188	9350	9228
K00360 assimilatory nitrate reductase electron transfer subunit [EC:1.7.99.-]	3	0	0	0	0	1	0	0	0	0	0	0
K00362 nitrite reductase (NADH) large subunit [EC:1.7.1.15]	7321	7187	6973	7380	4370	4329	4308	4460	6239	6149	6048	6145
K00363 nitrite reductase (NADH) small subunit [EC:1.7.1.15]	730	770	719	766	457	492	452	427	517	526	448	476
K00366 ferredoxin-nitrite reductase [EC:1.7.7.1]	30	42	26	40	12	3	11	5	19	26	24	29
K00368 nitrite reductase (NO-forming) [EC:1.7.2.1]	0	0	0	2	1	2	1	2	4	16	3	5
K00370 nitrate reductase / nitrite oxidoreductase, alpha subunit [EC:1.7.5.1 1.7.99.-]	9684	9631	9278	9660	5346	5224	4896	5196	7670	7529	7284	7779
K00371 nitrate reductase / nitrite oxidoreductase, beta subunit [EC:1.7.5.1 1.7.99.-]	2332	2280	2264	2393	1238	1296	1185	1290	2727	2691	2562	2621
K00372 assimilatory nitrate reductase catalytic subunit [EC:1.7.99.-]	5354	5390	5194	5361	3092	3005	2854	2930	3085	3080	2888	3275
K00374 nitrate reductase gamma subunit [EC:1.7.5.1 1.7.99.-]	1527	1484	1539	1529	920	870	839	857	1255	1177	1231	1213
K00376 nitrous-oxide reductase [EC:1.7.2.4]	1875	1864	1726	1846	980	1035	970	1056	578	579	588	540
K00459 nitronate monooxygenase [EC:1.13.12.16]	5347	5300	5010	5282	2775	2694	2695	2877	2322	2312	2341	2508
K00531 nitrogenase delta subunit [EC:1.18.6.1]	3	0	2	1	1	1	0	4	2	5	0	2
K00926 carbamate kinase [EC:2.7.2.2]	1632	1635	1708	1774	978	970	941	925	1466	1519	1470	1404
K01501 nitrilase [EC:3.5.5.1]	21	22	22	21	17	12	5	10	39	45	44	36
K01673 carbonic anhydrase [EC:4.2.1.1]	4600	4718	4518	4496	3490	3383	3441	3485	3333	3341	3278	3471
K01674 carbonic anhydrase [EC:4.2.1.1]	25	27	33	33	14	5	6	10	31	50	41	21
K01725 cyanate lyase [EC:4.2.1.104]	420	419	364	384	208	246	210	255	110	111	107	136
K01948 carbamoyl-phosphate synthase (ammonia) [EC:6.3.4.16]	1	0	0	0	0	1	0	0	0	0	0	0
K02305 nitric oxide reductase subunit C	682	676	629	675	332	371	336	389	241	209	215	189
K02567 periplasmic nitrate reductase NapA [EC:1.7.99.-]	3951	4109	3678	3776	2245	2189	2197	2421	1612	1601	1529	1539
K02568 cytochrome c-type protein NapB	659	618	619	583	323	356	339	319	220	219	210	214
K02575 MFS transporter, NNP family, nitrate/nitrite transporter	5611	5652	5390	5528	2960	3034	2762	2960	3469	3354	3279	3647
K02586 nitrogenase molybdenum-iron protein alpha chain [EC:1.18.6.1]	336	316	283	348	347	427	416	424	78	100	101	95
K02588 nitrogenase iron protein NifH	289	262	266	309	316	344	334	326	137	183	169	163
K02591 nitrogenase molybdenum-iron protein beta chain [EC:1.18.6.1]	315	315	305	292	471	456	447	438	18	42	27	41
K03385 nitrite reductase (cytochrome c-552) [EC:1.7.2.2]	1591	1725	1843	1715	5713	5810	5969	5551	4412	4137	4603	4216
K04561 nitric oxide reductase subunit B [EC:1.7.2.5]	2180	2103	1969	2058	1250	1118	1042	1223	724	676	616	701
K05601 hydroxylamine reductase [EC:1.7.99.1]	2761	2650	2685	2897	4045	4002	4128	3953	4184	4047	4092	4162
K15371 glutamate dehydrogenase [EC:1.4.1.2]	7394	7227	6792	7016	3804	3820	3548	3904	2316	2180	2149	2180
K15576 nitrate/nitrite transport system substrate-binding protein	777	655	646	707	419	412	419	450	1017	968	973	1050
K15577 nitrate/nitrite transport system permease protein	384	438	418	415	287	277	321	337	510	594	591	600
K15578 nitrate/nitrite transport system ATP-binding protein [EC:3.6.3.-]	477	442	394	445	284	358	304	264	520	557	553	611
K15864 nitrite reductase (NO-forming) / hydroxylamine reductase [EC:1.7.2.1 1.7.99.1]	2360	2254	2132	2155	1234	1232	1176	1326	759	712	630	750
K15876 cytochrome c nitrite reductase small subunit	290	319	369	298	986	1063	1062	1037	790	815	784	697
ko00920 Sulfur metabolism	96207	96252	92776	95736	67620	67770	65884	68472	68196	68496	67501	68763
K00299 FMN reductase [EC:1.5.1.38]	1645	1659	1532	1701	898	914	843	982	789	875	765	915
K00380 sulfite reductase (NADPH) flavoprotein alpha-component [EC:1.8.1.2]	5044	4900	4798	4852	2923	2808	2932	2771	2580	2662	2748	2818
K00381 sulfite reductase (NADPH) hemoprotein beta-component [EC:1.8.1.2]	6118	6141	5914	6135	3497	3591	3390	3681	3065	2912	2792	2922
K00385 anaerobic sulfite reductase subunit C	563	491	552	542	98	120	141	113	436	463	521	475
K00390 phosphoadenosine phosphosulfate reductase [EC:1.8.4.8 1.8.4.10]	1562	1515	1571	1588	575	610	560	570	1002	1192	1134	1214
K00394 adenylylsulfate reductase, subunit A [EC:1.8.99.2]	24	14	16	14	5	9	7	6	21	36	26	29
K00395 adenylylsulfate reductase, subunit B [EC:1.8.99.2]	4	13	2	9	9	11	5	9	3	9	0	4
K00640 serine O-acetyltransferase [EC:2.3.1.30]	3960	3994	3982	4081	4079	4016	4022	3925	3366	3741	3521	3468
K00641 homoserine O-acetyltransferase/O-succinyltransferase [EC:2.3.1.31 2.3.1.46]	2008	1968	1997	1939	1195	1153	1046	1201	703	632	644	623
K00651 homoserine O-succinyltransferase/O-acetyltransferase [EC:2.3.1.46 2.3.1.31]	1767	1758	1818	1816	2681	2773	2688	2704	2670	2718	2753	2761
K00860 adenylylsulfate kinase [EC:2.7.1.25]	725	772	758	778	1408	1355	1567	1466	1322	1429	1381	1414
K00955 bifunctional enzyme CysN/CysC [EC:2.7.7.4 2.7.1.25]	2782	2672	2860	2862	1714	1659	1620	1664	991	876	916	840
K00956 sulfate adenylyltransferase subunit 1 [EC:2.7.7.4]	1789	1797	1856	1898	3663	3894	3870	3734	3343	3321	3303	3223
K00957 sulfate adenylyltransferase subunit 2 [EC:2.7.7.4]	1611	1619	1611	1618	2522	2665	2560	2458	2268	2038	2169	2153
K00958 sulfate adenylyltransferase [EC:2.7.7.4]	5	9	9	7	2	4	3	3	9	14	8	22
K01011 thiosulfate/3-mercaptopyruvate sulfurtransferase [EC:2.8.1.1 2.8.1.2]	2402	2361	2238	2334	1421	1280	1297	1442	1247	1241	1223	1251
K01082 3'(2'), 5'-bisphosphate nucleotidase [EC:3.1.3.7]	2530	2409	2464	2419	2850	2841	2836	2810	2174	2084	2295	2239

K01738 cysteine synthase [EC:2.5.1.47]	5828	5923	5827	5924	6532	6628	6576	6497	6452	6559	6224	6254
K01739 cystathionine gamma-synthase [EC:2.5.1.48]	774	733	744	871	557	639	570	495	1056	1074	1219	1158
K02045 sulfate/thiosulfate transport system ATP-binding protein [EC:7.3.2.3]	2029	1857	1833	1972	1270	1194	1245	1187	1482	1409	1431	1375
K02046 sulfate/thiosulfate transport system permease protein	1860	1919	1712	1923	1118	1130	1126	1156	1145	1123	1214	1115
K02047 sulfate/thiosulfate transport system permease protein	1419	1475	1404	1437	902	884	909	929	1071	1095	1007	1031
K02048 sulfate/thiosulfate transport system substrate-binding protein	1531	1579	1521	1574	1018	978	870	1008	1170	1180	1148	1151
K02439 thiosulfate sulfurtransferase [EC:2.8.1.1]	727	741	741	780	497	496	417	473	421	449	517	507
K03119 taurine dioxygenase [EC:1.14.11.17]	2826	2871	2720	2625	1552	1438	1320	1472	1485	1438	1385	1494
K04091 alkanesulfonate monooxygenase [EC:1.14.14.5]	6864	6964	6398	6640	3271	3206	2913	3432	4432	4178	4191	4399
K05907 adenylyl-sulfate reductase (glutathione) [EC:1.8.4.9]	0	1	0	0	0	0	0	0	0	0	0	0
K06881 bifunctional oligoribonuclease and PAP phosphatase NrnA [EC:3.1.3.7 3.1.13.3]	1176	1280	1336	1269	2344	2524	2387	2379	2009	2022	2079	2008
K07306 anaerobic dimethyl sulfoxide reductase subunit A [EC:1.8.5.3]	1341	1417	1470	1467	1024	1062	1010	974	1886	2099	1944	2014
K07307 anaerobic dimethyl sulfoxide reductase subunit B	709	712	628	621	426	482	495	509	909	1046	959	1030
K07308 anaerobic dimethyl sulfoxide reductase subunit C	505	551	481	557	337	363	373	287	748	812	706	767
K08352 thiosulfate reductase / polysulfide reductase chain A [EC:1.8.5.5]	71	62	52	48	36	34	22	36	95	68	84	105
K08353 thiosulfate reductase electron transport protein	3	0	0	1	0	2	0	0	0	0	0	0
K08354 thiosulfate reductase cytochrome b subunit	4	2	5	1	7	22	8	14	40	53	34	33
K08357 tetrathionate reductase subunit A	14	22	8	20	4	17	5	14	14	32	27	45
K08358 tetrathionate reductase subunit B	4	0	4	3	5	0	0	0	10	12	1	4
K08359 tetrathionate reductase subunit C	0	0	1	1	2	2	2	2	1	6	3	2
K10764 O-succinylhomoserine sulfhydrylase [EC:2.5.1.-]	1782	1803	1799	1710	900	953	926	1075	592	547	536	528
K10831 taurine transport system ATP-binding protein [EC:7.6.2.7]	1730	1629	1524	1597	743	805	720	770	1103	950	966	1068
K11180 dissimilatory sulfite reductase alpha subunit [EC:1.8.99.5]	8	21	15	13	6	5	5	4	11	16	9	7
K11181 dissimilatory sulfite reductase beta subunit [EC:1.8.99.5]	16	5	19	6	4	3	6	7	4	35	9	15
K15551 taurine transport system substrate-binding protein	1978	1940	1804	1930	1142	1208	1080	1150	1166	1223	1114	1285
K15552 taurine transport system permease protein	1927	1906	1773	1774	1065	1098	1007	1061	1207	1220	1226	1221
K15553 sulfonate transport system substrate-binding protein	9591	9871	9258	9576	4932	4712	4690	5068	5048	4838	4846	5025
K15554 sulfonate transport system permease protein	4942	4799	4470	4804	2505	2319	2292	2630	2186	2104	1983	2178
K15555 sulfonate transport system ATP-binding protein [EC:3.6.3.-]	4238	4267	3970	4307	2165	2222	1919	2348	1861	1976	1874	1853
K16937 thiosulfate dehydrogenase [quinone] large subunit [EC:1.8.5.2]	5	4	7	6	0	0	2	0	1	2	0	1
K16950 anaerobic sulfite reductase subunit A	480	399	490	510	100	108	101	129	363	445	427	427
K16951 anaerobic sulfite reductase subunit B	376	385	455	411	88	93	103	76	309	371	326	332
K17069 O-acetylhomoserine/O-acetylserine sulfhydrylase [EC:2.5.1.49 2.5.1.47]	0	2	0	0	0	0	0	1	0	0	0	0
K17217 cystathionine gamma-lyase / homocysteine desulfhydrase [EC:4.4.1.1 4.4.1.2]	0	0	1	0	0	0	0	0	0	0	1	1
K17218 sulfide:quinone oxidoreductase [EC:1.8.5.4]	3466	3442	3097	3222	1692	1618	1590	1787	1106	1004	960	1028
K17222 L-cysteine S-thiosulfotransferase [EC:2.8.5.2]	0	0	0	0	0	0	0	0	2	0	0	0
K17226 sulfur-oxidizing protein SoxY	2	0	0	0	0	0	0	0	0	0	0	0
K17228 dimethylsulfone monooxygenase [EC:1.14.14.35]	536	566	474	540	268	293	259	332	186	165	169	162
K20034 3-(methylthio)propionyl---CoA ligase [EC:6.2.1.44]	597	593	554	653	170	156	156	151	1277	1254	1263	1289
K20035 3-(methylthio)propanoyl-CoA dehydrogenase [EC:1.3.8.-]	2	0	1	1	0	0	0	0	1	1	1	0
K21307 sulfite dehydrogenase (quinone) subunit SoeA [EC:1.8.5.6]	10	1	2	9	2	1	3	3	4	11	13	11
K21308 sulfite dehydrogenase (quinone) subunit SoeB	1	0	0	0	0	0	0	0	0	0	0	0
K23163 sulfate/thiosulfate transport system substrate-binding protein	2296	2418	2200	2339	1391	1371	1387	1477	1354	1436	1406	1468
K23304 serine O-acetyltransferase [EC:2.3.1.30]	0	0	0	1	5	1	3	0	0	0	0	1
Lipid metabolism	309133	311310	306979	308645	306871	305504	305084	306841	292771	289500	288010	289817
ko00061 Fatty acid biosynthesis	66452	66872	66378	66508	70556	69105	70239	70803	62022	61518	61559	61627
K00059 3-oxoacyl-[acyl-carrier protein] reductase [EC:1.1.1.100]	13605	13158	13121	13004	9680	9267	9323	9691	8537	8520	8456	8451
K00208 enoyl-[acyl-carrier protein] reductase I [EC:1.3.1.9 1.3.1.10]	1438	1495	1521	1533	2368	2390	2378	2253	2194	2014	2126	2133
K00645 [acyl-carrier-protein] S-malonyltransferase [EC:2.3.1.39]	3126	3021	3156	3243	2939	2989	2957	2925	3173	3232	3216	3225
K00647 3-oxoacyl-[acyl-carrier-protein] synthase I [EC:2.3.1.41]	4745	4733	4459	4601	3014	2927	2781	3133	2225	2219	2169	2295
K00648 3-oxoacyl-[acyl-carrier-protein] synthase III [EC:2.3.1.180]	3715	4111	4025	3925	5668	5615	5633	5569	4836	4801	4796	4751
K00667 fatty acid synthase subunit alpha, fungi type [EC:2.3.1.86]	0	2	0	0	0	0	0	0	0	0	0	0
K01071 medium-chain acyl-[acyl-carrier-protein] hydrolase [EC:3.1.2.21]	65	58	52	57	24	20	30	21	53	90	50	57
K01716 3-hydroxyacyl-[acyl-carrier protein] dehydratase / trans-2-decenoyl-[acyl-carrier protein] isomerase [EC:4.2.1.59 5.3.3.14]	750	917	808	819	537	511	492	553	599	602	634	596
K01897 long-chain acyl-CoA synthetase [EC:6.2.1.3]	8770	9406	9278	9093	17228	16696	17565	17395	13676	13087	13358	13238
K02078 acyl carrier protein	1597	1533	1518	1673	1524	1501	1511	1628	1542	1495	1572	1570
K02371 enoyl-[acyl-carrier protein] reductase II [EC:1.3.1.9]	1555	1614	1626	1652	2122	2055	2065	2065	2538	2576	2560	2511
K02372 3-hydroxyacyl-[acyl-carrier-protein] dehydratase [EC:4.2.1.59]	1529	1441	1523	1471	819	796	844	837	1074	1132	1061	1174
K03921 acyl-[acyl-carrier-protein] desaturase [EC:1.14.19.2 1.14.19.11 1.14.19.26]	0	2	0	0	0	0	0	0	0	0	2	0
K09458 3-oxoacyl-[acyl-carrier-protein] synthase II [EC:2.3.1.179]	7077	6999	7052	7012	6574	6571	6635	6363	6355	6449	6461	6317
K10780 enoyl-[acyl-carrier protein] reductase III [EC:1.3.1.104]	2	2	1	1	0	0	1	0	3	1	3	1
K11533 fatty acid synthase, bacteria type [EC:2.3.1.-]	11	8	7	15	8	10	12	14	39	49	53	101
K13370 17beta-estradiol 17-dehydrogenase / 3alpha(17beta)-hydroxysteroid dehydrogenase (NAD+) / 3-oxoacyl-[acyl-carrier protein] reductase alpha subuni	1	1	0	1	0	1	0	0	0	0	0	0
K16363 UDP-3-O-[3-hydroxymyristoyl] N-acetylglucosamine deacetylase / 3-hydroxyacyl-[acyl-carrier-protein] dehydratase [EC:3.5.1.108 4.2.1.59]	974	927	938	919	2968	3011	2989	2986	2053	1991	2036	1939
K18473 acetoacetyl-[acyl-carrier protein] synthase [EC:2.3.1.180]	3589	3386	3346	3566	2120	1860	1943	2037	1148	1092	1056	1157
K18474 trans-2-decenoyl-[acyl-carrier protein] isomerase [EC:5.3.3.14]	3	7	10	5	0	0	2	0	0	0	2	3
K18660 malonyl-CoA/methylmalonyl-CoA synthetase [EC:6.2.1.-]	0	0	0	0	0	1	0	0	0	0	0	0
K22540 3-hydroxyacyl-thioester dehydratase, animal type [EC:4.2.1.-]	0	0	1	1	0	0	0	0	0	0	0	0
ko00062 Fatty acid elongation	5	0	4	1	2	0	0	3	1	0	0	0
K01068 acyl-coenzyme A thioesterase 1/2/4 [EC:3.1.2.2]	0	0	0	0	2	0	0	0	0	0	0	0
K01074 palmitoyl-protein thioesterase [EC:3.1.2.22]	0	0	1	0	0	0	0	0	0	0	0	0
K07508 acetyl-CoA acyltransferase 2 [EC:2.3.1.16]	1	0	1	0	0	0	0	2	0	0	0	0
ko00071 Fatty acid degradation	83216	82836	80666	81489	59791	58151	57555	59765	53800	53036	52240	53654
K00249 acyl-CoA dehydrogenase [EC:1.3.8.7]	12040	11683	11576	11551	6589	6315	6025	6736	3910	3629	3652	3657
K00252 glutaryl-CoA dehydrogenase [EC:1.3.8.6]	1884	1899	1710	1822	1064	957	991	992	606	580	507	596
K00496 alkane 1-monooxygenase [EC:1.14.15.3]	3112	2999	3072	3017	1802	1663	1528	1644	984	1003	897	988
K00529 3-phenylpropionate/trans-cinnamate dioxygenase ferredoxin reductase component [EC:1.18.1.3]	127	149	118	99	20	5	13	8	4	7	5	5
K00632 acetyl-CoA acyltransferase [EC:2.3.1.16]	6771	6698	6402	6636	4031	3892	3923	3925	3815	3724	3658	3839
K01909 long-chain-fatty-acid--[acyl-carrier-protein] ligase [EC:6.2.1.20]	0	0	1	1	0	0	0	0	0	0	0	1
K05297 rubredoxin---NAD+ reductase [EC:1.18.1.1]	2218	2122	2033	1965	1193	1119	1081	1201	704	613	622	710
K05939 acyl-[acyl-carrier-protein]-phospholipid O-acyltransferase / long-chain-fatty-acid--[acyl-carrier-protein] ligase [EC:2.3.1.40 6.2.1.20]	1432	1359	1316	1323	896	1075	946	966	1758	1863	1890	1888
K06445 acyl-CoA dehydrogenase [EC:1.3.99.-]	5037	4873	4680	4861	2879	2925	2805	2925	3217	3266	3076	3375
K09478 short/branched chain acyl-CoA dehydrogenase [EC:1.3.99.12]	6	2	5	1	1	0	2	1	4	7	8	3
K09479 very long chain acyl-CoA dehydrogenase [EC:1.3.8.9]	0	1	0	1	1	1	3	1	2	1	0	0
K13239 Delta3-Delta2-enoyl-CoA isomerase [EC:5.3.3.8]	1	0	0	0	0	0	0	0	0	0	0	0
K13767 enoyl-CoA hydratase [EC:4.2.1.17]	0	1	0	0	0	0	0	0	0	1	0	0
ko00072 Synthesis and degradation of ketone bodies	16578	16749	15589	16376	9086	8818	8510	9209	9181	9493	9076	9436
ko00100 Steroid biosynthesis	0	1	0	2	0	1	0	0	2	1	3	8
K12298 bile salt-stimulated lipase [EC:3.1.1.3 3.1.1.13]	0	1	0	2	0	1	0	0	2	1	3	8
ko00120 Primary bile acid biosynthesis	2512	2686	2823	2640	6374	6506	6651	6256	5338	5106	5378	5153
K01442 choloylglycine hydrolase [EC:3.5.1.24]	2512	2685	2822	2640	6374	6506	6651	6256	5338	5105	5377	5153
K01796 alpha-methylacyl-CoA racemase [EC:5.1.99.4]	0	1	1	0	0	0	0	0	0	1	1	0
ko00121 Secondary bile acid biosynthesis	2784	2957	3101	2906	6442	6575	6715	6334	5557	5342	5614	5384
K00076 7-alpha-hydroxysteroid dehydrogenase [EC:1.1.1.159]	5	0	3	3	3	4	8	15	30	40	36	36
K15868 bile acid-coenzyme A ligase [EC:6.2.1.7]	15	7	2	5	5	5	7	12	24	21	32	23
K22604 3alpha-hydroxycholanate dehydrogenase (NAD+) [EC:1.1.1.52]	2	0	3	3	1	1	0	0	0	0	1	0
K22605 3alpha-hydroxycholanate dehydrogenase (NADP+) [EC:1.1.1.392]	0	0	1	0	0	0	1	0	1	0	1	1
K22606 3beta-hydroxycholanate 3-dehydrogenase (NAD+) [EC:1.1.1.391]	250	265	270	255	59	59	48	51	164	176	167	171
ko00140 Steroid hormone biosynthesis	6731	7112	6987	6939	13699	13841	14055	13839	12228	11460	11859	11357
K00038 3alpha(or 20beta)-hydroxysteroid dehydrogenase [EC:1.1.1.53]	8	17	23	9	1	0	3	0	11	6	8	18
K01130 arylsulfatase [EC:3.1.6.1]	6304	6570	6426	6461	12061	12191	12306	12246	11033	10408	10703	10185
K01131 steryl-sulfatase [EC:3.1.6.2]	0	0	0	0	0	0	0	0	1	10	4	7	7
K05296 3(or 17)beta-hydroxysteroid dehydrogenase [EC:1.1.1.51]	1	0	0	1	0	0	0	0	1	0	1	1
K12343 3-oxo-5-alpha-steroid 4-dehydrogenase 1 [EC:1.3.1.22]	417	524	538	467	1637	1648	1746	1592	1173	1042	1140	1146
K15680 corticosteroid 11-beta-dehydrogenase isozyme 1 [EC:1.1.1.146]	0	0	0	0	0	1	0	0	0	0	0	0
ko00561 Glycerolipid metabolism	55557	55846	54836	55553	43485	43921	43349	43586	56522	57228	55353	56920
K00631 glycerol-3-phosphate O-acyltransferase [EC:2.3.1.15]	5380	5546	5306	5526	3300	3295	3243	3293	3417	3501	3446	3469
K00655 1-acyl-sn-glycerol-3-phosphate acyltransferase [EC:2.3.1.51]	3000	2920	3125	2866	2710	2794	2705	2866	2810	2835	2743	2793
K00864 glycerol kinase [EC:2.7.1.30]	9915	9952	9554	9759	5312	5490	5238	5655	6381	6677	6150	6601
K00901 diacylglycerol kinase (ATP) [EC:2.7.1.107]	908	784	836	879	567	572	545	542	537	527	451	557
K01046 triacylglycerol lipase [EC:3.1.1.3]	2846	2685	2656	2739	1445	1388	1389	1448	986	881	817	915
K01054 acylglycerol lipase [EC:3.1.1.23]	1	0	0	1	0	1	0	1	0	0	0	1
K03429 processive 1,2-diacylglycerol beta-glucosyltransferase [EC:2.4.1.315]	208	204	187	182	72	83	47	64	220	278	276	246
K03621 phosphate acyltransferase [EC:2.3.1.274]	2248	2183	2244	2195	1169	1108	1233	1215	1595	1681	1531	1654
K05878 phosphoenolpyruvate---glycerone phosphotransferase subunit DhaK [EC:2.7.1.121]	1017	991	957	1107	653	644	639	585	1679	1702	1597	1680
K05879 phosphoenolpyruvate---glycerone phosphotransferase subunit DhaL [EC:2.7.1.121]	687	632	560	605	356	390	390	374	913	867	854	918
K05881 phosphoenolpyruvate---glycerone phosphotransferase subunit DhaM [EC:2.7.1.121]	890	966	907	1002	586	611	553	594	1311	1258	1326	1362
K06117 glycerol-1-phosphatase [EC:3.1.3.21]	1	4	0	3	0	5	5	3	12	10	8	11
K06119 sulfoquinovosyltransferase [EC:2.4.1.-]	0	0	0	0	0	0	0	0	0	1	1	1
K06120 glycerol dehydratase large subunit [EC:4.2.1.30]	661	741	600	728	193	190	186	223	1333	1405	1329	1349
K06121 glycerol dehydratase medium subunit [EC:4.2.1.30]	244	251	298	285	60	70	64	60	510	502	543	555

K06122 glycerol dehydratase small subunit [EC:4.2.1.30]	176	177	213	184	66	47	47	52	389	357	292	337
K07029 diacylglycerol kinase (ATP) [EC:2.7.1.107]	59	62	43	72	15	28	24	17	43	102	66	76
K08591 acyl phosphate:glycerol-3-phosphate acyltransferase [EC:2.3.1.275]	1652	1663	1529	1593	919	867	870	897	1300	1325	1257	1283
K13677 1,2-diacylglycerol-3-alpha-glucose alpha-1,2-glucosyltransferase [EC:2.4.1.208]	134	157	152	140	85	92	72	61	193	231	221	211
K18097 glycerol 2-dehydrogenase (NADP+) [EC:1.1.1.156]	0	0	0	1	2	0	0	0	1	1	1	0
K19002 1,2-diacylglycerol 3-alpha-glucosyltransferase [EC:2.4.1.337]	226	222	285	229	304	382	336	307	453	525	465	507
K19003 1,2-diacylglycerol 3-beta-glucosyltransferase [EC:2.4.1.336]	1	0	4	1	0	0	1	0	5	3	3	1
K19005 lipoteichoic acid synthase [EC:2.7.8.20]	130	106	113	129	50	57	66	69	107	174	155	154
K22251 glycerol dehydrogenase [EC:1.1.1.6]	3	5	2	2	3	0	3	0	11	5	6	7
ko00564 Glycerophospholipid metabolism	77188	77094	75885	77077	65895	66463	65676	66179	70002	69934	68518	69693
K00057 glycerol-3-phosphate dehydrogenase (NAD(P)+) [EC:1.1.1.94]	3404	3354	3433	3401	3649	3771	3650	3735	3691	3369	3504	3480
K00096 glycerol-1-phosphate dehydrogenase [NAD(P)+] [EC:1.1.1.261]	257	242	316	296	182	199	191	170	632	631	602	611
K00105 alpha-glycerophosphate oxidase [EC:1.1.3.21]	87	88	106	112	24	37	32	52	95	126	98	116
K00111 glycerol-3-phosphate dehydrogenase [EC:1.1.5.3]	9039	8963	8559	9138	5136	4938	4829	4954	5979	5996	5881	6057
K00112 glycerol-3-phosphate dehydrogenase subunit B [EC:1.1.5.3]	779	745	664	789	534	581	549	538	1036	1121	1045	1140
K00113 glycerol-3-phosphate dehydrogenase subunit C	643	703	606	692	408	534	501	497	934	934	836	1016
K00570 phosphatidylethanolamine/phosphatidyl-N-methylethanolamine N-methyltransferase [EC:2.1.1.17 2.1.1.71]	3	0	0	1	0	3	0	0	0	2	0	1
K00968 choline-phosphate cytidylyltransferase [EC:2.7.7.15]	2	0	0	0	3	7	5	2	7	9	4	11
K00980 glycerol-3-phosphate cytidylyltransferase [EC:2.7.7.39]	128	112	121	117	122	135	154	134	69	72	65	66
K00981 phosphatidate cytidylyltransferase [EC:2.7.7.41]	4288	4498	4412	4303	5619	5594	5414	5341	4725	4684	4710	4563
K00995 CDP-diacylglycerol---glycerol-3-phosphate 3-phosphatidyltransferase [EC:2.7.8.5]	2497	2587	2647	2492	2480	2396	2450	2518	2057	2024	2017	2075
K00998 CDP-diacylglycerol---serine O-phosphatidyltransferase [EC:2.7.8.8]	792	787	797	785	497	629	570	514	1092	1170	1179	1107
K01004 phosphatidylcholine synthase [EC:2.7.8.24]	1093	1075	1019	1055	610	611	575	605	338	366	320	332
K01048 lysophospholipase [EC:3.1.1.5]	730	681	699	776	552	529	541	444	975	1046	1005	1073
K01058 phospholipase A1/A2 [EC:3.1.1.32 3.1.1.4]	506	600	573	624	373	403	400	382	768	767	775	839
K01095 phosphatidylglycerophosphatase A [EC:3.1.3.27]	1286	1315	1254	1325	1485	1451	1476	1513	1355	1531	1346	1275
K01096 phosphatidylglycerophosphatase B [EC:3.1.3.27 3.1.3.81 3.1.3.4 3.6.1.27]	476	475	467	485	269	341	291	302	786	832	708	714
K01115 phospholipase D1/2 [EC:3.1.4.4]	0	0	0	0	0	0	0	0	0	0	1	0
K01126 glycerophosphoryl diester phosphodiesterase [EC:3.1.4.46]	10729	10609	10677	10476	13418	13053	13563	13347	12072	11885	11814	11854
K01521 CDP-diacylglycerol pyrophosphatase [EC:3.6.1.26]	463	456	462	501	338	379	306	375	677	704	626	679
K01613 phosphatidylserine decarboxylase [EC:4.1.1.65]	2101	2214	2222	2138	2511	2566	2603	2499	2540	2529	2457	2424
K03735 ethanolamine ammonia-lyase large subunit [EC:4.3.1.7]	3617	3521	3472	3614	1959	1924	1872	1928	3009	2967	2743	2784
K03736 ethanolamine ammonia-lyase small subunit [EC:4.3.1.7]	2089	1998	1899	1919	1039	1008	986	1051	1785	1810	1786	1879
K04019 ethanolamine utilization protein EutA	632	660	637	693	170	176	178	152	1209	1256	1267	1408
K06131 cardiolipin synthase A/B [EC:2.7.8.-]	6648	6775	6634	6800	7975	8246	8057	7929	8213	8151	8305	8149
K06132 cardiolipin synthase C [EC:2.7.8.-]	3218	3215	3112	3166	1911	1907	1917	2208	1262	1269	1212	1301
K08744 cardiolipin synthase (CMP-forming) [EC:2.7.8.41]	244	256	272	248	112	117	97	133	323	273	309	293
K13333 lysophospholipase [EC:3.1.1.5]	0	0	0	0	0	0	0	0	2	0	0	0
K13535 cardiolipin-specific phospholipase [EC:3.1.1.-]	0	0	0	0	0	1	0	0	0	0	0	1
K13622 S-adenosylmethionine-diacylglycerol 3-amino-3-carboxypropyl transferase	0	0	3	0	0	4	0	0	0	4	2	3
K13623 S-adenosylmethionine-diacylglycerolhomoserine-N-methlytransferase	0	0	0	0	0	0	0	2	0	2	0	1
K17103 CDP-diacylglycerol---serine O-phosphatidyltransferase [EC:2.7.8.8]	2051	1856	2184	2020	2455	2446	2492	2366	1806	1746	1710	1702
K17104 phosphoglycerol geranylgeranyltransferase [EC:2.5.1.41]	0	0	0	1	2	0	0	0	0	1	2	11
K17105 geranylgeranylglycerol-phosphate geranylgeranyltransferase [EC:2.5.1.42]	0	0	0	1	0	0	0	0	8	6	1	9
K17830 digeranylgeranylglycerophospholipid reductase [EC:1.3.1.101 1.3.7.11]	1	0	0	1	3	0	3	0	4	6	6	3
K18695 glycerophosphocholine phosphodiesterase GPCPD1 [EC:3.1.4.2]	0	1	0	0	0	0	0	0	1	0	0	0
K18697 phosphatidylglycerophosphatase C [EC:3.1.3.27]	441	400	385	395	262	302	289	273	542	527	517	572
K19664 CDP-2,3-bis-(O-geranylgeranyl)-sn-glycerol synthase [EC:2.7.7.67]	0	2	0	1	2	2	0	0	0	8	4	5
K19665 archaetidylserine synthase [EC:2.7.8.38]	0	0	0	0	0	2	0	0	2	3	0	3
ko00565 Ether lipid metabolism	9206	9409	8708	9131	4871	4989	4837	5257	3592	3364	3216	3687
K00803 alkyldihydroxyacetonephosphate synthase [EC:2.5.1.26]	2186	2237	2037	2054	1110	1046	1085	1241	681	643	632	778
K22387 lysophospholipase D [EC:3.1.4.39]	1	0	0	0	0	0	0	0	0	0	0	0
ko00600 Sphingolipid metabolism	43452	45719	47280	45329	92594	92965	93944	91652	79267	76794	78525	76530
K01134 arylsulfatase A [EC:3.1.6.8]	4	2	2	1	1	0	1	2	3	0	0	0
K01186 sialidase-1 [EC:3.2.1.18]	2176	2319	2579	2298	7677	7901	8247	7864	6136	5760	6039	5759
K01201 glucosylceramidase [EC:3.2.1.45]	1191	1264	1341	1326	2858	2834	3006	2823	2322	2407	2435	2307
K01202 galactosylceramidase [EC:3.2.1.46]	0	0	0	0	0	0	0	0	0	3	3	2
K04708 3-dehydrosphinganine reductase [EC:1.1.1.102]	2	2	2	2	0	0	0	0	3	5	0	1
K12349 neutral ceramidase [EC:3.5.1.23]	1013	1084	1084	1083	591	584	534	594	349	336	342	310
K17108 non-lysosomal glucosylceramidase [EC:3.2.1.45]	4	3	1	3	9	3	4	13	0	0	2	0
ko00590 Arachidonic acid metabolism	6272	6577	6237	6248	4543	4560	4500	4880	4094	3858	4112	3981
K00432 glutathione peroxidase [EC:1.11.1.9]	3214	3293	3268	3173	2836	2907	2880	3011	2566	2388	2579	2357
K18592 gamma-glutamyltranspeptidase / glutathione hydrolase / leukotriene-C4 hydrolase [EC:2.3.2.2 3.4.19.13 3.4.19.14]	0	0	0	0	0	0	0	0	0	1	0	1
K19246 arachidonate 15-lipoxygenase [EC:1.13.11.33]	2551	2684	2396	2450	1334	1250	1220	1487	760	702	757	783
K23486 9,11-endoperoxide prostaglandin H2 reductase [EC:1.1.1.-]	1	0	0	1	0	0	0	0	0	0	1	1
ko00591 Linoleic acid metabolism	1600	1779	1452	1725	975	935	954	1024	1128	1105	1087	1195
K00508 linoleoyl-CoA desaturase [EC:1.14.19.3]	1094	1179	879	1101	602	532	554	642	360	338	312	356
ko00592 alpha-Linolenic acid metabolism	7277	7298	6975	7260	4404	4295	4323	4307	4583	4492	4436	4678
K10526 OPC-8:0 CoA ligase 1 [EC:6.2.1.-]	0	0	0	0	0	0	0	0	0	1	0	0
ko01040 Biosynthesis of unsaturated fatty acids	3612	3527	3206	3417	2253	2179	2077	2167	2306	2323	2265	2300
K00507 stearoyl-CoA desaturase (Delta-9 desaturase) [EC:1.14.19.1]	1	0	0	2	0	0	0	0	0	0	0	0
K10804 acyl-CoA thioesterase I [EC:3.1.2.- 3.1.2.2 3.1.1.2 3.1.1.5]	1295	1250	1073	1161	804	759	722	798	772	799	764	813
K10805 acyl-CoA thioesterase II [EC:3.1.2.-]	1421	1451	1311	1448	932	889	814	879	1019	1060	980	987
K10806 acyl-CoA thioesterase YciA [EC:3.1.2.-]	895	824	822	806	515	531	541	490	515	464	516	500
Nucleotide metabolism	313861	313109	312215	313931	302565	306153	303551	302813	312106	311970	308504	310587
ko00230 Purine metabolism	232710	232578	231060	232794	215359	217957	216249	216099	224942	224048	221457	223084
K00073 ureidoglycolate dehydrogenase (NAD+) [EC:1.1.1.350]	105	97	90	114	33	47	30	28	136	156	163	144
K00087 xanthine dehydrogenase molybdenum-binding subunit [EC:1.17.1.4]	1397	1235	1202	1161	450	511	579	427	1154	1155	1139	1178
K00088 IMP dehydrogenase [EC:1.1.1.205]	6113	6256	6328	6186	5917	5929	5688	5877	6696	6633	6656	6447
K00106 xanthine dehydrogenase/oxidase [EC:1.17.1.4 1.17.3.2]	0	2	0	0	0	4	0	0	0	0	0	0
K00364 GMP reductase [EC:1.7.1.7]	851	748	793	795	546	649	569	568	967	1054	1017	1132
K00525 ribonucleoside-diphosphate reductase alpha chain [EC:1.17.4.1]	13702	13778	13670	13792	12280	12707	12530	12324	11834	11674	11872	11631
K00526 ribonucleoside-diphosphate reductase beta chain [EC:1.17.4.1]	3364	3435	3494	3409	2172	2083	2073	2205	2985	3126	2896	2972
K00527 ribonucleoside-triphosphate reductase (thioredoxin) [EC:1.17.4.2]	0	4	5	0	0	0	0	0	0	0	3	0
K00602 phosphoribosylaminoimidazolecarboxamide formyltransferase / IMP cyclohydrolase [EC:2.1.2.3 3.5.4.10]	6501	6459	6298	6439	8668	8594	8740	8669	7719	7319	7267	7176
K00759 adenine phosphoribosyltransferase [EC:2.4.2.7]	1881	1730	1859	1756	2044	2048	2018	2092	2126	2020	2115	2097
K00760 hypoxanthine phosphoribosyltransferase [EC:2.4.2.8]	1754	1724	1747	1742	1867	2028	1899	1948	1907	1905	1916	1783
K00764 amidophosphoribosyltransferase [EC:2.4.2.14]	6666	6934	6999	6655	8994	8899	9306	8904	8218	8390	8157	7922
K00769 xanthine phosphoribosyltransferase [EC:2.4.2.22]	366	263	330	309	200	232	228	201	386	457	453	450
K00839 (S)-ureidoglycine---glyoxylate transaminase [EC:2.6.1.112]	778	856	701	773	489	545	501	550	924	1048	932	1000
K00856 adenosine kinase [EC:2.7.1.20]	21	23	33	23	28	27	26	32	29	36	41	25
K00892 inosine kinase [EC:2.7.1.73]	762	853	789	733	556	557	524	551	1097	1159	1098	1121
K00939 adenylate kinase [EC:2.7.4.3]	2278	2392	2287	2309	2317	2314	2309	2307	2295	2302	2241	2242
K00940 nucleoside-diphosphate kinase [EC:2.7.4.6]	1293	1297	1283	1331	1669	1584	1622	1657	1383	1281	1352	1408
K00942 guanylate kinase [EC:2.7.4.8]	2224	2361	2259	2284	2343	2319	2419	2373	2354	2243	2421	2309
K00951 GTP pyrophosphokinase [EC:2.7.6.5]	8335	8795	8948	8781	12489	12533	12563	12401	10402	10539	10661	10155
K01081 5'-nucleotidase [EC:3.1.3.5]	601	692	723	572	2066	1866	1899	1819	1548	1413	1592	1529
K01119 2',3'-cyclic-nucleotide 2'-phosphodiesterase / 3'-nucleotidase [EC:3.1.4.16 3.1.3.6]	2063	2122	2135	2070	2992	3075	2865	2958	3265	3362	3009	3238
K01129 dGTPase [EC:3.1.5.1]	5928	5682	5651	5698	5441	5507	5437	5429	5223	5168	5054	5183
K01139 GTP diphosphokinase / guanosine-3',5'-bis(diphosphate) 3'-diphosphatase [EC:2.7.6.5 3.1.7.2]	2661	2580	2551	2683	1714	1888	1727	1802	2428	2485	2366	2480
K01239 purine nucleosidase [EC:3.2.2.1]	2083	2000	1842	1966	1081	1028	996	1081	976	960	833	871
K01241 AMP nucleosidase [EC:3.2.2.4]	3632	3717	3515	3590	3442	3374	3221	3261	2988	2834	2780	2887
K01427 urease [EC:3.5.1.5]	0	1	2	1	0	0	0	1	0	0	0	0
K01428 urease subunit alpha [EC:3.5.1.5]	3987	3782	3507	3779	1882	1699	1728	1859	2884	2892	2701	2946
K01429 urease subunit beta [EC:3.5.1.5]	660	709	630	611	318	310	294	348	402	401	419	446
K01430 urease subunit gamma [EC:3.5.1.5]	584	622	555	574	257	295	268	293	439	443	452	474
K01466 allantoinase [EC:3.5.2.5]	334	292	304	325	149	192	156	172	324	335	318	327
K01477 allantoicase [EC:3.5.3.4]	1509	1516	1417	1468	825	762	787	820	494	407	431	428
K01483 ureidoglycolate lyase [EC:4.3.2.3]	777	794	715	718	451	412	390	371	234	227	249	252
K01486 adenine deaminase [EC:3.5.4.2]	2414	2266	2294	2344	624	587	612	596	2638	3008	2783	3082
K01487 guanine deaminase [EC:3.5.4.3]	4282	4148	4110	4335	2042	2144	2015	2100	2466	2586	2269	2508
K01488 adenosine deaminase [EC:3.5.4.4]	1348	1350	1330	1429	549	561	616	517	1394	1573	1511	1534
K01515 ADP-ribose pyrophosphatase [EC:3.6.1.13]	1708	1705	1696	1753	942	862	1051	1030	1202	1228	1128	1284
K01518 bis(5'-nucleosidyl)-tetraphosphatase [EC:3.6.1.17]	0	0	0	0	0	0	0	1	2	0	1	0
K01524 exopolyphosphatase / guanosine-5'-triphosphate,3'-diphosphate pyrophosphatase [EC:3.6.1.11 3.6.1.40]	5769	5686	5729	5637	3508	3685	3614	3602	4826	4859	4863	4873
K01525 bis(5'-nucleosyl)-tetraphosphatase (symmetrical) [EC:3.6.1.41]	1844	1661	1791	1880	1138	1130	1117	1163	1277	1188	1217	1218
K01588 5-(carboxyamino)imidazole ribonucleotide mutase [EC:5.4.99.18]	1580	1637	1637	1564	1756	1871	1753	1800	1900	1711	1818	1767
K01589 5-(carboxyamino)imidazole ribonucleotide synthase [EC:6.3.4.18]	1984	1984	1810	1889	1216	1312	1160	1313	1568	1437	1575	1488
K01756 adenylosuccinate lyase [EC:4.3.2.2]	4686	4570	4836	4919	5238	5319	5155	5028	5016	5066	4726	4872
K01768 adenylate cyclase [EC:4.6.1.1]	1713	1620	1470	1628	874	800	827	846	577	476	507	508
K01923 phosphoribosylaminoimidazole-succinocarboxamide synthase [EC:6.3.2.6]	2751	2713	2651	2728	3209	3193	3283	3222	3071	2993	2966	2975

K01933 phosphoribosylformylglycinamidine cyclo-ligase [EC:6.3.3.1]	3845	3888	3914	4006	4236	4459	4139	4206	4268	4279	4371	4262
K01939 adenylosuccinate synthase [EC:6.3.4.4]	4749	4755	4893	4816	5048	5100	4981	4927	4913	4993	4954	5049
K01945 phosphoribosylamine---glycine ligase [EC:6.3.4.13]	4766	4817	4698	4624	5075	5203	5160	5270	4978	4924	5054	4804
K01951 GMP synthase (glutamine-hydrolysing) [EC:6.3.5.2]	7368	7456	7759	7400	9383	9471	9336	9310	8978	8745	8786	8640
K01952 phosphoribosylformylglycinamidine synthase [EC:6.3.5.3]	12215	12259	12273	12342	13001	13269	13457	13621	12170	11877	11837	12247
K02083 allantoate deiminase [EC:3.5.3.9]	841	833	793	829	517	602	526	504	970	1110	1022	1052
K02428 XTP/dITP diphosphohydrolase [EC:3.6.1.66]	2249	2296	2426	2300	3759	3755	3788	3673	3398	3276	3362	3300
K03651 3',5'-cyclic-AMP phosphodiesterase [EC:3.1.4.53]	2371	2281	2089	2282	1228	1218	1255	1392	1983	1897	1829	1988
K03783 purine-nucleoside phosphorylase [EC:2.4.2.1]	973	1038	1075	993	1973	2101	2051	1976	1723	1673	1671	1749
K03784 purine-nucleoside phosphorylase [EC:2.4.2.1]	868	985	869	1002	591	631	509	517	965	1076	1039	1019
K03787 5'-nucleotidase [EC:3.1.3.5]	1497	1639	1662	1567	2344	2396	2515	2301	2187	2128	2139	2191
K03815 xanthosine phosphorylase [EC:2.4.2.-]	3	0	3	0	3	12	9	16	34	65	66	42
K03816 xanthine phosphoribosyltransferase [EC:2.4.2.22]	1731	1816	1755	1746	1795	1838	1924	1799	1565	1552	1527	1418
K04765 nucleoside triphosphate diphosphatase [EC:3.6.1.9]	703	695	699	766	494	503	476	458	704	742	643	837
K05851 adenylate cyclase, class 1 [EC:4.6.1.1]	6311	6262	5805	6160	3757	3673	3565	3734	3676	3624	3452	3589
K05873 adenylate cyclase, class 2 [EC:4.6.1.1]	0	5	5	7	8	2	3	3	0	9	13	7
K06863 5-formaminoimidazole-4-carboxamide-1-(beta)-D-ribofuranosyl 5'-monophosphate synthetase [EC:6.3.4.23]	0	0	2	1	0	2	0	0	7	9	6	15
K06928 nucleoside-triphosphatase [EC:3.6.1.15]	1	0	0	0	12	18	15	20	3	0	3	9
K06966 pyrimidine/purine-5'-nucleotide nucleosidase [EC:3.2.2.10 3.2.2.-]	929	1029	974	976	650	657	641	707	1261	1242	1230	1346
K07127 5-hydroxyisourate hydrolase [EC:3.5.2.17]	944	944	851	901	487	517	469	476	744	850	729	834
K07816 putative GTP pyrophosphokinase [EC:2.7.6.5]	1089	1086	1222	1117	315	332	293	286	1116	1201	1183	1185
K08289 phosphoribosylglycinamide formyltransferase 2 [EC:2.1.2.2]	2128	2104	2047	2107	2336	2350	2356	2228	2495	2475	2450	2507
K08312 ADP-ribose diphosphatase [EC:3.6.1.-]	1128	1004	1100	1154	689	729	692	625	735	648	753	665
K08693 2',3'-cyclic-nucleotide 2'-phosphodiesterase / 3'-nucleotidase / 5'-nucleotidase [EC:3.1.4.16 3.1.3.6 3.1.3.5]	0	0	1	0	0	0	0	0	1	0	3	1
K08723 5'-nucleotidase [EC:3.1.3.5]	433	488	433	460	303	312	318	312	546	649	526	623
K09913 purine/pyrimidine-nucleoside phosphorylase [EC:2.4.2.1 2.4.2.2]	554	642	623	593	368	320	340	368	407	420	382	442
K10213 ribosylpyrimidine nucleosidase [EC:3.2.2.8]	164	168	154	159	231	247	223	253	31	61	59	58
K10353 deoxyadenosine kinase [EC:2.7.1.76]	0	0	0	0	0	3	0	2	1	0	0	0
K10807 ribonucleoside-diphosphate reductase subunit M1 [EC:1.17.4.1]	0	0	0	0	0	0	0	0	0	0	0	3
K11175 phosphoribosylglycinamide formyltransferase 1 [EC:2.1.2.2]	2241	2257	2294	2276	2476	2529	2566	2446	2400	2427	2297	2273
K11176 IMP cyclohydrolase [EC:3.5.4.10]	0	5	0	0	2	0	0	0	0	3	5	2
K11177 xanthine dehydrogenase YagR molybdenum-binding subunit [EC:1.17.1.4]	3204	3053	3060	3008	1685	1584	1622	1687	901	845	966	942
K11178 xanthine dehydrogenase YagS FAD-binding subunit [EC:1.17.1.4]	1319	1325	1181	1242	672	634	663	649	331	319	313	399
K11751 5'-nucleotidase / UDP-sugar diphosphatase [EC:3.1.3.5 3.6.1.45]	2851	2774	2825	2851	988	1083	967	938	3132	3277	3245	3338
K11788 phosphoribosylamine--glycine ligase / phosphoribosylformylglycinamidine cyclo-ligase [EC:6.3.4.13 6.3.3.1]	1	0	1	1	0	1	0	2	2	0	3	1
K13479 xanthine dehydrogenase FAD-binding subunit [EC:1.17.1.4]	465	435	428	459	110	134	128	144	372	464	405	434
K13480 xanthine dehydrogenase iron-sulfur-binding subunit	2	8	7	3	8	5	11	11	25	25	24	38
K13481 xanthine dehydrogenase small subunit [EC:1.17.1.4]	1804	1887	1875	1885	1019	1047	978	1081	639	556	516	612
K13482 xanthine dehydrogenase large subunit [EC:1.17.1.4]	3438	3542	3398	3534	1833	1840	1816	1983	1123	1089	953	1095
K13483 xanthine dehydrogenase YagT iron-sulfur-binding subunit	642	707	590	631	309	372	330	380	205	184	192	210
K14048 urease subunit gamma/beta [EC:3.5.1.5]	13	23	15	16	4	5	3	1	33	34	24	49
K14642 golgi apyrase [EC:3.6.1.5]	0	0	0	0	0	0	0	0	0	0	2	0
K14977 (S)-ureidoglycine aminohydrolase [EC:3.5.3.26]	1534	1319	1269	1377	755	776	692	841	600	585	497	589
K15518 deoxyguanosine kinase [EC:2.7.1.113]	2	0	0	0	0	0	0	0	0	0	0	0
K15519 deoxyadenosine/deoxycytidine kinase [EC:2.7.1.76 2.7.1.74]	2	0	0	0	0	0	0	0	0	0	0	0
K15780 bifunctional protein TilS/HprT [EC:6.3.4.19 2.4.2.8]	0	0	1	0	0	0	0	0	0	0	2	1
K16839 FAD-dependent urate hydroxylase [EC:1.14.13.113]	691	717	599	666	450	454	427	468	930	996	884	972
K16840 2-oxo-4-hydroxy-4-carboxy-5-ureidoimidazoline decarboxylase [EC:4.1.1.97]	267	263	238	276	166	184	214	185	377	372	404	410
K16841 allantoin racemase [EC:5.1.99.3]	414	428	422	481	278	302	333	264	630	586	564	625
K16842 allantoinase [EC:3.5.2.5]	544	544	491	562	394	364	375	359	593	656	579	641
K16856 ureidoglycolate lyase [EC:4.3.2.3]	0	0	0	1	0	0	0	0	0	0	0	0
K18151 ureidoglycolate amidohydrolase [EC:3.5.1.116]	7	2	5	5	0	1	1	0	3	2	2	0
K18532 adenylate kinase [EC:2.7.4.3]	0	0	0	0	2	0	0	0	2	4	0	10
K19572 adenosine deaminase CECR1 [EC:3.5.4.4]	0	0	0	1	0	0	0	0	1	0	0	1
K19710 ATP adenylyltransferase [EC:2.7.7.53]	6	5	3	3	0	4	0	1	4	13	4	9
K20881 5'-nucleotidase [EC:3.1.3.5]	1623	1681	1635	1586	972	1035	963	1047	1004	1011	951	1006
K21053 adenine deaminase [EC:3.5.4.2]	1663	1684	1419	1586	849	816	805	890	525	463	465	487
K21636 ribonucleoside-triphosphate reductase (formate) [EC:1.1.98.6]	7675	7450	7766	7789	9242	9496	9482	9090	9724	9824	9930	9508
K22026 nucleoside kinase [EC:2.7.1.73 2.7.1.213 2.7.1.-]	2	0	0	0	2	2	0	1	4	4	4	8
K22601 oxamate carbamoyltransferase [EC:2.1.3.5]	686	686	724	748	505	550	498	612	824	843	807	909
K22602 oxamate amidohydrolase [EC:3.5.1.126]	956	959	899	896	608	652	654	621	1278	1195	1158	1245
K23264 phosphoribosylformylglycinamidine synthase subunit PurS [EC:6.3.5.3]	14	16	15	16	2	5	4	2	19	35	19	15
K23265 phosphoribosylformylglycinamidine synthase subunit PurQ / glutaminase [EC:6.3.5.3 3.5.1.2]	36	43	47	40	16	6	19	15	50	70	53	51
K23269 phosphoribosylformylglycinamidine synthase subunit PurL [EC:6.3.5.3]	115	126	122	149	30	40	29	16	133	160	137	158
K23270 phosphoribosylformylglycinamidine synthase subunit PurSL [EC:6.3.5.3]	36	26	33	26	5	8	6	11	31	51	33	31
ko00240 Pyrimidine metabolism	120448	120448	121181	121053	126543	128294	126776	125646	129870	130885	129597	130410
K00226 dihydroorotate dehydrogenase (fumarate) [EC:1.3.98.1]	564	580	641	647	1926	1835	1959	1870	1445	1374	1317	1250
K00254 dihydroorotate dehydrogenase [EC:1.3.5.2]	1807	1666	1607	1751	1165	1114	1060	1044	1148	1259	1176	1240
K00560 thymidylate synthase [EC:2.1.1.45]	2636	2575	2688	2634	2925	2856	2896	2814	2874	2808	2929	2870
K00609 aspartate carbamoyltransferase catalytic subunit [EC:2.1.3.2]	2939	2896	2850	2874	3021	3172	3208	3139	2955	2907	3075	3068
K00610 aspartate carbamoyltransferase regulatory subunit	769	735	804	794	1095	1202	1096	1110	1211	1227	1249	1201
K00756 pyrimidine-nucleoside phosphorylase [EC:2.4.2.2]	463	442	446	439	81	93	84	97	330	387	360	349
K00757 uridine phosphorylase [EC:2.4.2.3]	2183	2103	2314	2113	2573	2578	2680	2605	2910	3000	2945	2912
K00758 thymidine phosphorylase [EC:2.4.2.4]	813	893	836	891	609	653	576	566	1222	1237	1100	1315
K00761 uracil phosphoribosyltransferase [EC:2.4.2.9]	2157	2183	2153	2242	2502	2393	2458	2481	2619	2616	2505	2579
K00762 orotate phosphoribosyltransferase [EC:2.4.2.10]	2979	2878	3209	3023	2765	2761	2728	2734	3093	3056	3101	3005
K00857 thymidine kinase [EC:2.7.1.21]	820	816	911	865	1564	1631	1663	1644	1519	1596	1547	1449
K00876 uridine kinase [EC:2.7.1.48]	2775	2805	2927	2935	5792	5796	5919	5533	5055	5071	5253	5061
K00943 dTMP kinase [EC:2.7.4.9]	773	786	699	754	480	520	570	498	781	814	742	735
K00945 CMP/dCMP kinase [EC:2.7.4.25]	1662	1789	1866	1810	2403	2393	2354	2287	2291	2369	2486	2314
K01240 uridine nucleosidase [EC:3.2.2.3]	0	0	0	1	0	0	1	2	3	0	0	0
K01464 dihydropyrimidinase [EC:3.5.2.2]	3509	3257	3061	3231	1774	1730	1630	1808	1484	1525	1433	1370
K01465 dihydroorotase [EC:3.5.2.3]	8609	8435	8482	8595	6462	6377	6196	6464	6230	5985	6074	6215
K01485 cytosine/creatinine deaminase [EC:3.5.4.1 3.5.4.21]	3946	4137	3722	3907	2167	2340	2245	2205	3905	4103	3980	3957
K01489 cytidine deaminase [EC:3.5.4.5]	1112	1096	1205	1186	1343	1273	1280	1303	1687	1758	1738	1697
K01493 dCMP deaminase [EC:3.5.4.12]	724	744	762	708	1123	1211	1214	1074	1234	1026	1076	1087
K01494 dCTP deaminase [EC:3.5.4.13]	1440	1367	1399	1417	768	772	734	740	1105	1108	1076	1173
K01520 dUTP pyrophosphatase [EC:3.6.1.23]	1968	1979	1980	1941	2141	2086	2055	2182	1868	1930	1921	1929
K01591 orotidine-5'-phosphate decarboxylase [EC:4.1.1.23]	2609	2494	2565	2615	2938	2865	2790	2940	2768	2611	2621	2746
K01937 CTP synthase [EC:6.3.4.2]	3440	3517	3557	3395	4528	4932	4744	4575	4789	4799	4742	4839
K01954 carbamoyl-phosphate synthase [EC:6.3.5.5]	0	0	1	0	0	0	0	0	0	0	0	0
K01955 carbamoyl-phosphate synthase large subunit [EC:6.3.5.5]	11446	11724	11976	11789	17411	17349	17323	17253	16167	16086	16146	15808
K01956 carbamoyl-phosphate synthase small subunit [EC:6.3.5.5]	3413	3507	3550	3397	3829	3872	3800	3724	3568	3644	3572	3711
K02823 dihydroorotate dehydrogenase electron transfer subunit	887	920	995	870	1506	1532	1662	1477	1386	1501	1370	1441
K02825 pyrimidine operon attenuation protein / uracil phosphoribosyltransferase [EC:2.4.2.9]	564	545	597	536	246	277	272	337	261	219	205	282
K03365 cytosine/creatinine deaminase [EC:3.5.4.1 3.5.4.21]	0	0	0	0	0	3	0	0	3	6	0	4
K03465 thymidylate synthase (FAD) [EC:2.1.1.148]	47	50	51	71	33	43	61	53	114	134	146	121
K06016 beta-ureidopropionase / N-carbamoyl-L-amino-acid hydrolase [EC:3.5.1.6 3.5.1.87]	2461	2229	2262	2343	1629	1721	1528	1566	1372	1452	1340	1460
K08722 5'-deoxynucleotidase [EC:3.1.3.89]	482	411	368	422	247	258	298	281	472	552	512	542
K08728 nucleoside deoxyribosyltransferase [EC:2.4.2.6]	0	0	0	0	2	2	0	1	0	0	0	0
K09018 pyrimidine oxygenase [EC:1.14.99.46]	567	569	544	586	390	477	416	440	784	774	712	796
K09019 3-hydroxypropanoate dehydrogenase [EC:1.1.1.-]	300	301	275	311	223	228	222	255	399	473	448	440
K09020 ureidoacrylate peracid hydrolase [EC:3.5.1.110]	370	410	410	405	263	281	313	279	546	558	513	507
K09021 aminoacrylate peracid reductase	191	216	174	201	127	151	153	130	262	263	263	252
K09023 aminoacrylate hydrolase [EC:3.5.1.-]	491	454	410	515	269	327	332	341	667	628	604	740
K09024 flavin reductase [EC:1.5.1.-]	300	315	319	285	214	242	213	216	460	468	390	458
K09903 uridylate kinase [EC:2.7.4.22]	2613	2606	2717	2626	2881	2951	2902	2792	2506	2754	2585	2703
K11540 carbamoyl-phosphate synthase / aspartate carbamoyltransferase / dihydroorotase [EC:6.3.5.5 2.1.3.2 3.5.2.3]	1	2	2	0	0	2	3	0	2	1	0	1
K11541 carbamoyl-phosphate synthase / aspartate carbamoyltransferase [EC:6.3.5.5 2.1.3.2]	1	2	1	0	1	2	1	3	1	0	0	1
K13421 uridine monophosphate synthetase [EC:2.4.2.10 4.1.1.23]	1	0	2	0	0	2	2	0	0	0	0	0
K16066 3-hydroxy acid dehydrogenase / malonic semialdehyde reductase [EC:1.1.1.381 1.1.1.-]	507	368	384	421	278	309	305	295	597	643	588	695
K16328 pseudouridine kinase [EC:2.7.1.83]	515	438	415	422	678	683	650	688	174	157	168	163
K16329 pseudouridylate synthase [EC:4.2.1.70]	271	234	215	241	321	346	310	334	99	113	123	93
K17722 dihydropyrimidine dehydrogenase (NAD+) subunit PreT [EC:1.3.1.1]	1823	1764	1655	1692	1294	1289	1292	1320	510	547	514	514
K17723 dihydropyrimidine dehydrogenase (NAD+) subunit PreA [EC:1.3.1.1]	2259	2260	2049	2121	1488	1395	1201	1539	618	659	641	665
K17828 dihydroorotate dehydrogenase (NAD+) catalytic subunit [EC:1.3.1.14]	944	1033	1099	1115	1731	1871	1903	1675	1670	1724	1761	1745
Amino acid metabolism	928212	920354	906433	918771	830701	829189	823081	834247	824292	823098	814008	821853
ko00250 Alanine, aspartate and glutamate metabolism	165048	165483	165153	164301	182305	181946	182325	181734	169127	169019	168291	167352
K00259 alanine dehydrogenase [EC:1.4.1.1]	721	851	830	738	2287	2337	2369	2165	1856	1597	1732	1630
K00278 L-aspartate oxidase [EC:1.4.3.16]	4449	4501	4437	4428	5809	5871	5924	5696	5188	5096	4897	4862
K00294 1-pyrroline-5-carboxylate dehydrogenase [EC:1.2.1.88]	21	14	18	16	3	0	0	0	3	10	4	3

K00812 aspartate aminotransferase [EC:2.6.1.1]	1589	1772	1943	1689	5232	5097	5317	5193	3844	3733	3875	3655
K00813 aspartate aminotransferase [EC:2.6.1.1]	671	674	656	736	477	493	430	444	884	942	872	1056
K01424 L-asparaginase [EC:3.5.1.1]	3849	3972	3973	4000	7585	7501	7727	7685	7429	7388	7394	7118
K01425 glutaminase [EC:3.5.1.2]	1423	1361	1424	1428	2703	2855	3083	2739	2523	2349	2628	2450
K01744 aspartate ammonia-lyase [EC:4.3.1.1]	4427	4526	4316	4271	5404	5342	5430	5568	5061	4961	4983	4932
K01755 argininosuccinate lyase [EC:4.3.2.1]	5369	5397	5524	5321	5867	6017	6011	6088	5538	5625	5496	5635
K01779 aspartate racemase [EC:5.1.1.13]	530	565	483	574	426	482	409	398	374	434	408	412
K01914 aspartate--ammonia ligase [EC:6.3.1.1]	1802	1845	1862	1810	2827	2924	3004	2780	2999	3036	3042	2906
K01940 argininosuccinate synthase [EC:6.3.4.5]	4155	4382	4200	3990	4692	4578	4660	4906	4226	4227	4107	4251
K01953 asparagine synthase (glutamine-hydrolysing) [EC:6.3.5.4]	11561	11240	10660	10963	8426	8356	8249	8602	7075	6942	6910	6985
K05597 glutamin-(asparagin-)ase [EC:3.5.1.38]	209	199	178	188	110	92	104	115	64	62	56	71
K09758 aspartate 4-decarboxylase [EC:4.1.1.12]	940	1089	1072	1119	2774	2888	2855	2967	2094	2022	2219	1939
K11358 aspartate aminotransferase [EC:2.6.1.1]	502	502	518	516	147	125	149	145	447	472	411	468
K13051 L-asparaginase / beta-aspartyl-peptidase [EC:3.5.1.1 3.4.19.5]	657	610	607	647	478	581	556	459	737	781	709	770
K13566 omega-amidase [EC:3.5.1.3]	2149	2128	2191	2119	2595	2652	2570	2537	2126	2238	2171	2142
K13821 RHH-type transcriptional regulator, proline utilization regulon repressor / proline dehydrogenase / delta 1-pyrroline-5-carboxylate dehydrogenase [E	6056	5732	5737	5696	3788	3676	3667	3932	4224	4224	3917	4216
K14260 alanine-synthesizing transaminase [EC:2.6.1.66 2.6.1.2]	2511	2457	2615	2520	1531	1566	1519	1504	1910	1937	1854	1927
K14681 argininosuccinate lyase / amino-acid N-acetyltransferase [EC:4.3.2.1 2.3.1.1]	8	8	12	9	10	12	15	11	16	11	14	12
K19244 alanine dehydrogenase [EC:1.4.1.1]	0	3	0	2	0	0	1	2	4	1	2	10
K22457 asparagine---oxo-acid transaminase [EC:2.6.1.14]	82	55	80	70	22	27	16	6	62	102	68	107
ko00260 Glycine, serine and threonine metabolism	158044	156295	152537	155504	140898	141299	139962	141766	141694	139878	139509	140126
K00003 homoserine dehydrogenase [EC:1.1.1.3]	2777	2707	2779	2703	1433	1334	1351	1461	1522	1514	1523	1526
K00060 threonine 3-dehydrogenase [EC:1.1.1.103]	1000	949	1044	1073	490	592	496	493	1565	1590	1511	1580
K00108 choline dehydrogenase [EC:1.1.99.1]	6688	6362	6082	6470	3340	3240	3512	3591	3390	3448	3156	3252
K00130 betaine-aldehyde dehydrogenase [EC:1.2.1.8]	3519	3673	3393	3561	1726	1657	1553	1708	2545	2587	2481	2467
K00133 aspartate-semialdehyde dehydrogenase [EC:1.2.1.11]	3907	4075	3992	4083	3987	4038	4111	3983	4784	4641	4814	4672
K00274 monoamine oxidase [EC:1.4.3.4]	2541	2664	2610	2588	1445	1409	1307	1544	864	874	791	749
K00276 primary-amine oxidase [EC:1.4.3.21]	799	774	720	792	209	222	195	209	1545	1642	1487	1568
K00302 sarcosine oxidase, subunit alpha [EC:1.5.3.1]	5148	5221	4686	4775	2694	2506	2568	2926	1705	1576	1489	1702
K00303 sarcosine oxidase, subunit beta [EC:1.5.3.1]	1941	1835	1589	1816	968	904	927	992	623	552	526	603
K00304 sarcosine oxidase, subunit delta [EC:1.5.3.1]	519	561	507	502	340	321	268	322	173	193	188	214
K00305 sarcosine oxidase, subunit gamma [EC:1.5.3.1]	6	13	2	3	3	3	1	5	4	4	3	1
K00314 sarcosine dehydrogenase [EC:1.5.8.3]	0	0	0	0	0	0	0	0	0	1	0	0
K00613 glycine amidinotransferase [EC:2.1.4.1]	11	8	5	11	12	9	7	12	19	30	26	31
K00639 glycine C-acetyltransferase [EC:2.3.1.29]	1585	1696	1665	1695	2989	3283	3250	3087	2927	2976	2973	2938
K00643 5-aminolevulinate synthase [EC:2.3.1.37]	1	0	0	1	2	1	3	0	2	1	3	3
K00836 diaminobutyrate-2-oxoglutarate transaminase [EC:2.6.1.76]	1899	1881	1861	1888	1116	1101	1039	1063	1493	1590	1377	1548
K00872 homoserine kinase [EC:2.7.1.39]	690	706	656	708	424	458	492	439	852	883	918	982
K00928 aspartate kinase [EC:2.7.2.4]	5433	5415	5737	5526	7655	7537	7771	7396	6513	6555	6530	6417
K01620 threonine aldolase [EC:4.1.2.48]	5121	5056	4889	5075	4321	4190	4266	4486	4014	3819	3782	3979
K01695 tryptophan synthase alpha chain [EC:4.2.1.20]	2398	2529	2414	2500	2507	2534	2459	2570	2450	2351	2350	2428
K01696 tryptophan synthase beta chain [EC:4.2.1.20]	3407	3488	3374	3542	3727	3666	3808	3760	3718	3670	3836	3655
K01697 cystathionine beta-synthase [EC:4.2.1.22]	2036	2021	1964	2117	1134	1258	1185	1214	1517	1575	1531	1593
K01733 threonine synthase [EC:4.2.3.1]	5015	4928	5092	5061	5326	5341	5175	5358	5075	4936	5149	5069
K01752 L-serine dehydratase [EC:4.3.1.17]	6980	6897	6631	6587	6102	6057	6175	6262	6318	6343	6198	6353
K01753 D-serine dehydratase [EC:4.3.1.18]	3125	3132	2796	3041	1806	1728	1710	1741	2098	2015	2003	1984
K01754 threonine dehydratase [EC:4.3.1.19]	6866	6744	6331	6812	3613	3717	3565	3712	4149	4296	4212	4377
K01758 cystathionine gamma-lyase [EC:4.4.1.1]	2729	2548	2406	2518	1539	1646	1410	1619	1603	1519	1478	1509
K02204 homoserine kinase type II [EC:2.7.1.39]	1420	1487	1433	1311	809	724	781	800	479	379	451	469
K06001 tryptophan synthase beta chain [EC:4.2.1.20]	1743	1712	1843	1772	3103	2922	3130	3111	2950	2971	3086	2895
K06720 L-ectoine synthase [EC:4.2.1.108]	0	0	0	0	2	1	1	0	0	2	0	1
K11440 choline dehydrogenase [EC:1.1.1.1]	1	0	0	0	0	1	1	0	0	0	0	0
K12524 bifunctional aspartokinase / homoserine dehydrogenase 1 [EC:2.7.2.4 1.1.1.3]	3160	3193	3211	3233	6630	6536	6608	6626	5999	5925	6009	5745
K12525 bifunctional aspartokinase / homoserine dehydrogenase 2 [EC:2.7.2.4 1.1.1.3]	1610	1664	1653	1589	1158	1176	1126	1080	2170	2228	2181	2255
K13290 serine---pyruvate transaminase [EC:2.6.1.51]	1	0	1	0	2	0	0	0	0	0	0	0
K13745 L-2,4-diaminobutyrate decarboxylase [EC:4.1.1.86]	783	741	729	765	481	520	533	537	1070	1082	965	1120
K15783 ectoine hydrolase [EC:3.5.4.44]	2	0	0	1	3	1	1	0	0	0	2	2
K15784 N2-acetyl-L-2,4-diaminobutanoate deacetylase [EC:3.5.1.125]	0	0	2	1	0	0	2	0	5	6	2	0
K15785 L-2,4-diaminobutyrate transaminase [EC:2.6.1.76]	17	12	14	19	0	2	3	0	8	13	11	9
K15786 aspartate-semialdehyde dehydrogenase [EC:1.2.1.-]	1	0	0	0	0	2	0	0	0	0	2	0
K20498 D-serine ammonia-lyase [EC:4.3.1.18]	2	2	0	0	1	0	1	0	0	0	0	1
ko00270 Cysteine and methionine metabolism	136148	134863	135435	136507	136745	137420	136655	137130	140832	141208	139817	139860
K00547 homocysteine S-methyltransferase [EC:2.1.1.10]	663	682	600	663	454	423	423	455	884	861	741	856
K00548 5-methyltetrahydrofolate--homocysteine methyltransferase [EC:2.1.1.13]	8752	8490	8722	8756	9047	9115	9238	9191	9750	9455	9498	9473
K00549 5-methyltetrahydropteroyltriglutamate--homocysteine methyltransferase [EC:2.1.1.14]	4885	4929	4876	5003	3146	3387	3120	3223	4303	4520	4335	4511
K00558 DNA (cytosine-5)-methyltransferase 1 [EC:2.1.1.37]	3000	3049	3137	3037	5781	6034	6013	6006	5676	5447	5471	5544
K00772 5'-methylthioadenosine phosphorylase [EC:2.4.2.28]	100	104	114	114	72	78	59	84	141	140	132	147
K00789 S-adenosylmethionine synthetase [EC:2.5.1.6]	4569	4549	4585	4822	4605	4545	4364	4428	4753	4870	4666	4677
K00797 spermidine synthase [EC:2.5.1.16]	3573	3591	3385	3518	2135	2097	1887	2094	1797	1759	1669	1721
K00816 kynurenine---oxoglutarate transaminase / cysteine-S-conjugate beta-lyase / glutamine---phenylpyruvate transaminase [EC:2.6.1.7 4.4.1.13 2.6.1.64]	0	0	0	0	0	0	0	0	0	0	1	0
K00826 branched-chain amino acid aminotransferase [EC:2.6.1.42]	3364	3225	3321	3308	4020	3688	3853	3828	3706	3768	3705	3625
K00832 aromatic-amino-acid transaminase [EC:2.6.1.57]	4599	4472	4392	4485	2742	2763	2799	2815	2957	2867	2938	3036
K00838 aromatic amino acid aminotransferase I / 2-aminoadipate transaminase [EC:2.6.1.57 2.6.1.39 2.6.1.27 2.6.1.5]	0	0	0	0	0	0	0	0	0	1	0	0
K00899 5-methylthioribose kinase [EC:2.7.1.100]	794	757	688	831	637	707	698	621	1106	1180	1034	1210
K01243 adenosylhomocysteine nucleosidase [EC:3.2.2.9]	1316	1216	1237	1342	1725	1655	1725	1685	1752	1740	1923	1808
K01251 adenosylhomocysteinase [EC:3.3.1.1]	2101	2254	2347	2234	3792	3988	3703	3764	3716	3771	3663	3584
K01505 1-aminocyclopropane-1-carboxylate deaminase [EC:3.5.99.7]	1155	1089	1002	1080	600	570	579	665	322	352	341	321
K01611 S-adenosylmethionine decarboxylase [EC:4.1.1.50]	2392	2220	2317	2268	1395	1415	1280	1362	1324	1416	1348	1333
K01740 O-acetylhomoserine (thiol)-lyase [EC:2.5.1.49]	4902	4900	5249	4953	6668	6795	7074	6918	5724	5624	5536	5354
K01760 cysteine-S-conjugate beta-lyase [EC:4.4.1.13]	1260	1241	1256	1308	802	879	874	852	2225	2238	2184	2242
K01761 methionine-gamma-lyase [EC:4.4.1.11]	33	17	26	19	94	134	111	106	267	255	222	233
K01919 glutamate--cysteine ligase [EC:6.3.2.2]	3641	3578	3483	3575	2210	2153	2038	2248	2446	2478	2536	2573
K01920 glutathione synthase [EC:6.3.2.3]	2012	2093	1998	2020	1250	1273	1269	1275	1310	1230	1180	1358
K05396 D-cysteine desulfhydrase [EC:4.4.1.15]	689	621	625	710	509	588	511	532	883	985	912	1012
K07173 S-ribosylhomocysteine lyase [EC:4.4.1.21]	720	706	722	737	534	536	522	530	602	658	600	702
K08963 methylthioribose-1-phosphate isomerase [EC:5.3.1.23]	1792	1744	1840	1799	1142	1180	1178	1162	1544	1551	1516	1635
K08964 methylthioribulose-1-phosphate dehydratase [EC:4.2.1.109]	1178	1136	1117	1183	781	678	662	732	687	744	683	730
K08965 2,3-diketo-5-methylthiopentyl-1-phosphate enolase [EC:5.3.2.5]	374	317	340	310	50	63	67	58	161	190	204	192
K08966 2-hydroxy-3-keto-5-methylthiopentenyl-1-phosphate phosphatase [EC:3.1.3.87]	1	0	2	0	0	0	0	0	0	0	0	0
K08967 1,2-dihydroxy-3-keto-5-methylthiopentene dioxygenase [EC:1.13.11.53 1.13.11.54]	635	712	620	726	513	555	453	503	551	513	469	544
K08968 L-methionine (R)-S-oxide reductase [EC:1.8.4.14]	1228	1262	1199	1180	1435	1560	1534	1530	1376	1356	1292	1275
K08969 aminotransferase [EC:2.6.1.-]	648	646	625	656	559	485	496	458	833	835	838	900
K09880 enolase-phosphatase E1 [EC:3.1.3.77]	1454	1325	1269	1358	791	764	830	786	887	799	867	850
K12339 S-sulfo-L-cysteine synthase (O-acetyl-L-serine-dependent) [EC:2.5.1.144]	1955	1957	1928	2010	1245	1233	1132	1160	1331	1200	1243	1314
K12960 5-methylthioadenosine/S-adenosylhomocysteine deaminase [EC:3.5.4.31 3.5.4.28]	2497	2335	2302	2298	1275	1307	1195	1285	1037	1055	1076	984
K13060 acyl homoserine lactone synthase [EC:2.3.1.184]	189	230	226	204	97	129	101	157	56	43	58	65
K13061 acyl homoserine lactone synthase [EC:2.3.1.184]	685	695	709	688	391	356	427	388	251	215	217	238
K14155 cysteine-S-conjugate beta-lyase [EC:4.4.1.13]	3799	3885	4001	3971	5788	5893	6057	5942	5877	6169	6249	6073
K16843 (S)-sulfolactate dehydrogenase [EC:1.1.1.310]	9	8	4	5	1	1	6	3	2	2	4	5
K16844 (2R)-3-sulfolactate dehydrogenase (NADP+) [EC:1.1.1.338]	7	4	3	5	0	0	0	0	0	3	0	0
K16845 (2R)-sulfolactate sulfo-lyase subunit alpha [EC:4.4.1.24]	3	8	2	5	4	1	4	5	8	14	13	8
K16846 (2R)-sulfolactate sulfo-lyase subunit beta [EC:4.4.1.24]	34	35	30	24	14	13	11	3	34	48	23	39
K17216 cystathionine beta-synthase (O-acetyl-L-serine) [EC:2.5.1.134]	0	1	0	0	0	0	0	0	0	0	0	1
K17398 DNA (cytosine-5)-methyltransferase 3A [EC:2.1.1.37]	16	10	13	15	42	45	34	35	5	8	11	4
K17462 putative AdoMet-dependent methyltransferase [EC:2.1.1.-]	45	33	37	36	14	7	7	10	29	20	29	22
K17950 L-cysteate sulfo-lyase [EC:4.4.1.25]	250	252	202	247	409	400	342	362	0	8	1	1
K19696 5'-methylthioinosine phosphorylase [EC:2.4.2.44]	997	912	938	948	521	418	469	542	326	314	265	317
ko00280 Valine, leucine and isoleucine degradation	100118	98754	96172	98193	78402	75896	75111	78377	66083	64843	64138	65077
K00020 3-hydroxyisobutyrate dehydrogenase [EC:1.1.1.31]	3819	3771	3646	3751	1823	1757	1864	2024	2096	1992	1959	1982
K00186 2-oxoisovalerate ferredoxin oxidoreductase, alpha subunit [EC:1.2.7.7]	2	2	0	3	0	0	2	0	3	6	3	7
K00187 2-oxoisovalerate ferredoxin oxidoreductase, beta subunit [EC:1.2.7.7]	0	0	1	1	1	2	0	0	7	10	7	15
K00188 2-oxoisovalerate ferredoxin oxidoreductase, delta subunit [EC:1.2.7.7]	0	0	0	0	0	0	0	0	0	2	2	1
K00253 isovaleryl-CoA dehydrogenase [EC:1.3.8.4]	1163	1258	1138	1176	657	655	580	679	442	387	371	343
K00263 leucine dehydrogenase [EC:1.4.1.9]	14	17	16	21	17	15	25	12	21	28	18	44
K01968 3-methylcrotonyl-CoA carboxylase alpha subunit [EC:6.4.1.4]	2713	2605	2290	2618	1305	1278	1257	1370	804	668	618	726
K01969 3-methylcrotonyl-CoA carboxylase beta subunit [EC:6.4.1.4]	2139	2018	1971	2011	1132	1014	1128	1238	697	609	587	637
K08683 3-hydroxyacyl-CoA dehydrogenase / 3-hydroxy-2-methylbutyryl-CoA dehydrogenase [EC:1.1.1.35 1.1.1.178]	0	0	0	1	0	0	0	0	0	0	0	0
K11410 short/branched chain acyl-CoA dehydrogenase [EC:1.3.99.12]	0	0	0	0	0	0	0	0	1	2	0	0
K13766 methylglutaconyl-CoA hydratase [EC:4.2.1.18]	3	4	3	3	3	1	4	1	4	6	3	4
K18661 malonyl-CoA/methylmalonyl-CoA synthetase [EC:6.2.1.-]	0	2	0	0	0	0	0	0	0	0	0	1
ko00290 Valine, leucine and isoleucine biosynthesis	60133	59525	59186	60762	53405	53901	52691	52867	59166	60533	59088	60512

K00053 ketol-acid reductoisomerase [EC:1.1.1.86]	3866	3730	3880	3924	4061	4120	3962	4071	4363	4431	4412	4490
K00835 valine--pyruvate aminotransferase [EC:2.6.1.66]	817	806	807	777	591	582	644	578	1124	1048	956	1128
K01687 dihydroxy-acid dehydratase [EC:4.2.1.9]	8688	8322	8541	8619	6945	7119	6911	7090	8859	9319	9037	9384
K01702 3-isopropylmalate dehydratase [EC:4.2.1.33]	1	0	0	0	0	0	0	0	0	0	1	0
K21360 3-isopropylmalate/methylthioalkylmalate dehydrogenase [EC:1.1.1.85 1.1.1.-]	0	0	0	0	1	0	0	0	0	0	0	0
ko00300 Lysine biosynthesis	57096	56875	57438	57773	66099	66061	66541	65933	65460	66424	65814	65405
K00215 4-hydroxy-tetrahydrodipicolinate reductase [EC:1.17.1.8]	2573	2480	2670	2483	3013	2926	2920	2925	2740	2726	2703	2757
K00290 saccharopine dehydrogenase (NAD+, L-lysine forming) [EC:1.5.1.7]	886	888	1022	899	2611	2589	2683	2681	1898	1907	1951	1821
K00293 saccharopine dehydrogenase (NADP+, L-glutamate forming) [EC:1.5.1.10]	0	0	2	0	0	0	0	0	0	0	0	1
K00674 2,3,4,5-tetrahydropyridine-2,6-dicarboxylate N-succinyltransferase [EC:2.3.1.117]	1486	1409	1370	1525	1589	1562	1515	1477	1603	1540	1563	1547
K00821 acetylornithine/N-succinyldiaminopimelate aminotransferase [EC:2.6.1.11 2.6.1.17]	3969	3790	4033	4230	4166	4199	4155	4092	4137	4117	4016	4109
K00841 aminotransferase [EC:2.6.1.-]	51	51	76	68	23	32	26	24	73	82	67	65
K01439 succinyl-diaminopimelate desuccinylase [EC:3.5.1.18]	4200	4116	3902	4232	1963	1900	1906	1850	3574	3814	3592	3793
K01586 diaminopimelate decarboxylase [EC:4.1.1.20]	3521	3526	3366	3564	3677	3768	3646	3791	4079	4198	4044	3920
K01714 4-hydroxy-tetrahydrodipicolinate synthase [EC:4.3.3.7]	7653	7602	7412	7583	5481	5507	5501	5630	5832	6314	6024	6051
K01778 diaminopimelate epimerase [EC:5.1.1.7]	3391	3264	3244	3354	2935	2940	2978	2942	2879	3109	2908	3027
K01928 UDP-N-acetylmuramoyl-L-alanyl-D-glutamate--2,6-diaminopimelate ligase [EC:6.3.2.13]	3073	3120	3241	3076	4560	4751	4650	4488	4568	4635	4478	4797
K01929 UDP-N-acetylmuramoyl-tripeptide--D-alanyl-D-alanine ligase [EC:6.3.2.10]	3391	3468	3536	3528	4813	5042	5129	4962	4166	4428	4240	4184
K03340 diaminopimelate dehydrogenase [EC:1.4.1.16]	1012	1010	1112	1043	1656	1695	1878	1730	1551	1637	1856	1678
K05822 tetrahydrodipicolinate N-acetyltransferase [EC:2.3.1.89]	70	50	64	63	76	76	33	34	78	73	66	74
K05823 N-acetyldiaminopimelate deacetylase [EC:3.5.1.47]	54	66	77	72	8	6	8	12	49	73	47	74
K05824 homoisocitrate dehydrogenase [EC:1.1.1.87]	3	0	0	1	0	0	0	0	9	13	13	9
K05825 2-aminoadipate transaminase [EC:2.6.1.-]	954	1032	1020	946	2515	2474	2406	2580	2427	2339	2483	2353
K05827 [lysine-biosynthesis-protein LysW]---L-2-aminoadipate ligase [EC:6.3.2.43]	0	0	0	0	0	0	0	0	2	3	0	0
K05828 LysW-gamma-L-alpha-aminoadipate/LysW-L-glutamate kinase [EC:2.7.2.-]	0	0	0	0	0	2	0	0	0	0	0	0
K05829 LysW-gamma-L-alpha-aminoadipyl-6-phosphate/LysW-L-glutamyl-5-phosphate reductase [EC:1.2.1.-]	2	2	0	0	0	4	3	0	1	2	3	1
K05830 LysW-gamma-L-lysine/LysW-L-ornithine aminotransferase [EC:2.6.1.-]	0	0	0	0	0	0	0	0	0	3	0	1
K05831 [amino group carrier protein]-lysine/ornithine hydrolase [EC:3.5.1.130 3.5.1.132]	0	0	2	0	0	0	0	0	2	0	0	1
K10206 LL-diaminopimelate aminotransferase [EC:2.6.1.83]	2008	2082	2134	2131	5095	5006	5048	5036	4180	3948	4045	3924
K14267 N-succinyldiaminopimelate aminotransferase [EC:2.6.1.17]	1897	1850	1768	1823	1042	942	1071	1109	606	564	626	546
K15792 murE/murF fusion protein [EC:6.3.2.13 6.3.2.10]	7	5	5	3	3	9	6	12	6	7	6	9
K16180 methylornithine synthase [EC:5.4.99.58]	4	5	2	5	1	0	3	3	0	0	2	3
K16181 pyrrolysine biosynthesis protein PylC	0	2	2	4	0	0	0	1	2	0	0	1
K16182 pyrrolysine biosynthesis protein PylD	0	0	0	1	0	0	0	2	0	0	0	0
K17450 homoaconitase [EC:4.2.1.-]	2	1	2	1	6	1	3	3	0	3	3	1
ko00310 Lysine degradation	64790	64535	62440	64129	48507	47720	47077	48598	50967	50225	49737	51131
K00137 aminobutyraldehyde dehydrogenase [EC:1.2.1.19]	2653	2680	2673	2777	1576	1569	1579	1506	1804	1643	1648	1625
K00468 lysine 2-monooxygenase [EC:1.13.12.2]	0	0	0	0	0	0	0	0	0	0	0	1
K00824 D-alanine transaminase [EC:2.6.1.21]	303	263	271	266	243	286	267	233	133	123	116	127
K01506 5-aminopentanamidase [EC:3.5.1.30]	6	9	12	13	3	4	3	4	0	0	0	0
K01582 lysine decarboxylase [EC:4.1.1.18]	2277	2322	2086	2428	1315	1493	1354	1251	3888	3932	3770	3918
K01843 lysine 2,3-aminomutase [EC:5.4.3.2]	1370	1425	1543	1341	4447	4328	4465	4320	2925	2854	3205	2965
K01844 beta-lysine 5,6-aminomutase alpha subunit [EC:5.4.3.3]	0	0	0	0	2	4	3	0	0	6	3	1
K03894 N2-citryl-N6-acetyl-N6-hydroxylysine synthase [EC:6.3.2.38]	0	2	3	0	3	2	3	5	3	6	3	6
K03895 aerobactin synthase [EC:6.3.2.39]	0	0	5	3	2	5	2	0	6	4	6	5
K03896 acetyl CoA:N6-hydroxylysine acetyl transferase [EC:2.3.1.102]	0	0	0	0	1	3	5	2	0	4	1	0
K03897 lysine N6-hydroxylase [EC:1.14.13.59]	0	0	1	0	3	0	0	4	6	14	3	9
K09251 putrescine aminotransferase [EC:2.6.1.82]	952	892	828	920	657	722	611	649	1272	1295	1186	1340
K13609 delta1-piperideine-2-carboxylate reductase [EC:1.5.1.21]	2009	1931	1872	1844	971	938	812	909	1270	1315	1210	1291
K15736 L-2-hydroxyglutarate oxidase [EC:1.1.5.-]	446	514	435	490	148	132	135	175	1074	1036	947	1114
K15737 glutarate hydroxylase [EC:1.14.11.-]	375	389	386	378	167	135	153	133	908	841	830	877
K18011 beta-lysine 5,6-aminomutase beta subunit [EC:5.4.3.3]	0	0	0	1	2	0	1	2	4	0	0	1
K18012 L-erythro-3,5-diaminohexanoate dehydrogenase [EC:1.4.1.11]	0	0	0	0	3	3	2	1	4	2	4	1
K18013 3-keto-5-aminohexanoate cleavage enzyme [EC:2.3.1.247]	33	33	34	33	10	5	6	8	52	54	42	64
K18014 3-aminobutyryl-CoA ammonia-lyase [EC:4.3.1.14]	0	0	0	0	1	4	1	2	2	0	3	2
K21672 2,4-diaminopentanoate dehydrogenase [EC:1.4.1.12 1.4.1.26]	352	395	378	380	86	88	98	97	245	298	257	286
K23385 D-ornithine/D-lysine decarboxylase [EC:4.1.1.116]	9	8	6	11	0	0	0	0	0	0	0	0
ko00220 Arginine biosynthesis	88438	87351	86788	87091	82048	81614	81599	82787	73934	73370	73133	73303
K00145 N-acetyl-gamma-glutamyl-phosphate reductase [EC:1.2.1.38]	3863	3704	3705	3740	4147	4166	4168	4274	3582	3664	3889	3713
K00611 ornithine carbamoyltransferase [EC:2.1.3.3]	4632	4603	4223	4517	2496	2427	2461	2531	2931	3050	2917	2933
K00619 amino-acid N-acetyltransferase [EC:2.3.1.1]	17	31	19	21	4	5	8	2	33	40	26	44
K00620 glutamate N-acetyltransferase / amino-acid N-acetyltransferase [EC:2.3.1.35 2.3.1.1]	2449	2332	2459	2494	1268	1313	1207	1291	1292	1226	1303	1266
K00818 acetylornithine aminotransferase [EC:2.6.1.11]	0	3	1	1	3	1	3	1	2	1	1	0
K00930 acetylglutamate kinase [EC:2.7.2.8]	2976	3045	2974	2812	3129	3150	3037	3011	3040	2852	2813	2924
K01438 acetylornithine deacetylase [EC:3.5.1.16]	5644	5532	5365	5417	5429	5466	5415	5537	4210	4031	3917	3952
K01457 allophanate hydrolase [EC:3.5.1.54]	401	333	316	417	532	607	527	569	0	0	2	0
K01476 arginase [EC:3.5.3.1]	330	351	343	337	207	222	250	209	640	626	603	652
K01478 arginine deiminase [EC:3.5.3.6]	2289	2248	2325	2369	1391	1330	1307	1307	979	930	902	1032
K01941 urea carboxylase [EC:6.3.4.6]	776	742	706	764	1071	1087	1083	1123	1	3	4	4
K14677 aminoacylase [EC:3.5.1.14]	1	0	3	1	0	1	1	0	0	1	0	2
K14682 amino-acid N-acetyltransferase [EC:2.3.1.1]	2813	2674	2591	2609	1617	1542	1486	1567	1753	1775	1615	1774
K22477 N-acetylglutamate synthase [EC:2.3.1.1]	150	139	122	126	29	20	19	29	116	119	136	117
ko00330 Arginine and proline metabolism	122379	121549	116445	119568	85355	84913	82894	86883	84914	83852	82967	84481
K00147 glutamate-5-semialdehyde dehydrogenase [EC:1.2.1.41]	3111	3201	3251	3186	4225	4097	4268	4323	4220	4145	4361	4216
K00286 pyrroline-5-carboxylate reductase [EC:1.5.1.2]	1964	1999	1944	1992	2477	2654	2443	2434	2616	2680	2738	2650
K00316 spermidine dehydrogenase [EC:1.5.99.6]	2957	3020	2903	2924	1666	1437	1342	1524	947	856	817	896
K00318 proline dehydrogenase [EC:1.5.5.2]	8	14	3	7	2	0	2	0	2	0	0	0
K00472 prolyl 4-hydroxylase [EC:1.14.11.2]	1	0	0	0	0	0	0	0	0	0	0	0
K00657 diamine N-acetyltransferase [EC:2.3.1.57]	1602	1692	1729	1763	1758	1840	1728	1747	1676	1784	1627	1624
K00673 arginine N-succinyltransferase [EC:2.3.1.109]	2463	2353	2273	2345	1256	1318	1306	1349	1934	2052	1914	2026
K00819 ornithine--oxo-acid transaminase [EC:2.6.1.13]	137	149	175	156	203	235	227	179	14	35	27	27
K00840 succinylornithine aminotransferase [EC:2.6.1.81]	3157	3003	3022	3106	1624	1658	1585	1761	2566	2339	2474	2452
K00931 glutamate 5-kinase [EC:2.7.2.11]	3625	3469	3519	3619	4005	3881	4175	4081	3738	3631	3897	3850
K01259 proline iminopeptidase [EC:3.4.11.5]	1733	1695	1748	1711	930	972	885	975	570	524	558	501
K01426 amidase [EC:3.5.1.4]	9167	9061	8492	8895	4978	4956	4644	5208	3233	3275	3176	3276
K01470 creatinine amidohydrolase [EC:3.5.2.10]	974	931	903	893	289	331	358	338	1348	1320	1252	1370
K01473 N-methylhydantoinase A [EC:3.5.2.14]	47	39	65	46	2	2	3	1	19	19	15	16
K01474 N-methylhydantoinase B [EC:3.5.2.14]	53	34	31	36	2	2	1	0	15	29	5	23
K01480 agmatinase [EC:3.5.3.11]	847	956	885	946	538	692	580	530	1294	1352	1290	1334
K01484 succinylarginine dihydrolase [EC:3.5.3.23]	2102	2175	1935	2222	1077	1177	1082	1133	2202	2309	2288	2375
K01581 ornithine decarboxylase [EC:4.1.1.17]	2931	2878	2973	3051	2098	2211	2148	2164	2776	2756	2679	2860
K01584 arginine decarboxylase [EC:4.1.1.19]	3205	3099	3012	3142	1650	1642	1540	1617	1275	1131	1263	1244
K01585 arginine decarboxylase [EC:4.1.1.19]	6227	6173	6159	6006	7212	7223	7308	7277	6418	6347	6530	6231
K01750 ornithine cyclodeaminase [EC:4.3.1.12]	1342	1356	1182	1287	642	632	602	731	477	474	422	509
K01777 proline racemase [EC:5.1.1.4]	66	71	80	66	84	90	93	109	111	106	112	121
K02626 arginine decarboxylase [EC:4.1.1.19]	8	5	9	7	0	2	0	0	1	4	2	0
K05526 succinylglutamate desuccinylase [EC:3.5.1.96]	1654	1639	1477	1561	817	806	804	774	1575	1724	1677	1790
K06447 succinylglutamic semialdehyde dehydrogenase [EC:1.2.1.71]	3752	3713	3505	3659	1914	1861	1884	1970	2955	2850	2897	3053
K08660 cytosolic nonspecific dipeptidase [EC:3.4.13.18]	1	0	0	0	0	0	0	0	0	0	0	0
K09123 cis-L-3-hydroxyproline dehydratase [EC:4.2.1.171]	1994	2004	1767	1902	963	933	925	1109	635	561	561	634
K09470 gamma-glutamylputrescine synthase [EC:6.3.1.11]	693	806	738	769	505	496	556	487	1067	1126	1094	1052
K09471 gamma-glutamylputrescine oxidase [EC:1.4.3.-]	6055	5978	5587	5777	2773	2866	2730	2892	3493	3458	3348	3370
K09472 4-(gamma-glutamylamino)butanal dehydrogenase [EC:1.2.1.99]	2648	2561	2434	2563	1299	1292	1154	1420	1722	1843	1677	1762
K09473 gamma-glutamyl-gamma-aminobutyrate hydrolase [EC:3.5.1.94]	287	283	305	278	69	76	87	78	589	684	658	629
K10536 agmatine deiminase [EC:3.5.3.12]	5492	5545	5061	5239	3165	3096	2936	3313	1783	1650	1584	1724
K10793 D-proline reductase (dithiol) PrdA [EC:1.21.4.1]	11	22	16	25	1	2	0	1	11	0	3	1
K10794 D-proline reductase (dithiol) PrdB [EC:1.21.4.1]	7	8	5	7	0	0	0	0	0	0	0	0
K12251 N-carbamoylputrescine amidase [EC:3.5.1.53]	3617	3497	3424	3406	3464	3402	3406	3615	2163	1835	1981	2032
K12252 arginine:pyruvate transaminase [EC:2.6.1.84]	2127	2046	1890	1988	1088	1051	958	1180	680	647	625	686
K12253 5-guanidino-2-oxopentanoate decarboxylase [EC:4.1.1.75]	2613	2606	2318	2444	1402	1225	1218	1324	857	754	780	796
K12254 4-guanidinobutyraldehyde dehydrogenase / NAD-dependent aldehyde dehydrogenase [EC:1.2.1.54 1.2.1.-]	2719	2717	2534	2661	1508	1569	1444	1547	887	816	865	853
K12255 guanidinobutyrase [EC:3.5.3.7]	1698	1767	1657	1755	765	794	701	772	1187	1137	1193	1104
K12256 putrescine---pyruvate transaminase [EC:2.6.1.113]	229	220	242	224	120	108	100	128	69	85	74	71
K12658 4-hydroxyproline epimerase [EC:5.1.1.8]	1522	1490	1187	1321	778	757	612	713	488	368	376	399
K13747 carboxynorspermidine decarboxylase [EC:4.1.1.96]	718	772	904	826	2351	2266	2464	2186	1701	1699	1737	1594
K21061 D-hydroxyproline dehydrogenase subunit beta [EC:1.5.99.-]	962	887	738	861	438	409	382	447	262	246	242	299
K21062 1-pyrroline-4-hydroxy-2-carboxylate deaminase [EC:3.5.4.22]	1407	1364	1276	1314	692	700	694	778	481	408	335	417
K22548 trans-L-3-hydroxyproline dehydratase [EC:4.2.1.77]	1301	1265	1169	1247	734	643	586	774	429	339	356	391
K22549 D-hydroxyproline dehydrogenase subunit alpha [EC:1.5.99.-]	1149	1075	842	1018	512	462	416	622	311	301	284	329
K22550 D-hydroxyproline dehydrogenase subunit gamma	335	315	228	286	155	134	129	155	78	81	96	87
K23121 trans-4-hydroxy-L-proline dehydratase [EC:4.2.1.172]	662	614	635	586	447	494	506	475	713	755	685	756

ko00340 Histidine metabolism	60479	59535	59990	59197	62077	61787	62134	62424	52445	51474	51374	51745
K00013 histidinol dehydrogenase [EC:1.1.1.23]	2886	2775	2704	2896	2688	2738	2759	2880	2762	2862	2734	2838
K00603 glutamate formiminotransferase / 5-formyltetrahydrofolate cyclo-ligase [EC:2.1.2.5 6.3.3.2]	671	669	766	679	2041	2184	2185	2182	1583	1536	1581	1478
K00765 ATP phosphoribosyltransferase [EC:2.4.2.17]	2507	2343	2433	2365	2773	2844	2825	2874	2649	2733	2589	2667
K00817 histidinol-phosphate aminotransferase [EC:2.6.1.9]	7709	7663	7838	7675	8330	8147	8236	8130	6354	6206	6285	6231
K01089 imidazoleglycerol-phosphate dehydratase / histidinol-phosphatase [EC:4.2.1.19 3.1.3.15]	1369	1390	1460	1370	2724	2567	2822	2592	2418	2451	2545	2539
K01468 imidazolonepropionase [EC:3.5.2.7]	3878	3650	3709	3931	4276	4223	4403	4414	3847	3604	3650	3629
K01479 formiminoglutamase [EC:3.5.3.8]	1815	1699	1721	1699	903	855	814	811	1166	1054	1114	1260
K01496 phosphoribosyl-AMP cyclohydrolase [EC:3.5.4.19]	723	760	748	719	408	394	457	428	294	270	241	325
K01523 phosphoribosyl-ATP pyrophosphohydrolase [EC:3.6.1.31]	598	593	565	579	343	336	334	403	232	244	201	214
K01590 histidine decarboxylase [EC:4.1.1.22]	12	5	0	2	3	2	3	0	17	17	13	12
K01663 imidazole glycerol-phosphate synthase [EC:4.3.2.10]	0	0	1	0	0	0	0	0	0	0	0	0
K01693 imidazoleglycerol-phosphate dehydratase [EC:4.2.1.19]	1305	1271	1255	1142	681	631	662	735	615	617	555	619
K01712 urocanate hydratase [EC:4.2.1.49]	5722	5474	5473	5440	6589	6727	6833	6651	5795	5866	5642	5721
K01745 histidine ammonia-lyase [EC:4.3.1.3]	8324	8421	8336	8264	9886	9874	9767	9966	8177	7973	8036	8016
K01814 phosphoribosylformimino-5-aminoimidazole carboxamide ribotide isomerase [EC:5.3.1.16]	2363	2406	2548	2385	2522	2483	2418	2545	2494	2317	2438	2459
K02500 imidazole glycerol-phosphate synthase subunit HisF [EC:4.3.2.10]	3360	3295	3402	3348	3250	3334	3384	3172	2792	2729	2798	2790
K02501 imidazole glycerol-phosphate synthase subunit HisH [EC:4.3.2.10]	2696	2584	2685	2615	2605	2544	2557	2564	2305	2162	2172	2305
K02502 ATP phosphoribosyltransferase regulatory subunit	1479	1342	1329	1282	698	649	613	686	740	829	793	784
K04486 histidinol-phosphatase (PHP family) [EC:3.1.3.15]	3182	3250	3462	3204	4102	4281	4268	4165	3607	3425	3470	3391
K05602 histidinol-phosphatase [EC:3.1.3.15]	0	0	0	1	0	0	0	0	0	0	0	1
K05603 formimidoylglutamate deiminase [EC:3.5.3.13]	691	671	572	655	591	583	542	611	102	132	123	112
K11755 phosphoribosyl-ATP pyrophosphohydrolase / phosphoribosyl-AMP cyclohydrolase [EC:3.6.1.31 3.5.4.19]	1087	1062	1086	1079	1699	1681	1701	1658	1595	1669	1775	1661
K13990 glutamate formiminotransferase / formiminotetrahydrofolate cyclodeaminase [EC:2.1.2.5 4.3.1.4]	5	2	3	2	7	9	10	12	0	1	3	2
K14152 phosphoribosyl-ATP pyrophosphohydrolase / phosphoribosyl-AMP cyclohydrolase / histidinol dehydrogenase [EC:3.6.1.31 3.5.4.19 1.1.1.23]	0	0	0	0	0	0	0	1	0	0	0	0
K17363 urocanate reductase [EC:1.3.99.33]	12	21	19	22	22	17	25	19	32	43	46	42
ko00350 Tyrosine metabolism	63187	61811	60724	62184	42265	41713	40887	42573	46100	46102	45123	46338
K00055 aryl-alcohol dehydrogenase [EC:1.1.1.90]	406	379	402	465	104	135	95	139	881	891	833	844
K00151 5-carboxymethyl-2-hydroxymuconic-semialdehyde dehydrogenase [EC:1.2.1.60]	2534	2420	2364	2407	1194	1183	1010	1175	1775	1746	1655	1830
K00450 gentisate 1,2-dioxygenase [EC:1.13.11.4]	1483	1452	1354	1397	726	707	746	830	472	464	416	396
K00451 homogentisate 1,2-dioxygenase [EC:1.13.11.5]	393	434	343	340	189	174	192	215	136	125	109	134
K00455 3,4-dihydroxyphenylacetate 2,3-dioxygenase [EC:1.13.11.15]	1658	1562	1439	1461	765	724	742	793	1088	1163	1061	1141
K00457 4-hydroxyphenylpyruvate dioxygenase [EC:1.13.11.27]	5276	5060	4895	4938	2674	2608	2324	2809	2760	2716	2748	2897
K00483 4-hydroxyphenylacetate 3-monooxygenase [EC:1.14.14.9]	3388	3198	3008	3277	1796	1609	1542	1705	2360	2156	1982	2181
K00484 flavin reductase (NADH) [EC:1.5.1.36]	584	571	486	500	234	242	273	281	515	534	479	541
K01555 fumarylacetoacetase [EC:3.7.1.2]	1681	1512	1501	1683	902	848	762	854	524	449	449	457
K01557 acylpyruvate hydrolase [EC:3.7.1.5]	1	0	0	0	0	0	0	0	0	0	0	0
K01593 aromatic-L-amino-acid/L-tryptophan decarboxylase [EC:4.1.1.28 4.1.1.105]	0	1	0	0	0	0	0	0	0	0	0	0
K01668 tyrosine phenol-lyase [EC:4.1.99.2]	306	280	273	283	359	405	407	360	13	14	5	8
K01800 maleylacetoacetate isomerase [EC:5.2.1.2]	1700	1716	1539	1668	919	833	803	929	556	464	459	513
K01826 5-carboxymethyl-2-hydroxymuconate isomerase [EC:5.3.3.10]	1071	1058	949	1044	495	546	491	537	587	609	574	636
K02509 2-oxo-hept-3-ene-1,7-dioate hydratase [EC:4.2.1.-]	1720	1620	1518	1636	853	839	774	958	1117	1075	1054	1123
K02510 4-hydroxy-2-oxoheptanedioate aldolase [EC:4.1.2.52]	2182	2003	1932	2165	1061	1147	1103	1118	1196	1210	1194	1248
K05921 5-oxopent-3-ene-1,2,5-tricarboxylate decarboxylase / 2-hydroxyhepta-2,4-diene-1,7-dioate isomerase [EC:4.1.1.68 5.3.3.-]	2889	2653	2638	2757	1384	1475	1328	1487	1862	1784	1707	1825
K10219 2-hydroxy-4-carboxymuconate semialdehyde hemiacetal dehydrogenase [EC:1.1.1.312]	8	5	7	11	0	0	0	0	4	6	4	4
K16165 fumarylpyruvate hydrolase [EC:3.7.1.20]	150	140	146	142	111	89	110	116	64	67	80	76
K16901 anthranilate 3-monooxygenase (FAD) / 4-hydroxyphenylacetate 3-monooxygenase [EC:1.14.14.8 1.14.14.9]	1	0	0	0	0	0	0	2	0	0	0	0
K22330 tyrosine decarboxylase [EC:4.1.1.25]	97	104	100	124	38	35	28	30	107	159	119	133
K23109 aromatic 2-oxoacid reductase [EC:1.1.1.110]	3	5	12	9	3	3	2	0	16	20	14	18
ko00360 Phenylalanine metabolism	67599	67361	65044	66281	47773	47165	46342	48668	56908	57652	56287	58293
K00146 phenylacetaldehyde dehydrogenase [EC:1.2.1.39]	3074	3228	2751	2991	1383	1316	1355	1467	2754	2743	2608	2749
K00285 D-amino-acid dehydrogenase [EC:1.4.5.1]	5374	5202	5358	5270	3033	2959	2812	3147	3184	3145	3203	3362
K00500 phenylalanine-4-hydroxylase [EC:1.14.16.1]	1235	1169	1118	1207	678	690	622	661	367	382	315	350
K01451 hippurate hydrolase [EC:3.5.1.32]	4297	4273	4089	4137	2111	1923	1897	2168	2794	2838	2619	2805
K01666 4-hydroxy 2-oxovalerate aldolase [EC:4.1.3.39]	424	414	433	447	96	102	116	85	812	819	761	789
K01912 phenylacetate-CoA ligase [EC:6.2.1.30]	2593	2821	2790	2674	5548	5595	5892	5643	4880	4983	4988	5098
K02554 2-keto-4-pentenoate hydratase [EC:4.2.1.80]	413	442	449	407	324	419	357	333	583	580	637	651
K02609 ring-1,2-phenylacetyl-CoA epoxidase subunit PaaA [EC:1.14.13.149]	388	358	346	401	95	79	94	109	744	692	696	750
K02610 ring-1,2-phenylacetyl-CoA epoxidase subunit PaaB	128	119	100	103	46	26	29	39	223	216	216	217
K02611 ring-1,2-phenylacetyl-CoA epoxidase subunit PaaC [EC:1.14.13.149]	270	274	276	307	86	86	66	87	512	585	558	657
K02612 ring-1,2-phenylacetyl-CoA epoxidase subunit PaaD	174	190	195	191	43	52	53	54	322	421	430	413
K02613 ring-1,2-phenylacetyl-CoA epoxidase subunit PaaE	310	428	410	390	101	99	113	102	726	826	860	895
K02614 acyl-CoA thioesterase [EC:3.1.2.-]	1143	1167	1155	1101	1313	1300	1305	1427	1301	1313	1249	1267
K02615 3-oxo-5,6-didehydrosuberyl-CoA/3-oxoadipyl-CoA thiolase [EC:2.3.1.223 2.3.1.174]	430	473	382	441	134	106	114	133	885	900	899	923
K02618 oxepin-CoA hydrolase / 3-oxo-5,6-dehydrosuberyl-CoA semialdehyde dehydrogenase [EC:3.3.2.12 1.2.1.91]	671	634	647	693	194	211	207	184	1352	1424	1402	1494
K03782 catalase-peroxidase [EC:1.11.1.21]	1258	1181	1142	1263	789	896	772	806	1641	1801	1708	1710
K05708 3-phenylpropionate/trans-cinnamate dioxygenase subunit alpha [EC:1.14.12.19]	0	0	0	0	11	3	8	4	4	1	3	1
K05709 3-phenylpropionate/trans-cinnamate dioxygenase subunit beta [EC:1.14.12.19]	0	0	0	0	2	2	3	3	0	1	1	1
K05710 3-phenylpropionate/trans-cinnamate dioxygenase ferredoxin component	0	1	0	0	3	4	4	3	1	0	2	4
K05711 2,3-dihydroxy-2,3-dihydrophenylpropionate dehydrogenase [EC:1.3.1.87]	0	0	0	0	10	10	4	5	4	2	6	2
K05712 3-(3-hydroxy-phenyl)propionate hydroxylase [EC:1.14.13.127]	601	533	477	546	157	190	148	178	1041	1126	1068	1154
K05713 2,3-dihydroxyphenylpropionate 1,2-dioxygenase [EC:1.13.11.16]	352	356	276	304	101	81	98	84	683	757	658	745
K05714 2-hydroxy-6-oxonona-2,4-dienedioate hydrolase [EC:3.7.1.14]	283	293	266	314	98	81	84	100	648	690	614	702
K10797 2-enoate reductase [EC:1.3.1.31]	1240	1260	1193	1190	199	187	213	189	1244	1330	1284	1369
K15866 2-(1,2-epoxy-1,2-dihydrophenyl)acetyl-CoA isomerase [EC:5.3.3.18]	1225	1220	1112	1080	588	529	521	643	866	851	866	872
K18356 phenylglyoxylate dehydrogenase beta subunit [EC:1.2.1.58]	1	2	0	1	0	0	0	0	5	3	7	13
ko00380 Tryptophan metabolism	67727	67568	65289	66791	46993	46039	45017	47573	42448	41918	41277	42338
K00452 3-hydroxyanthranilate 3,4-dioxygenase [EC:1.13.11.6]	1	0	0	0	0	2	0	0	0	0	1	0
K00453 tryptophan 2,3-dioxygenase [EC:1.13.11.11]	1198	1272	1328	1245	689	730	729	786	355	366	356	383
K01556 kynureninase [EC:3.7.1.3]	450	470	445	444	238	240	210	217	133	163	127	138
K01667 tryptophanase [EC:4.1.99.1]	992	1068	1125	1117	3054	2955	3140	3031	1964	1901	2035	1842
K03392 aminocarboxymuconate-semialdehyde decarboxylase [EC:4.1.1.45]	14	9	16	22	3	0	2	2	4	0	3	9
K04103 indolepyruvate decarboxylase [EC:4.1.1.74]	693	702	727	670	203	190	229	198	1443	1568	1374	1535
K10217 aminomuconate-semialdehyde/2-hydroxymuconate-6-semialdehyde dehydrogenase [EC:1.2.1.32 1.2.1.85]	1	0	0	0	0	0	0	0	0	0	0	0
K14264 kynurenine aminotransferase [EC:2.6.1.7]	0	0	0	0	0	0	0	1	0	0	0	0
K20807 nitrile hydratase subunit beta [EC:4.2.1.84]	0	0	0	0	0	0	0	0	1	0	0	0
K21801 indoleacetamide hydrolase [EC:3.5.1.-]	1	0	0	0	0	0	0	0	0	0	0	0
K23384 indoleacetate decarboxylase [EC:4.1.1.115]	6	6	7	9	0	0	0	0	0	2	1	0
ko00400 Phenylalanine, tyrosine and tryptophan biosynthesis	82297	82245	82864	82360	80409	79695	80181	79849	78406	78029	77696	78041
K00014 shikimate dehydrogenase [EC:1.1.1.25]	4001	3925	4135	4063	3512	3324	3485	3433	4683	4655	4415	4703
K00210 prephenate dehydrogenase [EC:1.3.1.12]	484	593	611	550	1720	1747	1773	1642	1302	1179	1262	1227
K00220 cyclohexadieny/prephenate dehydrogenase [EC:1.3.1.43 1.3.1.12]	2	0	1	1	3	0	2	3	2	3	4	1
K00766 anthranilate phosphoribosyltransferase [EC:2.4.2.18]	2809	2797	2832	2778	3137	3063	3089	3018	2440	2233	2427	2257
K00800 3-phosphoshikimate 1-carboxyvinyltransferase [EC:2.5.1.19]	5576	5694	5740	5617	5392	5341	5479	5349	4614	4702	4753	4710
K00891 shikimate kinase [EC:2.7.1.71]	2326	2469	2468	2371	2276	2358	2300	2480	2640	2612	2515	2510
K01609 indole-3-glycerol phosphate synthase [EC:4.1.1.48]	2147	2194	2150	2113	2385	2239	2417	2510	1794	1763	1912	1820
K01626 3-deoxy-7-phosphoheptulonate synthase [EC:2.5.1.54]	8495	8301	8246	8294	4738	4501	4268	4534	5044	5246	4836	5059
K01656 anthranilate synthase / indole-3-glycerol phosphate synthase [EC:4.1.3.27 4.1.1.48]	0	0	0	0	0	1	0	0	0	0	0	0
K01657 anthranilate synthase component I [EC:4.1.3.27]	5953	6024	6120	5943	5535	5595	5523	5413	5197	4925	4909	4982
K01658 anthranilate synthase component II [EC:4.1.3.27]	2717	2705	2636	2690	2255	2308	2218	2346	1660	1627	1614	1567
K01713 cyclohexadienyl dehydratase [EC:4.2.1.51 4.2.1.91]	1488	1454	1382	1433	714	631	634	695	1043	1112	1022	1021
K01735 3-dehydroquinate synthase [EC:4.2.3.4]	3884	3778	3727	3933	4281	4278	4111	4072	3840	3881	3949	3808
K01736 chorismate synthase [EC:4.2.3.5]	3778	3757	3785	3685	3912	4003	4219	3944	3782	3981	3958	4017
K01817 phosphoribosylanthranilate isomerase [EC:5.3.1.24]	1294	1299	1349	1259	1575	1536	1605	1633	1362	1326	1407	1352
K03785 3-dehydroquinate dehydratase I [EC:4.2.1.10]	616	615	628	653	476	487	533	458	804	989	865	930
K03786 3-dehydroquinate dehydratase II [EC:4.2.1.10]	2215	2284	2358	2375	2111	2065	2051	2081	1753	1788	1743	1775
K03856 3-deoxy-7-phosphoheptulonate synthase [EC:2.5.1.54]	646	605	667	619	206	224	246	213	744	785	659	743
K04092 chorismate mutase [EC:5.4.99.5]	1	0	2	1	3	1	0	0	4	10	6	9
K04093 chorismate mutase [EC:5.4.99.5]	920	980	844	933	538	522	534	504	314	277	257	310
K04516 chorismate mutase [EC:5.4.99.5]	676	727	806	726	2316	2326	2246	2317	1689	1525	1738	1578
K04517 prephenate dehydrogenase [EC:1.3.1.12]	631	620	627	618	205	211	172	182	615	734	637	621
K04518 prephenate dehydratase [EC:4.2.1.51]	560	658	751	655	1974	1931	2037	1926	1422	1411	1429	1473
K05358 quinate dehydrogenase (quinone) [EC:1.1.5.8]	1340	1300	1259	1230	802	901	823	811	1722	1761	1619	1750
K05359 arogenate/prephenate dehydratase [EC:4.2.1.91 4.2.1.51]	0	0	0	0	0	0	0	0	2	2	0	4
K05887 quinate/shikimate dehydrogenase [EC:1.1.1.282]	36	54	49	36	89	70	89	65	98	88	98	116
K06208 chorismate mutase [EC:5.4.99.5]	0	1	0	2	0	0	0	0	6	12	5	5
K06209 chorismate mutase [EC:5.4.99.5]	268	237	261	275	60	85	85	89	226	248	262	289
K11646 3-dehydroquinate synthase II [EC:1.4.1.24]	15	16	12	19	16	2	5	6	21	25	10	27
K13497 anthranilate synthase/phosphoribosyltransferase [EC:4.1.3.27 2.4.2.18]	969	943	912	966	572	623	600	602	1301	1384	1442	1367
K13498 indole-3-glycerol phosphate synthase / phosphoribosylanthranilate isomerase [EC:4.1.1.48 5.3.1.24]	811	744	756	813	546	540	595	545	1107	1076	971	1128
K13503 anthranilate synthase [EC:4.1.3.27]	0	0	0	0	2	7	1	2	2	6	3	0

K13829 shikimate kinase / 3-dehydroquinate synthase [EC:2.7.1.71 4.2.3.4]	10	15	8	15	26	14	21	19	25	25	24	32
K13832 3-dehydroquinate dehydratase / shikimate dehydrogenase [EC:4.2.1.10 1.1.1.25]	0	2	2	1	2	2	3	0	1	0	0	1
K13853 3-deoxy-7-phosphoheptulonate synthase / chorismate mutase [EC:2.5.1.54 5.4.99.5]	0	0	2	2	0	0	1	0	0	1	2	1
K14170 chorismate mutase / prephenate dehydratase [EC:5.4.99.5 4.2.1.51]	3001	2781	2992	2825	1598	1754	1517	1626	2205	2038	2000	2037
K14187 chorismate mutase / prephenate dehydrogenase [EC:5.4.99.5 1.3.1.12]	772	691	649	743	487	568	544	502	964	996	968	1025
K15652 3-dehydroshikimate dehydratase [EC:4.2.1.118]	0	0	1	0	1	0	0	0	0	0	0	0
K17749 3-benzylmalate isomerase [EC:4.2.1.-]	1	0	0	0	0	0	0	0	0	0	0	0
K17750 3-benzylmalate dehydrogenase [EC:1.1.1.-]	0	0	0	0	0	0	0	0	0	1	0	0
Metabolism of other amino acids	285297	284883	280899	284452	282862	283029	281654	282129	282463	280216	279210	280375
ko00410 beta-Alanine metabolism	54483	53513	52036	52952	36094	34911	33537	35631	33584	33314	32046	33284
K01579 aspartate 1-decarboxylase [EC:4.1.1.11]	968	1037	1032	990	1278	1240	1193	1165	1013	1069	1076	1027
K01918 pantoate--beta-alanine ligase [EC:6.3.2.1]	2715	2495	2689	2623	3123	3153	3021	3109	2737	2592	2593	2551
K09722 4-phosphopantoate---beta-alanine ligase [EC:6.3.2.36]	1	0	0	1	1	0	0	0	2	2	2	3
K15372 taurine---2-oxoglutarate transaminase [EC:2.6.1.55]	852	915	766	875	512	591	496	535	1206	1209	1140	1240
ko00430 Taurine and hypotaurine metabolism	30793	30662	29791	30251	25650	25797	25113	26040	23162	22413	22532	23041
K00681 gamma-glutamyltranspeptidase / glutathione hydrolase [EC:2.3.2.2 3.4.19.13]	9341	8938	8847	9001	5414	5263	5126	5548	4207	4148	4127	4293
K03851 taurine-pyruvate aminotransferase [EC:2.6.1.77]	10	6	16	9	3	5	0	2	10	7	4	4
K03852 sulfoacetaldehyde acetyltransferase [EC:2.3.3.15]	8	18	6	7	7	5	2	6	23	20	20	39
K15373 sulfoacetaldehyde reductase [EC:1.1.1.313]	497	507	468	509	281	313	344	323	643	627	611	763
ko00440 Phosphonate and phosphinate metabolism	17247	17191	16246	16839	10181	9988	9611	10350	14425	14444	13863	14440
K01841 phosphoenolpyruvate phosphomutase [EC:5.4.2.9]	132	132	142	146	74	64	66	63	83	85	108	71
K03430 2-aminoethylphosphonate-pyruvate transaminase [EC:2.6.1.37]	2348	2353	2331	2360	1232	1341	1253	1410	1969	2037	1902	1914
K03823 phosphinothricin acetyltransferase [EC:2.3.1.183]	2141	2223	2057	2159	1490	1407	1447	1485	1689	1680	1599	1732
K05306 phosphonoacetaldehyde hydrolase [EC:3.11.1.1]	1963	1815	1677	1838	1005	1000	1026	1046	1320	1424	1312	1387
K05780 alpha-D-ribose 1-methylphosphonate 5-triphosphate synthase subunit PhnL [EC:2.7.8.37]	1001	964	912	976	425	424	385	456	1000	962	919	1019
K06162 alpha-D-ribose 1-methylphosphonate 5-triphosphate diphosphatase [EC:3.6.1.63]	1845	1853	1663	1690	824	774	704	795	1543	1395	1414	1547
K06163 alpha-D-ribose 1-methylphosphonate 5-phosphate C-P lyase [EC:4.7.1.1]	1651	1657	1628	1612	786	811	741	844	1254	1251	1214	1243
K06164 alpha-D-ribose 1-methylphosphonate 5-triphosphate synthase subunit PhnI [EC:2.7.8.37]	1761	1656	1558	1683	822	723	665	923	1422	1451	1302	1410
K06165 alpha-D-ribose 1-methylphosphonate 5-triphosphate synthase subunit PhnH [EC:2.7.8.37]	1260	1315	1096	1263	589	523	511	602	898	891	788	858
K06166 alpha-D-ribose 1-methylphosphonate 5-triphosphate synthase subunit PhnG [EC:2.7.8.37]	963	900	835	889	488	372	421	398	706	692	695	688
K06167 phosphoribosyl 1,2-cyclic phosphate phosphodiesterase [EC:3.1.4.55]	2000	2144	2173	2030	2270	2357	2183	2149	2220	2221	2220	2209
K09459 phosphonopyruvate decarboxylase [EC:4.1.1.82]	72	66	50	64	35	30	41	27	109	107	124	122
K09994 (aminoalkyl)phosphonate N-acetyltransferase [EC:2.3.1.280]	12	25	16	18	3	6	7	6	43	44	61	84
K19670 phosphonoacetate hydrolase [EC:3.11.1.2]	72	65	64	82	127	138	146	128	105	92	114	89
K20859 phosphoribosyl 1,2-cyclic phosphate 1,2-diphosphodiesterase [EC:3.1.4.57]	24	23	44	29	8	11	10	16	57	103	87	56
ko00450 Selenocompound metabolism	49483	49507	49234	50116	48072	49879	48981	48250	55905	55802	55947	55855
K00384 thioredoxin reductase (NADPH) [EC:1.8.1.9]	5048	5103	5254	5193	4075	4326	4004	3898	5206	5283	5317	5310
K01008 selenide, water dikinase [EC:2.7.9.3]	2726	2788	2603	2646	1346	1332	1280	1444	1713	1789	1707	1815
K01042 L-seryl-tRNA(Ser) seleniumtransferase [EC:2.9.1.1]	2558	2671	2455	2469	1542	1562	1633	1584	2588	2805	2590	2854
K01874 methionyl-tRNA synthetase [EC:6.1.1.10]	6412	6714	6299	6516	7488	7743	7705	7311	7785	7475	7715	7530
K07309 Tat-targeted selenate reductase subunit YnfE [EC:1.97.1.9]	1255	1258	1157	1358	893	967	820	875	1950	1859	1894	1844
K07310 Tat-targeted selenate reductase subunit YnfF [EC:1.97.1.9]	5	11	11	8	34	39	29	33	114	144	137	114
K11717 cysteine desulfurase / selenocysteine lyase [EC:2.8.1.7 4.4.1.16]	1734	1738	1834	1809	3381	3485	3574	3359	3340	3274	3425	3343
K12527 putative selenate reductase [EC:1.97.1.9]	1310	1273	1237	1279	392	470	448	416	1301	1482	1357	1361
K12528 putative selenate reductase molybdopterin-binding subunit	16	10	17	6	38	34	44	33	173	170	187	189
K12529 putative selenate reductase FAD-binding subunit	0	1	0	1	9	6	11	10	43	41	37	58
ko00460 Cyanoamino acid metabolism	54789	55753	56554	55832	93081	91752	94310	92020	83690	82659	84193	81963
K10814 hydrogen cyanide synthase HcnA [EC:1.4.99.5]	397	408	399	379	236	188	190	240	150	137	112	102
K10815 hydrogen cyanide synthase HcnB [EC:1.4.99.5]	652	653	577	703	333	293	316	354	190	155	193	197
K10816 hydrogen cyanide synthase HcnC [EC:1.4.99.5]	1731	1712	1501	1742	869	850	793	913	489	493	486	471
K18282 cyanide dihydratase [EC:3.5.5.-]	0	0	0	0	0	0	0	1	0	0	0	0
ko00471 D-Glutamine and D-glutamate metabolism	15224	15081	14944	15213	16826	17335	17417	17037	17085	16882	17014	17054
K01776 glutamate racemase [EC:5.1.1.3]	2921	2983	2975	3007	3151	3357	3239	3144	3002	3005	3000	2980
K01924 UDP-N-acetylmuramate--alanine ligase [EC:6.3.2.8]	5304	5105	5096	5223	5477	5627	5569	5659	5370	5179	5158	5376
K01925 UDP-N-acetylmuramoylalanine--D-glutamate ligase [EC:6.3.2.9]	4613	4697	4526	4574	5196	5177	5211	5184	4866	4958	4854	4875
K22210 D-glutamate cyclase [EC:4.2.1.48]	1	0	0	1	0	0	0	0	0	0	0	1
K23169 UDP-N-acetylmuramoyl-L-alanine---L-glutamate ligase [EC:6.3.2.53]	75	73	68	64	8	19	15	23	37	52	40	44
K23170 UDP-N-acetyl-alpha-D-muramoyl-L-alanyl-L-glutamate epimerase [EC:5.1.1.23]	58	80	66	42	18	29	13	14	48	38	34	35
ko00472 D-Arginine and D-ornithine metabolism	4026	4096	3930	3926	2283	2259	2158	2272	1573	1428	1390	1514
K17898 D-ornithine 4,5-aminomutase subunit beta [EC:5.4.3.5]	1	0	0	0	0	0	2	3	1	4	5	2
K17899 D-ornithine 4,5-aminomutase subunit alpha [EC:5.4.3.5]	0	3	0	0	0	0	2	0	0	0	0	3
K19744 L-arginine dehydrogenase [EC:1.4.1.25]	1557	1535	1412	1442	918	860	777	839	478	449	421	452
K19746 D-arginine dehydrogenase [EC:1.4.99.6]	1785	1847	1822	1796	996	968	983	1063	689	529	547	592
K21898 ornithine racemase [EC:5.1.1.12]	19	45	41	31	40	57	29	37	27	25	44	52
ko00473 D-Alanine metabolism	11584	11376	11284	11249	12835	12757	12829	12622	12103	12115	11999	11890
K01775 alanine racemase [EC:5.1.1.1]	5618	5449	5415	5381	7500	7409	7367	7298	6646	6678	6636	6487
K01921 D-alanine-D-alanine ligase [EC:6.3.2.4]	5117	5141	5072	5038	4567	4518	4615	4569	5003	5021	4923	4991
K03367 D-alanine--poly(phosphoribitol) ligase subunit 1 [EC:6.1.1.13]	469	453	456	482	466	463	498	443	268	260	267	241
K14188 D-alanine--poly(phosphoribitol) ligase subunit 2 [EC:6.1.1.13]	77	70	70	82	59	81	82	79	53	33	57	44
ko00480 Glutathione metabolism	72117	71420	70079	71801	54876	55182	53923	55239	55062	55030	54029	55526
K00383 glutathione reductase (NADPH) [EC:1.8.1.7]	2927	2810	2641	2678	1547	1643	1704	1649	1776	1850	1640	1831
K00799 glutathione S-transferase [EC:2.5.1.18]	8066	7801	7767	8129	4589	4538	4351	4600	5020	4797	4675	4936
K01255 leucyl aminopeptidase [EC:3.4.11.1]	3148	3053	2887	3141	1693	1832	1609	1785	2297	2556	2342	2495
K01256 aminopeptidase N [EC:3.4.11.2]	5582	5558	5449	5600	3753	3630	3708	3565	3451	3498	3494	3525
K01270 dipeptidase D [EC:3.4.13.-]	3016	3181	3164	3080	6994	7072	7013	6803	6070	6048	5922	6206
K01460 glutathionylspermidine amidase/synthetase [EC:3.5.1.78 6.3.1.8]	664	693	768	780	250	183	231	200	1538	1638	1592	1483
K01469 5-oxoprolinase (ATP-hydrolysing) [EC:3.5.2.9]	2	2	2	3	0	5	3	0	4	10	4	8
K06048 glutamate---cysteine ligase / carboxylate-amine ligase [EC:6.3.2.2 6.3.-.-]	2188	2244	2138	2295	1250	1269	1182	1253	1345	1489	1400	1502
K07160 5-oxoprolinase (ATP-hydrolysing) subunit A [EC:3.5.2.9]	3965	4053	3697	3950	2222	2221	2098	2406	1956	1786	1667	1790
K07232 glutathione-specific gamma-glutamylcyclotransferase [EC:4.3.2.7]	366	390	365	427	289	320	260	252	503	529	532	539
K07751 PepB aminopeptidase [EC:3.4.11.23]	867	819	775	760	482	577	542	548	1031	1114	1060	1178
K15428 Cys-Gly metallodipeptidase DUG1 [EC:3.4.13.-]	2	2	2	0	0	0	0	0	0	0	0	0
K23123 5-oxoprolinase (ATP-hydrolysing) subunit B [EC:3.5.2.9]	355	358	351	363	278	288	294	269	408	444	461	485
K23124 5-oxoprolinase (ATP-hydrolysing) subunit C [EC:3.5.2.9]	489	562	520	477	385	405	391	362	702	711	731	670
Glycan biosynthesis and metabolism	241570	246088	250980	246569	364846	367201	367403	360665	333664	329566	333903	329667
ko00510 N-Glycan biosynthesis	791	837	897	859	1733	1750	1884	1744	1186	1137	1310	1226
K00721 dolichol-phosphate mannosyltransferase [EC:2.4.1.83]	763	807	855	829	1728	1742	1879	1741	1150	1063	1255	1170
K00737 beta-1,4-mannosyl-glycoprotein beta-1,4-N-acetylglucosaminyltransferase [EC:2.4.1.144]	8	9	13	9	0	4	1	1	13	15	10	12
K01001 UDP-N-acetylglucosamine--dolichyl-phosphate N-acetylglucosaminephosphotransferase [EC:2.7.8.15]	0	0	2	0	2	0	0	1	4	11	5	7
K07151 dolichyl-diphosphooligosaccharide---protein glycosyltransferase [EC:2.4.99.18]	20	21	27	21	3	4	4	1	19	48	40	37
ko00513 Various types of N-glycan biosynthesis	12253	13647	14863	13737	42752	42817	43878	42788	31402	30085	31721	30060
ko00515 Mannose type O-glycan biosynthesis	53	56	40	57	46	51	59	46	76	58	79	113
K00728 dolichyl-phosphate-mannose-protein mannosyltransferase [EC:2.4.1.109]	53	56	40	57	46	51	59	46	76	58	79	112
ko00514 Other types of O-glycan biosynthesis	53	56	40	57	46	51	59	46	76	58	79	112
ko00531 Glycosaminoglycan degradation	21940	23170	25148	23636	62104	61784	63099	61476	47710	45944	48090	46046
K01135 arylsulfatase B [EC:3.1.6.12]	0	0	0	1	0	0	0	0	2	0	1	1
K01137 N-acetylglucosamine-6-sulfatase [EC:3.1.6.14]	2	4	2	0	2	1	0	0	0	0	0	0
K01197 hyaluronoglucosaminidase [EC:3.2.1.35]	255	265	275	308	396	310	390	345	22	50	29	63
K01205 alpha-N-acetylglucosaminidase [EC:3.2.1.50]	2600	2712	3198	2864	9707	9394	9500	9246	6704	6511	6790	6454
K01217 L-iduronidase [EC:3.2.1.76]	0	1	5	1	0	0	0	0	1	0	0	1
K01565 N-sulfoglucosamine sulfohydrolase [EC:3.10.1.1]	8	4	6	4	7	5	4	2	2	4	3	1
ko00563 Glycosylphosphatidylinositol (GPI)-anchor biosynthesis	0	1	0	0	0	0	0	0	0	0	0	0
K03434 N-acetylglucosaminylphosphatidylinositol deacetylase [EC:3.5.1.89]	0	1	0	0	0	0	0	0	0	0	0	0
ko00601 Glycosphingolipid biosynthesis - lacto and neolacto series	3	1	0	1	0	0	0	0	0	0	1	0
K00709 histo-blood group ABO system transferase [EC:2.4.1.40 2.4.1.37]	3	1	0	1	0	0	0	0	0	0	1	0
ko00603 Glycosphingolipid biosynthesis - globo and isoglobo series	22516	24234	25747	24358	57871	58084	59275	57657	50553	49484	50719	49190
K00722 globoside alpha-N-acetylgalactosaminyltransferase [EC:2.4.1.88]	1	0	0	0	0	0	0	0	0	0	0	0
K01204 alpha-N-acetylgalactosaminidase [EC:3.2.1.49]	1	0	0	0	0	0	0	0	0	0	0	0
ko00604 Glycosphingolipid biosynthesis - ganglio series	12237	13631	14837	13720	42758	42818	43881	42796	31387	30047	31685	30028
ko00540 Lipopolysaccharide biosynthesis	66881	66967	66581	67463	71571	71940	68813	69446	65795	65943	65880	66690
K00677 UDP-N-acetylglucosamine acyltransferase [EC:2.3.1.129]	2770	2740	2830	2703	4653	4531	3883	3869	3556	3626	3787	3728
K00713 UDP-glucose:(glucosyl)LPS alpha-1,2-glucosyltransferase [EC:2.4.1.-]	0	2	0	0	0	0	0	0	3	1	0	2
K00748 lipid-A-disaccharide synthase [EC:2.4.1.182]	3125	3234	3284	3270	4118	4108	4159	4172	3297	3140	3304	3184
K00912 tetraacyldisaccharide 4'-kinase [EC:2.7.1.130]	2414	2506	2585	2674	3674	3722	3750	3709	3173	3032	3022	3115
K00979 3-deoxy-manno-octulosonate cytidylyltransferase (CMP-KDO synthetase) [EC:2.7.7.38]	1974	1930	1876	2088	2453	2364	2353	2372	2043	2041	2070	2009
K01627 2-dehydro-3-deoxyphosphooctonate aldolase (KDO 8-P synthase) [EC:2.5.1.55]	2071	2202	2086	2038	2779	2741	2641	2791	2147	2132	2182	2170
K02517 Kdo2-lipid IVA lauroyltransferase/acyltransferase [EC:2.3.1.241 2.3.1.-]	3808	3961	4091	4021	3871	4034	3783	3886	3632	3574	3706	3707
K02527 3-deoxy-D-manno-octulosonic-acid transferase [EC:2.4.99.12 2.4.99.13 2.4.99.14 2.4.99.15]	3225	3236	3198	3155	4292	4216	4254	4338	3576	3638	3666	3697
K02535 UDP-3-O-[3-hydroxymyristoyl] N-acetylglucosamine deacetylase [EC:3.5.1.108]	1773	1928	1896	1957	1209	1309	1214	1203	1270	1455	1291	1318
K02536 UDP-3-O-[3-hydroxymyristoyl] glucosamine N-acyltransferase [EC:2.3.1.191]	3561	3477	3705	3580	5749	5688	4056	3973	4542	4399	4505	4457
K02560 lauroyl-Kdo2-lipid IVA myristoyltransferase [EC:2.3.1.243]	556	540	481	527	416	329	421	427	728	750	646	770

K02840 UDP-D-galactose:(glucosyl)LPS alpha-1,6-D-galactosyltransferase [EC:2.4.1.-]	0	1	2	0	2	1	3	5	1	2	8	2
K02841 heptosyltransferase I [EC:2.4.-.-]	1136	1156	1031	1117	787	813	819	709	1056	1108	1020	1084
K02843 heptosyltransferase II [EC:2.4.-.-]	1417	1459	1494	1453	2864	2763	2854	2843	2798	2936	3055	2937
K02844 UDP-glucose:(heptosyl)LPS alpha-1,3-glucosyltransferase [EC:2.4.1.-]	2594	2631	2620	2715	1550	1600	1572	1548	1569	1694	1541	1768
K02847 O-antigen ligase [EC:2.4.1.-]	3	3	6	3	11	20	22	13	93	79	95	76
K02848 heptose I phosphotransferase [EC:2.7.1.-]	1272	1369	1271	1324	796	712	722	712	483	457	433	465
K02849 heptosyltransferase III [EC:2.4.-.-]	670	660	670	723	514	528	483	512	935	995	911	977
K02850 heptose II phosphotransferase [EC:2.7.1.-]	3	5	2	0	9	8	15	7	30	30	29	46
K03269 UDP-2,3-diacylglucosamine hydrolase [EC:3.6.1.54]	1327	1451	1477	1400	2227	2327	2303	2302	2065	1922	2066	1982
K03270 3-deoxy-D-manno-octulosonate 8-phosphate phosphatase (KDO 8-P phosphatase) [EC:3.1.3.45]	1622	1568	1564	1644	1846	2004	1798	1865	1543	1647	1530	1508
K03271 D-sedoheptulose 7-phosphate isomerase [EC:5.3.1.28]	1990	2035	2006	2133	1773	1831	1810	1818	1959	1942	1886	1913
K03272 D-beta-D-heptose 7-phosphate kinase / D-beta-D-heptose 1-phosphate adenosyltransferase [EC:2.7.1.167 2.7.7.70]	3566	3596	3293	3507	2168	2069	1971	2076	2075	2174	2221	2334
K03273 D-glycero-D-manno-heptose 1,7-bisphosphate phosphatase [EC:3.1.3.82 3.1.3.83]	1994	1962	2018	1991	2343	2506	2489	2469	2153	2214	2190	2103
K03274 ADP-L-glycero-D-manno-heptose 6-epimerase [EC:5.1.3.20]	2039	1895	1910	1962	1281	1294	1168	1222	1447	1423	1403	1386
K03275 UDP-glucose:(glucosyl)LPS alpha-1,3-glucosyltransferase [EC:2.4.1.-]	4	4	5	9	16	9	18	8	51	38	56	51
K03276 UDP-glucose/galactose:(glucosyl)LPS alpha-1,2-glucosyl/galactosyltransferase [EC:2.4.1.-]	10	9	13	14	50	38	36	23	98	82	59	71
K03277 heptosyltransferase IV [EC:2.4.-.-]	0	1	0	0	0	2	0	0	0	0	0	0
K03760 lipid A ethanolaminephosphotransferase [EC:2.7.8.43]	3898	3769	3561	3734	2223	2275	2116	2246	2325	2238	2254	2280
K06041 arabinose-5-phosphate isomerase [EC:5.3.1.13]	1936	1913	1875	1927	2737	2829	2835	2656	2108	2325	2257	2307
K07031 D-glycero-alpha-D-manno-heptose-7-phosphate kinase [EC:2.7.1.168]	2056	2087	2180	2135	2988	2871	3189	3087	3007	3028	3033	3081
K07264 4-amino-4-deoxy-L-arabinose transferase [EC:2.4.2.43]	2759	2548	2371	2608	1493	1523	1410	1659	1853	1839	1702	1928
K09778 Kdo2-lipid IVA 3' secondary acyltransferase [EC:2.3.1.-]	3	3	5	7	0	0	3	2	1	0	2	1
K12973 lipid IVA palmitoyltransferase [EC:2.3.1.251]	327	330	358	339	287	318	314	313	478	599	525	532
K12974 KDO2-lipid IV(A) palmitoleoyltransferase [EC:2.3.1.242]	500	504	485	554	334	414	367	350	707	701	678	821
K12975 KDO II ethanolaminephosphotransferase [EC:2.7.8.42]	1181	1121	1104	1121	806	806	771	791	1578	1572	1557	1592
K12976 lipid A 3-O-deacylase [EC:3.1.1.-]	426	467	442	431	257	274	315	373	138	136	141	142
K12979 beta-hydroxylase [EC:1.14.11.-]	3162	3088	3045	3050	1659	1635	1596	1728	1543	1377	1480	1554
K12981 KDO transferase III [EC:2.4.99.-]	86	88	82	82	123	114	172	128	0	1	0	0
K12983 UDP-glucose:(glucosyl)LPS beta-1,3-glucosyltransferase [EC:2.4.1.-]	2	0	0	2	15	14	11	13	43	44	49	44
K12985 (galactosyl)LPS 1,2-glucosyltransferase [EC:2.4.1.-]	1	3	1	6	8	15	5	4	35	49	55	50
K12986 1,5-rhamnosyltransferase [EC:2.4.1.-]	0	0	0	0	3	0	2	0	0	0	0	1
K15669 D-glycero-alpha-D-manno-heptose 1-phosphate guanylyltransferase [EC:2.7.7.71]	672	663	749	639	1895	1754	1964	1889	1498	1401	1434	1430
K19353 heptose-I-phosphate ethanolaminephosphotransferase [EC:2.7.8.-]	3056	2983	3105	2989	1698	1800	1585	1716	1257	1230	1256	1260
K19803 Kdo2-lipid A phosphotransferase [EC:2.7.4.29]	829	826	764	838	499	599	443	486	723	765	630	745
K21344 D-glycero-beta-D-manno-heptose-7-phosphate kinase [EC:2.7.1.167]	56	63	62	47	88	84	86	93	78	87	77	81
K21345 D-glycero-beta-D-manno-heptose 1-phosphate adenylyltransferase [EC:2.7.7.70]	33	23	40	27	39	37	43	54	47	29	32	41
K23067 beta-1,4-galactosyltransferase	0	0	0	0	0	0	0	0	0	0	0	1
ko00550 Peptidoglycan biosynthesis	86848	87008	87124	86745	88584	90742	90751	89004	93249	94341	93030	93778
K00687 penicillin-binding protein 2B	110	135	106	126	39	39	32	21	116	185	110	144
K00887 undecaprenol kinase [EC:2.7.1.66]	25	23	29	15	3	5	8	8	31	35	39	29
K01000 phospho-N-acetylmuramoyl-pentapeptide-transferase [EC:2.7.8.13]	2617	2792	2880	2888	3912	4145	4297	3919	4056	4316	4115	3923
K02563 UDP-N-acetylglucosamine--N-acetylmuramyl-(pentapeptide) pyrophosphoryl-undecaprenol N-acetylglucosamine transferase [EC:2.4.1.227]	3791	3850	3717	3791	4322	4365	4445	4345	4045	3783	3953	3955
K03587 cell division protein FtsI (penicillin-binding protein 3) [EC:3.4.16.4]	7696	7695	7686	7682	8334	8378	8283	8272	8046	7716	7911	7807
K03693 penicillin-binding protein 1B	125	118	93	124	17	45	26	40	115	158	117	118
K03814 monofunctional glycosyltransferase [EC:2.4.1.129]	2004	2089	2011	2010	2209	2110	2163	2224	1960	1845	1838	1928
K05362 UDP-N-acetylmuramoyl-L-alanyl-D-glutamate-L-lysine ligase [EC:6.3.2.7]	80	98	104	99	19	17	23	22	102	136	82	94
K05363 serine/alanine adding enzyme [EC:2.3.2.10]	48	65	51	72	4	7	7	9	76	72	50	60
K05364 penicillin-binding protein A	396	381	382	369	78	77	117	86	310	408	358	395
K05365 penicillin-binding protein 1B [EC:2.4.1.129 3.4.16.4]	5137	5053	5024	5118	3198	3236	2919	3065	3079	3245	3213	3295
K05366 penicillin-binding protein 1A [EC:2.4.1.129 3.4.16.4]	8090	8285	8511	8221	8144	8350	8238	8210	8035	8209	8080	8270
K05367 penicillin-binding protein 1C [EC:2.4.1.129]	2517	2542	2480	2586	4923	5069	5341	4910	4851	4925	4855	4779
K05515 penicillin-binding protein 2 [EC:3.4.16.4]	6289	6351	6261	6331	6424	6962	6650	6492	7691	7628	7682	7322
K06153 undecaprenyl-diphosphatase [EC:3.6.1.27]	2935	3057	3042	2946	3187	3061	3096	3008	3367	3219	3317	3238
K07009 lipid II isoglutaminyl synthase (glutamine-hydrolyzing) [EC:6.3.5.13]	92	118	91	92	87	76	73	90	145	161	170	154
K07258 serine-type D-Ala-D-Ala carboxypeptidase (penicillin-binding protein 5/6) [EC:3.4.16.4]	6654	6142	6430	6460	2947	3104	2973	2969	5581	5957	5625	5964
K07259 serine-type D-Ala-D-Ala carboxypeptidase/endopeptidase (penicillin-binding protein 4) [EC:3.4.16.4 3.4.21.-]	3295	3261	3325	3077	4556	4888	5044	4764	4140	3979	4147	4049
K07260 zinc D-Ala-D-Ala carboxypeptidase [EC:3.4.17.14]	295	265	307	294	80	88	86	85	312	364	325	337
K08384 stage V sporulation protein D (sporulation-specific penicillin-binding protein)	1553	1440	1465	1499	413	438	483	406	1472	1676	1562	1640
K08724 penicillin-binding protein 2B	3	3	3	4	7	15	19	10	9	5	5	11
K11693 peptidoglycan pentaglycine glycine transferase (the first glycine) [EC:2.3.2.16]	0	2	0	0	4	0	0	0	0	0	2	0
K11694 peptidoglycan pentaglycine glycine transferase (the second and third glycine) [EC:2.3.2.17]	1	0	0	0	0	2	0	3	1	2	0	3
K12554 alanine adding enzyme [EC:2.3.2.-]	76	102	90	50	16	15	18	11	81	87	87	99
K12555 penicillin-binding protein 2A [EC:2.4.1.129 3.4.16.4]	263	337	304	311	64	60	72	60	250	299	216	232
K12556 penicillin-binding protein 2X	107	115	107	117	36	39	35	22	131	140	137	128
K18149 penicillin-binding protein	106	97	88	98	15	8	17	11	85	94	79	119
K18770 penicillin-binding protein 4 [EC:2.4.1.129 3.4.16.4]	2	0	0	0	0	0	0	5	0	0	0	0
K18866 zinc D-Ala-D-Ala dipeptidase/carboxypeptidase [EC:3.4.13.22 3.4.17.14]	7	5	12	5	8	0	4	4	4	8	9	5
K19302 undecaprenyl-diphosphatase [EC:3.6.1.27]	3331	3310	3180	3247	2750	2751	2951	3076	2635	2592	2702	2670
K21464 penicillin-binding protein 2D [EC:2.4.1.129 3.4.16.4]	6	5	5	4	5	7	4	3	14	9	8	11
K21465 penicillin-binding protein A	0	0	2	0	3	4	3	2	3	7	0	0
K21469 serine-type D-Ala-D-Ala carboxypeptidase [EC:3.4.16.4]	3	0	0	0	13	10	10	9	10	9	7	15
K23393 lipid II isoglutaminyl synthase (glutamine-hydrolyzing) [EC:6.3.5.13]	209	204	209	223	156	153	109	107	288	307	278	331
ko00511 Other glycan degradation	64353	68557	72676	68431	166618	166689	169506	165064	136142	130969	136413	130815
K01191 alpha-mannosidase [EC:3.2.1.24]	4577	4779	4906	4864	5245	5153	5480	5166	8407	8418	8678	8612
K01192 beta-mannosidase [EC:3.2.1.25]	5160	5322	5520	5290	9105	8987	9196	8860	11382	11333	11323	11053
K01206 alpha-L-fucosidase [EC:3.2.1.51]	5778	5816	6254	5966	12704	12642	12804	12621	11046	10391	10911	10345
K01227 mannosyl-glycoprotein endo-beta-N-acetylglucosaminidase [EC:3.2.1.96]	868	851	866	879	159	209	140	124	469	569	542	553
K01444 N4-(beta-N-acetylglucosaminyl)-L-asparaginase [EC:3.5.1.26]	2	0	0	0	0	0	0	0	0	0	2	1
K15923 alpha-L-fucosidase 2 [EC:3.2.1.51]	9302	10187	10877	10000	31704	31856	32206	31458	23944	22760	24037	22497
ko00571 Lipoarabinomannan (LAM) biosynthesis	1751	1744	1665	1680	1219	1166	1119	1207	693	621	662	751
K08256 phosphatidyl-myo-inositol alpha-mannosyltransferase [EC:2.4.1.345]	60	79	76	85	270	285	292	268	186	175	178	183
K14335 alpha-1,6-mannosyltransferase [EC:2.4.1.-]	1691	1663	1589	1595	949	881	827	939	507	446	484	568
K22311 phosphatidylinositol dimannoside acyltransferase [EC:2.3.1.265]	0	2	0	0	0	0	0	0	0	0	0	0
ko00572 Arabinogalactan biosynthesis - Mycobacterium	904	770	775	775	607	626	652	604	1030	1153	1085	1109
K02851 UDP-GlcNAc:undecaprenyl-phosphate/decaprenyl-phosphate GlcNAc-1-phosphate transferase [EC:2.7.8.33 2.7.8.35]	899	768	770	768	601	622	649	597	1015	1125	1085	1095
K16650 galactofuranosylgalactofuranosylrhamnosyl-N-acetylglucosaminyl-diphospho-decaprenol beta-1,5/1,6-galactofuranosyltransferase [EC:2.4.1.288]	0	0	0	0	0	1	3	0	6	17	0	10
K22902 D-galactosaminyltransferase	5	2	5	7	6	3	0	7	9	11	0	4
Metabolism of cofactors and vitamins	535572	534387	523670	530729	500589	500853	497817	500763	493153	490778	487504	492888
ko00730 Thiamine metabolism	50870	51286	51216	51131	57812	58113	58381	57578	55567	56271	55802	55628
K00788 thiamine-phosphate pyrophosphorylase [EC:2.5.1.3]	2332	2451	2410	2347	3140	3356	3377	3192	3054	3112	3124	3008
K00877 hydroxymethylpyrimidine/phosphomethylpyrimidine kinase / thiaminase [EC:2.7.1.49 2.7.4.7 3.5.99.2]	1	0	0	0	1	0	0	0	0	0	1	1
K00878 hydroxyethylthiazole kinase [EC:2.7.1.50]	1075	1055	979	1087	494	461	464	500	1125	1242	1289	1205
K00941 hydroxymethylpyrimidine/phosphomethylpyrimidine kinase [EC:2.7.1.49 2.7.4.7]	2533	2627	2533	2549	2677	2559	2537	2576	2655	2768	2610	2679
K00946 thiamine-monophosphate kinase [EC:2.7.4.16]	2743	2729	2744	2634	3546	3540	3659	3592	3121	3005	2964	3160
K00949 thiamine pyrophosphokinase [EC:2.7.6.2]	689	780	810	751	1511	1565	1519	1410	1380	1386	1431	1414
K01077 alkaline phosphatase [EC:3.1.3.1]	4527	4672	4930	4814	7494	7562	7672	7634	6192	6091	6417	6081
K01662 1-deoxy-D-xylulose-5-phosphate synthase [EC:2.2.1.7]	7630	7602	7659	7587	10318	10121	10353	10111	8777	8802	8735	8488
K03147 phosphomethylpyrimidine synthase [EC:4.1.99.17]	5511	5615	5403	5549	5776	6134	5935	5893	5663	5637	5592	5612
K03148 sulfur carrier protein ThiS adenylyltransferase [EC:2.7.7.73]	463	506	491	504	338	399	391	360	563	684	685	716
K03149 thiazole synthase [EC:2.8.1.10]	2400	2321	2316	2286	2682	2684	2706	2685	2312	2394	2266	2344
K03150 2-iminoacetate synthase [EC:4.1.99.19]	2820	2952	3123	2994	5164	5307	5410	5255	4832	5121	5029	4892
K03151 tRNA uracil 4-sulfurtransferase [EC:2.8.1.4]	3924	3873	3721	3831	2120	2105	2009	2050	2627	2657	2514	2650
K03153 glycine oxidase [EC:1.4.3.19]	1655	1667	1702	1643	995	933	915	883	548	490	545	558
K03707 thiaminase (transcriptional activator TenA) [EC:3.5.99.2]	642	662	727	669	163	142	163	147	1120	1119	1144	1151
K04487 cysteine desulfurase [EC:2.8.1.7]	4959	4725	4754	4819	2670	2858	2652	2803	3115	3420	3078	3237
K06949 ribosome biogenesis GTPase / thiamine phosphate phosphatase [EC:3.6.1.- 3.1.3.100]	4179	4204	4153	4278	6058	5742	5991	5834	5565	5440	5456	5486
K07251 thiamine kinase [EC:2.7.1.89]	502	449	466	472	328	307	296	323	615	599	669	689
K10810 thiazole tautomerase (transcriptional regulator TenI) [EC:5.3.99.10]	0	0	0	0	0	0	0	1	0	0	0	0
K14153 hydroxymethylpyrimidine kinase / phosphomethylpyrimidine kinase / thiamine-phosphate diphosphorylase [EC:2.7.1.49 2.7.4.7 2.5.1.3]	2	1	3	3	8	5	7	1	3	2	7	4
K14154 thiamine-phosphate diphosphorylase / hydroxyethylthiazole kinase [EC:2.5.1.3 2.7.1.50]	1	0	0	0	0	0	0	0	0	0	2	0
K14394 low molecular weight phosphotyrosine protein phosphatase [EC:3.1.3.2 3.1.3.48]	0	1	0	0	0	1	0	1	0	0	0	1
K18278 pyrimidine precursor biosynthesis enzyme	0	0	0	0	0	0	0	0	2	0	0	0
K21219 hydroxymethylpyrimidine kinase / phosphomethylpyrimidine kinase / thiamine-phosphate diphosphorylase [EC:2.7.1.49 2.7.4.7 2.5.1.3]	0	0	0	0	0	0	0	0	0	0	0	1
K22699 sulfide-dependent adenosine diphosphate thiazole synthase [EC:2.4.2.59]	3	2	5	5	0	0	1	0	0	0	0	0
ko00740 Riboflavin metabolism	21818	22168	20993	21472	21783	21538	21803	21674	20935	21071	21151	21578
K00793 riboflavin synthase [EC:2.5.1.9]	1809	1813	1763	1828	2157	2248	2266	2293	1585	1633	1582	1642
K00794 6,7-dimethyl-8-ribityllumazine synthase [EC:2.5.1.78]	1539	1505	1390	1321	1856	1851	1881	1822	1584	1441	1616	1517
K01497 GTP cyclohydrolase II [EC:3.5.4.25]	1432	1332	1287	1330	840	759	719	786	777	833	810	741
K02858 3,4-dihydroxy 2-butanone 4-phosphate synthase [EC:4.1.99.12]	429	403	392	446	288	326	331	302	592	610	543	659
K03788 acid phosphatase (class B) [EC:3.1.3.2]	467	433	415	481	331	300	294	288	574	538	611	627
K04719 5,6-dimethylbenzimidazole synthase [EC:1.13.11.79]	718	703	572	618	321	292	291	378	202	206	155	213

K05368 aquacobalamin reductase / NAD(P)H-flavin reductase [EC:1.16.1.3 1.5.1.41]	494	491	431	458	324	363	321	358	616	705	678	647
K07732 riboflavin kinase, archaea type [EC:2.7.1.161]	0	0	1	0	0	0	0	0	0	0	0	4
K08096 GTP cyclohydrolase IIa [EC:3.5.4.29]	0	0	0	0	0	0	0	0	2	1	0	9
K09474 acid phosphatase (class A) [EC:3.1.3.2]	324	343	281	298	221	196	215	167	500	502	523	577
K11752 diaminohydroxyphosphoribosylaminopyrimidine deaminase / 5-amino-6-(5-phosphoribosylamino)uracil reductase [EC:3.5.4.26 1.1.1.193]	3137	3256	3132	3150	3768	3781	3814	3841	3253	3172	3285	3229
K11753 riboflavin kinase / FMN adenylyltransferase [EC:2.7.1.26 2.7.7.2]	3398	3497	3462	3572	3861	3778	3835	3685	3328	3489	3526	3620
K14379 tartrate-resistant acid phosphatase type 5 [EC:3.1.3.2]	0	0	2	0	0	0	0	0	0	1	0	0
K14652 3,4-dihydroxy 2-butanone 4-phosphate synthase / GTP cyclohydrolase II [EC:4.1.99.12 3.5.4.25]	2644	2748	2624	2532	4167	3995	4181	4032	2922	2656	2914	2777
K14653 2-amino-5-formylamino-6-ribosylaminopyrimidin-4(3H)-one 5'-monophosphate deformylase [EC:3.5.1.102]	0	1	0	0	3	0	2	0	2	7	0	2
K14654 2,5-diamino-6-(ribosylamino)-4(3H)-pyrimidinone 5'-phosphate reductase [EC:1.1.1.302]	98	102	120	106	19	23	9	12	56	68	76	77
K14655 tRNA pseudouridine32 synthase / 2,5-diamino-6-(5-phospho-D-ribitylamino)-pyrimidin-4(3H)-one deaminase [EC:5.4.99.28]	0	1	0	0	0	0	0	0	0	0	0	0
K14656 FAD synthetase [EC:2.7.7.2]	0	2	0	1	0	0	0	2	3	5	2	3
K19285 FMN reductase (NADPH) [EC:1.5.1.38]	284	258	313	245	57	61	59	33	152	233	198	174
K19286 FMN reductase [NAD(P)H] [EC:1.5.1.39]	0	2	9	4	0	4	3	1	4	4	5	5
K20861 FMN hydrolase / 5-amino-6-(5-phospho-D-ribitylamino)uracil phosphatase [EC:3.1.3.102 3.1.3.104]	253	283	235	273	74	89	90	74	574	606	520	603
K20862 FMN hydrolase / 5-amino-6-(5-phospho-D-ribitylamino)uracil phosphatase [EC:3.1.3.102 3.1.3.104]	1697	1768	1620	1671	963	1019	936	1056	994	1014	957	1087
K20884 riboflavin kinase / FMN hydrolase [EC:2.7.1.26 3.1.3.102]	0	1	0	1	2	1	1	2	3	3	0	3
K21063 5-amino-6-(5-phospho-D-ribitylamino)uracil phosphatase [EC:3.1.3.104]	388	408	421	395	1222	1162	1305	1153	830	797	854	840
K21064 5-amino-6-(5-phospho-D-ribitylamino)uracil phosphatase [EC:3.1.3.104]	46	48	58	35	20	6	19	14	49	71	64	42
K22393 flavin reductase (NADH) subunit 1 [EC:1.5.1.36]	0	2	2	1	2	0	0	2	2	0	2	0
K22394 flavin reductase (NADH) subunit 2 [EC:1.5.1.36]	0	0	0	0	2	2	0	0	2	0	0	1
ko00750 Vitamin B6 metabolism	23086	22667	23367	23030	24096	24317	24101	24190	23554	23284	23850	23711
K00097 4-hydroxythreonine-4-phosphate dehydrogenase [EC:1.1.1.262]	2960	2953	3132	2991	3372	3586	3568	3516	2813	2853	3013	2932
K00275 pyridoxamine 5'-phosphate oxidase [EC:1.4.3.5]	1726	1683	1734	1679	2065	2058	2103	2149	1777	1748	1757	1745
K00868 pyridoxine kinase [EC:2.7.1.35]	3578	3318	3436	3500	3294	3252	3229	3131	3631	3703	3660	3803
K03472 D-erythrose 4-phosphate dehydrogenase [EC:1.2.1.72]	2305	2249	2257	2171	1443	1364	1336	1300	1414	1362	1385	1490
K03473 erythronate-4-phosphate dehydrogenase [EC:1.1.1.290]	2646	2529	2568	2668	2464	2501	2379	2635	2134	2163	2225	2285
K03474 pyridoxine 5-phosphate synthase [EC:2.6.99.2]	1775	1969	1952	1812	2458	2493	2542	2416	2119	2156	2131	1970
K05275 pyridoxine 4-dehydrogenase [EC:1.1.1.65]	465	476	494	556	375	374	337	353	780	749	789	740
K06215 pyridoxal 5'-phosphate synthase pdxS subunit [EC:4.3.3.6]	220	174	229	199	57	62	88	55	377	384	351	362
K08681 5'-phosphate synthase pdxT subunit [EC:4.3.3.6]	93	95	88	108	26	27	33	30	208	222	190	212
ko00760 Nicotinate and nicotinamide metabolism	59558	60554	59167	60289	56365	56642	55916	56048	57758	57918	56796	57969
K00322 NAD(P) transhydrogenase [EC:1.6.1.1]	2781	2779	2718	2741	1775	1707	1608	1634	1688	2005	1783	1817
K00324 H+-translocating NAD(P) transhydrogenase subunit alpha [EC:1.6.1.2 7.1.1.1]	2888	2849	2754	2906	1638	1695	1518	1726	1814	2016	1922	1929
K00325 H+-translocating NAD(P) transhydrogenase subunit beta [EC:1.6.1.2 7.1.1.1]	2693	2723	2530	2680	1608	1625	1494	1679	1727	1767	1546	1640
K00763 nicotinate phosphoribosyltransferase [EC:6.3.4.21]	5586	5535	5523	5912	5298	5116	4970	5139	4930	4817	4804	4864
K00767 nicotinate-nucleotide pyrophosphorylase (carboxylating) [EC:2.4.2.19]	2535	2631	2535	2675	2884	3032	2916	2886	2527	2422	2480	2451
K00858 NAD+ kinase [EC:2.7.1.23]	2402	2491	2459	2402	2983	2853	2957	2879	2913	2792	2797	3023
K00952 nicotinamide-nucleotide adenylyltransferase [EC:2.7.7.1]	0	0	2	1	0	0	0	0	2	2	4	4
K00969 nicotinate-nucleotide adenylyltransferase [EC:2.7.7.18]	1937	1985	1914	1872	2119	2178	2093	2230	2280	1964	1991	2106
K01440 nicotinamidase [EC:3.5.1.19]	1	3	2	2	1	0	4	2	4	2	2	4
K01916 NAD+ synthase [EC:6.3.1.5]	1964	2007	1923	1863	1167	1176	1132	1231	1231	1249	1141	1332
K01950 NAD+ synthase (glutamine-hydrolysing) [EC:6.3.5.1]	1641	1831	1760	1693	2907	2996	3072	2914	2775	2845	2856	2671
K03426 NAD+ diphosphatase [EC:3.6.1.22]	2450	2564	2504	2504	2696	2686	2733	2785	2295	2350	2267	2416
K03462 nicotinamide phosphoribosyltransferase [EC:2.4.2.12]	316	300	294	274	392	430	469	434	8	21	7	5
K03517 quinolinate synthase [EC:2.5.1.72]	2648	2927	2791	2837	3454	3494	3466	3475	3065	2987	3012	2910
K03742 nicotinamide-nucleotide amidase [EC:3.5.1.42]	1172	1183	1295	1218	2183	2336	2352	2279	2190	2152	2240	2092
K03743 nicotinamide-nucleotide amidase [EC:3.5.1.42]	1064	1001	1037	979	684	754	695	749	654	601	662	682
K06211 HTH-type transcriptional regulator, transcriptional repressor of NAD biosynthesis genes [EC:2.7.7.1 2.7.1.22]	840	843	815	782	546	548	485	510	1189	1171	1161	1174
K06989 aspartate dehydrogenase [EC:1.4.1.21]	859	938	1007	903	2005	1840	2028	1959	1554	1555	1521	1463
K08281 nicotinamidase/pyrazinamidase [EC:3.5.1.19 3.5.1.-]	1235	1297	1163	1248	683	780	717	738	826	876	870	853
K13522 bifunctional NMN adenylyltransferase/nudix hydrolase [EC:2.7.7.1 3.6.1.-]	3	2	4	9	12	16	11	16	9	10	8	7
K18029 nicotinate dehydrogenase subunit A [EC:1.17.2.1]	749	786	699	827	402	387	388	397	265	216	232	231
K18030 nicotinate dehydrogenase subunit B [EC:1.17.2.1]	1	0	0	0	0	0	1	0	0	1	0	0
K19647 2-hydroxymethylglutarate dehydrogenase [EC:1.1.1.291]	3	0	1	2	0	2	0	0	4	5	1	5
K20445 nicotinate dehydrogenase FAD-subunit [EC:1.17.1.5]	10	5	9	15	3	1	3	2	7	6	6	4
K20446 nicotinate dehydrogenase small FeS subunit [EC:1.17.1.5]	3	2	1	3	0	1	0	0	5	3	2	1
K20447 nicotinate dehydrogenase large molybdopterin subunit [EC:1.17.1.5]	25	31	31	35	11	5	8	13	20	18	19	18
K20448 nicotinate dehydrogenase medium molybdopterin subunit [EC:1.17.1.5]	0	3	0	0	0	0	0	0	0	0	0	1
K20449 6-hydroxynicotinate reductase [EC:1.3.7.1]	0	0	0	1	0	0	0	0	3	5	2	1
K20451 methylitaconate Delta-isomerase [EC:5.3.3.6]	9	3	5	11	0	0	0	0	0	1	3	1
K20452 dimethylmaleate hydratase large subunit [EC:4.2.1.85]	20	17	21	21	13	17	21	21	42	61	50	51
K20453 dimethylmaleate hydratase small subunit [EC:4.2.1.85]	4	2	4	3	1	5	1	10	4	16	14	11
K20454 2,3-dimethylmalate lyase [EC:4.1.3.32]	10	3	6	6	0	0	0	0	6	3	7	5
ko00770 Pantothenate and CoA biosynthesis	63114	62017	61895	62925	55709	56100	54483	55734	58102	59151	58005	59172
K00077 2-dehydropantoate 2-reductase [EC:1.1.1.169]	3579	3561	3309	3444	3587	3491	3369	3663	3820	3883	3711	3763
K00606 3-methyl-2-oxobutanoate hydroxymethyltransferase [EC:2.1.2.11]	3045	2788	2804	2908	3043	2993	2992	3058	2371	2307	2438	2362
K00859 dephospho-CoA kinase [EC:2.7.1.24]	1675	1650	1809	1684	1928	2039	1977	2001	1939	1992	1988	1995
K00867 type I pantothenate kinase [EC:2.7.1.33]	670	646	648	681	465	498	492	476	902	945	829	888
K00954 pantetheine-phosphate adenylyltransferase [EC:2.7.7.3]	1663	1703	1758	1690	1844	1954	1803	1872	1711	1763	1769	1812
K00997 holo-[acyl-carrier protein] synthase [EC:2.7.8.7]	264	284	285	306	170	146	160	140	375	373	337	401
K01598 phosphopantothenoylcysteine decarboxylase [EC:4.1.1.36]	31	43	30	38	5	13	10	7	34	57	26	32
K02201 pantetheine-phosphate adenylyltransferase [EC:2.7.7.3]	0	1	2	0	0	3	1	0	2	0	2	3
K03525 type III pantothenate kinase [EC:2.7.1.33]	921	977	1016	985	1615	1725	1678	1703	1611	1536	1591	1657
K06133 4'-phosphopantetheinyl transferase [EC:2.7.8.-]	159	139	128	124	68	76	77	73	269	248	234	234
K06982 pantoate kinase [EC:2.7.1.169]	0	0	2	0	2	0	0	0	8	1	3	5
K08682 acyl carrier protein phosphodiesterase [EC:3.1.4.14]	454	438	392	407	255	293	263	221	622	670	617	678
K09680 type II pantothenate kinase [EC:2.7.1.33]	469	477	519	513	242	223	254	245	203	268	256	222
K13038 phosphopantothenoylcysteine decarboxylase / phosphopantothenate---cysteine ligase [EC:4.1.1.36 6.3.2.5]	3792	3774	3977	3930	4328	4285	4247	4235	3864	3742	3981	3962
K21977 phosphopantothenate---cysteine ligase (CTP) [EC:6.3.2.5]	30	58	36	59	17	14	13	16	64	53	56	44
ko00780 Biotin metabolism	52419	52001	51297	51644	50898	50582	50622	50821	47169	46759	46602	46867
K00652 8-amino-7-oxononanoate synthase [EC:2.3.1.47]	3864	3973	3828	4002	5987	6010	5968	6186	4780	4732	4738	4513
K00833 adenosylmethionine---8-amino-7-oxononanoate aminotransferase [EC:2.6.1.62]	4656	4722	4614	4617	6637	6775	6836	6557	5454	5287	5334	5309
K01012 biotin synthase [EC:2.8.1.6]	3621	3621	3651	3482	3961	3827	4014	3916	3594	3594	3557	3486
K01906 6-carboxyhexanoate--CoA ligase [EC:6.2.1.14]	36	31	43	36	3	2	3	0	47	51	50	66
K01935 dethiobiotin synthetase [EC:6.3.3.3]	2240	2175	1965	2068	2315	2356	2229	2328	2340	2337	2244	2432
K02169 malonyl-CoA O-methyltransferase [EC:2.1.1.197]	2220	2226	2058	2114	2486	2417	2372	2491	2141	2005	1976	2027
K02170 pimeloyl-[acyl-carrier protein] methyl ester esterase [EC:3.1.1.85]	1319	1318	1166	1214	739	786	761	776	899	892	897	987
K03524 BirA family transcriptional regulator, biotin operon repressor / biotin---[acetyl-CoA-carboxylase] ligase [EC:6.3.4.15]	2884	2920	3011	3065	2971	2878	3016	2861	2984	2990	3044	3041
K08351 biotin/methionine sulfoxide reductase [EC:1.-.-.-]	2765	2777	2770	2965	1999	2199	2088	2057	3616	3592	3542	3745
K09789 pimeloyl-[acyl-carrier protein] methyl ester esterase [EC:3.1.1.85]	420	412	515	460	1345	1381	1374	1372	929	945	947	891
ko00785 Lipoic acid metabolism	5132	5236	5166	5296	6687	6801	6843	6620	6268	6246	6287	6388
K03644 lipoyl synthase [EC:2.8.1.8]	2019	1993	1996	2054	2671	2719	2634	2661	2222	2341	2273	2342
K03800 lipoate---protein ligase [EC:6.3.1.20]	1521	1540	1446	1557	1963	1961	1997	1876	2337	2238	2273	2323
K03801 lipoyl(octanoyl) transferase [EC:2.3.1.181]	1592	1703	1724	1685	2053	2121	2212	2083	1709	1667	1741	1723
ko00790 Folate biosynthesis	58287	58335	57107	58486	56359	55992	56085	56472	50063	49360	49527	49777
K00287 dihydrofolate reductase [EC:1.5.1.3]	1870	1966	1797	1854	2007	1976	1869	1991	1901	1836	1768	1819
K00796 dihydropteroate synthase [EC:2.5.1.15]	2557	2549	2575	2525	3195	3197	3098	3016	2376	2229	2421	2288
K00950 2-amino-4-hydroxy-6-hydroxymethyldihydropteridine diphosphokinase [EC:2.7.6.3]	1869	1999	1975	2000	2617	2627	2654	2564	2174	2141	2140	2191
K01113 alkaline phosphatase D [EC:3.1.3.1]	2489	2529	2267	2456	1483	1253	1253	1372	763	775	716	816
K01495 GTP cyclohydrolase IA [EC:3.5.4.16]	2349	2352	2405	2392	2446	2325	2518	2410	2027	1996	2009	2010
K01633 7,8-dihydroneopterin aldolase/epimerase/oxygenase [EC:4.1.2.25 5.1.99.8 1.13.11.81]	1082	1174	1080	1178	1361	1246	1327	1260	1139	1204	1066	1125
K01664 para-aminobenzoate synthetase component II [EC:2.6.1.85]	396	402	425	402	248	307	336	254	511	543	550	595
K01665 para-aminobenzoate synthetase component I [EC:2.6.1.85]	2908	3036	3014	3122	3426	3505	3416	3479	3038	3042	2995	2953
K01724 4a-hydroxytetrahydrobiopterin dehydratase [EC:4.2.1.96]	540	489	493	470	278	261	249	271	155	150	119	143
K01737 6-pyruvoyltetrahydropterin/6-carboxytetrahydropterin synthase [EC:4.2.3.12 4.1.2.50]	975	1026	920	971	1213	1217	1267	1276	990	1045	987	992
K01930 folylpolyglutamate synthase [EC:6.3.2.17]	0	0	2	1	0	0	1	0	0	1	0	0
K02619 4-amino-4-deoxychorismate lyase [EC:4.1.3.38]	2023	2066	1963	2004	2076	2157	2160	2119	1902	1726	1869	1817
K03635 molybdopterin synthase catalytic subunit [EC:2.8.1.12]	1026	943	931	983	587	635	554	606	718	675	606	651
K03637 cyclic pyranopterin monophosphate synthase [EC:4.6.1.17]	1454	1302	1284	1376	690	796	750	777	884	893	856	875
K03638 molybdopterin adenylyltransferase [EC:2.7.7.75]	1785	1930	1651	1778	950	995	967	1055	896	905	900	1006
K03639 GTP 3',8-cyclase [EC:4.1.99.22]	4352	4054	4045	4159	2344	2304	2291	2440	2369	2216	2268	2430
K03750 molybdopterin molybdotransferase [EC:2.10.1.1]	4770	4504	4363	4442	2670	2749	2553	2789	2549	2829	2764	2924
K03752 molybdenum cofactor guanylyltransferase [EC:2.7.7.77]	1288	1276	1185	1245	691	762	741	713	821	799	775	822
K03831 molybdopterin adenylyltransferase [EC:2.7.7.75]	403	442	396	477	348	379	342	359	615	612	637	668
K06879 7-cyano-7-deazaguanine reductase [EC:1.7.1.13]	1563	1628	1546	1649	1008	987	1003	1015	1163	1150	1049	1202
K06897 7,8-dihydropterin-6-yl-methyl-4-(beta-D-ribofuranosyl)aminobenzene 5'-phosphate synthase [EC:2.5.1.105]	538	566	663	592	1360	1291	1391	1342	1345	1442	1357	1270
K06920 7-cyano-7-deazaguanine synthase [EC:6.3.4.20]	1614	1602	1727	1862	2139	2234	2088	2091	1981	1870	1820	1721
K06984 beta-ribofuranosylaminobenzene 5'-phosphate synthase [EC:2.4.2.54]	0	0	2	0	0	0	0	2	5	4	8	5
K07141 molybdenum cofactor cytidylyltransferase [EC:2.7.7.76]	1371	1336	1194	1399	651	631	634	596	632	663	652	599
K07142 2-amino-4-hydroxy-6-hydroxymethyldihydropteridine diphosphokinase [EC:2.7.6.3]	2	2	1	0	0	0	0	0	4	0	0	10

K07589 D-erythro-7,8-dihydroneopterin triphosphate epimerase [EC:5.1.99.7]	710	719	711	738	412	411	452	487	460	460	431	422
K08310 dihydroneopterin triphosphate diphosphatase [EC:3.6.1.67]	267	215	243	272	207	200	185	191	345	336	349	334
K09007 GTP cyclohydrolase IB [EC:3.5.4.16]	1681	1672	1617	1648	929	927	955	995	582	581	526	602
K09457 7-cyano-7-deazaguanine reductase [EC:1.7.1.13]	280	306	328	315	969	913	1012	960	718	766	700	668
K09739 dihydroneopterin aldolase [EC:4.1.2.25]	0	0	2	0	1	0	0	0	1	3	1	4
K10026 7-carboxy-7-deazaguanine synthase [EC:4.3.99.3]	1826	1828	1887	1846	2044	2061	2148	2107	1737	1666	1679	1801
K11754 dihydrofolate synthase / folylpolyglutamate synthase [EC:6.3.2.12 6.3.2.17]	3561	3514	3520	3516	4296	4091	4236	4304	4116	3981	4245	4130
K13818 molybdopterin-guanine dinucleotide biosynthesis protein [EC:2.7.7.77]	49	35	55	46	30	41	35	36	16	30	15	20
K13938 dihydromonapterin reductase / dihydrofolate reductase [EC:1.5.1.50 1.5.1.3]	761	842	766	768	473	460	376	451	711	632	630	692
K13939 dihydroneopterin aldolase / 2-amino-4-hydroxy-6-hydroxymethyldihydropteridine diphosphokinase / dihydropteroate synthase [EC:4.1.2.25 2.7.6.3 2	0	0	2	0	0	0	0	0	0	0	0	0
K13940 dihydroneopterin aldolase / 2-amino-4-hydroxy-6-hydroxymethyldihydropteridine diphosphokinase [EC:4.1.2.25 2.7.6.3]	87	107	104	111	23	41	24	24	154	181	153	200
K13950 para-aminobenzoate synthetase [EC:2.6.1.85]	0	0	2	1	0	0	0	0	0	0	1	1
K15376 gephyrin [EC:2.10.1.1 2.7.7.75]	1	0	3	3	1	1	4	2	0	2	2	3
K17488 GTP cyclohydrolase IV [EC:3.5.4.39]	0	2	0	1	2	5	0	0	1	9	3	8
K18590 dihydrofolate reductase (trimethoprim resistance protein) [EC:1.5.1.3]	0	0	0	0	0	1	0	0	0	0	0	1
K18824 dihydropteroate synthase type 2 [EC:2.5.1.15]	0	0	0	0	0	0	0	0	0	3	0	1
K18853 dihydromethanopterin reductase (acceptor) [EC:1.5.99.15]	2	0	3	0	0	0	0	0	0	2	2	6
K18974 dihydropteroate synthase type 1 [EC:2.5.1.15]	0	0	0	0	3	0	2	2	4	0	2	2
K20967 GTP 3',8-cyclase / cyclic pyranopterin monophosphate synthase [EC:4.1.99.22 4.6.1.17]	0	0	0	0	0	0	0	3	1	0	0	0
ko00670 One carbon pool by folate	54772	54409	54069	54069	59811	59720	60266	59574	56390	54279	54334	54214
K00288 methylenetetrahydrofolate dehydrogenase (NADP+) / methenyltetrahydrofolate cyclohydrolase / formyltetrahydrofolate synthetase [EC:1.5.1.5 3.5.4	0	0	1	0	0	0	0	2	0	0	0	0
K00604 methionyl-tRNA formyltransferase [EC:2.1.2.9]	3337	3486	3389	3382	3518	3551	3628	3484	3402	3362	3462	3391
K01500 methenyltetrahydrofolate cyclohydrolase [EC:3.5.4.9]	20	9	9	7	1	3	0	3	11	14	20	15
K01746 formiminotetrahydrofolate cyclodeaminase [EC:4.3.1.4]	1	0	0	0	34	28	34	21	3	4	4	7
K01934 5-formyltetrahydrofolate cyclo-ligase [EC:6.3.3.2]	1982	1962	1802	1880	2027	2058	1962	1890	1803	1750	1746	1715
K13403 methylenetetrahydrofolate dehydrogenase(NAD+) / 5,10-methenyltetrahydrofolate cyclohydrolase [EC:1.5.1.15 3.5.4.9]	0	0	1	0	0	0	0	0	0	1	1	0
ko00830 Retinol metabolism	5941	6112	5761	5748	3891	3929	3897	3874	4653	4767	4640	4615
K07249 retinal dehydrogenase [EC:1.2.1.36]	0	1	0	0	0	0	0	0	1	0	0	0
K09516 all-trans-retinol 13,14-reductase [EC:1.3.99.23]	166	209	195	173	300	277	335	302	19	50	29	34
K11153 retinol dehydrogenase 12 [EC:1.1.1.300]	1	0	0	0	0	1	0	0	0	0	1	0
K15734 all-trans-retinol dehydrogenase (NAD+) [EC:1.1.1.105]	0	0	1	1	0	0	0	0	0	0	0	0
ko00860 Porphyrin and chlorophyll metabolism	107231	106167	101632	103492	85572	84623	83570	85907	87872	86755	86125	88181
K00228 coproporphyrinogen III oxidase [EC:1.3.3.3]	1705	1678	1563	1636	1004	1006	907	1004	1132	1089	1047	1205
K00230 menaquinone-dependent protoporphyrinogen oxidase [EC:1.3.5.3]	643	656	658	601	1344	1476	1412	1398	1393	1233	1347	1301
K00231 protoporphyrinogen/coproporphyrinogen III oxidase [EC:1.3.3.4 1.3.3.15]	21	19	9	23	3	4	2	1	5	6	2	3
K00435 Fe-coproporphyrin III decarboxylase [EC:1.11.1.-]	5	1	16	4	0	2	0	0	0	0	0	0
K00595 precorrin-6Y C5,15-methyltransferase (decarboxylating) [EC:2.1.1.132]	2523	2527	2392	2232	2508	2397	2435	2517	1992	1918	1940	1911
K00768 nicotinate-nucleotide--dimethylbenzimidazole phosphoribosyltransferase [EC:2.4.2.21]	3496	3344	3195	3212	3467	3553	3463	3463	4066	4046	4190	4181
K00798 cob(I)alamin adenosyltransferase [EC:2.5.1.17]	1525	1524	1519	1485	1838	1768	1950	1872	1274	1233	1348	1355
K01599 uroporphyrinogen decarboxylase [EC:4.1.1.37]	4728	4731	4722	4832	4010	3847	3962	3800	5051	5254	5075	5250
K01698 porphobilinogen synthase [EC:4.2.1.24]	1666	1702	1755	1713	1127	1159	1066	1119	1610	1740	1710	1633
K01719 uroporphyrinogen-III synthase [EC:4.2.1.75]	2077	2174	2045	2125	2745	2754	2751	2744	2278	2258	2234	2258
K01749 hydroxymethylbilane synthase [EC:2.5.1.61]	2741	2700	2590	2731	1548	1509	1568	1546	1858	1789	1711	1825
K01772 protoporphyrin/coproporphyrin ferrochelatase [EC:4.99.1.1 4.99.1.9]	2321	2326	2149	2417	1410	1385	1369	1402	1427	1399	1430	1451
K01845 glutamate-1-semialdehyde 2,1-aminomutase [EC:5.4.3.8]	4650	4500	4416	4575	2808	2739	2266	2515	2773	2796	2700	2837
K01885 glutamyl-tRNA synthetase [EC:6.1.1.17]	4654	4628	4821	4547	5461	5490	5648	5570	5240	5181	5266	5201
K02188 cobalt-precorrin-5B (C1)-methyltransferase [EC:2.1.1.195]	2691	2742	2441	2512	2670	2616	2549	2681	2672	2605	2617	2604
K02189 cobalt-precorrin 5A hydrolase [EC:3.7.1.12]	1400	1388	1294	1422	803	711	690	808	1327	1310	1346	1356
K02190 sirohydrochlorin cobaltochelatase [EC:4.99.1.3]	1394	1517	1392	1537	2321	2366	2446	2294	2292	2211	2297	2212
K02191 cobalt-precorrin-6B (C15)-methyltransferase [EC:2.1.1.196]	340	336	315	309	219	228	207	224	346	341	319	361
K02224 cobyrinic acid a,c-diamide synthase [EC:6.3.5.9 6.3.5.11]	3600	3658	3339	3466	3065	2838	2823	3001	3075	3041	2956	3034
K02225 cobalamin biosynthetic protein CobC	1352	1390	1102	1185	678	657	558	642	424	331	346	395
K02226 alpha-ribazole phosphatase [EC:3.1.3.73]	1656	1656	1589	1579	1915	1876	1886	1880	1761	1676	1748	1696
K02227 adenosylcobinamide-phosphate synthase [EC:6.3.1.10]	2266	2255	2046	2109	1745	1598	1468	1659	1636	1659	1666	1730
K02229 precorrin-3B synthase [EC:1.14.13.83]	1437	1389	1114	1231	608	611	628	761	414	366	340	416
K02230 cobaltochelatase CobN [EC:6.6.1.2]	10190	10207	9284	9685	5545	5371	5200	5913	3310	3008	2924	3061
K02231 adenosylcobinamide kinase / adenosylcobinamide-phosphate guanylyltransferase [EC:2.7.1.156 2.7.7.62]	1568	1572	1478	1543	1596	1572	1593	1530	1548	1489	1579	1625
K02232 adenosylcobyric acid synthase [EC:6.3.5.10]	3752	3683	3480	3543	2597	2595	2562	2602	3047	3109	2919	3026
K02233 adenosylcobinamide-GDP ribazoletransferase [EC:2.7.8.26]	2102	2147	2069	2043	2459	2477	2280	2476	2285	2246	2388	2309
K02234 cobalamin biosynthesis protein CobW	1692	1648	1506	1438	853	763	694	727	494	493	419	485
K02302 uroporphyrin-III C-methyltransferase / precorrin-2 dehydrogenase / sirohydrochlorin ferrochelatase [EC:2.1.1.107 1.3.1.76 4.99.1.4]	2627	2711	2438	2655	1564	1675	1647	1602	2539	2649	2605	2650
K02303 uroporphyrin-III C-methyltransferase [EC:2.1.1.107]	2540	2582	2452	2542	1472	1467	1417	1445	812	693	715	777
K02304 precorrin-2 dehydrogenase / sirohydrochlorin ferrochelatase [EC:1.3.1.76 4.99.1.4]	239	210	208	241	52	59	45	57	227	238	244	246
K02492 glutamyl-tRNA reductase [EC:1.2.1.70]	3206	3030	3076	2865	1904	1693	1830	1865	2167	2095	2028	2120
K02495 oxygen-independent coproporphyrinogen III oxidase [EC:1.3.98.3]	1933	1963	2004	1974	1293	1287	1341	1257	1671	1782	1621	1736
K02496 uroporphyrin-III C-methyltransferase [EC:2.1.1.107]	2751	2649	2568	2620	1636	1609	1527	1599	1570	1506	1495	1559
K03394 precorrin-2/cobalt-factor-2 C20-methyltransferase [EC:2.1.1.130 2.1.1.151]	1890	1885	1725	1834	978	956	1001	1056	1240	1266	1230	1234
K03399 cobalt-precorrin-7 (C5)-methyltransferase [EC:2.1.1.289]	450	366	350	360	244	256	210	208	542	522	497	540
K03404 magnesium chelatase subunit D [EC:6.6.1.1]	76	93	95	64	8	4	5	2	82	90	95	121
K03405 magnesium chelatase subunit I [EC:6.6.1.1]	356	357	324	316	159	176	190	211	165	136	135	172
K03594 bacterioferritin [EC:1.16.3.1]	2524	2510	2698	2466	2382	2384	2170	2433	1866	1864	1890	1780
K04032 ethanolamine utilization cobalamin adenosyltransferase [EC:2.5.1.17]	328	275	327	334	83	99	106	107	690	732	654	810
K04034 anaerobic magnesium-protoporphyrin IX monomethyl ester cyclase [EC:1.21.98.3]	3	1	3	1	1	0	0	0	5	9	5	6
K04720 threonine-phosphate decarboxylase [EC:4.1.1.81]	1353	1439	1470	1432	1879	1811	1959	1945	2207	2261	2235	2173
K05895 precorrin-6A/cobalt-precorrin-6A reductase [EC:1.3.1.54 1.3.1.106]	1412	1332	1208	1373	745	731	710	796	1056	1057	988	1052
K05934 precorrin-3B C17-methyltransferase [EC:2.1.1.131]	600	600	558	583	319	314	339	343	769	812	699	788
K05936 precorrin-4/cobalt-precorrin-4 C11-methyltransferase [EC:2.1.1.133 2.1.1.271]	1988	1775	1744	1822	954	955	888	930	1301	1257	1141	1277
K06042 precorrin-8X/cobalt-precorrin-8 methylmutase [EC:5.4.99.61 5.4.99.60]	1366	1374	1266	1262	562	610	627	659	988	882	972	1090
K07145 heme oxygenase (staphylobilin-producing) [EC:1.14.99.48]	1	0	0	0	0	0	0	0	0	0	0	0
K07215 heme oxygenase (biliverdin-IX-beta and delta-forming) [EC:1.14.99.58]	684	709	666	685	327	384	342	342	247	227	192	196
K08260 adenosylcobinamide hydrolase [EC:3.5.1.90]	1	2	2	2	1	0	0	0	4	2	11	6
K09882 cobaltochelatase CobS [EC:6.6.1.2]	34	46	44	54	33	20	31	43	69	66	45	74
K09883 cobaltochelatase CobT [EC:6.6.1.2]	1	0	0	0	2	2	0	0	5	3	3	4
K13541 cobalt-precorrin 5A hydrolase / precorrin-3B C17-methyltransferase [EC:3.7.1.12 2.1.1.131]	1939	1966	1599	1746	881	948	956	1098	599	540	495	528
K13542 uroporphyrinogen III methyltransferase / synthase [EC:2.1.1.107 4.2.1.75]	678	632	641	639	131	162	167	132	708	756	724	866
K13543 uroporphyrinogen III methyltransferase / synthase [EC:2.1.1.107 4.2.1.75]	34	39	44	45	42	30	51	67	57	51	57	50
K13786 cob(II)yrinic acid a,c-diamide reductase [EC:1.16.8.1]	2	0	0	0	0	0	0	2	3	0	2	1
K16651 L-threonine kinase [EC:2.7.1.177]	593	507	552	534	379	351	383	398	736	731	658	786
K19221 cob(I)alamin adenosyltransferase [EC:2.5.1.17]	940	858	927	976	430	440	437	449	910	920	893	966
K19712 adenosylcobinamide-phosphate guanylyltransferase [EC:2.7.7.62]	0	2	2	1	0	0	0	0	2	2	3	1
K21479 cobalt-factor III methyltransferase [EC:2.1.1.272]	1	0	0	0	0	0	0	5	0	0	0	0
K21480 heme oxygenase (biliverdin-producing, ferredoxin) [EC:1.14.15.20]	1	0	0	0	0	6	0	1	0	0	0	0
K21610 Ni-sirohydrochlorin a,c-diamide reductive cyclase subunit CfbC [EC:6.3.3.7]	0	0	0	0	0	0	0	0	0	2	0	12
K21611 Ni-sirohydrochlorin a,c-diamide reductive cyclase subunit CfbD [EC:6.3.3.7]	2	0	0	0	0	2	0	0	3	3	3	9
K21612 coenzyme F430 synthetase [EC:6.4.1.9]	3	2	1	1	0	2	2	0	5	13	3	7
K22011 sirohydrochlorin cobalto/nickelchelatase [EC:4.99.1.3 4.99.1.11]	0	0	0	0	0	0	3	0	6	6	5	10
K22012 Ni-sirohydrochlorin a,c-diamide synthase [EC:6.3.5.12]	0	0	0	0	0	0	0	0	0	0	0	1
K22226 Fe-coproporphyrin III synthase	18	10	14	18	12	6	8	9	22	31	27	26
K22227 heme synthase	480	396	463	447	306	371	371	385	124	201	198	181
K22316 ribonuclease H / adenosylcobalamin/alpha-ribazole phosphatase [EC:3.1.26.4 3.1.3.73]	1	2	2	4	0	0	0	0	0	4	3	1
ko00130 Ubiquinone and other terpenoid-quinone biosynthesis	45775	46182	44534	45669	37405	38116	37464	37982	38645	38412	38247	38384
K00355 NAD(P)H dehydrogenase (quinone) [EC:1.6.5.2]	2683	2813	2569	2767	1666	1603	1531	1798	1381	1306	1406	1253
K00568 2-polyprenyl-6-hydroxyphenyl methylase / 3-demethylubiquinone-9 3-methyltransferase [EC:2.1.1.222 2.1.1.64]	1445	1476	1336	1398	860	859	847	802	868	968	954	937
K01075 4-hydroxybenzoyl-CoA thioesterase [EC:3.1.2.23]	527	565	585	501	338	317	295	337	133	180	160	180
K01661 naphthoate synthase [EC:4.1.3.36]	1142	1140	1181	1180	2133	2077	2150	2162	2048	2051	2008	1901
K01851 salicylate biosynthesis isochorismate synthase [EC:5.4.4.2]	2193	2185	2061	1980	1178	1199	1080	1193	779	777	587	649
K01904 4-coumarate--CoA ligase [EC:6.2.1.12]	1	0	0	0	0	1	0	0	0	0	0	0
K01911 O-succinylbenzoic acid---CoA ligase [EC:6.2.1.26]	1651	1572	1666	1659	2798	2936	3019	2972	2962	2813	2866	2796
K02361 isochorismate synthase [EC:5.4.4.2]	1345	1495	1415	1408	2784	3006	2892	2783	2615	2573	2648	2626
K02548 1,4-dihydroxy-2-naphthoate polyprenyltransferase [EC:2.5.1.74]	1290	1336	1345	1315	2460	2458	2675	2391	2221	2330	2334	2267
K02549 O-succinylbenzoate synthase [EC:4.2.1.113]	736	716	638	736	429	446	419	443	910	981	924	960
K02551 2-succinyl-5-enolpyruvyl-6-hydroxy-3-cyclohexene-1-carboxylate synthase [EC:2.2.1.9]	2671	2693	2629	2639	4076	4220	4122	4083	4170	4050	4277	4098
K02552 menaquinone-specific isochorismate synthase [EC:5.4.4.2]	860	860	803	841	481	524	516	543	1116	1202	1125	1156
K03179 4-hydroxybenzoate polyprenyltransferase [EC:2.5.1.39]	2082	2081	2030	2079	1232	1303	1197	1260	1256	1232	1148	1187
K03181 chorismate--pyruvate lyase [EC:4.1.3.40]	1232	1303	1216	1406	749	834	776	899	901	851	829	810
K03182 4-hydroxy-3-polyprenylbenzoate decarboxylase [EC:4.1.1.98]	6185	6218	6215	6471	3644	3761	3422	3624	3197	3151	3203	3226
K03183 demethylmenaquinone methyltransferase / 2-methoxy-6-polyprenyl-1,4-benzoquinol methylase [EC:2.1.1.163 2.1.1.201]	2175	2244	2323	2195	2667	2669	2821	2595	2381	2414	2521	2380
K03184 3-demethoxyubiquinol 3-hydroxylase [EC:1.14.99.60]	786	769	723	714	483	443	488	486	1071	1096	981	1065
K03185 2-octaprenyl-6-methoxyphenol hydroxylase [EC:1.14.13.-]	2494	2664	2552	2625	1547	1583	1496	1534	1578	1548	1495	1661
K03186 flavin prenyltransferase [EC:2.5.1.129]	1990	1929	1674	1811	1097	1146	1155	1146	1469	1430	1460	1401
K03809 NAD(P)H dehydrogenase (quinone) [EC:1.6.5.2]	1252	1300	1317	1289	760	721	804	699	726	724	718	822

K06127 2-methoxy-6-polyprenyl-1,4-benzoquinol methylase [EC:2.1.1.201]	1	0	0	0	2	0	1	3	0	0	0	0
K06134 3-demethoxyubiquinol 3-hydroxylase [EC:1.14.99.60]	770	762	781	739	454	457	470	411	257	249	259	219
K08680 2-succinyl-6-hydroxy-2,4-cyclohexadiene-1-carboxylate synthase [EC:4.2.99.20]	520	525	477	506	284	302	311	302	632	697	627	674
K11782 chorismate dehydratase [EC:4.2.1.151]	9	8	5	5	7	11	3	6	5	5	5	7
K11783 futalosine hydrolase [EC:3.2.2.26]	2	3	2	1	3	4	3	0	7	7	4	8
K11784 cyclic dehypoxanthinyl futalosine synthase [EC:1.21.98.1]	7	2	10	8	5	18	8	2	10	15	6	14
K11785 1,4-dihydroxy-6-naphthoate synthase [EC:1.14.-.-]	3	5	9	3	3	10	5	13	18	11	8	11
K18285 aminodeoxyfutalosine synthase [EC:2.5.1.120]	19	5	9	9	3	11	9	7	12	19	13	15
K18286 aminodeoxyfutalosine deaminase [EC:3.5.4.40]	1	2	2	1	0	0	0	0	0	0	0	0
K18800 2-polyprenylphenol 6-hydroxylase [EC:1.14.13.240]	3061	3005	2822	2994	1750	1682	1716	1760	1871	1709	1746	1833
K19222 1,4-dihydroxy-2-naphthoyl-CoA hydrolase [EC:3.1.2.28]	884	927	750	855	504	472	466	523	539	492	490	534
K19267 NAD(P)H dehydrogenase (quinone) [EC:1.6.5.2]	481	519	494	596	333	434	443	396	752	814	697	796
K20810 aminodeoxyfutalosine deaminase [EC:3.5.4.40]	1	0	0	0	1	1	0	0	0	0	0	1
Metabolism of terpenoids and polyketides	154125	153253	148955	151067	124182	123608	122819	122769	130332	129860	127856	130163
ko00900 Terpenoid backbone biosynthesis	46655	47022	46688	46804	47838	47169	47643	47051	44876	45329	45135	44698
K00021 hydroxymethylglutaryl-CoA reductase (NADPH) [EC:1.1.1.34]	0	0	0	0	0	0	0	0	0	2	10	3	9
K00054 hydroxymethylglutaryl-CoA reductase [EC:1.1.1.88]	63	55	59	51	8	18	5	7	64	75	75	66
K00099 1-deoxy-D-xylulose-5-phosphate reductoisomerase [EC:1.1.1.267]	3978	4155	4145	4129	4369	4365	4375	4273	4072	4161	4110	4079
K00795 farnesyl diphosphate synthase [EC:2.5.1.1 2.5.1.10]	584	611	556	619	505	469	444	417	816	909	835	873
K00805 heptaprenyl diphosphate synthase [EC:2.5.1.30]	351	403	389	348	107	85	69	98	330	370	427	397
K00806 undecaprenyl diphosphate synthase [EC:2.5.1.31]	2035	2016	2098	2092	2578	2639	2695	2553	2507	2686	2570	2686
K00869 mevalonate kinase [EC:2.7.1.36]	75	49	52	58	26	16	15	10	53	94	76	68
K00919 4-diphosphocytidyl-2-C-methyl-D-erythritol kinase [EC:2.7.1.148]	2604	2616	2691	2541	2838	2750	2710	2558	2734	2690	2845	2647
K00938 phosphomevalonate kinase [EC:2.7.4.2]	62	67	70	68	24	16	19	18	82	104	75	87
K00991 2-C-methyl-D-erythritol 4-phosphate cytidylyltransferase [EC:2.7.7.60]	2549	2397	2531	2609	2428	2371	2597	2526	2422	2523	2344	2422
K01597 diphosphomevalonate decarboxylase [EC:4.1.1.33]	49	79	58	61	17	10	13	16	52	78	73	73
K01770 2-C-methyl-D-erythritol 2,4-cyclodiphosphate synthase [EC:4.6.1.12]	1547	1699	1652	1685	1785	1777	1782	1705	1619	1705	1652	1719
K01823 isopentenyl-diphosphate Delta-isomerase [EC:5.3.3.2]	436	490	434	506	261	294	302	286	631	695	621	606
K02523 octaprenyl-diphosphate synthase [EC:2.5.1.90]	2606	2446	2653	2517	3389	3610	3615	3531	3009	2964	3020	2948
K03526 (E)-4-hydroxy-3-methylbut-2-enyl-diphosphate synthase [EC:1.17.7.1 1.17.7.3]	4251	4405	4447	4420	6126	6050	6161	5977	5224	4958	5337	4994
K03527 4-hydroxy-3-methylbut-2-en-1-yl diphosphate reductase [EC:1.17.7.4]	2847	2824	2873	2679	3256	3123	3159	3158	3313	3252	3240	3215
K06013 STE24 endopeptidase [EC:3.4.24.84]	9	4	2	3	9	9	11	7	4	5	10	13
K06981 isopentenyl phosphate kinase [EC:2.7.4.26]	0	3	3	0	1	0	0	1	2	6	8	4
K12506 2-C-methyl-D-erythritol 4-phosphate cytidylyltransferase / 2-C-methyl-D-erythritol 2,4-cyclodiphosphate synthase [EC:2.7.7.60 4.6.1.12]	63	60	63	70	62	72	67	99	90	77	85	77
K13787 geranylgeranyl diphosphate synthase, type I [EC:2.5.1.1 2.5.1.10 2.5.1.29]	11	9	11	16	10	7	7	6	31	20	21	23
K13789 geranylgeranyl diphosphate synthase, type II [EC:2.5.1.1 2.5.1.10 2.5.1.29]	2253	2148	2200	2235	2812	2752	2832	2709	2146	2176	2251	2226
K15888 tritrans,polycis-undecaprenyl-diphosphate synthase [geranylgeranyl-diphosphate specific] [EC:2.5.1.89]	1	1	0	0	2	2	0	0	5	3	3	10
K21273 trans,polycis-polyprenyl diphosphate synthase [EC:2.5.1.88]	1	0	1	0	0	1	0	0	0	0	0	0
ko00902 Monoterpenoid biosynthesis	1	0	0	0	0	0	0	0	0	1	0	0
K15095 (+)-neomenthol dehydrogenase [EC:1.1.1.208]	1	0	0	0	0	0	0	0	0	0	0	0
K23232 8-hydroxygeraniol dehydrogenase [EC:1.1.1.324]	0	0	0	0	0	0	0	0	0	1	0	0
ko00909 Sesquiterpenoid and triterpenoid biosynthesis	3	0	2	3	0	0	0	0	0	0	0	0
K06045 squalene-hopene/tetraprenyl-beta-curcumene cyclase [EC:5.4.99.17 4.2.1.129]	3	0	2	3	0	0	0	0	0	0	0	0
ko00906 Carotenoid biosynthesis	29	53	60	56	43	55	56	50	125	101	88	98
K02291 15-cis-phytoene synthase [EC:2.5.1.32]	10	16	12	25	14	20	18	20	42	37	26	34
K09835 prolycopene isomerase [EC:5.2.1.13]	1	0	2	2	0	0	0	0	1	1	3	1
K10027 phytoene desaturase [EC:1.3.99.26 1.3.99.28 1.3.99.29 1.3.99.31]	14	24	35	25	24	35	32	27	72	53	55	54
K10210 diapolycopene oxygenase [EC:1.14.99.44]	2	6	5	3	0	0	6	3	6	2	2	6
K10211 4,4'-diaponeurosporenoate glycosyltransferase [EC:2.4.1.-]	0	5	4	1	0	0	0	0	4	0	2	2
K10212 glycosyl-4,4'-diaponeurosporenoate acyltransferase [EC:2.3.1.-]	2	2	2	0	5	0	0	0	0	4	0	1
K15746 beta-carotene 3-hydroxylase [EC:1.14.15.24]	0	0	0	0	0	0	0	0	0	4	0	0
ko00981 Insect hormone biosynthesis	5102	5084	4807	4818	3106	2867	2785	3017	1916	1755	1686	1825
K14938 cholesterol 7-desaturase [EC:1.14.19.21]	0	0	0	0	0	0	0	0	0	1	0	0
ko00908 Zeatin biosynthesis	3123	3207	3387	3217	5160	5000	5206	4962	4225	4307	4377	4260
K00791 tRNA dimethylallyltransferase [EC:2.5.1.75]	3123	3207	3387	3217	5160	5000	5206	4962	4225	4307	4377	4260
ko00903 Limonene and pinene degradation	16698	16373	16025	16123	9818	9523	9105	9778	8914	8576	8466	9038
K14731 epsilon-lactone hydrolase [EC:3.1.1.83]	92	97	120	106	24	27	52	33	118	121	151	167
ko00281 Geraniol degradation	32077	31388	30074	30983	17949	17595	16844	17810	15019	14526	14201	14848
K11731 citronellyl-CoA dehydrogenase [EC:1.3.99.-]	1806	1704	1660	1740	939	903	941	923	511	506	498	480
K13774 citronellol/citronellal dehydrogenase	849	907	840	831	449	471	408	457	280	260	252	275
K13775 citronellol/citronellal dehydrogenase	1202	1233	1030	1152	659	603	543	642	388	373	421	352
K13776 citronellyl-CoA synthetase [EC:6.2.1.-]	2534	2508	2308	2469	1315	1348	1220	1343	794	764	691	764
K13777 geranyl-CoA carboxylase alpha subunit [EC:6.4.1.5]	2904	2884	2601	2663	1520	1451	1323	1417	892	864	787	827
K13778 geranyl-CoA carboxylase beta subunit [EC:6.4.1.5]	2360	2282	2336	2284	1279	1250	1202	1241	726	681	668	638
K13779 isohexenylglutaconyl-CoA hydratase [EC:4.2.1.57]	818	731	646	765	407	391	378	481	286	228	200	254
ko01052 Type I polyketide structures	0	1	1	0	1	1	2	5	7	7	8	2
K16378 pipecolate-incorporating enzyme	0	0	0	0	0	1	2	3	4	6	8	1
K16384 polyene macrolide polyketide synthase	0	0	0	0	1	0	0	1	2	0	0	1
K16395 epothilone synthetase B	0	1	1	0	0	0	0	0	1	1	0	0
K16397 epothilone polyketide synthase D	0	0	0	0	0	0	0	1	0	0	0	0
ko01051 Biosynthesis of ansamycins	13517	13414	13461	13442	9604	9691	9778	9303	14442	14290	14105	14499
ko01059 Biosynthesis of enediyne antibiotics	0	0	0	0	0	0	0	0	0	0	0	1
K15320 6-methylsalicylic acid synthase [EC:2.3.1.165]	0	0	0	0	0	0	0	0	0	0	0	1
ko01056 Biosynthesis of type II polyketide backbone	2	1	0	0	0	0	0	0	2	1	0	1
K12420 ketoreductase [EC:1.1.1.-]	2	1	0	0	0	0	0	0	2	1	0	1
ko01057 Biosynthesis of type II polyketide products	0	0	0	0	0	0	0	0	0	1	0	0
K05556 ketoreductase RED1 [EC:1.1.1.-]	0	0	0	0	0	0	0	0	0	1	0	0
ko00253 Tetracycline biosynthesis	857	898	977	897	3124	3092	3110	3055	2340	2086	2325	2189
K18221 tetracycline 11a-monooxygenase, tetracycline resistance protein [EC:1.14.13.231]	855	897	977	897	3124	3092	3110	3055	2338	2085	2325	2188
ko00523 Polyketide sugar unit biosynthesis	14902	15109	15013	14959	16606	17024	17232	16617	13995	14146	13887	13846
K00067 dTDP-4-dehydrorhamnose reductase [EC:1.1.1.133]	4197	4324	4281	4273	4572	4728	4685	4483	3463	3571	3423	3316
K00973 glucose-1-phosphate thymidylyltransferase [EC:2.7.7.24]	4335	4362	4461	4283	5425	5406	5669	5509	4932	4888	4972	4998
K01710 dTDP-glucose 4,6-dehydratase [EC:4.2.1.46]	4263	4310	4172	4262	4525	4735	4663	4518	3965	4125	3926	3887
K01790 dTDP-4-dehydrorhamnose 3,5-epimerase [EC:5.1.3.13]	2099	2103	2089	2123	2084	2149	2213	2106	1619	1546	1541	1629
K13315 NDP-hexose C3-ketoreductase / dTDP-4-oxo-2-deoxy-alpha-D-pentos-2-ene 2,3-reductase [EC:1.1.1.-]	6	5	6	10	0	0	0	0	3	4	3	5
K13327 dTDP-3,4-didehydro-2,6-dideoxy-alpha-D-glucose 3-reductase [EC:1.1.1.384]	0	0	0	3	0	0	0	0	3	0	2	0
K13328 dTDP-4-dehydro-2,6-dideoxy-D-glucose 3-dehydratase [EC:4.2.1.164]	0	0	0	0	0	0	0	1	0	0	0	0
K16435 dTDP-4-dehydro-6-deoxy-alpha-D-glucopyranose 2,3-dehydratase [EC:4.2.1.159]	0	3	2	3	0	4	0	0	3	5	8	3
K16436 dTDP-3-amino-2,3,6-trideoxy-4-keto-D-glucose/dTDP-3-amino-3,4,6-trideoxy-alpha-D-glucose/dTDP-2,6-dideoxy-D-kanosamine transaminase [EC:2.6	0	2	0	2	0	2	0	0	0	0	0	1
K23144 UDP-N-acetylglucosamine diphosphorylase / glucose-1-phosphate thymidylyltransferase / UDP-N-acetylgalactosamine diphosphorylase / glucosamine	2	0	2	0	0	0	2	0	7	7	12	7
ko01054 Nonribosomal peptide structures	545	573	494	584	430	483	410	398	391	459	418	429
K15654 surfactin family lipopeptide synthetase A	1	1	1	0	0	1	0	0	2	1	0	3
K15655 surfactin family lipopeptide synthetase B	0	0	0	0	0	0	0	0	2	1	0	1
K15656 surfactin family lipopeptide synthetase C	3	0	0	1	0	0	0	0	0	0	0	0
K15662 iturin family lipopeptide synthetase B	2	2	2	3	1	0	0	0	1	5	3	3
K15664 fengycin family lipopeptide synthetase A	0	0	0	0	0	0	0	0	0	1	0	0
K15665 fengycin family lipopeptide synthetase B	0	1	0	1	0	0	0	0	1	2	1	1
K15667 fengycin family lipopeptide synthetase D	1	0	0	0	0	0	0	0	1	0	0	0
K15668 fengycin family lipopeptide synthetase E	0	0	0	0	0	0	0	0	0	0	0	1
K16093 bacitracin synthase 1	1	2	1	1	1	0	0	0	4	5	1	3
K16094 bacitracin synthase 2	3	0	2	2	2	0	1	0	1	1	0	1
K16095 bacitracin synthase 3	3	2	2	2	0	0	0	0	4	3	2	3
K16119 lichenysin synthetase A	0	0	0	0	0	0	0	0	1	3	2	1
K16122 tyrocidine synthetase I	0	0	1	0	0	0	0	0	0	0	0	0
K16124 tyrocidine synthetase III	1	0	2	0	0	0	0	0	0	0	0	0
K16129 microcystin synthetase protein McyE	0	0	0	0	0	0	0	0	0	1	0	0
K16133 microcystin synthetase protein McyI	0	0	0	0	0	0	0	0	0	2	1	0
ko01053 Biosynthesis of siderophore group nonribosomal peptides	37222	36407	33871	35198	20296	20603	19701	20468	32877	32731	31475	33301
K00216 2,3-dihydro-2,3-dihydroxybenzoate dehydrogenase [EC:1.3.1.28]	458	465	427	532	313	313	384	333	630	625	643	696
K01252 bifunctional isochorismate lyase / aryl carrier protein [EC:3.3.2.1 6.3.2.14]	599	509	515	536	359	338	413	351	703	748	688	789
K02362 enterobactin synthetase component D [EC:6.3.2.14 2.7.8.-]	603	666	545	609	402	415	355	378	581	599	561	601
K02363 2,3-dihydroxybenzoate-AMP ligase [EC:6.3.2.14 2.7.7.58]	976	1012	803	910	602	722	697	582	1354	1379	1363	1418
K02364 enterobactin synthetase component F [EC:6.3.2.14]	2262	2096	1927	2212	1656	1688	1535	1523	2661	2674	2612	2816
K04780 nonribosomal peptide synthetase DhbF	0	0	0	0	1	0	0	0	0	0	0	0
K04781 salicylate synthetase [EC:5.4.4.2 4.2.99.21]	284	257	287	329	84	100	94	82	645	713	627	772
K04782 isochorismate pyruvate lyase [EC:4.2.99.21]	540	528	501	490	286	267	260	295	177	137	153	159
K04783 yersiniabactin salicyl-AMP ligase [EC:6.3.2.-]	5668	5505	5691	5563	3100	3069	2981	2944	3076	2930	2845	2787
K04784 yersiniabactin nonribosomal peptide synthetase	3308	3413	3295	3564	1120	1147	1135	1079	5606	5762	5451	5755
K04785 yersiniabactin synthetase, thiazolinyl reductase component	452	463	391	429	116	135	106	108	938	904	967	931
K04786 yersiniabactin nonribosomal peptide/polyketide synthase	3662	3508	3359	3641	969	1039	967	867	7355	7636	7382	7909
K04787 mycobactin salicyl-AMP ligase [EC:6.3.2.-]	0	0	0	0	0	0	0	1	1	0	0	0

K04788 mycobactin phenyloxazoline synthetase	56	51	54	40	18	23	45	36	10	11	7	13
K04792 mycobactin peptide synthetase MbtF	0	0	0	0	0	0	0	0	0	0	0	1
K12238 pyochelin biosynthesis protein PchD	2623	2311	2267	2387	1293	1242	1170	1312	802	711	660	743
K12239 dihydroaeruginoic acid synthetase	2557	2576	2172	2273	1306	1227	1234	1445	850	675	692	790
K12240 pyochelin synthetase	7108	6939	6050	6098	3437	3387	3113	3701	2465	2153	2021	2271
K12241 pyochelin biosynthetic protein PchG	1668	1568	1308	1356	791	762	724	912	513	522	443	419
ko01055 Biosynthesis of vancomycin group antibiotics	4263	4315	4174	4267	4526	4742	4663	4518	3969	4130	3934	3891
K16428 nonribosomal peptide synthetase CepA	0	0	0	0	1	1	0	0	1	0	0	0
Biosynthesis of other secondary metabolites	190903	190852	192732	191796	233503	232484	235180	231851	217484	215139	217005	214577
ko00940 Phenylpropanoid biosynthesis	32196	33340	34471	33540	71230	70199	72603	70003	65143	64596	66190	63984
K12355 coniferyl-aldehyde dehydrogenase [EC:1.2.1.68]	0	0	0	0	0	1	0	1	0	0	0	0
K20039 phenacrylate decarboxylase [EC:4.1.1.102]	1	0	0	0	0	0	0	2	0	0	0	0
ko00944 Flavone and flavonol biosynthesis	2965	2979	3153	3092	7622	7568	7800	7477	7097	6746	7115	6902
ko00901 Indole alkaloid biosynthesis	0	1	0	0	0	0	0	0	0	0	0	0
ko00950 Isoquinoline alkaloid biosynthesis	10701	10860	10839	10806	10253	10109	10199	10350	10541	10532	10374	10532
ko00960 Tropane, piperidine and pyridine alkaloid biosynthesis	22018	22019	22162	22222	20537	20411	20343	20144	21829	21745	21503	21807
K00808 homospermidine synthase [EC:2.5.1.44]	1853	1903	2132	2044	1112	1128	1049	1047	632	622	648	573
K08081 tropinone reductase I [EC:1.1.1.206]	1	0	0	0	0	0	0	0	0	0	0	0
K13607 cinnamoyl-CoA:phenyllactate CoA-transferase [EC:2.8.3.17]	9	5	5	9	1	5	0	0	4	9	5	7
K19064 lysine 6-dehydrogenase [EC:1.4.1.18]	0	0	0	4	0	0	0	1	4	3	2	4
ko00232 Caffeine metabolism	0	2	0	0	0	4	0	0	0	0	0	0
ko00965 Betalain biosynthesis	1718	1703	1494	1617	930	975	830	922	1039	1035	1005	963
K15777 4,5-DOPA dioxygenase extradiol [EC:1.13.11.-]	1718	1702	1494	1617	930	975	830	922	1039	1035	1005	963
ko00966 Glucosinolate biosynthesis	4521	4354	4339	4552	4914	5049	4994	4747	4882	4859	4710	4855
ko00311 Penicillin and cephalosporin biosynthesis	8866	8634	8627	8540	7640	7633	7507	7599	5580	5068	5180	5221
K01060 cephalosporin-C deacetylase [EC:3.1.1.41]	124	150	136	154	88	95	70	91	66	62	64	63
K01434 penicillin amidase [EC:3.5.1.11]	7230	6914	6660	6777	3698	3620	3556	3726	2261	1970	1884	2099
K17836 beta-lactamase class A [EC:3.5.2.6]	1501	1561	1824	1604	3845	3906	3872	3772	3243	3030	3224	3054
K19200 isopenicillin-N N-acyltransferase like protein	11	9	7	5	9	12	9	10	10	6	8	5
ko00332 Carbapenem biosynthesis	6736	6670	6770	6805	8230	7978	8443	8404	7958	7776	8258	8066
ko00261 Monobactam biosynthesis	30837	30802	31277	31199	35977	36067	36221	35656	34760	34734	34737	34241
K05375 MbtH protein	314	276	266	317	152	125	131	157	111	96	80	106
ko00521 Streptomycin biosynthesis	33159	33370	33617	33510	39818	40498	40907	40356	38527	38516	38430	38505
ko00524 Neomycin, kanamycin and gentamicin biosynthesis	5641	5770	5818	5761	10192	10337	10553	10631	9141	9227	9378	9288
ko00525 Acarbose and validamycin biosynthesis	8600	8672	8635	8545	9951	10141	10334	10028	8904	9020	8913	8892
K19974 2-epi-5-epi-valiolone 7-phosphate 2-epimerase [EC:5.1.3.35]	0	0	0	0	0	0	0	1	0	0	0	0
K20437 validoxylamine A 7'-phosphate phosphatase [EC:3.1.3.101]	0	0	0	0	1	0	0	0	0	0	3	0
ko00401 Novobiocin biosynthesis	16959	16987	17235	17013	19343	19151	19422	19056	17369	17133	17252	17320
ko00404 Staurosporine biosynthesis	1	0	0	2	0	0	0	0	0	0	0	0
K14266 tryptophan 7-halogenase [EC:1.14.19.9]	1	0	0	2	0	0	0	0	0	0	0	0
ko00405 Phenazine biosynthesis	23666	23152	22957	22726	16136	15901	15484	16081	11436	10981	10783	10955
K06998 trans-2,3-dihydro-3-hydroxyanthranilate isomerase [EC:5.3.3.17]	208	153	160	144	257	276	265	225	4	7	5	3
K13063 2-amino-4-deoxychorismate synthase [EC:2.6.1.86]	2265	2135	2010	2043	1114	1129	1062	1184	653	703	650	646
K17940 2-heptyl-3-hydroxy-4(1H)-quinolone synthase [EC:1.14.13.182]	566	565	591	550	336	273	287	293	167	157	180	167
K18000 anthranilate-CoA ligase [EC:6.2.1.32]	2371	2396	2409	2415	1474	1327	1240	1360	770	690	666	807
K18001 2-heptyl-4(1H)-quinolone synthase subunit PqsB [EC:2.3.1.230]	1422	1379	1447	1388	807	804	765	850	463	410	445	413
K18002 2-heptyl-4(1H)-quinolone synthase subunit PqsC [EC:2.3.1.230]	1481	1476	1555	1402	864	810	828	821	478	463	453	426
K18003 anthraniloyl-CoA anthraniloyltransferase [EC:2.3.1.262]	1711	1526	1560	1526	918	868	870	885	515	514	449	474
K20257 2-aminobenzoylacetyl-CoA thioesterase [EC:3.1.2.32]	272	265	248	260	136	135	149	166	79	89	123	82
K20260 phenazine biosynthesis protein	1399	1332	1291	1318	722	720	624	819	407	464	384	441
K20261 trans-2,3-dihydro-3-hydroxyanthranilic acid synthase [EC:3.3.2.15]	812	892	774	807	448	423	459	434	297	243	217	277
K20262 dihydrophenazinedicarboxylate synthase [EC:1.10.3.16]	542	526	459	447	256	242	243	256	153	136	115	134
K20940 5-methylphenazine-1-carboxylate 1-monooxygenase [EC:1.14.13.218]	1944	1773	1695	1788	1012	988	948	1026	591	551	573	536
K21103 phenazine-1-carboxylate N-methyltransferase [EC:2.1.1.327]	3	5	2	5	2	3	3	3	2	2	0	0
ko00333 Prodigiosin biosynthesis	18169	17674	17798	17780	14987	14646	14658	14869	13904	13766	13798	13809
ko00254 Aflatoxin biosynthesis	0	1	0	0	0	0	0	0	0	0	0	0
ko00998 Biosynthesis of secondary metabolites - other antibiotics	1602	1539	1528	1680	1581	1563	1563	1682	1555	1506	1587	1594
K19548 dihydroanticapsin dehydrogenase [EC:1.1.1.385]	0	0	0	1	0	0	0	0	0	1	0	1
K21721 N-[(2S)-2-amino-2-carboxyethyl]-L-glutamate dehydrogenase [EC:1.5.1.51]	5	6	10	6	57	62	52	54	13	10	15	23
ko00999 Biosynthesis of secondary metabolites - unclassified	25	51	51	37	97	120	81	91	40	35	59	75
Xenobiotics biodegradation and metabolism	199531	197852	190839	196157	142449	142050	138282	143945	152281	150905	148117	152118
ko00362 Benzoate degradation	70270	69863	66855	68977	44700	44506	43305	45274	55403	54819	53560	56141
K00448 protocatechuate 3,4-dioxygenase, alpha subunit [EC:1.13.11.3]	737	762	660	790	327	316	308	372	628	607	537	626
K00449 protocatechuate 3,4-dioxygenase, beta subunit [EC:1.13.11.3]	721	633	618	649	313	290	285	278	653	651	622	668
K00481 p-hydroxybenzoate 3-monooxygenase [EC:1.14.13.2]	919	937	924	986	413	399	411	394	1075	1205	1125	1166
K01031 3-oxoadipate CoA-transferase, alpha subunit [EC:2.8.3.6]	227	233	224	237	62	72	78	76	483	467	480	498
K01032 3-oxoadipate CoA-transferase, beta subunit [EC:2.8.3.6]	232	203	203	236	52	54	50	62	490	484	465	471
K01055 3-oxoadipate enol-lactonase [EC:3.1.1.24]	2533	2579	2441	2533	1371	1420	1303	1544	1404	1278	1265	1360
K01607 4-carboxymuconolactone decarboxylase [EC:4.1.1.44]	2843	2945	2818	2956	3284	3509	3411	3366	4000	3821	3975	3897
K01821 4-oxalocrotonate tautomerase [EC:5.3.2.6]	286	247	234	251	150	177	206	194	206	221	217	220
K01856 muconate cycloisomerase [EC:5.5.1.1]	1916	1833	1716	1885	888	867	798	906	1370	1326	1303	1316
K01857 3-carboxy-cis,cis-muconate cycloisomerase [EC:5.5.1.2]	2839	2790	2531	2662	1457	1398	1324	1481	1532	1502	1398	1626
K03381 catechol 1,2-dioxygenase [EC:1.13.11.1]	1027	979	895	903	462	441	418	451	811	823	753	885
K03464 muconolactone D-isomerase [EC:5.3.3.4]	530	514	524	509	248	269	214	282	383	382	338	377
K04108 4-hydroxybenzoyl-CoA reductase subunit alpha [EC:1.3.7.9]	4	6	4	5	1	3	1	1	4	1	2	1
K04116 cyclohexanecarboxylate-CoA ligase [EC:6.2.1.-]	4	0	2	1	0	0	1	0	2	8	4	0
K05549 benzoate/toluate 1,2-dioxygenase subunit alpha [EC:1.14.12.10 1.14.12.-]	2308	2308	2172	2153	1056	1027	953	1069	1568	1568	1504	1603
K05550 benzoate/toluate 1,2-dioxygenase subunit beta [EC:1.14.12.10 1.14.12.-]	924	904	755	851	426	421	395	474	546	519	569	560
K05783 dihydroxycyclohexadiene carboxylate dehydrogenase [EC:1.3.1.25 1.3.1.-]	1165	1103	1003	1077	439	486	480	532	758	705	684	804
K05784 benzoate/toluate 1,2-dioxygenase reductase component [EC:1.18.1.-]	1819	1665	1768	1794	920	866	763	911	1109	1109	1124	1222
K07104 catechol 2,3-dioxygenase [EC:1.13.11.2]	98	104	107	110	21	23	21	18	64	131	102	116
K07535 2-hydroxycyclohexanecarboxyl-CoA dehydrogenase [EC:1.1.1.-]	1	2	2	1	0	0	0	0	9	1	0	2
K07536 2-ketocyclohexanecarboxyl-CoA hydrolase [EC:3.1.2.-]	0	0	0	0	0	0	0	0	0	1	0	0
K07823 3-oxoadipyl-CoA thiolase [EC:2.3.1.174]	2241	2106	2116	2080	1212	1157	1123	1234	728	638	661	731
K14333 2,3-dihydroxybenzoate decarboxylase [EC:4.1.1.46]	401	428	378	390	112	131	104	105	879	837	755	875
K16173 glutaryl-CoA dehydrogenase (non-decarboxylating) [EC:1.3.99.32]	1	0	0	0	0	0	0	0	0	0	0	0
K16514 4-oxalomesaconate tautomerase [EC:5.3.2.8]	464	485	481	501	127	142	141	143	852	918	870	929
K16515 4-oxalomesaconate hydratase [EC:4.2.1.83]	317	350	297	342	95	68	87	96	609	666	664	689
K18365 4-hydroxy-2-oxovalerate/4-hydroxy-2-oxohexanoate aldolase [EC:4.1.3.39 4.1.3.43]	0	0	0	0	0	0	0	0	1	0	0	0
K19066 cyclohex-1-ene-1-carbonyl-CoA dehydrogenase [EC:1.3.8.10]	0	0	0	0	0	0	0	0	1	3	0	1
K19067 cyclohexane-1-carbonyl-CoA dehydrogenase [EC:1.3.8.11]	0	0	2	2	0	0	2	0	0	1	0	1
ko00627 Aminobenzoate degradation	28705	28147	25968	27495	15633	15452	14431	15936	15093	14652	14286	15017
K00141 benzaldehyde dehydrogenase (NAD) [EC:1.2.1.28]	0	0	2	1	0	0	0	0	1	0	3	0
K01101 4-nitrophenyl phosphatase [EC:3.1.3.41]	48	62	44	49	163	144	130	153	48	72	71	61
K01576 benzoylformate decarboxylase [EC:4.1.1.7]	2777	2649	2356	2493	1388	1423	1230	1496	901	759	726	782
K01612 vanillate/4-hydroxybenzoate decarboxylase subunit C [EC:4.1.1.- 4.1.1.61]	721	697	620	755	537	578	458	516	773	856	812	837
K01781 mandelate racemase [EC:5.1.2.2]	0	0	2	2	2	0	0	0	2	4	0	1
K03862 vanillate monooxygenase [EC:1.14.13.82]	1886	1818	1655	1723	1021	995	953	1066	580	531	468	595
K03863 vanillate monooxygenase ferredoxin subunit	1631	1612	1389	1511	931	804	810	909	525	418	442	439
K04099 gallate dioxygenase [EC:1.13.11.57]	542	613	544	617	176	176	140	136	1130	1066	1034	1091
K05599 anthranilate 1,2-dioxygenase (deaminating, decarboxylating) large subunit [EC:1.14.12.1]	1917	1855	1782	2006	1033	994	872	1032	659	654	533	614
K05600 anthranilate 1,2-dioxygenase (deaminating, decarboxylating) small subunit [EC:1.14.12.1]	711	688	649	648	359	306	345	333	217	203	206	160
K08295 2-aminobenzoate-CoA ligase [EC:6.2.1.32]	0	2	0	0	0	0	0	3	2	3	2	4
K09461 anthraniloyl-CoA monooxygenase [EC:1.14.13.40]	3	3	2	2	1	0	2	1	5	13	5	5
K11311 anthranilate 1,2-dioxygenase reductase component [EC:1.18.1.-]	1479	1341	1249	1296	717	652	634	752	391	379	367	419
K14581 naphthalene 1,2-dioxygenase ferredoxin reductase component [EC:1.18.1.7]	2	0	0	1	2	0	0	0	0	2	3	4
K15054 (S)-mandelate dehydrogenase [EC:1.1.99.31]	219	226	220	206	299	370	293	315	0	0	2	1
K15066 vanillate/3-O-methylgallate O-demethylase [EC:2.1.1.341]	4	11	5	7	0	0	0	0	0	0	1	0
K20712 3-(hydroxyamino)phenol mutase [EC:5.4.4.3]	2	0	0	0	0	0	0	0	0	0	0	0
K21759 vanillate/4-hydroxybenzoate decarboxylase subunit D [EC:4.1.1.- 4.1.1.61]	131	174	132	165	82	120	114	107	217	195	198	206
K21802 vanillin dehydrogenase [EC:1.2.1.67]	10	9	7	15	0	2	2	1	2	2	3	4
K22958 gallate decarboxylase subunit C [EC:4.1.1.59]	1567	1502	1451	1528	790	784	745	755	1880	1888	1915	2041
K22959 gallate decarboxylase subunit B	46	42	50	48	8	5	7	6	39	37	25	34
K22960 gallate decarboxylase subunit D	0	0	0	1	3	4	9	1	5	11	10	4
ko00364 Fluorobenzoate degradation	11923	11475	10761	11216	5574	5506	5066	5738	8307	8265	7839	8534
K01061 carboxymethylenebutenolidase [EC:3.1.1.45]	2764	2683	2452	2553	1383	1398	1259	1395	2144	2215	1902	2144
ko00625 Chloroalkane and chloroalkene degradation	23344	23348	22868	23078	13900	13717	13373	14123	13057	13185	12792	12980
K01560 2-haloacid dehalogenase [EC:3.8.1.2]	1018	1018	910	991	470	427	459	529	448	416	417	474
K01561 haloacetate dehalogenase [EC:3.8.1.3]	1186	1173	1149	1175	609	610	575	648	473	373	349	394
ko00361 Chlorocyclohexane and chlorobenzene degradation	8845	8646	8012	8465	4252	4247	3967	4404	5607	5527	5098	5588
K21607 tetrachlorobenzoquinone reductase [EC:1.1.1.404]	836	856	783	848	419	481	437	457	297	243	272	259

ko00623 Toluene degradation	6114	5879	5469	5807	2841	2841	2570	2893	5207	5255	4797	5189
K07540 benzylsuccinate synthase [EC:4.1.99.11]	0	5	2	0	3	0	0	0	0	0	3	0
K07546 E-phenylitaconyl-CoA hydratase [EC:4.2.1.-]	0	0	0	0	0	0	0	1	0	0	0	0
K20199 4-hydroxybenzaldehyde dehydrogenase (NADP+) [EC:1.2.1.96]	1	0	0	0	1	0	0	1	0	0	0	0
ko00622 Xylene degradation	9871	9674	9163	9556	4523	4632	4263	4765	7636	7565	7427	7904
K10619 p-cumate 2,3-dioxygenase subunit alpha [EC:1.14.12.25]	1712	1828	1572	1686	916	891	805	935	538	464	460	527
ko00633 Nitrotoluene degradation	6332	6311	6128	6155	4941	5183	5347	5250	4905	5077	4827	5081
K06281 hydrogenase large subunit [EC:1.12.99.6]	537	526	562	504	733	803	760	739	400	415	383	415
K06282 hydrogenase small subunit [EC:1.12.99.6]	357	315	329	370	491	441	472	475	245	255	265	314
K10678 nitroreductase [EC:1.-.-.-]	464	467	415	442	267	309	305	247	491	580	574	608
K10679 nitroreductase / dihydropteridine reductase [EC:1.-.-.- 1.5.1.34]	441	456	399	425	353	399	366	352	613	669	593	582
K10680 N-ethylmaleimide reductase [EC:1.-.-.-]	3703	3635	3487	3532	2135	2195	2304	2345	1508	1370	1272	1375
ko00642 Ethylbenzene degradation	6773	6698	6402	6637	4033	3892	3923	3925	3815	3726	3661	3843
ko00643 Styrene degradation	18851	18860	17262	18375	9778	9520	9073	10010	9489	9345	9140	9558
K01502 aliphatic nitrilase [EC:3.5.5.7]	0	1	0	0	0	0	0	0	4	6	2	9
ko00791 Atrazine degradation	6469	6237	5767	6209	4067	4011	3908	4196	3783	3807	3628	3940
K03382 hydroxydechloroatrazine ethylaminohydrolase [EC:3.5.4.43]	35	20	22	35	2	5	3	0	19	28	24	20
K03383 cyanuric acid amidohydrolase [EC:3.5.2.15]	3	0	0	0	0	0	0	0	0	0	0	0
K19837 1-carboxybiuret hydrolase [EC:3.5.1.131]	1	0	0	0	0	0	0	0	0	0	0	0
K23359 biuret amidohydrolase [EC:3.5.1.84]	9	5	14	12	1	3	2	2	5	6	2	1
ko00930 Caprolactam degradation	17149	16501	16484	16659	9697	9599	8993	9768	8999	8844	8740	9199
K06446 acyl-CoA dehydrogenase [EC:1.3.99.-]	1797	1620	1614	1787	889	907	798	977	573	569	589	532
K17754 cyclopentanol dehydrogenase [EC:1.1.1.163]	0	0	0	0	0	0	0	0	0	1	0	0
K19962 6-oxohexanoate dehydrogenase [EC:1.2.1.63]	1	0	0	0	0	1	0	0	0	0	0	0
ko00363 Bisphenol degradation	861	817	768	864	467	483	480	503	290	239	272	286
K05913 2,4'-dihydroxyacetophenone dioxygenase [EC:1.13.11.41]	861	817	768	864	467	483	480	503	290	239	272	286
ko00621 Dioxin degradation	1439	1383	1382	1420	641	783	751	687	2173	2181	2151	2229
K00480 salicylate hydroxylase [EC:1.14.13.1]	1	0	0	1	0	0	0	0	2	3	3	1
ko00626 Naphthalene degradation	10423	10467	10613	10543	6135	6295	6069	6079	8427	8877	8655	8530
ko00624 Polycyclic aromatic hydrocarbon degradation	1461	1395	1278	1441	642	606	593	650	1283	1263	1165	1299
ko00365 Furfural degradation	32	44	36	20	1	2	3	4	11	9	7	9
K16873 5-(hydroxymethyl)furfural/furfural oxidase [EC:1.1.3.47 1.1.3.-]	0	0	0	0	0	0	0	2	0	0	0	0
K16874 2,5-furandicarboxylate decarboxylase 1	30	44	35	20	1	2	3	2	10	9	7	9
K16875 2,5-furandicarboxylate decarboxylase 2	1	0	1	0	0	0	0	0	1	0	0	0
K16880 2-oxoglutaroyl-CoA hydrolase	1	0	0	0	0	0	0	0	0	0	0	0
ko00984 Steroid degradation	8	2	0	7	0	4	1	2	5	1	4	3
K03333 cholesterol oxidase [EC:1.1.3.6]	0	0	0	2	0	2	0	0	2	0	1	1
K05898 3-oxosteroid 1-dehydrogenase [EC:1.3.99.4]	3	1	0	1	0	0	1	2	2	1	2	1
K16048 3-hydroxy-9,10-secoandrosta-1,3,5(10)-triene-9,17-dione monooxygenase reductase component [EC:1.5.1.-]	1	1	0	0	0	1	0	0	0	0	0	0
K16050 4,5:9,10-diseco-3-hydroxy-5,9,17-trioxoandrosta-1(10),2-diene-4-oate hydrolase [EC:3.7.1.17]	2	0	0	3	0	0	0	0	0	0	0	0
K18687 HIP---CoA ligase [EC:6.2.1.41]	1	0	0	0	0	1	0	0	0	0	0	0
ko00980 Metabolism of xenobiotics by cytochrome P450	13914	13780	13393	13756	8198	8210	7931	8189	9759	9639	9396	9631
K15303 aflatoxin B1 aldehyde reductase	55	56	49	40	4	8	3	7	97	117	97	103
ko00982 Drug metabolism - cytochrome P450	16397	16383	15952	16301	9639	9610	9235	9726	10520	10392	10082	10270
ko00983 Drug metabolism - other enzymes	42944	42575	43326	43204	48183	48290	47711	47793	48778	48281	48403	47848
K00569 thiopurine S-methyltransferase [EC:2.1.1.67]	946	930	910	899	551	523	438	477	310	255	258	286
K01044 carboxylesterase 1 [EC:3.1.1.1]	0	0	1	0	0	0	0	0	0	1	0	0
K03927 carboxylesterase 2 [EC:3.1.1.1 3.1.1.84 3.1.1.56]	0	0	0	0	0	0	0	0	1	0	0	0
Not included in regular maps	3286	3287	3218	3416	1938	2038	1810	1973	1851	1965	1844	1886
ko09113 Global maps only	3286	3287	3218	3416	1938	2038	1810	1973	1851	1965	1844	1886
K00528 ferredoxin/flavodoxin---NADP+ reductase [EC:1.18.1.2 1.19.1.1]	3105	3135	3077	3251	1703	1779	1580	1759	1851	1965	1844	1883
K08088 fimbrial protein FimY	181	152	141	165	235	259	230	214	0	0	0	3
Genetic Information Processing	688494	689615	696901	695431	729254	736705	735480	727754	712116	714939	716173	713046
Transcription	27799	28114	28971	28527	34261	35071	34594	33772	33945	34478	34784	33403
ko03020 RNA polymerase	27778	28095	28948	28514	34250	35071	34590	33769	33902	34425	34744	33346
K02999 DNA-directed RNA polymerase I subunit RPA1 [EC:2.7.7.6]	0	0	0	0	0	0	0	0	2	0	0	0
K03002 DNA-directed RNA polymerase I subunit RPA2 [EC:2.7.7.6]	0	2	0	0	0	0	0	0	0	0	0	0
K03011 DNA-directed RNA polymerase II subunit RPB3	0	2	0	0	0	0	0	0	0	0	0	0
K03040 DNA-directed RNA polymerase subunit alpha [EC:2.7.7.6]	3331	3411	3419	3372	3946	3945	3957	3979	3914	3962	3948	3902
K03041 DNA-directed RNA polymerase subunit A' [EC:2.7.7.6]	2	0	0	3	3	0	3	3	18	31	13	32
K03042 DNA-directed RNA polymerase subunit A [EC:2.7.7.6]	2	0	8	0	2	0	0	1	12	16	4	7
K03043 DNA-directed RNA polymerase subunit beta [EC:2.7.7.6]	10872	11269	11552	11363	14140	14675	14483	13922	13783	14035	14231	13540
K03044 DNA-directed RNA polymerase subunit B' [EC:2.7.7.6]	2	0	2	1	0	0	2	0	4	12	8	15
K03045 DNA-directed RNA polymerase subunit B [EC:2.7.7.6]	0	1	1	1	0	1	0	2	2	4	8	6
K03046 DNA-directed RNA polymerase subunit beta' [EC:2.7.7.6]	12740	12599	13223	12943	15703	15944	15695	15415	15625	15800	15959	15315
K03047 DNA-directed RNA polymerase subunit D [EC:2.7.7.6]	1	2	0	0	0	1	1	0	1	5	2	6
K03048 DNA-directed RNA polymerase subunit delta	20	27	11	23	4	5	5	7	24	13	20	27
K03049 DNA-directed RNA polymerase subunit E' [EC:2.7.7.6]	0	0	0	1	0	0	0	0	0	7	0	3
K03050 DNA-directed RNA polymerase subunit E [EC:2.7.7.6]	0	0	0	0	0	0	0	1	1	0	2	1
K03051 DNA-directed RNA polymerase subunit F [EC:2.7.7.6]	0	0	0	0	0	0	0	1	0	1	3	0
K03053 DNA-directed RNA polymerase subunit H [EC:2.7.7.6]	0	0	0	0	2	0	2	0	5	4	0	1
K03055 DNA-directed RNA polymerase subunit K [EC:2.7.7.6]	2	0	0	0	0	0	0	0	3	0	1	1
K03056 DNA-directed RNA polymerase subunit L [EC:2.7.7.6]	0	0	0	1	0	0	0	0	0	2	2	1
K03058 DNA-directed RNA polymerase subunit N [EC:2.7.7.6]	0	0	0	0	2	0	0	0	0	0	0	1
K03059 DNA-directed RNA polymerase subunit P [EC:2.7.7.6]	0	0	0	0	0	0	0	0	0	1	1	1
K03060 DNA-directed RNA polymerase subunit omega [EC:2.7.7.6]	805	781	730	805	447	499	442	437	508	531	538	482
K13797 DNA-directed RNA polymerase subunit beta-beta' [EC:2.7.7.6]	1	1	0	0	1	0	0	1	0	0	1	0
K13798 DNA-directed RNA polymerase subunit B [EC:2.7.7.6]	0	0	2	1	0	1	0	0	0	1	3	5
ko03022 Basal transcription factors	3	2	4	1	11	0	4	3	14	24	10	16
K03120 transcription initiation factor TFIID TATA-box-binding protein	0	2	0	0	2	0	0	0	3	4	3	3
K03124 transcription initiation factor TFIIB	0	0	2	1	3	0	0	0	5	7	3	6
K03135 transcription initiation factor TFIID subunit 11	0	0	0	0	1	0	0	0	0	0	0	0
K03136 transcription initiation factor TFIIE subunit alpha	0	0	2	0	3	0	0	0	3	4	1	0
K10844 DNA excision repair protein ERCC-2 [EC:3.6.4.12]	3	0	0	0	2	0	4	3	3	9	3	7
ko03040 Spliceosome	18	17	19	12	0	0	0	0	29	29	30	41
K03283 heat shock 70kDa protein 1/2/6/8	0	0	1	0	0	0	0	0	0	0	0	1
K12733 peptidyl-prolyl cis-trans isomerase-like 1 [EC:5.2.1.8]	18	13	17	12	0	0	0	0	29	27	30	39
K12823 ATP-dependent RNA helicase DDX5/DBP2 [EC:3.6.4.13]	0	1	1	0	0	0	0	0	0	2	0	1
K12827 splicing factor 3A subunit 3	0	1	0	0	0	0	0	0	0	0	0	0
K12856 pre-mRNA-processing factor 8	0	2	0	0	0	0	0	0	0	0	0	0
Translation	231419	233704	237905	236785	242947	245463	245107	240471	239930	242047	241466	242016
ko03010 Ribosome	78384	79586	82698	81135	88612	88928	90151	86988	84998	85396	85577	85767
K02863 large subunit ribosomal protein L1	2408	2351	2592	2524	2685	2884	2814	2668	2571	2730	2743	2735
K02864 large subunit ribosomal protein L10	1440	1481	1633	1602	1914	2024	1966	1858	1801	1851	1798	1729
K02865 large subunit ribosomal protein L10Ae	0	0	0	0	0	0	0	0	0	2	0	0
K02866 large subunit ribosomal protein L10e	0	0	0	0	0	0	0	0	2	6	3	3
K02867 large subunit ribosomal protein L11	1591	1667	1685	1674	1764	1771	1747	1756	1766	1760	1759	1733
K02869 large subunit ribosomal protein L12	0	0	0	0	0	0	0	1	0	0	0	1
K02871 large subunit ribosomal protein L13	1662	1700	1614	1696	1833	1844	1712	1764	1728	1808	1830	1796
K02874 large subunit ribosomal protein L14	1469	1320	1396	1436	1491	1581	1519	1445	1482	1457	1404	1498
K02875 large subunit ribosomal protein L14e	0	0	0	0	0	0	0	0	2	1	2	0
K02876 large subunit ribosomal protein L15	1576	1651	1688	1715	1793	1799	1953	1904	1737	1842	1831	1831
K02877 large subunit ribosomal protein L15e	0	0	0	1	0	1	0	0	0	6	2	7
K02878 large subunit ribosomal protein L16	1648	1558	1642	1643	1743	1865	1762	1839	1733	1706	1843	1807
K02879 large subunit ribosomal protein L17	1628	1563	1778	1660	1737	1696	1762	1684	1631	1699	1630	1751
K02881 large subunit ribosomal protein L18	1263	1294	1374	1288	1426	1545	1530	1405	1379	1348	1348	1351
K02883 large subunit ribosomal protein L18e	1	0	2	1	0	1	0	2	0	3	0	4
K02884 large subunit ribosomal protein L19	1192	1116	1279	1160	1434	1347	1356	1299	1346	1282	1262	1338
K02885 large subunit ribosomal protein L19e	0	0	2	0	3	0	0	3	4	4	0	3
K02886 large subunit ribosomal protein L2	2448	2431	2632	2452	3071	2911	3201	3021	3097	3168	3032	3127
K02887 large subunit ribosomal protein L20	1118	1197	1213	1168	1210	1182	1240	1177	1136	1193	1261	1170
K02888 large subunit ribosomal protein L21	1165	1264	1267	1222	1321	1258	1238	1174	1319	1301	1196	1257
K02889 large subunit ribosomal protein L21e	3	0	0	0	0	0	0	2	0	3	2	1
K02890 large subunit ribosomal protein L22	1298	1351	1419	1459	1500	1558	1598	1465	1512	1601	1475	1395
K02892 large subunit ribosomal protein L23	1159	1096	1155	1156	1172	1204	1229	1129	1205	1194	1273	1241
K02895 large subunit ribosomal protein L24	1186	1113	1141	1166	1208	1300	1280	1233	1192	1251	1198	1132
K02896 large subunit ribosomal protein L24e	0	0	0	0	0	0	0	1	0	0	0	4
K02897 large subunit ribosomal protein L25	1730	1746	1690	1746	2019	2068	1941	1977	1629	1638	1746	1725
K02899 large subunit ribosomal protein L27	1075	1123	1068	1099	1078	1112	1105	1014	1057	1125	1033	1086
K02902 large subunit ribosomal protein L28	807	845	802	790	950	911	991	840	834	797	827	827
K02904 large subunit ribosomal protein L29	719	730	798	711	780	784	833	813	740	737	753	774

K02906 large subunit ribosomal protein L3	2422	2528	2666	2514	2605	2682	2652	2572	2428	2222	2407	2424
K02907 large subunit ribosomal protein L30	720	677	723	687	723	736	728	763	704	736	693	751
K02908 large subunit ribosomal protein L30e	0	0	0	0	0	0	0	1	2	0	0	5
K02909 large subunit ribosomal protein L31	1479	1511	1535	1459	1428	1386	1325	1296	1325	1355	1276	1246
K02910 large subunit ribosomal protein L31e	0	0	0	0	0	0	0	0	2	0	2	1
K02911 large subunit ribosomal protein L32	615	620	672	679	736	785	777	747	762	662	739	767
K02912 large subunit ribosomal protein L32e	0	2	0	0	0	2	0	3	4	6	0	3
K02913 large subunit ribosomal protein L33	577	649	656	666	636	660	692	608	555	628	653	641
K02914 large subunit ribosomal protein L34	519	553	519	516	535	544	611	611	523	522	551	560
K02915 large subunit ribosomal protein L34e	2	0	0	0	0	0	0	0	2	0	0	1
K02916 large subunit ribosomal protein L35	561	629	650	576	641	668	681	653	561	639	627	639
K02919 large subunit ribosomal protein L36	876	781	783	888	689	704	714	680	649	654	690	696
K02921 large subunit ribosomal protein L37Ae	0	0	0	0	0	0	0	0	0	2	1	4
K02922 large subunit ribosomal protein L37e	1	0	0	1	0	0	0	0	0	0	0	0
K02924 large subunit ribosomal protein L39e	0	0	0	0	0	0	0	0	0	0	1	1
K02925 large subunit ribosomal protein L3e	0	0	2	0	0	0	0	0	0	0	0	0
K02926 large subunit ribosomal protein L4	1580	1777	1733	1774	2221	2155	2306	2168	2291	2242	2276	2211
K02927 large subunit ribosomal protein L40e	0	0	0	1	0	0	0	0	0	0	0	0
K02929 large subunit ribosomal protein L44e	1	0	0	0	0	1	0	0	0	0	0	0
K02930 large subunit ribosomal protein L4e	0	2	0	0	1	2	0	0	4	13	2	5
K02931 large subunit ribosomal protein L5	2074	2130	2145	2160	2287	2503	2277	2328	2283	2398	2251	2311
K02933 large subunit ribosomal protein L6	1988	2022	2134	2056	2398	2359	2380	2238	2135	2095	2264	2030
K02935 large subunit ribosomal protein L7/L12	1405	1452	1385	1414	1499	1579	1484	1286	1447	1424	1454	1451
K02936 large subunit ribosomal protein L7Ae	0	0	0	0	0	0	0	0	1	8	1	2
K02939 large subunit ribosomal protein L9	1565	1528	1625	1664	1731	1765	1842	1787	1639	1731	1682	1704
K02942 large subunit ribosomal protein LP1	0	1	0	0	0	0	0	0	0	0	0	0
K02944 large subunit ribosomal protein LX	1	0	0	1	0	0	0	0	0	0	0	4
K02945 small subunit ribosomal protein S1	5297	5553	5571	5476	6499	6100	6274	6259	6058	5901	6041	5817
K02946 small subunit ribosomal protein S10	1122	1208	1283	1176	1339	1210	1344	1192	1259	1272	1334	1320
K02948 small subunit ribosomal protein S11	1380	1422	1511	1437	1624	1650	1636	1590	1580	1585	1512	1511
K02950 small subunit ribosomal protein S12	1453	1585	1598	1651	1590	1616	1545	1608	1565	1594	1694	1688
K02952 small subunit ribosomal protein S13	1414	1417	1475	1412	1529	1551	1600	1449	1437	1450	1426	1471
K02954 small subunit ribosomal protein S14	1149	1177	1261	1209	1234	1272	1236	1221	1210	1203	1182	1189
K02956 small subunit ribosomal protein S15	888	988	981	1024	1127	1017	1091	1126	1045	1049	997	1020
K02957 small subunit ribosomal protein S15Ae	0	0	0	0	0	0	0	0	0	0	0	1
K02958 small subunit ribosomal protein S15e	0	0	0	0	1	0	0	0	0	0	0	0
K02959 small subunit ribosomal protein S16	985	973	1145	996	1233	1259	1265	1267	1088	973	1166	1189
K02961 small subunit ribosomal protein S17	889	887	1090	982	1083	1119	1151	1021	1037	1097	1045	1062
K02962 small subunit ribosomal protein S17e	0	2	1	0	0	0	0	0	0	2	0	1
K02963 small subunit ribosomal protein S18	947	1005	1069	1049	1040	1078	1139	1115	988	984	997	1097
K02965 small subunit ribosomal protein S19	1047	1114	1156	1082	1191	1151	1111	1113	1245	1217	1150	1202
K02966 small subunit ribosomal protein S19e	1	0	0	3	0	0	0	0	0	5	2	1
K02967 small subunit ribosomal protein S2	2361	2530	2496	2533	2993	2977	3183	2906	2881	2807	2899	2836
K02968 small subunit ribosomal protein S20	861	849	910	796	1084	1025	1104	1033	813	915	949	925
K02970 small subunit ribosomal protein S21	738	727	741	758	842	717	877	856	950	946	860	875
K02974 small subunit ribosomal protein S24e	0	0	0	0	0	0	0	0	0	0	0	1
K02977 small subunit ribosomal protein S27Ae	0	2	0	0	0	0	0	0	0	1	1	1
K02978 small subunit ribosomal protein S27e	0	0	0	0	0	2	0	0	0	0	0	0
K02979 small subunit ribosomal protein S28e	0	0	0	1	0	0	0	0	1	4	2	3
K02982 small subunit ribosomal protein S3	2646	2621	2896	2758	2817	2979	2892	2890	2876	2768	2823	2881
K02984 small subunit ribosomal protein S3Ae	0	0	0	0	0	2	0	0	0	5	7	10
K02986 small subunit ribosomal protein S4	2416	2453	2472	2458	2697	2637	2661	2736	2594	2622	2467	2552
K02987 small subunit ribosomal protein S4e	5	1	0	1	2	0	0	0	11	6	2	2
K02988 small subunit ribosomal protein S5	1862	1807	2030	2022	2011	2083	2176	2003	1938	1903	1886	1967
K02990 small subunit ribosomal protein S6	1218	1223	1222	1202	1237	1233	1292	1298	1183	1195	1283	1276
K02991 small subunit ribosomal protein S6e	2	0	0	0	0	0	0	0	0	4	0	3
K02992 small subunit ribosomal protein S7	1658	1621	1658	1635	1876	1882	1883	1920	1931	1854	1953	1873
K02994 small subunit ribosomal protein S8	1525	1527	1555	1578	1731	1655	1777	1640	1566	1660	1600	1602
K02995 small subunit ribosomal protein S8e	1	0	2	0	0	0	0	0	0	2	0	6
K02996 small subunit ribosomal protein S9	1533	1417	1460	1452	1554	1528	1650	1521	1498	1496	1456	1573
K07590 large subunit ribosomal protein L7A	14	18	17	19	16	8	18	9	24	26	22	29
ko00970 Aminoacyl-tRNA biosynthesis	142167	143005	144509	144727	143932	146276	144845	143047	144532	146340	145552	145840
K01866 tyrosyl-tRNA synthetase [EC:6.1.1.1]	6742	6605	6727	6974	6388	6321	6459	6334	5664	5665	5753	5770
K01867 tryptophanyl-tRNA synthetase [EC:6.1.1.2]	3614	3622	3676	3711	3792	4023	4044	4030	4428	4742	4735	4608
K01868 threonyl-tRNA synthetase [EC:6.1.1.3]	6660	6655	7015	6994	6974	6942	6936	6609	7203	7560	7247	7437
K01869 leucyl-tRNA synthetase [EC:6.1.1.4]	9583	9582	9705	9565	10668	10848	10592	10552	10301	10537	10280	10063
K01870 isoleucyl-tRNA synthetase [EC:6.1.1.5]	10289	10379	10980	10790	11304	11532	11439	11340	10797	10641	10720	10544
K01872 alanyl-tRNA synthetase [EC:6.1.1.7]	9066	9219	9137	9110	9633	9766	9737	9506	9083	9105	9340	9507
K01873 valyl-tRNA synthetase [EC:6.1.1.9]	9179	8897	9059	9315	7199	7385	7188	7018	7897	7971	7694	8077
K01875 seryl-tRNA synthetase [EC:6.1.1.11]	4363	4409	4466	4443	4674	4740	4702	4674	4469	4824	4691	4769
K01876 aspartyl-tRNA synthetase [EC:6.1.1.12]	5602	5653	5659	5922	4487	4708	4525	4545	4629	4751	4821	4732
K01878 glycyl-tRNA synthetase alpha chain [EC:6.1.1.14]	2497	2510	2409	2488	1531	1499	1458	1493	1542	1541	1603	1523
K01879 glycyl-tRNA synthetase beta chain [EC:6.1.1.14]	5273	5390	5062	5278	3211	3377	3074	3328	3368	3520	3394	3562
K01880 glycyl-tRNA synthetase [EC:6.1.1.14]	2009	1953	2101	2054	3947	4053	3875	3831	3549	3258	3509	3352
K01881 prolyl-tRNA synthetase [EC:6.1.1.15]	5371	5276	5451	5584	4901	5020	4916	4819	5372	5524	5216	5467
K01883 cysteinyl-tRNA synthetase [EC:6.1.1.16]	4181	4270	4450	4306	5064	5226	4883	5016	4914	4708	4919	4899
K01886 glutaminyl-tRNA synthetase [EC:6.1.1.18]	5316	5378	5480	5444	5606	5793	5776	5569	5269	5325	5470	5239
K01887 arginyl-tRNA synthetase [EC:6.1.1.19]	6214	6256	6241	6249	7091	7075	6708	6915	6311	6389	6285	6315
K01889 phenylalanyl-tRNA synthetase alpha chain [EC:6.1.1.20]	3043	3223	3267	3119	3450	3369	3418	3400	3250	3406	3288	3315
K01890 phenylalanyl-tRNA synthetase beta chain [EC:6.1.1.20]	7083	6995	7352	7149	7961	7927	7809	7840	7446	7653	7654	7474
K01892 histidyl-tRNA synthetase [EC:6.1.1.21]	4269	4468	4612	4493	4784	4923	4876	4876	4404	4574	4406	4337
K01893 asparaginyl-tRNA synthetase [EC:6.1.1.22]	2827	2850	2953	2865	4053	4108	4264	3926	4302	4435	4302	4279
K02433 aspartyl-tRNA(Asn)/glutamyl-tRNA(Gln) amidotransferase subunit A [EC:6.3.5.6 6.3.5.7]	3038	2913	2942	2974	1487	1489	1511	1571	1644	1777	1588	1823
K02434 aspartyl-tRNA(Asn)/glutamyl-tRNA(Gln) amidotransferase subunit B [EC:6.3.5.6 6.3.5.7]	1873	1908	1765	1871	1007	978	1039	1096	1222	1275	1235	1285
K02435 aspartyl-tRNA(Asn)/glutamyl-tRNA(Gln) amidotransferase subunit C [EC:6.3.5.6 6.3.5.7]	572	584	589	576	335	312	324	307	320	347	330	354
K03330 glutamyl-tRNA(Gln) amidotransferase subunit E [EC:6.3.5.7]	2	2	3	0	0	4	3	2	18	16	19	14
K04566 lysyl-tRNA synthetase, class I [EC:6.1.1.6]	70	55	78	80	17	23	13	19	119	180	138	160
K04567 lysyl-tRNA synthetase, class II [EC:6.1.1.6]	5796	5870	5768	5831	6175	6320	6497	6310	7141	6987	7010	7076
K09482 glutamyl-tRNA(Gln) amidotransferase subunit D [EC:6.3.5.7]	2	0	0	0	0	1	0	0	13	14	13	8
K09698 nondiscriminating glutamyl-tRNA synthetase [EC:6.1.1.24]	171	178	159	173	57	59	57	53	235	263	279	268
K09759 nondiscriminating aspartyl-tRNA synthetase [EC:6.1.1.23]	501	406	439	455	126	109	108	119	607	529	580	606
K11627 pyrrolysyl-tRNA synthetase [EC:6.1.1.26]	0	0	0	0	0	0	0	0	0	0	0	1
K14164 glycyl-tRNA synthetase [EC:6.1.1.14]	0	0	0	0	1	0	0	0	0	0	0	0
ko03013 RNA transport	6412	6540	6301	6264	5983	5863	5904	5942	6316	6304	6250	6427
K00784 ribonuclease Z [EC:3.1.26.11]	1950	1994	1984	1970	2651	2610	2667	2549	3011	2983	2929	3015
K00974 tRNA nucleotidyltransferase (CCA-adding enzyme) [EC:2.7.7.72 3.1.3.- 3.1.4.-]	3378	3396	3247	3271	2040	1991	1921	2029	2262	2277	2269	2303
K03113 translation initiation factor 1	1068	1131	1058	1015	1286	1257	1307	1356	1015	975	1021	1049
K03231 elongation factor 1-alpha	0	3	3	0	2	0	0	0	2	11	3	12
K03236 translation initiation factor 1A	0	0	1	0	0	0	0	0	2	1	3	5
K03237 translation initiation factor 2 subunit 1	0	0	0	1	0	1	2	0	4	1	0	4
K03238 translation initiation factor 2 subunit 2	0	0	0	0	0	2	0	1	3	1	3	3
K03239 translation initiation factor eIF-2B subunit alpha	8	9	5	4	1	0	2	2	2	4	4	5
K03242 translation initiation factor 2 subunit 3	2	0	2	0	1	0	2	0	10	11	7	9
K03243 translation initiation factor 5B	2	2	1	1	2	0	0	2	1	16	3	12
K03251 translation initiation factor 3 subunit D	0	0	0	2	0	0	0	0	0	0	0	0
K03537 ribonuclease P/MRP protein subunit POP5 [EC:3.1.26.5]	0	0	0	0	0	0	0	0	0	4	0	1
K03538 ribonuclease P protein subunit POP4 [EC:3.1.26.5]	0	0	0	0	0	0	0	0	0	1	0	0
K03539 ribonuclease P/MRP protein subunit RPP1 [EC:3.1.26.5]	2	0	0	0	0	0	0	0	0	6	2	1
K03540 ribonuclease P protein subunit RPR2 [EC:3.1.26.5]	2	1	0	0	0	0	0	0	0	3	0	1
K07562 nonsense-mediated mRNA decay protein 3	0	2	0	0	0	1	1	2	4	9	6	6
K07936 GTP-binding nuclear protein Ran	0	0	0	0	0	0	0	1	0	0	0	0
K13126 polyadenylate-binding protein	0	0	0	0	0	0	2	0	0	0	0	1
K14288 exportin-T	0	2	0	0	0	0	0	0	0	0	0	0
K14293 importin subunit beta-1	0	0	0	0	0	0	0	0	0	1	0	0
K14296 nuclear pore complex protein Nup153	0	0	0	0	0	1	0	0	0	0	0	0
ko03015 mRNA surveillance pathway	0	1	5	1	0	2	7	3	5	14	8	15
K03265 peptide chain release factor subunit 1	0	0	1	1	0	0	2	3	5	13	3	9
K06269 serine/threonine-protein phosphatase PP1 catalytic subunit [EC:3.1.3.16]	0	0	0	0	0	2	0	0	0	0	0	1
K06965 protein pelota	0	0	2	0	0	0	3	0	0	1	3	4
K14403 cleavage and polyadenylation specificity factor subunit 3 [EC:3.1.27.-]	0	0	2	0	0	0	0	0	0	0	0	0
K14408 cleavage stimulation factor subunit 3	0	0	0	0	0	0	0	0	0	0	2	0

K15544 RNA polymerase II subunit A C-terminal domain phosphatase SSU72 [EC:3.1.3.16]	0	1	0	0	0	0	0	0	0	0	0	0
ko03008 Ribosome biogenesis in eukaryotes	4458	4574	4392	4658	4420	4395	4203	4494	4083	4013	4087	3976
K03264 translation initiation factor 6	4	2	0	0	0	0	2	0	5	10	5	6
K03685 ribonuclease III [EC:3.1.26.3]	2267	2346	2291	2492	3097	3097	3018	3174	2861	2793	2859	2738
K06943 nucleolar GTP-binding protein	0	2	0	1	2	0	0	0	2	7	1	9
K07178 RIO kinase 1 [EC:2.7.11.1]	1020	1090	964	1024	567	572	522	600	361	339	346	318
K11130 H/ACA ribonucleoprotein complex subunit 3	0	0	0	0	0	1	0	1	0	0	2	1
K11131 H/ACA ribonucleoprotein complex subunit 4 [EC:5.4.99.-]	0	0	0	0	0	0	2	0	0	14	5	9
K13288 oligoribonuclease [EC:3.1.-.-]	1165	1127	1135	1141	747	718	656	712	844	816	854	864
K14536 ribosome assembly protein 1 [EC:3.6.5.-]	0	0	0	0	2	0	0	0	0	0	2	0
K14538 nuclear GTP-binding protein	0	0	2	0	0	0	0	0	0	0	0	0
K14550 U3 small nucleolar RNA-associated protein 10	0	0	0	0	3	0	0	0	0	0	0	0
K14558 periodic tryptophan protein 2	0	0	0	0	0	1	0	0	0	0	0	0
K14561 U3 small nucleolar ribonucleoprotein protein IMP4	0	0	0	0	0	0	0	0	0	4	0	3
K14564 nucleolar protein 56	0	5	0	0	0	2	0	2	4	4	0	4
K14572 midasin	0	0	0	0	0	1	0	0	0	0	0	1
K14574 ribosome maturation protein SDO1	0	0	0	0	0	2	2	2	0	2	5	5
Folding, sorting and degradation	174908	175003	177026	175944	193526	195858	195289	193074	181441	180503	180689	179546
ko03060 Protein export	53866	54160	54942	54304	61801	62256	62358	61735	57576	56944	56998	55828
K03070 preprotein translocase subunit SecA [EC:7.4.2.8]	10636	10483	10919	10637	11748	12054	12206	12189	11170	11026	10733	10698
K03071 preprotein translocase subunit SecB	1175	1157	1179	1120	702	770	702	752	698	794	706	760
K03072 preprotein translocase subunit SecD	4397	4244	4335	4240	2718	2657	2590	2699	2659	2863	2592	2701
K03073 preprotein translocase subunit SecE	1019	1096	1075	1013	1008	1037	1073	929	913	986	980	942
K03074 preprotein translocase subunit SecF	2190	2071	2004	2046	1228	1245	1263	1258	1487	1553	1511	1455
K03075 preprotein translocase subunit SecG	1123	1199	1200	1236	1332	1245	1054	1023	1139	1098	1089	1151
K03076 preprotein translocase subunit SecY	3699	3992	4003	4120	4596	4977	4909	4691	4737	4881	4778	4751
K03100 signal peptidase I [EC:3.4.21.89]	5225	5239	5296	5203	8286	8407	8553	8605	7011	6772	7071	6586
K03101 signal peptidase II [EC:3.4.23.36]	2010	2059	2083	2088	2159	2103	2138	2050	1992	1913	2007	1894
K03105 signal recognition particle subunit SRP19	0	0	0	2	2	0	1	0	0	0	0	4
K03106 signal recognition particle subunit SRP54 [EC:3.6.5.4]	4524	4669	4614	4627	4994	4932	4883	4805	4624	4591	4572	4428
K03107 signal recognition particle subunit SRP68	0	0	2	0	0	0	0	0	0	0	0	0
K03110 fused signal recognition particle receptor	3300	3317	3191	3120	3400	3219	3550	3550	3295	3072	3238	3093
K03116 sec-independent protein translocase protein TatA	693	696	691	670	638	648	615	694	604	582	595	563
K03117 sec-independent protein translocase protein TatB	764	676	724	677	504	557	489	572	548	474	499	490
K03118 sec-independent protein translocase protein TatC	2545	2535	2586	2472	3282	3200	3104	3217	2643	2539	2553	2516
K03210 preprotein translocase subunit YajC	1080	1137	1098	1124	1096	1105	994	1023	1057	1035	1019	1118
K03217 YidC/Oxa1 family membrane protein insertase	5842	5725	5902	6007	6328	6366	6404	6031	5969	5965	5895	5903
K03425 sec-independent protein translocase protein TatE	81	104	97	102	80	81	66	60	131	125	121	141
K09490 heat shock 70kDa protein 5	0	1	0	0	0	0	0	0	0	1	0	0
K12257 SecD/SecF fusion protein	3134	3331	3515	3330	7399	7358	7463	7290	6275	5974	6345	5940
K13280 signal peptidase I [EC:3.4.21.89]	95	107	127	100	72	60	60	76	163	172	215	196
K13301 secretion monitor	334	322	301	370	229	235	241	221	459	528	479	498
K13431 signal recognition particle receptor subunit alpha	0	0	0	0	0	0	0	0	2	0	0	0
ko04141 Protein processing in endoplasmic reticulum	8412	8499	8701	8658	12448	12451	12587	12521	10096	10188	10311	10445
K04079 molecular chaperone HtpG	8026	8029	8183	8223	11366	11368	11453	11419	9422	9406	9524	9661
K08852 serine/threonine-protein kinase/endoribonuclease IRE1 [EC:2.7.11.1 3.1.26.-]	0	0	0	0	0	0	0	0	0	0	1	0
K09487 heat shock protein 90kDa beta	1	0	0	0	0	0	0	0	0	0	0	0
K09523 DnaJ homolog subfamily C member 3	21	18	12	25	61	50	56	49	42	49	37	36
K13525 transitional endoplasmic reticulum ATPase	3	2	1	1	1	5	4	0	4	16	19	27
K13993 HSP20 family protein	341	426	477	387	1017	1023	1068	1052	603	665	686	678
K13996 protein disulfide-isomerase [EC:5.3.4.1]	0	0	0	0	0	0	0	0	0	0	0	1
K14002 DnaJ-related protein SCJ1	0	0	0	0	0	0	0	0	0	0	1	0
K14005 protein transport protein SEC31	0	0	0	0	0	0	0	0	2	0	0	0
K14006 protein transport protein SEC23	0	0	0	0	0	0	0	0	0	0	3	0
K14007 protein transport protein SEC24	0	2	0	0	0	0	0	0	0	0	0	0
K14026 SEL1 protein	0	0	0	0	0	0	0	0	0	1	0	0
K16196 eukaryotic translation initiation factor 2-alpha kinase 4 [EC:2.7.11.1]	0	0	0	0	0	0	0	0	0	1	0	0
ko04130 SNARE interactions in vesicular transport	0	0	0	0	0	0	0	0	0	0	0	1
K08517 vesicle transport protein SEC22	0	0	0	0	0	0	0	0	0	0	0	1
ko04120 Ubiquitin mediated proteolysis	2	4	6	2	0	0	0	0	0	3	5	1
K03178 ubiquitin-activating enzyme E1 [EC:6.2.1.45]	0	0	0	0	0	0	0	0	0	0	1	0
K03349 anaphase-promoting complex subunit 2	0	1	0	0	0	0	0	0	0	0	0	0
K03355 anaphase-promoting complex subunit 8	0	0	1	0	0	0	0	0	0	0	0	0
K03364 cell division cycle 20-like protein 1, cofactor of APC complex	0	0	0	0	0	0	0	0	0	2	0	0
K05633 NEDD4-like E3 ubiquitin-protein ligase WWP1 [EC:2.3.2.26]	0	0	2	0	0	0	0	0	0	0	0	0
K10595 E3 ubiquitin-protein ligase HERC2 [EC:2.3.2.26]	1	0	2	0	0	0	0	0	0	0	3	0
K10614 E3 ubiquitin-protein ligase HERC3 [EC:2.3.2.26]	0	1	1	1	0	0	0	0	0	1	0	1
K10615 E3 ubiquitin-protein ligase HERC4 [EC:2.3.2.26]	1	2	0	1	0	0	0	0	0	0	0	0
K10685 ubiquitin-like 1-activating enzyme E1 B [EC:6.2.1.45]	0	0	0	0	0	0	0	0	0	0	1	0
ko04122 Sulfur relay system	30434	29942	29174	29910	24195	24700	24441	24495	25246	24898	24555	25199
K00566 tRNA-uridine 2-sulfurtransferase [EC:2.8.1.13]	4897	5091	5183	5058	8673	8651	8798	8422	7448	7019	7170	7094
K03154 sulfur carrier protein	598	599	514	556	586	613	750	622	512	505	540	528
K03636 sulfur-carrier protein	598	593	581	606	322	319	345	372	380	412	370	387
K04085 tRNA 2-thiouridine synthesizing protein A [EC:2.8.1.-]	569	582	607	550	336	308	308	339	388	398	378	385
K07235 tRNA 2-thiouridine synthesizing protein D [EC:2.8.1.-]	350	364	353	346	197	230	235	182	399	384	411	415
K07236 tRNA 2-thiouridine synthesizing protein C	763	722	685	705	424	432	431	435	432	434	411	531
K07237 tRNA 2-thiouridine synthesizing protein B	615	633	577	560	361	403	351	374	381	352	344	356
K11179 tRNA 2-thiouridine synthesizing protein E [EC:2.8.1.-]	645	631	565	639	401	372	399	439	356	314	347	383
K11996 adenylyltransferase and sulfurtransferase [EC:2.7.7.80 2.8.1.11]	3	9	5	7	1	1	0	0	7	1	4	1
K21028 molybdopterin synthase sulfurtransferase [EC:2.8.1.11]	795	811	622	671	418	490	439	381	1082	1073	934	1009
K21029 molybdopterin-synthase adenylyltransferase [EC:2.7.7.80]	1608	1680	1595	1721	1939	2114	2095	2081	1714	1598	1764	1618
K21140 [CysO sulfur-carrier protein]-S-L-cysteine hydrolase [EC:3.13.1.6]	5	12	22	1	11	0	0	0	0	0	10	0
K21147 sulfur-carrier protein adenylyltransferase/sulfurtransferase [EC:2.7.7.80 2.7.7.- 2.8.1.11 2.8.1.-]	5	9	5	7	8	11	4	11	9	10	5	14
ko03050 Proteasome	4	7	4	5	4	5	0	7	22	23	23	31
K03039 26S proteasome regulatory subunit N9	0	1	0	0	0	0	0	0	0	0	0	0
K03063 26S proteasome regulatory subunit T3	0	0	0	0	0	0	0	0	1	0	1	0
K03420 proteasome regulatory subunit	0	2	2	0	0	0	0	0	2	3	8	2
K03432 proteasome alpha subunit [EC:3.4.25.1]	0	0	0	1	2	1	0	0	6	9	1	6
K03433 proteasome beta subunit [EC:3.4.25.1]	1	0	0	0	0	0	0	0	6	1	7	3
K13527 proteasome-associated ATPase	3	4	2	4	2	4	0	7	7	10	6	20
ko03018 RNA degradation	82190	82392	84199	83065	95078	96446	95903	94316	88501	88448	88797	88041
K00962 polyribonucleotide nucleotidyltransferase [EC:2.7.7.8]	7679	7904	8044	8058	8614	8710	8825	8374	8209	8316	7938	8093
K00970 poly(A) polymerase [EC:2.7.7.19]	3405	3310	3282	3277	2105	2040	2088	2169	2083	2185	1965	2123
K03628 transcription termination factor Rho	3808	3769	4105	4006	4689	4575	5157	5110	4393	4267	4525	4306
K03654 ATP-dependent DNA helicase RecQ [EC:3.6.4.12]	9850	9595	10348	9987	18898	18974	19017	18434	15542	14692	15454	14979
K03666 host factor-I protein	541	586	606	538	415	376	374	360	325	429	373	382
K03679 exosome complex component RRP4	0	2	0	0	0	0	0	0	2	2	3	11
K03732 ATP-dependent RNA helicase RhlB [EC:3.6.4.13]	3058	2982	2950	2932	1795	1837	1830	1735	1790	1813	1701	1762
K04043 molecular chaperone DnaK	6782	6847	7192	6984	7162	7302	7013	6897	6986	7299	7201	6902
K04077 chaperonin GroEL	4258	4169	4478	4105	5113	5245	4692	4639	5013	5069	5107	4892
K05592 ATP-dependent RNA helicase DeaD [EC:3.6.4.13]	6496	6599	6481	6510	6860	7077	7188	7071	7441	7488	7184	7423
K07573 exosome complex component CSL4	0	0	0	1	0	0	0	0	5	8	9	3
K08300 ribonuclease E [EC:3.1.26.12]	2774	2841	2769	2880	1795	1858	1938	1914	2096	2241	2152	2136
K08311 putative (di)nucleoside polyphosphate hydrolase [EC:3.6.1.-]	1125	1054	1078	1139	668	725	649	658	698	743	733	740
K11600 exosome complex component RRP41	0	0	0	0	0	1	3	0	4	4	4	1
K11927 ATP-dependent RNA helicase RhlE [EC:3.6.4.13]	3911	4039	3857	4050	4521	4517	4339	4461	3678	3646	3848	3741
K12573 ribonuclease R [EC:3.1.13.1]	8142	8077	8224	8081	8966	8956	9063	8952	7979	8218	8282	8047
K12574 ribonuclease J [EC:3.1.-.-]	1906	1968	2014	1927	3270	3316	3342	3348	3590	3746	3737	3665
K12589 exosome complex component RRP42	0	0	0	0	0	5	0	2	4	5	5	1
K12601 superkiller protein 8	0	0	0	0	1	0	0	0	0	0	0	0
K12614 ATP-dependent RNA helicase DDX6/DHH1 [EC:3.6.4.13]	0	0	2	0	0	0	0	0	0	0	0	0
K18682 ribonucrease Y [EC:3.1.-.-]	1753	1806	1919	1803	3248	3262	3137	3079	2807	2799	2937	2784
Replication and repair	254371	252794	253000	254176	258522	260314	260498	260440	256807	257921	259237	258094
ko03030 DNA replication	78377	78897	80254	79572	91059	92029	92411	91046	85196	84381	85484	84684
K01972 DNA ligase (NAD+) [EC:6.5.1.2]	7294	7394	7403	7548	7446	7596	7788	7460	7698	8173	8061	8046
K02314 replicative DNA helicase [EC:3.6.4.12]	12109	12288	12651	12770	18488	18486	18875	18597	13804	13601	13721	13307
K02316 DNA primase [EC:2.7.7.101]	6773	6889	6719	6761	7903	7819	8041	8151	7274	6722	7058	7247
K02319 DNA polymerase, archaea type [EC:2.7.7.7]	0	2	2	0	2	0	2	6	14	30	12	18
K02322 DNA polymerase II large subunit [EC:2.7.7.7]	5	2	0	3	2	0	2	7	9	34	18	34
K02323 DNA polymerase II small subunit [EC:2.7.7.7]	3	0	0	1	0	0	0	0	3	8	8	17
K02335 DNA polymerase I [EC:2.7.7.7]	10609	10694	10868	10636	11635	11745	11911	11697	10668	11017	11010	10671

K02337 DNA polymerase III subunit alpha [EC:2.7.7.7]	12304	12500	12857	12459	14834	14955	14807	14744	13683	13284	13590	13450
K02338 DNA polymerase III subunit beta [EC:2.7.7.7]	3544	3711	3902	3862	3852	3894	3772	3616	4045	4105	4010	4060
K02339 DNA polymerase III subunit chi [EC:2.7.7.7]	1024	964	913	1029	606	630	545	670	717	679	574	583
K02340 DNA polymerase III subunit delta [EC:2.7.7.7]	3246	3131	3203	3116	3335	3431	3507	3327	3331	3048	3351	3205
K02341 DNA polymerase III subunit delta' [EC:2.7.7.7]	3618	3562	3561	3527	3787	3761	3873	3861	3515	3566	3591	3736
K02342 DNA polymerase III subunit epsilon [EC:2.7.7.7]	3123	3318	3288	3223	4529	4563	4564	4333	3484	3250	3467	3476
K02343 DNA polymerase III subunit gamma/tau [EC:2.7.7.7]	5536	5208	5411	5293	5960	6163	5981	5726	5693	5579	5643	5520
K02344 DNA polymerase III subunit psi [EC:2.7.7.7]	289	255	234	275	161	226	180	208	297	340	346	374
K02345 DNA polymerase III subunit theta [EC:2.7.7.7]	121	128	142	134	92	95	84	89	199	195	211	202
K02683 DNA primase small subunit [EC:2.7.7.102]	0	0	0	0	5	0	0	0	0	2	0	4
K03111 single-strand DNA-binding protein	2962	2949	3065	3127	3031	3075	3098	3041	3808	3804	3864	3883
K03469 ribonuclease HI [EC:3.1.26.4]	1788	1825	1880	1767	2550	2523	2523	2689	2240	2290	2234	2204
K03470 ribonuclease HII [EC:3.1.26.4]	1745	1797	1871	1735	2183	2349	2144	2191	2205	2053	2232	2062
K03471 ribonuclease HIII [EC:3.1.26.4]	75	76	78	74	13	27	19	12	112	105	83	103
K03763 DNA polymerase III subunit alpha, Gram-positive type [EC:2.7.7.7]	2192	2181	2196	2214	635	679	686	610	2336	2415	2351	2395
K04799 flap endonuclease-1 [EC:3.-.-.-]	3	5	0	3	0	0	2	0	9	12	0	11
K04800 replication factor C large subunit	2	0	2	0	5	5	0	2	9	12	9	11
K04801 replication factor C small subunit	3	0	2	1	0	0	2	2	7	9	4	12
K04802 proliferating cell nuclear antigen	1	0	0	1	1	0	0	1	2	4	3	4
K07466 replication factor A1	0	2	0	0	2	0	5	0	11	10	8	14
K10726 replicative DNA helicase Mcm [EC:3.6.4.-]	3	5	2	3	2	5	0	3	5	8	7	11
K10747 DNA ligase 1 [EC:6.5.1.1 6.5.1.6 6.5.1.7]	2	9	0	3	0	2	2	0	14	16	10	11
K10755 replication factor C subunit 2/4	0	0	0	0	0	0	0	0	0	0	2	0
K18882 DNA primase large subunit	2	0	2	3	0	0	0	3	4	6	3	12
ko03410 Base excision repair	44773	44916	44980	45088	43972	44468	44484	43947	44065	45365	45474	44824
K01142 exodeoxyribonuclease III [EC:3.1.11.2]	5333	5424	5434	5283	4344	4223	4228	4264	3684	3715	3665	3741
K01151 deoxyribonuclease IV [EC:3.1.21.2]	1465	1420	1481	1487	2137	2135	2176	2067	2180	2289	2399	2209
K01246 DNA-3-methyladenine glycosylase I [EC:3.2.2.20]	1589	1646	1593	1659	888	984	914	925	1197	1280	1193	1320
K01247 DNA-3-methyladenine glycosylase II [EC:3.2.2.21]	2246	2214	2037	2143	1385	1312	1266	1389	1281	1157	1230	1286
K03575 A/G-specific adenine glycosylase [EC:3.2.2.31]	2237	2158	2127	2192	2876	2750	2886	2942	2958	2982	2997	2932
K03648 uracil-DNA glycosylase [EC:3.2.2.27]	1481	1589	1653	1620	2033	1995	2067	2011	2024	2182	2269	2096
K03649 double-stranded uracil-DNA glycosylase [EC:3.2.2.28]	217	194	160	203	69	68	63	56	413	305	380	407
K03652 DNA-3-methyladenine glycosylase [EC:3.2.2.21]	1241	1110	1012	1146	624	708	600	628	372	402	381	410
K03660 N-glycosylase/DNA lyase [EC:3.2.2.- 4.2.99.18]	284	237	283	257	126	114	96	95	298	296	286	291
K05522 endonuclease VIII [EC:3.2.2.- 4.2.99.18]	439	478	464	488	243	333	289	269	638	669	650	633
K07462 single-stranded-DNA-specific exonuclease [EC:3.1.-.-]	5683	5786	5842	5684	6305	6696	6356	6466	6125	6051	6218	6025
K10563 formamidopyrimidine-DNA glycosylase [EC:3.2.2.23 4.2.99.18]	2623	2534	2466	2671	1660	1604	1544	1577	2024	2139	2013	2128
K10771 AP endonuclease 1 [EC:4.2.99.18]	0	0	0	0	0	0	0	2	0	0	0	0
K10773 endonuclease III [EC:4.2.99.18]	1858	1881	1994	1913	2106	2124	2200	2017	2231	2375	2409	2324
K13529 AraC family transcriptional regulator, regulatory protein of adaptative response / DNA-3-methyladenine glycosylase II [EC:3.2.2.21]	65	40	62	47	3	1	3	3	71	122	102	101
K21929 uracil-DNA glycosylase [EC:3.2.2.27]	103	103	101	104	91	78	93	78	178	179	198	178
ko03420 Nucleotide excision repair	68927	68654	69435	69165	72260	72411	73537	72615	75462	78424	77456	77000
K03657 DNA helicase II / ATP-dependent DNA helicase PcrA [EC:3.6.4.12]	13959	13587	13834	13842	12531	12381	12758	12375	18755	19996	19413	19434
K03701 excinuclease ABC subunit A	12837	12866	13045	12916	16770	16619	16982	17180	15194	15087	15327	15103
K03702 excinuclease ABC subunit B	6889	7033	6994	7125	6930	6852	7055	6939	6883	7136	6805	6969
K03703 excinuclease ABC subunit C	6235	6121	6226	6094	6834	7122	7032	6976	6308	6712	6659	6527
K03723 transcription-repair coupling factor (superfamily II helicase) [EC:3.6.4.-]	11097	10946	11065	10996	10106	10088	9994	9974	9925	10260	10148	10209
K10841 DNA excision repair protein ERCC-6	1	2	0	4	3	6	6	10	1	4	7	5
ko03430 Mismatch repair	96434	95961	97299	96882	100847	101951	102336	100654	105670	106202	106571	106294
K01141 exodeoxyribonuclease I [EC:3.1.11.1]	3094	3153	3079	3148	1940	1876	1907	1952	2024	1959	1898	1999
K03555 DNA mismatch repair protein MutS	8181	8083	7996	8009	10025	10112	10301	10184	8969	8929	9114	9086
K03572 DNA mismatch repair protein MutL	5814	5784	5754	5890	6807	6647	6918	6824	6349	6272	6196	6187
K03573 DNA mismatch repair protein MutH	424	459	420	436	266	341	326	331	587	544	492	618
K03601 exodeoxyribonuclease VII large subunit [EC:3.1.11.6]	5514	5199	5382	5300	4797	4911	4421	4515	4545	4356	4419	4458
K03602 exodeoxyribonuclease VII small subunit [EC:3.1.11.6]	975	963	972	928	856	892	852	887	761	789	770	826
K06223 DNA adenine methylase [EC:2.1.1.72]	3937	3953	4012	4001	3805	3804	3802	3866	3212	3265	3275	3256
K07456 DNA mismatch repair protein MutS2	3276	3307	3504	3443	4979	4958	5551	5323	4987	5104	5149	4925
K08737 DNA mismatch repair protein MSH6	0	0	0	0	0	0	0	0	0	1	0	0
K10857 exodeoxyribonuclease X [EC:3.1.11.-]	321	375	329	390	265	263	252	245	523	468	545	521
ko03440 Homologous recombination	115605	114607	114030	114803	111260	113115	111375	112172	111679	110741	112283	111588
K01159 crossover junction endodeoxyribonuclease RuvC [EC:3.1.22.4]	1084	1191	1093	1122	1634	1607	1576	1556	1468	1486	1439	1367
K02317 DNA replication protein DnaT	350	344	336	358	201	216	252	217	443	460	455	467
K02686 primosomal replication protein N	216	241	200	216	173	162	166	181	347	354	292	341
K03550 holliday junction DNA helicase RuvA [EC:3.6.4.12]	1507	1510	1466	1461	1832	1809	1759	1854	1762	1757	1758	1745
K03551 holliday junction DNA helicase RuvB [EC:3.6.4.12]	2946	3039	3132	3059	3829	3977	3902	4018	3727	3691	3896	3601
K03553 recombination protein RecA	3020	2886	3154	3134	3405	3368	3476	3560	3545	3530	3761	3492
K03581 exodeoxyribonuclease V alpha subunit [EC:3.1.11.5]	6270	6197	5845	6211	3229	3318	3096	3235	4359	4356	4215	4450
K03582 exodeoxyribonuclease V beta subunit [EC:3.1.11.5]	7894	7640	7007	7779	4271	4573	4291	4800	4797	4655	4728	4680
K03583 exodeoxyribonuclease V gamma subunit [EC:3.1.11.5]	7570	7308	6888	6984	4327	4274	4037	4392	4547	4392	4452	4560
K03584 DNA repair protein RecO (recombination protein O)	1658	1664	1793	1750	2446	2467	2494	2419	2361	2387	2417	2328
K03629 DNA replication and repair protein RecF	3793	3791	3811	3940	3981	4027	4090	3937	3821	3946	4000	4084
K03655 ATP-dependent DNA helicase RecG [EC:3.6.4.12]	8326	8125	8243	8217	8738	8822	8758	8759	9776	9910	9952	10112
K04066 primosomal protein N' (replication factor Y) (superfamily II helicase) [EC:3.6.4.-]	8251	8202	8099	8320	9268	9339	9160	9485	8474	8238	8457	8377
K04067 primosomal replication protein N''	6143	5830	5430	5469	3088	3090	2817	3095	2208	2030	2066	2156
K06187 recombination protein RecR	2326	2250	2051	2203	2072	2152	2132	2276	2132	2206	2161	2234
K10866 DNA repair protein RAD50 [EC:3.6.-.-]	0	0	0	0	2	0	0	0	0	0	0	0
K10877 DNA repair and recombination protein RAD54B [EC:3.6.4.-]	0	0	0	0	0	1	0	0	0	0	0	0
K10901 bloom syndrome protein [EC:3.6.4.12]	0	0	0	1	0	0	0	0	0	0	0	0
ko03450 Non-homologous end-joining	5222	5037	4877	4964	3160	3082	3141	3237	3143	3099	3084	3201
K01971 bifunctional non-homologous end joining protein LigD [EC:6.5.1.1]	3714	3624	3514	3500	2674	2536	2649	2671	2200	2139	2135	2200
K10979 DNA end-binding protein Ku	1505	1408	1363	1461	484	546	490	566	934	948	949	990
ko03460 Fanconi anemia pathway	0	4	0	1	5	4	5	0	21	31	18	29
K10896 fanconi anemia group M protein	0	2	0	0	3	4	0	0	10	21	10	15
Environmental Information Processing	1423762	1411500	1378838	1410317	953242	962025	932646	956282	1095507	1116892	1087436	1117623
Membrane transport	804952	799226	778794	799027	505515	511270	495820	508714	653310	672004	649760	670760
ko02010 ABC transporters	547645	543469	532152	545891	332376	337586	327016	334562	461008	476958	462089	476914
K01995 branched-chain amino acid transport system ATP-binding protein	4231	4260	4059	4370	2411	2442	2382	2302	3121	3284	3050	3227
K01996 branched-chain amino acid transport system ATP-binding protein	3653	3632	3751	3822	2071	2168	2049	2130	2987	3009	2961	3022
K01997 branched-chain amino acid transport system permease protein	5127	4999	5054	5121	2665	2934	2760	2881	3825	3988	3665	3793
K01998 branched-chain amino acid transport system permease protein	8864	8720	8806	8876	4940	4946	4720	4962	5061	5479	5384	5335
K01999 branched-chain amino acid transport system substrate-binding protein	8994	9109	8708	9021	5632	5660	5267	5536	6171	6501	6015	6441
K02000 glycine betaine/proline transport system ATP-binding protein [EC:7.6.2.9]	4639	4718	4750	4732	4895	4809	4933	4927	3930	3915	3968	3858
K02001 glycine betaine/proline transport system permease protein	4094	4130	3946	4040	3765	3730	3559	3709	3061	3121	3061	2990
K02002 glycine betaine/proline transport system substrate-binding protein	7561	7843	7293	7444	5841	5722	5405	5987	4243	4281	4178	4184
K02006 cobalt/nickel transport system ATP-binding protein	802	826	815	843	468	444	414	435	1051	1137	1104	1130
K02007 cobalt/nickel transport system permease protein	882	824	787	911	390	462	401	389	976	1053	917	1070
K02008 cobalt/nickel transport system permease protein	701	636	593	645	316	359	304	304	811	840	706	787
K02009 cobalt/nickel transport protein	360	380	370	375	251	254	229	209	469	467	417	508
K02010 iron(III) transport system ATP-binding protein [EC:7.2.2.7]	3959	3796	3797	3971	2415	2529	2219	2396	3027	3084	2875	3146
K02011 iron(III) transport system permease protein	7035	6903	6708	7117	4153	4286	4217	4327	4604	4858	4768	4831
K02012 iron(III) transport system substrate-binding protein	6176	6052	5748	6226	3509	3690	3512	3693	3641	3779	3558	3864
K02017 molybdate transport system ATP-binding protein [EC:7.3.2.5]	2407	2456	2176	2432	1414	1436	1433	1414	1683	1786	1695	1869
K02018 molybdate transport system permease protein	1537	1587	1431	1439	975	886	903	894	1085	1080	1071	1134
K02020 molybdate transport system substrate-binding protein	2644	2442	2401	2486	1364	1500	1355	1530	2351	2549	2406	2505
K02036 phosphate transport system ATP-binding protein [EC:7.3.2.1]	2994	3062	3102	3145	2911	2966	3018	2925	2859	2895	2973	3007
K02037 phosphate transport system permease protein	5890	5888	5899	6043	5253	5409	5207	5181	4692	4428	4520	4454
K02038 phosphate transport system permease protein	3703	3676	3791	3728	3561	3609	3507	3366	3364	3228	3282	3321
K02040 phosphate transport system substrate-binding protein	4868	4915	4815	4837	5744	5738	5693	5738	4821	4756	4964	4935
K02041 phosphonate transport system ATP-binding protein [EC:7.3.2.2]	3010	2934	2815	2947	1936	1922	1922	2005	1683	1622	1644	1670
K02042 phosphonate transport system permease protein	4162	4339	3910	4053	2562	2582	2608	2669	2881	2864	2547	2876
K02044 phosphonate transport system substrate-binding protein	3908	3879	3761	3746	2563	2546	2465	2502	2132	2176	1973	2202
K02062 thiamine transport system ATP-binding protein	417	456	417	425	311	331	338	273	640	591	627	638
K02063 thiamine transport system permease protein	1025	1031	1061	1113	691	805	694	717	1416	1503	1389	1544
K02064 thiamine transport system substrate-binding protein	594	607	530	599	368	381	379	371	798	887	786	880
K02065 phospholipid/cholesterol/gamma-HCH transport system ATP-binding protein	3638	3659	3835	3683	3431	3671	3358	3531	2807	2832	2817	2776
K02066 phospholipid/cholesterol/gamma-HCH transport system permease protein	4989	4914	5061	5085	4252	4343	4129	4256	3101	3046	3050	2978
K02067 phospholipid/cholesterol/gamma-HCH transport system substrate-binding protein	3831	3838	3776	3784	3765	3884	3626	3634	2808	2777	2824	2757
K02071 D-methionine transport system ATP-binding protein	6454	6312	6158	6340	3960	4117	3921	3972	4593	4751	4576	4898
K02072 D-methionine transport system permease protein	3618	3502	3307	3548	2286	2320	2157	2279	2441	2613	2656	2777
K02073 D-methionine transport system substrate-binding protein	6114	6299	5860	6090	3799	3763	3688	3716	5251	5218	5050	5145
K02193 heme exporter protein A [EC:7.6.2.5]	293	287	253	274	140	137	130	147	536	565	524	614

K02194 heme exporter protein B	1200	1198	1051	1109	553	596	612	635	867	826	794	849
K02195 heme exporter protein C	1473	1485	1375	1377	725	707	724	749	1010	978	971	987
K02196 heme exporter protein D	343	346	287	301	193	177	141	166	268	224	204	289
K02424 L-cystine transport system substrate-binding protein	1933	2054	1989	2026	1320	1408	1394	1345	1119	1020	1200	1224
K02471 vitamin B12/bleomycin/antimicrobial peptide transport system ATP-binding/permease protein	154	157	138	157	190	180	208	189	259	282	278	267
K03523 biotin transport system substrate-specific component	554	570	487	537	181	213	182	157	412	533	445	498
K05643 ATP-binding cassette, subfamily A (ABC1), member 3	0	0	1	0	0	0	0	0	1	0	0	1
K05655 ATP-binding cassette, subfamily B (MDR/TAP), member 8	1	0	0	1	0	0	0	0	0	3	1	0
K05656 ATP-binding cassette, subfamily B (MDR/TAP), member 9	1	0	0	1	0	1	0	0	0	0	0	1
K05657 ATP-binding cassette, subfamily B (MDR/TAP), member 10	4	2	3	3	0	1	0	0	2	1	6	5
K05658 ATP-binding cassette, subfamily B (MDR/TAP), member 1 [EC:7.6.2.2]	11	6	5	8	2	3	6	3	16	11	12	12
K05659 ATP-binding cassette, subfamily B (MDR/TAP), member 4 [EC:7.6.2.2]	1	0	1	0	0	0	0	0	2	2	3	3
K05661 ATP-binding cassette, subfamily B (MDR/TAP), member 6	0	0	0	0	1	0	0	0	0	0	1	0
K05662 ATP-binding cassette, subfamily B (MDR/TAP), member 7	1	0	0	0	0	0	0	0	0	0	0	0
K05663 ATP-binding cassette, subfamily B, mitochondrial transporter ATM	0	0	0	0	0	1	0	0	0	0	0	0
K05666 ATP-binding cassette, subfamily C (CFTR/MRP), member 2	0	0	0	0	0	1	0	0	1	1	0	0
K05673 ATP-binding cassette, subfamily C (CFTR/MRP), member 4	0	1	1	1	0	0	0	0	0	1	0	0
K05685 macrolide transport system ATP-binding/permease protein [EC:3.6.3.-]	3942	3828	3702	3735	2174	2211	2058	2263	2446	2393	2267	2274
K05772 tungstate transport system substrate-binding protein	20	19	12	19	17	15	19	16	122	113	93	113
K05773 tungstate transport system permease protein	9	12	13	15	8	7	18	19	56	85	78	108
K05776 molybdate transport system ATP-binding protein	1641	1674	1684	1589	3015	2877	3120	2958	2921	2963	3065	2958
K05813 sn-glycerol 3-phosphate transport system substrate-binding protein	1650	1637	1597	1651	759	856	794	872	2238	2249	2177	2258
K05814 sn-glycerol 3-phosphate transport system permease protein	1486	1525	1472	1455	679	687	686	661	1939	2126	1960	2094
K05815 sn-glycerol 3-phosphate transport system permease protein	1430	1473	1454	1432	564	568	631	616	1862	2080	1934	2062
K05816 sn-glycerol 3-phosphate transport system ATP-binding protein [EC:7.6.2.10]	1417	1455	1379	1482	751	813	831	763	1953	2111	1966	2191
K05845 osmoprotectant transport system substrate-binding protein	1816	1829	1774	1837	1056	1183	1054	1004	1469	1618	1480	1594
K05846 osmoprotectant transport system permease protein	2582	2539	2373	2460	1624	1615	1612	1734	2051	2084	1966	2113
K05847 osmoprotectant transport system ATP-binding protein [EC:7.6.2.9]	3223	3345	3177	3330	1840	1857	1767	1827	1939	2129	1941	1995
K06073 vitamin B12 transport system permease protein	516	507	527	498	385	371	340	310	728	759	711	728
K06074 vitamin B12 transport system ATP-binding protein [EC:7.6.2.8]	420	399	409	464	312	293	296	260	625	573	558	587
K06159 multidrug/microcin transport system ATP-binding/permease protein	940	895	894	944	601	727	627	639	1228	1354	1355	1229
K06160 putative pyoverdin transport system ATP-binding/permease protein	2	2	2	3	1	1	0	1	0	1	1	1
K06726 D-ribose pyranase [EC:5.4.99.62]	343	288	336	358	208	202	204	183	448	434	452	407
K06857 tungstate transport system ATP-binding protein [EC:7.3.2.6]	8	22	19	25	7	19	23	12	80	63	88	77
K06858 vitamin B12 transport system substrate-binding protein	1776	1675	1598	1714	942	1006	967	1012	983	1113	981	1106
K06861 lipopolysaccharide export system ATP-binding protein [EC:3.6.3.-]	1919	2093	1960	1967	2553	2694	2615	2526	2186	2174	2200	2212
K07091 lipopolysaccharide export system permease protein	3525	3823	3800	3887	5367	5518	5515	5296	4388	4170	4460	4250
K07122 phospholipid transport system transporter-binding protein	651	716	699	742	448	478	415	376	432	400	449	453
K07323 phospholipid transport system substrate-binding protein	1092	1033	1004	1109	739	737	660	691	616	777	672	726
K08712 ATP-binding cassette, subfamily G (WHITE), member 2, SNQ2	0	0	0	0	0	2	0	0	0	0	0	0
K09688 capsular polysaccharide transport system permease protein	162	171	164	187	267	282	247	251	45	50	44	34
K09689 capsular polysaccharide transport system ATP-binding protein [EC:7.6.2.12]	163	148	132	112	199	233	203	174	14	9	7	20
K09690 lipopolysaccharide transport system permease protein	3240	3339	3241	3092	1923	1867	1836	2024	1569	1523	1497	1495
K09691 lipopolysaccharide transport system ATP-binding protein	3037	2905	2996	3065	2109	2092	1949	2071	1287	1291	1352	1422
K09692 teichoic acid transport system permease protein	442	426	451	451	105	125	113	101	313	385	324	385
K09693 teichoic acid transport system ATP-binding protein [EC:7.5.2.4]	706	689	668	748	127	150	143	103	546	635	681	740
K09694 lipooligosaccharide transport system permease protein	0	2	2	0	0	0	0	0	0	0	3	0
K09695 lipooligosaccharide transport system ATP-binding protein	3	2	6	3	2	0	0	1	4	7	4	4
K09696 sodium transport system permease protein	3	12	10	4	7	7	3	4	34	24	21	21
K09697 sodium transport system ATP-binding protein [EC:7.2.2.4]	4	10	6	7	2	2	0	4	9	3	24	19
K09808 lipoprotein-releasing system permease protein	4499	4629	4754	4653	7028	6978	7290	7387	6099	6030	6108	6050
K09810 lipoprotein-releasing system ATP-binding protein [EC:3.6.3.-]	788	877	887	875	1824	1829	1885	1898	1662	1537	1590	1589
K09811 cell division transport system permease protein	3783	3733	3688	3814	3684	3912	3776	3795	3557	3810	3908	3809
K09812 cell division transport system ATP-binding protein	2764	2846	2992	2918	2654	2587	2731	2653	2805	2827	2885	2874
K09815 zinc transport system substrate-binding protein	4900	4844	4722	4900	4342	4503	4293	4560	4145	3968	3963	4032
K09816 zinc transport system permease protein	3819	3711	3730	3775	3561	3486	3358	3363	3193	3144	3284	3131
K09817 zinc transport system ATP-binding protein [EC:7.2.2.-]	3825	3835	3776	3825	3777	3667	3650	3735	3444	3195	3346	3425
K09969 general L-amino acid transport system substrate-binding protein	1983	2075	1854	1891	1450	1430	1335	1372	717	745	696	627
K09970 general L-amino acid transport system permease protein	317	288	301	342	445	468	436	439	157	155	170	160
K09971 general L-amino acid transport system permease protein	267	273	319	296	456	500	501	477	142	156	132	138
K09972 general L-amino acid transport system ATP-binding protein [EC:7.4.2.1]	173	136	131	150	234	279	202	261	74	52	43	65
K09996 arginine transport system substrate-binding protein	702	688	645	707	385	375	413	348	1173	1238	1108	1197
K09997 arginine transport system substrate-binding protein	269	250	275	262	215	276	264	237	268	287	277	235
K09998 arginine transport system permease protein	393	357	376	334	238	238	213	233	483	569	452	463
K09999 arginine transport system permease protein	399	389	375	416	281	332	272	294	572	633	586	652
K10000 arginine transport system ATP-binding protein [EC:7.4.2.1]	476	418	422	525	276	340	330	341	655	674	638	648
K10001 glutamate/aspartate transport system substrate-binding protein	2101	2176	2111	2150	1252	1249	1245	1282	1412	1395	1385	1390
K10002 glutamate/aspartate transport system permease protein	1423	1356	1303	1195	818	910	810	814	855	809	813	904
K10003 glutamate/aspartate transport system permease protein	1278	1269	1297	1284	811	909	806	821	927	915	888	950
K10004 glutamate/aspartate transport system ATP-binding protein [EC:7.4.2.1]	1324	1218	1269	1350	833	853	809	832	889	945	854	901
K10005 glutamate transport system substrate-binding protein	2	2	5	2	0	2	0	3	4	5	2	11
K10006 glutamate transport system permease protein	0	0	2	5	2	3	1	2	4	1	0	4
K10007 glutamate transport system permease protein	2	5	9	1	4	6	3	3	7	13	4	20
K10008 glutamate transport system ATP-binding protein [EC:7.4.2.1]	19	20	21	27	5	5	3	7	21	17	13	15
K10009 L-cystine transport system permease protein	1688	1671	1593	1615	1186	1186	1045	1154	860	824	796	907
K10010 L-cystine transport system ATP-binding protein [EC:7.4.2.1]	532	544	523	584	573	605	518	555	570	549	510	533
K10013 lysine/arginine/ornithine transport system substrate-binding protein	805	722	734	769	528	644	566	569	978	968	1002	983
K10014 histidine transport system substrate-binding protein	1528	1520	1557	1549	846	978	899	1025	1002	995	941	1021
K10015 histidine transport system permease protein	1239	1275	1132	1183	727	679	665	661	692	822	763	767
K10016 histidine transport system permease protein	982	1051	961	1068	597	616	573	607	767	731	728	843
K10017 histidine transport system ATP-binding protein [EC:7.4.2.1]	1450	1443	1320	1375	900	794	761	842	856	878	856	864
K10018 octopine/nopaline transport system substrate-binding protein	5	1	4	0	0	0	5	2	6	3	3	9
K10019 octopine/nopaline transport system permease protein	1	0	0	0	0	1	1	0	4	0	5	1
K10020 octopine/nopaline transport system permease protein	2	1	0	0	1	5	2	0	3	1	3	3
K10021 octopine/nopaline transport system ATP-binding protein [EC:7.4.2.1]	4	4	4	1	0	5	0	1	3	3	1	5
K10022 arginine/ornithine transport system substrate-binding protein	1984	1818	1846	1841	994	985	1047	990	646	586	574	575
K10023 arginine/ornithine transport system permease protein	1067	1011	974	1049	581	598	526	543	322	300	349	280
K10024 arginine/ornithine transport system permease protein	1109	1133	1058	1018	531	622	577	613	416	393	345	297
K10025 arginine/ornithine transport system ATP-binding protein [EC:7.4.2.1]	1030	967	1065	965	601	562	522	586	330	288	319	320
K10036 glutamine transport system substrate-binding protein	662	663	592	619	548	550	497	488	848	918	855	920
K10037 glutamine transport system permease protein	610	637	606	581	440	474	426	407	795	893	811	862
K10038 glutamine transport system ATP-binding protein [EC:7.4.2.1]	527	470	495	473	343	362	293	339	762	687	750	714
K10039 aspartate/glutamate/glutamine transport system substrate-binding protein	56	65	75	68	19	23	22	39	54	79	60	68
K10040 aspartate/glutamate/glutamine transport system permease protein	109	116	102	108	40	42	40	33	97	116	95	106
K10041 aspartate/glutamate/glutamine transport system ATP-binding protein [EC:7.4.2.1]	59	73	94	91	14	42	26	33	73	78	66	93
K10107 capsular polysaccharide transport system permease protein	245	262	263	253	429	421	419	365	93	98	70	77
K10108 maltose/maltodextrin transport system substrate-binding protein	740	770	737	767	558	590	553	576	1047	1143	1009	1084
K10109 maltose/maltodextrin transport system permease protein	942	1024	994	967	708	750	691	694	1383	1417	1357	1361
K10110 maltose/maltodextrin transport system permease protein	575	591	583	579	388	422	401	420	717	861	848	846
K10111 multiple sugar transport system ATP-binding protein [EC:3.6.3.-]	2239	2152	2095	2158	1339	1412	1414	1350	2162	2200	2009	2254
K10112 multiple sugar transport system ATP-binding protein	3863	3896	3839	3936	2048	2019	2003	2103	2015	2306	2122	2109
K10117 raffinose/stachyose/melibiose transport system substrate-binding protein	8532	8247	8648	8405	1482	1482	1500	1408	8794	9580	9305	9575
K10118 raffinose/stachyose/melibiose transport system permease protein	7026	6708	6919	6980	1310	1429	1341	1283	7569	7993	7766	7928
K10119 raffinose/stachyose/melibiose transport system permease protein	6078	5831	6355	6047	1162	1222	1215	1083	6315	7366	6612	7179
K10188 lactose/L-arabinose transport system substrate-binding protein	402	388	429	419	111	105	125	93	756	773	784	813
K10189 lactose/L-arabinose transport system permease protein	294	280	308	276	73	74	59	57	636	673	653	653
K10190 lactose/L-arabinose transport system permease protein	317	273	280	302	68	83	72	71	597	567	581	643
K10191 lactose/L-arabinose transport system ATP-binding protein	1	2	0	2	0	0	0	0	0	1	1	1
K10192 oligogalacturonide transport system substrate-binding protein	1682	1680	1706	1773	366	419	355	334	1526	1763	1694	1741
K10193 oligogalacturonide transport system permease protein	2280	2198	2197	2347	385	408	383	384	1745	1866	1826	1953
K10194 oligogalacturonide transport system permease protein	2571	2351	2307	2451	374	420	426	415	1678	2057	2008	2035
K10195 oligogalacturonide transport system ATP-binding protein	459	477	423	476	108	134	130	123	878	809	823	847
K10200 N-acetylglucosamine transport system substrate-binding protein	137	125	150	136	21	15	16	19	83	91	85	107
K10201 N-acetylglucosamine transport system permease protein	204	210	252	226	13	11	22	8	135	152	156	160
K10202 N-acetylglucosamine transport system permease protein	454	470	446	439	103	116	129	99	329	361	316	410
K10227 sorbitol/mannitol transport system substrate-binding protein	1676	1661	1470	1489	772	792	734	878	543	475	420	464
K10228 sorbitol/mannitol transport system permease protein	1324	1211	1061	1132	556	612	569	620	396	383	302	322
K10229 sorbitol/mannitol transport system permease protein	1038	953	930	907	550	525	508	573	328	281	283	317
K10232 alpha-glucoside transport system substrate-binding protein	0	0	0	1	0	2	0	0	0	0	0	0
K10233 alpha-glucoside transport system permease protein	1	2	0	2	0	1	1	0	3	1	2	2
K10234 alpha-glucoside transport system permease protein	1	0	0	0	4	3	2	1	2	1	4	3

K10235 alpha-glucoside transport system ATP-binding protein	0	1	0	2	1	1	0	0	7	5	3	7
K10236 trehalose/maltose transport system substrate-binding protein	4	4	2	1	2	0	0	0	3	4	6	4
K10237 trehalose/maltose transport system permease protein	59	61	70	55	11	18	17	7	59	56	53	55
K10238 trehalose/maltose transport system permease protein	7	7	10	9	0	0	0	0	11	2	8	10
K10240 cellobiose transport system substrate-binding protein	3	5	2	1	0	1	1	0	4	3	8	3
K10241 cellobiose transport system permease protein	464	424	432	450	160	211	191	178	537	592	470	567
K10242 cellobiose transport system permease protein	182	174	199	208	69	66	69	61	239	259	257	283
K10439 ribose transport system substrate-binding protein	4577	4555	4434	4547	2595	2701	2612	2625	4447	4753	4538	4753
K10440 ribose transport system permease protein	4661	4416	4450	4525	2291	2528	2277	2299	4390	4478	4297	4530
K10441 ribose transport system ATP-binding protein [EC:7.5.2.7]	6976	6946	6751	6969	3754	3967	3804	3849	5483	5969	5577	5740
K10537 L-arabinose transport system substrate-binding protein	600	558	572	596	396	413	403	449	830	898	775	866
K10538 L-arabinose transport system permease protein	447	460	471	488	299	363	359	372	689	748	727	759
K10539 L-arabinose transport system ATP-binding protein [EC:7.5.2.12]	854	843	867	891	551	589	602	622	1226	1171	1274	1245
K10540 methyl-galactoside transport system substrate-binding protein	1930	1915	1939	1943	735	831	683	737	2077	2340	2113	2231
K10541 methyl-galactoside transport system permease protein	1704	1646	1822	1711	656	746	727	673	1809	1999	1943	1987
K10542 methyl-galactoside transport system ATP-binding protein [EC:7.5.2.11]	2100	2132	2028	2140	929	1011	931	938	2422	2479	2372	2455
K10543 D-xylose transport system substrate-binding protein	884	844	806	845	467	511	520	482	1041	1102	1021	1007
K10544 D-xylose transport system permease protein	673	719	699	637	493	464	524	560	643	703	640	672
K10545 D-xylose transport system ATP-binding protein [EC:7.5.2.10]	1106	1188	1121	1163	689	759	751	800	1365	1528	1350	1372
K10546 putative multiple sugar transport system substrate-binding protein	58	52	62	68	107	77	73	96	81	90	70	80
K10547 putative multiple sugar transport system permease protein	62	50	52	57	65	63	65	87	61	100	104	87
K10548 putative multiple sugar transport system ATP-binding protein [EC:7.5.2.-]	149	120	113	144	90	110	81	95	117	149	146	148
K10549 D-allose transport system substrate-binding protein	5	7	13	6	3	5	8	6	61	72	83	66
K10550 D-allose transport system permease protein	11	7	11	10	6	3	2	7	85	91	79	60
K10551 D-allose transport system ATP-binding protein [EC:7.5.2.8]	29	26	34	23	4	6	16	10	109	99	116	122
K10552 fructose transport system substrate-binding protein	0	2	0	0	0	4	0	0	0	6	2	1
K10553 fructose transport system permease protein	0	0	0	0	3	0	2	0	1	1	0	4
K10554 fructose transport system ATP-binding protein	0	0	2	0	0	0	0	0	0	0	2	3
K10555 AI-2 transport system substrate-binding protein	651	663	638	637	457	511	448	469	823	868	765	857
K10556 AI-2 transport system permease protein	666	612	597	652	418	465	456	459	887	818	809	863
K10557 AI-2 transport system permease protein	663	577	538	637	500	453	429	498	742	702	772	786
K10558 AI-2 transport system ATP-binding protein	1018	928	850	970	659	726	725	687	1258	1263	1259	1267
K10559 rhamnose transport system substrate-binding protein	732	690	663	764	436	544	492	458	820	902	823	893
K10560 rhamnose transport system permease protein	634	638	548	651	464	529	430	452	826	893	820	849
K10561 rhamnose transport system permease protein	626	607	658	640	402	416	444	435	830	815	749	780
K10562 rhamnose transport system ATP-binding protein [EC:7.5.2.-]	971	1032	957	1092	705	736	714	659	1216	1376	1203	1293
K10823 oligopeptide transport system ATP-binding protein	2717	2678	2653	2620	878	897	800	793	2781	3113	2881	3056
K10824 nickel transport system ATP-binding protein [EC:7.2.2.11]	536	571	554	629	478	462	463	434	735	760	841	784
K10829 ferric hydroxamate transport system ATP-binding protein [EC:7.2.2.16]	654	713	612	690	479	467	477	487	719	724	832	858
K10830 manganese/zinc transport system ATP-binding protein [EC:7.2.2.5]	35	36	33	46	2	4	9	5	43	49	29	44
K11004 ATP-binding cassette, subfamily B, bacterial HlyB/CyaB	16	6	9	11	5	5	13	3	126	125	123	129
K11050 multidrug/hemolysin transport system ATP-binding protein	8	6	13	9	0	2	3	3	13	11	8	10
K11051 multidrug/hemolysin transport system permease protein	3	3	5	3	2	2	3	0	4	11	12	8
K11069 spermidine/putrescine transport system substrate-binding protein	4047	4125	4217	4157	4356	4057	4167	4281	3783	3881	3800	3789
K11070 spermidine/putrescine transport system permease protein	3718	3764	3752	3871	3335	3248	3220	3292	2712	2761	2833	2914
K11071 spermidine/putrescine transport system permease protein	4117	4067	3997	3992	3241	3289	3182	3386	3059	3085	3023	2967
K11072 spermidine/putrescine transport system ATP-binding protein [EC:7.6.2.11]	5793	5782	5714	5778	5372	5378	5359	5353	4753	4609	4770	4703
K11073 putrescine transport system substrate-binding protein	9814	9998	9252	9772	5568	5291	5207	5640	3824	3884	3541	3705
K11074 putrescine transport system permease protein	1880	1766	1725	1721	1083	1057	1113	1087	1159	1134	1156	1065
K11075 putrescine transport system permease protein	2021	2159	2083	2159	1297	1319	1213	1299	1148	1136	1161	1119
K11076 putrescine transport system ATP-binding protein	2267	2190	2339	2242	1350	1431	1263	1395	1416	1444	1489	1453
K11077 mannopine transport system substrate-binding protein	2	0	0	0	0	0	0	2	0	4	0	0
K11078 mannopine transport system permease protein	1	2	2	0	0	0	0	0	1	3	3	1
K11079 mannopine transport system permease protein	0	0	0	0	0	0	0	0	0	1	2	1
K11080 mannopine transport system ATP-binding protein	0	0	1	1	7	0	0	2	2	1	3	0
K11081 2-aminoethylphosphonate transport system substrate-binding protein	434	413	469	470	127	113	121	131	835	946	807	871
K11082 2-aminoethylphosphonate transport system permease protein	253	283	266	298	71	68	70	65	479	489	446	539
K11083 2-aminoethylphosphonate transport system permease protein	309	333	292	348	84	101	96	69	594	660	609	653
K11084 2-aminoethylphosphonate transport system ATP-binding protein	492	491	427	540	151	161	124	134	964	1048	885	998
K11085 ATP-binding cassette, subfamily B, bacterial MsbA [EC:3.6.3.-]	2478	2726	2768	2788	5640	5360	5728	5493	4930	4875	4906	4912
K11604 manganese/iron transport system substrate-binding protein	371	375	350	412	98	100	110	102	786	844	757	753
K11605 manganese/iron transport system permease protein	316	328	332	333	84	73	93	91	626	718	579	635
K11606 manganese/iron transport system permease protein	267	286	233	224	75	92	103	89	522	566	564	590
K11607 manganese/iron transport system ATP-binding protein	335	319	310	361	70	86	90	91	555	662	572	648
K11631 bacitracin transport system ATP-binding protein	0	3	3	3	3	5	1	3	2	2	5	3
K11632 bacitracin transport system permease protein	2	3	0	1	0	0	0	0	0	0	0	0
K11705 iron/zinc/manganese/copper transport system permease protein	0	0	0	0	0	0	0	0	1	0	0	1
K11707 manganese/zinc/iron transport system substrate-binding protein	4	10	7	13	2	0	2	0	0	1	0	3
K11708 manganese/zinc/iron transport system permease protein	2	16	5	19	0	3	0	2	0	0	4	1
K11709 manganese/zinc/iron transport system permease protein	8	16	10	23	0	0	3	1	0	2	2	0
K11710 manganese/zinc/iron transport system ATP- binding protein [EC:7.2.2.5]	7	16	5	15	3	4	1	0	0	2	0	3
K11720 lipopolysaccharide export system permease protein	2712	2636	2690	2789	3417	3561	3445	3184	2652	2747	2803	2631
K11953 bicarbonate transport system ATP-binding protein [EC:3.6.3.-]	0	0	0	0	0	0	0	0	2	0	1	0
K11955 neutral amino acid transport system permease protein	0	0	0	1	0	0	0	0	0	0	0	0
K11958 neutral amino acid transport system ATP-binding protein	0	0	1	0	0	1	0	0	0	0	0	1
K11959 urea transport system substrate-binding protein	2731	2773	2642	2774	1360	1392	1337	1460	1960	1887	1851	1874
K11960 urea transport system permease protein	3481	3212	3082	3362	1720	1594	1548	1634	2183	2208	2201	2193
K11961 urea transport system permease protein	2023	1832	1773	1819	875	853	797	841	1416	1467	1395	1514
K11962 urea transport system ATP-binding protein	1572	1565	1598	1718	727	680	702	801	1303	1163	1172	1266
K11963 urea transport system ATP-binding protein	1490	1445	1381	1400	637	653	665	721	1045	1148	1113	1108
K12368 dipeptide transport system substrate-binding protein	9050	9007	8930	8954	5453	5432	5421	5754	3938	3966	3753	3792
K12369 dipeptide transport system permease protein	2231	2235	2167	2115	1169	1278	1279	1286	1341	1464	1351	1476
K12370 dipeptide transport system permease protein	1737	1757	1639	1753	1128	1109	1018	996	1143	1128	1156	1126
K12371 dipeptide transport system ATP-binding protein	2117	1909	1903	2064	1220	1222	1236	1167	1456	1318	1302	1337
K12372 dipeptide transport system ATP-binding protein	2113	2201	2082	2043	1233	1304	1228	1226	1328	1323	1247	1265
K12536 ATP-binding cassette, subfamily C, bacterial exporter for protease/lipase	4110	4027	3615	3782	2115	1930	1898	2258	1268	1166	1041	1160
K12541 ATP-binding cassette, subfamily C, bacterial LapB	4305	3982	3648	3962	2987	2939	2738	3068	1141	1017	989	1073
K13409 ATP-binding cassette, subfamily B, bacterial RaxB	4471	4504	4283	4386	2178	2241	2051	2258	2795	3111	2867	2966
K13889 glutathione transport system substrate-binding protein	1335	1288	1371	1349	1235	1362	1307	1313	1942	1908	1894	1999
K13890 glutathione transport system permease protein	541	571	527	599	425	443	408	434	677	733	691	722
K13891 glutathione transport system permease protein	579	604	569	556	440	455	384	431	780	801	738	743
K13892 glutathione transport system ATP-binding protein	1145	1195	1065	1166	843	857	808	770	1574	1616	1630	1657
K13893 microcin C transport system substrate-binding protein	7254	7335	7122	7214	4225	4138	3876	4182	3407	3270	3234	3416
K13894 microcin C transport system permease protein	3381	3511	3283	3487	1782	1881	1863	1985	1751	1765	1718	1709
K13895 microcin C transport system permease protein	3293	3074	2934	3035	1763	1678	1673	1779	1640	1486	1554	1525
K13896 microcin C transport system ATP-binding protein	3696	3704	3213	3397	1982	1910	1851	2140	2067	2101	2020	2131
K14698 ATP-binding cassette, subfamily B, bacterial IrtA [EC:3.6.3.-]	1	0	1	0	0	0	0	1	0	0	0	0
K14699 ATP-binding cassette, subfamily B, bacterial IrtB [EC:3.6.3.-]	17	18	19	22	8	10	1	0	1	4	8	9
K15497 molybdate/tungstate transport system ATP-binding protein [EC:7.3.2.5 7.3.2.6]	0	0	0	0	0	0	0	0	1	0	1	0
K15580 oligopeptide transport system substrate-binding protein	5868	5926	5927	6258	2553	2656	2523	2388	5978	6768	6461	6479
K15581 oligopeptide transport system permease protein	1627	1578	1615	1588	505	579	572	525	1586	1834	1932	1868
K15582 oligopeptide transport system permease protein	1770	1658	1718	1826	521	638	461	510	1844	2110	1855	1972
K15583 oligopeptide transport system ATP-binding protein	2354	2281	2304	2407	805	800	785	674	2312	2657	2299	2453
K15584 nickel transport system substrate-binding protein	1236	1212	1136	1129	836	861	883	799	1535	1660	1515	1630
K15585 nickel transport system permease protein	734	709	691	689	597	526	509	510	933	932	944	928
K15586 nickel transport system permease protein	643	642	630	672	472	477	474	502	832	938	841	903
K15587 nickel transport system ATP-binding protein [EC:7.2.2.11]	540	486	446	540	377	443	406	454	614	705	606	692
K15598 putative hydroxymethylpyrimidine transport system substrate-binding protein	336	365	284	267	101	110	60	69	706	749	725	708
K15599 putative hydroxymethylpyrimidine transport system permease protein	230	281	231	229	72	59	68	79	507	581	529	490
K15600 putative hydroxymethylpyrimidine transport system ATP-binding protein	290	221	247	296	66	87	64	61	576	545	552	552
K15770 arabinogalactan oligomer / maltooligosaccharide transport system substrate-binding protein	1019	999	1004	1027	461	469	496	485	1235	1264	1246	1317
K15771 arabinogalactan oligomer / maltooligosaccharide transport system permease protein	1257	1202	1364	1273	459	528	490	551	1380	1434	1459	1408
K15772 arabinogalactan oligomer / maltooligosaccharide transport system permease protein	873	839	875	884	354	399	401	348	1019	1051	925	1044
K16012 ATP-binding cassette, subfamily C, bacterial CydC	1016	1134	1010	1099	666	702	730	676	1429	1366	1358	1531
K16013 ATP-binding cassette, subfamily C, bacterial CydD	1120	1174	1096	1166	756	812	752	722	1388	1588	1456	1580
K16014 ATP-binding cassette, subfamily C, bacterial CydCD	41	48	36	53	8	6	8	9	11	23	6	9
K16199 dipeptide transport system substrate-binding protein	17	8	7	7	0	4	2	2	6	5	7	7
K16200 dipeptide transport system permease protein	1	1	2	1	0	1	0	0	2	2	2	0
K16201 dipeptide transport system permease protein	16	5	16	9	4	2	5	2	13	9	11	5
K16202 dipeptide transport system ATP-binding protein	19	16	16	13	3	4	5	2	25	24	24	17
K16299 ATP-binding cassette, subfamily C, bacterial EexD	17	28	26	9	1	5	3	2	9	14	10	17

K16784 biotin transport system ATP-binding protein [EC:3.6.3.-]	0	2	0	0	4	3	2	2	6	5	7	0
K16785 energy-coupling factor transport system permease protein	1679	1505	1694	1719	523	516	523	488	2110	2185	2033	2177
K16786 energy-coupling factor transport system ATP-binding protein [EC:3.6.3.-]	793	701	766	724	213	195	197	169	618	800	753	757
K16787 energy-coupling factor transport system ATP-binding protein [EC:3.6.3.-]	2391	2213	2272	2302	784	805	811	776	2586	2886	2788	2956
K16905 fluoroquinolone transport system permease protein	561	498	575	567	63	103	82	67	408	463	454	495
K16906 fluoroquinolone transport system permease protein	406	365	385	418	79	80	64	69	339	347	354	420
K16907 fluoroquinolone transport system ATP-binding protein [EC:3.6.3.-]	547	594	614	555	101	118	102	86	611	651	712	620
K16915 nickel transport protein	14	13	19	16	14	14	15	19	13	9	27	19
K16916 putative peptide transport system permease protein	0	0	0	2	0	0	0	2	0	0	0	0
K16918 acetoin utilization transport system permease protein	0	0	0	0	0	0	0	2	0	0	0	0
K16920 acetoin utilization transport system ATP-binding protein	0	0	0	0	0	0	0	0	0	0	1	0
K16956 L-cystine transport system substrate-binding protein	0	2	1	2	0	0	0	0	0	2	0	0
K16957 L-cystine transport system substrate-binding protein	56	57	55	58	1	4	6	0	27	22	29	31
K16958 L-cystine transport system permease protein	91	65	65	88	4	4	4	3	46	43	48	47
K16959 L-cystine transport system permease protein	23	30	38	35	8	3	7	6	30	22	28	31
K16960 L-cystine transport system ATP-binding protein [EC:7.4.2.1]	200	191	158	225	15	17	29	20	110	125	119	141
K16961 putative S-methylcysteine transport system substrate-binding protein	126	124	127	110	2	7	3	10	82	76	80	84
K16962 putative S-methylcysteine transport system permease protein	158	156	176	144	6	5	11	4	56	97	100	99
K16963 putative S-methylcysteine transport system ATP-binding protein	227	188	188	193	8	11	9	4	94	121	114	137
K17062 arginine/lysine/histidine/glutamine transport system substrate-binding and permease protein	1	2	0	2	1	1	2	0	1	4	2	3
K17063 arginine/lysine/histidine/glutamine transport system ATP-binding protein [EC:7.4.2.1]	0	0	0	0	0	0	0	1	0	0	0	0
K17073 putative lysine transport system substrate-binding protein	293	314	322	336	83	120	91	72	207	257	219	256
K17074 putative lysine transport system permease protein	567	586	592	564	127	160	140	123	430	564	461	500
K17076 putative lysine transport system ATP-binding protein [EC:3.6.3.-]	508	486	499	521	159	118	134	118	402	459	385	415
K17077 arginine/lysine/histidine transport system permease protein	478	510	500	532	138	138	126	125	415	489	451	424
K17202 erythritol transport system substrate-binding protein	495	519	555	580	384	354	352	323	700	734	746	728
K17203 erythritol transport system permease protein	667	623	598	624	424	421	437	429	878	892	838	823
K17204 erythritol transport system ATP-binding protein	923	980	878	945	604	665	658	582	1219	1259	1134	1268
K17205 putative xylitol transport system substrate-binding protein	3	2	1	1	0	4	0	0	7	6	3	5
K17206 putative xylitol transport system permease protein	32	40	33	36	3	3	3	1	47	40	20	31
K17207 putative xylitol transport system ATP-binding protein	1	1	1	1	2	1	0	2	0	10	5	4
K17209 inositol transport system permease protein	11	9	19	15	2	4	3	1	7	5	5	7
K17210 inositol transport system ATP-binding protein	3	1	1	2	2	2	1	0	0	0	2	1
K17213 inositol transport system substrate-binding protein	718	749	698	784	186	163	217	188	1420	1440	1367	1550
K17214 inositol transport system permease protein	393	428	392	399	105	124	113	110	845	869	839	872
K17215 inositol transport system ATP-binding protein	673	766	624	687	207	217	191	192	1215	1213	1197	1291
K17234 arabinosaccharide transport system substrate-binding protein	346	321	325	302	103	131	123	110	408	439	440	479
K17235 arabinosaccharide transport system permease protein	366	294	322	303	100	83	108	100	420	403	374	421
K17236 arabinosaccharide transport system permease protein	253	264	248	264	102	112	119	116	326	343	322	361
K17237 inositol-phosphate transport system substrate-binding protein	3	0	0	3	4	6	0	2	2	1	5	2
K17238 inositol-phosphate transport system permease protein	5	4	2	3	13	27	10	9	3	5	3	3
K17239 inositol-phosphate transport system permease protein	2	2	0	3	14	8	1	13	1	5	3	1
K17240 inositol-phosphate transport system ATP-binding protein	32	41	36	43	36	53	40	37	22	44	24	23
K17241 alpha-1,4-digalacturonate transport system substrate-binding protein	181	154	179	172	61	51	27	32	109	142	123	121
K17242 alpha-1,4-digalacturonate transport system permease protein	251	235	197	226	55	59	45	45	159	159	126	152
K17243 alpha-1,4-digalacturonate transport system permease protein	374	364	377	376	67	59	72	89	233	248	203	257
K17244 putative chitobiose transport system substrate-binding protein	1	2	1	1	0	0	0	0	0	0	0	0
K17245 putative chitobiose transport system permease protein	64	65	58	71	17	26	18	17	68	63	65	90
K17246 putative chitobiose transport system permease protein	4	5	9	7	2	5	1	0	9	12	10	10
K17311 trehalose transport system substrate-binding protein	1	1	5	3	0	0	0	1	0	0	3	0
K17312 trehalose transport system permease protein	3	2	4	3	1	1	1	0	3	2	5	8
K17313 trehalose transport system permease protein	1	2	5	1	1	0	1	2	0	0	0	1
K17315 glucose/mannose transport system substrate-binding protein	2006	1850	1807	1836	1049	1011	1025	1049	633	543	526	589
K17316 glucose/mannose transport system permease protein	1333	1391	1424	1384	780	719	702	826	452	384	410	404
K17317 glucose/mannose transport system permease protein	1106	1132	1131	1085	611	603	607	655	369	356	349	317
K17318 putative aldouronate transport system substrate-binding protein	9680	9526	9810	9727	2005	2019	2054	1975	13413	13975	13772	14136
K17319 putative aldouronate transport system permease protein	11884	11815	12003	11855	2376	2394	2334	2297	16531	17696	17315	17904
K17320 putative aldouronate transport system permease protein	11366	11243	11559	11595	2314	2269	2113	2060	15701	16901	16601	16970
K17321 glycerol transport system substrate-binding protein	0	1	0	1	3	0	0	3	7	2	13	3
K17322 glycerol transport system permease protein	3	0	1	4	0	0	0	0	4	1	3	1
K17323 glycerol transport system permease protein	5	6	6	9	0	1	2	3	4	5	6	10
K17324 glycerol transport system ATP-binding protein	0	1	0	0	0	2	0	0	0	2	3	5
K17325 glycerol transport system ATP-binding protein	3	2	2	3	0	0	2	2	2	1	4	1
K17326 xylobiose transport system substrate-binding protein	6	6	5	7	7	0	1	2	8	12	13	15
K17327 xylobiose transport system permease protein	0	2	2	2	3	0	0	0	0	4	2	1
K17328 xylobiose transport system permease protein	0	2	3	1	3	2	2	2	0	4	2	0
K17329 N,N'-diacetylchitobiose transport system substrate-binding protein	16	19	25	17	6	3	6	6	7	14	16	13
K17330 N,N'-diacetylchitobiose transport system permease protein	9	8	9	7	3	3	1	1	10	3	14	10
K17331 N,N'-diacetylchitobiose transport system permease protein	43	36	41	35	4	6	3	9	44	29	35	33
K18104 ATP-binding cassette, subfamily B, bacterial AbcA/BmrA [EC:7.6.2.2]	25	12	26	17	7	6	14	13	22	39	28	23
K18216 ATP-binding cassette, subfamily B, tetracycline resistant protein	3	3	3	9	0	0	0	0	1	0	2	0
K18217 ATP-binding cassette, subfamily B, tetracycline resistant protein	6	0	0	7	0	3	0	3	0	4	1	0
K18230 macrolide transport system ATP-binding/permease protein	0	1	0	1	0	0	1	4	0	1	0	0
K18231 macrolide transport system ATP-binding/permease protein	98	118	87	128	17	37	41	29	73	88	87	106
K18887 ATP-binding cassette, subfamily B, multidrug efflux pump	618	563	567	561	123	154	120	116	443	468	484	517
K18888 ATP-binding cassette, subfamily B, multidrug efflux pump	429	420	478	467	147	142	84	93	404	483	378	376
K18889 ATP-binding cassette, subfamily B, multidrug efflux pump	1119	1145	1148	1243	758	720	851	786	1519	1610	1552	1583
K18890 ATP-binding cassette, subfamily B, multidrug efflux pump	1264	1171	1209	1243	733	827	855	748	1743	1660	1653	1763
K18891 ATP-binding cassette, subfamily B, multidrug efflux pump	79	118	107	88	14	26	28	30	86	110	71	97
K18892 ATP-binding cassette, subfamily B, multidrug efflux pump	104	64	125	104	47	43	44	31	99	151	114	130
K18893 ATP-binding cassette, subfamily B, multidrug efflux pump	2737	2631	2668	2588	1465	1468	1340	1464	922	827	795	816
K18895 ATP-binding cassette, subfamily B, salmochelin/enterobactin exporter	12	9	5	7	8	2	8	5	221	175	204	213
K19083 bacitracin transport system ATP-binding protein	0	0	1	0	0	0	0	0	0	1	1	0
K19226 cationic peptide transport system substrate-binding protein	850	838	841	928	616	668	619	570	1259	1238	1223	1323
K19227 cationic peptide transport system permease protein	462	470	466	546	305	341	343	319	683	736	638	732
K19228 cationic peptide transport system permease protein	508	443	499	490	276	319	371	308	663	650	665	661
K19229 cationic peptide transport system ATP-binding protein	521	521	498	524	350	396	379	389	782	818	786	787
K19230 cationic peptide transport system ATP-binding protein	450	431	467	461	273	340	318	297	674	618	640	681
K19309 bacitracin transport system ATP-binding protein	467	420	429	433	41	49	49	24	414	420	394	434
K19310 bacitracin transport system permease protein	395	401	410	449	40	31	34	27	400	433	407	433
K19340 Cu-processing system ATP-binding protein	1510	1468	1289	1390	740	679	701	819	458	352	401	415
K19341 Cu-processing system permease protein	1193	1085	1012	1109	486	596	602	747	335	285	333	321
K19349 pleuromutilin/lincosamide/streptogramin A transport system ATP-binding/permease protein	6	12	7	11	0	0	0	0	21	20	20	18
K19350 lincosamide and streptogramin A transport system ATP-binding/permease protein	205	246	217	241	83	98	85	79	343	386	338	386
K19971 manganese/zinc transport system substrate-binding protein	53	71	51	81	3	10	3	3	41	57	38	54
K19972 manganese/zinc transport system permease protein	28	27	53	45	9	10	8	3	39	50	45	43
K19973 manganese transport system ATP-binding protein [EC:7.2.2.5]	37	30	48	51	19	9	15	12	30	62	44	47
K19975 manganese transport system substrate-binding protein	45	45	63	64	9	10	8	4	50	36	36	65
K19976 manganese transport system permease protein	44	56	35	35	17	21	31	20	49	66	40	45
K20344 ATP-binding cassette, subfamily C, bacteriocin exporter	328	342	385	340	418	459	468	418	169	239	190	248
K20386 ATP-binding cassette, subfamily B, bacterial CylB	131	185	170	151	3	6	12	13	11	15	12	11
K20459 lantibiotic transport system ATP-binding protein	166	156	173	163	66	39	41	43	283	276	267	291
K20460 lantibiotic transport system permease protein	68	70	83	61	39	31	40	29	101	130	112	163
K20461 lantibiotic transport system permease protein	117	114	136	118	38	44	46	44	199	210	208	206
K20490 lantibiotic transport system ATP-binding protein	72	59	74	53	46	51	43	38	106	85	73	88
K20491 lantibiotic transport system permease protein	75	51	60	64	38	42	46	30	90	106	90	95
K20492 lantibiotic transport system permease protein	63	59	45	45	25	20	35	36	61	92	102	123
K23055 putative lysine/arginine/ornithine/histidine/octopine transport system substrate-binding protein	79	65	72	62	51	57	29	51	21	22	29	28
K23056 putative lysine/arginine/ornithine/histidine/octopine transport system permease protein	1278	1172	1144	1292	697	652	591	643	374	368	377	387
K23057 putative lysine/arginine/ornithine/histidine/octopine transport system permease protein	1063	1130	1077	1094	618	601	577	577	365	350	327	348
K23058 putative lysine/arginine/ornithine/histidine/octopine transport system ATP-binding protein [EC:7.4.2.1]	1068	1164	1077	1020	647	612	547	615	383	332	335	311
K23059 arginine/lysine/histidine transporter system substrate-binding protein	317	293	356	374	111	89	118	101	267	284	298	299
K23060 arginine/lysine/histidine transport system ATP-binding protein [EC:7.4.2.1]	366	410	372	345	116	124	148	125	296	380	316	343
K23061 hydroxyproline transporter system substrate-binding protein	1154	1139	1101	1055	635	552	589	596	379	341	281	348
K23062 hydroxyproline transport system permease protein	953	831	796	783	471	470	443	532	276	227	238	238
K23063 hydroxyproline transport system permease protein	840	885	837	815	477	405	408	491	312	236	248	246
K23064 hydroxyproline transport system ATP-binding protein [EC:7.4.2.1]	678	657	583	627	358	366	313	337	209	214	199	179
K23181 ferric citrate transport system substrate-binding protein	3	9	0	1	4	3	6	3	51	56	47	50
K23182 ferric citrate transport system permease protein	5	6	9	3	10	6	0	2	81	87	74	70
K23183 ferric citrate transport system permease protein	0	1	0	1	1	1	0	2	47	66	38	50
K23184 ferric citrate transport system ATP-binding protein [EC:7.2.2.18]	32	24	30	38	18	12	18	19	66	58	56	38

K23185 ferric enterobactin transport system substrate-binding protein	541	605	436	574	394	406	395	414	744	825	785	784
K23186 ferric enterobactin transport system permease protein	588	502	472	572	396	454	427	358	728	718	719	783
K23187 ferric enterobactin transport system permease protein	628	537	481	550	413	371	390	374	772	776	679	848
K23188 ferric enterobactin transport system ATP-binding protein [EC:7.2.2.17]	587	636	561	595	393	414	381	411	738	737	702	751
K23227 ferric hydroxamate transport system substrate-binding protein	575	533	530	597	361	420	353	378	888	857	832	811
K23228 ferric hydroxamate transport system permease protein	1162	1148	1012	1230	960	938	819	859	1493	1528	1485	1597
ko02060 Phosphotransferase system (PTS)	60928	59813	58541	61350	37321	38082	37156	36742	66414	70443	66735	69633
K02757 PTS system, beta-glucoside-specific IIC component	3583	3454	3436	3590	2480	2627	2532	2287	2783	3113	2751	2912
K02784 phosphocarrier protein HPr	143	157	193	150	107	98	126	124	240	242	240	217
K02806 PTS system, nitrogen regulatory IIA component [EC:2.7.1.-]	1131	1124	1011	1130	665	675	639	744	696	734	659	679
K08483 phosphotransferase system, enzyme I, PtsI [EC:2.7.3.9]	1980	1872	2001	2019	1128	1192	1203	1097	2345	2657	2402	2470
K08484 phosphotransferase system, enzyme I, PtsP [EC:2.7.3.9]	5022	4883	4614	4885	2967	2906	2937	3070	3073	3027	2846	3100
K08485 phosphocarrier protein NPr	161	161	176	178	109	128	123	94	222	277	249	230
K11183 phosphocarrier protein FPr	1193	1090	1054	1075	671	712	709	739	1113	1201	1106	1175
K11200 PTS system, 2-O-A-mannosyl-D-glycerate-specific IIC component	4	7	3	5	19	16	18	26	6	10	17	8
K19506 PTS system, fructoselysine/glucoselysine-specific IIA component [EC:2.7.1.-]	308	316	283	328	68	79	57	63	442	539	483	471
K19507 PTS system, fructoselysine/glucoselysine-specific IIB component [EC:2.7.1.-]	327	330	359	348	87	90	127	76	522	529	474	521
K19508 PTS system, fructoselysine/glucoselysine-specific IIC component	619	601	573	674	127	156	148	155	885	935	814	907
K19509 PTS system, fructoselysine/glucoselysine-specific IID component	902	921	896	888	176	172	171	171	931	1050	943	1009
K20112 PTS system, galactose-specific IIA component [EC:2.7.1.204]	29	30	26	27	0	7	1	3	21	16	33	15
K20113 PTS system, galactose-specific IIB component [EC:2.7.1.204]	5	22	19	11	3	5	0	2	11	12	14	12
K20114 PTS system, galactose-specific IIC component	66	47	76	84	16	16	24	16	61	88	71	80
ko03070 Bacterial secretion system	196395	195950	188110	191797	135823	135607	131661	137413	126014	124728	121059	124342
K02452 general secretion pathway protein C	1997	1941	1703	1893	957	952	866	960	1197	1203	1073	1257
K02453 general secretion pathway protein D	10056	9959	9079	9366	4893	4857	4591	5256	4562	4546	4268	4511
K02454 general secretion pathway protein E [EC:7.4.2.8]	7020	7015	6450	6689	3444	3402	3400	3687	3381	3247	3139	3221
K02455 general secretion pathway protein F	4872	4697	4366	4396	2400	2183	2113	2643	2550	2268	2285	2451
K02456 general secretion pathway protein G	2645	2612	2544	2654	1311	1203	1153	1246	1191	1216	1106	1158
K02457 general secretion pathway protein H	1422	1483	1352	1343	740	623	647	751	795	858	741	748
K02458 general secretion pathway protein I	1848	1651	1582	1714	987	888	855	903	800	803	769	795
K02459 general secretion pathway protein J	2988	2992	2671	2811	1520	1469	1328	1553	1411	1260	1171	1328
K02460 general secretion pathway protein K	3033	2986	2819	2924	1488	1466	1390	1522	1714	1605	1542	1768
K02461 general secretion pathway protein L	4283	4211	3641	3977	1954	1956	1940	2150	2394	2094	2013	2227
K02462 general secretion pathway protein M	1575	1474	1347	1527	794	740	697	769	778	759	708	869
K02464 general secretion pathway protein O [EC:3.4.23.43 2.1.1.-]	318	383	282	295	81	92	75	82	650	692	673	688
K02465 general secretion pathway protein S	144	150	171	185	37	41	40	32	303	316	283	318
K03194 type IV secretion system protein VirB1	261	250	226	241	60	78	63	73	546	532	468	498
K03195 type IV secretion system protein VirB10	368	396	363	414	91	108	81	109	826	751	732	786
K03196 type IV secretion system protein VirB11 [EC:7.4.2.8]	338	363	340	373	127	118	93	107	718	845	784	813
K03197 type IV secretion system protein VirB2	111	109	97	138	30	36	26	11	218	203	213	224
K03198 type IV secretion system protein VirB3	0	0	0	0	0	0	0	0	3	3	0	2
K03199 type IV secretion system protein VirB4 [EC:7.4.2.8]	941	982	1029	1041	273	289	274	263	2076	2407	2219	2147
K03200 type IV secretion system protein VirB5	244	241	277	258	58	77	67	75	547	553	575	574
K03201 type IV secretion system protein VirB6	358	371	401	419	108	114	95	106	782	882	811	792
K03202 type IV secretion system protein VirB7	0	1	1	0	0	0	0	2	0	0	0	0
K03203 type IV secretion system protein VirB8	233	233	248	231	46	76	66	57	461	474	408	463
K03204 type IV secretion system protein VirB9	366	377	379	330	98	109	108	105	726	832	670	788
K03205 type IV secretion system protein VirD4 [EC:7.4.2.8]	9143	9101	9102	8845	4544	4597	4258	4384	4998	5468	5298	5521
K03219 type III secretion protein C	2641	2708	2581	2628	1509	1411	1296	1353	754	791	762	777
K03221 type III secretion protein F	326	347	338	362	180	159	201	217	94	119	88	94
K03222 type III secretion protein J	1036	1027	933	1034	557	511	517	550	357	317	330	349
K03223 type III secretion protein L	43	40	30	41	18	16	10	17	11	7	13	9
K03224 ATP synthase in type III secretion protein N [EC:7.4.2.8]	1288	1315	1270	1361	719	708	660	752	457	373	401	387
K03225 type III secretion protein Q	1406	1287	1253	1217	670	684	698	750	394	404	373	397
K03226 type III secretion protein R	935	920	787	859	505	496	489	477	290	273	245	300
K03227 type III secretion protein S	321	326	297	320	213	142	143	128	66	93	77	88
K03228 type III secretion protein T	873	727	741	740	393	420	527	622	251	199	202	227
K03229 type III secretion protein U	1372	1358	1271	1283	662	743	696	765	475	399	396	439
K03230 type III secretion protein V	299	290	267	250	156	195	187	175	106	95	95	89
K04056 type III secretion protein O	235	248	256	250	159	151	196	205	73	77	60	83
K04057 type III secretion protein P	316	308	277	312	151	189	131	161	107	95	87	105
K04058 type III secretion protein W	87	72	88	80	44	49	71	87	30	31	21	16
K04059 type III secretion protein X	589	495	551	504	241	291	291	286	136	144	144	189
K11003 hemolysin D	10	3	5	7	7	2	5	0	67	67	74	89
K11891 type VI secretion system protein ImpL	19635	19375	18382	19132	11317	11006	10706	11572	9536	9422	8474	9226
K11892 type VI secretion system protein ImpK	4963	4873	4359	4684	2868	2840	2705	2922	2267	2182	2170	2241
K11903 type VI secretion system secreted protein Hcp	2669	2663	2558	2535	1564	1617	1492	1616	1418	1468	1323	1419
K11904 type VI secretion system secreted protein VgrG	28524	28444	27959	28775	16304	16426	14901	15661	11992	11726	11627	11929
K11906 type VI secretion system protein VasD	2051	1977	1832	1845	1161	1057	1108	1107	1175	1162	1092	1136
K11907 type VI secretion system protein VasG	13354	13568	12620	12864	8095	8383	7688	8190	5367	5446	5303	5438
K11912 serine/threonine-protein kinase PpkA [EC:2.7.11.1]	3564	3692	3156	3223	1850	1750	1621	1957	1225	982	994	1128
K11913 type VI secretion system protein	1861	1894	1566	1652	963	907	976	1114	684	549	567	584
K11915 serine/threonine protein phosphatase Stp1 [EC:3.1.3.16]	828	852	773	748	397	420	389	540	292	276	273	242
K12340 outer membrane protein	6053	6403	6047	6105	7792	7869	8112	8336	6229	6003	6091	6137
Signal transduction	635989	629723	616992	628286	464972	468313	454285	465392	457522	459896	452900	462323
ko02020 Two-component system	573175	567064	554878	565370	405596	407925	395160	406201	400438	402988	396030	404778
K00373 nitrate reductase molybdenum cofactor assembly chaperone NarJ/NarW	1203	1197	1214	1246	660	628	604	620	1336	1298	1270	1340
K00575 chemotaxis protein methyltransferase CheR [EC:2.1.1.80]	1007	1058	968	984	586	627	611	628	727	828	770	729
K00990 [protein-PII] uridylyltransferase [EC:2.7.7.59]	5846	5799	5887	5891	3794	3826	3585	3552	3709	3822	3730	3876
K01104 protein-tyrosine phosphatase [EC:3.1.3.48]	1739	1781	1749	1683	1825	1849	1788	1744	1863	1914	1870	1823
K01467 beta-lactamase class C [EC:3.5.2.6]	4	12	4	1	9	13	18	16	59	78	72	61
K01545 potassium-transporting ATPase KdpF subunit	255	269	257	214	154	148	143	161	83	84	58	91
K01546 potassium-transporting ATPase potassium-binding subunit	4681	4743	4778	4638	5447	5568	5556	5511	5831	5942	6142	5851
K01547 potassium-transporting ATPase ATP-binding subunit [EC:7.2.2.6]	6384	6442	6277	6173	6839	6599	6799	6774	6674	6701	6769	6628
K01548 potassium-transporting ATPase KdpC subunit	1639	1681	1463	1622	1817	1718	1662	1760	1750	1630	1806	1682
K01643 citrate lyase subunit alpha / citrate CoA-transferase [EC:2.8.3.10]	2215	2057	2018	2201	1587	1522	1457	1590	3000	3135	2889	3147
K01644 citrate lyase subunit beta / citryl-CoA lyase [EC:4.1.3.34]	1279	1204	1137	1232	829	838	913	788	1679	1853	1660	1728
K01646 citrate lyase subunit gamma (acyl carrier protein)	393	428	424	388	262	310	277	319	580	576	553	564
K01910 [citrate (pro-3S)-lyase] ligase [EC:6.2.1.22]	1325	1377	1404	1461	953	1064	1005	954	2005	2097	1897	1992
K01991 polysaccharide biosynthesis/export protein	1639	1737	1927	1739	3526	3493	3633	3528	2772	2741	2642	2675
K02106 short-chain fatty acids transporter	1166	1190	1162	1158	665	611	595	673	538	506	519	507
K02253 competence protein ComX	0	0	0	2	0	0	0	0	0	0	0	0
K02313 chromosomal replication initiator protein	5654	5570	5696	5718	5497	5710	5770	5762	5510	5350	5596	5472
K02398 negative regulator of flagellin synthesis FlgM	348	379	371	385	278	285	377	359	146	176	140	178
K02402 flagellar transcriptional activator FlhC	119	90	89	124	138	179	206	180	38	27	34	27
K02403 flagellar transcriptional activator FlhD	137	124	112	99	180	188	156	174	27	25	12	26
K02405 RNA polymerase sigma factor for flagellar operon FliA	1624	1606	1622	1620	901	935	860	944	761	865	774	775
K02406 flagellin	1133	1080	1101	1137	1042	1085	1172	1120	509	615	505	519
K02487 type IV pili sensor histidine kinase and response regulator	1425	1259	1392	1360	1160	1186	1595	1706	477	433	411	453
K02488 two-component system, cell cycle response regulator [EC:2.7.7.65]	42	40	37	39	8	4	7	9	82	105	79	74
K02490 two-component system, response regulator, stage 0 sporulation protein F	6	2	15	8	0	2	0	0	4	2	7	4
K02491 two-component system, sporulation sensor kinase A [EC:2.7.13.3]	0	0	0	0	0	0	0	0	0	0	0	1
K02556 chemotaxis protein MotA	2296	2160	2140	2224	1195	1216	1153	1194	1178	1188	1087	1092
K02584 Nif-specific regulatory protein	373	365	307	366	372	452	437	457	54	50	51	80
K02650 type IV pilus assembly protein PilA	932	891	868	905	517	470	518	461	325	299	348	302
K02657 twitching motility two-component system response regulator PilG	745	722	740	703	446	395	378	449	266	221	200	240
K02658 twitching motility two-component system response regulator PilH	614	617	568	623	321	362	336	334	198	154	169	205
K02659 twitching motility protein PilI	721	707	689	700	379	446	384	400	257	212	234	196
K02660 twitching motility protein PilJ	3060	3093	2998	3096	1656	1807	1474	1645	1002	1002	976	954
K02661 type IV pilus assembly protein PilK	1620	1526	1447	1448	834	773	805	926	523	436	446	507
K02667 two-component system, NtrC family, response regulator PilR	941	996	981	1020	564	554	538	629	340	323	318	316
K02668 two-component system, NtrC family, sensor histidine kinase PilS [EC:2.7.13.3]	2767	2633	2772	2693	1542	1491	1446	1438	924	813	797	829
K03092 RNA polymerase sigma-54 factor	3637	3592	3689	3611	5026	5041	4837	4827	4527	4342	4434	4297
K03406 methyl-accepting chemotaxis protein	47131	45884	44271	45269	28990	29267	25343	26712	15030	14579	14353	14700
K03407 two-component system, chemotaxis family, sensor kinase CheA [EC:2.7.13.3]	6775	6826	6232	6213	3662	3508	3228	3569	2918	2742	2763	2873
K03408 purine-binding chemotaxis protein CheW	2815	2847	2804	2757	1585	1549	1458	1721	1115	1048	1061	1007
K03412 two-component system, chemotaxis family, protein-glutamate methylesterase/glutaminase [EC:3.1.1.61 3.5.1.44]	2468	2366	2322	2355	1454	1424	1550	1464	970	899	886	913
K03413 two-component system, chemotaxis family, chemotaxis protein CheY	1552	1554	1537	1537	860	859	805	849	744	738	707	858
K03415 two-component system, chemotaxis family, chemotaxis protein CheV	1321	1335	1327	1396	863	926	937	859	335	426	378	383
K03563 carbon storage regulator	466	545	514	534	239	256	232	216	421	405	389	428
K03620 Ni/Fe-hydrogenase 1 B-type cytochrome subunit	42	48	65	58	63	66	66	50	105	112	114	131

K03739 membrane protein involved in D-alanine export	364	304	369	354	408	410	347	319	184	229	187	203
K03740 D-alanine transfer protein	345	389	353	405	382	404	407	345	141	219	234	224
K03776 aerotaxis receptor	2704	2651	2717	2803	2201	2371	2140	2310	794	716	657	739
K04751 nitrogen regulatory protein P-II 1	654	688	678	665	976	906	933	907	980	1073	1034	1059
K04771 serine protease Do [EC:3.4.21.107]	4021	3942	3976	4213	2224	2042	2155	2320	2934	3065	2982	3131
K05338 holin-like protein	37	40	29	20	3	2	0	3	18	25	29	23
K05339 holin-like protein LrgB	32	33	55	37	0	9	5	5	30	57	25	58
K05874 methyl-accepting chemotaxis protein I, serine sensor receptor	1054	1089	915	993	1439	1589	1398	1397	79	92	92	85
K05875 methyl-accepting chemotaxis protein II, aspartate sensor receptor	1214	1234	1185	1240	1825	1826	1731	1536	88	72	77	74
K05876 methyl-accepting chemotaxis protein III, ribose and galactose sensor receptor	286	312	294	336	419	454	434	447	11	15	10	12
K05877 methyl-accepting chemotaxis protein IV, peptide sensor receptor	263	301	373	308	427	471	371	332	11	12	18	17
K05964 holo-ACP synthase [EC:2.7.7.61]	506	484	479	513	375	439	426	440	537	571	557	571
K05966 triphosphoribosyl-dephospho-CoA synthase [EC:2.4.2.52]	1206	1138	1056	1147	753	785	756	706	1436	1644	1596	1640
K06080 RcsF protein	236	246	209	219	163	208	132	160	299	369	308	364
K06596 chemosensory pili system protein ChpA (sensor histidine kinase/response regulator)	10787	10711	9807	10448	6017	5761	5568	6414	3468	3436	3240	3338
K06597 chemosensory pili system protein ChpB (putative protein-glutamate methylesterase)	1682	1589	1510	1602	838	887	864	956	596	519	447	488
K06598 chemosensory pili system protein ChpC	789	724	709	679	402	381	379	452	247	296	248	224
K07165 transmembrane sensor	11201	11026	10136	10621	5792	5595	5582	6171	3509	3182	3131	3203
K07636 two-component system, OmpR family, phosphate regulon sensor histidine kinase PhoR [EC:2.7.13.3]	5436	5371	5230	5269	6181	6321	6253	6123	5350	5438	5254	5301
K07637 two-component system, OmpR family, sensor histidine kinase PhoQ [EC:2.7.13.3]	2514	2553	2418	2480	1419	1538	1360	1547	1580	1721	1630	1704
K07638 two-component system, OmpR family, osmolarity sensor histidine kinase EnvZ [EC:2.7.13.3]	2667	2564	2445	2536	1717	1698	1529	1637	1828	1792	1846	1845
K07639 two-component system, OmpR family, sensor histidine kinase RstB [EC:2.7.13.3]	3005	2905	2744	3013	1633	1711	1578	1754	1631	1778	1606	1757
K07640 two-component system, OmpR family, sensor histidine kinase CpxA [EC:2.7.13.3]	902	901	888	945	663	674	693	671	1254	1220	1253	1305
K07641 two-component system, OmpR family, sensor histidine kinase CreC [EC:2.7.13.3]	1888	1911	1951	1895	1237	1190	1213	1188	1460	1498	1346	1578
K07642 two-component system, OmpR family, sensor histidine kinase BaeS [EC:2.7.13.3]	835	758	756	792	533	573	572	575	1122	1184	1100	1188
K07643 two-component system, OmpR family, sensor histidine kinase BasS [EC:2.7.13.3]	605	604	605	703	436	417	459	427	838	953	881	896
K07644 two-component system, OmpR family, heavy metal sensor histidine kinase CusS [EC:2.7.13.3]	12082	11941	11475	11882	7896	8077	7446	7698	4518	4263	4286	4445
K07645 two-component system, OmpR family, sensor histidine kinase QseC [EC:2.7.13.3]	2904	2923	2697	2902	1497	1433	1386	1504	1753	1837	1796	1958
K07646 two-component system, OmpR family, sensor histidine kinase KdpD [EC:2.7.13.3]	7688	7460	7132	7380	5527	5448	5583	5753	7249	7325	7202	7356
K07647 two-component system, OmpR family, sensor histidine kinase TorS [EC:2.7.13.3]	7	11	10	14	20	19	29	25	130	194	179	166
K07648 two-component system, OmpR family, aerobic respiration control sensor histidine kinase ArcB [EC:2.7.13.3]	1322	1290	1266	1369	971	1050	999	1015	1749	1674	1689	1710
K07649 two-component system, OmpR family, sensor histidine kinase TctE [EC:2.7.13.3]	2258	2167	2102	2130	1163	1132	1177	1208	704	724	609	756
K07650 two-component system, OmpR family, sensor histidine kinase CssS [EC:2.7.13.3]	0	0	2	1	2	2	0	0	4	3	7	7
K07651 two-component system, OmpR family, sensor histidine kinase ResE [EC:2.7.13.3]	6	9	12	11	2	3	3	1	12	20	22	25
K07652 two-component system, OmpR family, sensor histidine kinase VicK [EC:2.7.13.3]	185	167	173	175	140	136	140	159	242	259	228	243
K07653 two-component system, OmpR family, sensor histidine kinase MprB [EC:2.7.13.3]	0	3	4	2	0	0	2	0	1	6	2	2
K07654 two-component system, OmpR family, sensor histidine kinase MtrB [EC:2.7.13.3]	8	4	12	8	3	3	1	3	17	17	14	24
K07656 two-component system, OmpR family, sensor histidine kinase TrcS [EC:2.7.13.3]	0	0	0	0	0	0	0	0	1	0	0	0
K07657 two-component system, OmpR family, phosphate regulon response regulator PhoB	1050	1044	1057	1050	749	682	685	679	881	839	759	866
K07658 two-component system, OmpR family, alkaline phosphatase synthesis response regulator PhoP	525	429	484	514	107	192	157	150	514	673	648	616
K07659 two-component system, OmpR family, phosphate regulon response regulator OmpR	1247	1320	1190	1257	863	874	850	773	1042	1025	1054	1046
K07660 two-component system, OmpR family, response regulator PhoP	1474	1545	1396	1494	796	825	696	771	1257	1375	1361	1422
K07661 two-component system, OmpR family, response regulator RstA	1445	1473	1382	1481	858	836	803	831	881	869	811	845
K07662 two-component system, OmpR family, response regulator CpxR	449	411	444	421	316	340	369	330	665	678	653	617
K07663 two-component system, OmpR family, catabolic regulation response regulator CreB	1541	1494	1464	1485	916	910	918	849	930	864	891	913
K07664 two-component system, OmpR family, response regulator BaeR	407	415	365	404	262	255	241	258	584	601	561	552
K07665 two-component system, OmpR family, copper resistance phosphate regulon response regulator CusR	5456	5225	5081	5339	3454	3499	3212	3447	1971	1897	1874	1842
K07666 two-component system, OmpR family, response regulator QseB	1077	1127	1131	1095	667	696	725	683	913	911	931	918
K07667 two-component system, OmpR family, KDP operon response regulator KdpE	2347	2055	2213	2222	899	922	879	930	2092	2061	2058	2121
K07668 two-component system, OmpR family, response regulator VicR	101	129	136	124	60	74	76	78	140	148	148	157
K07669 two-component system, OmpR family, response regulator MprA	13	8	10	13	9	21	7	16	12	12	19	11
K07670 two-component system, OmpR family, response regulator MtrA	9	9	11	2	5	5	1	7	12	10	10	17
K07671 two-component system, OmpR family, response regulator PrrA	0	0	0	2	0	1	2	0	1	0	2	3
K07672 two-component system, OmpR family, response regulator TrcR	0	0	0	3	0	0	0	0	3	3	4	1
K07673 two-component system, NarL family, nitrate/nitrite sensor histidine kinase NarX [EC:2.7.13.3]	2305	2392	2075	2283	1526	1579	1404	1505	1972	1898	1819	1876
K07674 two-component system, NarL family, nitrate/nitrite sensor histidine kinase NarQ [EC:2.7.13.3]	344	346	371	385	557	592	548	473	125	104	95	109
K07675 two-component system, NarL family, sensor histidine kinase UhpB [EC:2.7.13.3]	592	621	554	625	209	172	174	163	1355	1285	1279	1314
K07676 two-component system, NarL family, sensor histidine kinase RcsD [EC:2.7.13.3]	1423	1589	1433	1521	1018	1012	1031	1050	1930	2055	1973	2023
K07677 two-component system, NarL family, capsular synthesis sensor histidine kinase RcsC [EC:2.7.13.3]	1648	1697	1558	1743	1083	1242	1206	1052	2299	2465	2226	2482
K07678 two-component system, NarL family, sensor histidine kinase BarA [EC:2.7.13.3]	5252	5278	4976	5261	3053	3115	3206	3375	3477	3402	3428	3438
K07679 two-component system, NarL family, sensor histidine kinase EvgS [EC:2.7.13.3]	10903	10843	10873	10764	5697	6016	5516	5433	5573	5689	5671	5932
K07680 two-component system, NarL family, sensor histidine kinase ComP [EC:2.7.13.3]	2	9	2	1	0	0	0	0	0	0	0	0
K07681 two-component system, NarL family, vancomycin resistance sensor histidine kinase VraS [EC:2.7.13.3]	0	0	0	0	0	0	0	1	0	0	0	0
K07683 two-component system, NarL family, sensor histidine kinase NreB [EC:2.7.13.3]	0	0	0	0	0	1	2	1	0	3	3	0
K07684 two-component system, NarL family, nitrate/nitrite response regulator NarL	1199	1132	1206	1197	736	774	790	916	828	809	764	753
K07685 two-component system, NarL family, nitrate/nitrite response regulator NarP	2	3	5	1	10	9	6	6	42	72	57	36
K07686 two-component system, NarL family, uhpT operon response regulator UhpA	867	871	764	872	438	370	411	409	568	653	645	631
K07687 two-component system, NarL family, captular synthesis response regulator RcsB	2525	2576	2617	2549	1445	1567	1465	1414	1278	1246	1185	1147
K07688 two-component system, NarL family, response regulator, fimbrial Z protein, FimZ	124	133	128	145	195	196	224	204	0	1	0	1
K07689 two-component system, NarL family, invasion response regulator UvrY	465	476	443	500	317	374	297	333	522	621	534	651
K07690 two-component system, NarL family, response regulator EvgA	2519	2453	2279	2346	1342	1424	1295	1313	1175	1105	1067	1108
K07691 two-component system, NarL family, competent response regulator ComA	1	0	0	3	0	0	0	0	0	0	0	0
K07692 two-component system, NarL family, response regulator DegU	1	0	0	0	0	0	0	0	0	0	0	0
K07693 two-component system, NarL family, response regulator DesR	6	2	9	4	0	2	2	2	11	7	8	4
K07694 two-component system, NarL family, vancomycin resistance associated response regulator VraR	2	2	0	5	2	3	1	3	0	0	0	0
K07696 two-component system, NarL family, response regulator NreC	3	6	2	6	3	5	3	7	10	4	2	6
K07698 two-component system, sporulation sensor kinase C [EC:2.7.13.3]	0	1	0	1	0	0	0	0	5	0	1	1
K07699 two-component system, response regulator, stage 0 sporulation protein A	490	538	539	552	184	194	187	211	444	541	430	458
K07700 two-component system, CitB family, cit operon sensor histidine kinase CitA [EC:2.7.13.3]	1632	1609	1461	1707	854	936	922	829	2755	3000	2601	2803
K07701 two-component system, CitB family, sensor histidine kinase DcuS [EC:2.7.13.3]	907	960	859	913	628	690	628	630	1296	1417	1205	1347
K07702 two-component system, CitB family, response regulator CitB	706	747	752	689	341	417	411	403	1129	1232	1214	1217
K07703 two-component system, CitB family, response regulator DcuR	392	364	384	393	232	273	268	312	518	571	520	587
K07704 two-component system, LytTR family, sensor histidine kinase LytS [EC:2.7.13.3]	279	287	287	276	86	127	127	95	334	409	359	379
K07705 two-component system, LytTR family, response regulator LytT	96	129	113	104	40	48	47	43	94	115	87	99
K07706 two-component system, LytTR family, sensor histidine kinase AgrC [EC:2.7.13.3]	383	381	359	419	92	98	64	79	556	549	563	536
K07707 two-component system, LytTR family, response regulator AgrA	145	159	176	162	61	57	70	44	200	243	223	218
K07708 two-component system, NtrC family, nitrogen regulation sensor histidine kinase GlnL [EC:2.7.13.3]	2067	1994	1938	2199	1347	1341	1230	1308	1372	1419	1375	1527
K07709 two-component system, NtrC family, sensor histidine kinase HydH [EC:2.7.13.3]	859	903	848	904	594	723	623	639	1271	1179	1151	1246
K07710 two-component system, NtrC family, sensor histidine kinase AtoS [EC:2.7.13.3]	5	5	12	4	19	27	45	16	107	119	102	116
K07711 two-component system, NtrC family, sensor histidine kinase GlrK [EC:2.7.13.3]	846	793	754	867	507	600	485	496	1214	1223	1115	1248
K07712 two-component system, NtrC family, nitrogen regulation response regulator GlnG	3562	3564	3384	3535	2330	2121	2127	2194	2080	2148	2170	2196
K07713 two-component system, NtrC family, response regulator HydG	3724	3810	4020	3871	9125	9085	9121	9160	7183	6957	7422	6929
K07714 two-component system, NtrC family, response regulator AtoC	37	24	47	32	42	28	56	32	103	103	98	92
K07715 two-component system, NtrC family, response regulator GlrR	798	753	745	771	538	629	619	570	1135	1146	1099	1201
K07716 two-component system, cell cycle sensor histidine kinase PleC [EC:2.7.13.3]	4	2	3	3	3	6	6	6	6	9	4	7
K07717 two-component system, sensor histidine kinase YcbA [EC:2.7.13.3]	15	12	12	25	5	8	13	7	10	13	3	7
K07718 two-component system, sensor histidine kinase YesM [EC:2.7.13.3]	14983	14467	15217	15053	2519	2695	2646	2313	16237	17870	17006	17327
K07719 two-component system, response regulator YcbB	48	34	34	36	14	13	12	11	32	27	28	14
K07720 two-component system, response regulator YesN	14237	13420	14020	13981	2689	2729	2700	2582	14228	15383	15143	15436
K07768 two-component system, OmpR family, sensor histidine kinase SenX3 [EC:2.7.13.3]	4	5	5	1	7	6	5	10	11	8	10	15
K07770 two-component system, OmpR family, response regulator CssR	1	1	0	0	3	3	0	0	2	5	2	2
K07771 two-component system, OmpR family, response regulator BasR	351	372	375	411	268	309	289	242	578	510	565	565
K07772 two-component system, OmpR family, torCAD operon response regulator TorR	1	1	2	1	8	7	8	1	28	25	33	33
K07773 two-component system, OmpR family, aerobic respiration control protein ArcA	340	323	368	332	250	252	248	242	387	454	386	444
K07774 two-component system, OmpR family, response regulator TctD	1185	1033	1016	1020	589	520	554	613	323	297	325	330
K07775 two-component system, OmpR family, response regulator ResD	11	8	10	7	8	5	2	9	13	26	19	17
K07776 two-component system, OmpR family, response regulator RegX3	6	9	16	8	14	12	8	16	18	31	25	18
K07777 two-component system, NarL family, sensor histidine kinase DegS [EC:2.7.13.3]	0	0	2	1	0	0	0	0	0	8	7	3
K07778 two-component system, NarL family, sensor histidine kinase DesK [EC:2.7.13.3]	0	2	0	3	0	2	0	0	0	0	0	1
K07781 LuxR family transcriptional regulator, capsular biosynthesis positive transcription factor	307	303	306	299	247	241	225	214	347	356	378	428
K07782 LuxR family transcriptional regulator, quorum-sensing system regulator SdiA	424	382	382	438	284	300	323	273	582	640	640	596
K07783 MFS transporter, OPA family, sugar phosphate sensor protein UhpC	2754	3130	2828	2978	3624	3411	3594	3499	4205	4173	4142	4237
K07784 MFS transporter, OPA family, hexose phosphate transport protein UhpT	598	650	562	675	150	239	206	188	1277	1309	1252	1292
K07786 MFS transporter, DHA2 family, multidrug resistance protein	3	2	7	7	15	11	17	25	75	87	84	87
K07787 Cu(I)/Ag(I) efflux system membrane protein CusA/SilA	8227	7974	8049	7961	6558	6830	6459	6582	4323	4324	4314	4326
K07788 multidrug efflux pump	5708	5639	5393	5541	3189	3289	3174	3379	3614	3888	3578	3835
K07789 multidrug efflux pump	6155	5998	5731	5962	3426	3508	3372	3553	3977	3756	3690	3863
K07790 putative membrane protein PagO	0	10	14	0	7	0	0	0	0	0	8	0
K07792 anaerobic C4-dicarboxylate transporter DcuB	1017	974	987	1079	694	812	757	799	1486	1534	1493	1580
K07793 putative tricarboxylic transport membrane protein	2971	2776	2893	2822	1644	1611	1339	1560	1160	1117	1097	1237
K07794 putative tricarboxylic transport membrane protein	593	511	638	583	310	307	295	308	210	184	173	192

K07795 putative tricarboxylic transport membrane protein	1498	1504	1424	1436	824	762	802	808	436	415	431	463
K07796 outer membrane protein, Cu(I)/Ag(I) efflux system	1435	1442	1375	1519	1455	1506	1411	1363	1331	1448	1352	1512
K07797 multidrug resistance protein K	7	1	0	7	10	14	10	10	68	57	69	58
K07798 membrane fusion protein, Cu(I)/Ag(I) efflux system	3645	3550	3730	3697	2795	2840	2895	2727	1638	1839	1722	1895
K07799 membrane fusion protein, multidrug efflux system	2191	2156	1998	2157	1314	1365	1339	1338	1409	1333	1279	1409
K07800 AgrD protein	7	6	12	9	4	3	3	2	4	9	10	5
K07803 zinc resistance-associated protein	263	314	301	257	185	225	202	181	434	404	374	421
K07810 Cu(I)/Ag(I) efflux system periplasmic protein CusF	301	326	317	339	336	332	283	295	300	365	314	334
K07813 accessory gene regulator B	200	213	218	209	98	90	97	76	166	192	177	214
K08082 two-component system, LytTR family, sensor histidine kinase AlgZ [EC:2.7.13.3]	1786	1765	1642	1757	962	919	925	1035	582	559	574	565
K08083 two-component system, LytTR family, response regulator AlgR	1333	1430	1347	1260	699	665	668	793	400	425	389	417
K08348 formate dehydrogenase-N, alpha subunit [EC:1.17.5.3]	2136	2146	2030	2134	1839	1922	1813	1865	2255	2343	2294	2364
K08349 formate dehydrogenase-N, beta subunit	674	709	667	712	719	685	575	649	746	747	807	788
K08350 formate dehydrogenase-N, gamma subunit	436	435	451	473	363	415	423	420	447	494	472	534
K08372 putative serine protease PepD [EC:3.4.21.-]	12	7	13	13	5	13	5	16	16	24	26	29
K08475 two-component system, NtrC family, phosphoglycerate transport system sensor histidine kinase PgtB [EC:2.7.13.3]	698	667	692	736	168	170	177	155	1364	1409	1362	1498
K08476 two-component system, NtrC family, phosphoglycerate transport system response regulator PgtA	479	447	433	437	107	129	129	133	975	900	912	1006
K08478 phosphoglycerate transport regulatory protein PgtC	381	466	431	430	132	108	146	112	883	874	992	951
K08641 zinc D-Ala-D-Ala dipeptidase [EC:3.4.13.22]	1905	1835	1716	1711	2501	2550	2426	2551	1714	1596	1754	1630
K08738 cytochrome c	536	445	470	493	285	267	263	266	206	158	146	142
K09475 outer membrane pore protein C	629	577	622	634	429	474	458	385	856	938	853	866
K09476 outer membrane pore protein F	794	832	785	825	668	798	790	655	871	921	848	861
K09477 citrate:succinate antiporter	16	21	18	18	5	16	10	23	64	73	69	82
K10125 two-component system, NtrC family, C4-dicarboxylate transport sensor histidine kinase DctB [EC:2.7.13.3]	2971	2775	2769	2925	1530	1610	1598	1635	1117	1038	973	1122
K10126 two-component system, NtrC family, C4-dicarboxylate transport response regulator DctD	4408	4410	4166	4315	2438	2340	2291	2415	1509	1399	1404	1417
K10697 two-component system, OmpR family, response regulator RpaA	3	2	1	1	0	0	0	0	2	2	2	4
K10715 two-component system, sensor histidine kinase RpfC [EC:2.7.13.3]	2	0	1	1	0	1	1	0	1	0	0	1
K10851 nitrogen regulatory protein A	0	0	0	0	0	1	0	2	0	0	0	0
K10909 two-component system, autoinducer 2 sensor kinase/phosphatase LuxQ [EC:2.7.13.3 3.1.3.-]	0	0	0	0	3	2	3	2	1	0	3	1
K10914 CRP/FNR family transcriptional regulator, cyclic AMP receptor protein	2190	2285	2142	2133	1190	1085	1039	1169	1181	1318	1193	1221
K10941 sigma-54 dependent transcriptional regulator, flagellar regulatory protein	1956	1955	1942	1910	1072	1122	987	1125	619	613	600	573
K10942 two-component system, sensor histidine kinase FlrB [EC:2.7.13.3]	1791	1805	1776	1728	924	900	741	849	518	512	532	533
K10943 two-component system, response regulator FlrC	2189	2121	2109	2065	1103	1097	1031	1139	757	636	676	663
K11103 aerobic C4-dicarboxylate transport protein	3967	4015	4054	3946	2380	2399	2250	2587	2224	2270	2113	2229
K11230 mitogen-activated protein kinase kinase kinase [EC:2.7.11.25]	0	0	0	0	0	0	0	0	0	0	0	1
K11231 osomolarity two-component system, sensor histidine kinase SLN1 [EC:2.7.13.3]	1	0	0	0	1	0	0	0	0	0	0	0
K11329 two-component system, OmpR family, response regulator RpaB	1	2	0	0	0	2	1	0	4	2	0	1
K11354 two-component system, chemotaxis family, sensor kinase Cph1 [EC:2.7.13.3]	0	0	0	1	0	0	0	0	3	0	0	0
K11356 two-component system, sensor histidine kinase and response regulator [EC:2.7.13.3]	1	1	2	0	1	0	1	1	1	1	1	1
K11357 two-component system, cell cycle sensor histidine kinase DivJ [EC:2.7.13.3]	2	3	3	3	2	2	4	1	7	3	5	3
K11382 MFS transporter, OPA family, phosphoglycerate transporter protein	417	459	491	443	84	142	115	108	1030	1040	957	974
K11383 two-component system, NtrC family, sensor histidine kinase KinB [EC:2.7.13.3]	2018	1866	1847	1951	1121	1099	1035	1035	601	570	631	649
K11384 two-component system, NtrC family, response regulator AlgB	2262	2308	2330	2359	1280	1221	1265	1418	696	749	656	731
K11443 two-component system, cell cycle response regulator DivK	0	2	0	0	0	0	0	0	0	0	0	0
K11444 two-component system, chemotaxis family, response regulator WspR [EC:2.7.7.65]	121	119	118	109	72	81	194	235	38	44	44	42
K11520 two-component system, OmpR family, manganese sensing sensor histidine kinase [EC:2.7.13.3]	0	1	0	1	0	0	1	0	0	0	0	1
K11521 two-component system, OmpR family, manganese sensing response regulator	0	1	2	3	1	1	1	0	4	0	3	1
K11525 methyl-accepting chemotaxis protein PixJ	0	1	1	0	0	0	0	0	0	0	0	0
K11614 two-component system, CitB family, sensor histidine kinase MalK [EC:2.7.13.3]	63	58	75	58	22	20	27	22	111	151	132	125
K11615 two-component system, CitB family, response regulator MalR	54	50	47	62	14	20	10	21	75	86	102	59
K11616 malate:Na+ symporter	68	55	69	68	63	95	85	86	100	97	85	102
K11617 two-component system, NarL family, sensor histidine kinase LiaS [EC:2.7.13.3]	93	66	86	73	24	16	13	24	66	96	80	100
K11618 two-component system, NarL family, response regulator LiaR	44	37	43	39	8	8	9	17	30	55	41	60
K11624 two-component system, NarL family, response regulator YdfI	6	5	3	3	3	0	0	0	6	10	9	13
K11626 putative sodium/glutamine symporter	1	1	2	1	4	5	2	3	3	3	1	2
K11629 two-component system, OmpR family, bacitracin resistance sensor histidine kinase BceS [EC:2.7.13.3]	2	2	0	1	0	0	0	0	0	0	0	0
K11630 two-component system, OmpR family, bacitracin resistance response regulator BceR	3	0	4	1	2	3	0	2	2	3	1	1
K11635 putative ABC transport system ATP-binding protein	42	57	41	33	3	21	9	3	35	50	36	49
K11636 putative ABC transport system permease protein	91	94	79	110	8	11	11	25	104	99	64	83
K11637 two-component system, CitB family, sensor histidine kinase CitS [EC:2.7.13.3]	0	2	0	3	0	1	1	0	1	0	1	2
K11688 C4-dicarboxylate-binding protein DctP	5009	5004	4905	4852	2796	2910	2662	2851	1473	1631	1505	1573
K11689 C4-dicarboxylate transporter, DctQ subunit	2012	1980	1926	1987	1166	1106	988	1096	695	685	733	713
K11690 C4-dicarboxylate transporter, DctM subunit	3764	3766	3629	3689	2067	1970	1887	2018	1633	1759	1662	1698
K11691 two-component system, CitB family, sensor histidine kinase DctS [EC:2.7.13.3]	0	2	2	3	0	0	0	1	0	2	0	1
K11692 two-component system, CitB family, response regulator DctR	8	4	10	5	40	33	37	38	13	17	5	12
K11711 two-component system, LuxR family, sensor histidine kinase DctS [EC:2.7.13.3]	4	6	2	6	10	5	8	14	10	4	6	10
K11712 two-component system, LuxR family, response regulator DctR	4	3	7	3	6	5	11	2	5	8	7	6
K12531 ATP-binding cassette, subfamily B, bacterial RtxE	6	9	10	7	4	4	7	5	5	3	1	1
K13040 two-component system, LuxR family, sensor histidine kinase TtrS [EC:2.7.13.3]	190	202	202	195	316	374	358	369	353	320	304	278
K13041 two-component system, LuxR family, response regulator TtrR	12	17	8	12	16	18	21	25	30	20	17	17
K13486 chemotaxis protein methyltransferase WspC	1918	1782	1700	1699	1024	957	1083	1068	588	608	558	563
K13487 methyl-accepting chemotaxis protein WspA	2047	2076	2052	2078	1121	1108	879	948	673	615	609	665
K13488 chemotaxis-related protein WspB	774	731	755	784	384	377	337	417	264	212	200	230
K13489 chemotaxis-related protein WspD	152	135	129	152	80	81	64	101	52	44	45	44
K13490 two-component system, chemotaxis family, sensor histidine kinase and response regulator WspE	3201	3067	2965	3064	1621	1576	1554	1858	886	848	934	911
K13491 two-component system, chemotaxis family, response regulator WspF [EC:3.1.1.61]	1658	1646	1582	1636	929	860	850	955	553	481	448	514
K13532 two-component system, sporulation sensor kinase D [EC:2.7.13.3]	0	0	0	0	0	1	0	0	0	0	0	0
K13533 two-component system, sporulation sensor kinase E [EC:2.7.13.3]	1	0	0	3	0	0	0	0	0	0	0	0
K13584 two-component system, cell cycle response regulator CtrA	0	0	3	1	0	4	0	1	4	2	0	1
K13587 two-component system, cell cycle sensor histidine kinase and response regulator CckA [EC:2.7.13.3]	2	4	0	1	0	0	0	2	6	8	3	3
K13588 histidine phosphotransferase ChpT	0	0	0	1	0	0	0	0	2	0	1	1
K13589 two-component system, cell cycle response regulator CpdR	0	0	0	1	0	0	1	0	0	0	0	1
K13598 two-component system, NtrC family, nitrogen regulation sensor histidine kinase NtrY [EC:2.7.13.3]	13	12	5	7	5	2	3	4	7	4	3	7
K13599 two-component system, NtrC family, nitrogen regulation response regulator NtrX	25	12	17	10	8	9	13	11	5	10	8	10
K13815 two-component system, response regulator RpfG	0	0	0	1	0	0	0	0	0	0	0	0
K13816 DSF synthase	175	160	180	169	243	236	221	220	0	2	0	0
K13924 two-component system, chemotaxis family, CheB/CheR fusion protein [EC:2.1.1.80 3.1.1.61]	112	89	97	76	14	6	13	9	84	69	61	63
K13927 holo-ACP synthase / triphosphoribosyl-dephospho-CoA synthase [EC:2.7.7.61 2.4.2.52]	126	112	130	112	27	25	13	7	144	153	136	152
K14205 phosphatidylglycerol lysyltransferase [EC:2.3.2.3]	4315	4186	4250	4077	2349	2292	2040	2243	1571	1581	1494	1675
K14978 two-component system, LuxR family, secretion system sensor histidine kinase SsrA	1	0	0	1	0	0	0	2	0	0	0	0
K14979 two-component system, LuxR family, secretion system response regulator SsrB	1	0	0	0	0	1	0	0	0	0	0	0
K14980 two-component system, OmpR family, sensor histidine kinase ChvG [EC:2.7.13.3]	3	5	3	3	1	0	0	3	6	3	1	8
K14981 two-component system, OmpR family, response regulator ChvI	3	1	1	1	0	0	1	2	1	0	2	0
K14982 two-component system, OmpR family, sensor histidine kinase CiaH [EC:2.7.13.3]	8	14	10	9	18	25	13	16	37	20	24	27
K14983 two-component system, OmpR family, response regulator CiaR	8	5	7	13	8	4	17	13	16	30	13	36
K14986 two-component system, LuxR family, sensor kinase FixL [EC:2.7.13.3]	3	1	1	1	1	0	0	0	3	1	0	3
K14987 two-component system, LuxR family, response regulator FixJ	13	14	11	13	27	16	17	23	30	23	17	19
K14988 two-component system, NarL family, secretion system sensor histidine kinase SalK	18	29	12	17	37	24	20	14	40	36	44	36
K14989 two-component system, NarL family, secretion system response regulator SalR	21	9	11	10	5	8	17	9	26	19	12	46
K15011 two-component system, sensor histidine kinase RegB [EC:2.7.13.3]	816	794	777	872	456	412	406	471	262	285	259	297
K15012 two-component system, response regulator RegA	805	777	754	782	435	406	355	398	245	226	209	210
K15739 D-alanine---(R)-lactate ligase [EC:6.1.2.1]	0	0	0	0	0	0	2	0	0	0	0	1
K15861 CRP/FNR family transcriptional regulator, nitrogen fixation regulation protein	0	2	1	0	0	0	0	0	0	0	0	0
K16692 tyrosine-protein kinase Etk/Wzc [EC:2.7.10.-]	572	579	646	669	755	816	679	723	537	611	591	613
K17060 two-component system, sensor histidine kinase AauS [EC:2.7.13.3]	430	403	392	431	228	235	162	177	146	114	115	103
K17061 two-component system, response regulator AauR	2081	2060	1921	1933	1056	950	1074	1117	538	548	655	601
K18072 two-component system, OmpR family, sensor kinase ParS [EC:2.7.13.3]	23	26	41	35	24	17	45	66	10	9	13	8
K18073 two-component system, OmpR family, response regulator ParR	381	392	437	393	209	261	207	229	114	127	117	115
K18093 imipenem/basic amino acid-specific outer membrane pore [EC:3.4.21.-]	1774	1832	1813	1803	1058	1049	967	1047	538	589	539	614
K18094 membrane fusion protein, multidrug efflux system	1211	1192	1128	1114	581	571	554	627	409	337	314	359
K18095 multidrug efflux pump	1789	1829	1692	1765	983	913	922	1005	537	457	536	574
K18321 membrane fusion protein, multidrug efflux system	0	1	0	1	0	1	0	2	0	1	1	0
K18322 multidrug efflux pump	0	0	0	0	0	0	0	0	1	1	0	0
K18324 multidrug efflux pump	1838	1756	1643	1870	1256	1286	1225	1171	2475	2588	2549	2696
K18326 MFS transporter, DHA2 family, multidrug resistance protein	792	755	728	831	505	551	538	550	1088	1164	1055	1140
K18344 two-component system, OmpR family, response regulator VanR	24	23	26	25	6	12	13	16	9	16	18	13
K18345 two-component system, OmpR family, sensor histidine kinase VanS [EC:2.7.13.3]	23	18	11	17	16	15	13	10	6	15	12	8
K18346 vancomycin resistance protein VanW	1331	1389	1321	1307	834	756	711	786	469	338	370	435
K18347 D-specific alpha-keto acid dehydrogenase [EC:1.1.1.-]	0	1	0	1	0	1	3	2	4	0	2	1
K18348 serine/alanine racemase [EC:5.1.1.18 5.1.1.1]	24	18	18	13	8	17	13	22	18	12	10	23
K18349 two-component system, OmpR family, response regulator VanR	347	413	406	433	137	166	146	153	388	471	403	429

K18350 two-component system, OmpR family, sensor histidine kinase VanS [EC:2.7.13.3]	214	207	192	207	95	102	76	76	319	339	331	351
K18351 two-component system, OmpR family, sensor histidine kinase VanS [EC:2.7.13.3]	0	0	1	0	0	0	0	0	1	2	2	1
K18856 D-alanine---D-serine ligase [EC:6.3.2.35]	13	12	19	21	25	12	14	15	9	11	15	12
K18940 two-component system, OmpR family, sensor histidine kinase ArlS [EC:2.7.13.3]	0	1	0	0	1	0	1	0	2	2	2	2
K18941 two-component system, OmpR family, response regulator ArlR	2	0	2	0	0	0	0	0	0	0	0	0
K18986 two-component system, OmpR family, sensor kinase Ihk [EC:2.7.13.3]	65	69	62	55	58	42	48	51	92	114	87	119
K18987 two-component system, OmpR family, response regulator Irr	0	0	1	0	1	2	0	0	1	2	0	0
K19078 two-component system, OmpR family, response regulator protein GraR	0	0	0	0	0	0	0	0	0	0	1	1
K19082 two-component system, OmpR family, response regulator protein BraR/BceR	2	0	0	0	0	0	0	0	2	1	0	1
K19609 two-component system, OmpR family, sensor histidine kinase PfeS [EC:2.7.13.3]	4136	3792	3783	3753	2095	2084	2050	2403	1353	1106	1172	1205
K19610 two-component system, OmpR family, response regulator PfeR	2198	2249	2038	2127	1194	1092	1100	1155	746	675	574	675
K19611 ferric enterobactin receptor	7723	7692	7242	7732	3954	3974	3781	3943	6619	6802	6502	6609
K19641 two-component system, NtrC family, response regulator HupR/HoxA	1	0	0	0	0	0	0	0	2	1	1	2
K19661 two-component system, NtrC family, sensor histidine kinase HupT/HoxJ [EC:2.7.13.3]	3	0	0	1	0	0	0	0	0	0	0	0
K19691 osomolarity two-component system, sensor histidine kinase NIK1 [EC:2.7.13.3]	1	0	0	0	1	0	0	1	0	0	0	0
K20263 two-component system, NarL family, sensor histidine kinase FusK [EC:2.7.13.3]	360	337	296	332	434	508	500	537	0	0	4	0
K20264 two-component system, NarL family, response regulator FusR	134	150	163	123	167	232	208	186	1	0	0	1
K20340 two-component system, LytTR family, response regulator AgrA2	0	0	0	0	0	0	0	0	1	1	0	1
K20483 lantibiotic biosynthesis protein	0	2	2	0	0	4	5	0	0	2	3	3
K20484 lantibiotic biosynthesis protein	0	2	0	1	2	0	0	0	2	1	0	0
K20485 ATP-binding cassette, subfamily B, bacterial NisT	239	231	259	231	30	39	27	37	508	468	494	527
K20486 lantibiotic leader peptide-processing serine protease [EC:3.4.21.-]	93	124	94	120	10	9	5	12	13	3	0	11
K20487 two-component system, OmpR family, lantibiotic biosynthesis sensor histidine kinase NisK/SpaK [EC:2.7.13.3]	243	271	234	277	85	98	101	88	506	568	526	584
K20488 two-component system, OmpR family, lantibiotic biosynthesis response regulator NisR/SpaR	193	213	200	176	62	77	72	63	331	404	342	320
K20489 lantibiotic immunity protein	19	16	10	11	0	0	0	0	0	0	0	3
K20973 two-component system, sensor histidine kinase SagS [EC:2.7.13.3]	3348	3247	3174	3282	1693	1726	1752	1793	1090	957	981	984
K20974 two-component system, sensor histidine kinase [EC:2.7.13.3]	3187	2912	2857	2969	1519	1512	1391	1589	1175	1037	965	1127
K20975 two-component system, sensor histidine kinase [EC:2.7.13.3]	3200	3285	2805	2985	1644	1505	1445	1694	1068	838	886	885
K20976 histidine phosphotransfer protein HptB	566	586	522	558	267	308	304	300	203	138	158	154
K20977 two-component system, HptB-dependent secretion and biofilm response regulator	2332	2218	2287	2321	1349	1339	1234	1281	758	729	735	713
K20978 HptB-dependent secretion and biofilm anti anti-sigma factor	464	456	431	389	261	249	225	257	159	142	141	135
K23236 two-component-system connector protein SafA	1	0	0	1	4	2	2	1	13	13	9	17
K23514 RNA polymerase sigma-19 factor, ECF subfamily	8732	8639	8155	8303	6516	6306	6009	6677	4279	3827	4088	4100
ko04014 Ras signaling pathway	0	0	0	0	1	2	0	0	0	0	2	2
K02677 classical protein kinase C alpha type [EC:2.7.11.13]	0	0	0	0	0	1	0	0	0	0	0	0
K03099 son of sevenless	0	0	0	0	0	0	0	0	0	0	1	0
K04393 cell division control protein 42	0	0	0	0	1	0	0	0	0	0	0	0
K07889 Ras-related protein Rab-5C	0	0	0	0	0	0	0	0	0	0	0	1
K08052 neurofibromin 1	0	0	0	0	0	1	0	0	0	0	0	1
ko04015 Rap1 signaling pathway	0	0	0	0	1	1	0	0	0	0	0	0
ko04010 MAPK signaling pathway	0	0	1	1	1	2	0	0	0	1	1	2
K04733 interleukin-1 receptor-associated kinase 4 [EC:2.7.11.1]	0	0	0	1	0	0	0	0	0	1	0	0
ko04013 MAPK signaling pathway - fly	3045	3028	2907	2997	2796	2839	2806	2673	2580	2571	2580	2589
K04564 superoxide dismutase, Fe-Mn family [EC:1.15.1.1]	3045	3028	2907	2997	2796	2839	2806	2673	2580	2571	2579	2589
ko04016 MAPK signaling pathway - plant	17254	16503	16581	17093	10411	10170	9933	10375	10603	10833	10503	10764
K13413 mitogen-activated protein kinase kinase 4/5 [EC:2.7.12.2]	0	0	0	0	0	0	0	0	0	0	0	1
K17686 P-type Cu+ transporter [EC:7.2.2.8]	8786	8204	8288	8572	4794	4828	4534	4873	5094	5467	5198	5354
K20716 mitogen-activated protein kinase kinase kinase 17/18	0	0	0	0	0	0	0	1	0	0	0	0
ko04011 MAPK signaling pathway - yeast	7195	7020	7036	7212	4025	3826	3777	3844	4174	4142	4025	4047
K04563 cyclin-dependent kinase [EC:2.7.11.22]	0	0	0	0	0	0	0	0	0	0	0	1
K06276 3-phosphoinositide dependent protein kinase-1 [EC:2.7.11.1]	0	0	0	0	0	0	0	0	1	0	0	0
K06665 general transcriptional corepressor CYC8	0	0	0	0	0	0	0	0	0	0	1	0
K11229 mitogen-activated protein kinase kinase kinase [EC:2.7.11.25]	0	0	0	0	0	0	0	0	0	0	0	1
K19806 tyrosine-protein phosphatase 2/3 [EC:3.1.3.48]	0	0	0	0	0	0	0	0	0	0	1	0
K19844 GTPase-activating protein BEM2	0	0	0	0	0	0	0	0	0	0	1	0
K23469 G2/mitotic-specific cyclin 2	0	0	2	0	0	0	0	0	0	0	0	0
ko04012 ErbB signaling pathway	0	1	0	0	0	1	0	0	0	0	1	0
K07203 serine/threonine-protein kinase mTOR [EC:2.7.11.1]	0	1	0	0	0	0	0	0	0	0	0	0
ko04310 Wnt signaling pathway	0	0	0	0	0	2	0	0	0	0	0	0
K04496 C-terminal binding protein	0	0	0	0	0	1	0	0	0	0	0	0
ko04330 Notch signaling pathway	0	0	0	0	0	1	0	0	0	0	0	0
ko04390 Hippo signaling pathway	1	0	1	0	0	2	0	1	3	0	0	4
K03982 plasminogen activator inhibitor 1	1	0	1	0	0	0	0	1	3	0	0	3
ko04370 VEGF signaling pathway	0	0	0	0	1	1	0	0	0	0	0	0
ko04371 Apelin signaling pathway	3	1	1	0	0	0	0	1	3	4	2	6
K07200 5'-AMP-activated protein kinase, regulatory gamma subunit	0	0	0	0	0	0	0	0	0	0	2	0
ko04630 Jak-STAT signaling pathway	0	1	0	0	0	0	0	0	0	0	1	0
ko04064 NF-kappa B signaling pathway	0	0	0	1	0	0	0	0	0	1	0	0
ko04066 HIF-1 signaling pathway	23815	24012	23460	23637	23723	24761	24150	23941	23363	23128	23168	23560
ko04068 FoxO signaling pathway	10256	10052	9947	10208	6787	6620	6625	6548	6752	6676	6569	6623
ko04020 Calcium signaling pathway	0	0	2	0	0	1	0	0	2	0	1	1
K00871 phosphorylase kinase gamma subunit [EC:2.7.11.19]	0	0	0	0	0	0	0	0	2	0	0	1
K05853 P-type Ca2+ transporter type 2A [EC:7.2.2.10]	0	0	2	0	0	0	0	0	0	0	1	0
ko04070 Phosphatidylinositol signaling system	7403	7692	7646	7601	9057	9096	8810	8743	9092	9007	9065	9039
ko04072 Phospholipase D signaling pathway	927	803	860	901	642	640	545	542	584	584	522	600
ko04071 Sphingolipid signaling pathway	1013	1084	1084	1083	591	585	534	594	350	336	343	310
ko04024 cAMP signaling pathway	0	1	3	1	0	2	0	0	0	1	5	1
ko04022 cGMP-PKG signaling pathway	0	0	2	0	0	2	0	0	0	0	1	1
ko04151 PI3K-Akt signaling pathway	8065	8053	8213	8244	11411	11392	11495	11451	9468	9430	9559	9697
K02091 cyclin-dependent kinase 6 [EC:2.7.11.22]	0	1	0	0	0	0	0	0	0	0	0	0
K08266 target of rapamycin complex subunit LST8	0	0	0	0	0	0	0	1	0	0	0	0
ko04152 AMPK signaling pathway	4959	5107	5036	5074	4847	5184	5014	4838	5442	5465	5453	5433
K03234 elongation factor 2	1	3	3	0	3	4	1	2	5	16	14	11
ko04150 mTOR signaling pathway	2	1	0	1	0	2	0	1	1	4	1	3
ko04075 Plant hormone signal transduction	0	0	0	0	0	0	0	0	0	0	0	1
Signaling molecules and interaction	87	86	94	103	126	97	144	114	110	129	87	106
ko04080 Neuroactive ligand-receptor interaction	87	86	94	103	126	97	144	114	110	129	87	106
K05770 translocator protein	87	86	94	103	126	97	144	114	110	129	87	106
Cellular Processes	852554	846686	827319	841913	686255	687756	673236	689112	616046	615205	604625	613288
Transport and catabolism	57136	59346	61196	59445	113373	112532	115402	112855	94031	91192	93876	91212
ko04144 Endocytosis	19	18	28	22	77	67	0	0	47	57	72	47
K10365 capping protein (actin filament) muscle Z-line, beta	0	0	0	0	0	0	0	0	0	0	2	0
K12191 charged multivesicular body protein 2A	0	0	0	0	1	0	0	0	0	0	0	0
K12481 rabenosyn-5	0	0	0	0	0	0	0	0	0	0	0	1
K17917 sorting nexin-1/2	0	0	0	0	0	0	0	0	0	0	0	1
K17918 sorting nexin-3/12	0	0	1	0	0	0	0	0	0	0	0	0
ko04145 Phagosome	0	0	0	1	0	1	0	0	0	0	0	2
ko04142 Lysosome	26364	28255	30655	28609	79743	79515	81638	79073	65050	62835	65419	62526
K01279 tripeptidyl-peptidase I [EC:3.4.14.9]	0	1	1	0	0	1	0	0	0	0	1	0
K01373 cathepsin F [EC:3.4.22.41]	3	1	0	1	1	0	1	1	2	3	0	2
K12381 arylsulfatase G [EC:3.1.6.-]	0	1	0	1	0	0	0	0	1	8	3	1
K12399 AP-3 complex subunit sigma	0	0	1	0	0	0	0	0	0	0	0	0
K13444 formylglycine-generating enzyme [EC:1.8.3.7]	14	14	7	9	5	5	2	2	11	12	19	13
ko04146 Peroxisome	30480	30814	30222	30538	33386	32764	33610	33591	28608	27944	28035	28219
K00624 carnitine O-acetyltransferase [EC:2.3.1.7]	0	1	0	0	0	2	0	0	0	0	0	0
K04565 superoxide dismutase, Cu-Zn family [EC:1.15.1.1]	347	403	322	357	229	235	244	260	524	550	489	563
K13343 peroxin-14	0	0	1	0	0	0	0	0	0	0	0	0
ko04140 Autophagy - animal	0	1	0	1	0	1	2	1	5	2	1	4
ko04138 Autophagy - yeast	272	259	289	275	166	183	154	191	325	356	349	418
K06655 negative regulator of the PHO system [EC:2.7.11.22]	0	0	1	0	0	0	0	0	0	0	0	0
K06902 MFS transporter, UMF1 family	272	258	286	274	165	182	152	188	321	354	349	414
K12761 carbon catabolite-derepressing protein kinase [EC:2.7.11.1]	0	0	2	0	0	0	0	0	0	0	0	0
K15296 alpha-soluble NSF attachment protein	0	0	0	0	0	0	0	2	0	0	0	0
K20179 vacuolar protein sorting-associated protein 11	0	0	0	0	1	0	0	0	0	0	0	0
ko04136 Autophagy - other	0	1	0	0	0	0	0	1	0	0	0	0
ko04139 Mitophagy - yeast	20	19	26	22	76	69	0	0	47	57	69	44
K08955 ATP-dependent metalloprotease [EC:3.4.24.-]	0	0	0	0	0	1	0	0	0	0	0	0
K17774 mitochondrial distribution and morphology protein 10	0	0	2	0	0	0	0	0	0	0	0	0
Cell growth and death	118730	119166	120118	120010	142997	142060	143408	142378	122481	120894	121855	120789
ko04110 Cell cycle	1	3	1	1	1	0	0	1	2	6	3	4
K02365 separase [EC:3.4.22.49]	0	1	0	0	0	0	0	0	0	0	0	0

ko04111 Cell cycle - yeast	0	2	4	0	0	0	0	0	0	2	1	2
K06647 transcription factor MBP1	0	0	0	0	0	0	0	0	0	0	0	1
ko04112 Cell cycle - Caulobacter	59720	60086	60909	61161	70546	70690	71067	69849	61788	60754	61176	60755
K01338 ATP-dependent Lon protease [EC:3.4.21.53]	7617	7601	7965	8096	8182	8413	8263	8171	8224	7962	8251	8173
K01358 ATP-dependent Clp protease, protease subunit [EC:3.4.21.92]	3123	3062	3133	3180	3074	3048	3016	3028	2738	2718	2749	2793
K02651 pilus assembly protein Flp/PilA	322	325	346	361	213	188	179	204	125	107	110	98
K03531 cell division protein FtsZ	3266	3160	3281	3324	4560	4673	4678	4325	4201	4482	4094	4068
K03544 ATP-dependent Clp protease ATP-binding subunit ClpX	3450	3474	3607	3551	3940	3963	4181	3954	4058	4384	4286	4243
K03588 cell division protein FtsW	4155	4338	4488	4244	4947	4868	4635	4880	4828	4564	4411	4501
K03589 cell division protein FtsQ	2766	2927	2756	2849	3055	2966	2992	2812	2724	2693	2764	2683
K03590 cell division protein FtsA	2525	2803	2862	2670	4311	4407	4183	4209	3755	3651	3748	3749
K06985 aspartyl protease family protein	2432	2397	2238	2323	2718	2787	2804	2725	1973	1729	2027	1981
K11749 regulator of sigma E protease [EC:3.4.24.-]	4442	4239	4221	4337	4908	4637	4943	4645	4369	4344	4144	4345
K13581 modification methylase [EC:2.1.1.72]	1	2	2	0	1	0	0	0	1	1	4	2
K13582 localization factor PodJL	2	2	2	0	0	2	0	1	6	4	5	7
K13583 GcrA cell cycle regulator	0	0	0	0	2	4	1	1	0	2	1	0
K13590 diguanylate cyclase [EC:2.7.7.65]	4013	3997	3896	3898	2315	2157	2082	2171	1320	1252	1212	1287
K13592 regulator of CtrA degradation	2	0	2	0	0	0	2	0	0	0	8	1
ko04113 Meiosis - yeast	1713	1628	1473	1628	874	804	827	846	577	476	507	510
K10878 meiotic recombination protein SPO11	0	3	0	0	0	0	0	0	0	0	0	0
K12762 G protein-coupled receptor GPR1	0	2	0	0	0	2	0	0	0	0	0	0
ko04114 Oocyte meiosis	0	2	1	0	0	2	0	0	0	0	0	1
ko04210 Apoptosis	4779	4705	4695	4691	7191	7152	7076	7293	5276	4819	5016	4828
K01173 endonuclease G, mitochondrial	4240	4259	4225	4196	6905	6884	6810	7026	5063	4657	4869	4680
ko04214 Apoptosis - fly	4512	4329	4375	4352	3495	3526	3510	3422	3243	3293	3203	3222
K03386 peroxiredoxin (alkyl hydroperoxide reductase subunit C) [EC:1.11.1.15]	3889	3798	3811	3756	3084	3162	3103	3042	2927	3006	2970	2974
ko04215 Apoptosis - multiple species	536	445	470	493	285	267	263	266	206	158	146	142
ko04216 Ferroptosis	10372	11098	11007	10856	18986	18536	19293	19142	15352	14871	14985	14862
ko04217 Necroptosis	38168	37764	38125	37814	42187	41619	41898	42090	36446	36833	37110	36746
K03767 peptidyl-prolyl cis-trans isomerase A (cyclophilin A) [EC:5.2.1.8]	1305	1186	1310	1288	815	735	717	827	884	877	828	854
ko04115 p53 signaling pathway	537	446	471	493	285	267	263	267	209	158	146	145
ko04218 Cellular senescence	1	2	1	0	2	2	0	1	3	0	0	4
Cellular community - eukaryotes	2	0	0	1	2	3	0	1	3	4	6	5
ko04510 Focal adhesion	0	0	0	0	1	3	0	0	1	0	1	1
ko04520 Adherens junction	0	0	0	0	1	0	0	0	0	0	0	0
ko04530 Tight junction	1	0	0	1	2	0	0	1	2	4	5	4
ko04540 Gap junction	0	0	0	0	0	1	0	0	0	0	1	0
ko04550 Signaling pathways regulating pluripotency of stem cells	1	0	0	0	0	0	0	0	0	0	0	0
K14439 SWI/SNF-related matrix-associated actin-dependent regulator of chromatin subfamily A containing DEAD/H box 1 [EC:3.6.4.12]	1	0	0	0	0	0	0	0	0	0	0	0
Cellular community - prokaryotes	561723	557523	538207	551468	386790	387392	376995	392329	374917	376662	365223	375727
ko02024 Quorum sensing	269533	268066	263584	268201	195748	196687	193120	197908	202660	205771	200171	204364
K01318 glutamyl endopeptidase [EC:3.4.21.19]	0	1	2	0	1	2	0	1	0	1	3	1
K01399 pseudolysin [EC:3.4.24.26]	2326	2348	2320	2310	1363	1294	1249	1447	724	714	694	774
K02031 peptide/nickel transport system ATP-binding protein	4119	4068	3953	4183	1432	1678	1495	1523	4947	5162	4987	5172
K02032 peptide/nickel transport system ATP-binding protein	2972	2980	2797	3048	1268	1290	1220	1225	4479	4652	4384	4538
K02033 peptide/nickel transport system permease protein	5025	4949	4791	4998	2095	2167	2083	1870	6403	6572	6573	6542
K02034 peptide/nickel transport system permease protein	5217	4944	5007	5244	2193	2308	2107	2118	6039	6250	5817	6129
K02035 peptide/nickel transport system substrate-binding protein	10637	10571	10359	10567	5462	5515	5272	5446	10203	10800	10154	10829
K02052 putative spermidine/putrescine transport system ATP-binding protein	4397	4422	4392	4387	2545	2640	2494	2609	2122	2153	2017	2180
K02053 putative spermidine/putrescine transport system permease protein	1936	1972	1781	2016	1152	1176	1342	1333	1146	1162	1175	1178
K02054 putative spermidine/putrescine transport system permease protein	2868	2916	2878	2854	1587	1707	1625	1627	1424	1545	1505	1541
K02055 putative spermidine/putrescine transport system substrate-binding protein	8661	8595	8348	8431	4986	4958	4743	5005	3415	3502	3450	3587
K02250 competence protein ComK	0	3	3	0	0	0	0	0	0	0	2	1
K02251 competence protein ComQ	0	0	0	2	0	0	0	3	0	0	0	0
K07344 type IV secretion system protein TrbL	4077	3982	4026	3967	2284	2189	2170	2469	1272	1194	1232	1206
K08321 3-hydroxy-5-phosphonooxypentane-2,4-dione thiolase [EC:2.3.1.245]	571	535	471	549	402	446	434	458	653	774	712	737
K08605 coccolysin [EC:3.4.24.30]	84	75	44	68	9	12	9	7	47	79	76	72
K08642 LasA protease [EC:3.4.24.-]	1740	1674	1595	1614	923	855	776	879	551	524	481	475
K09823 Fur family transcriptional regulator, zinc uptake regulator	582	587	535	524	402	418	345	346	596	605	636	624
K09936 bacterial/archaeal transporter family-2 protein	2202	2199	2024	2025	1037	976	977	1001	1693	1779	1621	1608
K11031 thiol-activated cytolysin	279	292	312	329	490	405	470	514	30	61	45	47
K11035 hemolysin BL binding component	0	0	0	0	0	0	0	2	0	0	0	0
K11063 toxin A/B	0	0	0	0	0	0	0	0	0	0	0	1
K11216 autoinducer-2 kinase [EC:2.7.1.189]	1013	968	960	1053	803	764	828	872	1197	1334	1296	1361
K11530 (4S)-4-hydroxy-5-phosphonooxypentane-2,3-dione isomerase [EC:5.3.1.32]	655	632	611	653	413	395	366	428	355	369	340	364
K11531 lsr operon transcriptional repressor	647	590	667	621	414	405	416	411	877	765	821	938
K12990 rhamnosyltransferase [EC:2.4.1.-]	472	458	405	465	215	201	188	244	126	129	87	130
K13075 N-acyl homoserine lactone hydrolase [EC:3.1.1.81]	288	292	258	245	97	99	108	95	683	640	581	584
K14051 c-di-GMP phosphodiesterase Gmr [EC:3.1.4.52]	393	378	372	368	494	618	558	527	101	93	109	95
K14645 serine protease [EC:3.4.21.-]	5	1	2	1	5	4	9	7	0	2	0	1
K18099 LuxR family transcriptional regulator, quorum-sensing system regulator RhlR	1069	1022	990	1045	570	575	612	639	348	339	314	377
K18100 rhamnosyltransferase subunit A [EC:2.4.1.-]	1252	1155	1317	1211	696	728	762	740	406	386	418	376
K18101 rhamnosyltransferase subunit B [EC:2.4.1.-]	2051	2050	1964	2090	1147	1151	1038	1109	617	608	590	592
K18139 outer membrane protein, multidrug efflux system	3509	3553	3566	3778	4510	4584	4565	4586	4067	3807	3808	3905
K18304 LuxR family transcriptional regulator, quorum-sensing system regulator LasR	3	2	2	2	0	3	0	2	0	0	0	0
K18306 membrane fusion protein, multidrug efflux system	1429	1472	1246	1319	772	681	806	905	439	408	441	398
K18307 multidrug efflux pump	2755	2747	2533	2568	1499	1438	1382	1667	962	896	741	880
K19735 LysR family transcriptional regulator, quorum-sensing system regulator MvfR	1552	1457	1456	1452	853	898	788	859	498	461	428	458
K20252 LuxR family transcriptional regulator, quorum-sensing system regulator CinR	0	0	0	0	0	0	0	0	0	0	0	1
K20258 PA-I galactophilic lectin	373	406	400	379	226	200	163	214	118	111	120	125
K20259 PA-IIL fucose-binding lectin	500	508	483	554	257	269	267	227	153	194	151	159
K20265 glutamate:GABA antiporter	3106	3243	3093	3117	4571	4411	4271	4472	3520	3383	3469	3350
K20266 type IV secretion system protein TrbJ	3280	3200	3279	3197	1777	1788	1352	1480	959	948	964	979
K20271 DeoR family transcriptional regulator, repressor of opine catabolism and conjugal transfer	0	0	0	0	0	0	0	0	2	4	2	2
K20276 large repetitive protein	8703	8291	7610	7945	6911	7360	7690	8251	1583	1572	1443	1571
K20277 mannose-binding lectin	564	558	523	563	273	297	287	315	181	143	170	169
K20345 membrane fusion protein, peptide pheromone/bacteriocin exporter	129	147	162	132	249	238	254	201	81	128	119	107
K20373 HTH-type transcriptional regulator, SHP2-responsive activator	3	0	2	3	0	0	2	1	0	2	0	0
K20375 HTH-type transcriptional regulator, pheromone-responsive regulator	0	0	0	0	5	7	3	8	14	14	17	20
K20377 HTH-type transcriptional regulator, major conjugation operon repressor	13	12	6	5	0	0	0	0	0	0	0	0
K20379 sex pheromone cAD1	48	67	58	74	7	13	5	12	51	69	24	58
K20380 HTH-type transcriptional regulator, pheromone-responsive repressor	69	58	91	76	8	7	5	7	4	0	3	5
K20381 regulatory protein	33	32	32	28	3	4	8	5	2	18	4	4
K20382 aggregation substance	280	308	308	310	20	17	14	23	25	13	17	15
K20383 CylL-L protein	19	16	30	20	5	0	0	3	0	1	1	0
K20384 CylL-S protein	24	13	11	19	2	1	2	0	0	2	2	1
K20385 CylM protein	210	235	217	247	14	16	9	15	5	16	10	5
K20387 regulatory protein CylR1	9	13	12	10	0	1	0	0	1	1	0	0
K20388 HTH-type transcriptional regulator, cytolysin regulator	8	9	14	11	0	1	0	0	0	2	3	0
K20480 HTH-type transcriptional regulator, quorum sensing regulator NprR	2	0	2	0	0	0	0	3	1	1	2	3
K20527 type IV secretion system protein TrbB [EC:7.4.2.8]	3292	3231	3157	3261	1770	1713	1661	1715	1064	928	936	944
K20528 type IV secretion system protein TrbC	1079	1060	1037	1016	599	615	506	607	321	298	302	334
K20529 type IV secretion system protein TrbD	750	779	775	730	375	417	398	393	219	212	211	208
K20530 type IV secretion system protein TrbE [EC:7.4.2.8]	8042	7997	7602	8030	4280	4199	4173	4688	2493	2407	2373	2514
K20531 type IV secretion system protein TrbF	2102	2075	2012	2123	1075	1043	946	1198	669	703	613	590
K20532 type IV secretion system protein TrbG	4341	4269	4134	4170	2294	2134	2097	2355	1294	1222	1258	1195
K20533 type IV secretion system protein TrbI	5616	5365	5233	5456	3040	2685	2669	2981	1663	1546	1653	1600
K20539 IclR family transcriptional regulator, blcABC operon repressor	0	0	0	0	2	0	2	0	0	2	2	1
ko05111 Biofilm formation - Vibrio cholerae	86461	86502	82069	84310	63006	62478	61230	64076	61266	61081	59257	61054
K02463 general secretion pathway protein N	289	350	283	306	65	79	88	88	524	542	532	639
K03087 RNA polymerase nonessential primary-like sigma factor	2240	2349	2215	2383	1378	1348	1313	1304	1385	1440	1417	1440
K03557 Fis family transcriptional regulator, factor for inversion stimulation protein	663	668	625	573	358	397	378	432	374	411	383	351
K03606 putative colanic acid biosysnthesis UDP-glucose lipid carrier transferase	2299	2322	2544	2432	7525	7374	7668	7551	5499	5040	5271	5065
K05946 N-acetylglucosaminyldiphosphoundecaprenol N-acetyl-beta-D-mannosaminyltransferase [EC:2.4.1.187]	599	646	645	638	458	497	522	477	701	819	754	739
K08604 vibriolysin [EC:3.4.24.25]	0	0	0	0	0	0	0	0	1	0	0	0
K10924 MSHA pilin protein MshA	1	2	0	1	0	0	0	0	1	1	0	0
K10939 accessory colonization factor AcfD	8	14	14	20	26	47	35	27	185	231	233	231
K12276 MSHA biogenesis protein MshE	1	2	2	1	0	0	1	1	4	0	3	1
K16554 polysaccharide biosynthesis transport protein	0	0	0	0	3	2	8	2	4	0	3	1
K20920 polysaccharide biosynthesis protein VpsM	0	0	0	0	2	2	0	2	4	0	2	0
K20922 polysaccharide biosynthesis protein VpsI	1	1	0	1	0	0	0	0	1	0	0	0

K20955 diguanylate cyclase [EC:2.7.7.65]	0	0	0	0	0	0	0	1	0	0	0	0
K20956 diguanylate cyclase [EC:2.7.7.65]	0	0	1	0	1	0	0	0	0	0	0	1
K20957 diguanylate cyclase [EC:2.7.7.65]	2	2	1	0	0	0	0	0	0	0	1	0
K20959 diguanylate cyclase [EC:2.7.7.65]	0	1	0	2	2	2	0	1	0	0	0	2
K20962 c-di-GMP phosphodiesterase [EC:3.1.4.52]	1	0	1	0	0	0	0	0	0	0	0	0
K20965 c-di-GMP phosphodiesterase [EC:3.1.4.52]	1	0	1	0	0	0	0	0	1	0	2	1
K20966 c-di-GMP phosphodiesterase [EC:3.1.4.52]	9	10	5	7	2	0	0	1	2	4	4	5
K20988 polysaccharide biosynthesis/export protein VpsN	0	0	3	0	0	0	0	0	0	0	2	0
ko02025 Biofilm formation - Pseudomonas aeruginosa	197505	194776	185121	190346	112339	111490	106507	115329	80408	77735	74610	78166
K11890 type VI secretion system protein ImpM	889	780	674	807	510	538	533	596	243	192	191	187
K11893 type VI secretion system protein ImpJ	6081	5798	5460	5615	3329	3372	3140	3184	3333	3324	3054	3176
K11895 type VI secretion system protein ImpH	5736	5720	5227	5540	3380	3399	3045	3343	2659	2725	2684	2760
K11900 type VI secretion system protein ImpC	6497	6612	6290	6482	3859	4000	3646	4078	2743	2594	2615	2669
K11901 type VI secretion system protein ImpB	2701	2737	2551	2664	1553	1578	1581	1646	1116	1108	1039	1186
K11902 type VI secretion system protein ImpA	3531	3402	3026	3247	2030	1925	1714	1852	988	897	830	973
K12992 rhamnosyltransferase [EC:2.4.1.-]	1687	1603	1482	1568	787	695	681	687	682	618	590	678
K20968 AraC family transcriptional regulator, exoenzyme S synthesis regulatory protein ExsA	154	175	190	209	109	111	88	102	49	49	54	50
K20971 two-component system, sensor histidine kinase LadS	42	55	38	44	19	28	171	223	16	27	16	17
K20972 two-component system, sensor histidine kinase RetS	432	454	404	413	256	226	196	228	168	111	132	149
K20987 polysaccharide biosynthesis/export protein PslD	108	127	94	103	51	44	46	55	40	27	24	30
K20997 polysaccharide biosynthesis protein PslA	1967	1897	1876	1921	1082	1020	982	976	616	555	483	571
K20998 polysaccharide biosynthesis protein PslE	6	5	5	7	1	3	3	1	2	2	1	2
K20999 polysaccharide biosynthesis protein PslF	1296	1364	1273	1311	732	668	640	700	456	418	410	421
K21000 polysaccharide biosynthesis protein PslG	388	345	288	311	182	186	187	207	116	101	90	88
K21001 polysaccharide biosynthesis protein PslH	1653	1503	1378	1484	734	830	738	826	463	468	409	402
K21002 polysaccharide biosynthesis protein PslI	1437	1257	1138	1263	641	682	558	668	400	385	308	410
K21003 polysaccharide biosynthesis protein PslJ	962	1052	917	919	489	467	459	510	320	317	243	287
K21004 polysaccharide biosynthesis protein PslK	1704	1704	1462	1638	845	837	743	896	451	448	426	467
K21005 polysaccharide biosynthesis protein PslL	374	373	350	350	180	188	167	227	109	91	107	120
K21006 polysaccharide biosynthesis protein PelA	3802	3722	3393	3447	1959	1783	1808	2021	1088	1087	1116	1167
K21007 polysaccharide biosynthesis protein PelB	3546	3424	3145	3192	1730	1712	1785	1823	1091	945	1039	976
K21008 polysaccharide biosynthesis protein PelC	124	100	91	91	84	58	49	57	39	30	33	34
K21009 polysaccharide biosynthesis protein PelD	1295	1296	1201	1214	676	637	637	775	441	388	373	379
K21010 polysaccharide biosynthesis protein PelE	1086	1032	1114	1066	610	588	563	580	359	373	299	319
K21011 polysaccharide biosynthesis protein PelF	2162	2091	2192	2145	1161	1252	1098	1154	819	725	715	776
K21012 polysaccharide biosynthesis protein PelG	1961	1948	2048	1961	1092	1002	1044	1089	649	655	703	667
K21019 diguanylate cyclase [EC:2.7.7.65]	2142	2191	2015	2029	1192	1257	1121	1118	714	662	610	657
K21020 diguanylate cyclase [EC:2.7.7.65]	1607	1550	1538	1575	915	857	787	885	536	497	437	488
K21021 diguanylate cyclase [EC:2.7.7.65]	2573	2370	2307	2348	1175	1145	1024	1195	1552	1451	1381	1551
K21022 diguanylate cyclase [EC:2.7.7.65]	1871	1730	1770	1663	891	883	899	983	520	596	535	548
K21023 diguanylate cyclase [EC:2.7.7.65]	1197	1199	1131	1293	1304	1303	1333	1325	171	186	173	179
K21024 c-di-GMP phosphodiesterase [EC:3.1.4.52]	3285	3215	3094	3172	1757	1800	1678	1935	1033	1026	1018	1060
K21025 multidomain signaling protein FimX	1367	1329	1297	1347	780	838	688	804	446	451	419	439
K23127 cyclic-di-GMP-binding protein	2914	2762	2722	2817	1521	1517	1524	1572	861	928	889	987
ko02026 Biofilm formation - Escherichia coli	70504	70566	68389	70531	55029	56103	54450	54952	66313	68170	66065	68148
K02425 regulator of sigma S factor FliZ	110	107	103	76	164	187	156	145	21	25	33	20
K03566 LysR family transcriptional regulator, glycine cleavage system transcriptional activator	5336	5334	4976	5280	3537	3561	3236	3655	4335	4519	4184	4455
K03567 glycine cleavage system transcriptional repressor	1285	1360	1280	1306	778	846	750	774	1040	1076	952	1115
K04333 LuxR family transcriptional regulator, csgAB operon transcriptional regulatory protein	401	395	361	403	243	263	252	298	482	524	494	563
K04334 major curlin subunit	92	97	103	118	122	142	94	69	21	22	32	18
K04335 minor curlin subunit	33	33	24	32	51	59	55	48	12	7	11	9
K04336 curli production protein	68	67	62	64	108	96	87	122	30	11	29	28
K04761 LysR family transcriptional regulator, hydrogen peroxide-inducible genes activator	3045	2983	2944	3090	3729	3509	3607	3738	2635	2622	2625	2597
K06204 DnaK suppressor protein	1816	1785	1720	1691	1047	1083	1068	1114	1004	918	918	957
K11931 poly-beta-1,6-N-acetyl-D-glucosamine N-deacetylase [EC:3.5.1.-]	922	877	860	863	213	241	251	249	1742	1737	1759	1930
K11935 biofilm PGA synthesis protein PgaA	972	1019	984	945	280	254	303	267	2140	2294	2206	2247
K11936 poly-beta-1,6-N-acetyl-D-glucosamine synthase [EC:2.4.1.-]	544	533	519	597	181	139	143	147	1154	1191	1123	1139
K11937 biofilm PGA synthesis protein PgaD	145	181	181	180	50	43	55	67	383	408	407	376
K12687 antigen 43	15	7	5	9	29	33	13	22	114	118	113	115
K18968 diguanylate cyclase [EC:2.7.7.65]	242	207	181	206	353	366	380	329	85	61	77	74
K21084 diguanylate cyclase [EC:2.7.7.65]	773	691	710	683	948	1094	1075	966	207	196	282	238
K21085 diguanylate cyclase [EC:2.7.7.65]	887	933	902	845	583	707	616	615	1160	1287	1265	1284
K21086 c-di-GMP phosphodiesterase [EC:3.1.4.52]	508	444	399	433	341	284	337	307	539	588	554	587
K21087 flagellar brake protein	168	129	154	155	244	226	231	198	50	35	38	44
K21088 diguanylate cyclase [EC:2.7.7.65]	602	571	625	672	458	498	442	440	876	847	863	942
K21089 MerR family transcriptional regulator, activator of the csg genes	166	143	146	152	246	222	211	236	28	44	44	39
K21090 c-di-GMP phosphodiesterase [EC:3.1.4.52]	881	837	787	915	663	631	663	622	1098	1194	1177	1282
Cell motility	142652	139723	136489	139293	88504	90244	83421	86997	62180	63149	60881	62317
ko02030 Bacterial chemotaxis	98814	96934	94197	95789	62468	63504	58017	60325	42698	42722	41364	42374
K02410 flagellar motor switch protein FliG	2057	1899	1896	1947	1123	1112	1064	1080	1137	1219	1158	1215
K02416 flagellar motor switch protein FliM	2209	2191	2104	2218	1200	1299	1232	1181	1128	1095	1091	1117
K02417 flagellar motor switch protein FliN/FliY	1652	1638	1632	1633	683	778	672	734	844	1017	976	969
K02557 chemotaxis protein MotB	3165	3098	3216	3065	3340	3365	3283	3221	2722	2564	2653	2498
K03409 chemotaxis protein CheX	2	2	2	0	0	0	0	0	0	0	0	0
K03410 chemotaxis protein CheC	346	308	340	356	233	276	268	235	407	392	438	403
K03411 chemotaxis protein CheD [EC:3.5.1.44]	1395	1213	1131	1220	601	629	597	693	659	617	554	586
K03414 chemotaxis protein CheZ	693	632	654	648	427	398	460	462	208	192	161	181
ko02040 Flagellar assembly	55198	53757	53256	54569	33501	34441	32808	34082	26444	27453	26412	26790
K02386 flagella basal body P-ring formation protein FlgA	1260	1313	1262	1214	721	773	763	858	324	396	333	334
K02387 flagellar basal-body rod protein FlgB	603	642	678	651	360	327	382	359	298	306	329	348
K02388 flagellar basal-body rod protein FlgC	985	918	987	986	529	525	496	515	537	563	495	496
K02389 flagellar basal-body rod modification protein FlgD	1327	1329	1377	1483	775	774	798	792	489	536	464	555
K02390 flagellar hook protein FlgE	1404	1372	1330	1360	885	884	799	842	770	853	714	795
K02391 flagellar basal-body rod protein FlgF	1290	1304	1331	1290	940	881	818	905	359	381	388	404
K02392 flagellar basal-body rod protein FlgG	1047	949	1008	1009	516	589	514	471	722	899	854	795
K02393 flagellar L-ring protein precursor FlgH	1193	1160	1230	1190	816	759	739	798	409	360	374	388
K02394 flagellar P-ring protein precursor FlgI	289	326	316	340	377	424	341	391	106	93	102	87
K02396 flagellar hook-associated protein 1 FlgK	2307	2223	2276	2288	1377	1476	1344	1319	1079	1208	1201	1210
K02397 flagellar hook-associated protein 3 FlgL	2783	2621	2716	2821	1400	1502	1471	1585	1263	1318	1193	1233
K02399 flagella synthesis protein FlgN	833	726	726	793	489	495	455	474	243	245	231	231
K02400 flagellar biosynthesis protein FlhA	4942	4672	4684	4872	2496	2614	2287	2630	2674	2652	2653	2788
K02401 flagellar biosynthetic protein FlhB	1416	1383	1498	1360	813	892	809	840	803	856	815	832
K02407 flagellar hook-associated protein 2	3135	3117	3015	3159	1702	1771	1691	1725	1307	1386	1303	1353
K02408 flagellar hook-basal body complex protein FliE	703	665	713	627	361	381	368	416	250	241	278	290
K02409 flagellar M-ring protein FliF	3024	3158	2875	3033	1790	1962	1586	1752	1258	1249	1128	1173
K02411 flagellar assembly protein FliH	1443	1364	1227	1334	813	760	745	851	497	520	491	533
K02412 flagellum-specific ATP synthase [EC:7.4.2.8]	2859	2827	2609	2693	1664	1647	1646	1642	1368	1367	1313	1294
K02413 flagellar FliJ protein	549	561	543	564	344	346	368	375	201	180	208	214
K02414 flagellar hook-length control protein FliK	1874	1908	1737	1940	1185	1214	1196	1357	580	542	567	585
K02418 flagellar protein FliO/FliZ	478	477	442	484	342	334	326	311	160	199	176	184
K02419 flagellar biosynthetic protein FliP	944	922	900	941	529	507	526	502	599	668	690	659
K02420 flagellar biosynthetic protein FliQ	513	469	455	530	262	277	291	282	245	280	250	238
K02421 flagellar biosynthetic protein FliR	851	832	814	817	467	542	513	450	468	498	564	500
K02422 flagellar protein FliS	923	867	868	851	535	510	503	522	409	443	449	410
K02423 flagellar protein FliT	84	65	61	93	119	116	121	121	20	10	22	23
K13820 flagellar biosynthetic protein FliR/FlhB	1	0	0	0	0	0	0	0	4	9	4	9
K21218 sodium-type flagellar protein MotY	1398	1322	1295	1394	814	717	737	810	512	404	393	413
ko04810 Regulation of actin cytoskeleton	19	18	24	22	76	69	0	0	47	57	70	44
Organismal Systems	178529	179361	178429	178268	184535	185254	184739	185634	165612	164450	164714	163088
Immune system	13041	13177	13501	13391	17039	16991	17169	17009	14716	14777	14722	14770
ko04610 Complement and coagulation cascades	1	0	1	0	0	0	0	1	3	0	0	3
ko04611 Platelet activation	0	0	0	0	0	2	0	0	0	0	0	1
ko04620 Toll-like receptor signaling pathway	0	0	0	1	0	0	0	0	0	1	0	0
ko04624 Toll and Imd signaling pathway	0	0	0	1	0	0	0	2	0	1	0	0
K10380 ankyrin	0	0	0	0	0	0	0	2	0	0	0	0
ko04621 NOD-like receptor signaling pathway	11995	12071	12429	12323	16270	16187	16457	16340	13252	13406	13353	13286
K03671 thioredoxin 1	2520	2662	2851	2688	3470	3304	3363	3367	3312	3363	3317	3101
K04612 calcium-sensing receptor	0	0	0	0	0	0	0	0	1	0	0	0
ko04622 RIG-I-like receptor signaling pathway	1025	1087	1046	1046	693	734	712	666	1413	1314	1298	1436
K11594 ATP-dependent RNA helicase DDX3X [EC:3.6.4.13]	0	0	0	0	0	0	0	0	0	1	0	0
ko04650 Natural killer cell mediated cytotoxicity	0	0	0	0	0	1	0	0	0	0	1	0

ko04612 Antigen processing and presentation	8026	8029	8184	8223	11366	11368	11453	11419	9422	9406	9524	9662
ko04660 T cell receptor signaling pathway	0	0	0	0	1	0	0	0	1	0	1	0
ko04659 Th17 cell differentiation	8026	8030	8183	8223	11366	11368	11453	11419	9422	9406	9524	9661
ko04657 IL-17 signaling pathway	8027	8029	8183	8223	11366	11368	11453	11419	9422	9406	9524	9661
ko04662 B cell receptor signaling pathway	0	0	0	0	0	0	0	0	0	0	1	0
ko04664 Fc epsilon RI signaling pathway	0	0	0	0	0	1	0	0	1	0	1	0
ko04666 Fc gamma R-mediated phagocytosis	19	18	24	22	76	68	0	0	47	57	70	43
ko04670 Leukocyte transendothelial migration	0	0	0	0	1	1	0	0	0	0	0	0
ko04062 Chemokine signaling pathway	0	0	0	0	1	0	0	0	0	0	1	0
Endocrine system	85228	86103	84534	84701	89535	90344	90104	91208	80633	79365	80025	79369
ko04911 Insulin secretion	0	0	0	0	0	1	0	0	0	0	0	0
ko04910 Insulin signaling pathway	10000	10423	10406	10170	14543	14946	14744	14399	14021	13968	14479	13865
K07192 flotillin	895	968	1003	957	2053	2140	2205	2095	1778	1672	1812	1623
ko04922 Glucagon signaling pathway	24570	24749	24363	24073	24600	25086	24727	24663	26096	26245	26454	25811
ko04923 Regulation of lipolysis in adipocytes	1	0	0	1	0	1	0	1	0	0	0	1
ko04920 Adipocytokine signaling pathway	8806	9429	9308	9114	17271	16719	17607	17426	13721	13107	13394	13271
ko03320 PPAR signaling pathway	36028	36345	35679	35800	36219	35948	36046	37037	30356	29701	29415	29738
K08770 ubiquitin C	1	0	0	0	1	0	0	0	0	0	0	0
ko04912 GnRH signaling pathway	0	0	0	0	1	1	0	0	0	0	2	0
ko04915 Estrogen signaling pathway	8027	8029	8184	8223	11366	11368	11453	11419	9422	9406	9525	9662
ko04914 Progesterone-mediated oocyte maturation	8026	8030	8184	8223	11366	11368	11453	11419	9422	9408	9524	9661
ko04917 Prolactin signaling pathway	1321	1413	1373	1488	1030	1083	983	977	1529	1441	1495	1511
ko04921 Oxytocin signaling pathway	1	3	3	0	3	7	1	2	5	16	16	12
ko04926 Relaxin signaling pathway	0	0	0	0	0	1	0	0	0	0	1	0
ko04918 Thyroid hormone synthesis	6142	6104	5909	5851	4383	4551	4584	4660	4342	4239	4219	4188
ko04919 Thyroid hormone signaling pathway	6513	6573	6100	6453	3388	3541	3352	3634	2144	1954	1809	2070
ko04928 Parathyroid hormone synthesis, secretion and action	0	0	0	0	0	2	0	1	5	1	3	1
K03626 nascent polypeptide-associated complex subunit alpha	0	0	0	0	0	1	0	1	4	1	2	1
ko04916 Melanogenesis	0	0	0	0	0	1	0	0	0	0	0	0
ko04614 Renin-angiotensin system	1101	1213	1233	1176	3977	4022	4125	4042	3029	2895	3260	2845
K01322 prolyl oligopeptidase [EC:3.4.21.26]	1101	1208	1233	1176	3977	4022	4125	4042	3028	2895	3257	2845
K01392 thimet oligopeptidase [EC:3.4.24.15]	0	0	0	0	0	0	0	0	1	0	0	0
K08780 carboxypeptidase A3 [EC:3.4.17.1]	0	5	0	0	0	0	0	0	0	0	3	0
ko04925 Aldosterone synthesis and secretion	0	0	0	0	0	1	0	0	0	0	0	0
ko04927 Cortisol synthesis and secretion	0	0	1	1	0	0	0	0	6	4	10	12
K14349 neutral cholesterol ester hydrolase 1 [EC:3.1.1.-]	0	0	1	1	0	0	0	0	6	4	10	12
Circulatory system	3668	3691	3846	3809	2174	2301	2108	2052	1263	1256	1169	1147
ko04260 Cardiac muscle contraction	3668	3691	3846	3809	2174	2298	2108	2052	1263	1256	1169	1146
ko04261 Adrenergic signaling in cardiomyocytes	0	0	2	0	0	3	0	0	0	0	1	1
ko04270 Vascular smooth muscle contraction	0	0	0	0	0	3	0	0	0	0	0	1
Digestive system	7127	7634	7782	7664	15544	15802	15992	15651	12931	12646	12883	12540
ko04970 Salivary secretion	0	0	0	0	0	1	0	0	0	0	0	0
ko04971 Gastric acid secretion	0	0	0	0	0	1	0	0	0	0	0	0
ko04972 Pancreatic secretion	0	6	2	2	0	2	0	0	2	1	7	8
ko04976 Bile secretion	12	7	8	10	2	4	6	3	27	29	28	36
ko04973 Carbohydrate digestion and absorption	3056	3194	3262	3327	4622	4866	4740	4750	5104	5028	5146	5167
ko04974 Protein digestion and absorption	3112	3443	3550	3343	10262	10368	10610	10263	7405	7158	7322	6934
K01278 dipeptidyl-peptidase 4 [EC:3.4.14.5]	3112	3437	3550	3343	10262	10368	10609	10261	7405	7158	7319	6931
K14208 Xaa-Pro aminopeptidase 2 [EC:3.4.11.9]	0	0	0	0	0	0	0	1	0	0	0	1
K14210 solute carrier family 3 (neutral and basic amino acid transporter), member 1	0	0	0	0	0	0	1	1	0	0	0	2
K16628 collagen, type VII, alpha	0	1	0	0	0	0	0	0	0	0	0	0
ko04975 Fat digestion and absorption	0	1	0	2	1	1	2	0	2	2	3	8
ko04979 Cholesterol metabolism	87	86	95	104	126	97	144	114	116	133	97	118
ko04978 Mineral absorption	860	903	866	879	531	465	490	521	283	300	296	289
K07213 copper chaperone	860	903	866	879	531	465	490	521	283	300	296	289
Excretory system	2043	1923	2018	2096	2893	3054	3289	2914	3694	3538	3853	3655
ko04962 Vasopressin-regulated water reabsorption	0	0	0	0	0	1	0	0	0	0	0	1
K12462 Rho GDP-dissociation inhibitor	0	0	0	0	0	1	0	0	0	0	0	0
ko04960 Aldosterone-regulated sodium reabsorption	0	0	0	0	0	1	0	0	1	0	0	0
ko04961 Endocrine and other factor-regulated calcium reabsorption	0	0	0	0	0	1	0	0	0	0	0	0
ko04964 Proximal tubule bicarbonate reclamation	2043	1923	2018	2095	2893	3051	3289	2914	3693	3538	3853	3654
ko04966 Collecting duct acid secretion	0	0	0	1	0	1	0	0	0	0	0	0
Nervous system	27075	26686	26746	26510	28824	28324	28853	29209	22373	22202	22656	22066
ko04724 Glutamatergic synapse	23491	22949	23048	22848	24057	23576	24216	24286	18930	18842	19237	18793
ko04727 GABAergic synapse	24531	24020	24134	23917	27376	26908	27543	27662	21506	21326	21862	21312
ko04725 Cholinergic synapse	0	0	0	0	0	1	0	0	0	0	0	0
ko04728 Dopaminergic synapse	2541	2665	2610	2588	1445	1412	1307	1544	864	874	791	750
ko04726 Serotonergic synapse	2541	2665	2610	2588	1445	1410	1307	1544	864	874	791	749
ko04720 Long-term potentiation	0	0	0	0	0	3	0	0	0	0	0	1
ko04730 Long-term depression	0	0	0	0	0	1	0	0	0	0	0	0
ko04723 Retrograde endocannabinoid signaling	1	0	2	1	2	4	3	1	2	1	1	4
ko04721 Synaptic vesicle cycle	2	1	0	3	0	1	0	2	0	0	0	0
K05038 solute carrier family 6 (neurotransmitter transporter, glycine) member 5/9	2	1	0	2	0	0	0	0	0	0	0	0
ko04722 Neurotrophin signaling pathway	0	0	0	1	1	1	0	0	1	1	1	0
Sensory system	0	0	0	0	0	3	0	0	0	0	0	1
ko04745 Phototransduction - fly	0	0	0	0	0	1	0	0	0	0	0	0
ko04750 Inflammatory mediator regulation of TRP channels	0	0	0	0	0	3	0	0	0	0	0	1
Development and regeneration	908	786	838	879	568	573	545	542	537	528	452	557
ko04320 Dorso-ventral axis formation	0	0	0	0	0	0	0	0	0	0	1	0
ko04360 Axon guidance	0	0	0	0	1	1	0	0	0	0	0	0
ko04361 Axon regeneration	908	786	836	879	567	572	545	542	537	527	451	557
ko04380 Osteoclast differentiation	0	0	2	0	0	0	0	0	0	1	0	0
Aging	33006	32777	33300	33166	31413	31363	30776	30146	30974	31053	30962	30479
ko04211 Longevity regulating pathway	10220	10031	9917	10187	6744	6597	6583	6517	6706	6656	6534	6590
ko04212 Longevity regulating pathway - worm	24384	24109	24720	24458	22093	22192	21304	21081	21443	21743	21589	21177
ko04213 Longevity regulating pathway - multiple species	18842	18699	18497	18895	16064	15768	16055	15582	16237	15966	15905	15892
K03695 ATP-dependent Clp protease ATP-binding subunit ClpB	6561	6640	6785	6721	8216	8134	8397	7957	8426	8282	8373	8226
Environmental adaptation	48101	48891	48185	48451	50342	50325	50690	51443	43711	43716	42953	43764
ko04710 Circadian rhythm	0	0	0	0	0	0	0	0	0	0	2	0
ko04713 Circadian entrainment	0	0	0	0	0	1	0	0	0	0	0	0
ko04714 Thermogenesis	17786	18246	18318	18105	22356	21963	22394	22413	17264	16738	16719	16712
K11450 lysine-specific histone demethylase 1A [EC:1.-.-.-]	0	0	0	0	0	0	0	0	0	0	1	0
K18160 NADH dehydrogenase [ubiquinone] 1 alpha subcomplex assembly factor 2	0	0	0	0	0	0	0	0	0	0	0	1
ko04626 Plant-pathogen interaction	30315	30645	29867	30346	27986	28361	28296	29030	26447	26978	26234	27052
K13408 membrane fusion protein	716	677	699	736	296	308	270	242	1111	1166	1097	1167
Human Diseases	433129	432509	428098	432004	385339	389210	379746	382930	388756	390610	386542	389318
Cancer: overview	58098	57711	56996	57707	57992	58696	58091	58929	54959	53497	54153	53819
ko05200 Pathways in cancer	24479	24180	23868	24491	21025	20992	20510	21131	18509	18069	18110	18426
ko05202 Transcriptional misregulation in cancer	1	1	1	1	0	0	0	1	0	2	0	1
K00069 15-hydroxyprostaglandin dehydrogenase (NAD) [EC:1.1.1.141]	1	0	0	1	0	0	0	1	0	0	0	0
ko05206 MicroRNAs in cancer	4450	4428	4579	4488	8528	8938	9136	8783	8220	7815	8123	8010
ko05205 Proteoglycans in cancer	2269	2348	2292	2492	3098	3100	3018	3176	2862	2799	2860	2743
ko05204 Chemical carcinogenesis	10340	10167	9884	10361	6013	5970	5699	5945	6581	6308	6186	6437
ko05203 Viral carcinogenesis	7147	7132	7138	6837	6773	6674	6742	6952	7213	6987	7129	6912
K11407 histone deacetylase 6 [EC:3.5.1.98]	0	0	0	0	0	0	0	0	2	0	0	0
K15979 staphylococcal nuclease domain-containing protein 1	0	0	2	0	0	0	0	0	0	0	0	0
ko05230 Central carbon metabolism in cancer	25119	24949	24698	24529	25968	26445	26612	26688	25725	25043	25647	24969
ko05231 Choline metabolism in cancer	929	804	860	901	645	647	550	544	593	593	526	611
ko05235 PD-L1 expression and PD-1 checkpoint pathway in cancer	0	1	0	0	0	0	0	0	0	0	0	0
Cancer: specific types	25303	25079	24709	25390	21636	21648	21092	21700	19748	19317	19222	19753
ko05210 Colorectal cancer	536	446	470	493	285	267	263	266	206	159	147	142
ko05212 Pancreatic cancer	0	2	0	0	1	0	0	0	0	0	0	0
ko05225 Hepatocellular carcinoma	10749	10616	10336	10896	6255	6142	5882	6398	6401	6103	6082	6189
ko05226 Gastric cancer	11	7	5	8	2	3	6	3	16	11	13	12
ko05214 Glioma	0	2	0	0	0	1	0	0	0	0	1	0
ko05221 Acute myeloid leukemia	0	1	0	0	0	0	0	0	0	0	1	0
ko05220 Chronic myeloid leukemia	0	1	0	0	0	1	0	0	0	0	1	0
ko05218 Melanoma	0	1	0	0	0	0	0	0	0	0	0	0
ko05211 Renal cell carcinoma	5167	5090	4879	4879	3119	3214	2912	3048	2480	2401	2359	2434
ko05219 Bladder cancer	813	893	836	891	609	653	576	566	1222	1237	1100	1315
ko05215 Prostate cancer	8027	8030	8183	8223	11366	11368	11453	11419	9423	9406	9525	9661

ko05213 Endometrial cancer	0	0	0	0	0	0	0	0	1	0	1	0
ko05224 Breast cancer	0	2	0	0	0	0	0	0	0	0	1	0
ko05222 Small cell lung cancer	536	446	470	493	285	267	263	266	206	158	146	142
ko05223 Non-small cell lung cancer	0	1	0	0	0	1	0	0	1	0	1	0
Immune disease	2829	2939	2985	3050	2582	2557	2683	2528	3418	3756	3780	3630
ko05323 Rheumatoid arthritis	0	0	2	1	0	1	0	0	0	1	0	0
ko05340 Primary immunodeficiency	2829	2939	2983	3049	2582	2556	2683	2528	3418	3755	3780	3630
Neurodegenerative disease	21284	21341	21199	21477	15092	15136	14898	14720	13950	14007	13605	13904
ko05010 Alzheimer disease	10716	10903	10958	10933	8115	8302	8068	7943	6715	6795	6579	6748
ko05012 Parkinson disease	4209	4144	4327	4309	2465	2573	2378	2319	1484	1422	1334	1298
ko05014 Amyotrophic lateral sclerosis (ALS)	8059	7850	7802	8040	4462	4260	4284	4370	4856	4793	4588	4706
K04575 amyotrophic lateral sclerosis 2 protein	1	0	0	0	0	0	0	0	0	0	0	0
ko05016 Huntington disease	7601	7579	7554	7663	5490	5645	5425	5252	4589	4546	4403	4453
ko05020 Prion diseases	347	404	322	357	229	235	244	260	524	551	489	563
Substance dependence	2541	2665	2610	2588	1445	1412	1307	1544	866	874	792	750
ko05030 Cocaine addiction	2541	2665	2610	2588	1445	1409	1307	1544	864	874	791	749
ko05031 Amphetamine addiction	2541	2665	2610	2588	1445	1412	1307	1544	864	874	791	750
ko05032 Morphine addiction	0	0	0	0	0	1	0	0	0	0	0	0
ko05034 Alcoholism	2541	2665	2610	2588	1445	1411	1307	1544	866	874	792	750
Cardiovascular disease	25987	26132	26042	26290	26068	25658	25621	26356	23567	23257	23178	23344
ko05418 Fluid shear stress and atherosclerosis	25451	25687	25570	25797	25783	25391	25358	26090	23361	23099	23029	23202
ko05410 Hypertrophic cardiomyopathy (HCM)	0	0	2	0	0	0	0	0	0	0	3	0
ko05412 Arrhythmogenic right ventricular cardiomyopathy (ARVC)	0	0	2	0	0	0	0	0	0	0	1	0
ko05414 Dilated cardiomyopathy (DCM)	0	0	2	0	0	0	0	0	0	0	1	0
ko05416 Viral myocarditis	536	445	470	493	285	267	263	266	206	158	146	142
Endocrine and metabolic disease	36409	36823	36940	36118	39470	39773	38709	38709	35804	35918	36416	35662
ko04930 Type II diabetes mellitus	7811	7857	7847	7535	9322	9295	9412	9660	9167	8776	9166	8828
ko04940 Type I diabetes mellitus	5297	5240	5561	5171	8431	8576	8015	8013	7589	7551	7731	7408
ko04932 Non-alcoholic fatty liver disease (NAFLD)	4204	4136	4314	4303	2460	2567	2373	2318	1474	1415	1317	1295
ko04931 Insulin resistance	13929	14500	14337	14229	16139	16120	15997	15669	15085	15771	15836	15682
ko04933 AGE-RAGE signaling pathway in diabetic complications	1	0	1	0	1	1	0	1	3	0	0	3
ko04934 Cushing syndrome	5167	5091	4880	4880	3118	3214	2912	3048	2486	2405	2368	2446
Infectious disease: bacterial	119846	119132	117207	118561	97186	98763	93431	94477	97836	98567	97925	99311
ko05110 Vibrio cholerae infection	2165	2130	2117	2034	1324	1294	1182	1336	631	667	615	599
K10954 zona occludens toxin	2165	2130	2117	2033	1324	1292	1182	1336	630	667	615	599
ko05120 Epithelial cell signaling in Helicobacter pylori infection	11809	11476	11592	11615	11994	12219	12309	11842	14163	14596	14320	14570
K03191 acid-activated urea channel	0	0	2	0	0	0	0	1	1	0	2	1
K08303 putative protease [EC:3.4.-.-]	7471	7308	7728	7446	9980	10396	10485	9874	10527	10825	10809	10761
ko05130 Pathogenic Escherichia coli infection	0	0	0	0	1	1	0	0	0	0	0	0
ko05132 Salmonella infection	9131	9041	9116	9238	10716	11184	11068	10743	10668	10515	10636	10673
K05916 nitric oxide dioxygenase [EC:1.14.12.17]	2505	2462	2425	2496	1545	1580	1450	1563	1474	1608	1521	1602
K12264 anaerobic nitric oxide reductase flavorubredoxin	996	911	928	945	594	705	639	569	1269	1263	1201	1319
K12265 nitric oxide reductase FlRd-NAD(+) reductase [EC:1.18.1.-]	697	748	688	748	500	529	516	466	995	926	884	963
K12266 anaerobic nitric oxide reductase transcription regulator	1898	1817	1883	1921	1113	1245	1096	1227	1595	1581	1539	1613
K13771 Rrf2 family transcriptional regulator, nitric oxide-sensitive transcriptional repressor	311	298	248	276	208	230	226	247	414	385	383	441
ko05131 Shigellosis	0	0	0	0	1	1	0	0	7	6	3	16
K11011 enterotoxin Set1A	0	0	0	0	0	1	0	0	6	6	3	15
K11012 enterotoxin Set1B	0	0	0	0	0	0	0	0	1	0	0	1
ko05135 Yersinia infection	1736	1863	1731	1773	1145	1101	899	1005	1011	1007	965	969
K13735 adhesin/invasin	20	26	22	25	112	133	156	129	418	442	407	435
K23475 transloator	1062	1181	1060	1081	629	560	436	543	361	301	317	307
K23476 transloator	635	638	625	644	328	341	307	333	185	206	172	184
ko05133 Pertussis	61860	61731	59032	60500	37204	37618	34012	35350	40093	39986	39353	41042
K07326 hemolysin activation/secretion protein	4645	4473	4114	4386	2414	2311	2314	2582	1429	1361	1346	1379
K07345 major type 1 subunit fimbrin (pilin)	3088	3162	3141	3209	1823	1884	1741	1802	3340	3214	3111	3378
K07347 outer membrane usher protein	21757	21819	20940	21618	11494	11469	11072	11356	17377	17890	17470	18304
K07389 cytolysin-activating lysine-acyltransferase [EC:2.3.1.-]	12	16	23	22	3	2	1	3	49	67	56	85
K15125 filamentous hemagglutinin	12530	12223	11243	11692	6340	6318	3629	4209	4250	3865	3819	3969
ko05134 Legionellosis	16792	16528	16951	17054	19206	19665	19000	19207	17522	17716	17866	17391
K03596 GTP-binding protein LepA	5434	5432	5318	5588	5745	5835	5734	6005	5719	5834	5837	5767
K07874 Ras-related protein Rab-1A	0	0	1	0	0	0	0	0	0	0	0	0
ko05150 Staphylococcus aureus infection	5693	5531	5629	5534	3697	3689	3404	3453	2345	2470	2361	2544
K11041 exfoliative toxin A/B	123	129	131	134	33	39	30	24	128	148	122	157
ko05152 Tuberculosis	16444	16376	16955	16420	18304	18552	17661	17540	17026	17283	17418	16872
ko05100 Bacterial invasion of epithelial cells	163	176	155	156	151	171	179	155	516	607	553	623
K13730 internalin A	143	150	133	131	38	38	23	26	98	165	146	188
Infectious disease: viral	7800	7696	7715	7465	7242	7093	7209	7385	7570	7323	7407	7205
ko05166 Human T-cell leukemia virus 1 infection	87	92	95	103	128	97	144	115	113	133	92	109
K08874 transformation/transcription domain-associated protein	0	0	0	0	0	0	0	0	0	0	2	0
K11304 histone acetyltransferase HTATIP [EC:2.3.1.48]	0	2	0	0	0	0	0	0	0	0	0	0
ko05170 Human immunodeficiency virus 1 infection	536	446	470	494	285	268	263	266	206	159	146	142
ko05162 Measles	536	446	471	495	285	268	265	266	210	161	146	147
ko05164 Influenza A	557	464	482	520	346	319	321	315	252	209	183	182
ko05161 Hepatitis B	537	445	470	495	286	268	263	267	208	164	150	146
ko05160 Hepatitis C	536	446	470	494	285	268	265	266	210	160	147	146
ko05168 Herpes simplex virus 1 infection	544	455	475	499	286	270	267	268	212	165	151	152
K09228 KRAB domain-containing zinc finger protein	0	0	0	0	0	0	0	0	0	0	1	0
ko05163 Human cytomegalovirus infection	536	447	470	493	285	268	263	266	206	158	147	142
ko05167 Kaposi sarcoma-associated herpesvirus infection	536	447	470	493	285	267	263	266	206	158	146	142
ko05169 Epstein-Barr virus infection	536	447	470	494	285	267	263	266	207	159	147	142
ko05165 Human papillomavirus infection	7147	7133	7132	6837	6768	6675	6742	6952	7203	6975	7126	6906
K12236 transcriptional repressor NF-X1	0	0	0	0	1	0	0	0	0	0	0	0
Infectious disease: parasitic	5030	4709	4864	4918	2754	2895	2836	2946	3399	3389	3225	3430
ko05146 Amoebiasis	340	367	352	346	212	226	250	212	661	651	628	681
K13963 serpin B	10	16	9	9	5	3	0	3	21	25	25	28
ko05145 Toxoplasmosis	536	445	471	494	285	267	263	266	207	159	146	143
ko05140 Leishmaniasis	0	0	0	1	0	0	0	0	0	1	0	0
ko05142 Chagas disease (American trypanosomiasis)	3862	3649	3788	3823	2109	2269	2185	2340	2426	2493	2357	2502
K01354 oligopeptidase B [EC:3.4.21.83]	3861	3649	3787	3822	2109	2269	2185	2339	2423	2492	2357	2499
ko05143 African trypanosomiasis	4153	3897	4040	4078	2257	2403	2323	2467	2528	2579	2451	2603
K14475 inhibitor of cysteine peptidase	292	248	253	256	148	133	138	128	104	87	94	104
Drug resistance: antimicrobial	184012	183965	182503	184152	164647	166555	163321	164537	171064	173124	169995	171510
ko01501 beta-Lactam resistance	102035	102455	102559	102669	94665	95957	94437	94402	97066	98109	96493	96791
K02171 BlaI family transcriptional regulator, penicillinase repressor	97	106	78	86	18	30	35	22	109	98	93	101
K02172 bla regulator protein blaR1	591	533	503	572	135	133	131	124	538	651	579	630
K03585 membrane fusion protein, multidrug efflux system	8515	8742	9237	8856	13268	13321	12784	12883	10656	10421	10443	10230
K08218 MFS transporter, PAT family, beta-lactamase induction signal transducer AmpG	5180	5314	5286	5070	7425	7460	7782	7326	5981	5833	5979	5694
K17837 metallo-beta-lactamase class B [EC:3.5.2.6]	291	311	328	278	748	675	720	706	470	414	425	420
K17838 beta-lactamase class D [EC:3.5.2.6]	0	0	0	0	1	0	0	1	0	0	0	0
K17850 LysR family transcriptional regulator, regulator of gene expression of beta-lactamase	1319	1327	1274	1225	808	690	701	754	420	403	406	380
K18129 TetR/AcrR family transcriptional regulator, mexXY operon repressor	847	798	781	835	450	413	417	433	256	255	216	250
K18130 TetR/AcrR family transcriptional regulator, transcriptional repressor NalC	1008	1018	970	979	566	526	469	577	295	272	281	318
K18131 MarR family transcriptional regulator, repressor of the mexAB-oprM multidrug resistance operon	596	569	578	600	318	330	324	322	208	193	186	172
K18135 TetR/AcrR family transcriptional regulator, repressor of the mexAB-oprM multidrug resistance operon	561	533	594	528	269	276	247	310	176	165	132	155
K18138 multidrug efflux pump	16452	16175	16335	16226	15802	15639	15705	15659	14086	13988	13943	13763
K18143 two-component system, OmpR family, sensor histidine kinase AdeS [EC:2.7.13.3]	520	506	584	551	383	431	363	385	692	841	793	827
K18144 two-component system, OmpR family, response regulator AdeR	382	388	364	379	258	266	268	260	521	592	484	550
K18145 membrane fusion protein, multidrug efflux system	0	0	0	0	0	0	0	1	0	0	0	1
K18146 multidrug efflux pump	1558	1599	1564	1695	1131	1248	1149	1089	2260	2288	2179	2404
K18148 release factor H-coupled RctB family protein	1908	1823	1604	1842	1036	949	936	1024	707	696	600	653
K18150 antirepressor for MexR	217	231	233	219	126	117	116	141	67	64	68	72
K18699 beta-lactamase class A SHV [EC:3.5.2.6]	24	31	16	22	8	7	6	12	47	46	41	41
K18767 beta-lactamase class A CTX-M [EC:3.5.2.6]	0	0	0	0	0	0	0	0	2	0	0	0
K18796 beta-lactamase class A LEN [EC:3.5.2.6]	2	0	2	0	0	1	4	4	1	0	0	0
K18973 beta-lactamase class D OXA-50 [EC:3.5.2.6]	1345	1441	1369	1386	777	784	795	861	488	443	417	412
K19096 beta-lactamase class C CMY-2 [EC:3.5.2.6]	0	0	0	0	2	0	2	0	1	2	0	1
K19215 beta-lactamase class C ACT/MIR [EC:3.5.2.6]	0	0	0	0	0	0	0	0	1	0	0	0
K19216 metallo-beta-lactamase class B IND [EC:3.5.2.6]	0	0	0	1	2	0	0	2	1	0	3	0
K20320 beta-lactamase class C PDC [EC:3.5.2.6]	1933	1961	1829	1890	1072	1061	941	1141	663	612	555	576
K21277 beta-lactamase class D OXA-60 [EC:3.5.2.6]	1	0	0	0	0	0	0	0	0	0	0	0
ko01502 Vancomycin resistance	24717	24886	24649	24660	28824	29198	29360	28813	27169	27398	27118	26786

ko01503 Cationic antimicrobial peptide (CAMP) resistance	88684	88362	87392	88438	78253	78507	76377	78495	77924	78165	76991	78186
K01406 serralysin [EC:3.4.24.40]	1684	1682	1566	1582	973	863	896	888	576	534	516	536
K01448 N-acetylmuramoyl-L-alanine amidase [EC:3.5.1.28]	8921	8949	8994	8960	9021	9132	8962	9053	8809	8877	8693	8769
K03673 thiol:disulfide interchange protein DsbA	1492	1669	1702	1634	1083	1021	1004	1129	1373	1488	1472	1500
K06079 copper homeostasis protein (lipoprotein)	492	465	477	445	337	397	331	424	519	638	558	569
K12962 undecaprenyl phosphate-alpha-L-ara4N flippase subunit ArnE	792	702	674	779	443	404	418	448	462	424	408	459
K12963 undecaprenyl phosphate-alpha-L-ara4N flippase subunit ArnF	466	423	344	453	297	276	262	299	374	387	397	404
K13632 AraC family transcriptional regulator, mar-sox-rob regulon activator	194	175	199	177	163	146	125	155	289	301	264	253
K19236 L,D-transpeptidase YcfS	522	519	506	488	311	389	396	373	811	723	734	739
K19238 signal transduction protein PmrD	173	113	168	149	101	101	110	94	213	217	225	176
Drug resistance: antineoplastic	39097	38159	37889	38521	32773	32581	32259	32942	31499	30544	30126	30545
ko01521 EGFR tyrosine kinase inhibitor resistance	2	1	0	0	0	2	0	0	0	4	1	4
ko01524 Platinum drug resistance	17388	16450	16525	17194	9668	9634	9148	9739	10322	10424	10019	10432
ko01523 Antifolate resistance	21707	21708	21364	21327	23105	22946	23111	23203	21178	20117	20106	20109
ko01522 Endocrine resistance	0	1	0	0	0	0	0	0	0	0	1	0
Brite Hierarchies	5418349	5398942	5339635	5401726	4741005	4780733	4715107	4749119	4925928	4956758	4890240	4953017
Protein families: metabolism	1096552	1092366	1082616	1095412	995638	1004854	990434	993473	995825	1000562	989552	999807
ko01001 Protein kinases	152919	150861	146140	150476	94896	96148	93043	95912	109312	110809	107760	111659
K00936 two-component system, sensor histidine kinase PdtaS [EC:2.7.13.3]	132	137	125	126	37	31	35	26	168	201	183	176
K02476 two-component system, CitB family, sensor kinase [EC:2.7.13.3]	87	77	71	74	12	20	11	14	93	101	81	85
K02478 two-component system, LytTR family, sensor kinase [EC:2.7.13.3]	2011	1853	1857	1941	1283	1324	1340	1293	2767	2768	2596	2731
K02480 two-component system, NarL family, sensor kinase [EC:2.7.13.3]	3	0	3	3	0	3	0	1	1	11	2	2
K02482 two-component system, NtrC family, sensor kinase [EC:2.7.13.3]	1771	1853	1886	1852	994	1065	983	1013	621	611	650	649
K02484 two-component system, OmpR family, sensor kinase [EC:2.7.13.3]	1004	1054	1149	1077	3603	3500	3623	3438	2652	2681	2773	2671
K02486 two-component system, sensor kinase [EC:2.7.13.3]	3	2	0	1	0	0	0	0	0	0	1	1
K04757 serine/threonine-protein kinase RsbW [EC:2.7.11.1]	136	106	136	149	39	43	57	36	242	251	261	270
K07154 serine/threonine-protein kinase HipA [EC:2.7.11.1]	1927	2100	2118	2082	5065	5138	5352	5097	4650	4574	4782	4675
K08857 NIMA (never in mitosis gene a)-related kinase 1/4/5 [EC:2.7.11.1]	0	0	0	0	0	1	0	0	0	0	0	0
K08869 aarF domain-containing kinase	0	0	0	0	1	0	0	0	0	0	0	0
K08884 serine/threonine protein kinase, bacterial [EC:2.7.11.1]	49	38	46	39	14	9	7	10	123	105	114	149
K11527 two-component system, sensor histidine kinase and response regulator [EC:2.7.13.3]	688	736	834	687	2331	2388	2448	2186	1672	1561	1782	1513
K12132 eukaryotic-like serine/threonine-protein kinase [EC:2.7.11.1]	1372	1307	1454	1366	732	856	773	668	1051	1149	1051	1170
K15904 bifunctional N6-L-threonylcarbamoyladenine synthase / protein kinase Bud32 [EC:2.3.1.234 2.7.11.1]	0	0	5	1	2	4	5	2	3	3	8	10
K16309 serine/threonine-protein kinase greatwall [EC:2.7.11.1]	0	0	2	0	0	0	0	0	4	2	2	1
K17752 serine/threonine-protein kinase RsbT [EC:2.7.11.1]	1	0	0	0	0	0	0	0	0	0	0	0
K19852 serine/threonine protein kinase KIN1/2 [EC:2.7.11.1]	0	0	0	0	0	1	0	0	0	0	0	0
ko01009 Protein phosphatases and associated proteins	15991	16129	16357	16336	22340	22960	22565	22176	20202	20375	20321	20436
K15732 RNA polymerase II subunit A C-terminal domain phosphatase [EC:3.1.3.16]	0	0	0	0	0	0	0	0	0	0	1	0
K17623 pseudouridine 5'-phosphatase [EC:3.1.3.96]	0	0	2	0	0	1	0	0	2	3	3	1
K20074 PPM family protein phosphatase [EC:3.1.3.16]	643	549	621	629	366	376	383	302	419	523	432	464
K21814 serine/threonine-protein phosphatase CPPED1 [EC:3.1.3.16]	3	1	1	0	1	0	0	2	0	0	3	0
ko01002 Peptidases	281229	280455	280046	282141	280291	281848	280342	278403	278351	278636	277235	277455
K00683 glutaminyl-peptide cyclotransferase [EC:2.3.2.5]	0	1	0	0	0	0	0	0	0	0	0	0
K01258 tripeptide aminopeptidase [EC:3.4.11.4]	2499	2497	2450	2486	3425	3429	3393	3298	3409	3442	3514	3493
K01261 glutamyl aminopeptidase [EC:3.4.11.7]	70	76	74	70	26	25	16	20	73	108	87	61
K01262 Xaa-Pro aminopeptidase [EC:3.4.11.9]	6820	7053	7095	7041	10081	10301	10219	10033	8131	8448	8328	8038
K01265 methionyl aminopeptidase [EC:3.4.11.18]	4600	4762	4781	5020	5587	5587	5539	5544	5322	5500	5452	5382
K01266 D-aminopeptidase [EC:3.4.11.19]	2205	2118	2051	2071	941	852	815	988	984	928	953	1052
K01267 aspartyl aminopeptidase [EC:3.4.11.21]	2173	2142	2113	2248	999	919	928	955	1107	1066	1151	1042
K01268 aminopeptidase I [EC:3.4.11.22]	0	0	2	0	0	0	0	0	0	0	0	0
K01271 Xaa-Pro dipeptidase [EC:3.4.13.9]	1815	1787	1660	1755	974	984	1030	909	1597	1885	1646	1640
K01273 membrane dipeptidase [EC:3.4.13.19]	2581	2349	2467	2544	1111	1233	1118	1135	1729	1838	1638	1805
K01277 dipeptidyl-peptidase III [EC:3.4.14.4]	1280	1431	1481	1341	4377	4244	4422	4242	3024	2818	3128	2955
K01281 X-Pro dipeptidyl-peptidase [EC:3.4.14.11]	4	2	2	5	3	12	3	3	4	2	5	3
K01284 peptidyl-dipeptidase Dcp [EC:3.4.15.5]	3812	4056	4237	4133	10238	9931	10208	10020	8067	7774	7922	7596
K01293 Gly-Xaa carboxypeptidase [EC:3.4.17.4]	0	0	0	0	2	0	0	1	2	2	2	2
K01295 glutamate carboxypeptidase [EC:3.4.17.11]	2183	2282	2121	2304	1588	1468	1385	1435	978	952	870	960
K01297 muramoyltetrapeptide carboxypeptidase [EC:3.4.17.13]	2724	2711	2622	2706	3036	3001	2891	2989	2721	2585	2753	2657
K01299 carboxypeptidase Taq [EC:3.4.17.19]	1355	1364	1301	1289	580	646	640	610	1509	1579	1616	1653
K01303 acylaminoacyl-peptidase [EC:3.4.19.1]	3	2	0	1	0	0	0	0	3	3	3	3
K01304 pyroglutamyl-peptidase [EC:3.4.19.3]	325	303	308	312	109	103	133	122	611	637	554	630
K01305 beta-aspartyl-dipeptidase (metallo-type) [EC:3.4.19.-]	205	201	220	171	194	211	231	214	332	376	374	358
K01308 g-D-glutamyl-meso-diaminopimelate peptidase [EC:3.4.19.11]	192	158	178	175	26	27	23	22	146	191	171	177
K01337 lysyl endopeptidase [EC:3.4.21.50]	2300	2223	1934	2176	1256	1150	1180	1302	761	693	643	676
K01342 subtilisin [EC:3.4.21.62]	0	0	0	1	2	1	1	2	3	2	0	0
K01355 omptin [EC:3.4.23.49]	7	5	3	2	3	0	12	0	99	80	103	99
K01356 repressor LexA [EC:3.4.21.88]	2122	2065	2086	2199	937	968	882	933	4131	4493	4364	4183
K01361 lactocepin [EC:3.4.21.96]	1	0	2	1	46	37	31	29	2	0	1	3
K01372 bleomycin hydrolase [EC:3.4.22.40]	1630	1700	1912	1721	5149	5117	5161	4992	3613	3709	3770	3509
K01387 microbial collagenase [EC:3.4.24.3]	2	2	4	0	0	2	0	0	0	0	0	1
K01400 bacillolysin [EC:3.4.24.28]	0	2	2	0	3	2	0	0	2	0	2	0
K01407 protease III [EC:3.4.24.55]	1799	1776	1758	1826	1263	1314	1298	1308	2398	2473	2408	2555
K01414 oligopeptidase A [EC:3.4.24.70]	3793	3749	3609	3777	2339	2380	2282	2412	2827	2680	2780	2850
K01419 ATP-dependent HslUV protease, peptidase subunit HslV [EC:3.4.25.2]	1209	1214	1198	1261	710	740	668	664	796	832	799	770
K01423 beta-barrel assembly-enhancing protease [EC:3.4.-.-]	1117	1147	1082	1200	845	875	829	873	1072	1077	1102	1148
K01436 amidohydrolase [EC:3.5.1.-]	67	57	57	55	31	34	30	27	36	56	49	58
K02278 prepilin peptidase CpaA [EC:3.4.23.43]	794	789	749	819	422	352	354	429	315	315	280	323
K02506 leader peptidase HopD [EC:3.4.23.43]	344	329	294	368	198	217	274	193	490	458	465	472
K02654 leader peptidase (prepilin peptidase) / N-methyltransferase [EC:3.4.23.43 2.1.1.-]	1657	1600	1687	1618	928	870	802	772	782	793	770	830
K03152 protein deglycase [EC:3.5.1.124]	1006	1040	1095	1081	1443	1372	1442	1468	1758	1645	1604	1688
K03503 DNA polymerase V [EC:3.4.21.-]	777	776	717	754	839	828	873	803	443	457	473	453
K03568 TldD protein	2812	2709	2586	2765	1562	1627	1647	1739	1859	1962	1776	1968
K03592 PmbA protein	2257	2097	2137	2134	1273	1341	1461	1273	1801	1655	1593	1751
K03605 hydrogenase maturation protease [EC:3.4.23.-]	160	146	132	161	216	266	241	204	123	105	101	107
K03797 carboxyl-terminal processing protease [EC:3.4.21.102]	10362	10318	10581	10739	15231	15344	15241	14813	12139	11830	12439	11888
K03798 cell division protease FtsH [EC:3.4.24.-]	7788	7625	8090	7725	7320	7558	7310	6910	8155	8566	8405	8347
K03799 heat shock protein HtpX [EC:3.4.24.-]	1932	1906	1866	1996	2826	2657	2691	2523	2365	2311	2436	2342
K04076 ATP-dependent Lon protease [EC:3.4.21.53]	6	1	3	3	2	3	1	0	10	16	19	14
K04691 serine protease DegS [EC:3.4.21.-]	2563	2511	2407	2530	1527	1551	1497	1434	1497	1601	1489	1543
K04770 Lon-like ATP-dependent protease [EC:3.4.21.-]	960	994	886	1025	696	752	709	675	1367	1431	1364	1427
K04772 serine protease DegQ [EC:3.4.21.-]	898	908	896	996	557	748	584	656	1320	1255	1277	1285
K04773 protease IV [EC:3.4.21.-]	2343	2493	2529	2537	4719	4893	4974	4673	4443	4195	4303	4345
K04774 serine protease SohB [EC:3.4.21.-]	1739	1727	1810	1757	988	1051	1047	1104	1009	1067	962	1068
K04775 protease YdgD [EC:3.4.21.-]	426	452	523	548	278	367	321	320	655	687	656	634
K05520 protease I [EC:3.5.1.124]	3325	3317	3206	3126	1795	1778	1762	1856	1793	1858	1688	1838
K05995 dipeptidase E [EC:3.4.13.21]	245	282	261	261	127	120	103	117	655	688	620	668
K05998 pseudomonalisin [EC:3.4.21.100]	0	0	0	0	0	1	0	0	0	0	0	0
K06012 spore protease [EC:3.4.24.78]	522	512	516	473	178	132	145	140	361	377	360	366
K06336 spore coat-associated protein N	14	12	11	16	5	5	5	10	21	15	27	18
K06383 stage II sporulation protein GA (sporulation sigma-E factor processing peptidase) [EC:3.4.23.-]	255	256	264	247	93	77	93	62	221	221	217	227
K06399 stage IV sporulation protein B [EC:3.4.21.116]	460	501	521	502	144	134	133	131	434	462	466	418
K06402 stage IV sporulation protein FB [EC:3.4.24.-]	1	2	0	2	2	1	0	1	3	6	7	7
K06972 presequence protease [EC:3.4.24.-]	1432	1431	1487	1523	322	349	336	356	1362	1408	1413	1327
K06974 archaemetzincin [EC:3.4.-.-]	7	5	12	9	34	30	34	33	20	29	19	32
K07010 putative glutamine amidotransferase	1625	1647	1621	1727	771	777	850	897	719	718	752	745
K07177 Lon-like protease	58	56	69	77	15	16	19	21	68	59	61	75
K07261 penicillin-insensitive murein DD-endopeptidase [EC:3.4.24.-]	525	535	449	560	320	324	374	323	725	644	637	754
K07262 serine-type D-Ala-D-Ala endopeptidase (penicillin-binding protein 7) [EC:3.4.21.-]	1929	1981	1959	1940	1148	1242	1197	1245	1207	1224	1228	1228
K07263 zinc protease [EC:3.4.24.-]	6389	6457	6391	6431	8190	8264	8277	8382	6039	5494	5684	5420
K07284 sortase A [EC:3.4.22.70]	239	220	234	270	76	142	88	101	356	440	352	402
K07386 putative endopeptidase [EC:3.4.24.-]	1875	2064	2107	1953	5104	4971	5077	5037	4074	3876	3854	3675
K07387 metalloprotease [EC:3.4.24.-]	1875	1812	1737	1753	2475	2545	2418	2552	2068	2023	1947	1970
K07991 archaeal preflagellin peptidase FlaK [EC:3.4.23.52]	0	0	0	0	0	2	2	0	0	3	3	4
K08258 staphopain A [EC:3.4.22.48]	87	60	77	57	3	4	1	7	66	100	59	82
K08315 hydrogenase 3 maturation protease [EC:3.4.23.51]	312	307	266	295	182	195	196	223	399	401	420	447
K08326 aminopeptidase [EC:3.4.11.-]	9	5	6	8	6	12	15	9	27	39	39	48
K08566 plasminogen activator [EC:3.4.23.48]	0	0	0	0	0	0	2	0	0	2	0	4
K08600 sortase B [EC:3.4.22.71]	625	623	645	666	196	188	234	174	663	712	681	750
K08602 oligoendopeptidase F [EC:3.4.24.-]	601	636	703	641	219	265	247	215	608	652	620	607
K08640 zinc D-Ala-D-Ala carboxypeptidase [EC:3.4.17.14]	23	20	12	34	31	28	37	18	61	39	72	71
K08651 thermitase [EC:3.4.21.66]	3	2	0	2	2	5	3	5	0	0	0	2

K08659 dipeptidase [EC:3.4.-.-]	78	86	97	70	111	149	146	112	98	105	115	101
K08675 ATP-dependent Lon protease [EC:3.4.21.53]	1	1	2	0	0	0	0	3	0	1	0	1
K08676 tricorn protease [EC:3.4.21.-]	432	429	472	380	594	614	591	607	39	82	76	98
K08956 AFG3 family protein [EC:3.4.24.-]	0	0	1	1	0	0	1	0	1	0	1	0
K09612 alkaline phosphatase isozyme conversion protein [EC:3.4.11.-]	654	690	565	665	465	463	486	424	838	893	842	877
K09933 MtfA peptidase	1509	1478	1471	1503	858	940	830	934	1024	1037	1000	960
K11873 ubiquitin carboxyl-terminal hydrolase 10 [EC:3.4.19.12]	0	0	0	0	0	0	0	0	1	0	0	0
K12940 aminobenzoyl-glutamate utilization protein A	943	959	980	936	529	600	494	577	1501	1484	1403	1592
K12941 aminobenzoyl-glutamate utilization protein B	920	955	853	936	260	320	257	301	1455	1445	1389	1514
K13049 carboxypeptidase PM20D1 [EC:3.4.17.-]	391	380	420	385	123	135	105	133	755	730	702	782
K13274 cell wall-associated protease [EC:3.4.21.-]	0	0	1	0	0	0	0	0	1	0	2	0
K13275 major intracellular serine protease [EC:3.4.21.-]	1	0	1	0	0	2	1	0	0	0	0	0
K13277 minor extracellular protease Epr [EC:3.4.21.-]	1	1	0	1	0	0	0	1	3	1	2	3
K13282 cyanophycinase [EC:3.4.15.6]	2	0	0	4	0	0	0	0	2	3	4	9
K13617 protein phosphatase methylesterase 1 [EC:3.1.1.89]	0	1	0	0	0	0	0	0	0	0	0	0
K13694 murein DD-endopeptidase / murein LD-carboxypeptidase [EC:3.4.-.- 3.4.17.13]	626	682	669	680	1330	1339	1365	1374	1176	1239	1166	1212
K13695 probable lipoprotein NlpC	306	264	250	325	169	226	215	220	454	422	435	474
K14358 secernin	33	26	30	27	18	29	22	5	30	24	49	31
K14664 IAA-amino acid hydrolase [EC:3.5.1.-]	50	49	37	37	15	18	14	10	29	35	45	40
K14665 amidohydrolase [EC:3.5.1.-]	99	105	107	96	66	76	79	100	150	130	118	160
K16203 D-amino peptidase [EC:3.4.11.-]	307	278	286	296	38	42	52	64	279	295	254	285
K16291 L,D-transpeptidase ErfK/SrfK	1765	1865	1835	1934	1045	1039	1088	1173	1196	1150	1097	1200
K16922 putative peptide zinc metalloprotease protein	464	402	417	446	713	704	673	661	44	63	60	66
K17733 peptidoglycan LD-endopeptidase CwlK [EC:3.4.-.-]	334	319	389	343	32	18	28	36	53	54	44	48
K17734 serine protease AprX [EC:3.4.21.-]	892	797	761	819	103	107	109	103	529	629	516	618
K18015 deubiquitinase [EC:3.4.22.-]	0	0	0	0	8	6	7	4	0	1	3	1
K18530 putative aminopeptidase FrvX [EC:3.4.11.-]	0	14	7	0	6	5	0	0	0	0	13	0
K18545 streptogrisin B [EC:3.4.21.81]	1	0	0	0	0	0	0	0	0	0	0	0
K18574 Xaa-Xaa-Pro tripeptidyl-peptidase [EC:3.4.14.12]	0	0	0	0	0	0	0	0	0	0	0	1
K18988 serine-type D-Ala-D-Ala carboxypeptidase/endopeptidase [EC:3.4.16.4 3.4.21.-]	667	674	636	719	467	538	521	514	873	937	834	1066
K19223 peptidoglycan DL-endopeptidase LytF [EC:3.4.-.-]	0	0	0	0	0	0	0	0	0	1	0	0
K19224 peptidoglycan DL-endopeptidase LytE [EC:3.4.-.-]	2	1	0	3	0	0	0	0	0	0	0	0
K19225 rhomboid protease GluP [EC:3.4.21.105]	353	338	399	368	76	61	147	107	287	300	320	298
K19234 L,D-transpeptidase YnhG	508	548	466	562	351	389	342	351	715	748	827	736
K19235 L,D-transpeptidase YbiS	580	550	585	605	365	413	410	378	804	916	820	888
K19303 murein DD-endopeptidase [EC:3.4.-.-]	369	352	381	376	247	283	252	256	505	494	484	516
K19304 murein DD-endopeptidase [EC:3.4.24.-]	764	809	732	753	484	502	565	436	1041	1053	1015	1025
K19689 aminopeptidase [EC:3.4.11.-]	220	221	231	227	118	120	109	115	369	397	361	350
K19701 aminopeptidase YwaD [EC:3.4.11.6 3.4.11.10]	4	3	2	1	0	0	2	0	4	9	2	4
K20609 putative aminopeptidase [EC:3.4.11.-]	7	3	3	1	30	20	18	10	13	17	14	12
K20742 gamma-D-glutamyl-L-lysine dipeptidyl-peptidase [EC:3.4.14.13]	8	3	1	4	4	1	1	0	0	1	5	1
K21429 immunomodulating metalloprotease	4119	4025	4030	4143	2436	2246	2231	2494	1320	1279	1297	1231
K21470 L,D-transpeptidase YcbB	1717	1737	1747	1725	3271	3384	3089	3146	3391	3151	3376	3247
K21471 peptidoglycan DL-endopeptidase CwlO [EC:3.4.-.-]	867	841	961	899	204	206	199	146	826	922	839	940
K21472 peptidoglycan LD-endopeptidase LytH [EC:3.4.-.-]	1764	1799	1830	1889	715	731	708	652	994	1137	1006	970
K21473 peptidoglycan DL-endopeptidase RipA [EC:3.4.-.-]	45	37	51	36	7	5	12	6	19	16	18	20
K21604 jasmonoyl-L-amino acid hydrolase [EC:3.5.1.127]	35	21	20	19	7	4	2	7	10	10	20	14
K21613 uncharacterized hydrolase [EC:3.-.-.-]	651	676	666	657	403	457	462	392	873	863	914	941
K22684 metacaspase-1 [EC:3.4.22.-]	5	5	3	5	21	17	16	10	13	16	10	7
ko01003 Glycosyltransferases	81413	80648	79395	80901	66846	67819	66539	67375	64413	65616	63893	65524
K00712 poly(glycerol-phosphate) alpha-glucosyltransferase [EC:2.4.1.52]	1	4	3	2	14	17	5	12	4	5	7	4
K00752 hyaluronan synthase [EC:2.4.1.212]	3	5	2	5	0	0	0	0	8	12	8	17
K02852 UDP-N-acetyl-D-mannosaminouronate:lipid I N-acetyl-D-mannosaminouronosyltransferase [EC:2.4.1.180]	560	511	479	515	365	373	357	363	635	726	589	642
K02853 enterobacterial common antigen polymerase [EC:2.4.1.-]	809	899	795	846	605	632	657	614	1127	1164	1181	1109
K03669 membrane glycosyltransferase [EC:2.4.1.-]	5774	5722	5474	5489	3213	3386	3125	3361	3286	3310	3247	3280
K05841 sterol 3beta-glucosyltransferase [EC:2.4.1.173]	0	0	0	1	0	0	0	1	0	0	0	0
K07272 rhamnosyltransferase [EC:2.4.1.-]	0	1	11	7	2	5	4	2	4	15	8	7
K12246 poly-alpha-2,8 sialosyl sialyltransferase [EC:2.4.-.-]	2	2	0	3	1	2	5	2	42	46	47	44
K12582 dTDP-N-acetylfucosamine:lipid II N-acetylfucosaminyltransferase [EC:2.4.1.325]	708	674	674	725	513	549	543	522	952	1073	1006	938
K12984 (heptosyl)LPS beta-1,4-glucosyltransferase [EC:2.4.1.-]	457	513	487	553	316	328	323	307	576	681	585	713
K12987 alpha-1,6-rhamnosyltransferase [EC:2.4.1.-]	1395	1444	1276	1335	791	721	757	811	387	395	405	424
K12988 alpha-1,3-rhamnosyltransferase [EC:2.4.1.-]	1512	1450	1517	1459	845	886	809	891	526	454	438	470
K12989 mannosyltransferase [EC:2.4.1.-]	0	0	0	1	0	0	0	0	1	3	0	0
K12993 alpha-1,2-mannosyltransferase/rhamnosyltransferase [EC:2.4.1.-]	2502	2467	2362	2314	1352	1344	1294	1437	815	745	719	759
K12994 mannosyl-N-acetyl-alpha-D-glucosaminyl-diphospho-ditrans,octacis-undecaprenol 3-alpha-mannosyltransferase / alpha-1,3-rhamnosyltransferase [EC	1773	1621	1670	1624	918	947	883	1001	528	487	546	512
K12995 N-acetyl-alpha-D-glucosaminyl-diphospho-ditrans,octacis-undecaprenol 3-alpha-mannosyltransferase / rhamnosyltransferase [EC:2.4.1.348 2.4.1.-]	1877	1871	1885	1945	1994	1942	1898	1889	1242	1149	1209	1134
K12996 rhamnosyltransferase [EC:2.4.1.-]	8	5	8	11	51	59	49	63	30	21	41	26
K12997 rhamnosyltransferase [EC:2.4.1.-]	25	33	31	44	8	10	12	10	3	7	1	6
K12999 glucosyltransferase [EC:2.4.1.-]	2	0	4	0	1	2	0	0	0	1	1	3
K13002 glycosyltransferase [EC:2.4.1.-]	101	100	110	89	100	122	156	136	19	11	12	26
K13004 galacturonosyltransferase [EC:2.4.1.-]	67	69	69	70	30	24	15	16	64	84	100	95
K13007 Fuc2NAc and GlcNAc transferase [EC:2.4.1.-]	1379	1391	1452	1529	765	789	868	858	467	453	411	414
K13678 1,2-diacylglycerol-3-alpha-glucose alpha-1,2-galactosyltransferase [EC:2.4.1.-]	24	55	40	29	7	17	9	5	96	95	86	90
K13683 putative colanic acid biosynthesis glycosyltransferase [EC:2.4.-.-]	132	133	147	175	189	201	181	198	56	35	45	52
K13684 putative colanic acid biosynthesis glycosyltransferase [EC:2.4.-.-]	268	254	259	296	320	354	352	336	70	76	61	74
K13688 cyclic beta-1,2-glucan synthetase [EC:2.4.1.-]	19	17	10	11	3	9	0	3	20	19	13	17
K16555 succinoglycan biosynthesis protein ExoO [EC:2.4.-.-]	0	0	0	0	1	0	1	0	1	0	0	0
K16698 teichuronic acid biosynthesis glycosyltransferase TuaG [EC:2.4.-.-]	93	97	103	96	37	30	28	25	129	114	90	101
K16702 amylovoran biosynthesis glycosyltransferase AmsE [EC:2.4.-.-]	1	1	2	0	0	0	0	0	3	9	10	5
K16703 colanic acid/amylovoran biosynthesis glycosyltransferase [EC:2.4.-.-]	251	243	225	259	311	370	350	342	45	53	87	53
K17248 N-acetylgalactosamine-N,N'-diacetylbacillosaminyl-diphospho-undecaprenol 4-alpha-N-acetylgalactosaminyltransferase [EC:2.4.1.291]	0	0	0	0	0	0	0	1	0	0	0	0
K17249 GalNAc-alpha-(1->4)-GalNAc-alpha-(1->3)-diNAcBac-PP-undecaprenol alpha-1,4-N-acetyl-D-galactosaminyltransferase [EC:2.4.1.292]	1	0	0	0	0	0	0	0	0	0	0	0
K19354 heptose III glucuronosyltransferase [EC:2.4.1.-]	8	9	0	5	14	27	12	10	60	50	56	79
K19423 glycosyltransferase EpsE [EC:2.4.-.-]	4	6	12	9	4	2	5	1	4	2	2	2
K19424 glycosyltransferase EpsF [EC:2.4.-.-]	53	46	31	45	19	22	16	21	37	35	25	31
K19425 glycosyltransferase EpsH [EC:2.4.-.-]	20	19	19	28	27	26	23	23	11	10	13	13
K20151 alpha(1,3/1,4) fucosyltransferase [EC:2.4.1.65 2.4.1.152]	53	58	67	71	153	123	146	128	133	114	116	165
K20926 cellobiuronic acid synthase [EC:2.4.1.-]	7	6	3	7	3	5	3	1	6	8	3	12
K21306 dolichyl-phosphooligosaccharide-protein glycotransferase [EC:2.4.99.21]	0	8	0	1	3	0	0	3	19	20	4	31
K21363 UDP-Glc:alpha-D-GlcNAc-glucosaminyl-diphosphoundecaprenol beta-1,3-glucosyltransferase [EC:2.4.1.305]	0	0	0	0	0	0	0	0	0	0	1	0
K21365 UDP-GalNAc:alpha-D-GalNAc-diphosphoundecaprenol alpha-1,3-N-acetylgalactosaminyltransferase [EC:2.4.1.306]	0	2	0	1	0	2	0	0	0	0	3	1
K21367 GDP-Fuc:beta-D-Gal-1,3-alpha-D-GalNAc-1,3-alpha-GalNAc-diphosphoundecaprenol alpha-1,2-fucosyltransferase [EC:2.4.1.308]	1	1	3	0	1	0	0	1	0	0	1	0
K22708 poly(ribitol-phosphate) beta-N-acetylglucosaminyltransferase [EC:2.4.1.355]	0	0	0	0	0	0	0	1	0	0	0	0
ko01005 Lipopolysaccharide biosynthesis proteins	106121	105764	104351	106555	92358	93522	88724	90743	84571	85176	84139	85816
K00996 undecaprenyl-phosphate galactose phosphotransferase [EC:2.7.8.6]	176	186	169	186	22	20	19	15	185	199	178	208
K05789 chain length determinant protein (polysaccharide antigen chain regulator)	1372	1425	1609	1488	846	894	792	821	466	501	505	517
K08280 lipopolysaccharide O-acetyltransferase [EC:2.3.1.-]	0	0	0	0	2	2	0	0	2	0	0	0
K13006 UDP-perosamine 4-acetyltransferase [EC:2.3.1.-]	0	0	0	1	4	1	0	0	1	2	1	3
K13012 O-antigen biosynthesis protein WbqP	5	4	7	10	9	18	8	14	79	62	51	56
K13013 O-antigen biosynthesis protein WbqV	0	0	0	0	0	0	0	0	1	0	0	0
K19714 3-deoxy-alpha-D-manno-octulosonate 8-oxidase [EC:1.1.3.48]	32	19	15	22	1	6	9	3	14	18	29	20
K19715 8-amino-3,8-dideoxy-alpha-D-manno-octulosonate transaminase [EC:2.6.1.109]	0	0	0	0	0	0	0	1	0	1	0	3
K20444 O-antigen biosynthesis protein [EC:2.4.1.-]	3451	3254	3218	3467	1715	1947	1683	1721	2063	2399	2174	2365
K20534 polyisoprenyl-phosphate glycosyltransferase [EC:2.4.-.-]	2786	2690	2512	2767	1356	1267	1227	1395	1040	1060	1003	1086
ko01011 Peptidoglycan biosynthesis and degradation proteins	148799	148842	148108	148413	144175	147682	146254	145208	151487	152531	150435	151970
K03806 N-acetyl-anhydromuramoyl-L-alanine amidase [EC:3.5.1.28]	1257	1321	1334	1370	793	854	743	744	834	832	819	804
K03980 putative peptidoglycan lipid II flippase	1049	946	983	978	785	744	899	831	1328	1457	1435	1417
K06078 murein lipoprotein	52	46	54	45	35	31	36	48	68	65	61	70
K08304 membrane-bound lytic murein transglycosylase A [EC:4.2.2.-]	2578	2546	2307	2447	1643	1585	1555	1665	1517	1503	1298	1509
K08305 membrane-bound lytic murein transglycosylase B [EC:4.2.2.-]	6399	6316	5935	6045	3613	3608	3456	3555	2774	2766	2568	2806
K08306 membrane-bound lytic murein transglycosylase C [EC:4.2.2.-]	660	636	677	744	462	539	457	468	1027	1055	1023	995
K08307 membrane-bound lytic murein transglycosylase D [EC:4.2.2.-]	3954	4082	3947	4012	4535	4958	4568	4863	4119	4108	4071	4031
K08308 membrane-bound lytic murein transglycosylase E [EC:4.2.2.-]	341	342	309	334	225	247	250	210	434	458	528	464
K08309 soluble lytic murein transglycosylase [EC:4.2.2.-]	4161	4061	4057	4074	2548	2539	2463	2631	2696	2668	2511	2775
K11066 N-acetylmuramoyl-L-alanine amidase [EC:3.5.1.28]	1389	1384	1358	1305	823	827	862	808	896	896	906	1006
K18691 membrane-bound lytic murein transglycosylase F [EC:4.2.2.-]	3857	3880	3906	3752	4371	4449	4389	4619	3985	3831	3899	3760
K21467 penicillin-binding protein 3	618	579	636	643	195	184	225	230	716	713	707	734
K21468 penicillin-binding protein 4B	2	1	1	2	0	0	0	0	0	0	0	0
ko01004 Lipid biosynthesis proteins	107647	107776	105211	106853	93393	92039	91907	94053	91152	90904	89886	91401
K00666 fatty-acyl-CoA synthase [EC:6.2.1.-]	13534	13471	13154	13175	10580	10545	10079	11129	6811	6501	6701	6805
K12431 phthioceranic/hydroxyphthioceranic acid synthase [EC:2.3.1.287]	0	0	0	0	0	1	0	0	0	0	0	0

K12436 polyketide synthase 12	1	1	0	0	0	0	2	1	2	2	0	1
K12441 phthiocerol/phenolphthiocerol synthesis type-I polyketide synthase B	0	0	0	0	0	0	0	0	0	0	0	1
K12443 phthiocerol/phenolphthiocerol synthesis type-I polyketide synthase D	0	0	0	0	0	2	0	0	1	0	0	1
K12500 thioesterase III [EC:3.1.2.-]	226	213	210	212	173	197	150	175	314	306	303	300
K12507 acyl-CoA synthetase [EC:6.2.1.-]	1121	1025	1003	1199	746	864	756	735	607	677	616	660
K13611 polyketide synthase PksJ	230	202	236	214	43	38	64	37	4	4	3	9
K13614 polyketide synthase PksN	20	16	23	17	18	9	20	10	7	3	9	15
ko01008 Polyketide biosynthesis proteins	32760	31711	29474	30693	15551	15491	14954	15681	27362	27072	26152	27715
K05374 yersiniabactin synthetase, thioesterase component	316	272	276	262	107	92	91	73	571	625	621	644
K12242 pyochelin biosynthetic protein PchC	1242	1262	1098	1186	614	572	659	700	436	322	383	394
K15395 hybrid polyketide synthase / nonribosomal peptide synthetase FtdB	0	0	0	0	0	0	0	0	0	0	1	0
K15644 coronafacic acid polyketide synthase Cfa6	0	0	1	0	0	0	0	0	1	0	0	0
K16167 alkylresorcinol/alkylpyrone synthase	3	3	2	0	0	0	0	0	0	0	0	0
ko01006 Prenyltransferases	17179	16907	17291	17177	19862	19971	20297	19622	18072	18543	18348	18473
ko01007 Amino acid related enzymes	214733	214798	216352	217549	215793	218266	216152	215217	213973	214937	213659	214340
K01894 glutamyl-Q tRNA(Asp) synthetase [EC:6.1.1.-]	845	870	743	838	574	587	566	603	880	875	880	887
K02805 dTDP-4-amino-4,6-dideoxygalactose transaminase [EC:2.6.1.59]	810	811	730	867	581	637	644	628	1307	1313	1244	1311
K03918 L-lysine 6-transaminase [EC:2.6.1.36]	0	3	0	0	0	0	0	0	2	0	1	4
K09469 2-aminoethylphosphonate-pyruvate transaminase	1	1	0	2	1	2	1	0	2	5	3	7
K10907 aminotransferase [EC:2.6.1.-]	857	901	966	948	387	395	421	346	962	958	984	1002
K14261 alanine-synthesizing transaminase [EC:2.6.1.-]	3003	2884	2815	2903	1682	1769	1641	1842	1819	1690	1727	1872
K14287 methionine transaminase [EC:2.6.1.88]	2671	2695	2605	2714	1498	1677	1599	1631	1569	1556	1663	1710
ko00199 Cytochrome P450	0	1	0	0	12	15	5	5	23	25	29	24
K15629 fatty-acid peroxygenase [EC:1.11.2.4]	0	1	0	0	12	15	5	5	23	23	29	24
K21119 cytochrome P450 family 130	0	0	0	0	0	0	0	0	0	2	0	0
ko00194 Photosynthesis proteins	26142	26229	27048	26875	24434	24776	24578	24154	24174	24255	24515	24112
K02116 ATP synthase protein I	1064	987	1040	1044	541	583	555	517	615	593	625	641
Protein families: genetic information processing	1747091	1747644	1751205	1755209	1724736	1740347	1733333	1727195	1747550	1755511	1742577	1751148
ko03000 Transcription factors	267307	264211	259048	263978	192182	194183	190278	194023	243829	248530	239279	248388
K00375 GntR family transcriptional regulator / MocR family aminotransferase	9309	9057	8594	8770	5533	6034	5760	5940	6407	6969	6832	6897
K01420 CRP/FNR family transcriptional regulator, anaerobic regulatory protein	915	958	938	957	594	719	649	622	1045	1013	1019	1034
K01926 redox-sensing transcriptional repressor	1048	1051	1164	1089	1553	1544	1630	1537	1544	1535	1526	1594
K01994 LuxR family transcriptional regulator, transcriptional regulator of spore coat protein	0	0	0	0	0	0	0	0	0	2	0	3
K02019 molybdate transport system regulatory protein	1759	1695	1606	1642	1053	1053	1001	1031	1222	1209	1084	1227
K02043 GntR family transcriptional regulator, phosphonate transport system regulatory protein	1247	1213	1124	1115	571	562	586	582	888	910	878	962
K02076 Fur family transcriptional regulator, zinc uptake regulator	21	30	38	29	5	5	8	6	22	25	24	29
K02081 DeoR family transcriptional regulator, aga operon transcriptional repressor	441	377	391	366	526	607	559	517	129	139	129	164
K02099 AraC family transcriptional regulator, arabinose operon regulatory protein	972	952	836	1021	555	537	571	593	1444	1513	1410	1574
K02103 GntR family transcriptional regulator, arabinose operon transcriptional repressor	444	456	435	469	118	85	115	92	416	470	387	413
K02167 TetR/AcrR family transcriptional regulator, transcriptional repressor of bet genes	452	396	369	385	177	190	196	178	548	542	538	545
K02430 DeoR family transcriptional regulator, L-fucose operon activator	446	498	463	488	274	299	318	292	542	581	511	611
K02436 DeoR family transcriptional regulator, galactitol utilization operon repressor	2	6	0	3	3	9	9	16	34	37	39	50
K02443 glycerol uptake operon antiterminator	214	198	211	265	113	133	109	99	572	584	559	565
K02444 DeoR family transcriptional regulator, glycerol-3-phosphate regulon repressor	2405	2299	2339	2391	1580	1724	1588	1556	1733	1634	1528	1741
K02468 DeoR family transcriptional regulator, glucitol operon repressor	446	452	399	481	347	270	327	288	564	638	614	668
K02508 AraC family transcriptional regulator, 4-hydroxyphenylacetate 3-monooxygenase operon regulatory protein	609	614	614	629	267	250	280	262	842	795	820	865
K02521 LysR family transcriptional regulator, positive regulator for ilvC	628	644	563	699	414	501	434	434	838	881	861	880
K02525 LacI family transcriptional regulator, kdg operon repressor	1294	1214	1176	1171	751	780	798	798	906	901	922	955
K02529 LacI family transcriptional regulator	15200	15226	15889	15612	18828	18535	19422	18441	25315	25807	25165	25397
K02530 DeoR family transcriptional regulator, lactose phosphotransferase system repressor	102	93	76	84	39	33	32	23	115	132	119	117
K02531 transcriptional antiterminator	1	4	8	0	6	0	0	0	0	0	7	0
K02538 activator of the mannose operon, transcriptional antiterminator	485	537	509	560	560	634	589	522	288	363	296	302
K02562 mannitol operon repressor	357	391	287	388	240	316	272	280	535	479	468	568
K02565 N-acetylglucosamine repressor	755	714	672	704	462	526	483	493	1003	1034	925	1058
K02616 phenylacetic acid degradation operon negative regulatory protein	336	336	297	358	89	91	77	102	649	733	553	662
K02623 LysR family transcriptional regulator, pca operon transcriptional activator	2094	1934	1843	1834	1044	1014	940	1142	754	631	590	623
K02624 IclR family transcriptional regulator, pca regulon regulatory protein	1333	1389	1333	1337	505	530	460	460	1800	1921	1929	1984
K02647 carbohydrate diacid regulator	3542	3281	3274	3415	1611	1548	1451	1536	2814	2985	2706	2889
K02688 transcriptional regulator, propionate catabolism operon regulatory protein	2419	2375	2375	2296	1329	1282	1200	1335	789	809	801	779
K02854 AraC family transcriptional regulator, L-rhamnose operon transcriptional activator RhaR	541	562	526	550	379	419	397	441	842	877	744	858
K02855 AraC family transcriptional regulator, L-rhamnose operon regulatory protein RhaS	643	627	558	637	381	442	461	378	711	676	650	770
K03402 transcriptional regulator of arginine metabolism	938	1002	1025	1003	1326	1408	1364	1216	1418	1484	1462	1469
K03435 LacI family transcriptional regulator, fructose operon transcriptional repressor	2642	2519	2343	2462	1260	1224	1308	1438	2130	1987	2055	2111
K03436 DeoR family transcriptional regulator, fructose operon transcriptional repressor	390	361	358	398	59	70	66	63	687	786	739	799
K03477 DeoR family transcriptional regulator, ulaG and ulaABCDEF operon transcriptional repressor	430	507	420	475	333	419	300	349	613	632	624	622
K03480 transcriptional antiterminator	14	15	15	21	4	26	18	21	39	41	26	37
K03481 RpiR family transcriptional regulator, glv operon transcriptional regulator	48	35	53	59	35	46	18	34	68	90	63	85
K03482 GntR family transcriptional regulator, glv operon transcriptional regulator	913	822	834	874	445	428	389	430	1025	1186	1084	1201
K03483 mannitol operon transcriptional antiterminator	170	141	124	177	24	33	33	45	163	172	121	177
K03484 LacI family transcriptional regulator, sucrose operon repressor	1877	1921	1864	1948	596	621	631	614	1817	2005	1937	1965
K03485 LacI family transcriptional regulator, trehalose operon repressor	602	663	621	622	455	425	473	446	839	911	808	950
K03486 GntR family transcriptional regulator, trehalose operon transcriptional repressor	509	446	463	471	89	83	76	74	434	428	456	435
K03487 LacI family transcriptional regulator, asc operon repressor	833	959	876	872	780	795	779	763	895	901	836	911
K03488 beta-glucoside operon transcriptional antiterminator	1341	1343	1304	1307	783	849	640	728	1215	1409	1253	1326
K03489 GntR family transcriptional regulator, transcriptional regulator of bglA	4	0	0	3	7	0	4	1	4	4	2	3
K03490 AraC family transcriptional regulator, dual regulator of chb operon	522	523	517	550	304	324	326	331	688	672	662	743
K03491 lichenan operon transcriptional antiterminator	706	659	752	671	108	111	99	94	518	729	623	540
K03492 GntR family transcriptional regulator	429	494	458	446	355	360	326	302	575	601	611	617
K03493 transcriptional antiterminator	2	0	0	0	0	0	0	0	0	0	0	0
K03497 ParB family transcriptional regulator, chromosome partitioning protein	15665	15894	15757	15732	16344	16071	16291	16292	17335	17504	17321	17147
K03556 LuxR family transcriptional regulator, maltose regulon positive regulatory protein	7906	7558	7507	7853	4618	4863	4585	4834	6551	6701	6608	6660
K03576 LysR family transcriptional regulator, regulator for metE and metH	1590	1569	1516	1598	967	933	849	928	1705	1765	1515	1662
K03577 TetR/AcrR family transcriptional regulator, acrAB operon repressor	254	254	227	287	213	235	220	247	292	308	273	283
K03603 GntR family transcriptional regulator, negative regulator for fad regulon and positive regulator of fabA	383	451	394	405	275	340	268	273	580	621	579	587
K03604 LacI family transcriptional regulator, purine nucleotide synthesis repressor	647	622	590	579	382	450	410	477	884	843	874	895
K03705 heat-inducible transcriptional repressor	665	576	645	689	234	210	235	234	563	665	599	610
K03706 transcriptional pleiotropic repressor	375	380	400	433	94	91	104	74	415	454	398	396
K03708 transcriptional regulator of stress and heat shock response	59	61	66	64	31	45	32	39	48	67	80	69
K03709 DtxR family transcriptional regulator, Mn-dependent transcriptional regulator	31	44	37	48	16	5	9	7	41	62	50	44
K03710 GntR family transcriptional regulator	2062	1893	2166	2014	2147	2146	2146	2076	2555	2516	2466	2615
K03711 Fur family transcriptional regulator, ferric uptake regulator	1984	2028	2114	2121	2719	2748	2758	2728	2502	2496	2515	2459
K03712 MarR family transcriptional regulator, multiple antibiotic resistance protein MarR	317	283	329	361	313	307	276	312	287	370	368	367
K03713 MerR family transcriptional regulator, glutamine synthetase repressor	16	14	26	23	4	4	5	10	17	39	26	19
K03717 LysR family transcriptional regulator, transcriptional activator of nhaA	515	464	452	483	424	395	368	352	588	612	583	641
K03718 Lrp/AsnC family transcriptional regulator, regulator for asnA, asnC and gidA	912	804	882	912	2303	2351	2342	2291	1691	1681	1785	1643
K03719 Lrp/AsnC family transcriptional regulator, leucine-responsive regulatory protein	4752	4699	4659	4629	3543	3579	3433	3728	2945	2961	2735	2940
K03720 TrpR family transcriptional regulator, trp operon repressor	280	228	221	289	160	193	143	136	353	385	349	348
K03721 transcriptional regulator of aroF, aroG, tyrA and aromatic amino acid transport	3259	3225	3068	3243	1801	1903	1878	1867	2029	1954	1871	1916
K03755 AraC family transcriptional regulator, transcriptional activator of adiA	2	4	4	1	8	16	17	13	73	86	39	48
K03764 MetJ family transcriptional regulator, methionine regulon repressor	289	220	269	229	143	179	178	140	389	412	340	415
K03765 transcriptional activator of cad operon	524	524	532	578	220	192	189	187	1145	1240	1092	1273
K03892 ArsR family transcriptional regulator, arsenate/arsenite/antimonite-responsive transcriptional repressor	768	801	725	782	640	574	602	628	395	487	452	423
K03973 phage shock protein C	186	201	192	214	123	133	107	137	262	301	251	287
K03974 psp operon transcriptional activator	506	562	455	549	331	407	365	337	726	702	791	778
K04033 AraC family transcriptional regulator, ethanolamine operon transcriptional activator	462	405	393	414	138	131	105	144	888	819	844	947
K04769 AbrB family transcriptional regulator, stage V sporulation protein T	413	381	389	353	96	130	97	92	297	356	293	326
K05311 central glycolytic genes regulator	107	114	93	114	40	38	41	35	140	190	180	172
K05346 deoxyribonucleoside regulator	95	67	65	78	24	43	58	36	131	135	127	188
K05372 AraC family transcriptional regulator	881	801	852	907	364	318	379	326	990	1071	918	1021
K05499 LacI family transcriptional regulator, repressor for deo operon, udp, cdd, tsx, nupC, and nupG	897	901	885	924	500	600	547	501	1311	1331	1177	1285
K05501 TetR/AcrR family transcriptional regulator	448	418	442	449	323	362	335	322	610	689	600	652
K05527 BolA family transcriptional regulator, general stress-responsive regulator	693	604	658	655	370	416	390	431	438	446	444	449
K05596 LysR family transcriptional regulator, chromosome initiation inhibitor	2185	2005	2022	2015	1295	1169	1227	1277	1244	1254	1151	1296
K05785 transcriptional antiterminator RfaH	325	303	315	280	221	262	215	214	441	481	432	477
K05798 LysR family transcriptional regulator, transcriptional activator for leuABCD operon	631	586	559	580	444	450	426	378	818	828	821	865
K05799 GntR family transcriptional regulator, transcriptional repressor for pyruvate dehydrogenase complex	1913	1776	1829	1864	1126	1087	990	1157	1770	1741	1701	1701
K05800 Lrp/AsnC family transcriptional regulator	907	955	900	848	525	492	525	544	589	681	600	534
K05804 AraC family transcriptional regulator, mar-sox-rob regulon activator	811	796	848	787	568	591	543	559	1148	1174	1103	1115
K05817 LysR family transcriptional regulator, hca operon transcriptional activator	1	1	1	0	5	10	6	3	1	5	2	5
K05818 IclR family transcriptional regulator, mhp operon transcriptional activator	366	314	301	303	79	80	113	94	635	593	576	635
K05836 GntR family transcriptional regulator, histidine utilization repressor	469	470	410	486	294	350	312	339	656	687	602	716

K05880 transcriptional activator for dhaKLM operon	1099	1168	1021	1091	748	740	710	702	1570	1670	1537	1688
K06075 MarR family transcriptional regulator, transcriptional regulator for hemolysin	1202	1224	1185	1150	749	881	836	901	837	883	834	862
K06140 regulator of nucleoside diphosphate kinase	869	869	888	859	505	513	527	532	581	578	579	564
K06145 LacI family transcriptional regulator, gluconate utilization system Gnt-I transcriptional repressor	946	846	819	983	781	782	804	701	940	993	943	988
K06146 LacI family transcriptional regulator, gluconate utilization system Gnt-II transcriptional activator	194	216	229	202	289	314	288	348	53	72	69	47
K06283 putative DeoR family transcriptional regulator, stage III sporulation protein D	171	159	152	154	33	38	33	42	99	143	110	128
K06284 AbrB family transcriptional regulator, transcriptional pleiotropic regulator of transition state genes	36	27	45	30	21	27	34	23	21	27	23	24
K06314 prespore-specific regulator	0	0	0	2	0	0	0	0	0	0	0	0
K06599 AraC family transcriptional regulator, chemosensory pili system protein ChpD	1354	1370	1297	1287	691	695	708	754	480	392	402	380
K06608 DeoR family transcriptional regulator, myo-inositol catabolism operon repressor	54	72	62	61	11	4	2	4	129	95	94	113
K06714 arginine utilization regulatory protein	2205	2290	2324	2166	1238	1197	1210	1227	841	856	779	927
K07110 XRE family transcriptional regulator, fatty acid utilization regulator	0	0	2	2	0	2	0	0	1	3	2	1
K07471 probable transcriptional regulator	7	2	1	3	8	2	6	5	27	32	26	29
K07506 AraC family transcriptional regulator	2283	2435	2223	2323	2497	2490	2469	2449	2270	2133	2111	2142
K07592 LysR family transcriptional regulator, tdc operon transcriptional activator	337	326	344	355	123	107	78	99	784	697	761	788
K07721 ArsR family transcriptional regulator	0	0	0	0	0	2	1	0	4	13	0	2
K07722 CopG family transcriptional regulator, nickel-responsive regulator	313	316	291	332	241	256	234	248	402	435	420	432
K07724 Ner family transcriptional regulator	230	215	224	198	135	174	151	122	288	323	309	313
K07726 putative transcriptional regulator	375	392	386	348	204	196	236	231	555	534	540	600
K07727 putative transcriptional regulator	1650	1610	1691	1666	859	864	785	816	817	786	812	796
K07728 putative transcriptional regulator	0	2	0	1	0	1	2	0	4	3	3	6
K07729 putative transcriptional regulator	680	661	606	603	329	299	270	293	565	607	583	618
K07730 putative transcriptional regulator	0	0	0	1	0	0	1	1	1	11	3	7
K07731 putative transcriptional regulator	0	0	0	0	0	0	0	0	0	0	0	1
K07733 prophage regulatory protein	778	800	838	800	484	523	498	504	938	928	865	784
K07734 transcriptional regulator	1001	972	997	940	548	533	527	640	300	325	291	321
K07735 putative transcriptional regulator	1710	1643	1611	1638	2014	2002	2035	1907	1651	1693	1745	1657
K07736 CarD family transcriptional regulator	197	222	196	190	109	93	129	112	314	341	303	332
K07738 transcriptional repressor NrdR	1386	1371	1278	1391	705	655	657	704	852	959	873	954
K07978 GntR family transcriptional regulator	0	0	0	0	0	0	0	0	1	0	0	0
K07979 GntR family transcriptional regulator	615	547	562	584	162	186	188	160	663	721	657	742
K08277 transcriptional activator CaiF	98	88	80	92	134	147	136	145	41	48	23	27
K08365 MerR family transcriptional regulator, mercuric resistance operon regulatory protein	940	860	973	945	657	697	741	642	254	225	218	267
K09017 TetR/AcrR family transcriptional regulator	2680	2595	2579	2547	1491	1509	1511	1731	1198	1234	1095	1211
K09681 LysR family transcriptional regulator, transcription activator of glutamate synthase operon	8	13	6	17	2	0	2	0	23	29	30	28
K09682 MarR family transcriptional regulator, protease production regulatory protein HPr	0	0	0	1	0	0	0	0	0	0	0	0
K09683 hut operon positive regulatory protein	36	29	30	38	0	0	0	0	60	45	63	40
K09684 PucR family transcriptional regulator, purine catabolism regulatory protein	2309	2297	2247	2329	1075	1071	1027	1109	978	1023	872	892
K09685 purine operon repressor	102	103	98	125	30	38	32	23	140	128	132	142
K09720 HTH-type transcriptional regulator, global nitrogen regulator NrpRI	0	5	1	5	2	0	0	2	9	19	7	6
K09825 Fur family transcriptional regulator, peroxide stress response regulator	526	520	595	540	942	1091	994	953	910	867	1021	900
K09826 Fur family transcriptional regulator, iron response regulator	0	0	0	0	0	4	0	2	0	1	0	0
K10711 GntR family transcriptional regulator, frlABCD operon transcriptional regulator	216	181	165	185	271	261	241	204	10	25	25	21
K10778 AraC family transcriptional regulator, regulatory protein of adaptative response / methylated-DNA-[protein]-cysteine methyltransferase [EC:2.1.1.63]	2735	2835	2665	2753	3023	3153	3160	2983	3282	3094	3324	3313
K10947 PadR family transcriptional regulator, regulatory protein PadR	595	625	646	667	677	695	788	685	927	855	892	966
K10972 LysR family transcriptional regulator, transcriptional activator of the allD operon	3	1	1	1	15	13	14	12	39	51	40	38
K10973 IclR family transcriptional regulator, negative regulator of allantoin and glyoxylate utilization operons	2	0	1	1	5	5	12	8	43	60	44	58
K11474 GntR family transcriptional regulator, glc operon transcriptional activator	1331	1272	1239	1355	697	723	700	695	522	462	433	468
K11475 GntR family transcriptional regulator, vanillate catabolism transcriptional regulator	1216	1202	1188	1210	675	698	644	732	403	392	322	425
K11534 DeoR family transcriptional regulator, deoxyribose operon repressor	858	793	824	843	597	649	543	687	688	742	690	802
K11908 sigma-54 dependent transcriptional regulator	2	0	0	1	1	2	1	0	1	1	0	1
K11914 sigma-54 dependent transcriptional regulator	2204	2156	1992	2087	1183	1171	1138	1145	636	672	587	641
K11917 sigma-54 dependent transcriptional regulator	1387	1339	1202	1289	712	771	659	824	408	411	350	362
K11920 AraC family transcriptional regulator	0	2	0	0	10	9	8	9	0	3	1	1
K11921 LysR family transcriptional regulator, cyn operon transcriptional activator	1490	1403	1367	1450	671	672	626	731	1051	1017	1000	1073
K11922 GntR family transcriptional regulator, mannosyl-D-glycerate transport/metabolism system repressor	3	13	12	9	14	32	24	29	11	9	16	9
K11924 DtxR family transcriptional regulator, manganese transport regulator	267	238	269	291	177	218	187	169	356	413	333	408
K11925 SgrR family transcriptional regulator	977	1097	997	1046	722	777	704	763	1284	1509	1336	1456
K11926 sigma factor-binding protein Crl	201	203	231	216	134	172	135	163	311	343	328	321
K12113 LacI family transcriptional regulator, ebg operon repressor	2	5	5	4	16	16	14	7	57	57	78	55
K12146 hydrogenase-4 transcriptional activator	362	356	389	385	594	663	554	597	4	2	3	2
K12243 AraC family transcriptional regulator, transcriptional activator of the genes for pyochelin and ferripyochelin receptors	1694	1614	1363	1358	812	723	705	851	489	431	406	439
K13530 AraC family transcriptional regulator, regulatory protein of adaptative response / methylphosphotriester-DNA alkyltransferase methyltransferase [EC	31	39	37	27	5	5	4	3	25	50	34	39
K13631 AraC family transcriptional regulator, mar-sox-rob regulon activator	425	415	406	405	285	307	295	299	601	707	634	543
K13633 AraC family transcriptional regulator, transcriptional activator FtrA	552	509	518	496	372	329	308	356	712	767	715	745
K13634 LysR family transcriptional regulator, cys regulon transcriptional activator	1743	1650	1649	1647	1023	975	1027	959	1086	1240	1135	1152
K13635 LysR family transcriptional regulator, cys regulon transcriptional activator	542	604	563	538	370	424	406	378	771	850	799	796
K13636 LysR family transcriptional regulator, D-serine deaminase activator	951	1001	915	931	483	628	516	562	1407	1467	1399	1549
K13637 GntR family transcriptional regulator, uxu operon transcriptional repressor	411	387	385	457	294	347	322	326	568	579	586	533
K13638 MerR family transcriptional regulator, Zn(II)-responsive regulator of zntA	273	243	283	294	188	202	210	193	379	372	400	385
K13639 MerR family transcriptional regulator, redox-sensitive transcriptional activator SoxR	430	442	467	464	321	311	301	301	441	422	431	436
K13640 MerR family transcriptional regulator, heat shock protein HspR	8	4	6	2	2	1	3	5	9	7	8	9
K13641 IclR family transcriptional regulator, acetate operon repressor	549	564	536	596	324	401	437	373	729	780	796	760
K13642 CRP/FNR family transcriptional regulator, transcriptional activator FtrB	0	0	0	0	0	0	0	0	2	2	0	0
K13643 Rrf2 family transcriptional regulator, iron-sulfur cluster assembly transcription factor	1029	1074	1121	1060	628	673	639	662	684	671	680	689
K13652 AraC family transcriptional regulator	1440	1448	1509	1406	2899	2870	2959	3080	2712	2637	2795	2586
K13653 AraC family transcriptional regulator	2752	2527	2769	2701	624	631	636	503	3002	3142	2975	3238
K13654 GntR family transcriptional regulator, colanic acid and biofilm gene transcriptional regulator	108	124	118	98	183	159	183	166	9	11	8	11
K13655 HTH-type transcriptional regulator / antitoxin MqsA	1	0	0	3	0	0	0	2	0	0	0	0
K13770 TetR/AcrR family transcriptional regulator, fatty acid metabolism regulator protein	0	0	0	0	2	1	3	1	0	1	0	0
K13772 Rrf2 family transcriptional regulator, iron-responsive regulator	0	0	0	1	0	0	0	0	0	2	0	2
K13794 LysR family transcriptional regulator, regulatory protein for tcuABC	1	4	0	1	2	5	5	2	2	0	1	2
K13928 LysR family transcriptional regulator, malonate utilization transcriptional regulator	2058	2132	1855	1914	1156	1164	1100	1190	1110	1072	1034	1115
K14056 HTH-type transcriptional regulator, repressor for puuD	158	184	197	192	46	49	46	52	422	443	401	360
K14057 LysR family transcriptional regulator, regulator of abg operon	285	255	275	304	89	69	62	87	626	601	524	567
K14063 AraC family transcriptional regulator, positive regulator of tynA and feaB	364	409	364	415	111	101	93	109	755	753	628	794
K14348 GntR family transcriptional regulator, L-lactate dehydrogenase operon regulator	514	523	473	525	383	393	414	386	707	710	775	750
K14414 transcriptional regulatory protein RtcR	3081	2907	2952	2869	2046	2024	2123	2023	922	899	870	908
K15545 transcriptional regulator of PTS gene	624	713	682	710	466	511	452	453	944	937	928	966
K15546 XRE family transcriptional regulator, aerobic/anaerobic benzoate catabolism transcriptional regulator	0	0	0	0	0	0	0	0	1	3	1	0
K15735 GntR family transcriptional regulator, carbon starvation induced regulator	291	246	270	292	82	83	82	87	631	556	540	551
K15773 HTH-type transcriptional regulator / antitoxin HipB	104	93	86	110	24	32	29	25	230	208	220	242
K15782 Lrp/AsnC family transcriptional regulator, regulator of ectoine-degradation genes	0	0	0	1	0	0	0	0	0	3	0	0
K15835 RpiR family transcriptional regulator, murPQ operon repressor	0	1	0	0	12	4	12	10	4	4	7	6
K15836 formate hydrogenlyase transcriptional activator	799	820	848	831	249	255	253	244	1875	1788	1830	1844
K15973 MarR family transcriptional regulator, 2-MHQ and catechol-resistance regulon repressor	15	26	22	21	3	4	1	7	17	27	15	21
K15974 MarR family transcriptional regulator, negative regulator of the multidrug operon emrRAB	303	287	284	359	245	224	259	232	357	479	399	491
K16135 LysR family transcriptional regulator, transcriptional activator for dmlA	340	302	325	347	86	93	102	93	691	658	669	794
K16136 LacI family transcriptional regulator, maltose regulon regulatory protein	510	526	475	531	350	448	395	348	648	678	622	651
K16137 TetR/AcrR family transcriptional regulator, transcriptional repressor for nem operon	2816	2808	2603	2828	1539	1488	1469	1676	1666	1636	1537	1655
K16138 TetR/AcrR family transcriptional regulator, repressor for uid operon	93	95	97	89	47	33	34	47	291	277	264	300
K16326 CRP/FNR family transcriptional regulator, putaive post-exponential-phase nitrogen-starvation regulator	82	108	102	81	154	151	160	152	55	36	46	35
K16516 LysR family transcriptional regulator, regulator for genes of the gallate degradation pathway	539	546	459	542	160	143	165	163	1019	1031	900	1061
K17472 Rrf2 family transcriptional regulator, cysteine metabolism repressor	0	0	0	0	0	0	0	0	3	2	3	5
K17473 sigma-54 dependent transcriptional regulator, dga operon transcriptional activator	146	150	157	185	19	24	17	22	177	201	180	167
K17640 LacI family transcriptional regulator, xylobiose transport system transcriptional regulator	0	0	2	0	0	0	0	0	0	1	0	0
K17736 AraC family transcriptional regulator, carnitine catabolism transcriptional activator	178	162	181	136	91	69	68	107	64	45	39	52
K17737 LysR family transcriptional regulator, carnitine catabolism transcriptional activator	1583	1494	1344	1464	876	885	780	877	960	921	931	891
K18140 TetR/AcrR family transcriptional regulator, acrEF/envCD operon repressor	248	265	264	277	84	97	93	65	496	534	496	579
K18294 TetR/AcrR family transcriptional regulator, mexCD-oprJ operon repressor	375	361	357	379	222	151	212	205	113	123	93	100
K18297 LysR family transcriptional regulator, mexEF-oprN operon transcriptional activator	1431	1406	1343	1457	770	666	584	661	451	429	367	426
K18301 TetR/AcrR family transcriptional regulator, mexJK operon transcriptional repressor	791	755	713	741	303	327	342	355	639	650	581	685
K18325 AraC family of transcriptional regulator, multidrug resistance transcriptional activator	415	364	421	395	278	318	288	273	545	526	537	501
K18476 TetR/AcrR family transcriptional regulator, tetracycline repressor protein	0	2	3	0	1	0	0	0	0	0	1	0
K18531 putative frv operon regulatory protein	0	1	0	0	14	12	12	15	0	6	0	7
K18830 HTH-type transcriptional regulator / antitoxin PezA	3	3	1	1	3	4	3	4	3	10	13	11
K18831 HTH-type transcriptional regulator / antitoxin HigA	1513	1698	1699	1754	1589	1501	1509	1481	1919	1935	1765	1914
K18900 LysR family transcriptional regulator, regulator for bpeEF and oprC	188	239	233	263	46	65	56	49	347	419	357	428
K18907 GntR family transcriptional regulator, regulator for abcA and norABC	12	11	13	11	10	15	13	14	28	27	24	36
K18909 MarR family transcriptional regulator, repressor for mepA	0	0	1	0	0	0	0	2	5	1	3	0
K18918 RHH-type transcriptional regulator, rel operon repressor / antitoxin RelB	105	93	93	106	125	119	110	97	4	0	4	7

K18954 AraC family transcriptional regulator, transcriptional activator of pobA	1636	1678	1646	1698	1068	995	958	1004	1139	1024	1027	1111
K18955 WhiB family transcriptional regulator, redox-sensing transcriptional regulator	0	0	5	5	0	2	1	0	8	4	3	5
K18991 AraC family transcriptional regulator, activator of mtrCDE	2147	2092	2058	2063	1157	1247	1101	1235	1226	1255	1218	1254
K19057 MerR family transcriptional regulator, mercuric resistance operon regulatory protein	735	698	748	757	518	535	545	497	202	156	162	170
K19242 LysR family transcriptional regulator, repressor for citA	36	32	33	35	5	4	2	3	66	57	76	70
K19333 IclR family transcriptional regulator, KDG regulon repressor	604	566	528	578	405	318	276	321	728	710	693	743
K19335 TetR/AcrR family transcriptional regulator, repressor for divergent bdcA	171	199	211	215	57	49	40	52	349	389	394	439
K19337 RpiR family transcriptional regulator, carbohydrate utilization regulator	2487	2482	2446	2409	1462	1504	1405	1444	1361	1258	1208	1338
K19338 LysR family transcriptional regulator, nitrogen assimilation regulatory protein	598	612	559	589	397	428	397	355	786	839	752	865
K19339 NosR/NirI family transcriptional regulator, nitrous oxide reductase regulator	17	11	20	24	11	23	6	7	10	14	16	17
K19449 XRE family transcriptional regulator, master regulator for biofilm formation	3	0	6	3	0	0	2	0	11	12	15	13
K19505 sigma-54 dependent transcriptional regulator, gfr operon transcriptional activator	1226	1318	1189	1235	348	389	363	329	2257	2398	2388	2390
K19575 MerR family transcriptional regulator, activator of bmr gene	4	3	11	8	0	0	1	1	3	4	3	1
K19587 Rrf2 family transcriptional regulator, repressor of oqxAB	309	301	262	321	200	222	181	181	418	374	373	423
K19591 MerR family transcriptional regulator, copper efflux regulator	883	924	823	891	564	507	515	551	558	589	553	522
K19592 MerR family transcriptional regulator, gold-responsive activator of gol and ges genes	0	0	0	0	0	0	3	2	0	2	3	1
K19736 TetR/AcrR family transcriptional regulator, regulator of autoinduction and epiphytic fitness	0	0	0	0	0	1	0	0	0	0	1	0
K19775 GntR family transcriptional regulator, hexuronate regulon transcriptional repressor	389	421	397	395	343	376	345	325	509	514	541	629
K19776 GntR family transcriptional regulator, galactonate operon transcriptional repressor	459	399	391	417	335	351	332	280	648	680	597	637
K21405 sigma-54 dependent transcriptional regulator, acetoin dehydrogenase operon transcriptional activator AcoR	5752	5607	5087	5380	2951	2989	2833	3226	3039	3090	2870	2998
K21555 CRP/FNR family transcriptional regulator, carbon monoxide oxidation system transcription regulator	3	2	5	7	3	4	4	2	4	24	7	6
K21556 CRP/FNR family transcriptional regulator, polysaccharide utilization system transcription regulator	400	442	459	449	1511	1418	1504	1403	1017	1073	1108	987
K21557 HTH-type transcriptional regulator, polysaccharide utilization system transcription regulator	963	1144	1196	1033	3593	3511	3602	3562	2633	2385	2519	2414
K21562 CRP/FNR family transcriptional regulator, anaerobic regulatory protein	4	2	8	11	20	17	10	17	25	37	31	30
K21563 CRP/FNR family transcriptional regulator, dissimilatory nitrate respiration regulator	463	437	409	390	245	227	210	254	143	108	102	120
K21564 CRP/FNR family transcriptional regulator, nitrogen oxide reductase regulator	2	2	2	0	0	0	0	0	0	5	0	2
K21600 CsoR family transcriptional regulator, copper-sensing transcriptional repressor	163	189	152	167	95	93	84	109	219	270	240	254
K21602 IclR family transcriptional regulator, carbohydrate utilization repressor	216	196	232	205	302	317	291	310	129	114	120	133
K21645 LysR family transcriptional regulator, hypochlorite-specific transcription factor HypT	569	574	557	560	240	268	252	260	761	834	717	817
K21686 XRE family transcriptional regulator, fatty acid utilization regulator	0	0	0	0	2	2	0	0	4	7	2	4
K21697 LuxR family transcriptional regulator, quorum-sensing system regulator VjbR	2	0	0	3	0	0	0	0	1	4	3	0
K21698 LysR family transcriptional regulator, transcriptional activator for aaeXAB operon	654	569	597	550	405	443	465	430	779	767	789	831
K21699 LysR family transcriptional regulator, transcriptional activator for bauABCD operon	1574	1559	1439	1481	865	879	793	902	497	495	436	447
K21701 AraC family transcriptional regulator, transcriptional activator for feuABC-ybbA operon	36	35	42	35	8	5	8	10	39	61	33	47
K21703 LysR family transcriptional regulator, low CO2-responsive transcriptional regulator	1	1	2	1	0	0	0	0	1	0	2	2
K21711 LysR family transcriptional regulator, putative pyruvate carboxylase regulator	1531	1623	1536	1638	926	874	869	982	551	468	536	550
K21742 LysR family transcriptional regulator, transcriptional activator for ttdABT operon	1	5	5	3	14	12	29	5	51	62	47	58
K21743 MerR family transcriptional regulator, multidrug-efflux activator	2	2	2	1	0	0	0	0	4	3	2	1
K21744 MerR family transcriptional regulator, thiopeptide resistance regulator	8	5	3	2	1	0	1	1	7	3	9	5
K21745 MerR family transcriptional regulator, aldehyde-responsive regulator	2	1	6	2	0	0	0	0	3	2	8	6
K21746 AraC family transcriptional regulator, reactive chlorine species (RCS)-specific activator of rcl operon	104	94	126	105	41	29	55	40	125	163	143	184
K21755 LysR family transcriptional regulator, salicylic acid-responsive activator of bsdBCD	171	185	186	191	289	299	284	298	5	6	16	10
K21757 LysR family transcriptional regulator, benzoate and cis,cis-muconate-responsive activator of ben and cat genes	1013	1029	897	1018	549	441	522	602	346	335	277	321
K21825 AraC family transcriptional regulator, L-arginine-responsive activator	1454	1494	1451	1452	851	778	675	859	486	456	427	410
K21826 AraC family transcriptional regulator, glycine betaine-responsive activator	1634	1675	1498	1590	872	917	769	943	550	462	492	512
K21828 CRP/FNR family transcriptional regulator, arginine deiminase pathway regulator	39	45	37	49	21	15	8	17	43	49	49	49
K21829 D-arginine utilization repressor	877	929	956	927	529	461	447	536	314	294	272	282
K21885 ArsR family transcriptional regulator, cadmium/lead-responsive transcriptional repressor	0	0	0	0	0	0	0	0	1	1	1	0
K21900 LysR family transcriptional regulator, transcriptional activator of the cysJI operon	31	23	42	31	2	4	2	9	47	60	35	54
K21901 LuxR family transcriptional regulator, dicarboxylate transport regulator	4	5	2	5	4	5	2	4	31	51	39	32
K21903 ArsR family transcriptional regulator, lead/cadmium/zinc/bismuth-responsive transcriptional repressor	197	185	187	187	100	103	119	123	220	210	177	187
K21905 AraC family transcriptional regulator, glutamate-dependent acid resistance regulator	3	1	4	1	10	15	5	4	47	41	31	30
K21906 AraC family transcriptional regulator, glutamate-dependent acid resistance regulator	0	0	2	1	11	9	5	10	64	68	42	57
K21907 LuxR family transcriptional regulator, glutamate-dependent acid resistance regulator	1	2	2	0	9	1	11	5	23	28	40	34
K21959 LysR family transcriptional regulator, cell division regulator	1	2	2	2	0	0	1	0	0	0	0	0
K21960 LysR family transcriptional regulator, regulator of the ytmI operon	0	2	3	3	0	0	1	0	0	2	4	0
K21961 TetR/AcrR family transcriptional regulator, ethionamide resistance regulator	107	117	101	102	0	4	0	0	59	62	77	74
K21963 LuxR family transcriptional regulator, Mat/Ecp fimbriae transcriptional regulator	106	114	104	104	37	32	35	51	243	224	210	266
K21972 MerR family transcriptional regulator, repressor of blue light- and temperature-responsive genes	436	369	387	442	348	342	328	282	572	583	559	567
K22041 TetR/AcrR family transcriptional regulator, copper-responsive repressor	338	370	358	351	228	257	291	240	521	626	596	621
K22043 ArsR family transcriptional regulator, zinc-responsive transcriptional repressor	21	18	8	17	10	9	8	8	21	25	34	27
K22103 DeoR family transcriptional regulator, carbon catabolite repression regulator	49	40	44	45	3	15	15	7	47	40	50	36
K22104 GntR family transcriptional regulator, sialic acid-inducible nan operon repressor	225	178	185	209	261	261	250	267	67	64	75	74
K22105 TetR/AcrR family transcriptional regulator, fatty acid biosynthesis regulator	440	451	406	447	309	371	337	313	596	592	591	581
K22106 TetR/AcrR family transcriptional regulator, repressor of fatR-cypB operon	11	23	12	7	8	21	10	4	7	29	26	34
K22108 TetR/AcrR family transcriptional regulator, cholesterol catabolism regulator	7	2	2	4	2	7	3	3	17	17	18	13
K22293 GntR family transcriptional regulator, rspAB operon transcriptional repressor	491	540	528	516	277	276	273	245	594	617	627	577
K22296 MarR family transcriptional regulator, lower aerobic nicotinate degradation pathway regulator	639	660	636	621	310	295	286	317	176	158	202	175
K22299 HTH-type transcriptional regulator, competence development regulator	1	1	0	1	0	0	0	0	1	1	0	4
K22300 HTH-type transcriptional regulator, cell division transcriptional repressor	73	51	38	87	93	110	103	108	2	4	1	0
K22302 transcriptional repressor of cell division inhibition gene dicB	54	61	59	58	73	76	87	73	6	13	10	12
K22486 transcriptional regulator HilA, main transcriptional regulator of SPI1	0	0	0	0	14	7	24	16	7	2	5	4
K22489 MarR family transcriptional regulator, temperature-dependent positive regulator of motility	264	266	242	243	146	195	174	184	261	356	309	325
K22491 MerR family transcriptional regulator, light-induced transcriptional regulator	1562	1494	1423	1382	828	789	719	868	451	430	423	420
K22650 luxR family transcriptional regulator, regulator of transport and utilization of aryl beta-glucosides	376	417	387	427	309	289	331	288	539	556	538	615
K23015 GntR family transcriptional regulator, transcriptional activator for L-galactonate catabolism	226	253	202	216	277	304	301	273	47	49	64	57
K23237 AraC family transcriptional regulator, melibiose operon regulatory protein	383	424	318	418	115	129	111	99	845	923	815	913
K23238 RpiR family transcriptional regulator, repressor of rpiB and als operon	6	2	6	8	6	3	3	3	50	70	58	68
K23239 FrmR/RcnR family transcriptional regulator, repressor of frmRAB operon	188	157	157	177	89	131	94	114	211	221	186	200
K23240 FrmR/RcnR family transcriptional regulator, repressor of rcnA expression	152	188	193	182	143	105	129	96	264	236	278	254
K23253 PucR family transcriptional regulator, proline-responsive transcriptional activator	0	1	0	0	0	0	0	0	4	1	0	3
K23356 HTH-type transcriptional regulator, sugar sensing transcriptional regulator	100	85	99	96	14	14	5	1	134	134	177	149
ko03021 Transcription machinery	91360	91530	92565	91532	99939	100209	100092	99903	93164	93260	93398	91380
K02600 transcription termination/antitermination protein NusA	3852	3876	4055	3982	4996	5027	5018	4994	4688	4834	4430	4619
K02601 transcription termination/antitermination protein NusG	1699	1740	1750	1844	2017	1937	2063	1895	1895	2097	2048	1954
K03057 transcription factor S	0	0	0	0	2	0	0	1	3	1	3	4
K03086 RNA polymerase primary sigma factor	4796	4817	4923	4757	4180	4189	3963	3918	5070	5076	5251	5143
K03088 RNA polymerase sigma-70 factor, ECF subfamily	10471	10767	11039	10236	19908	19856	20345	20347	14507	13939	14524	13727
K03089 RNA polymerase sigma-32 factor	2116	1990	2008	1957	1295	1174	1166	1295	1234	1216	1223	1243
K03090 RNA polymerase sigma-B factor	9	11	19	17	10	18	23	9	22	25	13	26
K03091 RNA polymerase sporulation-specific sigma factor	1368	1381	1413	1465	440	513	549	463	1341	1642	1383	1469
K03093 RNA polymerase sigma factor	15	21	16	20	6	2	3	2	15	16	13	14
K03580 ATP-dependent helicase HepA [EC:3.6.4.-]	5931	5578	5652	5672	3367	3260	3305	3401	3801	3939	3582	3725
K03597 sigma-E factor negative regulatory protein RseA	1367	1357	1251	1286	825	849	753	801	862	885	745	818
K03598 sigma-E factor negative regulatory protein RseB	1815	1772	1773	1862	1129	1112	1076	1139	1118	1136	1112	1136
K03599 stringent starvation protein A	627	650	684	619	433	480	485	490	623	644	605	619
K03600 stringent starvation protein B	909	870	854	929	597	570	540	480	584	631	612	636
K03624 transcription elongation factor GreA	1361	1416	1469	1489	1704	1734	1819	1771	1903	2010	1963	1927
K03625 transcription antitermination protein NusB	1827	1955	1773	1809	2594	2555	2623	2622	2472	2152	2232	2204
K03803 sigma-E factor negative regulatory protein RseC	1193	1252	1206	1194	1374	1356	1318	1361	1118	1300	1176	1128
K04749 anti-sigma B factor antagonist	28	14	23	19	7	6	7	6	7	15	10	17
K04760 transcription elongation factor GreB	1196	1112	1079	1057	655	678	655	604	739	715	687	803
K07315 phosphoserine phosphatase RsbU/P [EC:3.1.3.3]	314	301	294	310	104	74	73	93	253	245	212	273
K11367 chromodomain-helicase-DNA-binding protein 1 [EC:3.6.4.12]	0	0	0	2	0	0	0	0	0	0	0	0
K14845 RAT1-interacting protein	0	0	0	0	1	0	0	0	0	0	0	0
K19000 Rho-binding antiterminator	691	706	688	728	438	415	413	427	402	383	399	387
ko03019 Messenger RNA biogenesis	85737	86214	86968	86768	87635	89767	88744	87845	86439	87295	86668	86568
K02553 regulator of ribonuclease activity A	1094	1047	1058	1070	610	699	597	656	649	682	650	746
K03698 3'-5' exoribonuclease [EC:3.1.-.-]	699	771	717	749	197	218	203	201	705	783	718	778
K06958 RNase adapter protein RapZ	2133	2021	1891	2018	1151	1189	1254	1178	1477	1667	1552	1633
K08301 ribonuclease G [EC:3.1.26.-]	4036	4064	4091	4058	4447	4957	4809	4779	4419	4149	4240	4181
K09893 regulator of ribonuclease activity B	269	254	236	306	165	213	190	161	413	422	368	356
K10357 myosin V	0	0	0	0	0	0	1	0	0	2	0	0
K11493 regulator of chromosome condensation	1	0	0	0	0	0	0	0	0	0	0	0
K18692 ATP-dependent RNA helicase CshB [EC:3.6.4.13]	63	58	70	60	20	32	14	17	80	103	77	63
K18765 RNase E specificity factor CsrD	1201	1178	1107	1260	779	908	878	865	1598	1676	1591	1669
ko03041 Spliceosome	19	17	19	12	0	2	2	0	30	31	32	43
K12736 peptidylprolyl isomerase domain and WD repeat-containing protein 1 [EC:5.2.1.8]	0	0	0	0	0	0	0	0	0	0	2	0
K13177 ATP-dependent RNA helicase DDX1 [EC:3.6.4.13]	0	0	0	0	0	0	0	0	1	0	0	0
K13179 ATP-dependent RNA helicase DDX18/HAS1 [EC:3.6.4.13]	1	0	0	0	0	0	0	0	0	0	0	0
K17043 ATP-dependent RNA helicase DDX43 [EC:3.6.4.13]	0	0	0	0	0	0	0	0	0	1	0	0

ko03011 Ribosome	78384	79588	82698	81135	88612	88928	90151	86988	84998	85396	85577	85767
K17437 large subunit ribosomal protein L15	0	2	0	0	0	0	0	0	0	0	0	0
ko03009 Ribosome biogenesis	156988	157286	156854	157650	151246	152400	152627	152332	156586	157365	155923	156824
K00561 23S rRNA (adenine-N6)-dimethyltransferase [EC:2.1.1.184]	387	525	544	531	1488	1520	1652	1535	1109	1014	1086	1057
K00563 23S rRNA (guanine745-N1)-methyltransferase [EC:2.1.1.187]	1715	1771	1581	1725	1103	1081	962	1076	1131	1173	1109	1027
K00564 16S rRNA (guanine1207-N2)-methyltransferase [EC:2.1.1.172]	2307	2357	2243	2350	1472	1287	1285	1411	1444	1447	1446	1554
K00783 23S rRNA (pseudouridine1915-N3)-methyltransferase [EC:2.1.1.177]	1706	1733	1758	1731	1843	1790	1747	1937	1801	1795	1750	1732
K02427 23S rRNA (uridine2552-2'-O)-methyltransferase [EC:2.1.1.166]	1198	1119	1212	1206	791	820	765	800	883	905	838	904
K02528 16S rRNA (adenine1518-N6/adenine1519-N6)-dimethyltransferase [EC:2.1.1.182]	2840	2744	2843	2791	2884	2837	2986	2976	2859	2919	2871	2888
K02687 ribosomal protein L11 methyltransferase [EC:2.1.1.-]	3044	3116	3033	2853	2949	3191	3011	2864	2822	3033	2822	2906
K02834 ribosome-binding factor A	1415	1372	1395	1364	1406	1428	1422	1405	1434	1519	1452	1430
K02860 16S rRNA processing protein RimM	1884	1769	1911	1727	1912	1957	2016	1936	1810	1803	1780	1895
K02972 stationary-phase-induced ribosome-associated protein	83	100	72	99	54	60	75	64	114	115	141	136
K03212 23S rRNA (uracil747-C5)-methyltransferase [EC:2.1.1.189]	667	660	627	634	388	396	420	410	898	914	864	861
K03214 RNA methyltransferase, TrmH family [EC:2.1.1.-]	603	602	632	619	444	438	490	467	852	921	819	912
K03215 23S rRNA (uracil1939-C5)-methyltransferase [EC:2.1.1.190]	3738	3895	3815	3824	2919	2987	2903	2860	3392	3412	3256	3518
K03218 23S rRNA (guanosine2251-2'-O)-methyltransferase [EC:2.1.1.185]	2222	2177	2222	2212	2608	2628	2459	2465	2464	2569	2542	2530
K03438 16S rRNA (cytosine1402-N4)-methyltransferase [EC:2.1.1.199]	3676	3332	3688	3503	3563	3629	3523	3542	3202	3291	3425	3279
K03500 16S rRNA (cytosine967-C5)-methyltransferase [EC:2.1.1.176]	3831	3714	3490	3820	2201	2162	2065	2225	2557	2671	2748	2654
K03501 16S rRNA (guanine527-N7)-methyltransferase [EC:2.1.1.170]	2268	2318	2287	2254	2086	2248	2229	2172	1902	1924	2016	1886
K03595 GTPase	2743	2781	2980	2758	3136	3332	3297	3167	3167	3242	3250	3196
K03665 GTPase	4234	4324	4318	4224	4706	4776	4748	4865	4318	4320	4511	4365
K03789 [ribosomal protein S18]-alanine N-acetyltransferase [EC:2.3.1.266]	1433	1342	1252	1394	701	706	750	798	933	1000	977	925
K03790 [ribosomal protein S5]-alanine N-acetyltransferase [EC:2.3.1.267]	1739	1768	1748	1725	919	863	894	971	1371	1368	1392	1449
K03812 ribosome modulation factor	317	334	336	327	198	210	196	201	197	191	210	219
K03817 ribosomal-protein-serine acetyltransferase [EC:2.3.1.-]	891	884	826	816	445	484	465	486	617	578	563	603
K03977 GTPase	3157	3311	3353	3288	4117	4545	4325	4196	4472	4614	4647	4528
K03979 GTPase [EC:3.6.5.-]	3851	3708	3807	3814	4099	3948	4206	4146	3938	4054	3870	3836
K04795 fibrillarin-like pre-rRNA processing protein	0	0	0	0	0	0	0	2	0	2	2	4
K05590 ATP-dependent RNA helicase SrmB [EC:3.6.4.13]	697	702	673	744	555	507	453	526	1047	1027	952	1046
K05591 ATP-independent RNA helicase DbpA [EC:3.6.4.13]	1614	1530	1575	1627	1080	1110	1032	1125	1311	1362	1322	1465
K05808 putative sigma-54 modulation protein	1176	1125	1165	1177	1177	1091	1125	1208	1105	1153	1164	1192
K05809 ribosome-associated inhibitor A	241	213	201	194	155	196	167	144	315	337	333	358
K05844 ribosomal protein S6--L-glutamate ligase [EC:6.3.2.-]	1068	1145	1117	1151	782	760	788	736	967	943	954	895
K06174 ATP-binding cassette, sub-family E, member 1	3	0	0	1	0	0	2	2	4	16	20	7
K06177 tRNA pseudouridine32 synthase / 23S rRNA pseudouridine746 synthase [EC:5.4.99.28 5.4.99.29]	2909	2844	2782	2807	3718	3804	3797	3649	3400	3258	3295	3219
K06178 23S rRNA pseudouridine2605 synthase [EC:5.4.99.22]	776	825	836	837	672	727	914	986	1026	1058	966	1054
K06179 23S rRNA pseudouridine955/2504/2580 synthase [EC:5.4.99.24]	2298	2421	2435	2420	1432	1486	1360	1382	1738	1676	1669	1669
K06180 23S rRNA pseudouridine1911/1915/1917 synthase [EC:5.4.99.23]	4835	4864	5154	5023	7249	7418	7297	7164	6469	6289	6697	6448
K06181 23S rRNA pseudouridine2457 synthase [EC:5.4.99.20]	1468	1385	1289	1334	812	761	657	754	881	847	828	882
K06182 23S rRNA pseudouridine2604 synthase [EC:5.4.99.21]	1838	1787	1745	1838	939	997	924	1067	1365	1263	1300	1261
K06183 16S rRNA pseudouridine516 synthase [EC:5.4.99.19]	1702	1784	1681	1669	1029	986	1031	1011	1247	1161	1208	1283
K06442 23S rRNA (cytidine1920-2'-O)/16S rRNA (cytidine1409-2'-O)-methyltransferase [EC:2.1.1.226 2.1.1.227]	506	462	464	530	172	176	156	171	521	568	491	537
K06941 23S rRNA (adenine2503-C2)-methyltransferase [EC:2.1.1.192]	3673	3546	3857	3715	3873	3928	3996	4010	4139	4110	4153	4117
K06942 ribosome-binding ATPase	3863	4025	4076	4048	4638	4857	4673	4580	4149	4293	4458	4260
K06948 30S ribosome assembly GTPase	73	69	65	63	14	19	33	27	70	97	64	77
K06961 ribosomal RNA assembly protein	0	0	0	1	2	0	0	0	2	3	3	10
K06968 23S rRNA (cytidine2498-2'-O)-methyltransferase [EC:2.1.1.186]	1380	1378	1330	1370	782	839	804	843	1156	1178	1200	1160
K06969 23S rRNA (cytosine1962-C5)-methyltransferase [EC:2.1.1.191]	5367	5358	5434	5248	4752	4722	5108	4930	4377	4525	4490	4397
K06970 23S rRNA (adenine1618-N6)-methyltransferase [EC:2.1.1.181]	1050	1094	1058	1061	2195	2005	2242	2102	2139	2097	2165	2200
K07042 probable rRNA maturation factor	1168	1132	1099	1115	689	670	647	694	897	866	814	960
K07056 16S rRNA (cytidine1402-2'-O)-methyltransferase [EC:2.1.1.198]	2360	2439	2542	2491	3846	3812	3961	3827	3485	3378	3588	3534
K07115 23S rRNA (adenine2030-N6)-methyltransferase [EC:2.1.1.266]	2488	2585	2502	2484	3029	3112	3006	3140	2672	2410	2452	2559
K07320 ribosomal protein L3 glutamine methyltransferase [EC:2.1.1.298]	950	934	854	1025	637	610	614	582	944	1003	989	965
K07566 L-threonylcarbamoyladenylate synthase [EC:2.7.7.87]	2186	2058	2045	2078	2294	2129	2321	2396	2386	2436	2375	2350
K07569 RNA-binding protein	2	2	0	1	0	0	0	0	1	5	0	1
K07574 RNA-binding protein	909	947	924	944	594	547	522	484	636	617	568	612
K08316 16S rRNA (guanine966-N2)-methyltransferase [EC:2.1.1.171]	1305	1275	1174	1206	781	726	734	779	855	865	821	898
K09136 ribosomal protein S12 methylthiotransferase accessory factor	1723	1553	1572	1660	1002	1086	1117	946	1650	1751	1604	1749
K09140 pre-rRNA-processing protein TSR3	0	0	0	0	0	0	0	0	0	3	0	6
K09710 ribosome-associated protein	972	986	1121	1041	1256	1132	1270	1204	1169	1183	1182	1151
K09748 ribosome maturation factor RimP	1563	1659	1778	1699	1892	1865	1858	1850	1638	1709	1759	1612
K09761 16S rRNA (uracil1498-N3)-methyltransferase [EC:2.1.1.193]	2612	2711	2533	2473	2778	2717	2697	2762	2569	2778	2569	2638
K09889 ribosome-associated protein	1107	1103	1070	1132	662	656	681	701	807	719	684	763
K11145 ribonuclease III family protein [EC:3.1.26.-]	192	218	210	209	71	48	71	53	215	214	188	238
K11391 23S rRNA (guanine1835-N2)-methyltransferase [EC:2.1.1.174]	1882	1977	1885	1922	1237	1187	1182	1239	1438	1342	1361	1447
K11392 16S rRNA (cytosine1407-C5)-methyltransferase [EC:2.1.1.178]	771	771	762	835	515	538	575	556	989	1089	1088	1174
K12297 23S rRNA (guanine2445-N2)-methyltransferase / 23S rRNA (guanine2069-N7)-methyltransferase [EC:2.1.1.173 2.1.1.264]	3654	3731	3517	3678	2190	2156	2110	2054	2569	2571	2568	2485
K14441 ribosomal protein S12 methylthiotransferase [EC:2.8.4.4]	3914	3764	3882	3909	4323	4251	4270	4288	3926	3895	3793	3982
K14540 ribosome biogenesis GTPase A	439	432	442	416	160	155	180	157	496	476	435	490
K14761 ribosome-associated protein	264	280	292	269	160	156	172	156	357	334	368	392
K14762 ribosome-associated protein	191	199	187	186	180	165	152	149	263	243	272	244
K14768 U3 small nucleolar RNA-associated protein 7	0	0	0	0	0	0	0	0	0	1	0	0
K14779 ATP-dependent RNA helicase DDX52/ROK1 [EC:3.6.4.13]	0	4	0	0	0	0	0	0	0	0	0	0
K14782 protein AATF/BFR2	0	0	0	1	0	0	0	0	0	0	0	0
K14797 essential nuclear protein 1	0	0	0	0	0	0	0	0	0	0	0	1
K14810 ATP-dependent RNA helicase DDX56/DBP9 [EC:3.6.4.13]	0	0	0	0	0	0	0	0	0	0	1	0
K14811 ATP-dependent RNA helicase DBP3 [EC:3.6.4.13]	0	0	2	0	0	0	0	0	0	0	0	0
K14831 protein MAK16	0	0	0	0	0	0	0	0	0	2	0	0
K14832 ribosome biogenesis protein MAK21	0	0	0	0	0	0	0	0	0	0	2	0
K14846 ribosome production factor 1	0	2	0	0	0	0	0	0	0	0	0	0
K14859 ribosome biogenesis protein SSF1/2	0	0	0	0	0	0	0	0	0	0	0	1
K15508 21S rRNA (uridine2791-2'-O)-methyltransferase [EC:2.1.1.168]	0	0	0	0	0	0	0	0	0	2	0	0
K15632 23S rRNA (adenine-C8)-methyltransferase [EC:2.1.1.224]	6	5	6	4	1	1	1	0	24	24	12	17
K15984 16S rRNA (guanine1516-N2)-methyltransferase [EC:2.1.1.242]	1978	1901	1731	1854	1159	1043	1029	1072	1251	1240	1198	1181
K18850 50S ribosomal protein L16 3-hydroxylase [EC:1.14.11.47]	2001	2026	2005	2115	1361	1375	1365	1313	2059	2221	1922	2059
K20224 importin-9	0	2	0	0	0	0	0	0	0	0	0	0
K21514 23S rRNA (uridine2479-2'-O)-methyltransferase [EC:2.1.1.208]	3	0	2	3	0	0	0	0	3	2	0	1
ko03016 Transfer RNA biogenesis	302875	303504	304679	304679	300276	304600	302449	299449	299014	301908	298864	300575
K00554 tRNA (guanine37-N1)-methyltransferase [EC:2.1.1.228]	2309	2356	2488	2380	2298	2431	2449	2318	2469	2459	2568	2435
K00555 tRNA (guanine26-N2/guanine27-N2)-dimethyltransferase [EC:2.1.1.215 2.1.1.216]	0	0	3	1	0	0	0	2	1	10	6	12
K00556 tRNA (guanosine-2'-O-)-methyltransferase [EC:2.1.1.34]	501	449	479	489	280	389	320	331	622	713	666	732
K00557 tRNA (uracil-5-)-methyltransferase [EC:2.1.1.35]	2379	2429	2258	2376	1396	1418	1365	1391	1479	1487	1401	1410
K00773 queuine tRNA-ribosyltransferase [EC:2.4.2.29]	3471	3449	3732	3538	3572	3588	3801	3557	3601	3978	3763	3837
K00989 ribonuclease PH [EC:2.7.7.56]	1901	1809	1898	1739	1220	1198	1143	1142	1229	1153	1142	1249
K01147 exoribonuclease II [EC:3.1.13.1]	1254	1236	1101	1171	947	1022	943	947	1731	1792	1685	1739
K01166 ribonuclease T2 [EC:4.6.1.19]	1067	1102	1176	997	638	593	582	571	354	350	328	354
K01169 ribonuclease I (enterobacter ribonuclease) [EC:4.6.1.21]	471	446	490	435	333	335	331	284	604	637	624	689
K01170 tRNA-intron endonuclease, archaea type [EC:4.6.1.16]	1	0	0	0	0	2	0	2	0	0	3	1
K01409 N6-L-threonylcarbamoyladenine synthase [EC:2.3.1.234]	3919	3728	3748	3765	3464	3511	3381	3491	3469	3477	3510	3602
K01855 tRNA pseudouridine38/39 synthase [EC:5.4.99.45]	0	0	0	0	1	0	0	0	0	0	0	0
K01975 RNA 2',3'-cyclic 3'-phosphodiesterase [EC:3.1.4.58]	1238	1253	1214	1248	683	746	618	735	827	746	735	727
K02533 tRNA/rRNA methyltransferase [EC:2.1.1.-]	412	523	544	475	358	418	394	338	744	682	717	750
K03177 tRNA pseudouridine55 synthase [EC:5.4.99.25]	3185	3226	3247	3218	3285	3292	3270	3183	3147	3086	3099	2940
K03216 tRNA (cytidine/uridine-2'-O-)-methyltransferase [EC:2.1.1.207]	1502	1471	1452	1472	794	819	819	739	1133	1074	1011	1135
K03437 RNA methyltransferase, TrmH family	895	875	993	828	1444	1530	1492	1379	1365	1390	1497	1401
K03439 tRNA (guanine-N7-)-methyltransferase [EC:2.1.1.33]	2645	2568	2538	2650	2927	3203	3055	3025	2691	2661	2640	2658
K03495 tRNA uridine 5-carboxymethylaminomethyl modification enzyme	4585	4452	4727	4489	6370	6530	6640	6370	6461	6698	6582	6403
K03536 ribonuclease P protein component [EC:3.1.26.5]	1390	1400	1413	1404	1515	1574	1557	1644	1507	1587	1556	1538
K03650 tRNA modification GTPase [EC:3.6.-.-]	5245	5145	5334	5165	5227	5432	5572	5267	5242	5197	5115	5092
K03683 ribonuclease T [EC:3.1.13.-]	1206	1177	1123	1232	658	680	608	601	821	783	775	743
K03684 ribonuclease D [EC:3.1.13.5]	2461	2455	2464	2476	1428	1437	1363	1400	1420	1478	1459	1494
K03976 Cys-tRNA(Pro)/Cys-tRNA(Cys) deacylase [EC:3.1.1.-]	1484	1554	1501	1628	1610	1637	1604	1655	1513	1592	1545	1508
K04075 tRNA(Ile)-lysidine synthase [EC:6.3.4.19]	4295	4540	4403	4425	4550	4591	4358	4527	4201	4345	4268	4313
K04094 methylenetetrahydrofolate--tRNA-(uracil-5-)-methyltransferase [EC:2.1.1.74]	171	138	129	128	100	79	109	118	142	180	194	180
K05539 tRNA-dihydrouridine synthase A [EC:1.-.-.-]	2108	2041	2088	2054	1246	1393	1225	1335	1432	1492	1309	1420
K05540 tRNA-dihydrouridine synthase B [EC:1.-.-.-]	2158	2087	2076	2209	1304	1279	1299	1325	1369	1382	1391	1369
K05541 tRNA-dihydrouridine synthase C [EC:1.-.-.-]	1338	1233	1209	1311	737	821	704	776	1028	996	964	1127
K06020 energy-dependent translational throttle protein EttA	3895	3784	3420	3593	2238	2243	2264	2346	2187	2318	2202	2414
K06168 tRNA-2-methylthio-N6-dimethylallyladenosine synthase [EC:2.8.4.3]	4371	4415	4476	4488	5198	5262	5421	5208	5044	5183	5037	5027
K06169 tRNA-(ms[2]io[6]A)-hydroxylase [EC:1.-.-.-]	1316	1370	1276	1277	778	750	804	782	935	935	872	979

K06173 tRNA pseudouridine38-40 synthase [EC:5.4.99.12]	2952	2849	2992	2847	2860	2760	2775	2878	2869	3071	3129	3044
K06175 tRNA pseudouridine65 synthase [EC:5.4.99.26]	447	487	442	504	326	358	365	359	632	709	684	691
K06176 tRNA pseudouridine13 synthase [EC:5.4.99.27]	2191	2075	1950	2030	1262	1215	1247	1257	1285	1333	1310	1371
K06917 tRNA 2-selenouridine synthase [EC:2.9.1.-]	966	1044	864	987	441	453	453	390	1082	1272	1228	1173
K06925 tRNA threonylcarbamoyladenosine biosynthesis protein TsaE	1487	1554	1632	1545	1645	1713	1693	1641	1565	1583	1611	1686
K06932 tRNA(Ile2)-agmatinylcytidine synthase [EC:6.3.4.22]	0	0	1	0	0	3	0	0	4	3	4	10
K06957 tRNA(Met) cytidine acetyltransferase [EC:2.3.1.193]	1210	1216	1130	1247	780	881	846	852	1600	1572	1515	1665
K06967 tRNA (adenine22-N1)-methyltransferase [EC:2.1.1.217]	319	306	308	294	74	84	103	99	331	370	326	345
K06980 tRNA-modifying protein YgfZ	2214	2098	1908	2032	1305	1267	1147	1339	1371	1365	1241	1467
K07050 misacylated tRNA(Ala) deacylase [EC:3.1.1.-]	0	0	0	0	0	0	2	1	0	0	0	1
K07055 tRNA wybutosine-synthesizing protein 2 [EC:2.5.1.114]	0	0	0	1	0	2	3	0	4	0	2	2
K07254 tRNA (cytidine56-2'-O)-methyltransferase [EC:2.1.1.206]	0	0	0	1	0	0	0	1	4	5	0	3
K07442 tRNA (adenine57-N1/adenine58-N1)-methyltransferase catalytic subunit [EC:2.1.1.219 2.1.1.220]	12	7	11	10	7	7	8	2	13	37	14	32
K07446 tRNA (guanine10-N2)-dimethyltransferase [EC:2.1.1.213]	0	0	0	1	0	2	0	0	0	5	2	1
K07558 tRNA nucleotidyltransferase (CCA-adding enzyme) [EC:2.7.7.72]	0	0	0	1	0	0	0	4	2	8	3	12
K07559 putative RNA 2'-phosphotransferase [EC:2.7.1.-]	1318	1255	1185	1190	770	710	686	773	477	493	435	415
K07560 D-aminoacyl-tRNA deacylase [EC:3.1.1.96]	1624	1610	1722	1659	1842	1928	1805	1900	1831	1754	1727	1835
K07568 S-adenosylmethionine:tRNA ribosyltransferase-isomerase [EC:2.4.99.17]	4985	5151	5224	5086	6857	6924	6796	6793	6108	6029	6146	6029
K07583 tRNA pseudouridine synthase 10 [EC:5.4.99.25]	0	3	2	1	0	4	0	2	2	13	3	17
K07739 elongator complex protein 3 [EC:2.3.1.48]	1	2	3	0	1	2	0	5	2	32	9	14
K09119 KEOPS complex subunit Cgi121	1	0	0	0	0	0	0	0	4	4	0	3
K09765 epoxyqueuosine reductase [EC:1.17.99.6]	705	692	706	666	1261	1245	1383	1305	1133	1081	1126	1125
K11991 tRNA(adenine34) deaminase [EC:3.5.4.33]	878	956	1041	898	1274	1308	1193	1280	1482	1511	1445	1498
K13628 iron-sulfur cluster assembly protein	704	718	822	688	434	475	418	452	519	443	441	484
K14058 tRNA 2-thiocytidine biosynthesis protein TtcA	1599	1596	1539	1664	1029	1082	994	1020	1192	1257	1197	1287
K14415 tRNA-splicing ligase RtcB (3'-phosphate/5'-hydroxy nucleic acid ligase) [EC:6.5.1.8]	4402	4455	4513	4269	5924	6137	6341	6268	4766	4684	4784	4593
K14742 tRNA threonylcarbamoyladenosine biosynthesis protein TsaB	2116	2176	2063	2054	2301	2286	2358	2328	2116	2093	2042	2056
K14864 tRNA (cytidine32/guanosine34-2'-O)-methyltransferase [EC:2.1.1.205]	0	2	0	0	0	0	0	0	0	0	0	0
K15256 tRNA (cmo5U34)-methyltransferase [EC:2.1.1.-]	2422	2505	2461	2510	1463	1504	1551	1534	1177	1153	1134	1122
K15257 tRNA (mo5U34)-methyltransferase [EC:2.1.1.-]	2078	1976	1987	1950	1073	1149	1088	1170	1225	1229	1274	1261
K15396 tRNA (cytidine32/uridine32-2'-O)-methyltransferase [EC:2.1.1.200]	1616	1642	1614	1610	937	958	905	896	1019	1041	976	1059
K15429 tRNA (guanine37-N1)-methyltransferase [EC:2.1.1.228]	0	0	0	0	0	0	0	0	0	5	2	6
K15437 aminoacyl tRNA synthase complex-interacting multifunctional protein 1	0	0	0	0	0	0	0	0	0	0	1	0
K15441 tRNA-specific adenosine deaminase 2 [EC:3.5.4.-]	1	0	0	0	0	0	0	0	0	0	0	0
K15449 tRNA wybutosine-synthesizing protein 1 [EC:4.1.3.44]	2	0	2	1	2	0	0	0	5	10	7	3
K15451 tRNA wybutosine-synthesizing protein 4 [EC:2.1.1.290 2.3.1.231]	2	0	0	0	0	0	0	0	0	0	0	0
K15460 tRNA1Val (adenine37-N6)-methyltransferase [EC:2.1.1.223]	805	855	873	895	1688	1712	1779	1483	1629	1481	1464	1581
K15461 tRNA 5-methylaminomethyl-2-thiouridine biosynthesis bifunctional protein [EC:2.1.1.61 1.5.-.-]	3428	3557	3154	3451	1987	1951	1793	2212	2179	2056	2018	2122
K15865 threonylcarbamoyladenosine tRNA methylthiotransferase CDKAL1 [EC:2.8.4.5]	0	0	0	0	0	0	0	0	13	8	3	12
K16317 tRNA (pseudouridine54-N1)-methyltransferase [EC:2.1.1.257]	0	0	0	0	0	0	0	0	0	0	0	1
K18707 threonylcarbamoyladenosine tRNA methylthiotransferase MtaB [EC:2.8.4.5]	1447	1439	1428	1422	2969	2905	3015	2868	2547	2476	2679	2497
K18779 7-cyano-7-deazaguanine tRNA-ribosyltransferase [EC:2.4.2.48]	2	2	0	2	3	0	5	0	9	12	3	13
K18828 tRNA(fMet)-specific endonuclease VapC [EC:3.1.-.-]	591	672	708	659	559	582	625	547	156	152	129	163
K18979 epoxyqueuosine reductase [EC:1.17.99.6]	2505	2511	2408	2521	1329	1379	1310	1447	1552	1693	1556	1643
K19055 Ala-tRNA(Pro) deacylase [EC:3.1.1.-]	1179	1217	1220	1228	717	676	661	639	848	908	821	966
K21947 tRNA-5-methyluridine54 2-sulfurtransferase [EC:2.8.1.15]	16	16	20	11	8	7	6	3	15	27	8	24
K22132 tRNA threonylcarbamoyladenosine dehydratase	2366	2404	2285	2282	2480	2449	2497	2398	2386	2431	2393	2330
K22446 tRNA (cytosine49-C5)-methyltransferase [EC:2.1.1.-]	1	2	0	0	0	0	0	0	0	4	4	1	1
K22900 tRNA (adenine37-N6)-methyltransferase [EC:2.1.1.-]	1267	1126	1073	1183	809	726	722	780	924	922	918	921
K22901 tRNA (cytosine40_48-C5)-methyltransferase [EC:2.1.1.-]	0	0	0	0	0	0	0	0	0	0	1	0
ko03012 Translation factors	74099	73560	74132	74198	79172	80030	80308	79532	75911	75938	75714	75841
K01056 peptidyl-tRNA hydrolase, PTH1 family [EC:3.1.1.29]	2220	2180	2234	2166	2362	2450	2222	2353	2148	2124	2197	2125
K02355 elongation factor G	13885	14034	14571	14373	16119	16274	16431	15978	15295	15373	15119	14999
K02356 elongation factor P	2301	2163	2274	2298	2447	2455	2424	2482	2651	2804	2664	2685
K02357 elongation factor Ts	2096	2094	2153	2163	3033	3178	3099	2950	3111	3183	3199	3084
K02358 elongation factor Tu	7248	7245	7465	7340	6619	7047	6673	6791	6268	6267	6172	6165
K02493 release factor glutamine methyltransferase [EC:2.1.1.297]	2942	2843	2800	2863	2979	3066	3051	3084	2841	2665	2896	2810
K02518 translation initiation factor IF-1	698	680	684	679	767	838	869	800	851	838	812	805
K02519 translation initiation factor IF-2	7933	7841	8057	7814	9232	9372	10195	10116	8034	8185	8140	8210
K02520 translation initiation factor IF-3	1846	1811	1875	1803	1995	1863	2155	2079	1886	1849	1968	1942
K02835 peptide chain release factor 1	3934	3744	3922	4025	4182	4000	4196	3990	4103	4015	3868	3936
K02836 peptide chain release factor 2	3520	3553	3531	3568	3587	3577	3639	3611	3541	3525	3545	3490
K02837 peptide chain release factor 3	4478	4339	4490	4542	5279	5364	5340	5320	5193	5041	5117	5186
K02838 ribosome recycling factor	1991	1899	2039	1971	2089	2159	2101	2094	2013	2032	2016	1978
K02839 peptide chain release factor	1409	1582	1480	1465	1783	1789	1758	1611	1276	1213	1293	1278
K03232 elongation factor 1-beta	0	0	0	0	0	0	0	0	0	0	0	1
K03235 elongation factor 3	0	0	1	0	0	0	0	0	0	1	0	0
K03263 translation initiation factor 5A	0	0	0	0	0	3	0	2	0	0	3	0
K03833 selenocysteine-specific elongation factor	4451	4348	3745	4191	2615	2412	2452	2563	2609	2592	2504	2768
K04568 elongation factor P--(R)-beta-lysine ligase [EC:6.3.1.-]	693	652	621	664	468	463	506	444	885	972	888	900
K04794 peptidyl-tRNA hydrolase, PTH2 family [EC:3.1.1.29]	2	1	0	1	0	5	0	3	0	7	3	3
K06158 ATP-binding cassette, subfamily F, member 3	9144	9213	8978	9047	10940	11018	10571	10559	9939	9971	10093	10068
K06927 diphthine-ammonia ligase [EC:6.3.1.14]	2	3	0	0	0	4	2	0	1	2	3	7
K07561 2-(3-amino-3-carboxypropyl)histidine synthase [EC:2.5.1.108]	2	0	0	0	0	2	0	0	4	3	3	9
K09890 alternative ribosome-rescue factor	339	374	355	366	191	197	188	187	307	277	283	308
K09906 elongation factor P hydroxylase [EC:1.14.-.-]	345	318	333	316	220	213	217	216	426	518	486	490
K15029 translation initiation factor 3 subunit L	0	0	0	0	0	0	0	0	0	0	0	1
K15034 ribosome-associated protein	868	840	836	866	536	483	439	484	506	482	473	473
K19810 L-lysine 2,3-aminomutase [EC:5.4.3.-]	667	653	614	653	434	534	462	449	969	940	903	995
ko03110 Chaperones and folding catalysts	140562	142302	144193	142461	171690	171961	172907	172002	156854	155575	157192	155640
K02199 cytochrome c biogenesis protein CcmG, thiol:disulfide interchange protein DsbE	775	734	716	743	370	356	351	409	583	533	556	562
K03611 disulfide bond formation protein DsbB	2288	2317	2178	2264	1297	1316	1217	1419	1114	1200	1066	1070
K03667 ATP-dependent HslUV protease ATP-binding subunit HslU	3231	3335	3160	3331	1963	2013	1682	1877	2028	2121	1976	2085
K03672 thioredoxin 2 [EC:1.8.1.8]	271	243	248	244	170	188	178	181	354	402	352	412
K03674 glutaredoxin 1	132	163	162	154	100	94	102	105	192	210	193	215
K03675 glutaredoxin 2	77	99	105	132	179	176	214	200	36	31	28	33
K03676 glutaredoxin 3	644	665	631	638	396	462	390	395	400	409	417	428
K03686 molecular chaperone DnaJ	3291	3442	3241	3286	3702	3566	3480	3493	3163	3138	3235	3430
K03687 molecular chaperone GrpE	1148	1061	1093	1097	1375	1433	1686	1548	1564	1559	1566	1466
K03694 ATP-dependent Clp protease ATP-binding subunit ClpA	2891	2798	2889	2884	1959	2008	1995	1932	2427	2450	2275	2439
K03696 ATP-dependent Clp protease ATP-binding subunit ClpC	7391	7401	7383	7143	7670	7260	7681	7673	6183	6162	6199	6048
K03697 ATP-dependent Clp protease ATP-binding subunit ClpE	105	128	122	122	42	40	37	34	128	142	149	157
K03768 peptidyl-prolyl cis-trans isomerase B (cyclophilin B) [EC:5.2.1.8]	1812	1814	1727	1862	913	981	957	1050	1434	1525	1478	1496
K03769 peptidyl-prolyl cis-trans isomerase C [EC:5.2.1.8]	2848	2795	2684	2660	1829	1882	1821	1887	1363	1339	1305	1406
K03770 peptidyl-prolyl cis-trans isomerase D [EC:5.2.1.8]	3607	3514	3713	3647	6022	6216	6396	6170	5246	5137	5271	5307
K03771 peptidyl-prolyl cis-trans isomerase SurA [EC:5.2.1.8]	2606	2845	2940	2787	6809	6968	7175	6967	5754	5251	5598	5336
K03772 FKBP-type peptidyl-prolyl cis-trans isomerase FkpA [EC:5.2.1.8]	570	563	568	561	429	449	446	467	772	666	741	750
K03773 FKBP-type peptidyl-prolyl cis-trans isomerase FklB [EC:5.2.1.8]	4047	4211	4211	4211	4598	4473	4611	4597	3453	3389	3466	3387
K03774 FKBP-type peptidyl-prolyl cis-trans isomerase SlpA [EC:5.2.1.8]	1015	997	1002	958	659	680	583	579	665	605	626	626
K03775 FKBP-type peptidyl-prolyl cis-trans isomerase SlyD [EC:5.2.1.8]	1341	1398	1393	1362	1788	1812	1794	1755	1505	1578	1499	1576
K03805 thiol:disulfide interchange protein DsbG	1360	1373	1257	1336	771	798	768	917	910	840	866	889
K03981 thiol:disulfide interchange protein DsbC [EC:5.3.4.1]	1206	1139	1100	1155	823	893	800	897	897	894	806	864
K04044 molecular chaperone HscA	2858	2831	2744	2776	1949	1810	1913	1930	2169	2209	2164	2199
K04045 molecular chaperone HscC	958	926	943	958	564	685	687	681	623	636	659	714
K04046 hypothetical chaperone protein	2847	2912	2814	2860	1786	1710	1713	1813	1877	1904	1855	2044
K04078 chaperonin GroES	1021	1006	1131	1045	1123	1054	1122	1138	1109	1134	1057	1110
K04080 molecular chaperone IbpA	1158	1147	1054	1226	714	697	725	715	1010	1004	932	1025
K04081 molecular chaperone IbpB	273	298	252	294	246	208	233	194	411	372	373	450
K04082 molecular chaperone HscB	947	885	892	875	545	516	580	558	633	662	597	633
K04083 molecular chaperone Hsp33	2333	2375	2267	2387	1362	1363	1397	1357	1732	1706	1658	1770
K04084 thiol:disulfide interchange protein DsbD [EC:1.8.1.8]	8690	8762	8465	8512	10895	10633	10815	11208	8294	7788	8407	8030
K04086 ATP-dependent Clp protease ATP-binding subunit ClpL	11	22	16	19	38	11	21	16	34	55	27	30
K04651 hydrogenase nickel incorporation protein HypA/HybF	379	389	383	383	268	325	278	260	521	532	512	480
K04652 hydrogenase nickel incorporation protein HypB	723	692	661	726	434	481	444	464	1073	1112	1002	1084
K04762 ribosome-associated heat shock protein Hsp15	1310	1230	1350	1189	1441	1513	1475	1480	1186	1142	1166	1132
K04797 prefoldin alpha subunit	0	0	0	0	2	0	2	3	0	0	1	2
K04798 prefoldin beta subunit	0	0	0	0	0	1	0	0	0	0	0	2
K05516 curved DNA-binding protein	910	966	1015	889	1932	1999	1974	1944	2081	1960	2004	1986
K05801 DnaJ like chaperone protein	1693	1554	1547	1615	1126	1068	931	1094	1067	1045	1042	1108
K05838 putative thioredoxin	2040	1861	1836	1929	1050	1158	1026	1220	1234	1101	1165	1162
K06006 periplasmic protein CpxP/Spy	551	547	480	578	375	400	443	378	710	671	722	765

K07346 fimbrial chaperone protein	3012	3090	2860	3003	1673	1702	1671	1796	1535	1499	1517	1563
K07390 monothiol glutaredoxin	676	663	678	691	387	394	370	454	395	411	375	448
K07533 foldase protein PrsA [EC:5.2.1.8]	418	429	374	437	128	117	100	102	404	471	430	439
K09488 TNF receptor-associated protein 1	3	0	2	3	1	2	0	3	3	4	2	1
K09510 DnaJ homolog subfamily B member 4	0	0	0	0	0	1	0	0	0	0	0	0
K09569 FK506-binding protein 2 [EC:5.2.1.8]	4	2	2	3	6	3	8	4	4	4	14	3
K09575 FK506-binding protein 9/10 [EC:5.2.1.8]	4	4	4	2	5	7	2	3	5	7	9	5
K09796 periplasmic copper chaperone A	784	760	733	742	419	394	346	478	256	258	220	264
K19777 acid stress chaperone HdeA	0	0	0	0	3	2	2	0	12	13	11	11
K19778 acid stress chaperone HdeB	238	302	256	246	76	79	84	73	509	573	545	563
K21804 protein N-lysine methyltransferase METTL21A [EC:2.1.1.-]	0	0	0	0	1	0	0	0	0	0	0	0
K22447 archaeal chaperonin	5	2	6	3	5	1	5	2	5	25	8	23
K23349 putative dimethyl sulfoxide reductase chaperone	339	352	319	345	239	259	283	257	523	496	512	539
ko04131 Membrane trafficking	33126	33650	33901	33584	38836	38843	39242	39087	39286	38833	39803	39386
K08332 vacuolar protein 8	0	0	2	0	0	0	0	0	0	0	0	0
K08518 syntaxin-binding protein 5	0	0	0	0	0	0	0	0	0	4	0	0
K10402 kinesin family member 20	0	0	0	0	0	0	0	1	0	0	0	0
K17922 sorting nexin-8	0	2	0	0	0	0	0	0	0	0	0	0
K19950 protein transport protein SEC9	0	0	1	0	0	0	0	0	0	0	0	0
K19998 sec1 family domain-containing protein 1	0	0	0	0	0	0	0	0	0	0	2	0
K20280 trafficking protein particle complex subunit 5	0	0	0	0	0	0	0	0	0	0	0	3
K20352 p24 family protein delta-1	0	0	0	0	0	0	0	0	0	0	2	0
K20456 oxysterol-binding protein 1	0	0	1	0	0	0	0	0	0	0	0	0
K21440 ankyrin repeat domain-containing protein 50	0	0	0	0	1	2	0	0	0	2	0	1
K23481 vacuolar protein sorting-associated protein 1	0	0	1	0	0	0	0	0	0	0	0	0
ko04121 Ubiquitin system	7	16	14	9	4	2	2	1	11	5	10	3
K10273 F-box and leucine-rich repeat protein 7	0	0	3	0	0	0	0	0	0	0	0	0
K11997 tripartite motif-containing protein 2/3	0	0	0	0	0	0	0	0	0	0	1	0
K12025 tripartite motif-containing protein 52	0	0	0	0	0	0	0	0	0	1	0	1
K13570 prokaryotic ubiquitin-like protein Pup	0	1	0	0	1	0	2	0	3	0	0	0
K15071 recyclin-1	0	1	0	0	0	0	0	0	0	0	0	0
K22372 E3 ISG15--protein ligase [EC:2.3.2.-]	1	0	0	0	0	0	0	1	0	0	0	0
K22382 WD repeat-containing protein 26	0	1	0	0	0	0	0	0	0	0	0	0
K23333 E3 ubiquitin-protein transferase RMND5 [EC:2.3.2.27]	0	0	0	0	0	1	0	0	0	0	0	0
ko03051 Proteasome	8035	8045	8198	8234	11373	11387	11460	11438	9455	9451	9563	9728
K13571 proteasome accessory factor A [EC:6.3.1.19]	2	5	6	4	0	5	6	3	5	17	12	20
K13573 proteasome accessory factor C	0	1	0	0	0	0	0	0	0	1	0	0
K20814 Pup amidohydrolase [EC:3.5.1.119]	3	3	4	2	3	9	1	9	6	4	4	15
ko03032 DNA replication proteins	151073	152131	154561	153379	177539	179508	179646	177943	166333	165020	164970	164458
K02315 DNA replication protein DnaC	2707	2698	2756	2779	1833	1810	1708	1755	1843	1918	1784	1820
K02469 DNA gyrase subunit A [EC:5.6.2.2]	9652	9879	9806	9889	9055	9280	9264	8947	9623	9891	9375	9607
K02470 DNA gyrase subunit B [EC:5.6.2.2]	8651	8590	8771	8696	8339	8597	8787	8460	8779	8915	8840	8797
K02621 topoisomerase IV subunit A [EC:5.6.2.2]	6129	6165	6441	6389	9185	9111	9095	9237	7947	7459	7545	7576
K02622 topoisomerase IV subunit B [EC:5.6.2.2]	5161	5311	5380	5383	6896	6807	6965	6869	5736	5672	5541	5578
K03163 DNA topoisomerase I [EC:5.6.2.1]	0	0	0	0	0	0	0	0	0	0	0	1
K03166 DNA topoisomerase VI subunit A [EC:5.6.2.2]	0	0	1	0	2	4	2	0	8	10	10	12
K03167 DNA topoisomerase VI subunit B [EC:5.6.2.2]	1	2	4	0	0	0	0	5	5	8	8	12
K03168 DNA topoisomerase I [EC:5.6.2.1]	10958	10964	10899	10779	10407	10381	10044	10254	8920	8753	8391	8541
K03169 DNA topoisomerase III [EC:5.6.2.1]	10565	10897	11225	10763	20071	20242	20277	20056	18116	17946	17799	17620
K03346 replication initiation and membrane attachment protein	72	69	69	80	21	22	22	15	90	86	82	66
K03530 DNA-binding protein HU-beta	1924	1902	1999	1869	2945	2860	2951	2919	2340	2348	2316	2349
K03645 negative modulator of initiation of replication	320	347	344	421	280	256	215	257	567	579	581	540
K04764 integration host factor subunit alpha	570	644	628	608	338	365	343	366	383	389	369	380
K05787 DNA-binding protein HU-alpha	648	629	600	639	384	392	353	396	395	378	363	362
K05788 integration host factor subunit beta	708	646	669	657	451	449	399	392	378	437	400	424
K09723 DNA replication factor GINS	0	0	0	0	2	0	2	0	2	4	4	7
K10725 archaeal cell division control protein 6	0	3	2	0	2	2	0	3	8	8	13	20
K10730 ATP-dependent DNA helicase Q4 [EC:3.6.4.12]	0	0	0	0	0	0	0	0	0	1	0	0
K10748 DNA replication terminus site-binding protein	502	498	474	503	321	375	372	352	625	712	691	721
K10763 DnaA-homolog protein	1473	1357	1325	1521	816	920	807	923	888	961	964	990
K10900 werner syndrome ATP-dependent helicase [EC:3.6.4.12]	0	0	0	0	0	0	0	0	0	1	0	0
K11144 primosomal protein DnaI	48	51	56	58	5	12	8	20	70	77	47	61
K18996 replication initiation protein RepC	0	0	0	0	0	0	0	1	11	2	3	4
K19783 DNA polymerase alpha-associated DNA helicase A [EC:3.6.4.12]	0	0	0	1	0	0	0	0	0	0	0	0
ko03036 Chromosome and associated proteins	178583	179508	180946	180709	189001	190387	188782	189035	187482	187266	185156	185336
K02499 tetrapyrrole methylase family protein / MazG family protein	198	204	203	193	83	105	109	109	224	241	253	259
K03466 DNA segregation ATPase FtsK/SpoIIIE, S-DNA-T family	7822	7851	8020	8036	8695	8577	8809	8863	8163	8314	8328	8095
K03496 chromosome partitioning protein	10687	10580	10690	11012	10427	10397	10027	10084	10784	10712	10585	10757
K03528 cell division protein ZipA	1434	1300	1413	1334	867	818	897	928	826	821	744	866
K03529 chromosome segregation protein	5563	5471	5316	5630	2683	2762	2556	2873	2641	2860	2749	2900
K03569 rod shape-determining protein MreB and related proteins	3986	3959	3848	3778	3953	4016	4010	4027	3957	4028	4044	4099
K03570 rod shape-determining protein MreC	1859	1920	1876	1977	2738	2820	2834	2649	2788	2695	2794	2720
K03571 rod shape-determining protein MreD	1215	1230	1163	1186	689	653	642	700	836	954	852	853
K03586 cell division protein FtsL	626	637	614	642	444	441	383	364	461	435	432	446
K03591 cell division protein FtsN	422	389	385	427	242	334	341	328	555	535	512	541
K03593 ATP-binding protein involved in chromosome partitioning	1548	1698	1765	1759	3216	3212	3222	3220	2954	2846	2830	2717
K03608 cell division topological specificity factor	638	683	660	709	347	338	306	333	470	478	472	474
K03609 septum site-determining protein MinD	2157	2167	2114	2092	1138	1037	1118	1101	1607	1654	1599	1588
K03610 septum site-determining protein MinC	1811	1723	1808	1886	1014	910	934	930	1209	1229	1156	1194
K03632 chromosome partition protein MukB	2380	2490	2360	2512	1641	1864	1810	1696	3661	3591	3356	3642
K03633 chromosome partition protein MukF	716	758	721	699	571	495	512	523	920	1040	1009	1000
K03733 integrase/recombinase XerC	2830	2719	2859	2801	3299	3292	3348	3424	3124	2899	2919	2916
K03746 DNA-binding protein H-NS	311	350	343	343	278	323	289	255	418	433	434	485
K03804 chromosome partition protein MukE	398	362	372	393	264	274	284	273	539	560	563	576
K03978 GTP-binding protein	1740	1747	1829	1743	2027	2042	2009	1885	1869	1917	2015	2011
K04047 starvation-inducible DNA-binding protein	1332	1243	1314	1233	1646	1664	1662	1716	1400	1359	1383	1304
K04074 cell division initiation protein	54	57	58	74	30	38	41	34	56	100	78	63
K04095 cell filamentation protein	558	556	473	542	329	395	342	307	678	689	606	773
K04763 integrase/recombinase XerD	2633	2665	2764	2729	3232	3281	3336	3182	3359	3467	3275	3226
K05589 cell division protein FtsB	565	550	545	542	357	354	354	401	356	369	341	351
K05837 rod shape determining protein RodA	3830	4058	4098	4093	4749	4924	4912	4739	4470	4271	4329	4254
K05896 segregation and condensation protein A	1765	1740	1570	1756	824	844	811	782	768	874	796	841
K06024 segregation and condensation protein B	1625	1579	1523	1568	862	847	909	993	746	728	681	680
K06286 septation ring formation regulator	106	114	104	124	42	31	32	23	98	159	102	136
K06899 nucleoid-associated protein	3001	3015	2958	2856	2158	2235	2085	2182	1552	1710	1701	1715
K06916 cell division protein ZapE	5184	5146	4922	5163	2751	2688	2647	2840	3225	3138	3113	3099
K09772 cell division inhibitor SepF	254	234	224	251	83	108	77	50	215	255	248	238
K09888 cell division protein ZapA	1161	1130	1153	1071	1082	1038	1005	1141	1042	1125	1000	1023
K09892 cell division protein ZapB	683	651	669	659	401	398	382	383	373	415	384	380
K11267 sister chromatid cohesion protein PDS5	0	0	0	0	1	0	0	0	0	0	0	0
K11274 chromosome transmission fidelity protein 4	0	0	0	0	0	2	0	0	0	0	0	0
K11396 chromatin modification-related protein YNG2	0	2	0	0	0	0	0	0	0	0	0	0
K11494 RCC1 and BTB domain-containing protein	4	3	2	2	0	0	0	0	1	0	1	1
K11673 actin-related protein 8	0	0	0	0	0	0	0	0	0	0	1	0
K11685 DNA-binding protein StpA	137	142	138	158	59	62	42	48	342	334	353	372
K11940 heat shock protein HspQ	213	164	184	199	118	143	140	135	271	276	281	293
K12961 DnaA initiator-associating protein	327	407	377	389	286	269	258	243	574	617	444	555
K13052 cell division protein DivIC	51	31	30	43	2	21	3	9	48	35	34	36
K13053 cell division inhibitor SulA	986	994	1042	1014	536	609	628	577	632	546	539	598
K14440 SWI/SNF-related matrix-associated actin-dependent regulator of chromatin subfamily A-like protein 1 [EC:3.6.4.12]	3	0	2	1	2	2	4	5	0	1	2	0
K14967 COMPASS component BRE2	0	0	0	0	1	0	0	0	0	0	0	0
K16570 gamma-tubulin complex component 3	0	0	0	0	0	2	0	1	0	0	0	1
K16732 protein regulator of cytokinesis 1	0	1	0	0	0	0	0	0	0	0	0	0
K16803 cytoskeleton-associated protein 5	0	0	0	0	0	0	0	0	0	0	2	0
K18657 cell division protein ZapC	275	245	294	309	216	249	218	201	384	379	353	418
K18778 cell division protein ZapD	488	500	512	500	376	397	387	360	695	692	622	705
K18997 chaperone modulatory protein CbpM	131	131	139	126	35	43	35	48	281	346	266	331
K19678 intraflagellar transport protein 80	0	0	0	0	0	0	1	0	0	1	0	0
K19788 obg-like ATPase 1	0	0	0	0	2	0	0	0	0	0	0	1
K20092 chromodomain-helicase-DNA-binding protein 1-like [EC:3.6.4.12]	5	6	5	3	23	27	22	21	14	17	19	17
K23398 activating signal cointegrator 1	11	9	17	5	21	22	20	19	18	22	8	20

ko03400 DNA repair and recombination proteins	411948	410576	410376	412556	402842	407222	405997	403014	407858	410156	410379	408771
K00567 methylated-DNA-[protein]-cysteine S-methyltransferase [EC:2.1.1.63]	1684	1680	1778	1632	1960	1943	1952	1883	1808	1833	1819	1922
K01160 crossover junction endodeoxyribonuclease RusA [EC:3.1.22.4]	104	100	114	121	143	192	169	161	68	77	81	61
K01554 8-oxo-dGDP phosphatase [EC:3.6.1.58]	0	0	0	0	0	0	0	0	1	1	2	1
K01669 deoxyribodipyrimidine photo-lyase [EC:4.1.99.3]	3191	3186	3009	3114	1839	1875	1794	2010	1818	1818	1839	2016
K02336 DNA polymerase II [EC:2.7.7.7]	3949	3857	3791	3818	2259	2170	2250	2253	2646	2819	2737	2800
K02346 DNA polymerase IV [EC:2.7.7.7]	3076	3189	3143	3199	3500	3641	3594	3485	4058	4154	4266	4237
K02347 DNA polymerase (family X)	3	0	2	1	0	0	0	0	0	0	0	0
K03502 DNA polymerase V	2395	2456	2395	2510	1939	2308	2196	2052	2687	2746	2620	2658
K03546 DNA repair protein SbcC/Rad50	7414	7508	7485	7672	9226	9480	9783	9107	8859	8461	8697	8576
K03547 DNA repair protein SbcD/Mre11	4770	4754	4857	4903	4971	4938	5068	4854	4891	5084	5054	4930
K03552 holliday junction resolvase Hjr [EC:3.1.22.4]	0	3	3	1	2	7	12	6	3	3	0	0
K03554 recombination associated protein RdgC	1589	1600	1554	1630	977	1008	934	997	1097	1119	1108	1044
K03565 regulatory protein	1907	1844	1895	1923	2082	2144	2134	1936	1905	1726	1908	1809
K03574 8-oxo-dGTP diphosphatase [EC:3.6.1.55]	3398	3384	3271	3459	1725	1766	1818	1754	1884	1891	1799	1900
K03631 DNA repair protein RecN (Recombination protein N)	5665	6045	5666	5678	6048	5758	5833	5811	5582	5245	5239	5293
K03656 ATP-dependent DNA helicase Rep [EC:3.6.4.12]	4877	4823	4625	4869	2931	3008	3013	2892	3073	3046	2926	3036
K03658 DNA helicase IV [EC:3.6.4.12]	1268	1208	1245	1205	781	895	904	839	1518	1585	1447	1473
K03700 recombination protein U	278	261	240	266	82	72	94	67	217	292	202	265
K03722 ATP-dependent DNA helicase DinG [EC:3.6.4.12]	6344	6227	6006	6172	3595	3628	3661	3753	4745	4902	4800	4769
K03724 ATP-dependent helicase Lhr and Lhr-like helicase [EC:3.6.4.-]	11378	11248	10753	11170	6527	6567	6273	6589	4624	4305	4271	4528
K03919 DNA oxidative demethylase [EC:1.14.11.33]	360	378	370	378	251	327	315	282	523	506	556	546
K04483 DNA repair protein RadA	0	2	0	0	0	0	2	0	2	12	3	5
K04484 DNA repair protein RadB	1	0	2	1	1	0	0	0	4	0	3	5
K04485 DNA repair protein RadA/Sms	4215	4142	4280	4225	4866	4942	4895	4985	4669	4670	4839	4679
K05982 deoxyribonuclease V [EC:3.1.21.7]	429	427	470	471	346	302	310	318	544	505	547	586
K05984 excinuclease Cho [EC:3.1.25.-]	434	463	432	481	293	372	314	283	623	606	602	684
K07455 recombination protein RecT	311	371	410	416	188	222	214	189	172	177	141	180
K07458 DNA mismatch endonuclease, patch repair protein [EC:3.1.-.-]	603	668	632	659	1445	1404	1500	1500	1348	1290	1293	1430
K10906 exodeoxyribonuclease VIII [EC:3.1.11.-]	78	75	59	96	0	4	3	1	5	3	1	2
K12149 DNA-damage-inducible protein I	143	160	134	156	107	89	114	119	251	246	262	263
K13531 methylated-DNA-[protein]-cysteine S-methyltransferase [EC:2.1.1.63]	25	15	10	18	16	16	6	4	16	16	17	15
K14160 protein ImuA	0	0	0	0	2	0	0	2	2	2	3	0
K14161 protein ImuB	2137	2024	2029	2181	1190	1066	1057	1241	700	647	658	624
K14162 error-prone DNA polymerase [EC:2.7.7.7]	4188	4165	4180	4136	2446	2297	2223	2362	1445	1469	1233	1340
K14623 DNA-damage-inducible protein D	781	872	797	828	1713	1611	1765	1641	1923	1788	1764	1874
K14635 ATP-dependent DNA helicase MPH1 [EC:3.6.4.12]	0	0	0	0	0	0	0	1	0	0	0	0
K15083 DNA repair protein RAD16	0	0	0	0	0	0	0	0	0	1	0	0
K15342 CRISP-associated protein Cas1	1315	1290	1343	1315	871	839	829	833	1332	1443	1448	1433
K15505 DNA repair protein RAD5 [EC:3.6.4.-]	0	0	1	0	0	0	0	0	0	0	0	0
K16898 ATP-dependent helicase/nuclease subunit A [EC:3.1.-.- 3.6.4.12]	1673	1588	1653	1628	528	494	488	500	1500	1730	1529	1664
K16899 ATP-dependent helicase/nuclease subunit B [EC:3.1.-.- 3.6.4.12]	1490	1466	1439	1459	511	519	484	459	1288	1617	1398	1557
K17816 8-oxo-dGTP diphosphatase / 2-hydroxy-dATP diphosphatase [EC:3.6.1.55 3.6.1.56]	2	0	0	0	0	2	0	0	0	0	0	0
K19789 DNA repair protein RadD	1025	1036	895	1019	670	772	725	633	1307	1431	1413	1422
K22585 DNA repair protein NreA	0	0	2	1	0	1	0	0	8	7	8	12
K22803 structural maintenance of chromosomes protein 5	0	0	0	0	0	0	0	0	0	0	1	0
K23258 8-oxo-dGTP phosphatase [EC:3.6.1.-]	6	5	7	1	7	2	8	7	8	10	12	9
K23387 golgi to ER traffic protein 4	0	1	0	0	0	0	0	0	0	0	0	0
ko03029 Mitochondrial biogenesis	136544	136136	139445	138257	146275	148240	147226	146676	136714	137422	136852	136371
K07152 protein SCO1/2	1060	1040	963	1046	608	552	571	653	332	327	334	319
K07277 outer membrane protein insertion porin family	7870	7983	8335	8113	9370	9474	9494	9579	9689	9646	9526	9529
K10908 DNA-directed RNA polymerase, mitochondrial [EC:2.7.7.6]	0	0	0	0	0	0	0	1	0	0	0	0
K14998 surfeit locus 1 family protein	3	2	1	0	1	2	0	0	1	4	3	4
K15980 acyl-CoA dehydrogenase family member 9 [EC:1.3.99.-]	1	0	1	1	0	0	0	0	1	0	0	0
K17662 cytochrome b pre-mRNA-processing protein 3	0	0	1	0	3	0	1	0	3	2	1	0
K17675 ATP-dependent RNA helicase SUPV3L1/SUV3 [EC:3.6.4.13]	113	93	97	106	81	89	79	68	71	97	80	79
K17677 ATP-dependent helicase IRC3 [EC:3.6.4.-]	0	0	2	0	0	0	0	0	0	0	0	0
K17679 ATP-dependent RNA helicase MSS116, mitochondrial [EC:3.6.4.13]	0	0	1	0	0	0	0	0	0	0	0	0
K17680 twinkle protein [EC:3.6.4.12]	390	474	454	389	551	604	508	568	55	62	94	63
K17768 mitochondrial import receptor subunit TOM70	0	2	0	0	0	0	0	0	0	0	0	0
K17807 mitochondrial translocator assembly and maintenance protein 41	0	0	1	0	0	0	0	0	0	0	0	0
K17970 mitochondrial division protein 1	0	0	0	0	0	0	0	0	0	2	0	0
K21594 translation factor GUF1, mitochondrial [EC:3.6.5.-]	1	0	3	0	3	2	1	6	0	3	0	0
K22140 dynamin-like GTPase MGM1, mitochondrial	0	0	0	0	0	1	0	0	0	0	2	0
K23164 reticulon-4-interacting protein 1, mitochondrial	0	0	0	0	0	1	0	0	0	0	0	0
Protein families: signaling and cellular processes	3127866	3111743	3058229	3107297	2539537	2559854	2507899	2546416	2706008	2727809	2678755	2727025
ko02000 Transporters	1995062	1986872	1951522	1985628	1667605	1682584	1655177	1673328	1836688	1854918	1822917	1858054
K00427 L-lactate permease	737	674	752	728	201	229	209	219	1513	1507	1530	1450
K01551 arsenite/tail-anchored protein-transporting ATPase [EC:7.3.2.7 7.3.-.-]	1647	1525	1526	1657	2639	2848	2865	2629	534	457	558	528
K01604 methylmalonyl-CoA decarboxylase subunit alpha [EC:7.2.4.3]	1567	1633	1821	1719	3293	3430	3455	3250	3173	3189	3215	3053
K01989 putative ABC transport system substrate-binding protein	2450	2435	2356	2419	1299	1313	1229	1270	1383	1400	1364	1430
K01990 ABC-2 type transport system ATP-binding protein	15758	15670	15717	16004	15108	15149	15054	14821	19362	19930	19765	19895
K01992 ABC-2 type transport system permease protein	20466	20595	20855	20092	28545	28480	28914	28787	27918	27762	27673	27841
K02003 putative ABC transport system ATP-binding protein	12512	12289	12210	12554	11986	11650	11653	11749	13459	13674	13478	14122
K02004 putative ABC transport system permease protein	22775	22823	22800	22817	23143	23266	23652	23184	25120	25598	25341	25783
K02013 iron complex transport system ATP-binding protein [EC:7.2.2.-]	9086	8997	8694	8831	8791	9058	8858	8963	9168	9179	9113	9242
K02014 iron complex outermembrane recepter protein	74685	75008	71861	73145	55899	54729	53641	57046	42642	40910	40986	41452
K02015 iron complex transport system permease protein	15581	15376	14840	15255	13547	13429	13594	13981	13661	13463	13634	14060
K02016 iron complex transport system substrate-binding protein	10307	10105	9747	10109	9270	9194	8895	9323	9705	9900	10011	9985
K02021 putative ABC transport system ATP-binding protein	0	0	0	0	0	0	0	1	0	0	0	0
K02024 maltoporin	2821	2761	2790	2888	1824	1984	1809	1807	3894	4069	3954	4337
K02025 multiple sugar transport system permease protein	15750	14967	15375	15583	3515	3592	3316	3153	14524	15690	14914	15610
K02026 multiple sugar transport system permease protein	14983	14174	14635	14438	3272	3438	3271	3016	13812	15708	14519	15180
K02027 multiple sugar transport system substrate-binding protein	15180	15003	15202	15533	3487	3748	3633	3343	14730	16296	15827	15828
K02028 polar amino acid transport system ATP-binding protein [EC:7.4.2.1]	4117	3916	3601	4002	2076	2095	2159	1978	4158	4258	4157	4270
K02029 polar amino acid transport system permease protein	7663	7491	7173	7462	4810	4782	4689	4730	5456	5759	5260	5718
K02030 polar amino acid transport system substrate-binding protein	16306	16048	15482	16113	9011	9297	8854	9580	9705	9683	9481	9826
K02049 NitT/TauT family transport system ATP-binding protein	5457	5256	5017	5182	2847	3150	2750	2977	3519	3559	3351	3740
K02050 NitT/TauT family transport system permease protein	5152	5028	4570	4919	2990	3106	2963	3000	3271	3301	3097	3453
K02051 NitT/TauT family transport system substrate-binding protein	6936	6693	6507	6882	5262	5221	5086	5341	6081	6169	5933	6209
K02056 simple sugar transport system ATP-binding protein [EC:7.5.2.-]	8099	7744	7591	7783	4083	4169	4000	4108	6604	6868	6519	6897
K02057 simple sugar transport system permease protein	9377	8980	8559	9025	4311	4480	4352	4369	6831	7336	6895	7344
K02058 simple sugar transport system substrate-binding protein	3564	3368	3234	3404	2143	2125	1991	2156	2765	2740	2582	2697
K02068 putative ABC transport system ATP-binding protein	699	673	715	701	1391	1238	1296	1243	1431	1430	1452	1511
K02069 putative ABC transport system permease protein	805	820	843	889	1650	1626	1614	1681	1781	1867	1749	1688
K02074 zinc/manganese transport system ATP-binding protein	528	513	453	543	300	307	326	322	667	842	721	743
K02075 zinc/manganese transport system permease protein	497	487	498	512	311	334	297	341	650	616	630	669
K02077 zinc/manganese transport system substrate-binding protein	508	519	525	549	382	375	359	368	609	624	660	682
K02100 MFS transporter, SP family, arabinose:H+ symporter	774	766	711	848	534	556	565	508	1242	1235	1169	1190
K02168 choline/glycine/proline betaine transport protein	6289	5989	6187	6310	3389	3299	3194	3425	3303	3496	3280	3383
K02198 cytochrome c-type biogenesis protein CcmF	3415	3319	3064	3154	1693	1799	1799	1980	2540	2477	2511	2569
K02205 arginine/ornithine permease	2115	2003	1885	2062	1103	1098	1027	1210	753	662	558	559
K02429 MFS transporter, FHS family, L-fucose permease	3322	3598	3642	3406	8546	8920	8712	8532	6475	6569	6630	6628
K02440 glycerol uptake facilitator protein	1451	1445	1401	1525	756	877	762	780	1612	1701	1705	1704
K02445 MFS transporter, OPA family, glycerol-3-phosphate transporter	3479	3529	3410	3697	1864	1876	1869	1937	2129	2075	1933	2240
K02511 MFS transporter, ACS family, 4-hydroxyphenylacetate permease	519	587	569	554	163	178	151	141	1037	1171	1021	1068
K02526 2-keto-3-deoxygluconate permease	1009	1019	1072	1107	1190	1197	1110	1054	875	915	826	859
K02532 MFS transporter, OHS family, lactose permease	3396	3314	3081	3273	1889	2050	1948	1842	4822	5035	4644	4988
K02550 glycolate permease	7	9	10	11	19	16	19	21	68	86	77	82
K02598 nitrite transporter	195	198	255	234	262	290	295	283	159	160	125	144
K02625 MFS transporter, MHS family, dicarboxylic acid transporter PcaT	1967	1839	1849	1861	1031	1060	1006	1131	657	610	558	592
K02824 uracil permease	3912	4241	4036	4220	4112	4080	3809	3998	4230	4086	4011	3993
K02856 L-rhamnose-H+ transport protein	1029	1107	1097	1055	2250	2243	2406	2276	2463	2356	2394	2293
K03282 large conductance mechanosensitive channel	1276	1359	1399	1412	1342	1243	1547	1501	1300	1271	1271	1318
K03284 magnesium transporter	4464	4535	4778	4542	5996	6222	6328	6111	5704	5578	5543	5563
K03286 OmpA-OmpF porin, OOP family	2269	2375	2168	2248	1424	1456	1416	1379	1483	1376	1453	1404
K03288 MFS transporter, MHS family, citrate/tricarballylate:H+ symporter	768	759	664	774	208	211	258	217	1543	1462	1396	1574
K03289 MFS transporter, NHS family, nucleoside permease	693	790	748	840	538	589	536	522	1117	1183	1131	1152
K03290 MFS transporter, SHS family, sialic acid transporter	332	326	291	330	494	504	476	481	119	99	98	111
K03291 MFS transporter, SET family, sugar efflux transporter	1111	1162	1003	1151	673	607	623	608	1754	1864	1807	1925
K03297 small multidrug resistance pump	588	581	486	542	374	371	331	376	187	174	177	207

K03298 drug/metabolite transporter, DME family	1	2	2	3	3	1	2	0	3	2	3	4
K03299 gluconate:H+ symporter, GntP family	5605	5622	5726	5728	4772	4715	4596	4503	4696	4761	4722	4740
K03303 lactate permease	3725	3487	3765	3611	4623	4677	4534	4498	3263	3066	3295	3077
K03304 tellurite resistance protein	500	510	533	550	348	346	381	338	855	734	733	737
K03312 glutamate:Na+ symporter, ESS family	2624	2500	2466	2458	1298	1495	1243	1414	1744	1808	1587	1820
K03313 Na+:H+ antiporter, NhaA family	1835	1825	1790	1764	3329	3245	3426	3241	2793	2685	2725	2706
K03314 Na+:H+ antiporter, NhaB family	1214	1199	1188	1134	500	480	443	419	1496	1456	1372	1491
K03315 Na+:H+ antiporter, NhaC family	2338	2446	2629	2346	3157	3165	3298	3195	3753	3865	3740	3579
K03318 tyrosine permease	0	2	1	2	0	2	0	0	0	0	1	0
K03320 ammonium transporter, Amt family	5470	5257	5260	5303	3946	4077	4340	4357	4263	4364	4142	4165
K03321 sulfate permease, SulP family	8560	8442	8326	8405	8420	8404	8154	8151	7011	6808	6710	6796
K03322 manganese transport protein	5824	5632	5244	5491	5080	5096	5105	5301	4239	4111	3976	4098
K03324 phosphate:Na+ symporter	10364	10191	9896	10012	8454	8660	7977	8418	7764	7604	7419	7624
K03325 arsenite transporter	409	473	505	451	1508	1367	1427	1385	1207	1178	1188	1129
K03326 C4-dicarboxylate transporter, DcuC family	1668	1709	1655	1711	1528	1685	1545	1617	2607	2640	2617	2735
K03327 multidrug resistance protein, MATE family	9981	9976	9482	9723	10092	9956	10059	10504	8810	8311	8595	8584
K03329 amino acid exporter	2	0	0	1	7	8	3	12	39	37	41	31
K03442 small conductance mechanosensitive channel	2944	2959	2712	2837	3050	3270	3217	3236	2817	2883	2812	2819
K03444 MFS transporter, SP family, sugar porter, other	0	2	5	0	0	2	2	0	2	0	0	0
K03445 MFS transporter, DHA1 family, purine ribonucleoside efflux pump	797	824	734	786	559	617	570	516	1272	1245	1148	1264
K03446 MFS transporter, DHA2 family, multidrug resistance protein	9206	8877	8779	8837	5603	5810	5574	5735	4422	4657	4267	4615
K03449 MFS transporter, CP family, cyanate transporter	5985	6012	5416	5870	3325	3363	3250	3636	3339	3247	3187	3375
K03459 formate transporter	0	0	0	0	11	7	8	7	5	4	8	3
K03468 p-hydroxybenzoic acid efflux pump subunit AaeB	1189	1229	1197	1251	923	997	850	872	1650	1737	1655	1666
K03498 trk system potassium uptake protein	6540	6150	6474	6269	5396	5499	5585	5508	6476	6752	6624	6798
K03499 trk system potassium uptake protein	6363	6470	6526	6353	6863	6957	6645	6717	7071	7199	6705	7179
K03535 MFS transporter, ACS family, glucarate transporter	1619	1656	1678	1786	1413	1614	1457	1437	2039	1958	1896	2057
K03543 membrane fusion protein, multidrug efflux system	7714	7735	7525	7461	6920	6632	6362	6499	5176	5201	4948	5145
K03548 putative permease	2158	2040	2087	2085	1276	1260	1227	1277	1273	1292	1279	1291
K03549 KUP system potassium uptake protein	4586	4672	4569	4721	3150	3239	3066	3190	3050	3007	2901	3025
K03559 biopolymer transport protein ExbD	2244	2081	2169	2197	3395	3416	3444	3359	2556	2630	2631	2471
K03560 biopolymer transport protein TolR	1607	1454	1406	1486	844	839	803	889	738	686	657	725
K03561 biopolymer transport protein ExbB	6350	6278	6177	6103	9149	8671	9081	8991	6613	6411	6445	6502
K03562 biopolymer transport protein TolQ	1405	1417	1511	1494	932	940	853	895	893	959	937	915
K03640 peptidoglycan-associated lipoprotein	984	1048	1071	1114	689	603	669	661	719	752	740	699
K03641 TolB protein	2490	2392	2443	2496	1436	1609	1506	1480	1707	1703	1614	1722
K03643 LPS-assembly lipoprotein	1026	1053	957	1058	656	662	532	633	714	723	733	764
K03646 colicin import membrane protein	1151	1163	1235	1255	669	696	661	648	1004	1074	1001	992
K03756 putrescine:ornithine antiporter	183	167	175	148	246	264	231	265	310	330	315	345
K03757 cadaverine:lysine antiporter	724	696	654	695	396	380	357	353	1388	1519	1472	1418
K03758 arginine:ornithine antiporter / lysine permease	3851	3720	3725	3926	2251	2275	2097	2397	2066	2062	1916	2060
K03759 arginine:agmatine antiporter	41	54	43	41	29	34	24	16	166	187	193	181
K03761 MFS transporter, MHS family, alpha-ketoglutarate permease	2882	3020	2856	2870	1727	1758	1736	1748	1683	1785	1751	1797
K03762 MFS transporter, MHS family, proline/betaine transporter	1432	1378	1372	1480	1380	1417	1344	1273	1318	1444	1196	1353
K03811 nicotinamide mononucleotide transporter	1727	1670	1593	1689	1730	1744	2044	2038	1677	1723	1669	1723
K03832 periplasmic protein TonB	5242	5066	5309	5333	8523	8533	8739	8627	6280	6128	6475	6249
K03834 tyrosine-specific transport protein	671	660	586	702	468	543	483	438	938	1060	986	1014
K03835 tryptophan-specific transport protein	3550	3747	3515	3612	2640	2562	2478	2591	1885	1904	1754	1912
K03836 low affinity tryptophan permease	2	6	5	8	17	25	13	13	98	105	102	90
K03837 serine transporter	1399	1389	1342	1364	1083	1141	992	1048	1845	2085	1828	1884
K03838 threonine transporter	385	417	384	378	121	125	130	111	875	885	883	877
K03893 arsenical pump membrane protein	2613	2575	2335	2528	1977	2109	2184	2086	1312	1388	1373	1355
K04744 LPS-assembly protein	6170	6260	6331	6229	3878	3815	3648	3765	3632	3815	3658	3821
K04758 ferrous iron transport protein A	991	1040	991	951	457	508	485	495	801	922	842	920
K04759 ferrous iron transport protein B	10206	10046	10215	10310	9331	9607	9350	9480	10819	10835	10638	10977
K05020 glycine betaine transporter	153	115	118	114	105	121	119	133	227	259	265	265
K05041 solute carrier family 6 (neurotransmitter transporter, creatine) member 8	3	2	0	0	0	0	0	0	0	0	0	0
K05245 L-carnitine/gamma-butyrobetaine antiporter	389	319	354	308	530	588	507	538	108	121	133	130
K05340 glucose uptake protein	757	796	807	864	2381	2347	2435	2335	1661	1621	1716	1618
K05373 MFS transporter, putative signal transducer	485	478	485	512	123	158	162	147	1052	1205	1132	1154
K05517 nucleoside-specific channel-forming protein	2010	2036	1878	1806	1184	1119	1063	1280	1248	1271	1165	1244
K05548 MFS transporter, AAHS family, benzoate transport protein	2175	2258	2068	2029	1153	1221	1182	1266	749	659	622	676
K05559 multicomponent K+:H+ antiporter subunit A	3964	3873	3827	3762	2130	2136	1861	2126	1275	1204	1092	1171
K05560 multicomponent K+:H+ antiporter subunit C	529	491	473	459	295	331	267	280	159	155	136	152
K05561 multicomponent K+:H+ antiporter subunit D	2215	2234	2132	2167	1164	1166	1210	1241	707	623	683	711
K05562 multicomponent K+:H+ antiporter subunit E	653	649	657	753	364	367	299	265	229	215	197	200
K05563 multicomponent K+:H+ antiporter subunit F	367	361	393	369	205	188	207	184	126	103	108	124
K05564 multicomponent K+:H+ antiporter subunit G	490	542	457	536	300	219	276	278	177	134	180	156
K05565 multicomponent Na+:H+ antiporter subunit A	1	2	2	1	0	0	0	0	0	0	0	0
K05566 multicomponent Na+:H+ antiporter subunit B	119	121	108	113	50	43	56	40	148	138	151	124
K05567 multicomponent Na+:H+ antiporter subunit C	74	58	65	64	31	23	12	22	99	104	79	87
K05568 multicomponent Na+:H+ antiporter subunit D	592	495	509	530	230	240	191	195	597	684	635	623
K05569 multicomponent Na+:H+ antiporter subunit E	82	89	72	74	27	40	22	22	117	121	117	106
K05570 multicomponent Na+:H+ antiporter subunit F	21	19	9	17	7	11	14	19	26	24	19	17
K05571 multicomponent Na+:H+ antiporter subunit G	29	19	22	23	16	10	35	11	38	30	23	26
K05595 multiple antibiotic resistance protein	2176	2216	2201	2133	3491	3475	3544	3680	2647	2602	2768	2655
K05777 putative thiamine transport system substrate-binding protein	450	451	431	454	108	103	99	134	941	868	864	971
K05778 putative thiamine transport system permease protein	460	473	431	509	95	102	121	146	997	927	918	929
K05779 putative thiamine transport system ATP-binding protein	233	226	194	270	57	55	65	74	499	499	513	581
K05782 benzoate membrane transport protein	2391	2293	2342	2302	1343	1337	1270	1283	1405	1384	1340	1466
K05786 chloramphenicol-sensitive protein RarD	2529	2480	2332	2411	1582	1562	1541	1496	1380	1367	1365	1341
K05802 potassium-dependent mechanosensitive channel	7130	7157	6815	7210	4297	4188	4067	4150	4362	4353	4331	4320
K05807 outer membrane protein assembly factor BamD	2483	2383	2464	2572	2697	2900	2799	2791	2383	2225	2295	2322
K05819 MFS transporter, AAHS family, 3-hydroxyphenylpropionic acid transporter	423	463	368	427	104	115	107	112	832	805	825	811
K05820 MFS transporter, PPP family, 3-phenylpropionic acid transporter	3792	3807	3481	3587	2134	2038	1975	2213	2035	1943	1807	2210
K05832 putative ABC transport system permease protein	2073	2138	2061	2103	1077	1077	1084	1111	1065	1157	1156	1130
K05833 putative ABC transport system ATP-binding protein	1715	1895	1835	1729	897	891	877	931	988	1219	1040	1030
K05834 homoserine/homoserine lactone efflux protein	1323	1336	1248	1327	835	816	759	868	787	784	793	821
K05835 threonine efflux protein	367	302	313	317	263	311	262	266	381	433	374	388
K06076 long-chain fatty acid transport protein	5265	5057	5127	5149	5055	5389	5338	5314	4313	4297	4295	4267
K06141 MFS transporter, TsgA protein	452	470	498	463	138	186	157	163	1062	1034	982	1028
K06144 universal stress protein B	204	205	174	185	173	127	137	144	279	308	287	280
K06147 ATP-binding cassette, subfamily B, bacterial	22047	21960	22237	22378	8890	9173	8919	8750	20780	22532	21125	22209
K06148 ATP-binding cassette, subfamily C, bacterial	2179	2109	1971	1957	1109	1051	1085	1215	726	694	631	734
K06155 Gnt-I system high-affinity gluconate transporter	769	846	784	900	514	674	592	580	1052	1145	1024	1103
K06156 Gnt-I system low-affinity gluconate transporter	971	998	988	1027	688	645	606	657	1271	1266	1196	1203
K06157 Gnt-II system L-idonate transporter	286	308	283	296	441	477	406	385	66	85	87	74
K06186 outer membrane protein assembly factor BamE	1024	951	928	986	597	572	537	577	530	497	522	544
K06188 aquaporin Z	1922	1985	1892	1945	2176	2047	2312	2160	1848	1771	1921	1871
K06189 magnesium and cobalt transporter	1574	1670	1604	1632	1055	1062	986	1008	1180	1208	1142	1286
K06196 cytochrome c-type biogenesis protein	606	634	590	653	95	114	95	97	529	541	563	590
K06199 fluoride exporter	1325	1276	1200	1287	1434	1457	1453	1418	1045	1025	1093	1103
K06212 formate transporter	742	646	600	681	607	723	704	688	741	802	697	712
K06213 magnesium transporter	3583	3604	3747	3881	4179	4139	4066	4025	4859	5068	4903	5137
K06216 putative ribose uptake protein	53	54	58	62	8	14	3	3	39	48	40	41
K06296 spore germination protein KB	0	0	0	0	0	2	0	0	4	0	0	3
K06311 spore germination protein	0	2	0	0	0	0	0	0	0	0	0	0
K06409 stage V sporulation protein B	1486	1357	1446	1434	344	440	394	372	1237	1491	1341	1361
K06518 holin-like protein	889	902	853	887	1175	1176	1018	1138	1276	1178	1263	1269
K06600 chemosensory pili system protein ChpE	961	924	865	920	483	485	521	535	279	235	243	317
K06609 MFS transporter, SP family, major inositol transporter	0	0	0	0	0	0	0	0	0	0	1	0
K06610 MFS transporter, SP family, inositol transporter	0	0	0	1	8	4	3	0	4	2	4	0
K06895 L-lysine exporter family protein LysE/ArgO	648	553	562	613	358	360	354	354	602	664	690	635
K06901 putative MFS transporter, AGZA family, xanthine/uracil permease	8447	8269	8272	8372	4564	4755	4457	4680	6169	6558	6238	6662
K07027 glycosyltransferase 2 family protein	2826	2906	2784	2757	3259	3314	3280	3353	3120	2927	2981	2958
K07084 putative amino acid transporter	1745	1692	1648	1745	944	958	843	888	609	512	510	554
K07114 Ca-activated chloride channel homolog	6032	5957	6004	5771	8887	8877	8976	8950	6670	6443	6626	6404
K07156 copper resistance protein C	366	353	356	330	332	362	295	339	318	318	283	354
K07221 phosphate-selective porin OprO and OprP	3407	3515	3584	3566	2265	2136	2099	2261	1072	1092	1034	1033
K07224 iron uptake system component EfeO	664	688	576	669	374	416	497	400	883	843	861	872
K07230 periplasmic iron binding protein	121	134	102	122	190	229	159	214	5	10	8	7

K07238 zinc transporter, ZIP family	2672	2497	2512	2581	1457	1481	1406	1416	1696	1668	1648	1782
K07240 chromate transporter	4549	4438	4569	4682	6586	6715	6742	6610	4796	4602	4623	4666
K07241 nickel/cobalt transporter (NiCoT) family protein	396	426	354	482	107	114	114	104	806	792	792	827
K07243 high-affinity iron transporter	4349	4305	4301	4236	2865	2995	2734	2947	1833	1732	1686	1842
K07244 mgtE-like transporter	0	3	0	0	3	0	2	3	6	3	1	12
K07245 copper resistance protein D	808	785	772	836	707	851	852	758	685	662	734	736
K07267 porin	5767	5637	5273	5421	2752	2954	2669	2936	2663	2380	2478	2559
K07274 MipA family protein	379	403	409	461	310	312	327	349	632	647	643	622
K07278 translocation and assembly module TamA	3693	3687	3613	3750	2206	2290	2105	2196	2327	2400	2346	2417
K07279 autotransporter family porin	8	9	4	15	42	32	26	38	182	173	200	205
K07287 outer membrane protein assembly factor BamC	1507	1515	1549	1611	964	1045	996	1039	1101	1230	1169	1173
K07300 Ca2+:H+ antiporter	547	549	518	584	411	342	384	387	754	805	764	780
K07301 cation:H+ antiporter	1288	1339	1463	1313	2777	2802	2966	2833	2666	2661	2800	2536
K07311 Tat-targeted selenate reductase subunit YnfG	3	1	6	1	4	11	0	5	29	22	21	23
K07312 Tat-targeted selenate reductase subunit YnfH	452	487	461	486	313	373	377	330	662	617	590	671
K07490 ferrous iron transport protein C	337	371	368	374	215	189	283	294	262	269	244	245
K07507 putative Mg2+ transporter-C (MgtC) family protein	4279	4250	4062	4178	3228	3304	3154	3275	2850	2748	2811	2671
K07552 MFS transporter, DHA1 family, multidrug resistance protein	4107	4112	3907	4048	2400	2347	2044	2122	2941	3107	2914	2976
K07791 anaerobic C4-dicarboxylate transporter DcuA	1670	1719	1808	1732	3848	3807	3894	3723	3296	3329	3405	3388
K07862 serine/threonine transporter	1389	1466	1322	1382	945	1031	933	958	1512	1526	1429	1530
K08137 MFS transporter, SP family, galactose:H+ symporter	816	819	849	888	578	668	575	592	1198	1214	1147	1232
K08138 MFS transporter, SP family, xylose:H+ symportor	766	889	1001	881	2773	2805	2782	2921	1965	1919	1840	1696
K08151 MFS transporter, DHA1 family, tetracycline resistance protein	1810	1775	1744	1658	1063	1012	1009	1050	648	545	620	573
K08153 MFS transporter, DHA1 family, multidrug resistance protein	444	410	402	387	91	89	65	63	256	304	229	266
K08154 MFS transporter, DHA1 family, 2-module integral membrane pump EmrD	805	812	785	777	553	607	602	521	1095	1081	1046	1096
K08156 MFS transporter, DHA1 family, arabinose polymer utilization protein	2020	2117	2115	2082	5723	5901	5862	5657	4823	4399	4809	4498
K08159 MFS transporter, DHA1 family, L-arabinose/isopropyl-beta-D-thiogalactopyranoside export protein	2370	2392	2283	2355	1492	1494	1296	1450	1560	1493	1397	1538
K08160 MFS transporter, DHA1 family, multidrug/chloramphenicol efflux transport protein	622	621	594	718	480	499	495	477	932	926	976	919
K08161 MFS transporter, DHA1 family, multidrug resistance protein	1496	1515	1426	1555	1056	1081	1085	1050	1979	2209	2010	2150
K08162 MFS transporter, DHA1 family, multidrug resistance protein	710	730	675	713	497	494	499	473	990	979	1005	1069
K08163 MFS transporter, DHA1 family, multidrug resistance protein	435	512	470	473	140	168	136	162	994	1105	1029	1075
K08164 MFS transporter, DHA1 family, putative efflux transporter	1	0	0	0	0	0	0	0	0	0	0	0
K08167 MFS transporter, DHA2 family, multidrug resistance protein	3046	2960	2684	2761	1607	1615	1478	1523	1715	1812	1695	1680
K08168 MFS transporter, DHA2 family, metal-tetracycline-proton antiporter	2	2	4	2	30	21	24	24	31	39	25	18
K08169 MFS transporter, DHA2 family, multidrug resistance protein	2454	2420	2516	2664	3939	3998	4139	3952	4453	4349	4165	4232
K08170 MFS transporter, DHA2 family, multidrug resistance protein	524	482	476	485	126	124	119	116	959	1017	1034	977
K08172 MFS transporter, MHS family, shikimate and dehydroshikimate transport protein	682	727	641	691	496	551	474	515	1064	1095	1129	1157
K08173 MFS transporter, MHS family, metabolite:H+ symporter	281	225	245	260	349	406	361	339	58	73	78	70
K08177 MFS transporter, OFA family, oxalate/formate antiporter	837	818	808	837	644	684	556	555	1122	1207	1074	1205
K08178 MFS transporter, SHS family, lactate transporter	420	393	410	411	100	135	115	105	882	843	855	900
K08191 MFS transporter, ACS family, hexuronate transporter	4812	4750	4790	4884	5614	5871	5823	5646	5393	5181	5238	5342
K08194 MFS transporter, ACS family, D-galactonate transporter	4318	4192	3928	4193	2663	2682	2609	2775	3572	3683	3550	3564
K08195 MFS transporter, AAHS family, 4-hydroxybenzoate transporter	5676	5946	5437	5874	3160	3015	3076	3209	3449	3327	3229	3577
K08196 MFS transporter, AAHS family, cis,cis-muconate transporter	1895	1847	1806	1889	1084	1057	971	1074	682	555	535	610
K08217 MFS transporter, DHA3 family, macrolide efflux protein	544	618	580	635	383	414	411	416	607	638	619	669
K08219 MFS transporter, UMF2 family, putative MFS family transporter protein	646	617	628	673	433	459	417	431	862	960	878	882
K08221 MFS transporter, ACDE family, multidrug resistance protein	0	0	0	0	14	6	3	8	14	16	13	16
K08222 MFS transporter, YQGE family, putative transporter	835	948	959	875	2049	1975	2264	2199	1833	1780	1897	1733
K08223 MFS transporter, FSR family, fosmidomycin resistance protein	1553	1424	1517	1532	2943	2971	3050	2794	2851	2852	3026	2785
K08224 MFS transporter, YNFM family, putative membrane transport protein	2471	2357	2302	2268	1321	1349	1332	1426	1562	1522	1542	1520
K08225 MFS transporter, ENTS family, enterobactin (siderophore) exporter	736	682	659	691	490	578	489	497	852	897	874	959
K08227 MFS transporter, LPLT family, lysophospholipid transporter	783	727	670	736	467	522	469	444	1039	1058	899	1042
K08344 suppressor for copper-sensitivity B	719	703	660	725	200	209	205	200	1387	1391	1399	1476
K08363 mercuric ion transport protein	707	685	727	671	543	539	518	531	176	158	179	171
K08364 periplasmic mercuric ion binding protein	658	709	699	685	1024	1006	941	931	594	542	591	484
K08368 MFS transporter, putative metabolite transport protein	2728	2606	2384	2582	1693	1739	1652	1684	876	841	756	890
K08369 MFS transporter, putative metabolite:H+ symporter	2502	2455	2304	2381	1454	1571	1501	1554	3001	3285	3250	3410
K08715 inward rectifier potassium channel	1	0	0	0	0	0	0	0	0	0	0	0
K08717 urea transporter	960	938	892	915	545	516	703	775	325	268	273	288
K08721 outer membrane protein, multidrug efflux system	2188	2349	2128	2155	1251	1200	1137	1227	709	657	680	745
K08970 nickel/cobalt transporter (NicO) family protein	1990	2003	1784	2054	1725	1627	1665	1653	2097	2082	2084	2054
K08978 bacterial/archaeal transporter family protein	1151	1029	1120	1103	613	684	606	613	560	563	580	578
K08994 ion channel-forming bestrophin family protein	501	474	452	536	443	390	352	339	733	651	700	694
K09013 Fe-S cluster assembly ATP-binding protein	1350	1340	1424	1388	2165	2184	2178	2234	2334	2228	2247	2268
K09016 putative pyrimidine permease RutG	521	524	474	484	151	157	141	140	1082	1159	1007	1098
K09771 small multidrug resistance family-3 protein	736	649	695	657	425	380	363	398	399	464	439	501
K09774 lipopolysaccharide export system protein LptA	1153	1114	1195	1200	732	799	736	729	734	752	753	741
K09800 translocation and assembly module TamB	7231	7086	6797	7026	4087	4143	3906	4241	5067	5007	4828	5004
K09820 manganese/iron transport system ATP-binding protein	2	2	2	3	2	2	0	4	3	4	2	1
K10120 fructooligosaccharide transport system substrate-binding protein	434	417	431	461	25	18	25	10	423	398	408	418
K10121 fructooligosaccharide transport system permease protein	35	33	23	42	4	6	8	3	14	18	16	17
K10122 fructooligosaccharide transport system permease protein	20	19	21	17	4	5	1	5	15	9	16	9
K10124 carbohydrate-specific outer membrane porin	1033	1038	1012	1033	796	877	770	800	1536	1629	1494	1580
K10716 voltage-gated potassium channel	2068	2113	2008	2033	2630	2547	2537	2537	2502	2393	2517	2513
K10820 galactofuranose transport system ATP-binding protein [EC:7.5.2.9]	1190	1202	1120	1138	867	838	828	808	1484	1583	1334	1567
K10974 cytosine permease	2611	2544	2304	2460	1504	1425	1407	1384	1740	1765	1615	1718
K10975 allantoin permease	96	102	71	82	20	30	28	23	108	136	124	112
K11005 hemolysin A	10	1	12	9	3	5	2	2	123	128	138	146
K11102 proton glutamate symport protein	3256	3243	3174	3259	1928	1965	1833	1944	1870	1913	1796	1852
K11104 melibiose permease	675	667	642	729	209	227	196	195	1299	1279	1275	1357
K11105 cell volume regulation protein A	4807	4894	4807	4941	4982	5047	4974	5104	4248	4185	4234	4255
K11106 L-tartrate/succinate antiporter	798	782	741	754	566	600	508	486	1107	1199	1079	1092
K11189 phosphocarrier protein	4139	4082	3843	3940	2073	2265	2245	2483	1867	1826	1730	1855
K11201 PTS system, fructose-specific IIA-like component [EC:2.7.1.-]	0	0	0	0	8	10	1	5	0	2	6	2
K11202 PTS system, fructose-specific IIB-like component [EC:2.7.1.-]	104	98	130	129	35	40	50	37	115	153	160	154
K11203 PTS system, fructose-specific IIC-like component	437	427	418	414	120	146	100	100	372	398	326	372
K11249 cysteine/O-acetylserine efflux protein	316	371	348	362	267	311	255	244	451	502	498	485
K11250 leucine efflux protein	1332	1308	1304	1362	924	928	861	931	695	726	717	689
K11535 nucleoside transport protein	637	758	613	790	498	510	519	471	923	1043	967	1113
K11537 MFS transporter, NHS family, xanthosine permease	734	697	889	794	2586	2546	2624	2477	1910	1885	1846	1833
K11719 lipopolysaccharide export system protein LptC	1199	1257	1220	1217	684	782	751	741	773	832	743	797
K11732 phenylalanine-specific permease	822	820	685	911	607	703	631	576	1100	1085	1069	1265
K11733 lysine-specific permease	3317	3324	3147	3271	1952	2005	1976	1934	1810	1895	1841	1898
K11734 aromatic amino acid transport protein AroP	4928	4803	4785	4885	2879	2979	2781	2953	3294	3275	3292	3163
K11735 GABA permease	2451	2330	2318	2325	1182	1130	1112	1188	1768	1658	1569	1686
K11736 proline-specific permease ProY	726	752	738	778	543	581	556	564	1024	1015	1024	1054
K11737 D-serine/D-alanine/glycine transporter	812	843	839	833	662	626	582	598	1216	1215	1181	1258
K11738 L-asparagine permease	764	763	821	732	544	552	565	527	933	1122	1054	1044
K11741 quaternary ammonium compound-resistance protein SugE	2736	2873	2904	2805	2151	2151	1962	2153	1583	1496	1510	1516
K11742 spermidine export protein MdtI	583	564	551	497	321	362	318	346	351	341	340	341
K11743 spermidine export protein MdtJ	681	703	674	663	370	428	371	405	403	367	377	409
K11745 glutathione-regulated potassium-efflux system ancillary protein KefC	1066	1113	1000	1157	785	870	810	790	1430	1461	1365	1476
K11746 glutathione-regulated potassium-efflux system ancillary protein KefF	376	338	347	366	224	246	227	237	474	457	471	494
K11747 glutathione-regulated potassium-efflux system protein KefB	1131	1108	1079	1168	828	835	866	875	1501	1564	1485	1578
K11748 glutathione-regulated potassium-efflux system ancillary protein KefG	396	393	345	372	270	274	263	273	531	516	611	517
K11815 multidrug resistance protein EbrB	1	0	0	0	0	0	0	0	0	0	0	0
K11928 sodium/proline symporter	3526	3470	3322	3456	2168	2094	2041	2086	2142	2037	2141	2240
K11929 outer membrane pore protein E	669	672	611	706	480	559	503	453	934	858	767	872
K11934 outer membrane protein X	429	441	366	432	401	416	387	396	334	391	378	434
K11939 inner membrane transporter RhtA	1759	1708	1571	1692	979	970	912	971	1051	1009	1013	1112
K12041 solute carrier family 9 (sodium/hydrogen exchanger), member 6/7	0	0	2	0	0	0	0	0	0	0	0	0
K12299 MFS transporter, ACS family, probable galactarate transporter	802	754	692	874	560	584	610	517	1076	1146	1064	1121
K12516 putative surface-exposed virulence protein	8	9	8	3	11	6	13	7	119	135	130	104
K12537 membrane fusion protein, protease secretion system	3507	3194	3022	3187	1725	1696	1522	1898	1048	915	926	958
K12538 outer membrane protein, protease secretion system	3813	3736	3479	3506	2096	1862	1751	2108	1215	1067	1041	1146
K12542 membrane fusion protein, adhesin transport system	1898	1985	1790	1904	1516	1568	1388	1430	543	526	473	513
K12543 outer membrane protein, adhesin transport system	2372	2280	2195	2200	1685	1638	1471	1687	565	632	553	649
K12545 heme acquisition protein HasA	323	301	275	278	178	155	131	155	115	76	83	94
K12678 autotransporter family porin	110	95	119	101	62	56	58	62	217	236	214	242
K12684 serine protease autotransporter [EC:3.4.21.-]	12	7	19	19	26	39	14	25	251	314	302	304
K12685 subtilase-type serine protease [EC:3.4.21.-]	3560	3382	3283	3310	1978	1900	1729	2046	1219	1184	1140	1208

K12686 outer membrane lipase/esterase	3023	3036	2692	2824	1583	1613	1628	1830	960	854	853	918
K13283 ferrous-iron efflux pump FieF	571	533	556	591	362	379	365	389	748	716	731	771
K13629 D-serine transporter	849	836	843	911	613	673	618	571	1189	1184	1246	1257
K13888 membrane fusion protein, macrolide-specific efflux system	1819	1798	1699	1714	1124	1061	1021	1118	976	1055	922	960
K13929 malonate decarboxylase alpha subunit [EC:2.3.1.187]	4267	4201	3664	3937	2221	2263	2049	2294	2142	2155	2003	2192
K13931 malonate decarboxylase delta subunit	710	752	639	624	395	382	353	369	389	373	371	359
K13936 malonate transporter	481	525	456	540	389	415	397	405	665	655	594	648
K14052 putrescine importer	2357	2203	2308	2165	1119	1108	941	1029	1503	1436	1446	1586
K14053 outer membrane protein G	3	2	6	8	8	12	4	10	48	54	44	33
K14062 outer membrane protein N	367	332	357	370	90	111	76	79	725	772	728	779
K14347 solute carrier family 10 (sodium/bile acid cotransporter), member 7	2024	1896	1808	1979	1126	1148	1127	1186	1285	1177	1238	1183
K14392 sodium/pantothenate symporter	1100	1092	1007	1050	753	824	770	630	1480	1581	1473	1478
K14393 cation/acetate symporter	5003	4954	5039	5245	2927	2925	2803	3006	2501	2619	2594	2736
K14430 phosphate transporter	0	0	0	0	0	0	0	0	1	0	0	0
K14445 solute carrier family 13 (sodium-dependent dicarboxylate transporter), member 2/3/5	1047	1003	1145	1080	3168	3097	3173	3183	2600	2458	2565	2654
K14717 solute carrier family 39 (zinc transporter), member 11	13	7	11	6	8	14	9	12	12	8	7	9
K15108 solute carrier family 25 (mitochondrial thiamine pyrophosphate transporter), member 19	0	0	0	0	0	0	0	0	1	0	0	0
K15110 solute carrier family 25 (mitochondrial 2-oxodicarboxylate transporter), member 21	0	0	0	0	1	0	0	0	0	0	0	0
K15268 O-acetylserine/cysteine efflux transporter	1970	1899	1784	1844	1094	1159	1026	1139	1144	1139	1145	1101
K15269 probable blue pigment (indigoidine) exporter	338	371	350	373	77	92	91	65	639	748	750	750
K15270 S-adenosylmethionine uptake transporter	3	4	3	3	3	4	8	3	11	6	3	8
K15272 solute carrier family 35 (UDP-sugar transporter), member A1/2/3	0	0	1	0	0	0	0	0	0	0	0	0
K15280 solute carrier family 35, member C2	1	0	0	0	0	0	0	0	0	0	0	0
K15547 multidrug resistance protein MdtO	1209	1181	1105	1197	803	791	725	748	1715	1738	1620	1700
K15548 p-hydroxybenzoic acid efflux pump subunit AaeA	588	583	604	638	420	472	424	408	799	858	736	893
K15549 membrane fusion protein, multidrug efflux system	603	579	582	574	438	392	416	359	829	853	891	970
K15550 outer membrane protein, multidrug efflux system	845	824	754	723	483	506	463	470	1068	1080	1119	1095
K15721 pesticin/yersiniabactin receptor	675	770	727	803	208	210	203	192	1625	1637	1605	1605
K15725 outer membrane protein, cobalt-zinc-cadmium efflux system	3821	3508	3564	3627	2026	2041	1891	1983	1160	1073	964	1092
K15726 cobalt-zinc-cadmium resistance protein CzcA	10540	10333	10489	10435	12447	12757	12708	12826	8708	8118	8519	7979
K15727 membrane fusion protein, cobalt-zinc-cadmium efflux system	3308	3243	3487	3257	3506	3594	3318	3525	2360	2243	2316	2102
K15738 ABC transport system ATP-binding/permease protein	4465	4334	4355	4388	5181	5133	5240	5111	5337	5521	5472	5435
K15987 K(+)-stimulated pyrophosphate-energized sodium pump	2949	3148	3194	3059	5609	5644	5686	5608	4930	4979	5001	4975
K16052 MscS family membrane protein	41	39	44	41	30	23	27	19	133	142	124	155
K16053 miniconductance mechanosensitive channel	3802	3897	3754	3889	4336	4531	4417	4567	4739	4543	4543	4538
K16074 zinc transporter	3579	3712	3694	3537	1817	1849	1837	1964	2257	2114	2118	2162
K16075 magnesium transporter	0	0	0	0	0	0	0	0	0	0	2	0
K16076 outer membrane porin protein LC	5	2	2	8	8	13	8	16	86	126	108	100
K16077 sucrose porin	1426	1420	1377	1528	1026	984	959	967	1849	1936	1811	1921
K16079 outer membrane immunogenic protein	0	2	0	5	0	4	2	3	4	6	3	7
K16081 alginate production protein	2042	2184	1876	2048	1185	1066	1038	1187	642	636	518	663
K16087 hemoglobin/transferrin/lactoferrin receptor protein	11754	11828	10885	11545	6456	6321	6136	6781	5365	5210	5219	5470
K16088 outer-membrane receptor for ferric coprogen and ferric-rhodotorulic acid	11032	11074	10190	10380	6219	6107	5617	6412	3692	3173	3140	3454
K16089 outer membrane receptor for ferrienterochelin and colicins	9650	9626	9649	9262	18389	18612	18690	18428	13848	13639	13875	13395
K16090 catecholate siderophore receptor	7795	7608	7613	7529	4198	3861	3736	3973	3959	3960	3790	3974
K16091 Fe(3+) dicitrate transport protein	4296	4454	4305	4162	2450	2335	2314	2538	1569	1572	1447	1505
K16092 vitamin B12 transporter	3982	3985	3489	3573	2120	2109	2119	2368	2572	2604	2510	2561
K16139 glucuronide carrier protein	12	5	3	6	8	14	8	9	48	28	45	31
K16140 putative glucuronide porin	2	1	3	1	28	16	19	10	70	59	61	61
K16152 heme acquisition protein HasR	4277	4068	3673	3748	2095	2051	1948	2268	1231	1105	1182	1218
K16169 xanthine permease	104	88	94	89	23	34	29	16	105	90	94	106
K16209 lactose/raffinose/galactose permease	30	31	37	27	32	36	25	27	90	89	77	103
K16210 oligogalacturonide transporter	2025	1971	1977	2177	1797	1802	1558	1764	2202	2400	2296	2392
K16211 maltose/moltooligosaccharide transporter	154	201	199	193	267	276	245	280	20	44	34	33
K16234 histidine transporter	2961	2887	2786	3074	1468	1397	1407	1581	1741	1851	1706	1702
K16235 S-methylmethionine transporter	554	523	518	590	146	175	170	133	1091	1137	1082	1159
K16238 ethanolamine permease	2211	2222	2167	2177	1272	1349	1308	1319	1561	1571	1406	1577
K16248 probable glucitol transport protein GutA	139	129	111	126	189	165	164	155	460	495	454	437
K16261 yeast amino acid transporter	0	2	0	0	2	0	0	0	0	0	0	0
K16263 amino acid efflux transporter	781	746	740	817	535	603	491	572	987	1030	974	976
K16264 cobalt-zinc-cadmium efflux system protein	2777	2676	2739	2675	3202	3400	3319	3370	2347	2211	2272	2250
K16300 membrane fusion protein, epimerase transport system	5	2	0	3	0	0	0	0	0	0	0	0
K16301 deferrochelatase/peroxidase EfeB [EC:1.11.1.-]	753	689	732	740	509	564	549	529	983	1098	1025	1033
K16321 high-affinity gluconate transporter	2	0	1	1	8	21	8	13	11	16	3	7
K16322 low-affinity inorganic phosphate transporter	2740	2768	2554	2665	1778	1712	1721	1689	2081	2077	1966	2023
K16323 purine nucleoside transport protein	0	0	3	0	0	0	0	0	0	0	0	0
K16324 putative pseudouridine transporter	365	343	362	381	530	665	602	675	62	84	79	71
K16325 nucleoside permease	81	110	72	76	115	126	98	94	62	58	77	68
K16331 sulfate permease	3	0	0	0	0	0	0	0	0	0	0	0
K16345 xanthine permease XanP	7074	7194	6663	6988	6412	6577	6115	6552	5634	5409	5464	5535
K16346 xanthine permease XanQ	3	9	1	6	9	27	24	19	93	89	80	87
K16347 entericidin A	76	84	75	83	68	67	55	48	132	119	131	107
K16348 entericidin B	121	95	103	127	71	69	73	62	150	177	157	156
K16693 enterobacterial common antigen flippase	772	783	755	716	510	580	507	539	1031	1175	1156	1214
K16695 lipopolysaccharide exporter	261	287	239	274	454	431	432	372	67	54	61	52
K16788 niacin transporter	62	40	34	45	11	11	11	12	77	62	40	49
K16789 thiamine transporter	297	319	337	349	130	145	118	111	257	311	257	294
K16923 energy-coupling factor transport system substrate-specific component	268	293	325	314	89	77	113	91	253	304	242	273
K16924 energy-coupling factor transport system substrate-specific component	233	288	224	243	46	41	55	43	262	274	245	258
K16925 energy-coupling factor transport system substrate-specific component	45	39	50	44	4	10	8	7	30	72	45	24
K16926 energy-coupling factor transport system substrate-specific component	160	164	178	185	138	135	100	135	524	506	497	528
K16927 energy-coupling factor transport system substrate-specific component	52	53	65	69	50	36	58	60	169	150	156	125
K16928 energy-coupling factor transport system substrate-specific component	0	0	0	0	0	0	0	1	0	1	1	0
K16929 energy-coupling factor transport system substrate-specific component	0	0	0	0	0	1	2	1	1	0	2	1
K17713 outer membrane protein assembly factor BamB	2467	2626	2483	2550	1556	1606	1471	1444	1601	1665	1475	1637
K17938 peptide/bleomycin uptake transporter	703	706	671	704	548	559	525	521	922	1119	991	1038
K18141 membrane fusion protein, multidrug efflux system	470	504	400	507	163	130	172	136	976	966	938	990
K18142 multidrug efflux pump	1346	1276	1220	1315	443	455	439	424	2742	2595	2697	2740
K18214 MFS transporter, DHA3 family, tetracycline resistance protein	17	19	23	11	6	6	5	20	20	14	13	31
K18218 tetracycline resistance efflux pump	1	2	1	1	4	7	3	3	0	1	2	1
K18295 membrane fusion protein, multidrug efflux system	1844	1843	1576	1675	978	994	798	905	603	550	477	581
K18296 multidrug efflux pump	5157	5119	4830	4644	2762	2580	2597	2930	1517	1564	1515	1626
K18298 membrane fusion protein, multidrug efflux system	1727	1726	1573	1633	914	911	862	892	501	442	436	505
K18299 multidrug efflux pump	3503	3461	3456	3280	1730	1782	1641	1913	1095	989	934	984
K18300 outer membrane protein, multidrug efflux system	708	678	673	677	347	345	366	339	202	200	175	222
K18302 membrane fusion protein, multidrug efflux system	1510	1522	1426	1530	834	803	722	928	562	503	514	547
K18303 multidrug efflux pump	4838	4849	4860	4685	2706	2615	2506	2689	1626	1543	1534	1619
K18305 transmembrane protein	796	831	738	792	438	428	369	480	226	240	224	227
K18308 outer membrane protein, multidrug efflux system	2500	2302	1989	2175	1150	1119	1062	1269	758	616	607	677
K18480 cholesterol transport system auxiliary component	1666	1617	1651	1627	864	849	828	968	464	459	480	459
K18552 MFS transporter, DHA1 family, chloramphenicol/florfenicol resistance protein	2	3	0	0	0	4	0	0	4	4	1	3
K18567 MFS transporter, DHA1 family, purine base/nucleoside efflux pump	2720	2793	2529	2663	1706	1555	1700	1797	786	781	720	775
K18814 putative inorganic carbon (hco3(-)) transporter	73	58	50	61	24	16	25	26	64	104	82	82
K18898 membrane fusion protein, multidrug efflux system	3	4	3	3	9	9	25	10	61	97	99	76
K18899 multidrug efflux pump	13	16	5	15	36	49	46	36	194	192	177	197
K18901 membrane fusion protein, multidrug efflux system	0	0	0	0	1	0	0	0	2	2	3	7
K18902 multidrug efflux pump	5	2	9	3	4	6	6	6	4	3	2	7
K18903 outer membrane protein, multidrug efflux system	1	0	0	0	0	0	0	2	0	0	0	0
K18904 outer membrane protein, multidrug efflux system	0	0	0	0	3	7	3	3	4	2	3	3
K18924 paired small multidrug resistance pump	20	18	19	25	8	5	2	3	18	19	20	23
K18925 paired small multidrug resistance pump	12	12	11	13	0	2	1	0	13	10	8	18
K18926 MFS transporter, DHA2 family, lincomycin resistance protein	6	4	4	9	0	5	4	8	14	10	12	10
K18934 MFS transporter, DHA2 family, multidrug resistance protein	0	0	0	1	0	0	0	0	0	0	0	0
K18967 diguanylate cyclase [EC:2.7.7.65]	3	3	3	2	5	3	3	3	3	7	3	4
K18975 small multidrug resistance pump	0	0	0	0	0	0	0	0	0	0	1	0
K19058 mercuric ion transport protein	76	68	74	68	93	132	126	105	0	0	2	0
K19059 mercuric ion transport protein	428	399	460	426	321	320	352	338	75	113	77	117
K19231 fibronectin-binding autotransporter adhesin	70	94	88	86	16	5	13	13	146	180	155	177
K19416 modulator of FtsH protease	1165	1066	1023	1037	596	650	603	642	692	714	610	686
K19419 transmembrane protein EpsG	0	0	0	1	3	0	9	1	0	0	1	1
K19540 fructoselysine transporter	285	251	274	290	409	525	487	449	4	5	8	10

K19564 choline transport protein	0	1	0	0	0	0	0	0	0	0	0	0
K19577 MFS transporter, DHA1 family, inner membrane transport protein	1819	1927	1716	1769	1115	1031	988	1089	1335	1314	1284	1352
K19578 MFS transporter, DHA1 family, multidrug resistance protein	1	0	0	0	0	0	0	0	0	0	0	0
K19585 multidrug efflux pump	1737	1835	1736	1906	1176	1195	1225	1122	2360	2527	2457	2600
K19586 membrane fusion protein, multidrug efflux system	676	729	657	708	510	495	484	506	917	983	930	1019
K19593 outer membrane protein, multidrug efflux system	2324	2202	2142	2210	1140	1156	1117	1242	708	686	648	689
K19594 gold/copper resistance efflux pump	4810	4654	4384	4469	2411	2498	2313	2695	1578	1404	1378	1433
K19595 membrane fusion protein, gold/copper resistance efflux system	1475	1431	1351	1420	736	783	769	892	465	454	389	465
K20510 malonyl-S-ACP:biotin-protein carboxyltransferase subunit MadC [EC:2.1.3.10]	154	131	147	148	0	0	0	0	63	47	43	52
K20511 malonyl-S-ACP:biotin-protein carboxyltransferase subunit MadD [EC:2.1.3.10]	114	149	125	121	0	0	0	0	50	73	45	63
K20541 cellulose synthase operon protein B	3483	3388	3153	3575	2859	3129	2934	2973	3830	3870	3600	3979
K20543 cellulose synthase operon protein C	5956	5625	5580	5802	4591	4988	4641	4464	6641	6664	6308	6730
K20989 urea-proton symporter	0	0	0	0	1	0	0	0	0	0	0	0
K21133 multidrug efflux pump	4551	4252	4024	4131	2364	2151	2238	2248	1529	1381	1231	1287
K21134 multidrug efflux pump	5407	5214	4966	5043	2961	2924	2791	3106	1781	1635	1702	1726
K21135 membrane fusion protein, multidrug efflux system	1551	1572	1311	1445	816	777	682	811	458	443	414	401
K21136 membrane fusion protein, multidrug efflux system	2139	2025	2078	2036	1268	1158	1042	1212	701	628	591	689
K21137 membrane fusion protein, multidrug efflux system	1166	1275	1124	1192	640	615	518	628	404	367	368	363
K21393 TRAP-type transport system large permease protein	48	40	42	39	22	33	33	26	90	148	101	131
K21394 TRAP-type transport system small permease protein	42	37	45	44	5	8	22	5	71	49	79	75
K21395 TRAP-type transport system periplasmic protein	246	231	234	243	73	61	66	75	336	331	294	336
K21449 trimeric autotransporter adhesin	907	972	1031	956	528	548	587	565	725	732	663	705
K21572 starch-binding outer membrane protein, SusD/RagB family	80802	88133	95078	87060	284791	283905	291892	281873	205554	195781	207048	194994
K21573 TonB-dependent starch-binding outer membrane protein SusC	2916	3156	3624	3262	9615	9950	9757	9546	6681	6419	6768	6321
K21864 voltage-dependent calcium channel	0	0	0	0	0	0	0	0	0	0	2	0
K21989 calcium permeable stress-gated cation channel	1	2	0	0	0	0	0	0	0	0	0	0
K21993 formate transporter	142	132	141	134	55	33	61	43	100	169	97	145
K22044 moderate conductance mechanosensitive channel	3033	2840	2838	2889	1720	1925	1662	1825	2260	2308	2200	2408
K22051 miniconductance mechanosensitive channel	2190	2053	2020	2133	1534	1606	1534	1483	2747	2896	2845	2999
K22110 oligogalacturonate-specific porin family protein	572	591	551	600	543	523	520	549	608	598	626	602
K22112 putative amide transporter protein	724	735	771	671	396	395	349	393	218	222	185	238
K22736 vacuolar iron transporter family protein	1234	1274	1111	1197	842	731	772	874	435	333	309	402
K22737 erythrin-vacuolar iron transport family protein	0	0	2	0	2	0	0	0	2	2	3	1
K23016 MFS transporter, ACS family, L-galactonate transporter	307	336	311	324	383	480	468	401	0	2	3	2
K23025 H+-translocating diphosphatase [EC:7.1.3.1]	0	0	0	0	0	0	1	0	0	0	1	1
K23241 MFS transporter, DHA1 family, multidrug resistance protein	1213	1227	1111	1224	647	632	646	631	1739	1821	1798	1996
K23242 manganese efflux pump family protein	491	449	446	461	381	470	408	453	532	500	452	524
K23329 ATP-binding cassette, subfamily B, vacuolar membrane transporter HMT1/ACLQ	1	0	0	1	0	0	0	1	0	0	1	0
K23360 methylmalonyl-CoA decarboxylase subunit epsilon	1	0	2	1	0	0	0	0	0	0	2	0
K23508 galactofuranose transport system substrate-binding protein	819	758	685	770	599	611	543	522	849	840	908	975
K23509 galactofuranose transport system permease protein	1460	1424	1348	1472	1070	1162	1051	1059	1787	1782	1862	1960
K23511 riboflavin transport system permease protein	0	1	1	1	0	0	0	0	0	0	0	1
ko02044 Secretion system	441359	438686	422560	433004	275189	275811	264933	278999	294480	296581	285975	295358
K02221 YggT family protein	1270	1255	1331	1325	776	822	770	673	895	846	840	831
K02236 leader peptidase (prepilin peptidase) / N-methyltransferase [EC:3.4.23.43 2.1.1.-]	45	35	65	49	14	10	11	17	36	54	69	44
K02237 competence protein ComEA	862	857	907	877	470	479	425	548	606	706	740	590
K02238 competence protein ComEC	5582	5679	5610	5771	5749	5730	5808	5924	5824	5868	5581	5861
K02240 competence protein ComFA	61	58	66	65	14	19	30	16	55	106	73	101
K02241 competence protein ComFB	78	72	52	59	6	12	7	3	66	76	62	87
K02242 competence protein ComFC	26	40	40	30	7	10	12	8	36	43	36	36
K02243 competence protein ComGA	48	67	48	56	20	16	17	28	59	106	56	72
K02244 competence protein ComGB	42	53	49	45	14	18	11	12	67	75	75	62
K02245 competence protein ComGC	8	13	15	11	5	5	4	3	14	19	13	17
K02246 competence protein ComGD	19	30	16	19	7	8	7	6	16	16	32	31
K02248 competence protein ComGF	25	19	23	33	2	7	12	5	22	24	22	26
K02279 pilus assembly protein CpaB	1644	1726	1559	1580	883	899	866	931	508	491	546	523
K02280 pilus assembly protein CpaC	1963	2012	1871	2014	1151	1172	1191	1185	703	682	657	694
K02281 pilus assembly protein CpaD	9	12	4	9	17	16	6	23	19	27	29	28
K02282 pilus assembly protein CpaE	1922	1979	1836	1854	1066	1064	1063	1120	650	622	659	660
K02283 pilus assembly protein CpaF [EC:7.4.2.8]	2441	2351	2445	2288	1303	1331	1301	1374	1062	1009	1058	1100
K02450 general secretion pathway protein A	6	9	4	6	5	4	2	5	100	94	87	102
K02451 general secretion pathway protein B	211	187	154	186	45	37	56	58	404	450	412	404
K02504 protein transport protein HofB	747	643	688	817	515	541	480	490	1004	975	1050	944
K02505 protein transport protein HofC	839	758	674	756	534	524	565	559	998	993	1055	1121
K02507 protein transport protein HofQ	744	760	809	834	551	659	568	497	1068	1183	1058	1170
K02652 type IV pilus assembly protein PilB	3107	3329	3181	3372	1677	1692	1623	1628	1576	1719	1750	1548
K02653 type IV pilus assembly protein PilC	2609	2592	2709	2666	1346	1407	1221	1262	1426	1633	1494	1578
K02655 type IV pilus assembly protein PilE	664	723	689	706	358	343	371	380	225	255	222	211
K02656 type IV pilus assembly protein PilF	704	709	688	672	393	406	389	406	232	226	244	200
K02662 type IV pilus assembly protein PilM	80	72	96	65	47	48	49	37	84	101	90	93
K02663 type IV pilus assembly protein PilN	705	681	673	715	383	389	410	398	246	234	271	257
K02664 type IV pilus assembly protein PilO	1119	1078	1086	1136	565	620	536	600	366	380	399	399
K02665 type IV pilus assembly protein PilP	838	821	801	822	503	481	466	444	268	244	273	263
K02666 type IV pilus assembly protein PilQ	3243	3343	3424	3316	1798	1941	1768	1912	1191	1195	1087	1144
K02669 twitching motility protein PilT	4499	4540	4495	4507	2850	2944	2850	2954	2897	2960	2669	2906
K02670 twitching motility protein PilU	2022	2053	2007	1953	1153	1164	1074	1120	629	661	597	654
K02671 type IV pilus assembly protein PilV	804	812	878	844	469	513	482	495	257	305	286	280
K02672 type IV pilus assembly protein PilW	1246	1279	1272	1317	805	804	704	814	427	389	423	398
K02673 type IV pilus assembly protein PilX	869	893	962	919	544	588	511	534	274	295	296	262
K02674 type IV pilus assembly protein PilY1	5374	5554	5888	5865	3216	3352	3295	3347	1958	1851	1810	1761
K02676 type IV pilus assembly protein PilZ	457	442	394	459	250	262	239	255	142	118	148	146
K02679 prepilin peptidase dependent protein A	415	336	373	397	236	222	231	239	528	516	542	576
K02680 prepilin peptidase dependent protein B	303	296	277	309	206	205	205	202	443	396	431	460
K02681 prepilin peptidase dependent protein C	180	185	157	172	128	125	136	118	278	242	251	271
K02682 prepilin peptidase dependent protein D	238	219	214	235	185	192	145	167	277	329	298	304
K03220 type III secretion protein D	1960	2043	1946	1958	1006	1043	1019	1063	559	554	595	527
K04049 type III secretion protein B	480	482	417	417	246	220	236	304	141	125	105	141
K04050 type III secretion protein E	269	240	238	213	137	122	127	120	77	67	69	77
K04051 type III secretion protein G	530	498	511	548	292	278	239	291	184	163	139	163
K04052 type III secretion protein H	634	573	549	568	298	340	314	277	214	153	158	181
K04053 type III secretion protein I	480	427	474	454	257	226	253	274	155	156	126	133
K04054 type III secretion protein K	833	731	695	725	458	385	379	455	228	203	229	233
K04060 type III secretion protein Y	366	442	382	433	213	218	225	236	97	113	108	103
K04061 flagellar biosynthesis protein	668	709	647	675	307	291	308	347	340	340	304	361
K04337 curli production assembly/transport component CsgE	72	91	65	77	99	113	103	112	19	20	13	31
K04338 curli production assembly/transport component CsgF	85	65	67	95	124	120	130	114	21	20	21	12
K06214 curli production assembly/transport component CsgG	145	143	156	156	256	256	279	229	34	58	48	48
K07332 archaeal flagellar protein FlaI	0	0	2	0	0	3	0	0	13	6	4	15
K07333 archaeal flagellar protein FlaJ	0	2	2	0	2	4	0	4	2	13	5	18
K07348 minor fimbrial subunit	351	376	313	359	229	241	253	208	537	552	521	514
K07349 minor fimbrial subunit	209	216	205	217	60	60	66	43	451	497	446	452
K07350 minor fimbrial subunit	594	595	575	622	475	575	463	427	815	808	783	810
K08084 type IV fimbrial biogenesis protein FimT	749	724	766	811	435	453	407	421	211	267	241	244
K08085 type IV fimbrial biogenesis protein FimU	1037	1111	1084	1065	546	557	547	557	338	326	285	328
K11894 type VI secretion system protein ImpI	1788	1779	1793	1822	1072	979	1050	1038	521	517	508	469
K11896 type VI secretion system protein ImpG	7259	6984	6620	6908	4112	4121	4073	4382	4259	3990	3906	4111
K11897 type VI secretion system protein ImpF	1013	990	920	877	612	609	652	696	283	246	257	270
K11898 type VI secretion system protein ImpE	1214	1170	1168	1164	820	783	767	810	330	290	296	345
K11905 type VI secretion system protein	884	939	876	899	492	511	549	536	632	568	569	581
K11909 type VI secretion system protein VasI	57	50	53	73	65	83	90	71	4	4	3	1
K11910 type VI secretion system protein VasJ	3055	3169	3021	2997	1900	1825	1796	1820	1686	1766	1787	1765
K11911 type VI secretion system protein VasL	832	864	771	887	593	589	595	580	1001	1023	1088	1022
K11916 serine/threonine-protein kinase Stk1 [EC:2.7.11.-]	1429	1315	1285	1352	660	724	653	727	430	418	450	378
K11918 type VI secretion system protein	575	636	604	576	309	301	315	360	195	161	172	193
K11919 type VI secretion system lysozyme-related protein	618	575	526	529	279	297	288	357	193	160	172	182
K12055 chromosome partitioning related protein ParA	1450	1464	1542	1390	1276	1288	1293	1221	589	555	576	531
K12056 conjugal transfer mating pair stabilization protein TraG	1460	1455	1528	1536	543	590	616	525	8713	9573	8826	8891
K12057 conjugal transfer pilus assembly protein TraF	386	429	459	472	141	176	184	149	2633	2918	2545	2720
K12058 conjugal transfer mating pair stabilization protein TraN	603	660	710	685	267	254	281	252	4068	4484	3969	4072
K12059 conjugal transfer pilus assembly protein TrbC	174	206	223	226	46	86	90	73	1236	1342	1316	1423
K12060 conjugal transfer pilus assembly protein TraU	532	533	484	536	192	217	211	188	3139	3322	3187	3294

K12061 conjugal transfer pilus assembly protein TraW	344	329	387	379	139	169	151	110	2208	2538	2168	2183
K12062 conjugal transfer pilin signal peptidase TrbI	230	192	231	229	82	76	81	83	1263	1423	1173	1319
K12063 conjugal transfer ATP-binding protein TraC	1448	1289	1445	1470	629	563	541	526	8524	9390	8573	9016
K12064 conjugal transfer pilus assembly protein TraV	80	89	111	106	50	44	37	30	479	547	529	584
K12065 conjugal transfer pilus assembly protein TraB	658	631	660	682	232	297	252	235	4093	4190	3884	4169
K12066 conjugal transfer pilus assembly protein TraK	245	267	269	289	98	120	110	114	1486	1606	1461	1680
K12067 conjugal transfer pilus assembly protein TraE	347	302	294	294	108	144	100	115	1776	1856	1764	1735
K12068 conjugal transfer pilus assembly protein TraL	187	153	161	169	50	62	72	64	862	985	961	957
K12069 conjugal transfer pilus assembly protein TraA	2	1	2	3	0	0	0	0	0	0	1	0
K12070 conjugal transfer pilus assembly protein TraI	0	2	0	0	0	0	0	0	0	0	2	1
K12071 conjugal transfer pilus assembly protein TraD	31	30	12	21	42	54	54	54	48	37	44	38
K12072 conjugative transfer pilus assembly protein TraH	829	766	803	838	369	393	383	365	4616	4805	4626	4823
K12203 defect in organelle trafficking protein DotB [EC:7.2.4.8]	0	1	0	1	0	0	0	0	2	0	1	1
K12204 defect in organelle trafficking protein DotC	0	0	0	0	0	0	0	0	1	0	0	1
K12209 intracellular multiplication protein IcmE	0	0	0	0	0	1	0	0	0	0	0	0
K12213 intracellular multiplication protein IcmK	0	0	0	0	0	2	0	0	0	0	0	0
K12217 intracellular multiplication protein IcmO [EC:7.2.4.8]	4	3	9	4	3	1	2	2	17	10	10	20
K12218 intracellular multiplication protein IcmP	0	0	0	0	0	0	0	0	0	1	0	0
K12268 accessory secretory protein Asp1	0	0	1	0	0	2	0	0	0	0	1	0
K12269 accessory secretory protein Asp2	0	1	0	1	3	2	4	1	2	0	1	0
K12270 accessory secretory protein Asp3	0	0	0	0	0	0	0	1	0	0	1	0
K12278 MSHA biogenesis protein MshG	0	1	0	1	0	0	0	0	0	2	0	1
K12288 pilus assembly protein HofM	482	560	490	540	379	387	387	409	700	770	626	692
K12289 pilus assembly protein HofN	399	326	339	363	290	297	236	237	465	460	458	485
K12290 pilus assembly protein HofO	267	252	213	263	141	200	188	179	318	326	309	385
K12291 pilus assembly protein HofP	183	234	186	217	131	146	138	169	332	326	292	291
K12510 tight adherence protein B	1546	1547	1517	1612	747	739	741	763	702	601	717	752
K12511 tight adherence protein C	1387	1424	1258	1294	750	740	741	842	481	401	449	408
K12517 major pilin subunit PapA	0	1	0	1	2	0	0	3	0	4	1	1
K12518 outer membrane usher protein PapC	7	8	9	11	41	43	40	44	127	120	115	170
K12519 chaperone protein PapD	2	4	6	4	13	10	11	6	39	35	40	44
K12520 minor pilin subunit PapE	0	0	0	0	0	0	0	0	1	2	1	0
K12521 minor pilin subunit PapF	0	0	1	0	1	0	0	0	1	0	0	0
K12522 adhesin PapG	0	0	2	1	1	1	1	0	4	3	2	0
K12523 minor pilin subunit PapK	2	0	0	0	1	0	3	0	2	0	1	1
K21964 Mat/Ecp fimbriae major subunit	307	285	281	296	74	55	81	67	482	459	475	488
K21965 Mat/Ecp fimbriae periplasmic chaperone	282	290	274	322	79	79	77	76	530	493	552	604
K21966 Mat/Ecp fimbriae outer membrane usher protein	1241	1337	1164	1303	288	292	308	273	2025	2231	2137	2207
K21967 Mat/Ecp fimbriae adhesin	818	797	726	816	180	216	184	210	1462	1379	1346	1484
K21968 Mat/Ecp fimbriae periplasmic chaperone	356	362	305	389	78	93	98	97	596	584	540	614
K22487 type III secretion system protein	0	3	2	0	3	0	0	0	0	0	2	0
K22488 type III secretion system protein	0	0	0	0	16	17	1	9	5	0	2	0
K22504 type III secretion system outer membrane ring protein	0	0	0	0	0	0	0	0	0	0	2	0
K22505 type III secretion system inner membrane ring protein	0	0	0	0	3	0	2	4	0	0	0	1
K22506 type III secretion system ATPase [EC:7.4.2.8]	0	0	1	0	0	0	0	0	0	0	0	0
K22507 type III secretion system export apparatus protein	1	0	0	0	5	6	0	8	0	0	0	0
K22508 type III secretion system export apparatus protein	0	0	0	0	0	3	5	4	0	2	1	0
K22509 type III secretion system export apparatus protein	0	0	0	0	7	5	3	9	0	0	2	1
K22510 type III secretion system export apparatus switch protein	0	0	0	0	2	6	7	3	1	0	0	2
K22514 type III secretion system protein	0	0	0	0	1	0	0	0	0	0	1	0
ko02042 Bacterial toxins	23408	23457	23290	23428	22753	22816	23118	23209	16945	16365	16465	16328
K03699 putative hemolysin	1844	1908	1958	1829	2836	2775	2919	2865	2850	2870	2960	2878
K11008 cytotoxic necrotizing factor 1	8	2	5	7	11	3	7	18	142	200	165	172
K11009 murine toxin	68	62	60	68	84	115	110	106	0	0	0	0
K11020 exotoxin A [EC:2.4.2.-]	449	411	367	392	219	191	215	234	125	143	117	120
K11021 insecticidal toxin complex protein TccC	0	0	0	1	0	0	0	0	0	0	0	1
K11045 cAMP factor	0	1	0	0	1	0	0	0	0	0	0	0
K11068 hemolysin III	1605	1595	1702	1604	2012	2058	2089	1950	2044	1928	2080	1953
K11139 hemolysin E	3	0	0	3	14	6	3	6	12	15	16	17
K16636 exoenzyme T	1645	1636	1654	1585	931	971	883	906	611	563	504	547
K16637 adenylate cyclase ExoY	883	822	758	784	407	406	379	433	219	241	210	228
K16638 exoenzyme U	2600	2537	2781	2767	1489	1465	1541	1463	894	846	823	783
ko02022 Two-component system	238634	234562	229833	235177	148390	149747	146323	150491	171306	173655	169638	173985
K02475 two-component system, CitB family, response regulator	28	28	22	31	3	5	7	8	39	32	22	28
K02477 two-component system, LytTR family, response regulator	1946	1959	2124	2112	3918	3979	3964	3940	4079	3921	4098	3833
K02479 two-component system, NarL family, response regulator	1	0	0	0	0	0	0	0	0	0	0	0
K02481 two-component system, NtrC family, response regulator	1696	1764	1813	1731	5340	5384	5591	5339	3940	3617	3923	3697
K02483 two-component system, OmpR family, response regulator	417	424	431	435	212	230	235	227	964	1038	908	931
K02485 two-component system, response regulator	526	519	580	550	424	451	380	410	772	860	767	832
K07183 two-component system, response regulator / RNA-binding antiterminator	567	590	501	586	306	305	294	262	190	167	156	135
K07814 putative two-component system response regulator	2584	2576	2470	2532	1460	1332	1260	1385	1243	1283	1250	1303
K21603 two-component system, OmpR family, flagellar system response regulator FtcR	0	2	1	0	0	0	0	3	0	0	3	0
K22010 two-component system, response regulator PdtaR	375	311	358	338	87	118	88	85	281	334	316	332
K22067 two-component system, oxyanion-binding sensor	1789	1729	1805	1741	921	952	885	1069	575	459	509	470
ko02035 Bacterial motility proteins	232520	230031	224589	229169	138908	141101	132665	138650	104936	105096	102415	105206
K02385 flagellar protein FlbD	115	100	128	101	29	24	31	26	76	84	77	90
K02395 flagellar protein FlgJ	2085	2045	2044	2046	1262	1285	1280	1276	695	616	636	608
K02404 flagellar biosynthesis protein FlhF	1539	1606	1463	1459	811	808	687	809	511	418	442	452
K02415 flagellar FliL protein	995	949	995	905	620	617	769	754	295	333	323	332
K02675 type IV pilus assembly protein PilY2	429	515	555	548	311	306	293	286	157	171	165	173
K03516 flagellar protein FlhE	81	81	74	93	145	105	113	121	20	22	25	17
K04562 flagellar biosynthesis protein FlhG	1170	1152	1156	1169	628	668	604	655	386	325	364	406
K06595 heam-based aerotactic trancducer	2	0	6	3	0	0	0	0	0	1	0	0
K06601 flagellar protein FlbT	0	0	1	0	0	0	0	0	0	0	2	1
K06602 flagellar protein FlaF	0	0	0	1	0	0	0	0	0	0	0	0
K06603 flagellar protein FlaG	524	565	530	558	286	299	316	312	176	154	179	165
K07351 fimbrial protein	242	236	240	264	103	91	98	50	549	588	532	606
K07352 type 1 fimbrial protein	0	4	7	0	2	0	0	0	0	0	3	0
K07353 fimbrial chaperone protein	0	0	0	0	9	4	8	7	0	0	3	1
K07354 outer membrane usher protein	0	0	0	0	16	11	21	16	0	12	6	5
K07355 fimbrial-like protein	0	0	0	0	5	8	1	2	0	0	3	2
K07356 fimbrial protein	0	0	0	0	14	16	8	5	24	15	14	7
K10564 chemotaxis protein MotC	1	2	0	1	3	0	0	0	5	0	3	5
K10565 chemotaxis protein MotD	0	1	0	0	3	0	4	0	0	6	1	5
K13626 flagellar assembly factor FliW	288	251	253	279	68	75	94	89	271	317	280	288
K18475 lysine-N-methylase [EC:2.1.1.-]	169	167	198	191	76	89	104	88	193	234	219	223
ko04812 Cytoskeleton proteins	42353	42608	42643	42945	43096	42923	42383	42182	48325	49299	48277	48294
K06990 MEMO1 family protein	7	3	4	7	0	2	3	0	28	24	16	27
K10401 kinesin family member 18/19	0	0	0	0	2	0	0	0	0	0	0	1
K17292 tubulin-specific chaperone A	0	0	0	0	0	0	0	0	1	0	0	0
K18640 plasmid segregation protein ParM	1591	1612	1576	1704	900	971	832	877	4905	5481	5150	5121
K19755 radial spoke head protein 1	20	23	43	31	100	99	0	0	73	54	88	68
ko04147 Exosome	228110	227877	228494	227107	244287	245232	242597	244941	227812	225518	225574	224253
K01482 dimethylargininase [EC:3.5.3.18]	72	69	75	84	44	33	47	41	39	34	25	39
K02503 histidine triad (HIT) family protein	2078	2105	2006	2175	1888	1911	1912	1875	1717	1648	1665	1624
K03098 apolipoprotein D and lipocalin family protein	1350	1343	1257	1291	739	686	719	714	880	819	889	861
K03522 electron transfer flavoprotein alpha subunit	3999	4137	3983	3921	4340	4137	4150	4411	3167	2835	3044	3117
K17086 transmembrane 9 superfamily member 2/4	0	0	0	0	0	0	0	0	0	0	0	1
K17255 Rab GDP dissociation inhibitor	0	1	0	0	0	0	0	0	0	0	0	0
K18222 arylsulfatase E [EC:3.1.6.-]	0	0	0	0	0	0	0	0	0	0	0	1
K18258 thiomorpholine-carboxylate dehydrogenase [EC:1.5.1.25]	0	0	0	1	0	1	0	2	0	0	1	1
K20011 peroxiredoxin 3 [EC:1.11.1.15]	1	0	0	0	0	0	0	0	0	0	0	0
ko02048 Prokaryotic defense system	80503	80809	82773	82175	83336	84742	84850	83760	85210	87396	85610	86848
K00571 site-specific DNA-methyltransferase (adenine-specific) [EC:2.1.1.72]	969	927	1063	970	1725	1765	1852	1666	2782	2965	2811	2818
K01153 type I restriction enzyme, R subunit [EC:3.1.21.3]	6070	6071	6312	6229	4517	4612	4472	4430	3152	3438	3379	3438
K01154 type I restriction enzyme, S subunit [EC:3.1.21.3]	3298	3282	3607	3402	4432	4464	4710	4430	2626	2662	2779	2731
K01155 type II restriction enzyme [EC:3.1.21.4]	132	142	166	143	167	174	183	169	21	29	21	22
K01156 type III restriction enzyme [EC:3.1.21.5]	130	140	160	173	24	37	28	37	292	405	358	334
K03427 type I restriction enzyme M protein [EC:2.1.1.72]	4622	4539	4928	4761	4681	4879	4800	4757	3089	2895	2892	3091
K03970 phage shock protein B	115	116	105	114	76	79	103	77	193	188	175	168
K05839 haemolysin expression modulating protein	305	289	319	347	168	194	161	163	934	1020	982	1052

K06218 mRNA interferase RelE/StbE	131	143	134	106	78	104	65	90	24	25	33	38
K07012 CRISPR-associated endonuclease/helicase Cas3 [EC:3.1.-.- 3.6.4.-]	3000	3144	3248	3259	1391	1428	1465	1285	3308	3491	3414	3467
K07016 CRISPR-associated protein Csm1	0	0	1	1	2	0	1	4	6	7	8	5
K07060 endoribonuclease Nob1 [EC:3.1.-.-]	0	0	2	3	0	0	0	0	2	4	2	0
K07062 toxin FitB [EC:3.1.-.-]	13	18	23	15	21	16	16	31	20	36	21	28
K07171 mRNA interferase MazF [EC:3.1.-.-]	665	570	576	614	303	261	225	254	608	801	666	700
K07172 antitoxin MazE	2	1	1	0	8	10	9	6	10	8	19	17
K07316 adenine-specific DNA-methyltransferase [EC:2.1.1.72]	85	68	86	81	53	60	50	35	218	300	237	218
K07317 adenine-specific DNA-methyltransferase [EC:2.1.1.72]	5	3	4	6	8	12	2	5	7	3	6	10
K07318 adenine-specific DNA-methyltransferase [EC:2.1.1.72]	365	392	406	399	69	90	56	64	164	251	243	262
K07319 adenine-specific DNA-methyltransferase [EC:2.1.1.72]	642	623	586	548	446	502	480	476	2052	2373	2144	2259
K07334 toxin HigB-1	498	497	479	488	614	538	561	596	373	421	407	416
K07339 mRNA interferase HicA [EC:3.1.-.-]	4	7	5	5	8	5	10	4	9	28	20	20
K07341 death on curing protein	192	217	214	221	78	64	77	71	357	375	410	421
K07448 restriction system protein	416	449	465	435	286	325	285	281	198	248	213	219
K07451 5-methylcytosine-specific restriction enzyme A [EC:3.1.21.-]	592	575	617	648	969	1022	1004	915	332	364	342	331
K07452 5-methylcytosine-specific restriction enzyme B [EC:3.1.21.-]	976	882	931	880	1191	1248	1286	1183	666	679	671	713
K07464 CRISPR-associated exonuclease Cas4 [EC:3.1.12.1]	237	181	231	237	216	204	227	207	276	297	285	336
K07473 DNA-damage-inducible protein J	405	428	389	425	200	188	152	181	855	935	932	869
K07746 antitoxin ParD1/3/4	563	526	596	570	524	551	557	608	202	197	216	192
K09002 CRISPR-associated protein Csm3	1	2	3	1	2	0	2	5	10	3	7	14
K09127 CRISPR-associated protein Cmr3	2	0	0	1	0	0	0	0	0	0	0	0
K09159 antitoxin CptB	614	572	533	551	336	362	346	355	384	365	326	367
K09951 CRISPR-associated protein Cas2	239	234	265	259	170	164	174	175	351	378	387	437
K09952 CRISPR-associated endonuclease Csn1 [EC:3.1.-.-]	255	268	262	308	85	113	80	89	211	285	251	215
K12148 biofilm regulator BssS	220	168	174	199	154	184	163	189	208	180	180	219
K12151 multiple stress resistance protein BhsA	161	148	171	172	146	162	146	149	169	173	168	192
K13650 MqsR-controlled colanic acid and biofilm protein A	93	109	85	90	25	31	34	36	215	214	241	211
K13651 motility quorum-sensing regulator / GCU-specific mRNA interferase toxin	2	4	2	1	0	0	2	0	0	0	0	1
K16214 UDP-N-acetylglucosamine kinase [EC:2.7.1.176]	47	34	49	29	24	32	24	23	30	29	30	28
K18829 antitoxin VapB	471	559	551	583	362	446	398	403	159	169	140	144
K18837 cytoskeleton-binding toxin CbtA and related proteins	8	9	12	7	8	8	3	12	89	81	87	101
K18838 cytoskeleton bundling-enhancing protein CbeA and related proteins	4	9	1	2	3	10	5	5	73	65	74	66
K18839 toxin GhoT	0	1	1	0	0	2	0	2	2	10	13	3
K18840 antitoxin GhoS	0	1	1	0	5	1	4	7	26	7	13	12
K18841 mRNA interferase ChpB [EC:3.1.-.-]	67	64	62	62	78	97	81	86	4	0	0	1
K18842 antitoxin ChpS	0	0	0	0	0	0	4	4	2	0	0	2
K18843 antitoxin HicB	8	11	9	13	24	22	18	17	34	40	51	44
K18862 small toxic polypeptide LdrA/B/C/D	5	0	1	1	17	14	17	16	20	23	38	27
K18919 protein HokC/D	3	2	2	1	4	5	3	3	21	26	16	17
K18920 protein HokA	82	100	75	110	24	40	43	24	166	227	170	179
K18921 protein HokB	1	0	4	0	3	4	3	7	16	20	17	23
K18922 protein HokE	101	84	82	93	35	28	28	23	195	206	185	204
K18923 antitoxin StbD	87	95	83	90	137	155	120	109	21	10	12	15
K19046 CRISPR system Cascade subunit CasB	212	250	213	217	74	79	77	80	524	464	508	485
K19048 toxic protein SymE	328	325	320	318	230	254	267	240	331	392	379	364
K19075 CRISPR-associated protein Cst2	0	4	3	0	4	0	0	0	0	0	5	0
K19076 CRISPR-associated protein Cmr2	1	2	0	1	0	0	0	0	0	0	0	0
K19088 CRISPR-associated protein Cst1	2	0	1	0	0	0	1	0	0	0	0	0
K19090 CRISPR-associated protein Cas5t	1	3	2	0	1	0	0	0	0	0	0	0
K19091 CRISPR-associated endoribonuclease Cas6 [EC:3.1.-.-]	4	6	9	11	13	5	8	9	74	72	108	112
K19092 toxin ParE1/3/4	884	862	808	875	697	810	677	800	295	288	286	292
K19114 CRISPR-associated protein Csh1	3	7	2	3	7	3	6	7	62	47	50	63
K19115 CRISPR-associated protein Csh2	4	11	18	7	9	15	13	15	101	122	82	109
K19116 CRISPR-associated protein Cas5h	9	9	8	8	14	5	13	6	64	94	77	66
K19117 CRISPR-associated protein Csd1	418	477	486	464	494	488	492	504	395	467	404	413
K19118 CRISPR-associated protein Csd2	626	604	657	607	266	301	298	268	380	482	420	497
K19119 CRISPR-associated protein Cas5d	442	428	396	457	213	182	182	197	318	367	299	320
K19123 CRISPR system Cascade subunit CasA	542	554	562	581	168	186	221	188	1130	1049	1098	1288
K19124 CRISPR system Cascade subunit CasC	390	358	405	389	130	142	150	147	763	836	833	844
K19125 CRISPR system Cascade subunit CasD	279	300	285	252	121	74	87	75	455	568	479	504
K19126 CRISPR system Cascade subunit CasE	241	236	233	280	80	84	93	68	494	544	547	580
K19127 CRISPR-associated protein Csy1	1283	1323	1287	1318	720	671	708	714	469	496	438	445
K19128 CRISPR-associated protein Csy2	593	554	576	522	287	330	312	312	195	216	198	217
K19129 CRISPR-associated protein Csy3	1103	1008	1021	1060	568	535	568	602	365	346	369	341
K19130 CRISPR-associated endonuclease Csy4 [EC:3.1.-.-]	763	774	734	770	422	424	378	432	243	263	283	224
K19134 CRISPR-associated protein Csx10	68	54	60	47	2	1	0	3	0	0	0	0
K19137 CRISPR-associated protein Csn2	38	35	28	39	6	13	10	12	32	46	33	32
K19138 CRISPR-associated protein Csm2	0	0	1	1	0	0	0	3	0	0	2	1
K19139 CRISPR-associated protein Csm4	0	2	0	0	0	0	0	0	2	3	3	9
K19140 CRISPR-associated protein Csm5	2	0	0	1	0	0	2	0	1	0	0	2
K19142 CRISPR-associated protein Cmr6	0	0	2	2	0	0	0	0	0	1	0	0
K19145 CRISPR-associated protein Csx16	1	0	0	0	0	0	0	1	0	0	0	0
K19147 5-methylcytosine-specific restriction enzyme subunit McrC	576	628	600	619	724	762	824	804	264	225	229	287
K19148 small toxic protein TisB	31	25	22	41	12	6	13	9	52	44	64	55
K19154 small toxic protein ShoB	1	1	1	0	0	0	0	2	4	3	5	5
K19155 toxin YhaV [EC:3.1.-.-]	0	0	3	0	6	3	2	4	21	19	19	28
K19156 antitoxin PrlF	0	0	2	0	1	4	2	2	11	9	19	20
K19157 mRNA interferase YafQ [EC:3.1.-.-]	440	424	436	460	184	244	199	189	864	849	854	886
K19158 toxin YoeB [EC:3.1.-.-]	53	62	69	70	30	38	42	50	29	30	34	21
K19159 antitoxin YefM	2	2	5	7	3	5	3	2	21	21	13	14
K19162 hha toxicity modulator TomB	214	249	218	249	179	164	183	182	339	322	335	307
K19163 toxin CcdB	191	178	156	192	148	174	133	141	202	188	188	194
K19164 antitoxin CcdA	68	59	54	68	89	95	90	82	16	23	24	15
K19165 antitoxin Phd	113	123	128	130	41	36	42	33	250	278	278	295
K19166 mRNA interferase HigB [EC:3.1.-.-]	376	448	375	419	694	710	722	703	1137	1240	1206	1132
K19167 protein AbiQ	2	1	0	3	3	4	12	8	0	0	0	0
K19168 toxin CptA	899	967	915	861	532	509	500	557	524	599	522	544
K19169 DNA sulfur modification protein DndB	8	7	5	6	18	28	33	24	26	28	18	20
K19170 DNA sulfur modification protein DndC	6	9	6	10	7	1	10	9	32	28	36	26
K19171 DNA sulfur modification protein DndD	55	52	69	62	8	3	8	3	154	158	168	173
K19172 DNA sulfur modification protein DndE	3	10	10	12	1	0	1	2	8	2	6	8
K19175 DNA phosphorothioation-dependent restriction protein DptH	3	5	3	6	1	2	2	3	0	0	0	0
K19334 biofilm protein TabA	460	487	457	493	246	267	263	266	579	544	547	539
K19686 ribonuclease VapC [EC:3.1.-.-]	0	0	0	0	0	0	0	0	0	2	0	1
K19687 antitoxin VapB	0	0	0	0	0	0	0	0	1	0	0	1
K19688 biofilm regulator BssR	207	231	236	231	166	186	153	163	376	349	333	310
K21487 toxin YobL [EC:3.1.-.-]	0	8	9	2	10	0	0	0	0	0	6	0
K21488 antitoxin YobK	98	79	99	98	363	309	381	373	267	227	279	261
K21493 toxin YxiD [EC:3.1.-.-]	1	0	0	5	0	0	0	5	0	0	0	0
K21495 antitoxin FitA	1	0	0	0	0	0	0	0	0	1	1	1
K21498 antitoxin HigA-1	717	762	780	726	776	737	707	771	517	510	503	507
ko04030 G protein-coupled receptors	0	2	0	0	0	2	0	0	1	0	0	0
ko04031 GTP-binding proteins	0	0	1	0	1	0	0	1	0	0	0	1
ko04090 CD molecules	3123	3443	3555	3351	10264	10371	10615	10264	7421	7170	7332	6944
ko00536 Glycosaminoglycan binding proteins	12786	12488	11519	12000	6736	6628	4019	4555	4275	3915	3848	4035
ko00537 Glycosylphosphatidylinositol (GPI)-anchored proteins	11270	11437	11492	11569	11459	11673	11658	11731	11295	11507	11411	11418
ko01504 Antimicrobial resistance genes	137682	137214	134550	136095	112296	112557	108757	112156	98321	96351	95857	97439
K00638 chloramphenicol O-acetyltransferase type B [EC:2.3.1.28]	906	991	926	948	535	503	464	543	334	271	271	300
K00662 aminoglycoside 3-N-acetyltransferase [EC:2.3.1.81]	164	149	137	154	12	16	14	10	162	156	193	164
K00663 aminoglycoside 6'-N-acetyltransferase [EC:2.3.1.82]	0	2	3	1	0	0	0	0	4	13	7	5
K00897 kanamycin kinase [EC:2.7.1.95]	3	3	5	7	2	2	2	2	0	2	2	0
K00984 streptomycin 3-adenylyltransferase [EC:2.7.7.47]	38	48	41	39	62	46	56	57	108	83	111	104
K04343 streptomycin 6-kinase [EC:2.7.1.72]	1036	978	994	1000	564	558	512	485	298	282	325	319
K05593 aminoglycoside 6-adenylyltransferase [EC:2.7.7.-]	1101	1093	1223	1150	1838	1733	1781	1774	2144	1993	2083	2078
K06880 erythromycin esterase [EC:3.1.1.-]	445	430	472	493	25	31	23	32	389	445	428	436
K06979 macrolide phosphotransferase	0	0	0	0	2	2	0	2	4	0	3	4
K10673 streptomycin 3-kinase [EC:2.7.1.87]	0	0	0	0	0	0	0	0	1	2	2	1
K17910 aminoglycoside 2''-phosphotransferase [EC:2.7.1.190]	0	0	0	0	0	0	0	1	0	0	0	0
K18220 ribosomal protection tetracycline resistance protein	2052	1884	2078	2086	2379	2257	2367	2180	2889	2838	2833	2991
K18234 virginiamycin A acetyltransferase [EC:2.3.1.-]	737	843	840	842	1399	1438	1457	1398	1430	1402	1523	1385

K18235 virginiamycin B lyase [EC:4.2.99.-]	1648	1626	1535	1623	949	925	486	554	488	520	517	545
K18554 chloramphenicol 3-O phosphotransferase [EC:2.7.1.-]	0	0	0	1	3	0	0	0	0	0	0	0
K18555 fluoroquinolone resistance protein	0	0	0	0	0	0	0	0	5	2	0	4
K18815 aminoglycoside 6'-N-acetyltransferase I [EC:2.3.1.82]	33	42	40	38	11	15	8	7	72	63	58	68
K18816 aminoglycoside 6'-N-acetyltransferase I [EC:2.3.1.82]	4	2	4	6	20	27	19	24	6	7	7	4
K19271 chloramphenicol O-acetyltransferase type A [EC:2.3.1.28]	1312	1279	1413	1391	1704	1679	1651	1761	1813	1853	1870	1871
K19272 aminoglycoside 3'-phosphotransferase I [EC:2.7.1.95]	0	0	0	0	0	0	1	2	1	6	0	0
K19273 streptothricin acetyltransferase [EC:2.3.-.-]	54	56	50	53	3	7	2	2	0	5	2	3
K19275 aminoglycoside 3-N-acetyltransferase II [EC:2.3.1.81]	0	0	1	0	0	0	0	0	0	0	5	0
K19278 aminoglycoside 6'-N-acetyltransferase Ib [EC:2.3.1.82]	3	2	3	4	25	19	24	22	0	1	1	0
K19299 aminoglycoside 3'-phosphotransferase III [EC:2.7.1.95]	93	66	69	103	11	6	9	15	1	2	5	7
K19300 aminoglycoside 3'-phosphotransferase II [EC:2.7.1.95]	1371	1300	1270	1315	705	745	672	740	422	348	424	424
K19545 lincosamide nucleotidyltransferase A/C/D/E	30	44	53	45	120	135	114	109	21	40	24	23
K19883 bifunctional aminoglycoside 6'-N-acetyltransferase / aminoglycoside 2''-phosphotransferase [EC:2.3.1.82 2.7.1.190]	11	5	9	9	26	30	34	23	0	4	8	4
K21252 fosfomycin resistance protein FosX	1	2	0	0	0	0	2	1	4	2	0	0
K21253 glutathione S-transferase fosA [EC:2.5.1.18]	691	611	558	628	347	302	284	348	205	175	167	211
K21265 glutathione S-transferase fosA5 [EC:2.5.1.18]	235	258	248	282	181	164	158	171	388	348	317	361
Not Included in Pathway or Brite	1286209	1286157	1270584	1287391	1247267	1255851	1233992	1242086	1221688	1222085	1215842	1223116
Unclassified: metabolism	532051	531480	522219	529695	514625	518736	514880	515915	514840	515478	511658	516219
ko99980 Enzymes with EC numbers	437737	437440	431257	436711	442535	445609	443797	442622	435652	435982	433944	435628
K00046 gluconate 5-dehydrogenase [EC:1.1.1.69]	1400	1484	1627	1316	2431	2388	2568	2357	2272	2269	2350	2215
K00063 D-arabinose 1-dehydrogenase [EC:1.1.1.117]	11	10	6	3	0	0	0	0	1	2	3	2
K00091 dihydroflavonol-4-reductase [EC:1.1.1.219]	1278	1344	1248	1261	680	704	681	688	650	614	587	565
K00098 L-idonate 5-dehydrogenase [EC:1.1.1.264]	222	215	218	245	339	347	365	307	63	78	81	60
K00119 alcohol dehydrogenase (nicotinoprotein) [EC:1.1.99.36]	0	1	0	0	0	0	0	0	0	0	0	1
K00153 S-(hydroxymethyl)mycothiol dehydrogenase [EC:1.1.1.306]	1	0	0	0	0	1	0	0	0	0	0	0
K00154 coniferyl-aldehyde dehydrogenase [EC:1.2.1.68]	2579	2568	2297	2440	1396	1421	1201	1385	753	772	759	796
K00179 indolepyruvate ferredoxin oxidoreductase, alpha subunit [EC:1.2.7.8]	1241	1392	1526	1362	3002	3313	3246	3171	2618	2545	2492	2465
K00180 indolepyruvate ferredoxin oxidoreductase, beta subunit [EC:1.2.7.8]	407	459	474	475	1148	1194	1137	1105	1002	877	909	900
K00219 2,4-dienoyl-CoA reductase (NADPH2) [EC:1.3.1.34]	7287	7058	6376	7036	3916	3939	3732	4163	3648	3562	3319	3687
K00221 alkylmercury lyase [EC:4.99.1.2]	0	0	0	0	0	0	0	0	0	0	1	3
K00298 N5-(carboxyethyl)ornithine synthase [EC:1.5.1.24]	1	2	1	3	19	23	28	15	0	0	0	1
K00311 electron-transferring-flavoprotein dehydrogenase [EC:1.5.5.1]	2197	2191	2092	2242	1201	1200	1126	1233	696	625	712	678
K00313 electron transfer flavoprotein-quinone oxidoreductase [EC:1.5.5.-]	614	578	641	627	905	938	1021	911	369	342	347	313
K00344 NADPH:quinone reductase [EC:1.6.5.5]	3174	3057	2843	3074	1914	1986	2004	2169	1662	1443	1452	1557
K00346 Na+-transporting NADH:ubiquinone oxidoreductase subunit A [EC:7.2.1.1]	2033	1991	2147	2017	2131	2081	2104	2109	1554	1414	1458	1484
K00347 Na+-transporting NADH:ubiquinone oxidoreductase subunit B [EC:7.2.1.1]	2884	2973	3022	2953	3406	3504	3470	3595	3299	3214	3213	3120
K00348 Na+-transporting NADH:ubiquinone oxidoreductase subunit C [EC:7.2.1.1]	1606	1610	1737	1753	1971	2027	2094	2018	1811	1826	1787	1764
K00349 Na+-transporting NADH:ubiquinone oxidoreductase subunit D [EC:7.2.1.1]	1341	1369	1288	1220	1709	1678	1751	1678	1709	1518	1532	1583
K00350 Na+-transporting NADH:ubiquinone oxidoreductase subunit E [EC:7.2.1.1]	1432	1380	1506	1402	1692	1676	1604	1723	1578	1518	1526	1495
K00351 Na+-transporting NADH:ubiquinone oxidoreductase subunit F [EC:7.2.1.1]	1102	1198	1197	1204	2510	2644	2621	2560	2428	2301	2363	2376
K00354 NADPH2 dehydrogenase [EC:1.6.99.1]	15	5	7	7	3	5	3	3	15	8	9	7
K00428 cytochrome c peroxidase [EC:1.11.1.5]	1524	1414	1517	1574	955	865	836	845	595	562	552	585
K00433 non-heme chloroperoxidase [EC:1.11.1.10]	1314	1203	1202	1274	521	517	487	511	1216	1162	1258	1182
K00437 [NiFe] hydrogenase large subunit [EC:1.12.2.1]	14	16	11	15	3	5	6	2	9	32	15	21
K00520 mercuric reductase [EC:1.16.1.1]	3453	3328	3393	3341	2355	2357	2394	2327	962	928	848	875
K00532 ferredoxin hydrogenase [EC:1.12.7.2]	0	2	5	0	0	0	0	0	13	15	10	14
K00533 ferredoxin hydrogenase large subunit [EC:1.12.7.2]	30	33	33	32	23	31	45	43	40	40	57	50
K00534 ferredoxin hydrogenase small subunit [EC:1.12.7.2]	2	2	2	1	2	2	1	1	1	0	0	0
K00537 arsenate reductase (glutaredoxin) [EC:1.20.4.1]	1549	1533	1649	1645	1707	1747	1747	1747	1614	1466	1612	1653
K00573 protein-L-isoaspartate(D-aspartate) O-methyltransferase [EC:2.1.1.77]	1440	1403	1296	1384	823	873	822	821	827	851	827	826
K00574 cyclopropane-fatty-acyl-phospholipid synthase [EC:2.1.1.79]	4102	3968	3878	4032	2403	2470	2445	2540	3089	3323	2982	3308
K00598 trans-aconitate 2-methyltransferase [EC:2.1.1.144]	1470	1536	1405	1499	775	763	732	774	958	1036	968	1080
K00612 carbamoyltransferase [EC:2.1.3.-]	4985	4813	4695	4599	2587	2488	2580	2818	1604	1484	1378	1500
K00633 galactoside O-acetyltransferase [EC:2.3.1.18]	179	178	188	173	106	146	237	220	287	283	309	318
K00661 maltose O-acetyltransferase [EC:2.3.1.79]	1866	1709	1868	1765	2611	2835	2581	2498	3133	3199	3257	3178
K00675 N-hydroxyarylamine O-acetyltransferase [EC:2.3.1.118]	1849	1860	1832	1827	1146	1063	1028	1103	1285	1193	1203	1176
K00684 leucyl/phenylalanyl-tRNA---protein transferase [EC:2.3.2.6]	1330	1296	1304	1409	867	799	863	780	953	838	850	924
K00754 L-malate glycosyltransferase [EC:2.4.1.-]	3	0	1	2	2	3	0	1	2	0	0	1
K00786 glycosyltransferase [EC:2.4.-.-]	34	18	47	31	5	0	9	5	30	32	36	29
K00809 deoxyhypusine synthase [EC:2.5.1.46]	1	2	2	3	2	2	1	0	3	10	2	12
K00862 erythritol kinase (D-erythritol 1-phosphate-forming) [EC:2.7.1.215]	1	1	0	0	0	2	0	0	4	1	0	3
K00906 isocitrate dehydrogenase kinase/phosphatase [EC:2.7.11.5 3.1.3.-]	3721	3643	3445	3612	2290	2252	2120	2079	2278	2493	2246	2354
K00924 kinase [EC:2.7.1.-]	1	0	0	0	0	0	0	0	0	0	0	0
K00982 [glutamine synthetase] adenylyltransferase / [glutamine synthetase]-adenylyl-L-tyrosine phosphorylase [EC:2.7.7.42 2.7.7.89]	6777	6719	6589	6610	3951	4214	4150	4148	4419	4080	3935	4426
K00986 RNA-directed DNA polymerase [EC:2.7.7.49]	5336	5187	5345	5269	2996	2902	2742	2906	1716	1725	1833	1793
K01002 phosphoglycerol transferase [EC:2.7.8.20]	1309	1476	1377	1487	1057	1048	1014	982	2004	2203	2075	2255
K01023 arylsulfate sulfotransferase [EC:2.8.2.22]	2643	2566	2575	2522	1939	1994	1948	1921	2644	2590	2688	2558
K01050 cholinesterase [EC:3.1.1.8]	4	2	2	1	0	0	0	0	4	4	3	3
K01066 acetyl esterase [EC:3.1.1.-]	2189	2314	2216	2225	1520	1457	1434	1513	1507	1518	1397	1528
K01073 acyl-CoA hydrolase [EC:3.1.2.20]	0	0	0	1	0	0	0	0	0	1	0	0
K01090 protein phosphatase [EC:3.1.3.16]	4	4	2	1	2	0	2	0	0	0	0	0
K01118 FMN-dependent NADH-azoreductase [EC:1.7.1.17]	2408	2436	2353	2345	1337	1369	1327	1450	1638	1596	1487	1602
K01133 choline-sulfatase [EC:3.1.6.6]	3914	3944	3938	3804	4225	4374	4159	4385	3245	3226	3194	3086
K01138 uncharacterized sulfatase [EC:3.1.6.-]	31	28	34	48	48	67	66	51	187	241	209	183
K01143 exodeoxyribonuclease (lambda-induced) [EC:3.1.11.3]	0	0	0	1	5	5	4	8	2	1	3	1
K01144 exodeoxyribonuclease V [EC:3.1.11.5]	232	236	248	277	501	483	529	538	202	238	234	229
K01150 deoxyribonuclease I [EC:3.1.21.1]	1512	1506	1472	1503	915	947	822	929	976	992	1024	1030
K01174 micrococcal nuclease [EC:3.1.31.1]	76	44	50	70	4	1	9	13	39	46	34	52
K01175 esterase [EC:3.1.-.-]	485	499	456	487	350	358	288	331	572	705	634	715
K01180 endo-1,3(4)-beta-glucanase [EC:3.2.1.6]	0	0	0	0	0	0	0	1	2	0	0	0
K01181 endo-1,4-beta-xylanase [EC:3.2.1.8]	1114	1306	1424	1342	4610	4266	4559	4452	3280	3120	3293	3066
K01185 lysozyme [EC:3.2.1.17]	1091	1088	1207	1150	2493	2542	2547	2546	1575	1490	1500	1635
K01215 glucan 1,6-alpha-glucosidase [EC:3.2.1.70]	136	188	167	195	46	32	42	24	208	229	206	239
K01219 beta-agarase [EC:3.2.1.81]	1	4	1	0	0	0	0	0	0	0	1	0
K01224 arabinogalactan endo-1,4-beta-galactosidase [EC:3.2.1.89]	1045	1043	1061	1079	986	1009	1044	975	916	977	927	1003
K01235 alpha-glucuronidase [EC:3.2.1.139]	1903	2004	2062	1937	5592	5467	5654	5562	4759	4623	4946	4528
K01250 pyrimidine-specific ribonucleoside hydrolase [EC:3.2.-.-]	946	853	924	922	505	487	478	455	974	1056	966	1101
K01274 D-alanyl-D-alanine dipeptidase [EC:3.4.13.-]	0	0	0	0	0	0	1	0	0	0	2	3
K01286 D-alanyl-D-alanine carboxypeptidase [EC:3.4.16.4]	104	72	99	75	8	16	17	10	76	120	119	108
K01447 N-acetylmuramoyl-L-alanine amidase [EC:3.5.1.28]	1357	1312	1259	1281	1170	1172	1032	1220	735	673	731	714
K01449 N-acetylmuramoyl-L-alanine amidase [EC:3.5.1.28]	340	370	338	429	110	129	125	84	275	349	329	365
K01461 N-acyl-D-glutamate deacylase [EC:3.5.1.82]	0	0	0	0	0	0	0	0	1	0	0	0
K01462 peptide deformylase [EC:3.5.1.88]	2994	2874	3015	2838	2477	2504	2611	2642	2395	2516	2458	2478
K01463 N-acetylglucosamine malate deacetylase 1 [EC:3.5.1.-]	25	18	18	33	1	0	0	0	61	60	57	62
K01475 isatin hydrolase [EC:3.5.2.20]	0	0	1	1	1	2	1	0	1	1	0	0
K01526 guanosine-diphosphatase [EC:3.6.1.42]	0	0	2	0	0	0	0	0	0	0	0	0
K01529 inosine/xanthosine triphosphatase [EC:3.6.1.-]	294	308	337	367	220	299	223	233	437	457	415	423
K01530 phospholipid-translocating ATPase [EC:7.6.2.1]	0	2	0	0	0	0	0	0	0	0	0	0
K01531 P-type Mg2+ transporter [EC:7.2.2.14]	5853	5767	5504	5708	3056	3254	3186	3088	5485	6163	5833	5893
K01533 P-type Cu2+ transporter [EC:7.2.2.9]	4687	4700	4836	4818	6208	6204	6110	6128	4162	4113	4461	4127
K01534 Cd2+/Zn2+-exporting ATPase [EC:3.6.3.3 7.2.2.12]	7640	7815	7493	7801	7676	7587	7833	7728	7780	8037	7768	7759
K01536 P-type Na+/K+ transporter [EC:7.2.2.3 7.2.2.-]	1	0	0	0	0	0	0	0	0	0	1	1
K01537 P-type Ca2+ transporter type 2C [EC:7.2.2.10]	5918	5708	6117	5789	7139	7144	7414	7174	7673	7943	8130	8045
K01567 peptidoglycan-N-acetylmuramic acid deacetylase [EC:3.5.1.-]	338	295	314	330	68	92	72	89	279	303	291	288
K01616 2-oxoglutarate decarboxylase [EC:4.1.1.71]	0	0	0	0	0	0	0	1	0	0	0	0
K01671 sulfofructosephosphate aldolase [EC:4.1.2.57]	202	179	214	207	346	345	308	352	45	53	37	58
K01683 D-arabinonate dehydratase [EC:4.2.1.5]	8	4	2	5	1	2	0	1	3	5	3	2
K01726 gamma-carbonic anhydrase [EC:4.2.1.-]	2	1	4	1	0	0	43	40	0	0	1	0
K01727 hyaluronate lyase [EC:4.2.2.1]	2388	2249	2327	2332	510	486	426	441	1451	1716	1519	1573
K01751 diaminopropionate ammonia-lyase [EC:4.3.1.15]	1205	1265	1182	1222	236	277	319	247	1537	1733	1579	1721
K01766 cysteine sulfinate desulfinase [EC:4.4.1.-]	2127	2152	1959	2084	1324	1267	1226	1307	1465	1437	1416	1570
K01780 histidine racemase [EC:5.1.1.24]	42	33	52	59	9	9	3	7	108	93	98	93
K01802 peptidylprolyl isomerase [EC:5.2.1.8]	720	677	683	649	710	615	546	548	415	417	421	453
K01811 alpha-D-xyloside xylohydrolase [EC:3.2.1.177]	8507	8600	8912	8899	13729	13646	13943	13554	14911	14534	14856	14297
K01932 gamma-polyglutamate synthase [EC:6.3.2.-]	3	2	2	3	0	1	0	1	0	0	5	1
K01974 RNA 3'-terminal phosphate cyclase (ATP) [EC:6.5.1.4]	44	40	36	25	29	37	133	151	60	65	61	53
K02082 tagatose-6-phosphate ketose/aldose isomerase [EC:5.-.-.-]	298	326	325	378	436	418	428	409	97	79	86	122
K02182 carnitine-CoA ligase [EC:6.2.1.48]	328	304	345	314	479	515	527	477	102	77	97	94
K02217 ferritin [EC:1.16.3.2]	951	945	1036	992	1462	1385	1402	1386	1315	1297	1262	1309
K02334 DNA polymerase bacteriophage-type [EC:2.7.7.7]	230	210	235	214	217	217	243	220	522	525	512	542

K02352 lactonase [EC:3.1.1.-]	239	238	271	287	71	54	61	65	561	643	581	607
K02431 L-fucose mutarotase [EC:5.1.3.29]	340	334	285	339	91	107	119	128	639	668	638	632
K02467 arabinose 5-phosphate isomerase [EC:5.3.1.13]	566	590	548	637	390	398	424	414	852	775	841	856
K02558 UDP-N-acetylmuramate: L-alanyl-gamma-D-glutamyl-meso-diaminopimelate ligase [EC:6.3.2.45]	3195	3127	2938	3029	1924	1959	1902	1978	2038	2079	1810	2016
K02846 N-methyl-L-tryptophan oxidase [EC:1.5.3.-]	573	612	586	605	402	416	393	449	884	907	877	888
K03207 colanic acid biosynthesis protein WcaH [EC:3.6.1.-]	110	63	69	74	129	129	127	141	29	36	31	26
K03387 alkyl hydroperoxide reductase subunit F [EC:1.6.4.-]	2467	2508	2509	2517	4401	4369	4239	4367	3952	3967	3993	3895
K03424 TatD DNase family protein [EC:3.1.21.-]	6516	6707	6477	6518	6537	6717	6481	6635	6397	6483	6037	6214
K03478 chitin disaccharide deacetylase [EC:3.5.1.105]	460	460	435	469	283	324	294	299	590	673	657	669
K03518 aerobic carbon-monoxide dehydrogenase small subunit [EC:1.2.5.3]	751	741	709	706	173	204	204	197	516	554	535	512
K03519 aerobic carbon-monoxide dehydrogenase medium subunit [EC:1.2.5.3]	86	80	80	74	14	12	15	20	58	62	71	47
K03520 aerobic carbon-monoxide dehydrogenase large subunit [EC:1.2.5.3]	0	1	2	1	0	0	0	1	0	3	2	3
K03534 L-rhamnose mutarotase [EC:5.1.3.32]	827	854	836	928	1732	1736	1785	1735	1475	1547	1571	1534
K03564 peroxiredoxin Q/BCP [EC:1.11.1.15]	1489	1524	1465	1572	1628	1607	1641	1589	1507	1512	1552	1468
K03578 ATP-dependent helicase HrpA [EC:3.6.4.13]	8193	8066	7896	8087	4979	4854	4632	5044	5089	5048	4972	5300
K03579 ATP-dependent helicase HrpB [EC:3.6.4.13]	2635	2606	2287	2537	1634	1599	1786	1847	2407	2238	2337	2415
K03614 Na+-translocating ferredoxin:NAD+ oxidoreductase subunit D [EC:7.2.1.2]	1788	1881	1906	1932	2678	2690	2784	2637	2813	2798	2721	2671
K03615 Na+-translocating ferredoxin:NAD+ oxidoreductase subunit C [EC:7.2.1.2]	2458	2390	2561	2449	3378	3511	3813	3847	3713	3866	3575	3671
K03616 Na+-translocating ferredoxin:NAD+ oxidoreductase subunit B [EC:7.2.1.2]	1459	1487	1442	1434	2233	2227	2099	2110	2132	2182	2129	2002
K03716 spore photoproduct lyase [EC:4.1.99.14]	384	345	417	334	77	69	82	63	409	459	388	458
K03725 archaea-specific helicase [EC:3.6.4.-]	0	2	3	2	1	3	3	2	4	13	2	10
K03726 helicase [EC:3.6.4.-]	847	850	781	872	330	352	337	314	2230	2165	2111	2204
K03727 ATP-dependent RNA helicase HelY [EC:3.6.4.-]	4	3	2	5	3	4	3	7	10	23	12	11
K03734 FAD:protein FMN transferase [EC:2.7.1.180]	4499	4658	4617	4611	5895	5853	6076	5804	5568	5231	5525	5470
K03741 arsenate reductase (thioredoxin) [EC:1.20.4.4]	1149	1264	1242	1212	1661	1661	1592	1723	1019	983	1065	1011
K03793 pteridine reductase [EC:1.5.1.33]	0	0	0	0	2	0	1	0	0	6	3	2
K03802 cyanophycin synthetase [EC:6.3.2.29 6.3.2.30]	5	3	3	2	0	0	0	0	15	9	13	22
K03813 molybdenum transport protein [EC:2.4.2.-]	20	26	24	46	67	43	45	59	114	85	94	82
K03818 putative colanic acid biosynthesis acetyltransferase WcaF [EC:2.3.1.-]	244	242	252	252	489	524	530	516	216	239	248	243
K03819 putative colanic acid biosynthesis acetyltransferase WcaB [EC:2.3.1.-]	91	83	84	100	137	169	118	138	19	23	17	23
K03820 apolipoprotein N-acyltransferase [EC:2.3.1.269]	3562	3430	3239	3323	2136	2127	2022	2093	2118	2050	1963	2117
K03824 putative acetyltransferase [EC:2.3.1.-]	1658	1765	1588	1743	1061	1021	1018	958	844	849	894	904
K03825 putative acetyltransferase [EC:2.3.1.-]	765	758	745	792	500	496	536	496	628	615	516	544
K03826 putative acetyltransferase [EC:2.3.1.-]	542	528	579	557	221	272	252	274	643	657	612	706
K03827 putative acetyltransferase [EC:2.3.1.-]	2075	2110	2133	2053	3205	3421	3404	3456	2816	2875	2777	2809
K03828 putative acetyltransferase [EC:2.3.1.-]	319	353	312	320	203	219	248	234	421	437	361	465
K03829 putative acetyltransferase [EC:2.3.1.-]	198	174	178	175	139	193	161	133	200	227	220	254
K03830 putative acetyltransferase [EC:2.3.1.-]	162	203	203	227	64	90	69	99	448	434	476	434
K03924 MoxR-like ATPase [EC:3.6.3.-]	8085	8174	8050	7997	10280	10356	10491	10375	8894	8407	8921	8443
K03928 carboxylesterase [EC:3.1.1.1]	61	73	69	55	8	16	4	11	90	97	81	95
K03929 para-nitrobenzyl esterase [EC:3.1.1.-]	1389	1248	1218	1308	723	748	743	767	1806	1921	1763	1807
K03930 putative tributyrin esterase [EC:3.1.1.-]	59	60	74	51	18	16	12	16	48	87	52	68
K04068 anaerobic ribonucleoside-triphosphate reductase activating protein [EC:1.97.1.4]	1883	1836	1744	1872	2029	1969	1939	1999	1817	1731	1769	1794
K04069 pyruvate formate lyase activating enzyme [EC:1.97.1.4]	3999	4058	3947	3956	4240	4306	4357	4217	5173	5430	5107	5439
K04070 putative pyruvate formate lyase activating enzyme [EC:1.97.1.4]	488	459	417	466	115	167	162	154	442	462	500	501
K04087 membrane protease subunit HflC [EC:3.4.-.-]	2837	2926	2818	2785	1755	1698	1630	1845	1619	1679	1492	1761
K04088 membrane protease subunit HflK [EC:3.4.-.-]	2223	2315	2242	2270	1347	1503	1307	1356	1477	1508	1526	1487
K04766 acetoin utilization protein AcuA [EC:2.3.1.-]	2	0	0	0	0	0	0	0	0	0	0	0
K04844 hypothetical glycosyl hydrolase [EC:3.2.1.-]	505	454	476	451	165	200	161	133	340	425	432	447
K04940 opine dehydrogenase [EC:1.5.1.28]	64	76	57	46	132	133	138	114	99	103	98	123
K05303 O-methyltransferase [EC:2.1.1.-]	14	16	10	15	10	13	20	11	0	0	1	2
K05521 ADP-ribosyl-[dinitrogen reductase] hydrolase [EC:3.2.2.24]	48	55	52	45	62	111	64	89	0	0	0	0
K05715 2-phosphoglycerate kinase [EC:2.7.2.-]	0	0	2	0	0	0	0	1	2	3	1	9
K05716 cyclic 2,3-diphosphoglycerate synthetase [EC:4.6.1.-]	0	0	0	0	0	0	0	0	4	14	6	9
K05810 polyphenol oxidase [EC:1.10.3.-]	2883	2920	2931	2928	2973	2759	2945	2829	3200	2995	3186	3137
K05882 aryl-alcohol dehydrogenase (NADP+) [EC:1.1.1.91]	0	0	0	1	0	0	0	1	0	0	0	0
K05886 serine 3-dehydrogenase (NADP+) [EC:1.1.1.276]	0	4	2	1	0	0	2	1	1	0	1	0
K05888 L-gulono-1,4-lactone dehydrogenase [EC:1.1.2.-]	2708	2757	2733	2740	1838	1748	1722	1763	894	788	829	738
K05910 NADH peroxidase [EC:1.11.1.1]	55	65	72	48	18	25	24	28	94	84	91	93
K05919 superoxide reductase [EC:1.15.1.2]	692	606	611	641	251	274	300	253	447	462	480	522
K05922 quinone-reactive Ni/Fe-hydrogenase large subunit [EC:1.12.5.1]	0	0	2	0	0	0	0	0	0	0	0	1
K05970 sialate O-acetylesterase [EC:3.1.1.53]	7102	7807	8283	7434	23314	23653	24167	23593	17506	16863	18058	16656
K05985 ribonuclease M5 [EC:3.1.26.8]	93	71	93	92	38	49	59	41	59	82	87	93
K05989 alpha-L-rhamnosidase [EC:3.2.1.40]	8454	8889	9485	8975	23443	22912	23681	22780	19571	18763	19771	18592
K06015 N-acyl-D-amino-acid deacylase [EC:3.5.1.81]	1069	1055	967	1045	259	245	278	228	1460	1673	1548	1674
K06023 HPr kinase/phosphorylase [EC:2.7.11.- 2.7.4.-]	580	577	505	563	199	192	205	180	552	690	550	592
K06113 arabinan endo-1,5-alpha-L-arabinosidase [EC:3.2.1.99]	2654	2818	2955	2839	9865	9926	9938	9754	7232	7007	7367	6918
K06137 pyrroloquinoline-quinone synthase [EC:1.3.3.11]	1346	1239	1188	1226	601	586	536	598	840	840	824	890
K06139 PqqA peptide cyclase [EC:1.21.98.4]	380	368	387	413	111	112	98	115	832	826	754	815
K06221 2,5-diketo-D-gluconate reductase A [EC:1.1.1.346]	674	642	681	677	343	459	427	382	884	987	907	984
K06222 2,5-diketo-D-gluconate reductase B [EC:1.1.1.346]	1712	1695	1471	1676	835	791	705	892	1092	1113	1113	1023
K06324 spore coat protein A, manganese oxidase [EC:1.16.3.3]	3	2	5	1	0	0	0	0	0	4	3	6
K06379 stage II sporulation protein AB (anti-sigma F factor) [EC:2.7.11.1]	217	264	249	204	84	70	73	63	208	224	209	267
K06382 stage II sporulation protein E [EC:3.1.3.16]	808	797	789	758	229	280	275	236	598	671	648	653
K06441 ferredoxin hydrogenase gamma subunit [EC:1.12.7.2]	31	44	33	29	45	63	65	49	52	50	53	44
K06607 myo-inositol catabolism protein IolS [EC:1.1.1.-]	3	0	2	1	5	2	1	5	10	26	7	9
K06864 pyridinium-3,5-biscarboxylic acid mononucleotide sulfurtransferase [EC:4.4.1.37]	381	357	372	331	89	97	113	108	357	404	431	437
K06898 pyridinium-3,5-biscarboxylic acid mononucleotide synthase [EC:2.5.1.143]	422	452	372	390	148	142	151	139	388	465	455	420
K06937 7,8-dihydro-6-hydroxymethylpterin dimethyltransferase [EC:2.1.1.-]	76	76	59	76	49	38	42	48	155	191	166	198
K06988 8-hydroxy-5-deazaflavin:NADPH oxidoreductase [EC:1.5.1.40]	3	2	2	5	5	2	11	5	0	8	5	6
K07037 cyclic-di-AMP phosphodiesterase PgpH [EC:3.1.4.-]	1483	1586	1688	1598	4884	4914	4959	4555	3560	3388	3684	3574
K07053 3',5'-nucleoside bisphosphate phosphatase [EC:3.1.3.97]	2618	2540	2427	2630	1148	1195	1147	1211	2052	2180	2044	2116
K07067 diadenylate cyclase [EC:2.7.7.85]	3	11	3	1	2	0	0	0	2	9	7	7
K07094 putative glycerol-1-phosphate prenyltransferase [EC:2.5.1.-]	2	0	0	0	0	0	0	0	0	0	0	0
K07107 acyl-CoA thioester hydrolase [EC:3.1.2.-]	5006	5099	4991	5009	4382	4330	4180	4439	3229	3235	3166	3101
K07116 acyl-homoserine-lactone acylase [EC:3.5.1.97]	6946	6846	6694	6684	3790	3704	3469	3880	2128	2102	1932	2052
K07132 ATP-dependent target DNA activator [EC:3.6.1.3]	41	44	44	46	86	92	70	71	90	96	86	111
K07147 methionine sulfoxide reductase catalytic subunit [EC:1.8.-.-]	2309	2305	2279	2321	1399	1487	1383	1508	1380	1394	1444	1535
K07223 porphyrinogen peroxidase [EC:1.11.1.-]	2214	2286	2287	2207	2640	2633	2674	2761	2402	2182	2260	2279
K07229 ferric-chelate reductase (NADPH) [EC:1.16.1.9]	471	491	421	531	338	393	380	372	537	684	632	679
K07271 lipopolysaccharide cholinephosphotransferase [EC:2.7.8.-]	1789	1805	2012	1893	3883	3959	3993	3810	3262	3129	3477	3214
K07302 isoquinoline 1-oxidoreductase subunit alpha [EC:1.3.99.16]	1157	1148	1094	1097	609	593	569	615	388	299	346	354
K07303 isoquinoline 1-oxidoreductase subunit beta [EC:1.3.99.16]	6365	6231	5595	5976	3307	3043	2916	3420	2050	1745	1688	1832
K07304 peptide-methionine (S)-S-oxide reductase [EC:1.8.4.11]	1547	1401	1379	1397	918	850	828	929	1025	1005	1000	969
K07305 peptide-methionine (R)-S-oxide reductase [EC:1.8.4.12]	828	831	789	764	505	499	512	456	441	495	424	482
K07313 serine/threonine protein phosphatase 1 [EC:3.1.3.16]	529	509	488	530	444	422	401	417	562	682	615	646
K07314 serine/threonine protein phosphatase 2 [EC:3.1.3.16]	5	0	2	3	2	6	8	7	41	52	35	45
K07336 PKHD-type hydroxylase [EC:1.14.11.-]	1480	1352	1441	1433	694	649	727	702	885	887	811	893
K07393 glutathionyl-hydroquinone reductase [EC:1.8.5.7]	2078	2050	2029	1989	1375	1436	1384	1305	1169	1227	1174	1200
K07444 putative N6-adenine-specific DNA methylase [EC:2.1.1.-]	1328	1508	1730	1459	3244	3254	3212	3313	2748	2702	2790	2707
K07447 putative holliday junction resolvase [EC:3.1.-.-]	1589	1502	1547	1521	1526	1582	1562	1601	1512	1428	1532	1517
K07469 aldehyde oxidoreductase [EC:1.2.99.7]	137	137	121	151	100	102	120	92	306	315	264	294
K07503 endonuclease [EC:3.1.-.-]	2	8	3	5	3	0	2	3	4	10	12	16
K07557 archaeosine synthase [EC:2.6.1.97]	0	0	2	1	3	0	0	0	0	13	2	9
K07588 LAO/AO transport system kinase [EC:2.7.-.-]	856	896	866	823	2535	2576	2512	2484	2025	1870	1868	1889
K07749 formyl-CoA transferase [EC:2.8.3.16]	1174	1233	1188	1137	636	691	687	702	491	478	418	457
K07755 arsenite methyltransferase [EC:2.1.1.137]	12	23	22	21	8	14	13	16	16	39	25	28
K07757 sugar-phosphatase [EC:3.1.3.23]	410	481	442	446	315	380	351	315	675	727	637	694
K08282 non-specific serine/threonine protein kinase [EC:2.7.11.1]	23	40	27	28	14	16	8	12	31	31	20	48
K08286 protein-serine/threonine kinase [EC:2.7.11.-]	0	2	0	0	2	0	0	0	0	0	0	0
K08296 phosphohistidine phosphatase [EC:3.1.3.-]	991	911	847	946	517	507	500	611	624	550	562	595
K08297 crotonobetainyl-CoA dehydrogenase [EC:1.3.8.13]	261	276	262	272	424	433	410	411	82	89	60	106
K08298 L-carnitine CoA-transferase [EC:2.8.3.21]	237	280	269	336	404	418	469	401	159	127	127	154
K08299 crotonobetainyl-CoA hydratase [EC:4.2.1.149]	193	160	183	180	209	288	275	247	68	72	41	60
K08313 fructose-6-phosphate aldolase 1 [EC:4.1.2.-]	3	5	3	6	19	10	16	13	31	43	44	44
K08314 fructose-6-phosphate aldolase 2 [EC:4.1.2.-]	460	437	458	428	321	405	310	343	548	597	572	612
K08317 uncharacterized oxidoreductase [EC:1.1.-.-]	1006	971	837	933	819	823	750	771	770	835	810	865
K08319 L-threonate 2-dehydrogenase [EC:1.1.1.411]	283	334	335	351	91	97	80	83	740	694	677	711
K08320 (d)CTP diphosphatase [EC:3.6.1.65]	261	230	213	225	176	152	162	142	283	328	244	348
K08961 chondroitin-sulfate-ABC endolyase/exolyase [EC:4.2.2.20 4.2.2.21]	2667	3073	3195	2990	11208	11056	11143	10593	8386	8326	8746	7861
K09001 anhydro-N-acetylmuramic acid kinase [EC:2.7.1.170]	2615	2434	2385	2605	1548	1526	1572	1526	1496	1564	1512	1589

K09022 2-iminobutanoate/2-iminopropanoate deaminase [EC:3.5.99.10]	895	1002	1112	1053	1081	1150	1163	1061	1491	1648	1644	1632
K09121 pyridinium-3,5-bisthiocarboxylic acid mononucleotide nickel chelatase [EC:4.99.1.12]	481	478	503	481	138	143	165	173	593	605	680	654
K09773 [pyruvate, water dikinase]-phosphate phosphotransferase / [pyruvate, water dikinase] kinase [EC:2.7.4.28 2.7.11.33]	1691	1659	1670	1703	1027	1026	1025	1059	1182	1208	1131	1132
K09809 CDP-glycerol glycerophosphotransferase [EC:2.7.8.12]	324	326	315	306	57	68	61	74	403	485	479	526
K10231 kojibiose phosphorylase [EC:2.4.1.230]	357	395	400	382	90	81	66	76	229	260	270	251
K10253 DOPA 4,5-dioxygenase [EC:1.14.99.-]	629	646	563	607	344	363	337	322	183	174	180	179
K10254 oleate hydratase [EC:4.2.1.53]	1406	1432	1438	1490	284	357	339	264	1574	1767	1599	1642
K10530 L-lactate oxidase [EC:1.1.3.2]	6	9	5	7	12	16	7	6	92	76	71	97
K10531 L-ornithine N5-monooxygenase [EC:1.14.13.195 1.14.13.196]	1533	1560	1452	1460	848	810	731	833	483	478	412	456
K10670 glycine/sarcosine/betaine reductase complex component A [EC:1.21.4.2 1.21.4.3 1.21.4.4]	50	38	37	36	23	27	19	19	23	50	11	33
K10671 glycine reductase complex component B subunit alpha and beta [EC:1.21.4.2]	88	96	108	118	42	44	38	34	35	81	48	38
K10672 glycine reductase complex component B subunit gamma [EC:1.21.4.2]	143	153	146	150	57	36	62	25	73	81	56	74
K10677 inulin fructotransferase (DFA-I-forming) [EC:4.2.2.17]	0	0	0	0	0	1	0	0	1	0	0	1
K10708 fructoselysine 6-phosphate deglycase [EC:3.5.-.-]	292	371	319	353	366	394	379	378	139	132	122	122
K10709 fructoselysine 3-epimerase [EC:5.1.3.41]	223	196	227	227	319	296	294	282	15	25	23	21
K10710 fructoselysine 6-kinase [EC:2.7.1.218]	255	266	259	299	299	307	292	283	113	86	102	90
K10819 histidine kinase [EC:2.7.13.3]	7	2	5	12	5	7	10	9	2	1	1	0
K11065 thiol peroxidase, atypical 2-Cys peroxiredoxin [EC:1.11.1.15]	558	650	699	657	1192	1159	1213	1180	1020	1136	1144	1097
K11161 retinol dehydrogenase 13 [EC:1.1.1.300]	0	0	0	0	0	1	0	0	1	1	0	0
K11169 dehydrogenase/reductase SDR family member 13 [EC:1.1.-.-]	0	0	0	1	0	0	0	0	0	0	0	0
K11206 deaminated glutathione amidase [EC:3.5.1.128]	2079	2083	1942	2014	1278	1159	1171	1176	1291	1347	1301	1204
K11209 GSH-dependent disulfide-bond oxidoreductase [EC:1.8.4.-]	2309	2186	2276	2329	1322	1247	1215	1210	1819	1932	1765	1850
K11750 esterase FrsA [EC:3.1.-.-]	685	748	678	752	574	593	532	520	989	1042	968	1070
K11933 NADH oxidoreductase Hcr [EC:1.-.-.-]	574	563	536	576	420	375	421	381	794	806	751	746
K11938 HMP-PP phosphatase [EC:3.6.1.-]	548	526	522	608	397	430	452	392	791	864	807	843
K11941 glucans biosynthesis protein C [EC:2.1.-.-]	524	530	557	555	386	444	415	361	845	772	759	828
K11942 isobutyryl-CoA mutase [EC:5.4.99.13]	8	3	2	4	3	2	5	2	0	0	0	0
K12136 hydrogenase-4 component A [EC:1.-.-.-]	141	150	125	141	163	213	209	202	4	13	4	15
K12137 hydrogenase-4 component B [EC:1.-.-.-]	526	528	521	586	755	835	714	667	185	233	193	208
K12138 hydrogenase-4 component C [EC:1.-.-.-]	211	202	207	191	329	302	361	303	3	1	2	2
K12139 hydrogenase-4 component D [EC:1.-.-.-]	325	292	273	309	430	438	501	442	4	3	0	4
K12140 hydrogenase-4 component E [EC:1.-.-.-]	139	120	146	165	162	223	182	175	39	37	50	51
K12141 hydrogenase-4 component F [EC:1.-.-.-]	360	361	315	362	406	464	499	454	92	90	99	101
K12142 hydrogenase-4 component G [EC:1.-.-.-]	380	385	308	369	520	650	511	477	4	7	8	7
K12144 hydrogenase-4 component I [EC:1.-.-.-]	175	170	138	145	210	254	231	220	4	1	0	0
K12145 hydrogenase-4 component J [EC:1.-.-.-]	90	97	67	87	130	157	115	112	1	0	0	1
K12152 phosphatase NudJ [EC:3.6.1.-]	235	221	250	254	206	198	183	174	365	385	342	459
K12267 peptide methionine sulfoxide reductase msrA/msrB [EC:1.8.4.11 1.8.4.12]	1258	1155	1219	1256	1974	1920	1988	2000	1989	2016	2176	2045
K12374 arylsulfatase D/F/H [EC:3.1.6.-]	0	1	0	0	0	0	0	0	0	1	0	0
K12375 arylsulfatase I/J [EC:3.1.6.-]	0	0	0	0	0	2	0	0	0	0	0	0
K12376 arylsulfatase K [EC:3.1.6.-]	0	0	0	0	1	2	4	2	2	0	1	0
K12410 NAD-dependent deacetylase [EC:3.5.1.-]	3727	3641	3516	3529	3251	3230	3116	3200	3085	3024	3058	3239
K12508 feruloyl-CoA synthase [EC:6.2.1.34]	3	0	0	0	0	0	0	2	0	0	0	0
K12700 non-specific riboncleoside hydrolase [EC:3.2.-.-]	621	690	580	594	396	469	464	424	797	927	829	878
K12944 nucleoside triphosphatase [EC:3.6.1.-]	268	276	242	270	203	184	158	171	367	356	363	375
K12945 GDP-mannose pyrophosphatase NudK [EC:3.6.1.-]	149	132	118	106	127	203	183	152	52	59	57	58
K12950 manganese/zinc-transporting P-type ATPase C [EC:7.2.2.-]	3	0	4	1	3	0	2	0	2	0	3	1
K12952 cation-transporting P-type ATPase E [EC:7.2.2.-]	474	470	474	522	191	248	201	203	711	815	734	799
K12953 cation-transporting P-type ATPase F [EC:7.2.2.-]	1	1	0	1	0	0	0	0	0	0	0	1
K12954 cation-transporting P-type ATPase G [EC:7.2.2.-]	0	0	0	0	0	0	0	0	1	0	2	1
K12957 uncharacterized zinc-type alcohol dehydrogenase-like protein [EC:1.-.-.-]	704	720	665	731	453	494	505	407	1040	939	951	1015
K13043 N-succinyl-L-ornithine transcarbamylase [EC:2.1.3.11]	643	716	765	623	2214	2179	2297	2300	1597	1503	1646	1513
K13059 N-acetylhexosamine 1-kinase [EC:2.7.1.162]	3	2	2	0	0	1	2	0	9	6	3	15
K13069 diguanylate cyclase [EC:2.7.7.65]	583	610	559	540	373	454	425	460	875	868	928	1014
K13243 c-di-GMP-specific phosphodiesterase [EC:3.1.4.52]	13	10	6	9	21	36	31	36	106	142	121	131
K13244 c-di-GMP-specific phosphodiesterase [EC:3.1.4.52]	1	3	2	0	12	16	17	10	38	57	51	44
K13252 putrescine carbamoyltransferase [EC:2.1.3.6]	32	23	20	36	13	11	8	15	40	50	60	42
K13278 60kDa lysophospholipase [EC:3.1.1.5 3.1.1.47 3.5.1.1]	0	0	0	0	0	0	0	0	0	1	0	0
K13281 UV DNA damage endonuclease [EC:3.-.-.-]	10	22	7	11	5	5	2	3	39	20	37	31
K13292 phosphatidylglycerol---prolipoprotein diacylglyceryl transferase [EC:2.5.1.145]	2433	2358	2257	2205	1350	1376	1290	1310	1667	1664	1780	1825
K13574 uncharacterized oxidoreductase [EC:1.1.1.-]	845	843	818	982	828	892	858	810	971	932	957	950
K13670 putative glycosyltransferase [EC:2.4.-.-]	2	0	0	0	0	0	0	0	0	0	0	0
K13714 bifunctional autolysin [EC:3.5.1.28 3.2.1.96]	0	0	0	0	0	0	0	0	3	0	0	0
K13727 phenolic acid decarboxylase [EC:4.1.1.-]	332	315	305	329	257	232	252	232	442	449	405	456
K13930 triphosphoribosyl-dephospho-CoA synthase [EC:2.4.2.52]	987	1034	843	958	652	680	577	608	726	777	743	769
K13932 malonate decarboxylase beta subunit [EC:4.1.1.87]	1760	1736	1443	1601	986	940	967	932	968	1014	925	968
K13933 malonate decarboxylase gamma subunit [EC:4.1.1.87]	2006	1959	1751	1856	1022	1142	1156	1139	1008	969	881	1052
K13934 phosphoribosyl-dephospho-CoA transferase [EC:2.7.7.66]	1238	1289	1117	1231	623	647	664	750	724	719	647	761
K13935 malonate decarboxylase epsilon subunit [EC:2.3.1.39]	1411	1301	1106	1296	783	752	783	779	895	901	865	939
K13979 uncharacterized zinc-type alcohol dehydrogenase-like protein [EC:1.-.-.-]	2377	2393	2160	2239	1450	1561	1395	1448	1446	1495	1328	1401
K14189 uncharacterized oxidoreductase [EC:1.-.-.-]	134	126	139	143	202	195	205	187	0	2	0	0
K14351 arylacetamide deacetylase-like 3/4 [EC:3.1.1.-]	0	0	0	1	0	0	0	0	0	0	0	0
K14587 protein sgcE [EC:5.1.3.-]	1	2	0	0	5	1	1	4	3	6	3	1
K14659 chitooligosaccharide deacetylase [EC:3.5.1.-]	1	0	0	0	0	0	0	0	0	0	0	0
K14744 prophage endopeptidase [EC:3.4.-.-]	470	487	519	492	537	560	497	462	154	130	135	112
K15509 sulfopropanediol 3-dehydrogenase [EC:1.1.1.308]	2	2	2	1	0	0	0	0	8	6	1	5
K15515 sulfoacetaldehyde dehydrogenase [EC:1.2.1.81]	47	33	60	53	6	11	3	6	15	21	12	20
K15524 mannosylglycerate hydrolase [EC:3.2.1.170]	33	33	23	22	40	38	56	32	22	54	32	39
K15527 cysteate synthase [EC:2.5.1.76]	3	6	6	1	14	8	3	10	0	2	0	0
K15531 oligosaccharide reducing-end xylanase [EC:3.2.1.156]	116	120	119	120	47	78	71	89	291	304	289	286
K15532 unsaturated rhamnogalacturonyl hydrolase [EC:3.2.1.172]	4181	4169	4269	4227	5527	5703	5706	5562	5684	5877	5818	5777
K15533 1,3-beta-galactosyl-N-acetylhexosamine phosphorylase [EC:2.4.1.211]	1281	1343	1348	1305	273	262	263	227	1080	1238	1138	1138
K15534 beta-D-galactosyl-(1->4)-L-rhamnose phosphorylase [EC:2.4.1.247]	175	164	167	156	22	36	27	12	155	134	160	181
K15538 glycoprotein endo-alpha-1,2-mannosidase [EC:3.2.1.130]	0	0	1	2	3	2	3	1	0	1	1	0
K15915 undecaprenyl phosphate N,N'-diacetylbacillosamine 1-phosphate transferase [EC:2.7.8.36]	10	12	14	11	3	3	9	3	7	7	2	6
K15922 sulfoquinovosidase [EC:3.2.1.199]	31	23	16	31	4	8	7	9	81	53	61	71
K15924 glucuronoarabinoxylan endo-1,4-beta-xylanase [EC:3.2.1.136]	0	0	2	0	0	0	0	3	0	2	0	0
K16212 4-O-beta-D-mannosyl-D-glucose phosphorylase [EC:2.4.1.281]	996	1052	1141	1055	2836	2799	2814	2735	2303	2132	2201	2223
K16213 cellobiose epimerase [EC:5.1.3.11]	263	288	255	268	202	226	236	205	181	223	220	189
K16704 dTDP-4-amino-4,6-dideoxy-D-galactose acyltransferase [EC:2.3.1.210]	449	425	412	505	294	351	294	309	559	582	576	587
K16868 tellurite methyltransferase [EC:2.1.1.265]	407	393	403	368	241	232	226	244	553	592	569	585
K16869 octanoyl-[GcvH]:protein N-octanoyltransferase [EC:2.3.1.204]	40	47	48	35	8	6	8	4	55	78	47	54
K16872 beta-ketodecanoyl-[acyl-carrier-protein] synthase [EC:2.3.1.207]	1575	1648	1593	1633	986	866	846	918	541	477	479	445
K17064 D-2-hydroxyacid dehydrogenase (NADP+) [EC:1.1.1.272]	2	2	1	0	3	1	0	0	0	0	0	0
K17624 endo-alpha-N-acetylgalactosaminidase [EC:3.2.1.97]	190	246	235	237	45	30	27	31	193	265	224	272
K17735 carnitine 3-dehydrogenase [EC:1.1.1.108]	1264	1286	1051	1161	673	593	609	650	383	366	343	383
K17758 ADP-dependent NAD(P)H-hydrate dehydratase [EC:4.2.1.136]	4835	4787	4670	4685	5119	5273	5251	5197	4768	4831	4810	4949
K17759 NAD(P)H-hydrate epimerase [EC:5.1.99.6]	3	5	5	12	7	16	14	10	13	8	15	12
K17760 quinohemoprotein ethanol dehydrogenase [EC:1.1.9.1]	1	0	0	0	0	0	0	0	0	0	0	0
K17810 D-aspartate ligase [EC:6.3.1.12]	38	16	19	26	17	28	12	21	41	26	30	31
K17869 NADH oxidase (H2O-forming) [EC:1.6.3.4]	0	0	2	0	2	0	0	0	0	0	0	0
K17870 NADH oxidase (H2O2-forming) [EC:1.6.3.3]	3	2	2	5	0	0	3	0	3	13	7	2
K17883 mycothione reductase [EC:1.8.1.15]	1	0	1	1	1	0	0	0	0	0	0	0
K17884 archaetidylinositol phosphate synthase [EC:2.7.8.39]	0	0	0	0	0	2	0	0	5	8	0	3
K17948 N-acetylneuraminate epimerase [EC:5.1.3.24]	0	10	5	7	9	23	8	10	63	92	62	82
K17992 NADP-reducing hydrogenase subunit HndB [EC:1.12.1.3]	232	181	208	213	55	37	42	56	144	158	133	152
K17998 iron-hydrogenase subunit beta [EC:1.12.1.4]	0	0	0	0	0	0	0	1	0	0	0	0
K18006 [NiFe] hydrogenase diaphorase moiety small subunit [EC:1.12.1.2]	0	0	0	0	0	1	0	0	0	0	0	0
K18008 [NiFe] hydrogenase small subunit [EC:1.12.2.1]	12	9	7	8	3	0	2	7	4	21	4	15
K18016 membrane-bound hydrogenase subunit alpha [EC:1.12.7.2]	2	0	0	0	0	0	0	0	0	0	0	0
K18197 rhamnogalacturonan endolyase [EC:4.2.2.23]	409	430	437	418	559	633	655	618	40	87	56	95
K18198 rhamnogalacturonan exolyase [EC:4.2.2.24]	2	0	1	0	0	0	0	0	0	0	0	0
K18200 peptidylamidoglycolate lyase [EC:4.3.2.5]	0	0	0	1	0	0	0	0	0	0	0	0
K18205 non-reducing end beta-L-arabinofuranosidase [EC:3.2.1.185]	16	23	18	13	3	11	3	12	13	38	24	19
K18206 beta-L-arabinobiosidase [EC:3.2.1.187]	4	5	2	4	9	9	2	3	31	34	27	61
K18237 ribose 1,5-bisphosphate isomerase [EC:5.3.1.29]	96	96	108	97	20	17	26	17	47	82	74	68
K18244 acyl-CoA dehydrogenase [EC:1.3.99.-]	20	10	26	10	2	3	3	1	64	54	53	50
K18313 succinyl-CoA---D-citramalate CoA-transferase [EC:2.8.3.20]	1	0	1	0	0	0	0	0	0	0	0	0
K18330 NADP-reducing hydrogenase subunit HndA [EC:1.12.1.3]	340	374	464	401	910	859	874	962	802	737	705	741
K18331 NADP-reducing hydrogenase subunit HndC [EC:1.12.1.3]	1805	1795	1997	1877	3768	3833	3955	3649	3432	3426	3621	3376
K18332 NADP-reducing hydrogenase subunit HndD [EC:1.12.1.3]	2259	2162	2308	2232	3601	3634	3721	3530	3414	3548	3414	3424

K18367 CoA-dependent NAD(P)H sulfur oxidoreductase [EC:1.8.1.18]	2	3	0	1	2	1	4	3	1	1	2	1
K18425 3-hydroxy-D-aspartate aldolase [EC:4.1.3.41]	0	1	0	1	0	0	0	0	12	1	3	1
K18427 4-hydroxyphenylacetate decarboxylase large subunit [EC:4.1.1.83]	75	76	56	58	0	0	0	0	127	141	136	128
K18428 4-hydroxyphenylacetate decarboxylase small subunit [EC:4.1.1.83]	2	1	1	3	0	1	0	2	10	7	4	10
K18446 triphosphatase [EC:3.6.1.25]	883	838	868	859	640	624	602	603	1147	1168	1140	1228
K18456 8-oxoguanine deaminase [EC:3.5.4.32]	2422	2261	2266	2211	1384	1171	1214	1393	770	669	713	767
K18457 L-proline amide hydrolase [EC:3.5.1.101]	0	0	0	0	0	0	0	1	0	0	0	0
K18459 guanidinopropionase [EC:3.5.3.17]	1435	1475	1350	1441	791	781	794	882	523	424	417	478
K18478 sulfofructose kinase [EC:2.7.1.184]	239	207	197	216	302	351	379	352	87	96	84	93
K18479 sulfoquinovose isomerase [EC:5.3.1.31]	0	0	0	3	0	2	0	1	2	0	0	4
K18540 (R)-amidase [EC:3.5.1.100]	998	1001	945	1018	570	550	527	573	341	310	253	318
K18561 FAD-dependent fumarate reductase [EC:1.3.8.-]	0	0	0	0	1	0	0	0	0	0	0	0
K18581 unsaturated chondroitin disaccharide hydrolase [EC:3.2.1.180]	4075	3950	4098	4085	4389	5040	4713	4714	4703	4787	4786	4757
K18650 exo-poly-alpha-galacturonosidase [EC:3.2.1.82]	3	2	3	2	19	16	13	13	0	0	1	2
K18672 diadenylate cyclase [EC:2.7.7.85]	482	444	477	471	131	148	148	130	470	495	477	512
K18673 beta-glucoside kinase [EC:2.7.1.85]	373	359	375	372	101	85	98	136	708	822	736	731
K18678 phytol kinase [EC:2.7.1.182]	3	9	6	8	9	16	7	15	24	19	17	25
K18700 fluoroacetyl-CoA thioesterase [EC:3.1.2.29]	0	1	0	0	1	0	0	0	3	2	2	1
K18702 CoA:oxalate CoA-transferase [EC:2.8.3.19]	19	12	16	17	36	40	40	25	73	95	102	79
K18703 succinate---hydroxymethylglutarate CoA-transferase [EC:2.8.3.13]	0	1	1	1	1	2	1	3	0	0	1	1
K18704 CDP-ribitol ribitolphosphotransferase / teichoic acid ribitol-phosphate polymerase [EC:2.7.8.14 2.7.8.47]	0	5	2	6	2	5	4	3	1	4	1	10
K18783 nigerose phosphorylase [EC:2.4.1.279]	15	11	26	17	6	3	7	2	10	23	21	15
K18785 beta-1,4-mannooligosaccharide/beta-1,4-mannosyl-N-acetylglucosamine phosphorylase [EC:2.4.1.319 2.4.1.320]	836	884	941	912	1959	2064	2139	1945	1626	1590	1598	1437
K18786 cellobionic acid phosphorylase [EC:2.4.1.321]	3	2	0	0	2	1	0	1	0	1	3	1
K18820 oligosaccharide 4-alpha-D-glucosyltransferase [EC:2.4.1.161]	0	0	0	1	0	0	0	0	0	0	0	0
K18855 UDP-N-acetylglucosamine 3-dehydrogenase [EC:1.1.1.374]	2	2	6	3	1	2	0	2	6	15	9	9
K18910 D-psicose/D-tagatose/L-ribulose 3-epimerase [EC:5.1.3.30 5.1.3.31]	328	309	293	329	16	15	14	7	262	284	247	237
K18915 ferric-chelate reductase [NAD(P)H] [EC:1.16.1.10]	0	0	0	0	0	0	0	0	0	0	0	1
K18916 phosphonate dehydrogenase [EC:1.20.1.1]	365	315	290	326	506	506	471	483	57	55	65	54
K18931 AMP phosphorylase [EC:2.4.2.57]	0	0	0	0	0	0	0	0	0	1	0	0	1
K19049 chondroitin AC lyase [EC:4.2.2.5]	0	0	2	0	0	4	1	1	5	2	3	1
K19050 heparin lyase [EC:4.2.2.7]	9	8	13	8	33	28	24	24	27	20	28	30
K19052 heparan-sulfate lyase [EC:4.2.2.8]	1029	1138	1205	1119	4108	4055	4208	3910	3002	2930	3086	2943
K19068 UDP-2-acetamido-2,6-beta-L-arabino-hexul-4-ose reductase [EC:1.1.1.367]	468	489	495	457	117	117	106	141	271	272	259	251
K19179 glycerophosphoinositol glycerophosphodiesterase [EC:3.1.4.44]	0	0	3	0	1	0	0	1	0	0	0	0
K19181 1,5-anhydro-D-fructose reductase (1,5-anhydro-D-mannitol-forming) [EC:1.1.1.292]	0	0	0	0	0	0	4	2	0	4	5	0
K19245 sulfonate dioxygenase [EC:1.14.11.-]	0	0	2	0	0	0	0	0	0	0	0	0
K19265 L-glyceraldehyde 3-phosphate reductase [EC:1.1.1.-]	2247	2265	2198	2288	3333	3411	3333	3393	3030	2946	3251	3143
K19270 sugar-phosphatase [EC:3.1.3.23]	764	736	758	750	543	492	537	511	967	996	985	1079
K19356 lytic cellulose monooxygenase (C1-hydroxylating) [EC:1.14.99.54]	0	0	0	0	0	0	0	0	0	0	0	1
K19386 NADH kinase [EC:2.7.1.86]	0	0	0	0	0	0	0	0	0	0	0	1
K19405 protein arginine kinase [EC:2.7.14.1]	374	413	458	393	93	103	76	63	265	295	275	273
K19422 glycosyltransferase EpsD [EC:2.4.-.-]	8	15	6	11	41	27	31	30	14	20	6	13
K19426 pyruvyl transferase EpsI [EC:2.-.-.-]	0	0	0	0	0	0	0	1	0	0	0	0
K19427 glycosyltransferase EpsJ [EC:2.4.-.-]	67	69	58	67	44	34	40	34	86	116	87	85
K19428 sugar transferase EpsL [EC:2.-.-.-]	28	30	26	37	5	8	10	5	14	21	11	18
K19429 acetyltransferase EpsM [EC:2.3.1.-]	93	77	98	68	90	88	70	82	9	11	26	25
K19430 pyridoxal phosphate-dependent aminotransferase EpsN [EC:2.6.1.-]	6	2	3	3	1	1	3	2	3	2	4	1
K19431 pyruvyl transferase EpsO [EC:2.-.-.-]	17	18	14	11	3	2	3	1	0	0	3	0
K19597 Au+-exporting ATPase [EC:3.6.1.-]	0	0	0	0	0	0	0	2	0	0	0	0
K19711 L-serine 3-dehydrogenase (NAD+) [EC:1.1.1.387]	1331	1218	1237	1220	647	638	668	784	447	349	424	411
K19713 thiosulfate dehydrogenase [EC:1.8.2.2]	4279	4310	3943	4238	2287	2265	2103	2299	1317	1244	1268	1299
K19802 L-Ala-D/L-Glu epimerase [EC:5.1.1.20]	1838	1821	1871	1845	2858	3037	2860	2800	2730	2870	2637	2666
K19814 glutamate 2,3-aminomutase [EC:5.4.3.9]	0	2	0	1	0	0	0	0	0	1	0	1
K19817 coenzyme F420H2 oxidase [EC:1.5.3.22]	0	2	0	3	5	1	3	3	13	19	19	17
K19954 alcohol dehydrogenase [EC:1.1.1.-]	25	27	23	28	6	6	4	7	16	11	24	15
K19955 alcohol dehydrogenase [EC:1.1.1.-]	1095	1015	1041	1089	309	343	401	283	1282	1228	1274	1215
K19967 D-threonine aldolase [EC:4.1.2.42]	0	0	0	0	0	0	0	0	1	1	1	2
K19997 GlcNAc-P-P-Und epimerase [EC:5.1.3.26]	119	88	125	108	140	150	132	151	9	14	12	9
K20025 (R)-2-hydroxyisocaproyl-CoA dehydratase alpha subunit [EC:4.2.1.157]	30	18	19	18	0	0	0	0	6	7	6	8
K20026 (R)-2-hydroxyisocaproyl-CoA dehydratase beta subunit [EC:4.2.1.157]	16	17	8	15	8	15	10	16	5	7	4	5
K20037 choline trimethylamine-lyase activating enzyme [EC:1.97.1.-]	310	303	308	310	74	61	66	69	173	214	201	195
K20038 choline trimethylamine-lyase [EC:4.3.99.4]	970	829	896	885	319	331	312	299	600	627	677	633
K20141 2-oxoglutarate carboxylase small subunit [EC:6.4.1.7]	1	0	0	0	0	0	0	0	1	0	0	0
K20201 protein arginine phosphatase [EC:3.9.1.2]	2	1	3	1	0	0	0	1	0	4	1	1
K20202 sulfide dehydrogenase subunit alpha [EC:1.8.1.19]	1	1	1	1	5	2	3	3	3	3	3	2
K20203 sulfide dehydrogenase subunit beta [EC:1.8.1.19]	0	0	0	0	0	0	0	0	0	0	0	1
K20215 diphthine synthase [EC:2.1.1.98]	3	0	2	0	0	2	0	3	5	3	3	9
K20238 sphingolipid C9-methyltransferase [EC:2.1.1.317]	1	0	0	0	0	0	0	0	0	0	0	0
K20415 ACP-SH:acetate ligase [EC:6.2.1.35]	135	150	139	145	0	0	0	2	46	57	48	53
K20429 dTDP-4-amino-4,6-dideoxy-D-glucose transaminase [EC:2.6.1.33]	0	0	0	1	0	0	0	0	0	0	0	0
K20525 oligo-alginate lyase [EC:4.2.2.26]	2	1	2	1	2	1	2	3	1	1	1	1
K20542 endoglucanase [EC:3.2.1.4]	1658	1639	1503	1619	1401	1366	1349	1361	1882	1905	1827	1965
K20548 3,6-anhydro-alpha-L-galactose dehydrogenase [EC:1.2.1.92]	0	0	0	0	0	0	0	0	0	0	1	0
K20549 3,6-anhydro-L-galactonate cycloisomerase [EC:5.5.1.25]	0	1	0	0	1	0	0	1	0	0	2	0
K20757 threo-3-hydroxy-D-aspartate ammonia-lyase [EC:4.3.1.27]	0	0	0	0	0	0	0	0	0	1	0	1
K20817 N-acyl-D-aspartate deacylase [EC:3.5.1.83]	0	0	1	0	0	0	0	0	0	0	0	0
K20848 fructan beta-(2,1)-fructosidase [EC:3.2.1.153]	0	0	0	0	0	0	0	0	1	0	0	1
K20882 (R)-2-hydroxy-4-methylpentanoate CoA-transferase [EC:2.8.3.24]	15	12	17	29	3	9	12	11	45	36	40	48
K20883 alpha-1,2-colitosyltransferase [EC:2.4.1.341]	0	4	2	1	4	2	0	3	0	0	0	1
K20885 beta-1,2-mannobiose phosphorylase / 1,2-beta-oligomannan phosphorylase [EC:2.4.1.339 2.4.1.340]	434	470	475	473	1860	1711	1771	1767	1243	1209	1280	1280
K20902 D-erythrulose 4-kinase [EC:2.7.1.210]	2	0	0	2	3	0	0	2	14	17	16	17
K20903 (R)-2-hydroxyglutaryl-CoA dehydratase subunit alpha [EC:4.2.1.167]	34	32	40	34	62	76	56	55	19	44	34	26
K20904 (R)-2-hydroxyglutaryl-CoA dehydratase subunit beta [EC:4.2.1.167]	20	26	18	23	58	59	40	33	13	30	24	23
K20937 1-butanol dehydrogenase (quinone) [EC:1.1.5.11]	1	0	2	0	0	1	0	0	0	0	0	0
K20938 long-chain alkane monooxygenase [EC:1.14.14.28]	2111	2125	1864	2073	981	978	819	918	1628	1667	1558	1693
K21054 D-erythritol 1-phosphate dehydrogenase [EC:1.1.1.402]	0	0	0	0	0	0	2	0	0	1	2	2
K21055 3-deoxy-D-glycero-D-galacto-nononate 9-phosphatase [EC:3.1.3.103]	0	2	0	0	0	0	0	2	0	2	0	0
K21065 beta-1,2-mannosidase [EC:3.2.1.197]	1849	1943	2090	1816	7206	6993	7155	6757	5423	5253	5241	4939
K21132 alpha-mannan endo-1,2-alpha-mannanase / glycoprotein endo-alpha-1,2-mannosidase [EC:3.2.1.198 3.2.1.130]	1	2	1	3	3	5	7	3	0	0	3	1
K21279 3-deoxy-D-glycero-D-galacto-nononate 9-phosphate synthase [EC:2.5.1.132]	1	0	0	0	2	0	0	0	0	0	0	0
K21285 teichoic acid glycerol-phosphate primase [EC:2.7.8.44]	1	2	0	1	0	1	0	0	0	0	0	0
K21298 1,2-beta-oligoglucan phosphorylase [EC:2.4.1.333]	171	189	181	189	33	16	45	31	143	158	135	128
K21350 sucrose 6(F)-phosphate phosphorylase [EC:2.4.1.329]	2	0	0	3	0	0	1	0	5	5	8	17
K21351 sucrose:sucrose fructosyltransferase [EC:2.4.1.99]	0	0	0	0	0	0	0	1	0	0	0	0
K21355 1,2-alpha-glucosylglycerol phosphorylase [EC:2.4.1.332]	9	20	14	13	0	0	0	0	4	9	8	8
K21399 2-amino-4-ketopentanoate thiolase alpha subunit [EC:2.3.1.263]	1	0	2	0	0	0	0	0	0	0	1	0
K21400 2-amino-4-ketopentanoate thiolase beta subunit [EC:2.3.1.263]	2	2	0	3	2	2	1	0	5	0	2	4
K21416 acetoin:2,6-dichlorophenolindophenol oxidoreductase subunit alpha [EC:1.1.1.-]	1743	1772	1547	1733	841	809	868	885	1267	1199	1108	1233
K21417 acetoin:2,6-dichlorophenolindophenol oxidoreductase subunit beta [EC:1.1.1.-]	1971	1961	1848	2024	1099	1025	956	1098	1342	1433	1286	1388
K21420 leucyl-tRNA---protein transferase [EC:2.3.2.29]	958	973	953	997	486	489	501	515	309	297	285	260
K21430 aldose sugar dehydrogenase [EC:1.1.5.-]	2312	2431	2248	2329	1314	1253	1365	1341	1382	1308	1327	1522
K21481 heme oxygenase (mycobilin-producing) [EC:1.14.99.57]	0	0	0	1	0	0	0	0	0	0	0	0
K21511 capsid assembly protease [EC:3.4.21.-]	0	0	0	0	15	27	9	8	3	7	3	4
K21512 terminase, large subunit [EC:3.1.21.4]	0	3	2	1	35	12	19	22	8	9	14	15
K21527 adenine modification enzyme [EC:2.3.1.-]	0	8	3	0	5	0	0	0	0	0	6	0
K21567 ferredoxin/flavodoxin---NADP+ reductase [EC:1.18.1.2 1.19.1.1]	53	83	83	83	18	19	24	20	56	89	77	74
K21575 neopullulanase [EC:3.2.1.135]	1353	1464	1661	1451	4922	4939	4903	4610	3386	3258	3585	3364
K21576 glycine/sarcosine/betaine reductase complex component C subunit alpha [EC:1.21.4.2 1.21.4.3 1.21.4.4]	56	58	76	58	35	26	22	43	64	65	54	61
K21577 glycine/sarcosine/betaine reductase complex component C subunit beta [EC:1.21.4.2 1.21.4.3 1.21.4.4]	95	75	84	91	31	41	48	31	58	81	69	73
K21578 betaine reductase complex component B subunit alpha [EC:1.21.4.4]	38	56	47	41	8	5	8	10	28	19	21	22
K21579 betaine reductase complex component B subunit beta [EC:1.21.4.4]	40	36	37	38	8	21	7	2	21	32	27	19
K21583 sarcosine reductase complex component B subunit alpha [EC:1.21.4.3]	52	22	39	35	8	10	10	10	23	38	25	15
K21584 sarcosine reductase complex component B subunit beta [EC:1.21.4.3]	5	14	15	14	1	1	3	3	11	26	13	9
K21592 teichoic acid ribitol-phosphate primase [EC:2.7.8.46]	1	2	1	1	0	0	0	0	0	0	0	1
K21616 D-altritol 5-dehydrogenase [EC:1.1.1.407]	0	0	0	1	2	2	0	0	4	4	2	3
K21702 galactitol 2-dehydrogenase (L-tagatose-forming) [EC:1.1.1.406]	0	2	0	0	0	0	0	0	0	1	0	0
K21713 lytic chitin monoxygenase [EC:1.14.99.53]	53	33	40	35	5	7	11	6	39	59	24	52
K21750 3-deoxy-D-glycero-D-galacto-nononate cytidylyltransferase [EC:2.7.7.92]	2	1	0	0	2	2	0	0	0	0	0	3
K21887 cation-transporting P-type ATPase J [EC:7.2.2.-]	0	1	2	0	0	1	1	1	1	1	1	1
K21909 D-erythrulose 1-phosphate 3-epimerase [EC:5.1.3.38]	16	13	9	23	16	17	20	16	34	39	37	41

K21910 L-erythrulose 1-phosphate isomerase [EC:5.3.1.33]	14	23	17	16	1	5	4	2	6	10	11	3
K21911 D-erythrulose 4-phosphate isomerase [EC:5.3.1.34]	1	0	0	0	0	0	0	2	1	1	4	1
K21935 beta-lysine N6-acetyltransferase [EC:2.3.1.264]	0	3	0	0	0	0	0	0	0	0	1	3
K21936 anaerobilin synthase [EC:2.1.1.342]	124	118	121	143	75	91	85	95	272	232	225	278
K21948 3-dehydrotetronate 4-kinase [EC:2.7.1.217]	586	476	494	531	131	113	141	125	1019	999	1008	1070
K22015 formate dehydrogenase (acceptor) [EC:1.17.99.7]	2567	2482	2373	2812	1900	1930	1858	1796	3643	3605	3464	3741
K22024 4-phospho-D-threonate 3-dehydrogenase / 4-phospho-D-erythronate 3-dehydrogenase [EC:1.1.1.408 1.1.1.409]	2366	2228	2299	2442	969	1135	1081	954	2452	2680	2449	2569
K22025 D-erythronate 2-dehydrogenase [EC:1.1.1.410]	0	0	0	0	0	0	0	0	0	3	3	4
K22027 indole-3-acetate monooxygenase [EC:1.14.13.235]	2	0	0	3	0	0	0	0	0	0	0	0
K22078 protein-glucosylgalactosylhydroxylysine glucosidase [EC:3.2.1.107]	3	5	10	4	21	16	20	11	10	8	8	4
K22129 D-threonate/D-erythronate kinase [EC:2.7.1.219 2.7.1.220]	2485	2773	2557	2700	1411	1377	1439	1418	2868	2997	2876	2961
K22130 3-dehydro-4-phosphotetronate decarboxylase [EC:4.1.1.104]	87	65	62	76	20	20	12	23	126	141	97	110
K22131 2-dehydrotetronate isomerase [EC:5.3.1.35]	1647	1570	1483	1623	792	790	745	878	927	996	925	988
K22209 D(-)-tartrate dehydratase [EC:4.2.1.81]	1216	1172	1118	1222	661	691	596	666	386	399	372	390
K22213 6-methylsalicylate decarboxylase [EC:4.1.1.52]	3	4	0	1	0	0	0	0	0	0	0	0
K22247 polysialic-acid O-acetyltransferase [EC:2.3.1.136]	2	1	0	1	0	0	0	2	9	16	13	12
K22250 poly(3-hydroxyoctanoate) depolymerase [EC:3.1.1.76]	1413	1314	1315	1364	768	727	766	834	428	437	423	428
K22278 peptidoglycan-N-acetylglucosamine deacetylase [EC:3.5.1.104]	885	742	835	823	238	200	188	162	607	727	739	692
K22303 alpha-ketoglutarate-dependent sulfate ester dioxygenase [EC:1.14.11.-]	1557	1533	1458	1545	994	1019	927	1031	488	387	418	417
K22306 glucosyl-3-phosphoglycerate phosphatase [EC:3.1.3.85]	3	5	2	0	0	2	1	2	2	4	3	6
K22310 L-ornithine Nalpha-acyltransferase [EC:2.3.2.30]	0	0	0	0	0	0	1	0	0	4	2	0
K22338 formate dehydrogenase (NAD+, ferredoxin) subunit A [EC:1.17.1.11]	0	9	10	0	3	0	0	0	0	0	12	0
K22339 formate dehydrogenase (NAD+, ferredoxin) subunit B [EC:1.17.1.11]	2	1	2	1	2	1	3	0	0	2	4	2
K22340 formate dehydrogenase (NAD+, ferredoxin) subunit C [EC:1.17.1.11]	0	0	1	1	1	4	1	0	4	2	0	3
K22341 formate dehydrogenase (NAD+, ferredoxin) subunit [EC:1.17.1.11]	3	1	4	3	6	2	6	5	4	8	4	9
K22342 dimethylamine monooxygenase subunit A [EC:1.14.13.238]	0	0	0	0	1	0	0	0	0	0	0	0
K22343 dimethylamine monooxygenase subunit B [EC:1.14.13.238]	2	2	0	0	0	0	0	1	0	0	0	0
K22344 dimethylamine monooxygenase subunit C [EC:1.14.13.238]	3	0	1	0	0	0	0	0	0	0	0	0
K22348 manganese oxidase [EC:1.16.3.3]	107	134	116	104	14	6	13	12	218	221	206	181
K22349 manganese oxidase [EC:1.16.3.3]	3	0	3	13	0	0	0	0	0	0	0	0
K22405 NADH oxidase (H2O-forming) [EC:1.6.3.4]	251	254	250	231	30	29	31	28	332	317	327	396
K22409 N-acetylmuramoyl-L-alanine amidase [EC:3.5.1.28]	0	0	0	0	0	0	0	0	0	0	0	1
K22430 caffeyl-CoA reductase-Etf complex subunit CarC [EC:1.3.1.108]	41	38	36	38	0	2	2	1	24	16	15	21
K22431 caffeyl-CoA reductase-Etf complex subunit CarD [EC:1.3.1.108]	60	63	36	58	4	1	8	3	34	22	19	28
K22432 caffeyl-CoA reductase-Etf complex subunit CarE [EC:1.3.1.108]	547	504	554	557	115	124	116	91	331	406	299	294
K22438 trans-aconitate 3-methyltransferase [EC:2.1.1.145]	0	0	0	0	0	0	0	0	0	1	0	1
K22441 diamine N-acetyltransferase [EC:2.3.1.57]	225	182	188	186	61	66	88	65	440	463	440	453
K22443 carnitine monooxygenase subunit [EC:1.14.13.239]	441	392	430	467	131	141	91	89	757	877	851	952
K22444 carnitine monooxygenase subunit [EC:1.14.13.239]	342	357	310	368	99	103	90	88	724	717	700	763
K22452 protein-glutamine gamma-glutamyltransferase [EC:2.3.2.13]	2555	2479	2331	2436	1367	1238	1219	1351	772	723	640	727
K22465 5-hydroxybenzimidazole synthase [EC:4.1.99.23]	33	15	26	31	51	54	50	33	78	63	76	93
K22551 N-methyl-L-proline demethylase [EC:1.5.3.-]	0	0	0	0	0	0	1	0	1	0	1	1
K22579 rifampicin phosphotransferase [EC:2.7.9.6]	74	64	72	80	17	12	20	10	36	51	44	52
K22589 threo-3-hydroxy-L-aspartate ammonia-lyase [EC:4.3.1.16]	1546	1492	1398	1419	733	774	671	816	466	401	456	493
K22597 glucosylglycerate phosphorylase [EC:2.4.1.352]	280	231	243	227	90	54	104	76	224	226	197	218
K22616 ornithine lipid ester-linked acyl 2-hydroxylase [EC:1.14.11.58]	3	0	0	0	2	0	0	3	2	2	3	1
K22617 lyso-ornithine lipid O-acyltransferase [EC:2.3.1.270]	1232	1225	1100	1147	626	644	519	707	407	332	340	314
K22704 hopanoid C-3 methylase [EC:2.1.1.-]	4	11	9	9	3	0	3	8	12	9	10	9
K22721 inositol phosphorylceramide mannosyltransferase catalytic subunit [EC:2.4.1.-]	1	0	0	0	0	1	0	0	0	0	0	0
K22757 glutaminyl-peptide cyclotransferase [EC:2.3.2.5]	6	2	2	5	6	0	3	4	2	0	0	1
K22837 SAMP-activating enzyme [EC:2.7.7.100]	1	0	0	1	0	1	1	1	1	1	0	0
K22874 L-tyrosine isonitrile desaturase/decarboxylase [EC:1.14.20.9 1.14.20.10]	1210	1114	991	1065	569	529	489	630	324	298	351	346
K22906 phloretin hydrolase [EC:3.7.1.4]	7	7	11	7	0	0	3	2	11	6	7	8
K22927 cyclic-di-AMP phosphodiesterase [EC:3.1.4.59]	1506	1463	1428	1521	424	439	463	380	1292	1346	1280	1312
K22952 L-tyrosine isonitrile synthase [EC:4.1.99.24]	1014	954	870	963	472	449	443	550	275	275	248	281
K23078 menaquinone C8-methyltransferase [EC:2.1.1.350]	98	86	129	104	168	145	167	139	158	151	139	162
K23107 1-deoxyxylulose-5-phosphate synthase [EC:1.1.-.-]	1120	1163	1062	1238	532	645	647	563	1736	1869	1761	1775
K23110 (R)-phenyllactyl-CoA dehydratase alpha subunit [EC:4.2.1.-]	12	4	3	5	0	0	0	0	1	3	3	3
K23111 (R)-phenyllactyl-CoA dehydratase beta subunit [EC:4.2.1.-]	3	2	2	1	0	0	0	0	0	0	0	0
K23122 staphylopine dehydrogenase [EC:1.5.1.52]	1	0	0	0	0	0	0	0	0	0	0	0
K23147 aminoimidazole riboside kinase [EC:2.7.1.223]	14	18	17	15	0	0	0	0	7	14	6	8
K23148 cytidine diphosphoramidate kinase [EC:2.7.1.224]	0	0	0	0	2	0	3	3	5	0	3	4
K23149 5-phospho-D-xylono-1,4-lactonase [EC:3.1.1.104]	721	538	637	595	51	55	40	40	413	406	411	404
K23244 D-apiose dehydrogenase [EC:1.1.1.-]	0	0	0	1	0	2	2	1	4	3	1	4
K23245 D-apionate oxidoisomerase [EC:1.1.1.-]	49	35	37	27	7	7	5	2	78	90	84	71
K23246 D-apionate lactonase [EC:3.1.1.-]	2	0	0	0	0	3	5	2	4	5	4	3
K23247 3-oxo-isoapionate kinase [EC:2.7.1.-]	0	2	0	0	2	5	3	0	2	4	8	0
K23248 3-oxo-isoapionate decarboxylase [EC:4.1.1.-]	0	0	0	0	0	0	0	0	2	0	1	0
K23254 P-type Ag+ transporter [EC:7.2.2.15]	1406	1455	1422	1368	2061	2280	2102	2054	136	151	132	139
K23256 NADPH-dependent curcumin reductase [EC:1.3.1.-]	2926	2835	2619	2773	1537	1548	1491	1730	1431	1375	1420	1417
K23271 erythritol/L-threitol dehydrogenase [EC:1.1.1.-]	284	317	294	287	65	67	57	56	423	453	443	460
K23300 menaquinone reductase, multiheme cytochrome c subunit [EC:1.97.-.-]	6	4	4	1	0	2	3	0	2	1	2	0
K23301 menaquinone reductase, iron-sulfur cluster-binding subunit [EC:1.97.-.-]	3	5	6	6	0	0	0	2	0	2	2	1
K23463 (Z)-2-((N-methylformamido)methylene)-5-hydroxybutyrolactone dehydrogenase [EC:1.2.1.-]	0	0	0	0	0	0	0	0	1	0	1	0
K23518 O-acetyl-ADP-ribose deacetylase [EC:3.1.1.106]	1245	1351	1195	1330	787	816	851	777	871	771	839	835
ko99981 Carbohydrate metabolism	11010	11083	10880	11239	10813	10748	10565	10942	10698	10732	10570	11169
K00782 L-lactate dehydrogenase complex protein LldG	497	610	628	646	1531	1552	1497	1447	1043	934	1018	949
K02566 NagD protein	566	628	507	599	359	382	296	316	720	699	708	826
K04767 acetoin utilization protein AcuB	35	31	37	27	3	4	8	10	64	56	67	52
K06206 sugar fermentation stimulation protein A	1827	1807	1709	1809	894	908	888	922	1329	1388	1331	1447
K06605 myo-inositol catabolism protein IolH	425	414	357	401	105	83	93	93	710	779	770	823
K11184 catabolite repression HPr-like protein	71	80	82	82	19	15	13	30	82	72	77	77
K11930 periplasmic protein TorT	3	5	1	7	14	5	15	8	58	59	44	43
K17641 beta-xylosidase	0	0	0	0	0	0	0	0	0	1	0	0
K18928 L-lactate dehydrogenase complex protein LldE	633	646	727	714	1774	1785	1858	1781	1144	1196	1229	1207
K18929 L-lactate dehydrogenase complex protein LldF	1218	1283	1415	1334	3268	3389	3335	3391	2271	2088	2229	2231
K18930 D-lactate dehydrogenase	4663	4520	4436	4560	2455	2246	2166	2554	1450	1448	1307	1498
K19504 glucoselysine-6-phosphate deglycase	534	561	509	528	149	170	164	150	975	996	915	1101
K19510 fructoselysine-6-phosphate deglycase	535	490	468	532	236	189	216	221	805	967	812	861
K22111 9-O-acetyl-N-acetylneuraminic acid deacetylase	3	6	1	0	6	17	16	19	46	48	63	54
K22136 bacillithiol synthase	0	2	2	0	0	2	0	0	1	1	0	0
K22216 dehydratase ilvD1	0	0	1	0	1	0	0	0	0	0	0	0
ko99982 Energy metabolism	32087	31907	30917	31470	25007	25227	24480	25537	26157	26545	25641	26431
K00183 prokaryotic molybdopterin-containing oxidoreductase family, molybdopterin binding subunit	47	54	51	64	45	27	43	30	111	157	151	146
K00184 prokaryotic molybdopterin-containing oxidoreductase family, iron-sulfur binding subunit	54	44	41	24	41	25	22	23	90	118	77	82
K00185 prokaryotic molybdopterin-containing oxidoreductase family, membrane subunit	46	55	44	37	24	23	33	30	81	106	91	86
K02164 nitric oxide reductase NorE protein	956	849	809	873	508	497	459	520	343	281	294	275
K02379 FdhD protein	2163	2000	2080	2116	1271	1285	1243	1360	1223	1312	1297	1304
K02381 FdrA protein	49	51	51	41	45	29	37	32	156	180	187	192
K02448 nitric oxide reductase NorD protein	2748	2754	2487	2552	1662	1454	1497	1697	951	779	765	839
K02569 cytochrome c-type protein NapC	485	492	540	514	273	299	254	293	191	195	198	197
K02570 periplasmic nitrate reductase NapD	430	487	433	435	232	236	241	259	143	195	143	171
K02571 periplasmic nitrate reductase NapE	245	244	267	238	149	97	144	142	82	60	60	62
K02572 ferredoxin-type protein NapF	785	699	721	704	409	318	265	319	270	220	204	222
K02573 ferredoxin-type protein NapG	91	76	55	65	132	103	111	121	180	179	167	163
K02574 ferredoxin-type protein NapH	65	72	83	78	94	111	103	106	164	177	157	135
K02585 nitrogen fixation protein NifB	240	258	257	323	397	438	399	386	8	23	10	17
K02587 nitrogenase molybdenum-cofactor synthesis protein NifE	332	319	317	308	350	444	405	376	34	36	19	24
K02592 nitrogenase molybdenum-iron protein NifN	242	285	235	264	387	403	396	364	0	1	0	4
K02593 nitrogen fixation protein NifT	37	37	37	31	58	57	61	65	0	0	2	0
K02595 nitrogenase-stabilizing/protective protein	31	48	44	39	72	70	54	61	0	0	0	0
K02596 nitrogen fixation protein NifX	224	198	199	212	281	294	301	296	0	0	2	2
K02597 nitrogen fixation protein NifZ	96	88	65	80	128	96	114	98	1	0	0	0
K03521 electron transfer flavoprotein beta subunit	2888	2882	2826	2865	3078	3008	3178	3203	2425	2488	2183	2362
K03612 Na+-translocating ferredoxin:NAD+ oxidoreductase subunit G	1198	1201	1230	1233	1844	1753	1698	1635	1737	1588	1722	1750
K03613 Na+-translocating ferredoxin:NAD+ oxidoreductase subunit E	2284	2209	2139	2262	2028	2279	2037	2198	2026	2053	1965	2027
K03617 Na+-translocating ferredoxin:NAD+ oxidoreductase subunit A	1423	1509	1487	1414	1588	1696	1604	1723	1571	1552	1627	1471
K03839 flavodoxin I	1026	1055	1072	1014	2319	2327	2357	2386	1809	1753	1786	1793
K03840 flavodoxin II	397	392	368	414	230	269	227	253	519	640	512	594
K03855 ferredoxin like protein	55	76	81	59	96	114	115	91	24	24	20	35
K04013 cytochrome c-type protein NrfB	1	2	0	0	5	10	8	9	41	31	26	28

K04014 protein NrfC	53	41	40	46	107	101	93	110	117	128	151	155
K04015 protein NrfD	62	83	56	58	101	128	93	125	146	146	193	163
K04016 cytochrome c-type biogenesis protein NrfE	8	3	10	2	9	17	13	17	99	98	83	95
K04017 formate-dependent nitrite reductase complex subunit NrfF	0	3	0	1	6	4	4	3	14	16	14	39
K04018 formate-dependent nitrite reductase complex subunit NrfG	0	0	2	4	8	4	3	5	30	41	47	40
K04488 nitrogen fixation protein NifU and related proteins	1371	1245	1308	1302	569	719	593	635	850	1048	992	1011
K04747 nitric oxide reductase NorF protein	0	0	0	0	0	0	0	0	0	2	1	0
K04748 nitric oxide reductase NorQ protein	792	824	817	748	443	353	368	479	285	308	281	273
K04755 ferredoxin, 2Fe-2S	716	790	740	728	478	489	497	446	473	514	478	498
K05337 ferredoxin	25	27	25	29	25	27	17	17	20	37	14	28
K05524 ferredoxin	906	840	823	864	483	513	462	506	286	257	261	277
K05796 electron transport protein HydN	565	573	517	598	364	336	340	346	897	867	860	931
K06205 MioC protein	1006	959	934	967	546	593	611	559	607	639	550	585
K06862 energy-converting hydrogenase B subunit Q	0	0	0	0	0	0	0	0	1	1	5	2
K09165 dodecin	413	389	403	363	208	232	188	251	136	137	116	142
K11107 ferredoxin	160	178	155	179	122	117	95	102	216	236	202	213
K12143 hydrogenase-4 component H	126	121	111	122	178	225	173	162	5	7	2	5
K12262 cytochrome b561	1339	1296	1250	1196	844	868	895	902	470	428	482	499
K13795 citrate/tricarballylate utilization protein	1	4	0	0	2	0	0	0	0	0	3	0
K13796 tricarballylate dehydrogenase	2	1	2	0	1	0	3	2	0	0	0	1
K13819 NifU-like protein	188	163	171	155	217	220	235	201	6	13	11	10
K14086 ech hydrogenase subunit A	59	52	73	57	18	16	20	19	66	110	105	86
K14087 ech hydrogenase subunit B	12	17	17	13	3	5	7	6	12	15	8	7
K14088 ech hydrogenase subunit C	24	33	26	24	6	5	3	3	55	46	40	34
K14089 ech hydrogenase subunit D	22	19	26	17	5	4	5	4	34	44	46	31
K14090 ech hydrogenase subunit E	77	103	100	72	25	29	16	19	144	123	161	136
K14091 ech hydrogenase subunit F	7	5	6	7	1	1	5	9	9	18	11	5
K14092 energy-converting hydrogenase A subunit A	0	0	0	1	0	0	0	1	0	1	1	1
K14093 energy-converting hydrogenase A subunit B	0	0	0	0	0	0	0	0	1	2	3	8
K14094 energy-converting hydrogenase A subunit C	0	0	0	1	0	0	0	0	0	0	0	2
K14095 energy-converting hydrogenase A subunit D	0	0	0	1	0	0	0	0	2	2	1	3
K14096 energy-converting hydrogenase A subunit E	0	0	0	0	0	0	0	3	0	2	0	3
K14097 energy-converting hydrogenase A subunit F	0	0	0	0	0	0	0	0	0	3	1	4
K14098 energy-converting hydrogenase A subunit G	0	2	0	0	0	0	0	0	4	1	2	3
K14099 energy-converting hydrogenase A subunit H	0	3	0	0	0	0	0	2	10	5	1	4
K14100 energy-converting hydrogenase A subunit I	0	0	0	0	0	0	0	0	0	1	2	0
K14101 energy-converting hydrogenase A subunit J	0	2	0	0	2	0	2	0	2	1	3	13
K14102 energy-converting hydrogenase A subunit K	0	1	0	0	0	0	0	2	0	0	2	1
K14103 energy-converting hydrogenase A subunit L	0	1	0	0	0	0	2	0	0	0	2	1
K14104 energy-converting hydrogenase A subunit M	0	1	0	1	0	0	0	1	0	3	3	1
K14105 energy-converting hydrogenase A subunit N	0	0	0	1	0	0	0	0	4	7	3	6
K14106 energy-converting hydrogenase A subunit O	3	0	3	1	0	0	0	0	4	5	11	12
K14107 energy-converting hydrogenase A subunit P	0	0	2	1	1	0	0	0	0	5	5	9
K14108 energy-converting hydrogenase A subunit Q	2	0	1	3	2	0	0	0	2	17	8	7
K14109 energy-converting hydrogenase A subunit R	0	0	2	0	0	2	0	2	4	9	5	10
K14110 energy-converting hydrogenase B subunit A	0	0	0	0	0	0	0	1	3	5	0	4
K14111 energy-converting hydrogenase B subunit B	0	2	0	0	0	0	0	0	3	1	0	0
K14112 energy-converting hydrogenase B subunit C	0	0	0	1	0	1	0	3	2	1	1	0
K14113 energy-converting hydrogenase B subunit D	0	0	0	0	0	1	0	0	0	0	0	4
K14114 energy-converting hydrogenase B subunit E	0	0	0	0	0	0	0	2	0	4	3	2
K14115 energy-converting hydrogenase B subunit F	2	2	2	1	2	1	2	2	2	17	8	7
K14116 energy-converting hydrogenase B subunit G	0	0	2	0	0	0	0	1	0	0	0	1
K14117 energy-converting hydrogenase B subunit H	0	0	0	0	0	0	0	0	0	0	0	1
K14118 energy-converting hydrogenase B subunit I	0	0	0	1	0	0	0	0	4	1	3	4
K14119 energy-converting hydrogenase B subunit J	0	0	0	1	0	0	0	0	2	3	2	2
K14120 energy-converting hydrogenase B subunit K	0	2	0	1	0	2	1	2	3	10	8	13
K14121 energy-converting hydrogenase B subunit L	0	0	0	0	0	0	3	0	0	2	0	2
K14122 energy-converting hydrogenase B subunit M	0	0	0	0	0	0	1	2	0	0	0	1
K14123 energy-converting hydrogenase B subunit N	0	0	0	1	2	0	0	2	7	14	5	9
K14124 energy-converting hydrogenase B subunit O	0	0	2	0	0	4	0	0	5	7	2	3
K14125 energy-converting hydrogenase B subunit P	0	0	0	0	0	0	0	2	0	0	3	1
K15536 soluble cytochrome b562	240	268	242	240	159	196	140	165	321	328	318	350
K15790 nitrogen fixation protein NifQ	105	93	104	118	157	163	132	158	2	0	0	0
K15827 formate hydrogenlyase subunit 2	229	270	211	268	69	104	80	71	496	512	501	509
K15828 formate hydrogenlyase subunit 3	737	794	738	790	208	233	220	227	1608	1565	1468	1523
K15829 formate hydrogenlyase subunit 4	323	340	342	332	97	115	107	110	732	792	752	776
K15830 formate hydrogenlyase subunit 5	695	716	592	726	220	187	199	221	1385	1455	1428	1481
K15831 formate hydrogenlyase subunit 6	223	202	218	217	84	65	54	51	443	460	469	462
K15832 formate hydrogenlyase subunit 7	295	319	294	330	99	117	99	81	634	675	589	643
K16183 methylamine methyltransferase corrinoid activation protein	0	0	0	0	0	0	0	1	2	0	1	1
K16885 quinone-modifying oxidoreductase, subunit QmoA	7	12	8	3	3	4	6	0	13	13	24	15
K16886 quinone-modifying oxidoreductase, subunit QmoB	13	16	13	17	3	13	8	3	28	32	15	31
K16887 quinone-modifying oxidoreductase, subunit QmoC	4	9	12	7	4	4	2	1	12	17	11	19
K19344 cytochrome c55X	340	371	313	378	197	197	174	178	124	110	89	120
K19345 protein NirF	1962	1923	1797	1896	1074	963	993	1129	649	515	588	633
K19824 rubrerythrin	0	0	0	0	0	0	2	0	1	4	4	4
K21739 probable pyridine nucleotide-disulfide oxidoreductase	315	330	367	296	98	82	98	102	476	508	505	458
K23077 dissimilatory sulfite reductase related protein	0	5	1	1	2	0	4	0	0	2	3	9
K23302 menaquinone reductase, molybdopterin-binding-like subunit	14	6	7	17	6	5	3	4	2	1	5	5
K23303 menaquinone reductase, integral membrane subunit	6	12	13	7	3	0	3	3	7	0	1	3
ko99983 Lipid metabolism	5661	5491	5247	5338	3110	3156	2915	3363	2883	2833	2881	2938
K09456 putative acyl-CoA dehydrogenase	3808	3802	3586	3613	2055	2168	2037	2324	2255	2283	2330	2352
K11729 acyl-CoA dehydrogenase family member 10	3	5	2	1	2	0	1	1	1	2	3	2
K11730 acyl-CoA dehydrogenase family member 11	2	0	0	0	0	0	0	0	0	0	0	0
K16229 glycopeptidolipid biosynthesis protein	1	0	0	1	0	0	0	0	0	0	0	0
K21830 phosphorylcholine phosphatase	1847	1684	1659	1723	1053	988	877	1038	627	548	548	584
ko99984 Nucleotide metabolism	442	456	365	422	216	225	235	219	423	448	390	434
K03647 protein involved in ribonucleotide reduction	239	254	191	201	166	161	183	154	322	278	251	316
K15051 DNA-entry nuclease	203	202	174	221	50	64	52	65	101	170	139	118
ko99985 Amino acid metabolism	8786	8702	8725	8805	5157	5479	5434	5242	10754	11042	10603	11020
K04023 ethanolamine transporter	935	968	891	952	337	359	382	326	1344	1419	1261	1349
K04024 ethanolamine utilization protein EutJ	560	611	650	585	165	206	184	142	886	869	833	869
K04025 ethanolamine utilization protein EutK	188	199	198	209	45	68	52	59	348	369	394	418
K04026 ethanolamine utilization protein EutL	316	353	341	327	93	88	103	97	619	719	689	714
K04027 ethanolamine utilization protein EutM	174	134	149	152	46	38	35	41	255	236	230	328
K04028 ethanolamine utilization protein EutN	304	297	288	274	62	73	90	75	403	443	431	434
K04029 ethanolamine utilization protein EutP	560	568	488	563	256	290	278	289	821	811	880	863
K04030 ethanolamine utilization protein EutQ	476	410	511	471	108	108	139	106	751	688	755	758
K04031 ethanolamine utilization protein EutS	521	505	473	536	215	245	256	192	721	789	708	694
K05806 leu operon leader peptide	15	11	16	14	24	30	29	24	3	2	3	4
K06203 CysZ protein	992	1003	1048	1041	600	644	688	701	771	951	872	866
K06997 PLP dependent protein	2492	2416	2403	2443	2487	2564	2523	2429	2526	2434	2382	2419
K08234 glyoxylase I family protein	351	333	368	376	211	261	194	198	583	550	495	547
K10795 D-proline reductase (dithiol)-stabilizing protein PrdD	0	0	0	0	0	0	0	0	0	1	1	1
K10796 D-proline reductase (dithiol)-stabilizing protein PrdE	1	0	2	1	0	0	0	1	1	0	0	2
K11325 L-cysteine/cystine lyase	20	16	9	17	0	0	0	0	13	16	8	10
K14054 protein MpaA	407	366	419	407	248	253	227	295	568	605	525	565
K18802 glutamine amidotransferase	0	0	2	0	0	0	0	0	0	0	0	0
K21700 beta-alanine degradation protein BauB	474	512	469	437	260	252	254	267	141	140	136	179
ko99986 Glycan metabolism	10957	10977	10358	10400	6818	6824	6637	7029	4686	4649	4306	4691
K03208 colanic acid biosynthesis glycosyl transferase WcaI	283	249	249	272	387	377	428	416	72	61	66	74
K07270 glycosyl transferase, family 25	0	2	0	0	2	5	0	0	2	5	6	3
K08992 lipopolysaccharide assembly protein A	169	159	195	177	116	112	151	117	226	257	218	203
K13620 putative colanic acid polymerase	242	203	212	269	363	363	428	366	64	73	69	75
K16568 exopolysaccharide production protein ExoZ	0	0	0	3	0	0	0	2	0	1	4	2
K16707 UDP-galactose-lipid carrier transferase	0	0	0	0	0	0	0	0	1	1	1	0
K16710 colanic acid/amylovoran biosynthesis protein	254	275	254	211	352	415	369	384	61	69	55	53
K16711 colanic acid biosynthesis protein WcaM	237	293	303	287	407	458	414	406	46	68	63	66
K19292 alginate biosynthesis protein AlgK	2119	2251	1994	1996	1179	1054	1142	1199	686	570	620	666
K19293 alginate biosynthesis protein AlgX	2317	2281	2067	2044	1252	1245	1105	1257	657	649	566	716
K19294 alginate O-acetyltransferase complex protein AlgI	2905	2923	2859	2846	1401	1421	1227	1436	1339	1369	1214	1385

K19295 alginate O-acetyltransferase complex protein AlgJ	940	820	815	776	445	450	437	498	265	233	257	258
K19296 alginate O-acetyltransferase complex protein AlgF	828	795	720	811	414	407	400	444	249	237	231	227
K19421 polysaccharide biosynthesis protein EpsC	3	5	4	3	0	0	0	0	9	2	11	6
K19804 lipopolysaccharide assembly protein B	660	720	686	705	500	517	536	503	1009	1054	923	957
K21688 resuscitation-promoting factor RpfB	0	1	0	0	0	0	0	0	1	0	0	2	0
ko99987 Cofactor metabolism	9552	9579	9355	9836	7410	7885	7472	7714	8999	8926	8830	9030
K02173 putative kinase	8	5	6	9	9	9	18	7	37	40	35	46
K02498 HemY protein	1673	1694	1649	1721	1018	1073	956	1053	1411	1322	1310	1286
K02859 riboflavin biosynthesis RibT protein	0	0	0	1	2	5	3	2	4	11	3	4
K03688 ubiquinone biosynthesis protein	3437	3350	3343	3506	2011	2166	1986	2102	2506	2505	2440	2672
K03690 ubiquinone biosynthesis protein UbiJ	1501	1498	1442	1507	903	909	818	920	839	915	880	968
K03753 molybdopterin-guanine dinucleotide biosynthesis adapter protein	348	364	358	346	263	321	296	273	491	500	488	528
K12942 aminobenzoyl-glutamate transport protein	1792	1920	1830	2002	2818	3046	3053	2965	3486	3403	3416	3301
K13580 magnesium chelatase subunit ChlD-like protein	793	748	727	744	386	356	342	392	225	229	254	223
K18588 coenzyme Q-binding protein COQ10	0	0	0	0	0	0	0	0	0	1	4	2
ko99988 Secondary metabolism	8587	8248	7845	8055	5006	4968	4726	5012	5616	5568	5590	5798
K03932 polyhydroxybutyrate depolymerase	1713	1536	1419	1562	883	862	828	862	497	445	491	477
K04753 suppressor of ftsI	933	890	915	957	660	658	582	640	1174	1329	1272	1352
K06219 S-adenosylmethionine-dependent methyltransferase	1726	1709	1682	1670	1021	924	920	993	1251	1221	1243	1274
K14588 blue copper oxidase	888	899	861	929	657	704	667	687	1214	1270	1261	1256
K18004 HQNO biosynthesis monooxygenase PqsL	2094	2040	1780	1816	1056	1059	1014	1103	702	560	547	590
K19784 chromate reductase, NAD(P)H dehydrogenase (quinone)	1232	1174	1188	1121	729	759	715	727	778	743	776	849
K23376 staphyloferrin B biosynthesis citrate synthase	1	0	0	0	0	2	0	0	0	0	0	0
ko99999 Others	7232	7597	7270	7419	8553	8615	8619	8235	8972	8753	8903	9080
K06999 phospholipase/carboxylesterase	1532	1591	1478	1500	903	878	830	859	1010	991	977	1071
K09181 acetyltransferase	2728	2774	2786	2800	5107	5347	5341	4874	5500	5338	5534	5512
K15975 glyoxalase family protein	153	165	177	173	32	21	33	30	193	185	172	164
K15977 putative oxidoreductase	1315	1458	1401	1423	1623	1594	1601	1549	1785	1775	1745	1792
K21831 cholinesterase	1504	1609	1428	1523	888	775	814	923	484	464	475	541
Unclassified: genetic information processing	125734	126742	126387	127572	140283	141546	129488	132441	130762	129906	130795	129454
ko99973 Transcription	15183	15122	15143	15212	12004	12068	11761	11714	13002	12850	12937	13005
K02466 glucitol operon activator protein	236	226	205	275	156	200	206	168	357	356	309	341
K02589 nitrogen regulatory protein PII 1	89	64	65	85	11	14	16	6	128	145	157	146
K02590 nitrogen regulatory protein PII 2	70	65	64	60	12	3	12	11	124	133	118	120
K03622 archaea-specific DNA-binding protein	0	0	0	0	0	0	0	0	1	2	2	1
K03623 ribonuclease inhibitor	191	147	160	157	127	136	125	115	243	241	245	246
K03627 putative transcription factor	0	0	0	0	0	0	0	0	3	2	0	4
K03704 cold shock protein	3198	3242	3230	3209	1739	1778	1720	1675	2136	2203	2145	2261
K04752 nitrogen regulatory protein P-II 2	828	845	826	839	528	577	496	553	523	542	480	490
K04796 small nuclear ribonucleoprotein	0	0	0	0	0	0	0	0	0	2	5	1
K06959 protein Tex	8174	8079	8125	8135	8233	8177	8038	7973	7530	7357	7519	7467
K07591 threonine dehydratase operon activator protein	2	2	1	2	2	5	3	7	22	21	23	28
K07740 regulator of sigma D	1251	1183	1199	1257	773	743	723	791	795	742	748	757
K07741 anti-repressor protein	780	846	914	817	270	292	289	314	313	294	326	314
K07744 transcriptional regulator	0	5	0	1	0	0	3	0	6	0	3	6
K15833 formate hydrogenlyase regulatory protein HycA	199	213	188	187	80	63	59	56	422	392	452	434
K15834 formate hydrogenlyase maturation protein HycH	143	174	146	162	53	63	45	39	364	371	359	353
K16509 regulatory protein spx	22	31	20	26	20	17	26	6	35	47	46	36
ko99974 Translation	7135	7095	7049	7456	3574	3570	3155	3681	4750	4677	4701	4977
K07570 general stress protein 13	30	30	21	23	4	12	4	4	39	29	24	32
K07571 S1 RNA binding domain protein	43	51	79	56	26	25	42	29	46	58	54	79
K07572 putative nucleotide binding protein	0	0	0	0	0	0	0	0	1	1	1	6
K07575 PUA domain protein	0	0	0	0	1	0	0	0	2	5	2	7
K07576 metallo-beta-lactamase family protein	1451	1491	1410	1539	626	593	498	581	966	955	944	1033
K07577 putative mRNA 3-end processing factor	1	0	2	3	2	1	0	0	4	3	0	4
K07579 putative methylase	0	0	1	0	2	2	2	0	0	4	3	4
K07580 Zn-ribbon RNA-binding protein	0	0	0	1	0	0	0	0	0	0	0	1
K07581 RNA-binding protein	0	0	0	0	0	2	2	0	2	0	3	1
K07585 tRNA methyltransferase	0	0	0	0	0	1	0	2	0	5	2	4
K13926 ribosome-dependent ATPase	5610	5523	5536	5834	2913	2934	2607	3065	3690	3617	3668	3806
ko99975 Protein processing	39934	39715	38004	39530	28186	28122	27285	28845	30048	30436	29983	30432
K02380 FdhE protein	2172	2080	1804	2003	1326	1295	1121	1346	1298	1401	1301	1340
K03187 urease accessory protein	837	860	692	825	338	380	318	348	713	699	649	694
K03188 urease accessory protein	704	640	628	695	229	278	225	260	825	846	772	878
K03189 urease accessory protein	753	712	732	763	332	340	290	326	732	745	699	716
K03190 urease accessory protein	1661	1691	1567	1561	777	772	718	885	1071	1095	1133	1147
K03192 urease accessory protein	1014	927	789	829	468	462	464	566	283	248	216	294
K03545 trigger factor	2970	2978	3203	3027	4323	4187	4149	4091	4512	4673	4584	4511
K03664 SsrA-binding protein	1445	1452	1504	1503	1531	1572	1577	1569	1572	1574	1558	1503
K03668 heat shock protein HslJ	875	982	892	927	527	572	523	563	550	579	600	549
K04063 lipoyl-dependent peroxidase	1865	1814	1716	1835	1299	1194	1143	1380	847	847	806	840
K04653 hydrogenase expression/formation protein HypC	240	207	163	217	200	198	220	219	208	212	198	215
K04654 hydrogenase expression/formation protein HypD	892	832	798	831	569	633	633	666	1122	1180	1156	1184
K04655 hydrogenase expression/formation protein HypE	784	722	642	805	518	555	538	563	1014	1010	1103	987
K04656 hydrogenase maturation protein HypF	1584	1610	1452	1601	1084	1150	1095	1064	2073	2184	2055	2228
K06191 glutaredoxin-like protein NrdH	193	161	172	184	111	114	134	108	203	238	200	215
K06891 ATP-dependent Clp protease adaptor protein ClpS	603	555	572	559	322	364	291	325	388	411	457	386
K07388 hydrogenase expression/formation protein	0	3	0	1	0	0	2	0	4	11	8	10
K07391 magnesium chelatase family protein	5000	5358	4996	5242	5169	5203	5196	5379	4843	4585	4800	4818
K07392 AAA family ATPase	0	3	1	0	1	0	0	0	4	8	3	5
K07394 SM-20-related protein	998	922	829	974	509	527	517	546	292	318	280	329
K07395 putative proteasome-type protease	1543	1441	1519	1431	868	914	816	836	829	829	859	849
K07396 putative protein-disulfide isomerase	2293	2330	2132	2287	1385	1337	1298	1461	660	599	680	666
K07397 putative redox protein	1619	1547	1626	1695	991	939	889	868	769	743	659	765
K07399 cytochrome c biogenesis protein	2	0	1	0	0	0	0	0	0	0	0	0
K07400 Fe/S biogenesis protein NfuA	1065	997	1079	1079	636	645	617	643	702	724	722	684
K07401 selenoprotein W-related protein	614	674	593	615	369	337	315	372	423	434	361	426
K07402 xanthine dehydrogenase accessory factor	3681	3781	3636	3683	1946	1816	1901	1966	1680	1816	1688	1680
K07403 membrane-bound serine protease (ClpP class)	2182	2230	1918	2020	1090	1063	1109	1218	687	649	611	686
K11208 GST-like protein	303	319	329	326	239	259	187	275	469	520	442	479
K16511 adapter protein MecA 1/2	453	403	473	413	110	97	112	95	287	344	357	358
K17247 methionine sulfoxide reductase heme-binding subunit	1366	1285	1346	1388	885	879	847	877	843	792	897	854
K19411 protein arginine kinase activator	223	199	200	211	34	40	40	30	145	122	129	136
ko99976 Replication and repair	62956	64271	65678	64812	96117	97350	86849	87782	82278	81169	82483	80293
K01146 protein Xni	334	361	356	381	300	312	326	338	407	493	441	493
K02086 DNA replication protein	40	49	39	37	4	5	14	12	30	61	44	37
K03630 DNA repair protein RadC	4666	4671	4599	4662	4330	4426	4258	4192	3804	3836	3747	3607
K04096 DNA processing protein	2482	2663	2427	2633	3419	3454	3585	3557	3576	3545	3314	3431
K06909 phage terminase large subunit	536	440	546	478	118	114	99	108	272	337	260	304
K06919 putative DNA primase/helicase	1894	1913	1949	1919	1590	1725	1574	1701	1041	1162	1199	1186
K06993 ribonuclease H-related protein	8	17	15	21	3	7	7	4	6	18	4	16
K07357 type 1 fimbriae regulatory protein FimB	254	246	275	300	84	90	82	72	529	596	537	575
K07358 type 1 fimbriae regulatory protein FimE	227	214	209	241	75	66	61	89	465	416	437	498
K07443 methylated-DNA-protein-cysteine methyltransferase related protein	1287	1315	1188	1313	1287	1216	1235	1229	1216	1113	1262	1080
K07445 putative DNA methylase	0	0	0	1	0	0	0	0	1	4	3	4
K07449 similar to archaeal holliday junction resolvase and Mrr protein	0	0	1	0	0	0	0	0	0	0	3	0
K07453 putative restriction endonuclease	1215	1177	1202	1184	694	661	601	673	387	370	394	401
K07454 putative restriction endonuclease	354	343	373	350	111	114	91	90	677	768	678	705
K07457 endonuclease III related protein	2	3	2	1	0	5	3	6	4	8	8	9
K07459 putative ATP-dependent endonuclease of the OLD family	1383	1211	1275	1304	694	761	765	799	1497	1557	1409	1602
K07460 putative endonuclease	1206	1125	1211	1149	1332	1438	1353	1419	1124	1133	1113	1116
K07461 putative endonuclease	843	785	809	835	449	476	459	444	594	574	572	611
K07463 archaea-specific RecJ-like exonuclease	2	3	3	3	0	2	0	0	13	19	3	15
K07467 phage replication initiation protein	242	220	232	278	382	373	409	335	531	554	499	477
K07470 DNA gyrase inhibitor	258	264	228	245	168	210	217	206	353	383	321	366
K07474 phage terminase small subunit	1043	1006	1024	1065	543	507	572	553	408	444	431	470
K07478 putative ATPase	4668	4590	4371	4582	4368	4351	4535	4295	4674	4577	4635	4600
K07479 putative DNA topoisomerase	334	354	329	401	262	268	271	261	507	506	517	475
K07480 insertion element IS1 protein InsB	487	520	510	484	713	762	730	666	63	88	83	95
K07481 transposase, IS5 family	788	886	856	843	941	965	997	999	213	243	228	203
K07482 transposase, IS30 family	0	3	1	1	2	3	3	2	1	0	0	4

K07483 transposase	1577	1561	1677	1676	818	859	681	641	1312	1512	1355	1444
K07484 transposase	15019	16486	17586	16296	56980	57237	48110	48958	44299	42213	44864	41872
K07486 transposase	0	0	2	1	6	5	5	8	4	3	10	9
K07487 transposase	617	667	655	633	424	384	362	363	224	259	211	184
K07488 transposase	0	2	2	0	1	2	0	0	0	3	2	1
K07489 transposase	0	0	0	0	0	0	0	0	0	0	1	0
K07491 putative transposase	1064	1027	1017	1033	950	1033	943	1003	653	664	627	668
K07492 putative transposase	1	1	2	1	0	0	0	0	0	1	3	4
K07493 putative transposase	527	489	571	508	401	459	434	422	314	325	303	313
K07495 putative transposase	2	2	2	1	3	3	5	7	56	49	51	58
K07496 putative transposase	179	191	202	223	355	344	323	357	315	316	302	310
K07497 putative transposase	12498	12627	12716	12697	7720	8092	7462	7500	8336	8500	8214	8653
K07498 putative transposase	366	353	339	420	119	129	119	154	181	209	188	207
K07499 putative transposase	0	0	0	0	0	2	0	0	0	0	0	0
K07501 3'-5' exonuclease	0	2	0	1	2	0	1	0	1	0	0	0
K07505 regulatory protein RepA	1351	1385	1400	1341	783	816	764	754	458	483	506	506
K09760 DNA recombination protein RmuC	3554	3555	3754	3548	3958	3876	3718	3887	3297	3347	3239	3245
K10762 putative replication protein	0	0	0	1	0	2	1	0	2	0	5	0
K14059 integrase	1087	1011	1141	1152	904	911	932	907	275	302	305	278
K14060 putative DNA-invertase from lambdoid prophage Rac	182	202	211	235	284	339	277	280	112	129	128	124
K18320 transposase, IS6 family	379	331	371	334	540	546	465	491	46	49	27	37
ko99998 Others	526	539	513	562	402	436	438	419	684	774	691	747
K03969 phage shock protein A	335	356	357	411	241	280	262	221	450	570	501	533
K03971 phage shock protein D	135	112	103	110	75	76	76	86	176	158	156	184
K03972 phage shock protein E	53	69	51	41	80	66	89	98	30	23	11	10
K07504 predicted type IV restriction endonuclease	3	2	2	0	6	14	11	14	28	23	23	20
Unclassified: signaling and cellular processes	237972	237817	236931	239334	238163	240445	239553	240111	237762	238299	236919	238552
ko99977 Transport	97253	97592	97859	98594	107521	108423	108177	108675	107036	107130	106473	107028
K03281 chloride channel protein, ClC family	2389	2473	2551	2613	4587	4688	4631	4558	4831	4866	4806	4956
K03285 general bacterial porin, GBP family	31	28	34	33	46	56	43	44	46	66	64	54
K03292 glycoside/pentoside/hexuronide:cation symporter, GPH family	7894	8058	8396	8358	8592	9099	8797	8597	13208	13552	13427	13196
K03293 amino acid transporter, AAT family	3346	3279	3096	3410	1867	1934	1726	1963	2428	2552	2647	2497
K03294 basic amino acid/polyamine antiporter, APA family	2309	2495	2352	2391	1454	1515	1500	1503	1320	1367	1325	1432
K03296 hydrophobic/amphiphilic exporter-1 (mainly G- bacteria), HAE1 family	3315	3456	3722	3497	9140	9093	9294	9315	5694	5772	5707	5439
K03300 citrate-Mg2+:H+ or citrate-Ca2+:H+ symporter, CitMHS family	2168	2078	2159	2061	1151	1119	1029	1164	766	729	731	753
K03305 proton-dependent oligopeptide transporter, POT family	5195	5228	5336	5291	7097	7263	7464	7170	10519	10263	10203	10254
K03306 inorganic phosphate transporter, PiT family	5724	5602	5499	5631	4676	4763	4620	4842	3495	3168	3081	3184
K03307 solute:Na+ symporter, SSS family	9003	9079	8979	9136	12466	12498	12483	12230	10757	10607	10939	10700
K03308 neurotransmitter:Na+ symporter, NSS family	3965	3734	4017	3746	4022	4257	4481	4144	4913	5446	5236	5167
K03309 dicarboxylate/amino acid:cation (Na+ or H+) symporter, DAACS family	22	19	19	23	22	39	26	34	28	23	28	15
K03310 alanine or glycine:cation symporter, AGCS family	6660	6501	6504	6645	5871	5962	6067	5992	6675	6661	6599	6680
K03311 branched-chain amino acid:cation transporter, LIVCS family	3118	3088	2967	3142	1720	1764	1567	1789	2435	2448	2345	2580
K03316 monovalent cation:H+ antiporter, CPA1 family	5462	5420	5095	5649	3004	2861	2871	3195	4006	4030	3889	4015
K03317 concentrative nucleoside transporter, CNT family	1160	1091	1124	1086	564	623	576	602	1876	1760	1705	1983
K03319 divalent anion:Na+ symporter, DASS family	818	787	847	878	1053	1083	1074	1064	288	260	261	294
K03328 polysaccharide transporter, PST family	188	192	184	190	252	289	251	259	315	309	290	333
K03451 betaine/carnitine transporter, BCCT family	1198	1255	1185	1210	359	332	326	289	1405	1490	1489	1495
K03453 bile acid:Na+ symporter, BASS family	4057	4076	4073	4018	3819	3688	3608	3655	3078	2922	2950	2960
K03455 monovalent cation:H+ antiporter-2, CPA2 family	9837	9785	9947	9857	12870	12586	13185	13218	9703	9674	9497	9565
K03457 nucleobase:cation symporter-1, NCS1 family	8710	9204	8630	8750	5076	4901	4541	4979	4747	4393	4340	4614
K03458 nucleobase:cation symporter-2, NCS2 family	2371	2164	2202	2181	1162	1156	1102	1189	751	792	812	818
K07011 rhamnosyltransferase	0	0	0	0	1	5	0	0	0	0	0	1
K07085 putative transport protein	6241	6432	6970	6679	15436	15624	15869	15653	12419	12503	12752	12518
K21990 formate-nitrite transporter family protein	1883	1889	1790	1941	1115	1084	909	1103	1081	1171	1123	1249
K23243 nickel/cobalt homeostasis protein	189	179	181	178	99	141	137	124	252	306	227	276
ko99978 Cell growth	16189	16041	15938	16131	8820	9372	8971	8371	14171	15347	14813	15262
K06190 intracellular septation protein	1044	945	951	1038	600	718	579	580	560	559	601	578
K06287 septum formation protein	2035	2116	2163	2066	2157	2254	2200	2104	2116	2134	2020	2137
K06295 spore germination protein KA	32	19	15	37	9	3	10	4	21	24	25	27
K06297 spore germination protein KC	5	3	2	5	2	0	4	2	3	9	3	4
K06298 germination protein M	66	94	76	84	40	40	40	42	85	155	100	128
K06305 spore germination protein Q	0	0	0	0	0	0	0	0	0	1	0	0
K06306 spore germination protein	667	677	671	673	144	157	139	135	408	510	532	520
K06308 spore germination protein	3	2	0	0	0	0	0	0	0	0	0	0
K06310 spore germination protein	70	58	58	66	3	6	2	0	204	187	157	211
K06313 spore germination protein	2	0	0	0	0	0	0	0	0	0	0	0
K06317 inhibitor of the pro-sigma K processing machinery	18	12	15	14	8	16	13	5	26	18	43	27
K06320 spore maturation protein CgeB	6	16	8	11	1	5	6	3	15	41	29	21
K06327 spore coat protein D	0	0	0	0	0	0	0	1	0	0	0	0
K06330 spore coat protein H	8	6	7	12	8	10	3	3	5	5	8	6
K06333 spore coat protein JB	282	276	237	261	38	87	95	49	230	251	238	291
K06334 spore coat protein JC	527	502	495	530	132	142	158	117	557	602	570	555
K06338 spore coat protein SA	19	20	25	22	57	76	91	77	45	51	52	71
K06344 spore coat protein Z	3	0	0	0	0	0	0	2	0	0	0	0
K06346 spoIIIJ-associated protein	499	536	572	518	122	183	175	146	443	512	500	530
K06348 sporulation inhibitor KapD	11	13	13	19	0	0	0	0	20	28	38	26
K06349 KinB signaling pathway activation protein	0	0	0	0	0	0	0	2	0	0	0	0
K06350 antagonist of KipI	1	2	0	1	8	9	7	10	2	2	0	1
K06351 inhibitor of KinA	0	2	0	0	0	0	1	0	0	1	0	1
K06373 spore maturation protein A	342	333	346	310	106	111	82	98	276	312	301	308
K06374 spore maturation protein B	359	279	283	308	95	106	108	82	221	273	253	270
K06378 stage II sporulation protein AA (anti-sigma F factor antagonist)	153	129	144	142	46	59	50	39	124	97	93	138
K06381 stage II sporulation protein D	628	586	554	574	162	196	209	168	563	560	526	574
K06384 stage II sporulation protein M	140	108	111	128	68	72	57	33	109	113	141	132
K06385 stage II sporulation protein P	373	386	347	372	109	138	130	131	255	357	356	293
K06386 stage II sporulation protein Q	0	1	0	1	0	0	0	0	2	0	0	1
K06387 stage II sporulation protein R	306	296	262	308	62	64	81	71	263	276	254	251
K06390 stage III sporulation protein AA	526	480	456	519	134	140	124	122	347	346	377	369
K06391 stage III sporulation protein AB	200	193	171	168	48	40	48	48	129	162	151	179
K06392 stage III sporulation protein AC	86	87	90	90	28	24	34	22	71	80	52	81
K06393 stage III sporulation protein AD	188	165	177	177	46	67	46	30	152	167	140	147
K06394 stage III sporulation protein AE	444	410	443	392	118	111	101	122	299	316	348	353
K06395 stage III sporulation protein AF	162	132	174	183	42	41	56	38	100	107	121	125
K06396 stage III sporulation protein AG	220	199	199	214	44	71	67	54	159	153	129	133
K06397 stage III sporulation protein AH	343	317	306	312	81	91	84	64	264	237	255	261
K06398 stage IV sporulation protein A	642	667	741	704	217	219	231	203	565	756	579	630
K06400 site-specific DNA recombinase	83	93	83	91	113	123	129	113	35	68	63	71
K06403 stage V sporulation protein AA	238	249	259	276	28	65	57	42	239	215	246	270
K06404 stage V sporulation protein AB	146	169	161	127	35	46	29	29	116	135	126	141
K06405 stage V sporulation protein AC	213	226	201	200	91	81	69	74	177	219	199	198
K06406 stage V sporulation protein AD	431	493	480	463	176	149	149	135	470	519	480	478
K06407 stage V sporulation protein AE	193	169	204	170	71	47	69	53	152	156	148	107
K06408 stage V sporulation protein AF	597	648	619	595	172	185	212	152	344	444	355	353
K06410 dipicolinate synthase subunit A	258	263	248	269	50	41	38	39	206	230	220	230
K06411 dipicolinate synthase subunit B	197	204	205	238	49	59	41	49	158	159	226	194
K06412 stage V sporulation protein G	233	256	237	262	110	96	105	81	144	167	218	178
K06413 stage V sporulation protein K	0	0	0	0	0	0	0	0	0	1	0	0
K06415 stage V sporulation protein R	30	30	26	26	23	33	29	29	41	55	50	44
K06416 stage V sporulation protein S	6	5	5	4	2	2	3	0	8	8	4	7
K06421 small acid-soluble spore protein D (minor alpha/beta-type SASP)	0	0	0	0	0	1	0	0	0	0	0	0
K06436 spore coat assemly protein	3	5	9	0	0	0	2	0	4	8	5	11
K06438 similar to stage IV sporulation protein	416	362	395	404	156	144	146	123	331	374	389	417
K07257 spore coat polysaccharide biosynthesis protein SpsF	8	14	5	13	6	6	5	0	11	17	14	22
K07321 CO dehydrogenase maturation factor	125	143	115	88	85	48	88	77	283	269	284	307
K07322 regulator of cell morphogenesis and NO signaling	977	1009	1051	978	1856	1867	1798	1835	1518	1506	1484	1528
K15722 cell division activator	132	159	112	128	102	98	119	102	178	226	199	208
K20073 mid-cell-anchored protein Z	18	22	19	15	6	2	3	7	14	22	19	13
K22304 cell division inhibition protein DicB	1	0	1	0	1	3	3	3	10	7	7	6
K22718 divisome-associated membrane protein	64	65	59	54	41	63	70	57	82	84	97	88
K22719 murein hydrolase activator	1410	1390	1332	1471	912	957	776	764	1011	1056	988	1012
ko99993 Cell motility	4395	4287	4261	4313	2734	2849	2734	2733	3810	3953	3754	3855

K07335 basic membrane protein A and related proteins	1860	1729	1839	1820	709	733	644	635	1445	1601	1582	1516
K07337 penicillin-binding protein activator	304	290	303	316	197	238	200	267	406	418	358	423
K07343 DNA transformation protein and related proteins	454	433	435	462	282	296	302	269	589	611	550	544
K08086 pilus assembly protein FimV	474	488	435	432	263	274	215	253	193	170	177	173
K08087 fimbrial protein FimW	0	0	0	0	0	2	2	2	1	0	0	0
K15540 chaperone protein EcpD	882	901	868	859	951	990	994	984	650	644	639	667
K15723 SecY interacting protein Syd	312	295	260	289	230	211	261	231	348	332	321	370
K22720 surface composition regulator	109	151	121	135	102	105	116	92	178	177	127	162
ko99995 Signaling proteins	38993	38110	38088	38920	30353	30602	30246	30102	32205	32468	32201	33127
K03607 ProP effector	513	461	466	521	259	342	284	282	439	469	440	488
K03807 AmpE protein	1961	1924	1860	1857	1043	1095	1120	1090	1138	1089	1140	1254
K05791 tellurium resistance protein TerZ	9	5	4	7	3	5	1	3	0	0	0	1
K05792 tellurite resistance protein TerA	0	0	0	0	0	1	3	3	2	0	0	1
K05795 tellurium resistance protein TerD	4	4	12	6	47	35	51	35	1	1	2	0
K06149 universal stress protein A	923	826	961	954	527	656	568	539	574	548	606	559
K06200 carbon starvation protein	6063	5852	5753	5863	3291	3241	3277	3396	3986	3996	4005	4130
K06207 GTP-binding protein	6026	6126	6272	6245	7201	7211	7165	6793	6744	6871	7093	6948
K06217 phosphate starvation-inducible protein PhoH and related proteins	4141	4035	4046	4045	3983	4084	3992	3952	4164	4152	4159	4200
K06875 programmed cell death protein 5	0	0	0	1	1	0	0	0	0	0	1	1
K07166 ACT domain-containing protein	184	131	163	145	47	60	42	43	142	217	166	180
K07167 putative transcriptional regulator	0	1	0	0	0	0	0	0	0	2	4	0
K07168 CBS domain-containing membrane protein	1851	1822	1713	1821	910	936	883	903	1087	974	922	1088
K07169 FHA domain-containing protein	345	316	292	335	484	555	526	470	0	2	0	0
K07175 PhoH-like ATPase	2388	2385	2391	2450	3515	3369	3430	3512	2674	2624	2659	2642
K07180 serine protein kinase	3751	3493	3811	3952	2267	2278	2234	2294	2407	2413	2358	2366
K07182 CBS domain-containing protein	2864	2895	2684	2751	1547	1377	1467	1485	873	823	823	864
K07184 SH3 domain protein	1234	1128	1195	1117	728	719	721	751	696	723	649	706
K07186 membrane protein	366	342	369	390	284	286	278	263	466	542	529	647
K07212 GGDEF domain	7	6	6	9	0	0	0	0	4	4	6	5
K09767 cyclic-di-GMP-binding protein	1093	1175	1105	1148	661	602	623	621	744	778	730	827
K11932 universal stress protein G	539	512	481	536	288	330	261	303	862	960	891	911
K13630 multiple antibiotic resistance protein MarB	122	104	90	118	67	80	72	72	156	181	115	150
K14055 universal stress protein E	1593	1492	1460	1486	948	965	881	936	1019	976	941	974
K14061 universal stress protein F	253	238	190	245	159	152	171	157	300	289	281	292
K14064 universal stress protein C	233	243	264	285	167	181	158	181	407	402	440	378
K14065 universal stress protein D	2	1	2	2	3	2	9	4	31	28	26	39
K19336 cyclic-di-GMP-binding biofilm dispersal mediator protein	271	279	224	270	68	94	70	75	529	569	541	549
K21637 anti-adapter protein IraD	1	1	2	0	3	12	6	2	27	18	15	29
K21638 anti-adapter protein IraM	111	127	96	128	43	32	40	36	206	247	241	257
K21973 blue light- and temperature-responsive anti-repressor	1281	1373	1297	1309	913	989	978	996	1703	1827	1708	1882
K21974 probable RcsB/C two-component-system connector	260	221	246	223	206	226	229	254	264	223	232	224
K21975 probable RcsB/C two-component-system connector	302	301	330	349	358	372	342	337	318	293	283	314
K21976 probable RcsB/C two-component-system connector, global regulator of biofilm formation and acid-resistance	302	291	303	352	332	315	364	314	242	227	195	221
ko99992 Structural proteins	50112	50621	50511	50748	65251	65764	65890	66423	57775	56668	57542	56604
K01993 HlyD family secretion protein	4068	4114	4110	4006	6672	6846	7012	6995	6564	6174	6150	6292
K02005 HlyD family secretion protein	3239	3547	3669	3415	7825	7915	7704	7896	5876	5582	6107	5677
K02022 HlyD family secretion protein	7	12	4	19	0	2	2	3	7	8	3	4
K02197 cytochrome c-type biogenesis protein CcmE	826	842	800	913	393	420	436	448	540	563	579	587
K02200 cytochrome c-type biogenesis protein CcmH	2638	2614	2337	2404	1348	1314	1337	1683	1264	1186	1175	1201
K02494 outer membrane lipoprotein LolB	1471	1396	1301	1438	842	849	794	836	758	792	781	832
K02497 HemX protein	1	0	0	0	0	0	0	0	0	0	0	0
K03634 outer membrane lipoprotein carrier protein	1189	1205	1162	1172	649	665	596	625	697	769	696	787
K03642 rare lipoprotein A	1956	1882	1878	1997	1173	1198	1085	1188	1195	1146	1129	1193
K04062 osmotically inducible lipoprotein OsmB	100	85	86	96	73	87	65	95	144	130	114	128
K04064 osmotically inducible lipoprotein OsmE	744	768	713	734	439	454	425	465	444	476	464	450
K04754 phospholipid-binding lipoprotein MlaA	2299	2256	2364	2307	1442	1349	1401	1296	1286	1313	1267	1386
K05790 lipopolysaccharide biosynthesis protein WzzE	729	634	640	733	514	560	490	530	990	942	915	885
K05803 lipoprotein NlpI	555	539	498	570	408	458	416	405	728	744	758	732
K06077 outer membrane lipoprotein SlyB	723	688	631	675	384	370	331	411	394	381	378	359
K06142 outer membrane protein	2185	2253	2349	2266	4500	4412	4234	4280	3277	3374	3235	3211
K06143 inner membrane protein	3610	3693	3747	3633	4036	3886	4180	4077	3438	3348	3473	3370
K06194 lipoprotein NlpD	1730	1645	1652	1723	1014	1160	1127	1217	1155	1196	1112	1114
K07265 capsular polysaccharide export protein	240	252	266	278	436	516	345	414	71	57	41	52
K07266 capsular polysaccharide export protein	459	456	481	465	617	765	708	622	68	81	75	90
K07273 lysozyme	704	727	736	747	1930	1920	2065	1927	1585	1527	1580	1609
K07275 outer membrane protein	616	542	519	595	402	370	355	349	604	550	524	568
K07282 gamma-polyglutamate biosynthesis protein CapA	0	0	0	0	0	0	0	0	1	0	0	0
K07283 putative salt-induced outer membrane protein	351	406	457	438	337	319	303	273	530	553	487	589
K07285 outer membrane lipoprotein	293	359	357	321	224	286	246	254	460	508	495	558
K07286 uncharacterized lipoprotein	384	410	424	388	265	301	296	274	573	512	566	556
K07289 AsmA protein	4345	4371	4273	4353	2682	2649	2653	2824	2617	2651	2578	2651
K07290 AsmA family protein	4582	4537	4297	4701	2768	2800	2764	2952	2818	2772	2781	2816
K07340 inner membrane protein	1051	1037	876	987	643	581	588	567	512	534	571	514
K11744 AI-2 transport protein TqsA	357	372	361	353	132	116	118	137	778	769	760	829
K12943 lipoprotein YgeR	976	975	884	955	581	651	543	603	804	705	769	774
K13665 pyruvyltransferase	0	0	3	0	0	0	0	0	0	0	0	0
K15539 cytoskeleton protein RodZ	1522	1413	1424	1601	920	903	1009	1011	870	904	774	933
K19061 macrolide resistance protein	0	0	0	0	3	1	0	3	1	0	3	0
K19525 vacuolar protein sorting-associated protein 13A/C	0	0	0	0	0	0	0	0	0	2	1	0
K21571 starch-binding outer membrane protein SusE/F	5804	6228	6844	6136	21425	21440	22049	21602	16075	15701	16460	15149
K21695 protein AaeX	134	134	151	124	101	122	123	102	176	256	239	200
K21908 membrane protein HdeD	221	227	216	203	73	79	90	58	475	462	502	508
K22116 gamma-polyglutamate biosynthesis protein CapC	3	2	1	2	0	0	0	1	0	0	0	0
ko99994 Others	26899	26847	26169	26270	18478	18344	18317	18616	17515	17444	17123	17624
K02039 phosphate transport system protein	2262	2331	2315	2307	2363	2348	2459	2373	2513	2456	2518	2494
K02192 bacterioferritin-associated ferredoxin	478	450	473	467	293	276	268	270	297	304	268	276
K02255 ferritin-like protein 2	280	259	274	241	197	177	197	180	410	464	412	415
K03670 periplasmic glucans biosynthesis protein	858	822	853	857	600	679	602	559	1157	1266	1137	1298
K03926 periplasmic divalent cation tolerance protein	185	174	197	224	144	120	141	138	266	245	256	241
K05781 putative phosphonate transport system ATP-binding protein	1039	1026	915	1040	420	386	431	431	1053	1039	935	978
K05794 tellurite resistance protein TerC	1400	1384	1395	1287	818	787	748	786	430	433	382	406
K06039 uncharacterized protein involved in oxidation of intracellular sulfur	161	196	172	187	139	124	136	112	248	268	220	242
K06195 ApaG protein	823	777	796	815	482	551	517	517	469	523	493	461
K06201 copper homeostasis protein	1104	1116	998	1071	1671	1698	1760	1718	1698	1611	1865	1662
K06202 CyaY protein	600	695	600	590	409	418	421	431	420	428	450	503
K07214 enterochelin esterase and related enzymes	1910	1905	1857	1960	1709	1746	1687	1770	2175	2287	2276	2434
K07216 hemerythrin	626	642	676	606	338	308	337	340	262	289	283	238
K07217 Mn-containing catalase	463	520	545	510	354	372	395	340	744	780	737	729
K07218 nitrous oxidase accessory protein	1827	1714	1758	1564	961	865	773	911	624	543	537	478
K07219 putative molybdopterin biosynthesis protein	0	0	2	0	3	2	5	2	0	4	0	4
K07222 putative flavoprotein involved in K+ transport	2279	2366	2180	2135	1466	1474	1353	1514	709	694	607	692
K07225 putative hemin transport protein	2322	2358	2153	2288	1378	1279	1301	1293	1427	1395	1317	1476
K07226 heme iron utilization protein	1199	1151	1215	1165	648	714	652	678	358	338	372	409
K07227 heme iron utilization protein	8	5	5	7	14	7	10	11	42	55	45	43
K07231 putative iron-regulated protein	1967	2007	1901	1973	1091	1110	1206	1155	683	654	589	633
K07233 copper resistance protein B	2608	2645	2695	2725	1736	1688	1613	1659	842	751	757	821
K07234 uncharacterized protein involved in response to NO	1631	1487	1484	1488	810	804	904	977	486	413	447	461
K19342 copper chaperone NosL	869	817	710	763	434	411	401	451	202	204	220	230
ko99979 Unclassified viral proteins	4131	4319	4105	4358	5006	5091	5218	5191	5250	5289	5013	5052
K22014 terminase small subunit	1	0	0	0	4	1	3	0	0	0	2	0
Poorly characterized	394582	394437	389152	395148	359198	360214	355286	358810	343574	343691	341481	343943
ko99996 General function prediction only	93819	93815	91925	93860	80950	80897	78999	79586	79302	80043	78278	79660
K00389 putative membrane protein	378	285	338	348	230	210	225	189	666	599	599	602
K00479 glycine betaine catabolism A	3533	3594	3602	3485	2233	2074	1985	2096	1139	1107	1096	1198
K01421 putative membrane protein	912	839	887	909	321	344	298	324	1037	1233	1009	1170
K02348 ElaA protein	1113	1091	1113	1129	656	600	576	661	660	682	685	693
K02351 putative membrane protein	3	0	0	0	0	0	0	0	0	0	0	0
K02426 cysteine desulfuration protein SufE	1331	1257	1285	1319	1701	1683	1654	1692	1632	1540	1631	1618
K02441 GlpG protein	1897	1839	1691	1816	1111	1101	981	1124	1127	1124	1029	1101
K02442 membrane protein GlpM	438	389	338	344	213	184	194	210	146	100	93	123
K02617 phenylacetic acid degradation protein	202	216	227	188	59	46	44	48	404	391	373	422

K02742 SprT protein	1159	1083	1038	1093	657	666	653	607	594	695	728	696
K03095 SprT-like protein	23	16	34	27	6	3	11	5	31	28	38	30
K03112 DamX protein	2064	2084	1929	2085	1264	1245	1323	1379	991	935	994	1017
K03558 membrane protein required for colicin V production	1241	1357	1209	1261	1629	1792	1354	1346	1262	1240	1155	1183
K03618 hydrogenase-1 operon protein HyaF	21	30	38	32	39	45	64	63	108	87	108	78
K03619 hydrogenase-1 operon protein HyaE	8	9	21	12	24	36	20	11	40	42	34	26
K03744 LemA protein	1475	1498	1458	1544	2529	2508	2683	2529	2197	2080	2309	2106
K03745 SlyX protein	436	444	410	453	258	234	273	257	247	287	272	325
K03747 Smg protein	298	274	284	277	178	220	185	239	360	401	413	412
K03748 SanA protein	356	385	326	423	278	255	241	258	509	654	536	554
K03749 DedD protein	783	787	782	734	429	484	518	543	593	604	533	565
K03791 putative chitinase	1389	1443	1487	1491	927	853	916	876	462	393	441	407
K03796 Bax protein	492	516	449	517	403	404	366	378	700	804	664	715
K03808 paraquat-inducible protein A	3258	3194	3157	3409	2140	2151	1888	2090	2472	2610	2456	2615
K03810 virulence factor	549	576	519	567	373	323	389	330	741	789	716	778
K03923 modulator of drug activity B	1244	1125	1124	1214	709	729	784	741	735	707	691	710
K03925 MraZ protein	1698	1663	1665	1643	1808	1878	1803	1779	1782	1740	1793	1710
K03931 putative isomerase	637	646	654	664	767	783	840	796	360	486	437	415
K03933 chitin-binding protein	1810	1668	1657	1658	992	935	899	935	610	540	471	546
K03975 membrane-associated protein	1920	1748	1879	1779	2261	2225	2164	2265	1820	1764	1872	1879
K04065 hyperosmotically inducible periplasmic protein	900	930	868	855	542	595	567	572	678	691	742	678
K04477 putative hydrolase	991	943	1037	1049	570	618	640	569	1056	1194	1013	993
K04750 PhnB protein	1675	1709	1601	1577	715	620	618	736	1050	1171	1103	1185
K04756 alkyl hydroperoxide reductase subunit D	0	2	0	0	0	0	0	0	0	2	0	2
K05594 ElaB protein	46	74	56	56	81	98	78	96	14	21	16	14
K05775 maltose operon periplasmic protein	452	531	544	560	413	388	374	355	725	711	662	697
K05805 CreA protein	718	660	696	695	424	534	433	465	597	585	617	593
K05811 putative lipoprotein	799	698	702	732	470	444	375	444	433	471	399	473
K05812 DTW domain-containing protein	2110	2001	1878	2008	1126	1163	1053	1229	1072	1092	1081	1060
K05997 Fe-S cluster assembly protein SufA	197	197	169	201	121	156	140	118	306	301	276	281
K06135 pyrroloquinoline quinone biosynthesis protein A	244	210	195	185	90	102	116	119	107	129	104	108
K06136 pyrroloquinoline quinone biosynthesis protein B	1697	1637	1504	1541	824	741	725	815	1046	980	990	1162
K06138 pyrroloquinoline quinone biosynthesis protein D	255	272	266	239	106	114	110	109	244	258	214	231
K06192 paraquat-inducible protein B	4639	4636	4686	4693	2797	2775	2701	2685	2472	2549	2346	2320
K06193 protein PhnA	864	799	823	907	575	573	538	477	530	589	533	614
K06197 cation transport regulator	132	127	111	146	78	85	82	100	206	179	199	224
K06198 competence protein CoiA	53	68	72	66	8	16	12	22	83	71	77	93
K06865 ATPase	0	0	0	1	3	5	1	2	7	22	7	12
K06866 autonomous glycyl radical cofactor	227	231	204	212	204	178	172	163	308	327	317	312
K06874 zinc finger protein	0	0	0	1	0	0	0	0	4	4	4	5
K06877 DEAD/DEAH box helicase domain-containing protein	886	931	883	858	160	162	193	155	902	1019	918	955
K06878 tRNA-binding protein	555	538	533	487	289	380	242	255	226	223	245	179
K06886 hemoglobin	6	11	10	10	20	18	13	13	8	13	14	14
K06994 putative drug exporter of the RND superfamily	8	2	1	9	6	6	2	5	4	7	6	8
K07001 NTE family protein	6584	6813	6893	6772	12020	12028	12229	11579	9088	8913	9215	9016
K07023 putative hydrolases of HD superfamily	1226	1136	1101	1146	546	534	456	460	736	720	783	760
K07025 putative hydrolase of the HAD superfamily	1324	1493	1579	1549	3336	3424	3383	3384	2852	2734	2790	2778
K07038 inner membrane protein	1179	1228	1176	1224	817	872	840	877	730	732	839	753
K07048 phosphotriesterase-related protein	351	342	326	330	114	113	145	139	888	1020	982	1068
K07049 TatD-related deoxyribonuclease	0	0	2	0	0	2	0	0	0	0	0	0
K07058 membrane protein	5215	5483	5440	5539	5226	5470	5380	5224	4915	4694	4739	4707
K07082 UPF0755 protein	3131	3188	3112	3198	3383	3299	3366	3320	3068	3022	3131	3120
K07100 putative phosphoribosyl transferase	2	0	0	0	0	1	0	0	0	0	6	0
K07113 UPF0716 protein FxsA	1176	1083	1088	1127	735	776	699	648	606	638	566	621
K07146 UPF0176 protein	1961	2001	1976	2029	1178	1194	1090	1031	1305	1335	1217	1318
K07153 high frequency lysogenization protein	1266	1250	1145	1312	782	707	786	735	820	787	795	755
K08276 ecotin	404	400	376	424	189	179	167	171	440	471	405	481
K08279 carnitine operon protein CaiE	118	116	125	144	192	181	153	152	30	47	32	49
K08971 putative membrane protein	0	0	0	0	0	3	0	1	1	11	3	9
K08972 putative membrane protein	16	11	13	17	6	7	7	13	17	33	17	26
K08973 putative membrane protein	662	692	747	724	412	362	413	416	256	216	196	239
K08974 putative membrane protein	443	424	459	353	120	124	162	134	400	452	362	456
K08976 putative membrane protein	2	0	0	0	0	0	0	0	0	0	0	0
K08979 putative membrane protein	0	0	0	1	0	2	0	2	13	12	7	0
K08981 putative membrane protein	18	19	9	14	18	7	8	10	21	25	29	28
K08982 putative membrane protein	0	0	1	1	0	1	0	0	0	0	1	3
K08983 putative membrane protein	688	694	586	653	397	398	362	403	205	211	171	221
K08984 putative membrane protein	3	3	3	1	14	8	8	9	40	50	31	39
K08985 putative lipoprotein	676	669	705	696	378	367	304	385	234	194	187	201
K08986 putative membrane protein	313	311	305	288	51	49	60	31	195	202	248	253
K08987 putative membrane protein	162	160	174	169	101	120	107	126	165	171	177	177
K08988 putative membrane protein	0	0	0	0	3	0	0	0	1	4	3	1
K08989 putative membrane protein	95	73	89	101	120	124	105	128	24	37	36	16
K08990 putative membrane protein	605	570	491	594	328	395	389	384	838	827	828	901
K08993 putative membrane protein	606	590	519	567	338	318	276	349	400	337	378	409
K08995 putative membrane protein	79	96	102	106	55	59	61	54	49	21	25	42
K08996 putative membrane protein	4	3	0	3	9	16	14	3	29	30	41	35
K09014 Fe-S cluster assembly protein SufB	1995	2364	2212	2199	4223	4345	4187	4201	3971	4212	4037	3922
K09015 Fe-S cluster assembly protein SufD	1659	1815	1875	1744	3837	3678	3737	3703	3412	3262	3336	3216
K09858 SEC-C motif domain protein	1021	970	960	968	568	586	551	582	597	633	526	633
K09914 putative lipoprotein	928	855	868	883	529	505	514	579	628	628	559	630
K11312 cupin 2 domain-containing protein	0	1	1	3	0	3	6	4	1	0	0	0
K11477 glc operon protein GlcG	530	537	581	556	318	271	282	335	235	206	214	203
K11739 bacteriophage N4 adsorption protein A	7	8	17	10	41	36	33	31	174	163	167	157
K11740 bacteriophage N4 adsorption protein B	17	18	14	23	46	27	20	23	157	165	153	125
K11811 arsenical resistance protein ArsH	1093	1123	1004	1099	743	725	682	769	301	269	278	298
K12147 acidic protein MsyB	225	245	200	217	164	167	166	135	323	418	306	298
K13255 ferric iron reductase protein FhuF	502	516	510	538	340	330	354	366	692	709	659	704
K13256 protein PsiE	253	289	276	276	227	215	177	219	374	412	364	361
K13918 glucarate dehydratase-related protein	860	859	792	847	566	614	554	590	1091	1137	1163	1232
K14591 protein AroM	508	462	429	520	297	368	368	267	650	731	680	733
K15640 uncharacterized phosphatase	8	5	6	5	2	0	0	2	61	73	56	74
K15724 iron-sulfur cluster insertion protein	764	855	739	764	443	517	469	455	481	515	455	543
K21740 reactive chlorine resistance protein B	1	0	0	0	1	2	3	2	11	10	11	14
K21741 reactive chlorine resistance protein C	5	2	0	6	10	16	3	8	32	34	37	32
K21832 glycine betaine catabolism B	1727	1682	1579	1531	973	814	860	912	537	571	495	501
K21833 dimethylglycine catabolism A	1775	1832	1682	1813	931	935	885	1001	615	555	528	545
K21834 dimethylglycine catabolism B	3510	3266	3198	3267	2006	1821	1662	1959	1090	1123	955	1050
ko99997 Function unknown	300763	300622	297227	301288	278248	279317	276287	279224	264272	263648	263203	264283
K00243 uncharacterized protein	1483	1516	1448	1528	2142	2226	2340	2258	1980	1965	1912	1853
K01163 uncharacterized protein	1441	1417	1491	1448	2236	2242	2396	2175	2108	2064	2123	1951
K05937 uncharacterized protein	3	4	3	7	2	2	1	2	4	18	2	4
K05952 uncharacterized protein	390	424	386	389	224	189	209	180	226	214	254	241
K05967 uncharacterized protein	1	1	0	1	0	0	0	0	0	0	0	0
K06867 uncharacterized protein	2371	2528	2373	2547	1431	1361	867	932	873	884	815	849
K06871 uncharacterized protein	3560	3440	3696	3687	3519	3558	3735	3478	6026	6306	6190	6409
K06872 uncharacterized protein	3978	3724	3738	3857	3261	3292	3076	3355	2593	2499	2479	2454
K06883 uncharacterized protein	0	0	0	3	0	0	0	0	0	0	0	2
K06884 uncharacterized protein	485	516	531	538	266	347	105	115	426	452	402	431
K06885 uncharacterized protein	1212	1430	1375	1334	2670	2778	2699	2749	2995	2984	2966	2866
K06887 uncharacterized protein	169	215	174	154	257	288	257	279	96	95	82	114
K06888 uncharacterized protein	683	665	729	702	158	161	154	166	568	659	638	666
K06889 uncharacterized protein	10587	10667	10374	10888	14616	14609	14657	14464	12566	12376	12847	11990
K06890 uncharacterized protein	703	845	814	824	545	532	580	534	806	849	781	791
K06893 uncharacterized protein	1033	1119	940	1031	523	529	527	560	316	322	264	321
K06894 uncharacterized protein	12152	12310	11708	12126	15286	15604	15473	15980	13858	13634	13518	13588
K06903 uncharacterized protein	590	611	618	617	303	319	313	323	188	215	184	187
K06904 uncharacterized protein	1362	1362	1504	1533	1186	1180	1180	1100	483	442	510	414
K06905 uncharacterized protein	1817	1828	1787	1852	926	1000	974	1043	1623	1884	1815	1688
K06906 uncharacterized protein	1584	1544	1462	1493	840	807	810	824	454	472	423	513
K06907 uncharacterized protein	2297	2290	2382	2347	1428	1429	1312	1394	2322	2620	2540	2481

K06908 uncharacterized protein	511	509	476	490	269	228	243	270	736	725	731	735
K06910 uncharacterized protein	850	867	786	836	625	640	617	642	1028	1079	1072	1178
K06911 uncharacterized protein	5847	5767	5670	5838	4365	4418	4279	4440	4522	4534	4368	4584
K06913 uncharacterized protein	0	2	0	0	0	2	0	2	8	8	1	11
K06915 uncharacterized protein	3344	3307	3187	3348	2009	1963	1911	1900	2261	2307	2248	2375
K06918 uncharacterized protein	567	480	481	526	500	551	407	432	643	637	597	603
K06921 uncharacterized protein	1733	1838	2036	1892	4446	4323	4594	4431	3663	3659	3855	3772
K06923 uncharacterized protein	1928	1927	1873	1917	797	869	700	828	957	1026	1047	959
K06926 uncharacterized protein	452	525	529	519	1338	1338	1423	1347	1173	1045	1144	1205
K06929 uncharacterized protein	254	249	238	227	150	201	163	146	295	384	343	344
K06933 uncharacterized protein	1	1	1	0	0	0	0	0	5	4	1	5
K06934 uncharacterized protein	87	72	60	56	13	16	10	20	200	177	162	235
K06936 uncharacterized protein	2	2	2	1	0	0	3	0	5	4	2	1
K06938 uncharacterized protein	1185	1132	1051	1104	629	628	602	629	500	509	484	489
K06940 uncharacterized protein	2704	2566	2445	2458	1359	1397	1297	1569	1475	1485	1411	1543
K06944 uncharacterized protein	0	0	0	0	2	1	0	3	0	7	7	10
K06946 uncharacterized protein	6	5	4	6	22	25	19	30	76	88	103	108
K06950 uncharacterized protein	1887	1934	2096	2064	2969	3024	3148	3049	2740	2931	2952	2906
K06951 uncharacterized protein	1	4	5	9	3	2	1	1	4	4	4	6
K06952 uncharacterized protein	3	0	0	3	3	7	5	0	20	31	39	49
K06953 uncharacterized protein	0	0	2	0	0	0	0	2	3	0	3	1
K06954 uncharacterized protein	477	435	386	423	158	155	184	188	741	774	739	775
K06955 uncharacterized protein	1047	1029	900	975	580	578	510	639	338	288	295	281
K06956 uncharacterized protein	937	901	902	936	602	606	635	568	1120	1276	1238	1283
K06960 uncharacterized protein	148	160	147	123	51	51	52	43	146	181	142	188
K06962 uncharacterized protein	71	46	62	69	14	14	42	24	116	127	107	136
K06971 uncharacterized protein	763	798	762	745	425	416	447	420	818	916	917	935
K06973 uncharacterized protein	790	854	911	894	1513	1432	1685	1535	1359	1432	1433	1431
K06975 uncharacterized protein	1069	1058	1107	1110	1616	1594	1558	1644	1353	1328	1447	1276
K06976 uncharacterized protein	98	103	80	106	23	13	33	20	137	120	111	133
K06977 uncharacterized protein	0	0	1	0	0	0	0	0	0	0	0	0
K06978 uncharacterized protein	326	357	385	363	330	394	375	350	131	100	146	128
K06986 uncharacterized protein	706	700	749	728	481	379	404	423	287	246	243	227
K06987 uncharacterized protein	2541	2451	2387	2302	1178	1129	1059	1150	1299	1327	1316	1406
K06991 uncharacterized protein	687	740	646	706	383	412	349	383	206	201	183	232
K06992 uncharacterized protein	1687	1612	1542	1706	934	893	869	972	538	513	488	504
K06995 uncharacterized protein	2798	2641	2593	2526	1465	1348	1349	1473	931	969	885	987
K06996 uncharacterized protein	955	920	968	1051	1179	1195	1226	1171	1036	942	962	963
K07000 uncharacterized protein	1940	1845	1809	1923	1110	1102	1116	1029	1311	1396	1383	1384
K07002 uncharacterized protein	881	850	776	798	489	599	554	602	225	210	181	208
K07003 uncharacterized protein	7973	7894	7822	8010	4085	4156	3903	4126	3108	2915	2845	2815
K07004 uncharacterized protein	3116	3113	2998	3019	1747	1750	1465	1767	1007	910	895	976
K07005 uncharacterized protein	496	558	623	615	1198	1139	1204	1133	1081	1011	951	1003
K07006 uncharacterized protein	1736	1648	1607	1599	867	816	791	871	966	952	941	1014
K07013 uncharacterized protein	8	6	8	9	0	1	2	3	8	10	9	15
K07014 uncharacterized protein	3808	3593	3561	3795	2189	2222	2069	2202	2255	2178	2194	2201
K07015 uncharacterized protein	297	245	282	277	98	92	109	89	341	350	298	337
K07017 uncharacterized protein	1307	1334	1139	1309	661	674	551	773	951	950	938	1041
K07018 uncharacterized protein	431	445	431	425	255	229	260	243	120	135	141	108
K07019 uncharacterized protein	1958	1857	1790	1844	1117	1279	1191	1190	1245	1237	1204	1256
K07020 uncharacterized protein	841	737	821	827	460	373	400	456	285	223	225	231
K07028 uncharacterized protein	2353	2265	2318	2368	1391	1203	1312	1469	782	679	815	764
K07030 uncharacterized protein	826	832	819	777	269	287	291	301	722	893	872	835
K07032 uncharacterized protein	687	716	677	675	391	401	360	375	267	229	235	227
K07033 uncharacterized protein	36	44	38	41	14	14	11	8	69	89	87	74
K07034 uncharacterized protein	397	407	371	381	248	284	305	276	555	508	516	541
K07035 uncharacterized protein	648	639	657	673	188	197	233	188	686	760	652	718
K07039 uncharacterized protein	1159	1241	1135	1249	736	708	764	776	834	815	827	850
K07040 uncharacterized protein	1315	1409	1336	1428	748	760	738	762	820	960	928	885
K07041 uncharacterized protein	2	2	2	1	3	2	0	3	7	8	10	10
K07043 uncharacterized protein	1561	1647	1732	1588	2274	2304	2311	2288	2102	2119	2124	2021
K07044 uncharacterized protein	3255	3246	3188	3165	1827	1726	1740	1865	1058	1021	1020	974
K07045 uncharacterized protein	811	759	816	804	196	222	211	175	730	707	698	767
K07051 uncharacterized protein	0	0	0	0	0	2	2	0	6	4	3	8
K07052 uncharacterized protein	3292	3395	3413	3393	3982	4016	4095	4156	3248	3091	3206	3182
K07054 uncharacterized protein	571	534	518	571	358	395	468	485	786	690	720	815
K07063 uncharacterized protein	0	1	0	0	0	0	0	0	0	0	1	1
K07068 uncharacterized protein	590	639	595	630	321	325	313	321	191	214	156	200
K07069 uncharacterized protein	53	45	63	60	17	19	16	20	83	110	105	85
K07070 uncharacterized protein	592	622	558	586	370	384	328	369	324	301	284	343
K07071 uncharacterized protein	2763	2663	2723	2707	3612	3519	3570	3654	3074	2946	3041	2970
K07074 uncharacterized protein	1896	1962	1939	1911	1185	1218	1062	1168	660	671	648	705
K07075 uncharacterized protein	765	876	869	821	2671	2545	2762	2725	1919	1626	1912	1785
K07076 uncharacterized protein	1165	1103	1119	1020	575	537	509	569	344	298	264	332
K07077 uncharacterized protein	0	0	2	3	3	2	0	0	1	1	2	11
K07078 uncharacterized protein	1602	1448	1606	1527	2045	2198	2134	2041	1433	1441	1456	1351
K07079 uncharacterized protein	2023	2069	2072	2219	3016	3101	3096	2971	3658	3984	3827	3840
K07080 uncharacterized protein	1249	1186	1150	1204	878	871	888	890	426	437	372	442
K07088 uncharacterized protein	3769	3890	3932	3869	2273	2405	2225	2317	2808	2804	2700	2945
K07089 uncharacterized protein	1765	1656	1733	1728	1084	1208	1173	1242	1720	1742	1848	1965
K07090 uncharacterized protein	11714	11633	11092	11548	5844	5696	5631	6211	5590	5571	5584	5720
K07092 uncharacterized protein	556	521	470	454	272	261	260	309	156	142	149	146
K07093 uncharacterized protein	228	234	243	245	114	126	133	131	91	75	62	67
K07095 uncharacterized protein	2032	2117	1982	2062	1898	1897	1826	1912	2001	2113	2106	1970
K07096 uncharacterized protein	23	19	23	27	4	3	8	3	14	14	4	17
K07097 uncharacterized protein	2	0	0	1	0	0	0	0	0	0	0	1
K07098 uncharacterized protein	1825	1845	1871	1834	2937	2991	3085	2911	2353	2319	2404	2393
K07099 uncharacterized protein	61	83	70	70	69	50	54	43	90	78	82	111
K07101 uncharacterized protein	16	12	19	19	38	39	50	39	53	40	31	30
K07103 uncharacterized protein	0	0	0	1	0	0	0	0	4	4	0	2
K07105 uncharacterized protein	152	148	146	170	63	70	56	51	202	210	179	197
K07108 uncharacterized protein	2	0	2	0	1	0	0	2	0	1	3	1
K07109 uncharacterized protein	317	330	329	340	234	267	257	253	494	458	505	481
K07112 uncharacterized protein	4577	4539	4150	4447	2601	2659	2574	2743	2243	2249	2066	2317
K07118 uncharacterized protein	1423	1509	1433	1444	1924	1904	1883	1848	1432	1330	1402	1329
K07120 uncharacterized protein	1933	1878	1817	1858	918	958	841	921	1199	1283	1304	1268
K07121 uncharacterized protein	4163	4147	4027	4157	2503	2481	2475	2652	2498	2481	2603	2626
K07123 uncharacterized protein	0	0	0	1	0	0	0	0	2	12	8	12
K07124 uncharacterized protein	3631	3926	3571	3821	2209	2298	2176	2231	2013	1816	1887	1992
K07126 uncharacterized protein	7306	7349	7465	7358	6094	6059	4548	4553	4966	4559	4904	4715
K07129 uncharacterized protein	33	41	30	31	2	0	3	2	46	61	57	60
K07133 uncharacterized protein	9046	9618	10346	9509	25822	26048	26838	26319	20872	20053	21045	20721
K07134 uncharacterized protein	0	0	2	0	0	3	0	0	1	5	2	8
K07135 uncharacterized protein	0	0	0	0	0	0	0	1	0	2	1	5
K07136 uncharacterized protein	279	228	215	264	181	179	176	154	365	402	382	380
K07137 uncharacterized protein	2626	2652	2631	2723	3939	3854	3926	3597	4038	4113	4163	3974
K07138 uncharacterized protein	1019	945	959	949	607	542	668	615	987	1018	965	1073
K07139 uncharacterized protein	1616	1569	1622	1605	2582	2610	2760	2486	2677	2656	2640	2715
K07140 uncharacterized protein	1735	1832	1727	1731	953	1010	993	959	1240	1258	1230	1300
K07148 uncharacterized protein	1999	2244	2401	2238	5524	5498	5405	5481	4422	4278	4508	4277
K07149 uncharacterized protein	25	36	30	29	12	11	10	10	17	39	21	39
K07150 uncharacterized protein	914	830	907	873	658	657	611	610	1031	1163	1095	1192
K07157 uncharacterized protein	241	261	217	223	115	117	103	138	65	68	91	79
K07158 uncharacterized protein	0	0	2	0	0	0	0	0	2	1	3	2
K07159 uncharacterized protein	1	0	0	0	0	0	2	2	5	2	5	3
K07161 uncharacterized protein	0	2	1	2	0	0	0	0	6	2	2	7
K07164 uncharacterized protein	476	540	544	477	1556	1491	1540	1496	1110	1155	1140	1111
K07220 uncharacterized protein	1412	1403	1419	1507	1931	1798	1884	1752	1508	1499	1476	1367
K07269 uncharacterized protein	337	347	376	381	239	257	239	221	516	489	488	500
K07276 uncharacterized protein	0	0	0	1	0	0	0	0	0	2	2	0
K07338 uncharacterized protein	1762	1715	1875	1664	943	951	910	1009	538	584	574	561

K07502 uncharacterized protein	372	340	354	321	60	80	71	53	287	315	309	304
K07584 uncharacterized protein	181	168	166	174	62	57	47	47	161	165	140	146
K07586 uncharacterized protein	36	37	32	45	12	14	4	4	39	43	24	24
K07742 uncharacterized protein	185	184	185	203	57	73	64	48	141	184	147	167
K07807 uncharacterized protein	384	412	469	424	377	319	294	306	99	111	89	117
K08997 uncharacterized protein	884	854	797	857	640	647	590	546	1117	1178	1086	1191
K08998 uncharacterized protein	906	886	864	901	995	983	1002	1044	1027	1058	1013	1014
K08999 uncharacterized protein	380	358	423	396	1257	1338	1236	1251	873	864	862	875
K09003 uncharacterized protein	2	0	2	3	6	0	0	2	0	8	3	4
K09004 uncharacterized protein	70	69	69	53	9	16	9	18	48	62	50	48
K09005 uncharacterized protein	3	2	4	1	2	4	0	0	13	2	7	7
K09009 uncharacterized protein	2	3	0	1	0	2	1	6	8	2	1	4
K09116 uncharacterized protein	61	62	47	58	25	8	13	23	115	158	131	166
K09117 uncharacterized protein	934	902	965	935	1283	1375	1451	1383	1172	1264	1175	1169
K09118 uncharacterized protein	56	86	60	61	60	53	45	49	77	112	108	114
K09122 uncharacterized protein	0	0	0	0	0	0	0	0	2	0	0	0
K09124 uncharacterized protein	71	98	104	83	310	319	321	287	36	69	49	51
K09125 uncharacterized protein	1815	1888	1923	1901	2182	2134	2151	2276	1599	1607	1611	1614
K09126 uncharacterized protein	0	0	1	0	3	2	4	4	4	11	3	2
K09128 uncharacterized protein	88	98	99	118	39	35	29	38	169	179	169	186
K09129 uncharacterized protein	0	0	0	0	0	0	0	0	4	0	0	0
K09131 uncharacterized protein	536	560	547	588	336	362	346	347	362	325	294	329
K09133 uncharacterized protein	121	157	132	147	62	61	51	72	162	182	153	176
K09137 uncharacterized protein	14	8	15	10	0	0	0	0	27	26	35	29
K09138 uncharacterized protein	135	128	125	131	28	22	24	20	91	92	83	99
K09139 uncharacterized protein	2	0	0	0	3	0	0	0	0	5	2	4
K09141 uncharacterized protein	5	6	8	5	5	1	3	2	4	22	18	17
K09142 uncharacterized protein	0	0	0	0	0	0	0	0	0	2	4	3
K09144 uncharacterized protein	0	0	2	0	0	0	3	2	6	7	2	7
K09145 uncharacterized protein	23	41	31	35	3	4	3	10	82	68	66	80
K09154 uncharacterized protein	0	0	0	0	0	0	0	0	3	7	3	4
K09155 uncharacterized protein	155	172	145	159	108	109	114	112	61	93	74	81
K09157 uncharacterized protein	848	881	860	874	378	419	388	397	956	1033	994	992
K09158 uncharacterized protein	576	582	600	610	372	376	343	408	336	428	373	379
K09160 uncharacterized protein	1005	938	925	1004	572	587	558	512	655	609	565	657
K09161 uncharacterized protein	309	269	287	313	176	227	233	183	415	461	416	466
K09163 uncharacterized protein	48	44	42	54	7	9	11	17	75	89	83	116
K09164 uncharacterized protein	2	2	2	0	0	2	0	0	0	1	0	1
K09167 uncharacterized protein	2	6	2	7	3	2	4	6	13	14	4	12
K09190 uncharacterized protein	0	0	0	0	0	0	0	2	0	0	0	0
K09384 uncharacterized protein	43	40	40	47	22	13	17	10	60	89	77	109
K09386 uncharacterized protein	0	0	0	0	0	0	0	2	3	0	0	6
K09701 uncharacterized protein	467	424	431	464	284	308	287	310	577	602	578	603
K09702 uncharacterized protein	307	333	324	316	294	285	252	238	348	402	343	418
K09703 uncharacterized protein	197	175	219	204	35	32	41	28	225	275	257	257
K09704 uncharacterized protein	3013	3094	3329	3077	9166	9174	9448	8972	8143	7748	7916	7290
K09705 uncharacterized protein	115	139	104	136	30	31	28	22	88	99	82	96
K09706 uncharacterized protein	1	3	0	1	0	3	0	0	1	0	6	4
K09707 uncharacterized protein	202	188	152	175	37	18	20	24	200	192	174	179
K09711 uncharacterized protein	1	0	1	1	0	1	0	1	0	0	2	7
K09712 uncharacterized protein	137	143	125	135	74	73	54	84	189	221	216	193
K09713 uncharacterized protein	0	0	0	0	0	0	0	0	2	0	0	12
K09717 uncharacterized protein	0	4	0	0	0	3	2	0	8	7	2	3
K09721 uncharacterized protein	0	0	0	1	0	0	0	0	0	2	0	1
K09724 uncharacterized protein	0	2	0	2	2	4	2	2	5	13	4	11
K09726 uncharacterized protein	0	0	0	0	0	0	0	0	5	1	6	12
K09727 uncharacterized protein	0	2	2	0	0	0	0	0	1	0	0	4
K09728 uncharacterized protein	0	0	0	0	0	1	0	0	0	0	0	0
K09729 uncharacterized protein	26	44	37	39	3	2	7	2	54	51	48	58
K09730 uncharacterized protein	0	0	0	0	0	0	5	0	1	5	2	3
K09735 uncharacterized protein	0	0	0	0	1	0	2	0	2	5	2	5
K09737 uncharacterized protein	0	0	0	0	0	0	0	0	1	0	0	0
K09738 uncharacterized protein	0	0	2	0	0	0	0	0	0	2	0	3
K09740 uncharacterized protein	9	4	16	13	15	13	19	10	17	32	31	22
K09747 uncharacterized protein	942	918	882	931	489	512	416	510	757	751	778	745
K09749 uncharacterized protein	345	347	379	372	84	113	115	78	308	340	361	391
K09762 uncharacterized protein	622	526	564	502	160	165	203	166	432	599	576	482
K09763 uncharacterized protein	0	0	0	0	0	0	0	1	0	0	0	0
K09764 uncharacterized protein	10	14	12	10	13	18	19	18	18	24	24	28
K09766 uncharacterized protein	1	2	0	1	0	0	0	0	0	1	2	2
K09768 uncharacterized protein	1317	1268	1344	1296	691	733	690	775	904	858	912	953
K09769 uncharacterized protein	126	89	92	98	57	90	76	72	90	122	78	132
K09770 uncharacterized protein	0	0	0	3	0	0	0	0	2	0	0	2
K09775 uncharacterized protein	261	192	216	257	58	64	73	81	182	170	192	183
K09776 uncharacterized protein	0	0	0	0	0	0	0	0	0	0	2	0
K09777 uncharacterized protein	39	37	51	37	24	28	40	18	53	64	69	66
K09779 uncharacterized protein	139	122	164	145	50	49	45	37	122	123	145	176
K09780 uncharacterized protein	631	614	571	598	295	355	363	387	384	339	405	425
K09781 uncharacterized protein	1105	1072	1012	1048	635	656	634	698	834	829	725	765
K09786 uncharacterized protein	2519	2526	2440	2578	1503	1506	1463	1563	1569	1587	1621	1609
K09787 uncharacterized protein	186	191	224	201	60	70	57	63	163	183	177	203
K09790 uncharacterized protein	1022	1001	998	918	1124	1167	1180	1119	1085	978	1049	986
K09791 uncharacterized protein	368	403	335	329	261	211	183	214	269	287	251	224
K09792 uncharacterized protein	326	334	315	312	146	184	84	101	97	83	92	82
K09793 uncharacterized protein	230	224	217	243	656	627	935	885	576	431	571	514
K09794 uncharacterized protein	380	347	323	351	213	178	183	188	116	103	94	126
K09795 uncharacterized protein	2	1	2	0	2	2	3	1	1	0	3	2
K09797 uncharacterized protein	206	227	219	249	144	128	149	163	227	256	246	233
K09798 uncharacterized protein	1872	1868	1783	1786	1085	1143	1053	1087	1306	1269	1162	1250
K09799 uncharacterized protein	0	0	0	0	0	5	0	2	2	2	6	5
K09801 uncharacterized protein	678	575	588	586	401	364	414	411	314	328	315	352
K09802 uncharacterized protein	232	200	184	213	158	147	155	173	251	301	232	235
K09803 uncharacterized protein	28	33	21	31	5	4	3	10	7	12	26	15
K09804 uncharacterized protein	3	0	0	0	0	0	0	0	0	0	0	0
K09805 uncharacterized protein	8	14	9	9	12	5	8	4	33	48	26	34
K09806 uncharacterized protein	729	653	692	692	424	419	468	444	452	403	399	391
K09807 uncharacterized protein	1120	990	1052	1081	1796	1725	1784	1732	1815	1814	1759	1776
K09824 uncharacterized protein	671	693	719	755	660	634	628	628	763	817	739	864
K09857 uncharacterized protein	1608	1568	1444	1529	922	891	868	904	790	799	851	886
K09861 uncharacterized protein	2472	2540	2529	2480	2935	2941	2906	2865	2681	2641	2838	2772
K09862 uncharacterized protein	441	414	444	421	269	310	298	271	223	278	267	264
K09891 uncharacterized protein	379	340	375	389	263	291	273	233	537	506	522	499
K09894 uncharacterized protein	218	231	248	270	197	209	136	138	343	350	322	369
K09895 uncharacterized protein	508	540	517	538	329	397	337	331	483	606	539	608
K09896 uncharacterized protein	151	182	206	185	139	149	178	110	254	248	236	257
K09897 uncharacterized protein	282	230	238	274	183	216	187	164	313	338	325	355
K09898 uncharacterized protein	523	507	451	518	285	299	260	286	302	310	296	302
K09899 uncharacterized protein	235	211	202	248	175	177	183	177	323	415	373	392
K09900 uncharacterized protein	169	177	166	181	127	136	155	144	278	245	209	281
K09901 uncharacterized protein	172	186	160	191	105	135	115	129	274	250	198	294
K09902 uncharacterized protein	569	598	571	601	322	341	343	358	375	379	363	415
K09904 uncharacterized protein	180	185	152	195	120	121	115	102	269	291	267	270
K09907 uncharacterized protein	328	332	361	334	213	272	249	207	453	453	500	468
K09908 uncharacterized protein	1013	977	938	894	548	595	565	570	694	636	561	590
K09909 uncharacterized protein	133	145	136	134	197	231	231	187	18	37	40	27
K09910 uncharacterized protein	210	192	207	238	192	168	189	181	286	334	337	334
K09911 uncharacterized protein	242	238	247	276	198	192	158	140	322	325	371	351
K09912 uncharacterized protein	997	943	1030	964	564	546	527	507	651	640	641	684
K09915 uncharacterized protein	1001	995	859	988	591	584	585	636	710	755	747	738
K09916 uncharacterized protein	575	555	569	583	342	344	311	327	421	415	357	357
K09917 uncharacterized protein	98	92	101	114	63	87	88	62	151	183	150	136

K09918 uncharacterized protein	425	413	434	368	289	254	289	322	158	147	129	123
K09919 uncharacterized protein	1789	1696	1686	1757	963	929	923	949	553	504	513	554
K09920 uncharacterized protein	973	972	959	931	582	621	591	635	708	638	605	635
K09921 uncharacterized protein	1384	1332	1248	1366	796	822	827	856	970	927	828	944
K09922 uncharacterized protein	645	738	777	699	1003	957	1044	1010	662	746	577	602
K09923 uncharacterized protein	709	686	725	731	462	449	358	348	451	472	485	465
K09924 uncharacterized protein	875	887	772	793	515	498	448	500	302	263	263	278
K09925 uncharacterized protein	859	844	904	835	462	460	429	454	353	328	320	322
K09926 uncharacterized protein	744	733	715	704	417	368	384	442	229	213	235	227
K09927 uncharacterized protein	3202	3095	3065	3110	1426	1498	1397	1434	2130	2158	2226	2147
K09928 uncharacterized protein	396	431	414	441	211	256	209	219	100	119	119	136
K09929 uncharacterized protein	760	814	772	744	397	411	328	375	273	219	242	224
K09930 uncharacterized protein	1300	1410	1432	1323	795	784	686	659	385	347	443	426
K09931 uncharacterized protein	849	785	718	708	404	367	327	390	264	274	232	211
K09932 uncharacterized protein	834	803	766	750	374	417	331	414	597	598	595	620
K09934 uncharacterized protein	861	878	892	846	537	497	506	541	374	270	316	301
K09935 uncharacterized protein	1114	1059	1050	1074	588	527	535	573	498	517	462	503
K09937 uncharacterized protein	503	543	509	510	306	292	278	268	289	336	352	367
K09938 uncharacterized protein	1629	1615	1522	1608	910	801	824	912	546	466	521	476
K09939 uncharacterized protein	58	68	79	67	97	107	87	98	2	19	9	12
K09940 uncharacterized protein	669	672	724	733	351	358	348	406	217	224	201	206
K09945 uncharacterized protein	1104	1051	1073	1092	603	640	721	850	704	662	585	664
K09946 uncharacterized protein	620	583	575	641	351	310	348	367	249	192	203	249
K09947 uncharacterized protein	491	459	492	477	280	304	266	268	124	132	138	148
K09948 uncharacterized protein	477	448	377	429	195	232	240	238	168	116	122	140
K09949 uncharacterized protein	36	26	45	47	4	11	3	4	41	59	49	58
K09950 uncharacterized protein	1764	1737	1569	1729	905	885	928	970	601	564	452	548
K09954 uncharacterized protein	491	489	459	488	270	312	263	284	315	340	299	308
K09955 uncharacterized protein	7153	7561	7758	7547	12477	12339	12973	12959	9589	9705	9638	9490
K09956 uncharacterized protein	517	510	443	411	257	268	215	266	154	153	151	154
K09957 uncharacterized protein	491	459	510	471	269	239	230	285	149	155	145	142
K09958 uncharacterized protein	1139	1158	1030	1081	638	734	580	648	650	631	628	648
K09959 uncharacterized protein	0	3	0	3	0	0	2	0	2	0	0	0
K09960 uncharacterized protein	227	212	238	249	16	19	12	14	13	9	18	15
K09961 uncharacterized protein	941	1003	1069	970	278	300	260	273	39	41	40	34
K09962 uncharacterized protein	889	878	823	841	475	477	467	487	274	255	207	246
K09963 uncharacterized protein	550	616	605	537	104	114	128	115	452	496	509	449
K09964 uncharacterized protein	861	784	766	788	1062	1038	1031	1077	743	670	728	638
K09965 uncharacterized protein	807	765	655	733	466	381	354	382	196	213	220	248
K09967 uncharacterized protein	474	452	393	450	605	671	570	596	36	38	48	38
K09968 uncharacterized protein	210	198	203	217	89	89	68	78	670	581	568	646
K09973 uncharacterized protein	946	866	954	952	2136	2029	2159	1976	2037	1924	1894	2021
K09974 uncharacterized protein	865	839	734	730	392	428	366	480	240	302	273	273
K09975 uncharacterized protein	1502	1534	1409	1644	944	1054	936	986	785	816	757	767
K09976 uncharacterized protein	16	12	10	5	2	4	3	3	6	7	6	11
K09977 uncharacterized protein	528	461	427	477	252	279	209	264	174	138	130	161
K09978 uncharacterized protein	685	597	645	647	370	322	314	348	442	484	443	426
K09979 uncharacterized protein	279	258	282	285	198	208	201	218	347	402	374	377
K09980 uncharacterized protein	980	997	1013	1053	629	518	517	599	341	364	377	313
K09982 uncharacterized protein	251	287	264	322	180	201	179	186	227	255	223	280
K09983 uncharacterized protein	610	623	570	605	342	330	303	351	223	183	176	182
K09984 uncharacterized protein	525	489	442	542	295	253	277	339	245	289	267	278
K09985 uncharacterized protein	0	0	0	0	2	2	0	2	0	0	2	2
K09986 uncharacterized protein	918	917	866	907	477	464	502	446	323	276	265	263
K09987 uncharacterized protein	0	0	0	0	2	0	0	0	0	0	5	0
K09989 uncharacterized protein	1626	1675	1607	1613	918	870	887	977	604	505	538	492
K09990 uncharacterized protein	2	0	0	1	0	0	0	0	3	0	3	0
K09991 uncharacterized protein	1	0	0	1	1	0	0	0	2	0	2	3
K09992 uncharacterized protein	76	84	65	69	11	19	27	27	147	180	130	120
