# Supplementary material for: Trehalose-Induced Remodelling of the Human Microbiota Affects Clostridioides difficile Infection Outcome in an In Vitro Colonic Model: A Pilot Study
Source: Front Cell Infect Microbiol. 2021 Jul 2;11:670935. doi: 10.3389/fcimb.2021.670935 (PMC8284250; doi:10.3389/fcimb.2021.670935)
Supplement: Supplementary file 4 [file DataSheet_4.pdf]

Metabolic pathways				Fold change ratio	
Function	KEGG path	Gene		Model S vs	Model G vs
Galactose	Galactose	K00883	2-dehydro-3-deoxygalactonokinase [EC:2.7.1.58]	1.587493	2.69429
		K01631	2-dehydro-3-deoxyphosphogalactonate aldolase [EC:4.1.2.21]	1.678875	3.011981
		K07406	alpha-galactosidase [EC:3.2.1.22]	3.734462	7.211474
		K12111	evolved beta-galactosidase subunit alpha [EC:3.2.1.23]	4.686072	7.265716
Lactose to GA-3P		K00917	tagatose 6-phosphate kinase [EC:2.7.1.144]	2.29208	4.381098
		K01220	6-phospho-beta-galactosidase [EC:3.2.1.85]	1.647299	2.990219
		K01635	tagatose 1,6-diphosphate aldolase [EC:4.1.2.40]	2.458287	3.441174
		K01819	galactose-6-phosphate isomerase [EC:5.3.1.26]	2.075043	3.059144
myo-Inosit	Inositol ph	K00010	myo-inositol 2-dehydrogenase / D-chiro-inositol 1-dehydrogenase [EC:1.1	4.326005	9.006917
		K01093	4-phytase / acid phosphatase [EC:3.1.3.26 3.1.3.2]	3.493064	8.044779
		K03335	inosose dehydratase [EC:4.2.1.44]	3.754518	8.375601
		K03337	5-deoxy-glucuronate isomerase [EC:5.3.1.30]	4.140919	7.86438
		K03338	5-dehydro-2-deoxygluconokinase [EC:2.7.1.92]	3.600228	7.57371
		K06606	2-keto-myo-inositol isomerase [EC:5.3.99.11]	3.492312	8.38526
		K22230	scyllo-inositol 2-dehydrogenase (NADP+) [EC:1.1.1.-]	3.583864	2.457945
Generating	Starch and	K00690	sucrose phosphorylase [EC:2.4.1.7]	5.022313	8.257956
		K00691	maltose phosphorylase [EC:2.4.1.8]	3.710037	3.83521
		K01208	cyclomaltodextrinase / maltogenic alpha-amylase / neopullulanase [EC:3.	4.283956	7.522596
		K01188	beta-glucosidase [EC:3.2.1.21]	7.069215	5.285367
		K01838	beta-phosphoglucomutase [EC:5.4.2.6]	4.018841	3.325884
		K05350	beta-glucosidase [EC:3.2.1.21]	2.54099	3.212327
Trehalose	Starch and	K01226	trehalose-6-phosphate hydrolase [EC:3.2.1.93]	-2.39529	-1.94513
		K02818	PTS system, trehalose-specific IIB component [EC:2.7.1.201]	-2.4115	-2.04198
		K05342	alpha,alpha-trehalose phosphorylase [EC:2.4.1.64]	-1.49816	-1.82375
		K16055	trehalose 6-phosphate synthase/phosphatase [EC:2.4.1.15 3.1.3.12]	-1.32908	-4.96915
Chitin to A	Amino sug	K01183	chitinase [EC:3.2.1.14]	1.983814	3.427092
		K01207	beta-N-acetylhexosaminidase [EC:3.2.1.52]	2.097189	1.553362
		K01654	N-acetylneuraminate synthase [EC:2.5.1.56]	3.77916	3.705011
		K01788	N-acylglucosamine-6-phosphate 2-epimerase [EC:5.1.3.9]	1.859656	3.039027
		K18675	N,N'-diacetylchitobiose phosphorylase [EC:2.4.1.280]	5.460405	6.790055
		K18676	glucosamine kinase [EC:2.7.1.8]	7.421275	5.756097
Produces S	Glyoxylate	K01846	methylaspartate mutase sigma subunit [EC:5.4.99.1]	5.086169	12.20496
		K01848	methylmalonyl-CoA mutase, N-terminal domain [EC:5.4.99.2]	15.99163	20.46842
		K01849	methylmalonyl-CoA mutase, C-terminal domain [EC:5.4.99.2]	27.9749	33.59269
		K04835	methylaspartate ammonia-lyase [EC:4.3.1.2]	9.763312	27.48151
		K17865	3-hydroxybutyryl-CoA dehydratase [EC:4.2.1.55]	11.19641	33.191
		K19268	methylaspartate mutase epsilon subunit [EC:5.4.99.1]	9.766065	27.47419
		K19312	acetyl-CoA/propionyl-CoA carboxylase carboxyl transferase subunit [EC:6	7.910646	11.9903
L-glutamin	Butanoate	K01615	glutaconyl-CoA decarboxylase subunit alpha [EC:7.2.4.5]	-2.69954	-2.27102
		K07516	3-hydroxyacyl-CoA dehydrogenase [EC:1.1.1.35]	-3.99697	-5.29791
		K08318	4-hydroxybutyrate dehydrogenase / sulfolactaldehyde 3-reductase [EC:1.1	-1.64089	-4.77526
		K14534	4-hydroxybutyryl-CoA dehydratase / vinylacetyl-CoA-Delta-isomerase [EC:	-3.9676	-2.04285
		K16871	4-aminobutyrate---pyruvate transaminase [EC:2.6.1.96]	-5.40541	-8.51064
Produces a	Nitrogen n	K00260	glutamate dehydrogenase [EC:1.4.1.2]	2.109315	3.865464
		K00261	glutamate dehydrogenase (NAD(P)+) [EC:1.4.1.3]	3.3961	7.304646
		K00366	ferredoxin-nitrite reductase [EC:1.7.7.1]	4.062796	3.110691
		K01501	nitrilase [EC:3.5.5.1]	1.787174	3.67446
		K01674	carbonic anhydrase [EC:4.2.1.1]	3.081896	4.025432
		K00926	carbamate kinase [EC:2.7.2.2]	1.617142	1.513475
Ethanolam	Glyceroph	K03735	ethanolamine ammonia-lyase large subunit [EC:4.3.1.7]	1.691808	1.47497
		K03736	ethanolamine ammonia-lyase small subunit [EC:4.3.1.7]	1.768648	1.751357
		K04019	ethanolamine utilization protein EutA	3.544227	7.490263
	Amino acid	K04023	ethanolamine transporter	2.438122	3.770232
		K04024	ethanolamine utilization protein EutJ	3.154406	4.886354
		K04025	ethanolamine utilization protein EutK	3.239121	6.724775

		K04026	ethanolamine utilization protein EutL	3.206721	7.087652
		K04027	ethanolamine utilization protein EutM	3.47818	6.459127
		K04028	ethanolamine utilization protein EutN	3.542527	5.618845
		K04029	ethanolamine utilization protein EutP	1.789027	2.987424
		K04030	ethanolamine utilization protein EutQ	3.702804	6.308611
		K04031	ethanolamine utilization protein EutS	2.048016	3.15954
Arginine tc	Arginine ar	K01484	succinylarginine dihydrolase [EC:3.5.3.23]	1.724344	2.022483
		K05526	succinylglutamate desuccinylase [EC:3.5.1.96]	1.807067	2.082274
		K06447	succinylglutamic semialdehyde dehydrogenase [EC:1.2.1.71]	1.752154	1.51799
produces (	Phenylalar	K01713	cyclohexadienyl dehydratase [EC:4.2.1.51 4.2.1.91]	1.967308	1.546609
		K03856	3-deoxy-7-phosphoheptulonate synthase [EC:2.5.1.54]	2.607757	3.24743
		K04517	prephenate dehydrogenase [EC:1.3.1.12]	2.962624	3.335702
		K06209	chorismate mutase [EC:5.4.99.5]	2.98206	3.165561
D-ribose-1	Phosphona	K05780	alpha-D-ribose 1-methylphosphonate 5-triphosphate synthase subunit PhnL	2.083421	2.273532
		K06162	alpha-D-ribose 1-methylphosphonate 5-triphosphate diphosphatase [EC:3.6	2.080379	1.876615
		K06163	alpha-D-ribose 1-methylphosphonate 5-phosphate C-P lyase [EC:4.7.1.1]	1.880595	1.536351
		K06164	alpha-D-ribose 1-methylphosphonate 5-triphosphate synthase subunit PhnI	1.941859	1.756228
		K06165	alpha-D-ribose 1-methylphosphonate 5-triphosphate synthase subunit PhnH	2.025884	1.520809
		K06166	alpha-D-ribose 1-methylphosphonate 5-triphosphate synthase subunit PhnG	1.951639	1.631562
		K09994	(aminoalkyl)phosphonate N-acetyltransferase [EC:2.3.1.280]	2.948262	10.3865
Produces p	Purine met	K07127	5-hydroxyisourate hydrolase [EC:3.5.2.17]	1.706728	1.595742
		K07816	putative GTP pyrophosphokinase [EC:2.7.6.5]	3.365454	3.764947
	ko00121 Secondary bile acid biosynthesis			-2.42767	-1.50825

Cell surface components

Function	KEGG path	Gene		Fold change ratio<br>Model S vs	 Model G vs
Siderophoi	Nonriboso	K04784	yersiniabactin nonribosomal peptide synthetase	2.769361	4.963087
		K04785	yersiniabactin synthetase, thiazolinyl reductase component	3.409583	7.923863
		K04786	yersiniabactin nonribosomal peptide/polyketide synthase	3.37029	7.765072
		K05374	yersiniabactin synthetase, thioesterase component	2.834566	6.679182
	ABC transp	K19309	bacitracin transport system ATP-binding protein	9.804384	10.04391
		K19310	bacitracin transport system permease protein	11.45523	12.48316
Curli		K04334	major curlin subunit	-1.13901	-4.65615
		K04335	minor curlin subunit	-1.91161	-5.54011
		K04336	curli production protein	-1.73176	-4.27524
	Secretion s	K04337	curli production assembly/transport component CsgE	-1.53205	-5.22194
		K04338	curli production assembly/transport component CsgF	-1.71163	-6.69375
		K06214	curli production assembly/transport component CsgG	-1.86035	-5.50711
Biofilm for	Biofilm for	K11931	poly-beta-1,6-N-acetyl-D-glucosamine N-deacetylase [EC:3.5.1.-]	3.373616	7.402321
		K11935	biofilm PGA synthesis protein PgaA	3.244679	7.93057
		K11936	poly-beta-1,6-N-acetyl-D-glucosamine synthase [EC:2.4.1.-]	3.285213	7.440578
		K11937	biofilm PGA synthesis protein PgaD	2.919934	7.212479
Capsule synthesis		K10107	capsular polysaccharide transport system permease protein	-1.7477	-4.90795
		K09688	capsular polysaccharide transport system permease protein	-1.67487	-6.14188
		K09689	capsular polysaccharide transport system ATP-binding protein [EC:7.6.2.12]	-1.59517	-16.4309
	Structural	K07265	capsular polysaccharide export protein	-1.80732	-7.8585
		K07266	capsular polysaccharide export protein	-1.59474	-8.76681
	ABC transp	K02006	cobalt/nickel transport system ATP-binding protein	1.7052	2.473879
		K02007	cobalt/nickel transport system permease protein	1.894377	2.40947
		K02008	cobalt/nickel transport system permease protein	1.833957	2.414102
		K05813	sn-glycerol 3-phosphate transport system substrate-binding protein	1.820151	2.679094
		K05814	sn-glycerol 3-phosphate transport system permease protein	2.000044	2.94801
		K05815	sn-glycerol 3-phosphate transport system permease protein	2.223547	3.286714
		K05816	sn-glycerol 3-phosphate transport system ATP-binding protein [EC:7.6.2.10]	1.658849	2.564327
	ABC transp	K09692	teichoic acid transport system permease protein	3.643198	3.121338
		K09693	teichoic acid transport system ATP-binding protein [EC:7.5.2.4]	4.911506	4.901749

		K09970	general L-amino acid transport system permease protein	-1.56774	-2.82623
		K09971	general L-amino acid transport system permease protein	-1.83207	-3.45678
		K09972	general L-amino acid transport system ATP-binding protein [EC:7.4.2.1]	-1.81001	-4.23338
		K10117	raffinose/stachyose/melibiose transport system substrate-binding protein	5.265328	6.250168
		K10118	raffinose/stachyose/melibiose transport system permease protein	4.708741	5.741947
		K10119	raffinose/stachyose/melibiose transport system permease protein	4.745545	5.779807
		K10188	lactose/L-arabinose transport system substrate-binding protein	3.449079	7.095217
		K10189	lactose/L-arabinose transport system permease protein	4.024596	9.796145
		K10190	lactose/L-arabinose transport system permease protein	3.643447	8.003709
		K10192	oligogalacturonide transport system substrate-binding protein	4.241823	4.494798
		K10193	oligogalacturonide transport system permease protein	5.285135	4.667196
		K10194	oligogalacturonide transport system permease protein	5.409529	4.686637
		K10195	oligogalacturonide transport system ATP-binding protein	3.387397	6.681372
		K10240	cellobiose transport system substrate-binding protein	5.02591	8.870601
		K10241	cellobiose transport system permease protein	2.185786	2.883182
		K10242	cellobiose transport system permease protein	2.630931	3.858546
		K10439	ribose transport system substrate-binding protein	1.571418	1.729503
		K10440	ribose transport system permease protein	1.756013	1.855714
		K10441	ribose transport system ATP-binding protein [EC:7.5.2.7]	1.643021	1.459017
		K15580	oligopeptide transport system substrate-binding protein	2.165352	2.500601
		K15581	oligopeptide transport system permease protein	2.684989	3.261692
		K15582	oligopeptide transport system permease protein	2.992395	3.600072
		K15583	oligopeptide transport system ATP-binding protein	2.787353	3.125194
		K15770	arabinogalactan oligomer / maltooligosaccharide transport system substrate-bindi	1.935968	2.609366
		K15771	arabinogalactan oligomer / maltooligosaccharide transport system permease prote	2.296824	2.760161
		K15772	arabinogalactan oligomer / maltooligosaccharide transport system permease prote	2.111815	2.648875
		K17318	putative aldouronate transport system substrate-binding protein	4.39627	6.764396
		K17319	putative aldouronate transport system permease protein	4.623048	7.27761
		K17320	putative aldouronate transport system permease protein	4.77654	7.445924
T4SS	Bacterial s	K03194	type IV secretion system protein VirB1	3.260979	7.348808
		K03195	type IV secretion system protein VirB10	3.620563	7.839676
		K03196	type IV secretion system protein VirB11 [EC:7.4.2.8]	2.903913	6.996573
		K03197	type IV secretion system protein VirB2	4.03749	8.210343
		K03199	type IV secretion system protein VirB4 [EC:7.4.2.8]	3.320638	7.932874
		K03200	type IV secretion system protein VirB5	3.365035	7.999439
		K03201	type IV secretion system protein VirB6	3.347466	7.610748
		K03203	type IV secretion system protein VirB8	3.525881	7.262667
		K03204	type IV secretion system protein VirB9	3.159318	7.072796
		K03205	type IV secretion system protein VirD4 [EC:7.4.2.8]	1.859912	1.179242
Chemotaxi	Two-comp	K05874	methyl-accepting chemotaxis protein I, serine sensor receptor	-1.573	-16.9844
		K05875	methyl-accepting chemotaxis protein II, aspartate sensor receptor	-1.55356	-22.5789
		K05876	methyl-accepting chemotaxis protein III, ribose and galactose sensor receptor	-1.56306	-17.0911
		K05877	methyl-accepting chemotaxis protein IV, peptide sensor receptor	-1.40724	-18.0185
		K13816	DSF synthase	-1.47153	-16.7727
